# Regioselective Rearrangement of Nitrogen- and Carbon-Centered Radical Intermediates in the Hofmann–Löffler–Freytag Reaction

**DOI:** 10.1021/acs.jpca.3c07892

**Published:** 2024-03-22

**Authors:** Gabrijel Zubčić, Jiangyang You, Fabian L. Zott, Salavat S. Ashirbaev, Maria Kolympadi Marković, Erim Bešić, Valerije Vrček, Hendrik Zipse, Davor Šakić

**Affiliations:** † Faculty of Pharmacy and Biochemistry, University of Zagreb, Ante Kovačića 1, 10000 Zagreb, Croatia; ‡ Division of Physical Chemistry, Rud̵er Bošković Institute, Bijenička Cesta 54, 10000 Zagreb, Croatia; § Department of Chemistry, Ludwig-Maximilians-Universität München, Butenandtstrasse 5-13, D-81377 München, Germany; ∥ Faculty of Physics, and Centre for Micro- and Nanosciences and Technologies, University of Rijeka, Radmile Matejčić 2, 51000 Rijeka, Croatia

## Abstract

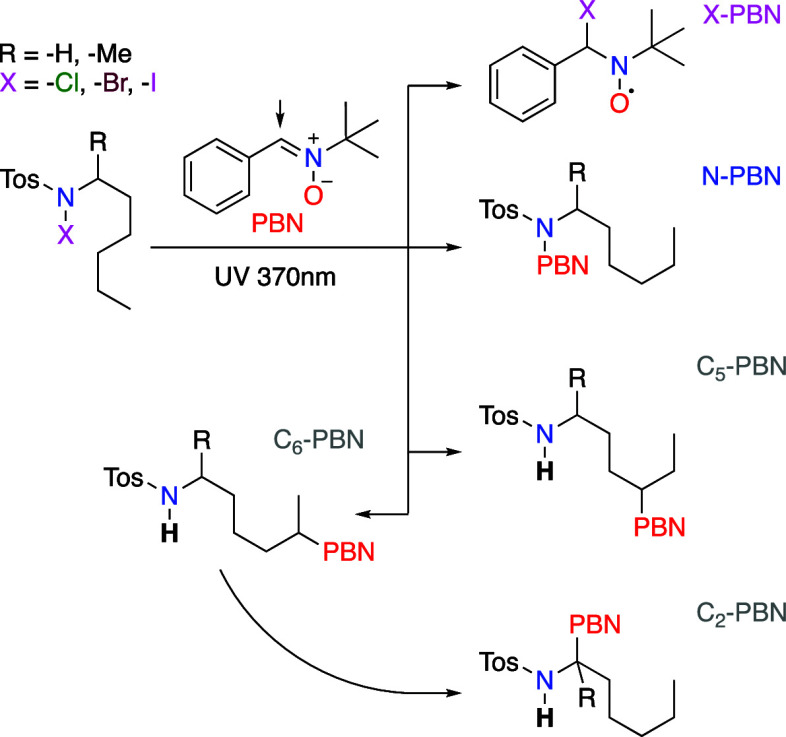

The Hofmann–Löffler–Freytag (HLF) reaction serves as a late-stage functionalization technique for generating pyrrolidine heterocyclic ring systems. Contemporary HLF protocols utilize in situ halogenated sulfonamides as precursors in the radical-mediated rearrangement cycle. Despite its well-established reaction mechanism, experiments toward the detection of radical intermediates using EPR techniques have only recently been attempted. However, the obtained spectra lack the distinct features of the N-centered radicals expected for the employed reactants. This paper presents phenylbutylnitrone spin-trapped C-centered and N-centered radicals, generated via light irradiation from N-halogen-tosyl-sulfonamide derivatives and detected using EPR spectroscopy. NMR spectroscopy and DFT calculations are used to explain the observed regioselectivity of the HLF reaction.

## Introduction

**Scheme 1:**
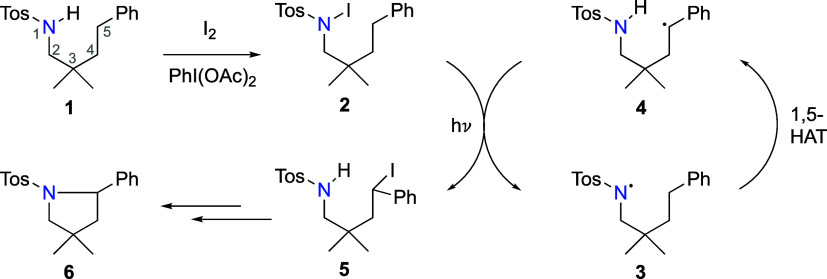
Modern HLF Reaction Sequence Involving Tosyl-Sulfonamides and In Situ Iodination with Iodine and a Co-Oxidant [PhI(OAc)_2_)]

Modern C–H functionalization chemistries have introduced late-stage functionalization (LSF) strategies in medicinal chemistry, targeting drug lead C–H bonds for creating new analogues. This toolbox includes photoredox-mediated and radical reactions and among them, amination reactions for the direct formation of C–N bonds.^1,2^ Recently, the focus is shifting from metal to organocatalytic protocols, paving the way to sustainability and adhering to green chemistry principles to minimize waste and improve yield and atom economy.^3^ This approach aligns with the EU’s sustainable development policy.^4,5^ Numerous research groups are exploring new C(sp^3^)-H functionalization reactions with high chemo-, regio-, and stereoselectivity. The Hofmann–Löffler–Freytag (HLF) reaction, used for building pyrrolidine (and in some cases, also piperidine) ring systems, is among photo-activated amination reactions without metal catalysis.^6,7^ The HLF reaction, first discovered in synthetic studies of N-haloamines,^8−11^ is a multistep process involving nitrogen atom activation through halogenation, N-centered radical generation via irradiation, intramolecular hydrogen atom transfer (HAT), and radical termination with cyclization to form the final C–N bond (Scheme 1).

**Figure 1:**
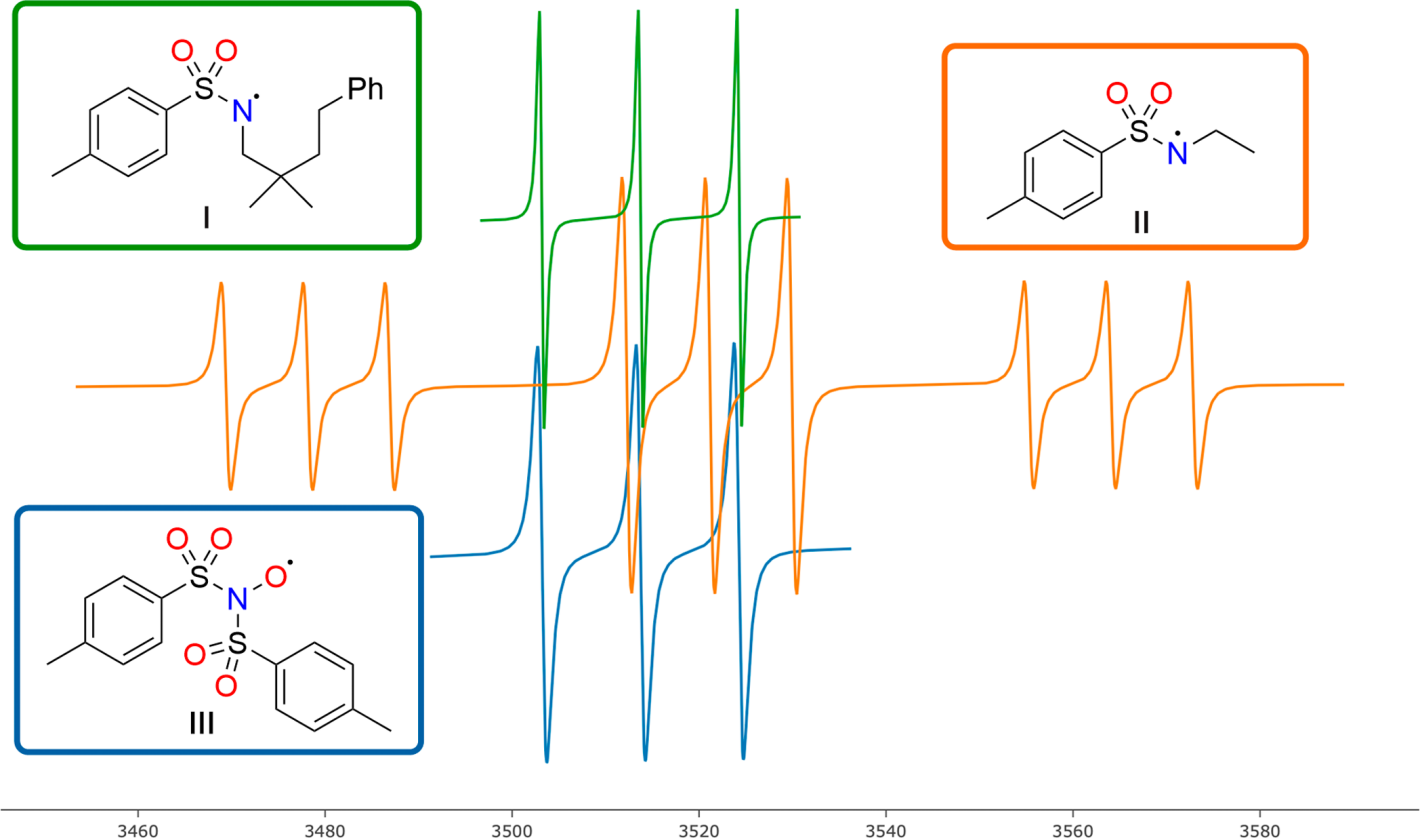
Simulated EPR spectra for tosyl-N-centered and ditosyl-N-centered radicals with the corresponding structures assigned by the authors. The green line is from EPR experiments,^12^ the orange line represents calculated EPR parameters,^13^ and the blue line is from EPR experiments.^14^

Contemporary adaptations of the HLF reaction employ toluenesulfonyl (tosyl, Tos)-activated amines (**1**), which undergo in situ iodination at the nitrogen atom (**2**) via an iodine source and a co-oxidant. The formation of an N-centered radical (**3**) was recently examined using EPR spectroscopy (Figure 1).^12^

However, the obtained spectra (**I**, in green), while presenting a triplet indicative of the nitrogen hyperfine splitting, raised questions due to issues such as broad line width, a high *g*-factor (2.0064) value for the proposed N-centered radical, and the absence of α-hydrogen splitting. Calculated EPR spectra for a model compound of **3** are shown in Figure 1 (**II**, orange line).^13^ The spectra of the ditosylated aminoxyl radical (**III**, in blue) fit with the EPR parameters of **I**, thus suggesting this species to be the correct assignment of the EPR spectra **I**.^14^ Neither C-centered radical (**4**) nor C_5_-iodo functionalized (**5**) intermediates were observed in the EPR and NMR studies. The only confirmed product in this reaction was the pyrrolidine ring compound (**6**). Experimental attempts for in situ generation of N-centered radicals and detection via time-resolved EPR included *N*-isopropyl-4-methoxybenzenesulfonamide as a radical precursor under electrochemical conditions.^15^ It is, however, likely that the detected radical is not an amidyl radical but a nitroxide radical instead. A reaction of the observed radical with 5,5-dimethyl-1-pyrroline-*N*-oxide (DMPO) did not occur, which is consistent with stable nitroxide radicals. The experimentally observed hyperfine coupling constant (hfc) value for hydrogen at C_2_ is significantly smaller than those in typical amidyl radicals but is similar to the calculated values for the nitroxide radicals.

Numerous synthetic studies utilizing HFL chemistry^16−20^ report the regioselective functionalization and subsequent formation of 5-membered pyrrolidine rings. In some especially rigid and/or previously functionalized systems, the formation of piperidines has been reported.^21−24^ The observed regioselectivity in HLF reactions occurs during the HAT phase and can result from two different pathways. The intramolecular mechanism, governed by the kinetic preference of 1,5- over 1,6-HAT steps, represents one pathway. Alternatively, an intermolecular route directs the product distribution based on the thermodynamic stability of the resulting C-centered radicals. Muñiz suggested the latter mechanism in the context of selective piperidine formation,^24^ where a phenyl-stabilized radical at the C_6_ position is formed from an N-centered radical in a bimolecular reaction and a pyrrolidine product was not observed. Between the three distinct pathways, it is unclear which one is dominant for a given set of reaction conditions, and detailed investigations are thus warranted.

## Materials and Methods

The purchased compounds were sourced from Kefo [sulfuric acid (98%), methanol, petroleum ether, *p*-toluenesulfonyl chloride, silica gel, pyridine, silver acetate, ethyl acetate, cyclohexane, trifluoroacetic anhydride, toluene, and trichloroisocyanuric acid], Ru-Ve [hydrochloric acid (37%), acetone, silicon oil, petroleum ether, and cyclohexane], and Biovit [toluene (anhydrous), acetonitrile (anhydrous), 1,4-dioxane (anhydrous), tetrahydrofuran (anhydrous), *N*,*N*-dimethylformamide (anhydrous), *N*,*N*-dimethylacetamide, 1,2-dichloroethane (anhydrous), and dichloromethane (anhydrous)]. All reagents and chemicals were obtained commercially and used without further purification, unless otherwise noted.

Moisture-sensitive reactions were performed using flame-dried glassware under a nitrogen atmosphere (N_2_). Air- and moisture-sensitive liquids and solutions were transferred with a plastic or glass syringe. Chromatographic purification of the products was carried out using column chromatography filled with silica gel (Macherey-Nagel) 0.063–0.2 mm, and appropriate solvent mixtures were used as eluents: petroleum ether/ethyl acetate. Thin-layer chromatography (TLC) was performed on precoated TLC plates ALUGRAM SIL G/UV254, 0.20 mm silica gel 60 with a fluorescent indicator UV254 (Macherey-Nagel) in the appropriate solvent system. TLC spots were observed after illumination with UV light at a wavelength of 254 nm and after immersion in an aqueous solution of KMnO_4_ (3 g KMnO_4_, 20 g K_2_CO_3_, 5 mL aq. NaOH 5%, and 300 mL water) followed by heating. If TLC spots were not visible after illumination with UV light, they were detected utilizing an iodine chamber.

Synthetic photocatalyzed reactions were performed in a custom-built photoreactor with built-in temperature control as well as standardized luminous intensity for a certain set of high-power LED light sources. The InGaN-based H2A1-420 LED (420 ± 20 nm) used in this experimental setup was purchased from Roithner Lasertechnik GmbH and mounted on a standard hexagonal aluminum package. Irradiation was performed in situ (EPR) and off-site (NMR) with Kessil PR-160L 370 ± 10 nm gen-2 LED UV, with an average intensity of 137 mW/cm^2^ when the sample is 6 cm from the lamp, according to the manufacturer.^25^

The reactant and products were identified using ^1^H NMR spectra, which were recorded on Varian Inova 400 and 600 machines in CDCl_3_ at 400 or 600 MHz at room temperature. All ^13^C NMR spectra were recorded, respectively, at 101 and 151 MHz. The chemical shifts are reported in ppm (δ), relative to the resonance of CDCl_3_ at δ = 7.27 ppm of ^1^H and for ^13^C, relative to the resonance of CDCl_3_ at δ = 77.16 ppm. NMR spectra of the reaction mixture were obtained on a Varian Inova 400 NMR spectrometer operating at 399.90 MHz for ^1^H NMR and 100.6 MHz for ^13^C NMR and are reported as chemical shifts (δ) in ppm. The spectra were imported and processed in the MestreNova 11.0.4 program.^26^

GC measurements were obtained at a Shimadzu GC-2010 Plus gas chromatograph with an AOC-20i autosampler (with a temperature-controlled sample holder) and an Optima 1701–0.25 μm (25 m × 0.25 mm) column.

HR-MS spectra were obtained using a Thermo Finnigan LTQ FT machine of the MAT 95 type with a direct exposure probe and electron impact ionization (70 eV).

EPR spectroscopy was done by using a Bruker E500 ELEXSYS EPR spectrometer with an ER4122SHQE cavity resonator. As this cavity resonator does not have an optical window for illumination, the light source was mounted underneath the cavity, with light coming through the bottom of the EPR 4 mm-inner-diameter tube. EPR deconvolution and simulation was done using an EasySpin module with the MATLAB program package.^27^ EPR visualization and spectroscopy were done using the VisualEPR Web page.^28^

The conformational space was sampled and investigated using the Conformer–Rotamer Ensemble Sampling Tool—CREST^29^ coupled with the xtb-GFN2 program package and MD simulation using xtb-GFN1^30^ and xtb-GFN2.^31^ The obtained structures were reoptimized using the B3LYP/6-31G(d) level of theory.^32−34^ For each structure with a stable wave function, frequency calculation was performed to identify the minima and transition-state structures. From the transition-state structure, an intrinsic reaction coordinate search was performed to characterize the corresponding reaction and product complexes/reactive conformers. Improved thermodynamics were obtained using RO-B2PLYP^35,36^ with a G3MP2 large basis set^37^ on geometries obtained at the B3LYP/6-31G(d) level of theory, with additional D3 dispersion correction.^38^ Additionally, for smaller and model systems, the G3B3^37^ composite method was used, with results matching the RO-B2PLYP-D3/G3MP2 large results.

Calculations of EPR parameters were done using the B3LYP functional and the mixed basis set: EPR-III for C, H, and O atoms, def2-QZVP for the S atom, and 6-31G(d) for the N atom. A small basis set on the N atom is necessary for the correct calculations of the *g*-factor and hfcs.^39,40^ When using a larger basis set for the N atom, e.g., EPR-III or def2-QZVP, the obtained results systematically underestimate the hfc. Calculations were performed on the Gaussian version 16.C01^41^ using the advanced computing service (clusters Isabella and Supek) provided by the University of Zagreb University Computing Centre—SRCE^42^ and the computational resources of the PharmInova project (sw.pharma.hr) at the University of Zagreb Faculty of Pharmacy and Biochemistry.^43^

## Results and Discussion

Insights into the HAT steps of the HLF reactions can be gained through the simulation of thermodynamic and kinetic parameters. The thermodynamics (driving force) of the reaction can be evaluated by comparing bond dissociation energies (BDEs) or by utilizing the radical stabilization energies (RSEs) of molecular fragments containing radical precursors and products (see below).^44−46^ Reaction kinetics are linked to thermodynamics via the Bell–Evans–Polanyi principle, a relationship that has been demonstrated for various HAT reactions for both inter- and intramolecular pathways.^47^ When the C_5_ and C_6_ positions are both unsubstituted as in substrate **7-H**, the regioselectivity toward the C_5_ product is experimentally evident even when the kinetic and thermodynamic parameters for 1,5- and 1,6-HAT align closely.^48^ There are no notable differences in energies vs the geometrical parameter (N–H–C angle) between 1,5- and 1,6-HAT energy profiles. Both of them resemble those of the intermolecular HAT (see Chart S1 in Supporting Information). There is room for unknown factors governing the regioselectivity of HLF reactions. To determine whether the transient N- and C-centered radicals influence the observed regioselectivity, we attempted in situ generation and trapping of these radical intermediates using the phenylbutylnitrone (PBN) spin trap and investigation of the resulting adducts with EPR. Additionally, the reaction progress was monitored using NMR techniques, with off-site light irradiation.

**Scheme 2:**
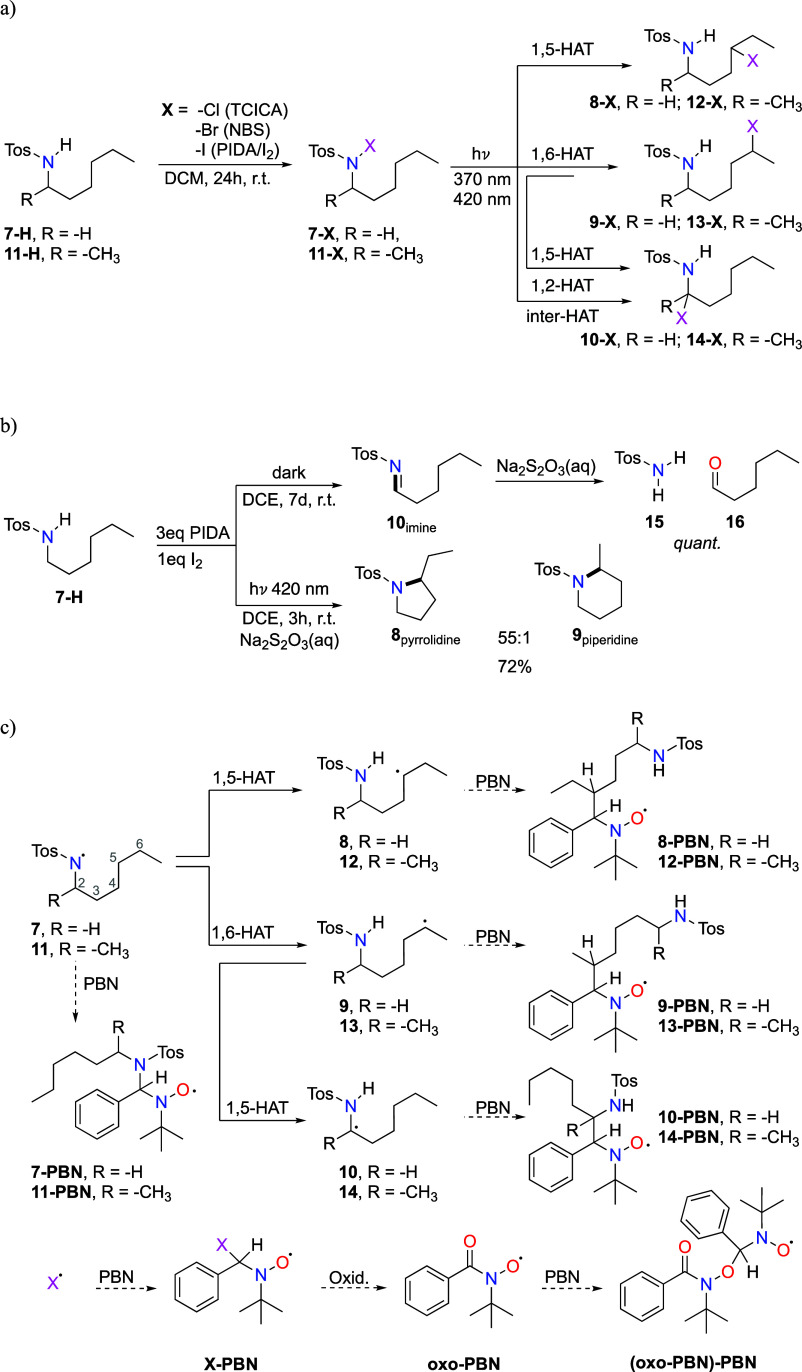
(a) Preparation of Precursors and Possible Intermediates in HLF Reactions of **7-H** and **11-H**, (b) Observed Products in the Reaction of **7-H** with PIDA/I_2_ under Dark and Irradiation Conditions, and (c) Investigated Reaction Sequences and Possible Products in Spin-Trapping Experiments

The same starting conditions reported earlier^12,16^ were employed for *N*-hexyl-4-methylbenzenesulfonamide (**7-H**) as the substrate. This reactant was chosen as the simplest model for unsubstituted C_5_ and C_6_ positions. The corresponding *N*-iodo (**7-I**) derivative was prepared in situ from **7-H** using hypervalent phenyliodine (III) diacetate (PIDA) as an oxidant with elemental I_2_ as the iodine source.^12,16^ After 3 h of irradiation of a reaction mixture containing 3 equiv of PIDA, 1 equiv of I_2_, and 1 equiv of **7-H** with a 420 nm light source, two distinct products could be observed (Scheme 2b). These products were identified through GC–MS and NMR techniques as a 55:1 mixture of the five-membered ring compound **8**_pyrrolidine_ and six-membered ring analogue **9**_piperidine_ in a combined yield of 72% (see Supporting Information). When the same reactant mixture was stored in darkness for 7 days, the crude ^1^H NMR spectrum revealed the unexpected presence of imine **10**_imine_ together with 4-methylbenzenesulfonamide (**15**) and hexanal (**16**). Subsequent processing with aqueous sodium thiosulfate resulted in near-quantitative recovery of compound **15**.

With these results in hand, an attempt was made to identify transient intermediates by monitoring reaction progress with NMR techniques. The mixture of **7-H**/PIDA/I_2_ was therefore irradiated off-site for 5 min (370 nm), followed by ^1^H NMR measurements during a full reaction sequence (see Supporting Information). In addition to signals of the starting material, two sets of new signals appeared, which is consistent with the formation of two products. These two cannot be clearly assigned, but the observed signal motifs and chemical shift values support the formation of two halogenated structures, one of them being the expected C_5_-halogenated product **8-I**. The assignment of the other product was not possible without ambiguity but clearly did not correspond to the C_6_-halogenated product **9-I**, or **10**_**imine**_, observed previously. To our knowledge, this is the first reaction monitoring HLF with NMR spectroscopy, with a large signal from excess PIDA covering the aromatic region of the spectra.

After NMR experiments, we monitored the complete reaction sequence with EPR spectrometry. Using continuous irradiation with the same UV lamp from the bottom of the cavity resonator, compound **7-I** was EPR-silent. This is in stark contrast to the published results,^12^ but as mentioned before, radicals observed in that experiment might stem from different oxidation pathways and rearrangement reactions that are not part of the HLF sequence.

**Figure 2:**
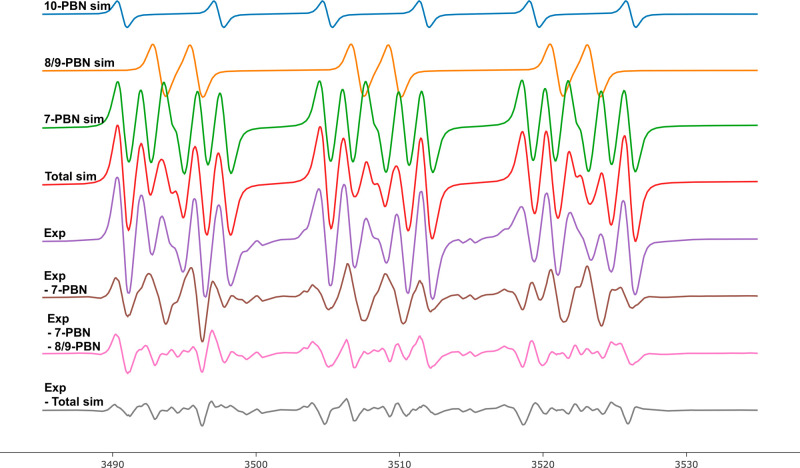
EPR spectra of spin-trapped radical intermediates generated with 370 nm irradiation of **7-Br**. The experimental spectrum is in purple, while blue, orange, green, and red correspond to simulated spectra for **10-PBN**, **8/9-PBN**, **7-PBN**, and total simulated spectra, respectively. Residuals from subtracted simulation from experimental spectra are at the bottom. More information on deconvolution and simulation is deposited in Supporting Information.

Next, we tried to capture nascent radicals from the reaction with PBN, but oxidizing and halogenating species present in the mixture reacted with PBN and produced an **oxo-PBN** (acylaminoxyl) radical, with α_N,exp_ = 8.0 G, as well as additional **(oxo-PBN)-PBN** adducts (see Figure 2).^49^ To summarize, we have not been able to detect short-lived radical intermediates of the HLF reaction using this procedure.

As in situ halogenation inhibits PBN’s ability to spin-trap radicals, the preparation of **7-Cl** and **7-Br** was performed in a separate step. While **7-Cl** was easily isolated and proved stable for a couple of days,^50^**7-Br** had to be synthesized, cleaned, isolated, and measured without any delay. Another major point is the sensitivity of the reaction to air. Line widths measured with EPR and reaction yields were greatly influenced by the effectiveness of air removal using freeze–pump–thaw cycles with backfill of argon or nitrogen gas. Experimental line widths of less than 0.4 G were deemed satisfactory for optimal resolution of radical adducts. Under these conditions with illumination with 370 nm light, we were able to observe a **Cl-PBN** adduct, proving homolytic cleavage of N–Cl bonds generating a chlorine radical that quickly combines with PBN (see Figure S20 in Supporting Information). From both **7-Cl** and **7-Br**, a PBN adduct **7-PBN** (*g*_exp_ = 2.0064, α_N,exp_ = 14.14 G, α_N′,exp_ = 1.58 G, and α_H,exp_ = 3.95 G) formed from an N-centered radical **7** was detected for the first time using EPR spectroscopy, proving that this is the correct method for investigating the HLF reaction and corresponding intermediates (see Figure 2). Calculated EPR parameters for **7-PBN** (*g*_**7**-**PBN**,calc_ = 2.00616, α_**7**-**PBN**,N,calc_ = 13.81 G, α_**7**-**PBN**,N′,calc_ = 1.52 G, and α_**7**-**PBN**,H,calc_ = 2.89 G) are in satisfactory agreement with experimental values.

In the EPR spectrum (see Figure 2), one signal corresponding to the two PBN adducts of C-centered radicals was expected, namely, C_5_ radical (**8**) and C_6_ radical (**9**). PBN adducts of those radicals, **8-PBN** and **9-PBN**, have similar calculated hfcs and *g*-factors (*g*_**8**-**PBN**,calc_ = 2.00595, α_**8**-**PBN**,N,calc_ = 15.09 G, and α_**8**-**PBN**,H,calc_ = 2.36 G and *g*_**9**-**PBN**,calc_ = 2.00597, α_**9**-**PBN**,N,calc_ = 14.80 G, and α_**9**-**PBN**,H,calc_ = 2.27 G), and it is thus difficult to distinguish between them, due to both being secondary alkyl C-centered radicals with similar environments around the radical center. A signal with *g*_exp_ = 2.0061, α_N_ = 13.84 G, and α_H_ = 2.47 G was observed that can be assigned to both **8-PBN** and **9-PBN**. The unexpected result was the formation and detection of PBN adduct **10-PBN**. It is characterized with *g*_exp_ = 2.0064, α_N_ = 13.80 G, and α_H_ = 7.34 G, which is different from the previously described radicals (more information about deconvolution can be found in the Supporting Information). The **10-PBN** radical adduct can be tentatively assigned as a C_2_ radical (**10**) due to having an hfc from N and H atoms in PBN and to different connectivities closer to the radical center, although it has an unusually high hfc value for a C-centered radical. To confirm this assignment, extensive computational analyses were performed. The calculated Boltzmann averaged values for **10-PBN** (*g*_**10**-**PBN**,calc_ = 2.00610, α_**10**-**PBN**,N,calc_ = 14.08 G, and α_**10**-**PBN**,H,calc_ = 5.46 G) demonstrated a trend similar to the experimental parameters, with significantly larger hfc values than computed for radicals **8/9-PBN**. A detailed analysis of the structural factors contributing to these values revealed interactions between the Tos-N(alkyl)-H and the O–N–PBN radical center, significantly impacting the α_H_ value in the lowest lying minima. Conformers of **10-PBN**, where this interaction is not present, are higher in energy by more than 10 kJ/mol from the global minima and have calculated α_**10**-**PBN**,H,calc_ 2.40 G, which is almost the same as that for radicals **8/9-PBN**. For more details on deconvolution and structure analysis, please consult Supporting Information.

From theoretical predictions calculated at the RO-B2PLYP-D3/G3MP2-large//B3LYP/6-31G(d) level of theory,^47,51^ the N-centered radical **7** rearranges in HAT steps with Δ*H*_298_^‡^ = +35.4 and +31.2 kJ/mol for 1,5-HAT and 1,6-HAT steps to C_5_-centered radical **8** and C_6_-centered radical **9** with reaction enthalpies Δ*H*_rx,298_ of −11.4 and −10.8 kJ/mol, respectively (see Supporting Information). Radical **10** can be produced through different HAT reactions: (a) intermolecular HAT from any N-centered or C-centered radical, (b) intramolecular from an N-centered radical (**7 → 10**) via 1,2-HAT_NC_, (c) intramolecular from C_5_ radical (**8 → 10**) via 1,4-HAT_CC_, and (d) intramolecular from C_6_ radical (**9 → 10**) via 1,5-HAT_CC_. Intermolecular HAT (**7-H** + **7 → 10** + **7-H**), involving neutral molecule (**7-H**) and N-centered radical (**7**), proceeds with a thermodynamic driving force of Δ*H*_rx,298_ = −50.6 kJ/mol and a predicted barrier of Δ*H*_298_^‡^ = 22.1 kJ/mol (Δ*G*_298_^‡^ = 37.2 kJ/mol from reaction complex). Intramolecular 1,2-HAT, a direct transformation from N-centered radical to C_2_ radical (**7 → 10**), has a predicted barrier of Δ*H*_298_^‡^ = 148.8 kJ/mol with a strong thermodynamic driving force of Δ*H*_rx,298_ = −32.3 kJ/mol, making it a kinetically prohibitive process.

**Figure 3:**
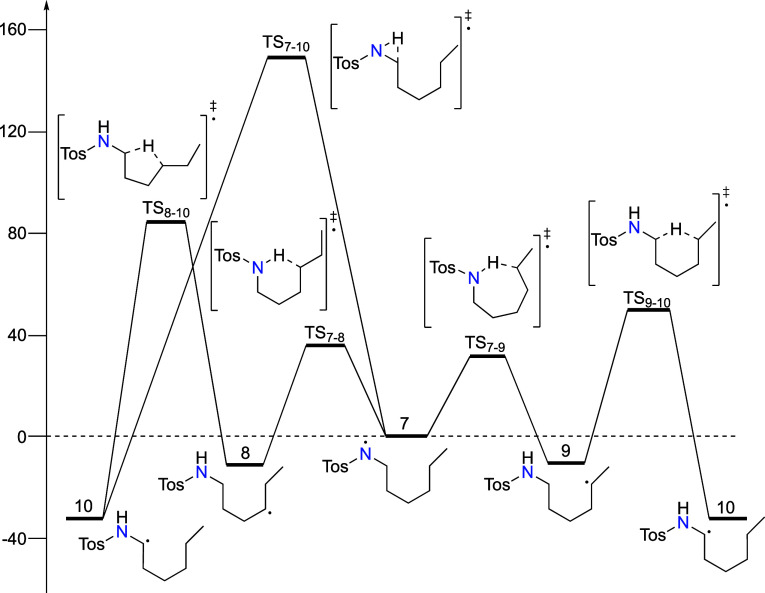
Energy diagram of intramolecular radical rearrangements from **7** calculated at RO-B2PLYP/G3MP2Large//B3LYP/6-31G(d).

Rearrangement to a C_2_ radical can occur from less stable distant C-centered radicals (**8 → 10** and **9 → 10**). Both inter- and intramolecular reactions are feasible with the latter (**7-H** + **8 → 10** + **7-H** and **7-H** + **9 → 10** + **7-H**) being characterized with Δ*H*_298_^‡^ = 33.5 kJ/mol and a thermodynamic driving force of Δ*H*_rx,298_ = −23.4 kJ/mol. This was calculated with the propane/propyl radical reference system as a reasonable model for the distant C-centered radicals in the hydrocarbon chain, due to negligible differences in reactivity and stability between the C_5_ radical, C_6_ radical, and propyl radical (RSEs and TS) toward the C_2_ radical. As seen in Figure 3, the barrier for intramolecular 1,4-HAT_CC_ rearrangement of the C_5_ radical to the C_2_ radical (**8 → 10**) is prohibitively high (Δ*H*_298_^‡^ = 84.1 kJ/mol), while 1,5-HAT_CC_ from the C_6_ radical to the C_2_ radical (**9 → 10**) proceeds through a 6-membered transition state with Δ*H*_298_^‡^ = 56.1 kJ/mol and a reaction enthalpy of Δ*H*_rx,298_ = −21.5 kJ/mol. These results indicate that this second intramolecular rearrangement, with the lowest energy of activation, is the probable origin of the experimentally observed regioselectivity in HLF reactions. It significantly decreases the lifetime of the C_6_ radical required for halogen atom abstraction and thus the yield of C_6_-functionalized products.

**Figure 4:**
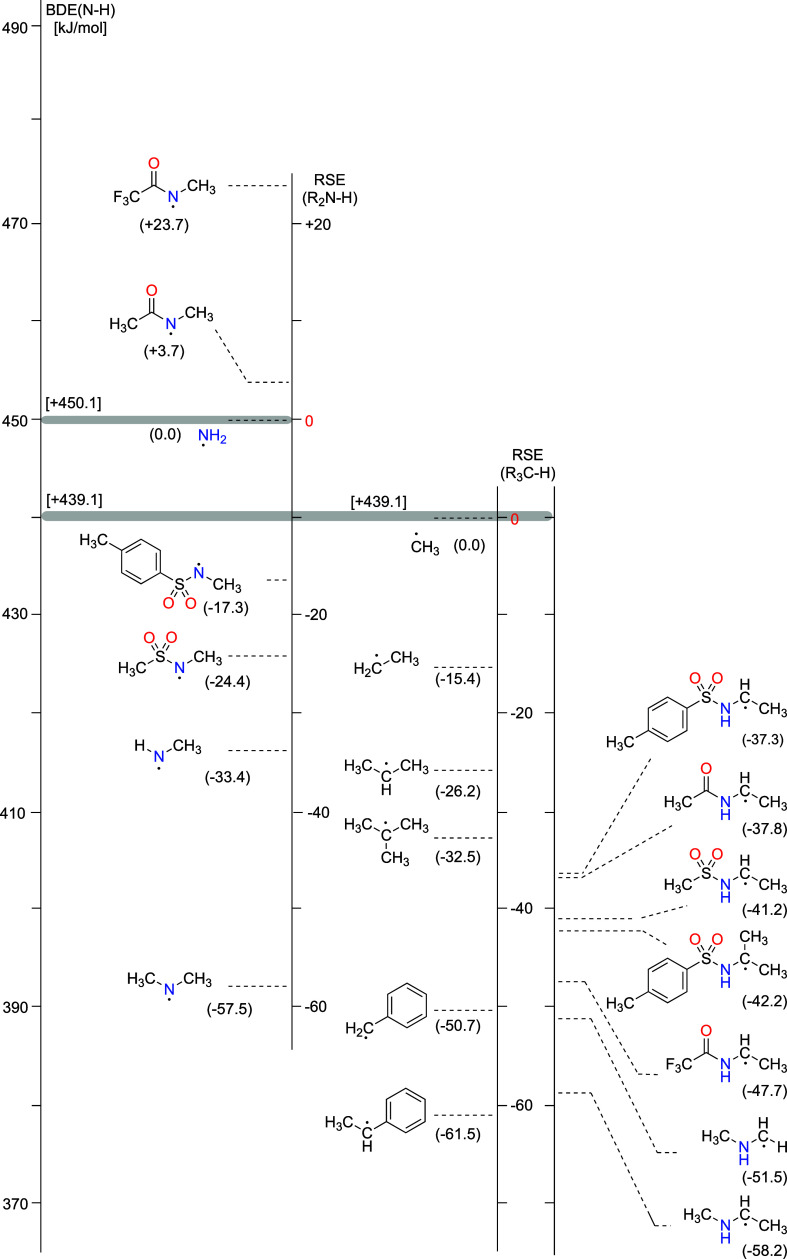
RSEs for selected N- and C-centered radicals present in systems **7**–**14**. The gray bands denote the anchor points for N-centered and C-centered radical RSE scales to a global BDE scale.

As mentioned before, different radical fragments present in systems **7**–**14** can be used to gauge the thermodynamic driving force for rearrangement reactions (see Figure 4).^44−47,51^ In our system, going from a tosylated methylamine radical (**7**) to a secondary C-centered radical (**8** and **9**) corresponds to an exothermic reaction (Δ*H*_predict,298_ = −19.8 kJ/mol, see Supporting Information). Additionally, rearrangement from a secondary C-centered radical to a tosylamide-substituted C-centered radical fragment such as **10** is also predicted to be exothermic (see Supporting Information), which is in line with our experimental observation.

The same rationale can be used to explain the regioselectivity reported in a recent study^52^ with different sulfonamide derivatives. While radical generation was achieved using visible light photoredox catalysis with Ru(bpy)_3_Cl_2_ in conjunction with blue LEDs, high yields and regioselectivity toward 1,5-HAT_NC_ rearrangement were observed when C_5_ and C_6_ positions were secondary and primary radicals. This stems from a notable difference in the radical stability (ΔRSE_prim/sec_ = 10.8 kJ/mol). When additional substituents at the C_6_ position are introduced to generate a (stabilized) tertiary C_6_ radical, a mixture of a 56% C_5_ product and a 40% C_6_ product is obtained (ΔRSE_sec/tert_ = 6.3 kJ/mol), making both 1,5-HAT_NC_ and 1,6-HAT_NC_ equivalent reactions. Proof of the existence of a C_2_ radical in HLF-type reactions is available in the literature. It was observed indirectly as an imine side product in electrochemically driven N-centered radical generation.^53^ The proposed generation of imine was either via elimination of HBr from an N-brominated precursor or via 1,2-HAT from a transient N-centered radical. The possible sequence of 1,6-HAT_NC_ followed by 1,5-HAT_CC_ was not explored.

In the ^1^H NMR spectra of the reaction mixture obtained after off-site irradiation of the **7-Cl** precursor, only two (main) products were observed and measured in the same proportion. The two pairs of triplets in the upfield and downfield regions are consistent with product structures **10-Cl** and **12-Cl** (see Supporting Information), which supports the formation of C_5_- and C_6_-radical intermediates, the latter undergoing a 1,5-H shift to the respective C_2_-centered radical. The same NMR results were obtained after irradiation of precursor **7-Br**.

To further investigate the second rearrangement process, we opted for a methyl-substituted derivative, *N*-(hept-2-yl)-4-methylbenzenesulfonamide (**11-H**, Scheme 2), which is a precursor for a more stable C_2_-centered radical [RSE(propyl/propane) = −26.2 kJ/mol vs RSE(*tert*-butyl/*tert*-butane) = −32.5 kJ/mol]. After chlorination to **11-Cl**, and sequential off-site irradiation, two products were observed in the NMR spectra. One product corresponds to the C_5_-chlorinated product **12-Cl**,^54^ without formation of a pyrrolidine ring (see Supporting Information).^55^ The C_6_-chlorinated product, **13-Cl**, was not observed, although the calculated barriers and driving forces are again similar (Δ*H*_298_^‡^ = +31.5 kJ/mol and +36.1 kJ/mol; Δ*H*_rx,298_ = −13.0 kJ/mol and −9.4 kJ/mol, for 1,5-HAT and 1,6-HAT, respectively). The second product is the C_2_-chlorinated product, **14-Cl**, with a characteristic triplet at 0.81 ppm (see Supporting Information). The lack of formation of **13-Cl** (or a piperidine ring product)^56^ indicates that **13** is a reactive intermediate, that quickly rearranges to **14**, which is converted to **14-Cl**. This is supported by calculations with the expected barrier for the second step 1,5-HAT rearrangement between C_6_ and C_2_ carbon centers of Δ*H*_298_^‡^ = 57.6 kJ/mol and Δ*H*_rx,298_ = −18.1 kJ/mol.

In the EPR spectra, when **11-Cl** was illuminated, a chlorine PBN adduct was formed (**Cl-PBN**), alongside N-centered radical adduct **11-PBN**. Both **7-PBN** and **11-PBN** have similar hfcs, namely, *g*_**11**-**PBN**,exp_ = 2.0062, α_**11**-**PBN**,N,exp_ = 14.1 G, α_**11**-**PBN**,N′,exp_ = 1.3 G, and α_**11**-**PBN**,H,exp_ = 4.21 G. Those results fit nicely with the calculated results (see Supporting Information). As expected, the C-centered radical signal corresponds well to the C_5_- and C_6_-radical adducts **12/13-PBN**, with the same hfc values as observed for **8/9-PBN** (*g*_**12/13**-**PBN**,exp_ = 2.0061, α_**12/13**-**PBN**,N,exp_ = 13.6 G, and α_**12/13**-**PBN**,H,exp_ = 2.6 G). The tentatively proposed C_2_-radical **14**, similar to radical **10**, made an adduct with PBN, **14-PBN** with a characteristic signal at *g*_**14**-**PBN**,exp_ = 2.0061, with hfc α_14-PBN,N,exp_ = 13.7 G and α_**14**-**PBN**,H,exp_ = 6.4 G, confirming our hypothesis that this species stems from rapid rearrangement from **13**. Again, a rather high hfc value for α_**14**-**PBN**,H_ can be attributed to the interaction between the sulfonamide hydrogen moiety and the oxygen-centered radical of the PBN in the thermodynamically stable conformers. Extensive calculations on radical adducts confirm the assignments (see Supporting Information). Similar results were obtained with **11-Br**, although the most pronounced peaks were **12/13-PBN. 11-PBN** was weak and **14-PBN** is not directly visible, possibly overshadowed by **11-PBN**. The lack of full correspondence between **11-Cl** and **11-Br** is most likely due to the instability of **11-Br**, which was already decomposing in the dark (see Supporting Information). We plan to continue the investigation on the weak components of the EPR spectrum deconvolution, currently assigned as **10**/**14-PBN**. Additional compounds with different substitution patterns on the C_5_ and C_6_ positions, with added modifications on C_2_ (blocking the route) and C_3_ positions (steric hindrance), will further test the origin of regioselectivity in HLF reactions.

## Conclusions

Using NMR and EPR spectroscopy in combination with DFT calculations, we successfully monitored the reaction profile and identified all significant intermediate radicals and products in the HLF reaction. The initial rearrangement step allows the N-centered radical to transform into both C_5_ and C_6_ radicals. The observed regioselectivity favoring 1,5-HAT products can be attributed to an additional rearrangement reaction exclusive to the C_6_ radical. Through another 1,5-HAT step, the C_6_ radical is transformed into the most stable C_2_ radical. The existence of C_2_ radical is experimentally proved not only through EPR spectroscopy but also via synthetic reactions and side products, notably imine and aldehyde. Future research should extend to more complex systems with additional substituents on the radical chain and varying functional groups, applying the methodology outlined in this study.

## Supporting Information Available

Experimental details, synthesis procedure, reactant and product characterization, EPR simulation parameters, calculation procedures, geometries and energies of optimized structures, and recorded NMR and EPR spectra (PDF)

## References

[ref1] Sinha S. K.; Guin S.; Maiti S.; Biswas J. P.; Porey S.; Maiti D. Toolbox for Distal C-H Bond Functionalizations in Organic Molecules. Chem. Rev. 2021, 122 (6), 5682–5841. 10.1021/acs.chemrev.1c00220. 34662117

[ref2] Roy S.; Panja S.; Sahoo S. R.; Chatterjee S.; Maiti D. Enroute Sustainability: Metal Free C-H Bond Functionalisation. Chem. Soc. Rev. 2023, 52 (7), 2391–2479. 10.1039/D0CS01466D. 36924227

[ref3] Flerlage H.; Slootweg J. C. Modern Chemistry Is Rubbish. Nat. Rev. Chem 2023, 7 (9), 593–594. 10.1038/s41570-023-00523-9. 37524899

[ref4] EUR-Lex—52019DC0640—EN—EUR-Lex; European Commision: Brussels, 2019; pp 1–24. https://eur-lex.europa.eu/legal-content/EN/TXT/?uri=COM%3A2019%3A640%3AFIN (accessed 2023-11-21).

[ref5] EUR-Lex—52020DC0667—EN—EUR-Lex; European Commision: Brussels, 2020; pp 1–24. https://eur-lex.europa.eu/legal-content/EN/TXT/?uri=COM%3A2020%3A667%3AFIN (accessed 2023-11-21).

[ref6] Pratley C.; Fenner S.; Murphy J. A. Nitrogen-Centered Radicals in Functionalization of Sp2 Systems: Generation, Reactivity, and Applications in Synthesis. Chem. Rev. 2022, 122 (9), 8181–8260. 10.1021/acs.chemrev.1c00831. 35285636

[ref7] Kwon K.; Simons R. T.; Nandakumar M.; Roizen J. L. Strategies to Generate Nitrogen-Centered Radicals That May Rely on Photoredox Catalysis: Development in Reaction Methodology and Applications in Organic Synthesis. Chem. Rev. 2022, 122 (2), 2353–2428. 10.1021/acs.chemrev.1c00444. 34623809 PMC8792374

[ref8] Hofmann A. W. Ueber Die Einwirkung Des Broms in Alkalischer Lösung Auf Die Amine. Ber. Dtsch. Chem. Ges. 1883, 16 (1), 558–560. 10.1002/cber.188301601120.

[ref9] Hofmann A. W. Ueber Die Einwirkung Des Broms in Alkalischer Lösung Auf Amide. Ber. Dtsch. Chem. Ges. 1881, 14 (2), 2725–2736. 10.1002/cber.188101402242.

[ref10] Hofmann A. W. Zur Kenntniss Der Coniin-Gruppe. Ber. Dtsch. Chem. Ges. 1885, 18 (1), 109–131. 10.1002/cber.18850180126.

[ref11] Löffler K.; Freytag C. Über Eine Neue Bildungsweise von N-alkylierten Pyrrolidinen. Ber. Dtsch. Chem. Ges. 1909, 42 (3), 3427–3431. 10.1002/cber.19090420377.

[ref12] Bosnidou A. E.; Duhamel T.; Muñiz K. Detection of the Elusive Nitrogen-Centered Radicals from Catalytic Hofmann-Löffler Reactions. Eur. J. Org Chem. 2020, 2020 (40), 6361–6365. 10.1002/ejoc.201900497.

[ref13] Korth H. Comment on “Detection of the Elusive Nitrogen-Centered Radicals from Catalytic Hofmann-Löffler Reactions. Eur. J. Org Chem. 2020, 2020 (40), 6366–6367. 10.1002/ejoc.201900922.

[ref14] Balakirev M. Yu.; Khramtsov V. V. ESR Study of Free Radical Decomposition of N,N-Bis(Arylsulfonyl)Hydroxylamines in Organic Solution. J. Org. Chem. 1996, 61 (21), 7263–7269. 10.1021/jo960427h. 11667648

[ref15] Liu Y.; Shi B.; Liu Z.; Gao R.; Huang C.; Alhumade H.; Wang S.; Qi X.; Lei A. Time-Resolved EPR Revealed the Formation, Structure, and Reactivity of N-Centered Radicals in an Electrochemical C(Sp3)-H Arylation Reaction. J. Am. Chem. Soc. 2021, 143 (49), 20863–20872. 10.1021/jacs.1c09341. 34851107

[ref16] Fan R.; Pu D.; Wen F.; Wu J. δ and α SP3 C-H Bond Oxidation of Sulfonamides with PhI(OAc)2/I2 under Metal-Free Conditions. J. Org. Chem. 2007, 72 (23), 8994–8997. 10.1021/jo7016982. 17929871

[ref17] Becker P.; Duhamel T.; Stein C. J.; Reiher M.; Muñiz K. Cooperative Light-Activated Iodine and Photoredox Catalysis for the Amination of C-H Bonds. Angew. Chem., Int. Ed. 2017, 56 (27), 8004–8008. 10.1002/anie.201703611. PMC5499658 28488354

[ref18] Teng X.; Yu T.; Shi J.; Huang H.; Wang R.; Peng W.; Sun K.; Yang S.; Wang X. Recent Advances in the Functionalization of Remote C-H Bonds by Hofmann-Löffler-Freytag-type Reactions. Adv. Synth. Catal. 2023, 365 (19), 3211–3226. 10.1002/adsc.202300718.

[ref19] Guo W.; Wang Q.; Zhu J. Visible Light Photoredox-Catalysed Remote C-H Functionalisation Enabled by 1,5-Hydrogen Atom Transfer (1,5-HAT). Chem. Soc. Rev. 2021, 50 (13), 7359–7377. 10.1039/D0CS00774A. 34013927

[ref20] Zubčić G.; Shkunnikova S.; Šakić D.; Marijan M. Renesansa Hofmann-Löffler-Freytag Reakcije - Razvoj C-H Funkcionalizacijskih Strategija Po Principima Zelene Kemije. Kem. Ind. 2022, 71 (5–6), 359–373. 10.15255/KUI.2021.070.

[ref21] Short M. A.; Blackburn J. M.; Roizen J. L. Modifying Positional Selectivity in C-H Functionalization Reactions with Nitrogen-Centered Radicals: Generalizable Approaches to 1,6-Hydrogen-Atom Transfer Processes. Synlett 2020, 31 (02), 102–116. 10.1055/s-0039-1691501. 33986583 PMC8115226

[ref22] Bafaluy D.; Muñoz-Molina J. M.; Funes-Ardoiz I.; Herold S.; de Aguirre A. J.; Zhang H.; Maseras F.; Belderrain T. R.; Pérez P. J.; Muñiz K. Copper-Catalyzed N-F Bond Activation for Uniform Intramolecular C-H Amination Yielding Pyrrolidines and Piperidines. Angew. Chem., Int. Ed. 2019, 58 (26), 8912–8916. 10.1002/anie.201902716. 30997949

[ref23] Muñoz-Molina J. M.; Bafaluy D.; Funes-Ardoiz I.; De Aguirre A.; Maseras F.; Belderrain T. R.; Pérez P. J.; Muñiz K. Mechanistic Studies on the Synthesis of Pyrrolidines and Piperidines via Copper-Catalyzed Intramolecular C-H Amination. Organometallics 2022, 41, 1099 10.1021/ACS.ORGANOMET.2C00095. 35572769 PMC9092462

[ref24] Zhang H.; Muñiz K. Selective Piperidine Synthesis Exploiting Iodine-Catalyzed Csp3-H Amination under Visible Light. ACS Catal. 2017, 7 (6), 4122–4125. 10.1021/acscatal.7b00928.

[ref25] Kessil PR-160L 370-Gen2 specification. https://www.kessil.com/products/science_PR160L.php (accessed 2023-12-01).

[ref26] Willcott M. R. MestRe Nova. J. Am. Chem. Soc. 2009, 131 (36), 13180 10.1021/ja906709t.

[ref27] Stoll S.; Schweiger A. EasySpin, a Comprehensive Software Package for Spectral Simulation and Analysis in EPR. J. Magn. Reson. 2006, 178 (1), 42–55. 10.1016/j.jmr.2005.08.013. 16188474

[ref28] Šakić D.; Bešić E.; Zubčić G.; Chechik V. VisualEPR. https://github.com/DSakicLab/visualEPR (accessed 2023-12-01).

[ref29] Pracht P.; Bohle F.; Grimme S. Automated Exploration of the Low-Energy Chemical Space with Fast Quantum Chemical Methods. Phys. Chem. Chem. Phys. 2020, 22 (14), 7169–7192. 10.1039/C9CP06869D. 32073075

[ref30] Bannwarth C.; Caldeweyher E.; Ehlert S.; Hansen A.; Pracht P.; Seibert J.; Spicher S.; Grimme S. Extended tight-binding Quantum Chemistry Methods. Wiley Interdiscip. Rev. Comput. Mol. Sci. 2021, 11 (2), e1493 10.1002/wcms.1493.

[ref31] Bannwarth C.; Ehlert S.; Grimme S. GFN2-XTB—An Accurate and Broadly Parametrized Self-Consistent Tight-Binding Quantum Chemical Method with Multipole Electrostatics and Density-Dependent Dispersion Contributions. J. Chem. Theory Comput. 2019, 15 (3), 1652–1671. 10.1021/acs.jctc.8b01176. 30741547

[ref32] Becke A. D. Density-Functional Thermochemistry. III. The Role of Exact Exchange. J. Chem. Phys. 1993, 98 (7), 5648–5652. 10.1063/1.464913.

[ref33] Stephens P. J.; Devlin F. J.; Chabalowski C. F.; Frisch M. J. Ab Initio Calculation of Vibrational Absorption and Circular Dichroism Spectra Using Density Functional Force Fields. J. Phys. Chem. 1994, 98 (45), 11623–11627. 10.1021/j100096a001.

[ref34] Ditchfield R.; Hehre W. J.; Pople J. A. Self-Consistent Molecular-Orbital Methods. IX. An Extended Gaussian-Type Basis for Molecular-Orbital Studies of Organic Molecules. J. Chem. Phys. 1971, 54 (2), 724–728. 10.1063/1.1674902.

[ref35] Grimme S. Semiempirical Hybrid Density Functional with Perturbative Second-Order Correlation. J. Chem. Phys. 2006, 124 (3), 034108 10.1063/1.2148954. 16438568

[ref36] Neese F.; Schwabe T.; Grimme S. Analytic Derivatives for Perturbatively Corrected “Double Hybrid” Density Functionals: Theory, Implementation, and Applications. J. Chem. Phys. 2007, 126 (12), 124115 10.1063/1.2712433. 17411116

[ref37] Curtiss L. A.; Redfern P. C.; Raghavachari K.; Rassolov V.; Pople J. A. Gaussian-3 Theory Using Reduced Mo/Ller-Plesset Order. J. Chem. Phys. 1999, 110 (10), 4703–4709. 10.1063/1.478385.

[ref38] Grimme S.; Antony J.; Ehrlich S.; Krieg H. A Consistent and Accurate *Ab Initio* Parametrization of Density Functional Dispersion Correction (DFT-D) for the 94 Elements H-Pu. J. Chem. Phys. 2010, 132 (15), 154104 10.1063/1.3382344. 20423165

[ref39] Vrček I. V.; Šakić D.; Vrček V.; Zipse H.; Biruš M. Computational Study of Radicals Derived from Hydroxyurea and Its Methylated Analogues. Org. Biomol. Chem. 2012, 10 (6), 1196–1206. 10.1039/C1OB06594G. 22179435

[ref40] Hermosilla L.; Calle P.; García de la Vega J. M.; Sieiro C. Density Functional Theory Study of ^14^N Isotropic Hyperfine Coupling Constants of Organic Radicals. J. Phys. Chem. A 2006, 110 (50), 13600–13608. 10.1021/jp064900z. 17165888

[ref41] Frisch M. J.; Trucks G. W.; Schlegel H. B.; Scuseria G. E.; Robb M. A.; Cheeseman J. R.; Scalmani G.; Barone V.; Petersson G. A.; Nakatsuji H.; ; Gaussian 16, Revision C. 01. Gaussian, Inc.: Wallingford CT, 2016.

[ref42] HR-ZOO, Cluster Supek; University of Zagreb University Computing Centre—SRCE. KK.01.1.1.08.0001, EU funded within OPCC for Republic of Croatia: Zagreb, 2023.

[ref43] PharmInova Project, Cluster Sw.Pharma.Hr; University of Zagreb Faculty of Pharmacy and Biochemistry. KK.01.1.1.02.0021, EU funded by the European Regional Development Fund: Zagreb, 2023.

[ref44] Šakić D.; Zipse H. Radical Stability as a Guideline in C-H Amination Reactions. Adv. Synth. Catal. 2016, 358 (24), 3983–3991. 10.1002/adsc.201600629.

[ref45] Hioe J.; Šakić D.; Vrček V.; Zipse H. The Stability of Nitrogen-Centered Radicals. Org. Biomol. Chem. 2015, 13 (1), 157–169. 10.1039/C4OB01656D. 25351112

[ref46] Hioe J.; Zipse H. Radical Stability—Thermochemical Aspects. In Encyclopedia of Radicals in Chemistry, Biology and Materials; Chatgilialoglu C., Studer A., Eds.; Wiley: UK, 2012, pp 449–476. 10.1002/9781119953678.RAD012.

[ref47] Shkunnikova S.; Zipse H.; Šakić D. Role of Substituents in the Hofmann-Löffler-Freytag Reaction. A Quantum-Chemical Case Study on Nicotine Synthesis. Org. Biomol. Chem. 2021, 19 (4), 854–865. 10.1039/D0OB02187C. 33406192

[ref48] Corey E. J.; Hertler W. R. A Study of the Formation of Haloamines and Cyclic Amines by the Free Radical Chain Decomposition of N-Haloammonium Ions (Hofmann-Löffler Reaction). J. Am. Chem. Soc. 1960, 82 (7), 1657–1668. 10.1021/ja01492a035.

[ref49] Carloni P.; Eberson L.; Greci L.; Sgarabotto P.; Stipa P. New Insights on N-Tert-Butyl-α-Phenylnitrone (PBN) as a Spin Trap. Part 1. Reaction between PBN and N-Chlorobenzotriazole. J. Chem. Soc. Perkin Trans. 2 1996, 7 (7), 1297–1305. 10.1039/P29960001297.

[ref50] Constantinou C. T.; Gkizis P. L.; Lagopanagiotopoulou O. T. G.; Skolia E.; Nikitas N. F.; Triandafillidi I.; Kokotos C. G. Photochemical Aminochlorination of Alkenes without the Use of an External Catalyst. Chem.—Eur. J. 2023, 29 (45), e202301268 10.1002/CHEM.202301268. 37254681

[ref51] Korotenko V.; Zipse H. The Stability of Oxygen-Centered Radicals and Its Response to Hydrogen Bonding Interactions. J. Comput. Chem. 2024, 45, 101–114. 10.1002/jcc.27221. 37747356

[ref52] Zhu Y.; Wang J. J.; Wu D.; Yu W. Visible-Light-Driven Remote C-H Chlorination of Aliphatic Sulfonamides with Sodium Hypochlorite. Asian J. Org. Chem. 2020, 9 (10), 1650–1654. 10.1002/ajoc.202000354.

[ref53] Nikolaienko P.; Jentsch M.; Kale A. P.; Cai Y.; Rueping M. Electrochemical and Scalable Dehydrogenative C(Sp3)-H Amination via Remote Hydrogen Atom Transfer in Batch and Continuous Flow. Chem.—Eur. J. 2019, 25 (29), 7177–7184. 10.1002/CHEM.201806092. 30861204

[ref54] Herron A. N.; Hsu C. P.; Yu J. Q. δ-C-H Halogenation Reactions Enabled by a Nitrogen-Centered Radical Precursor. Org. Lett. 2022, 24 (20), 3652–3656. 10.1021/acs.orglett.2c01261. 35549294 PMC10292861

[ref55] Leijondahl K.; Borén L.; Braun R.; Bäckvall J. E. Enantiopure 1,5-Diols from Dynamic Kinetic Asymmetric Transformation. Useful Synthetic Intermediates for the Preparation of Chiral Heterocycles. Org. Lett. 2008, 10 (10), 2027–2030. 10.1021/ol800468h. 18402460

[ref56] Marichev K. O.; Takacs J. M. Ruthenium-Catalyzed Amination of Secondary Alcohols Using Borrowing Hydrogen Methodology. ACS Catal. 2016, 6 (4), 2205–2210. 10.1021/acscatal.6b00175. 28936364 PMC5604847

