# Supplementary material for: Regioselective Rearrangement of Nitrogen- and Carbon-Centered Radical Intermediates in the Hofmann–Löffler–Freytag Reaction
Source: J Phys Chem A. 2024 Mar 22;128(13):2574–83. doi: 10.1021/acs.jpca.3c07892 (PMC11000220; doi:10.1021/acs.jpca.3c07892)
Supplement: Supplementary file 1 — jp3c07892_si_001.pdf [file jp3c07892_si_001.pdf]

SUPPORTING INFORMATION

Regioselective Rearrangement of Nitrogen- and Carbon-Centered Radical Intermediates in the Hofmann–Löffler–Freytag Reaction

Gabrijel Zubčić,[1] Jiangyang You,[2] Fabian L. Zott,[3] Salavat S. Ashirbaev,[3] Maria Kolympadi Marković,[4] Erim Bešić,[1] Valerije Vrček,[1] Hendrik Zipse,[3] and Davor Šakić[1]*

AUTHOR ADDRESS

[1] Faculty of Pharmacy and Biochemistry, University of Zagreb, Ante Kovačića 1, 10000 Zagreb, Croatia
[2] Division of Physical Chemistry, Ruđer Bošković Institute, Bijenička cesta 54, 10000 Zagreb
[3] Department of Chemistry, Ludwig-Maximilians-Universität München, Butenandtstrasse 5-13, D-81377 München, Germany
[4] Faculty of Physics, and Centre for micro- and nanosciences and technologies, University of Rijeka, Radmile Matejčić 2, 51000 Rijeka, Croatia

*Davor Šakić –
Faculty of Pharmacy and Biochemistry, University of Zagreb, Ante Kovačića 1, 10000 Zagreb, Croatia; orcid.org/0000-0002-8871-6622; Email: davor.sakic@pharma.unizg.hr

TABLE OF CONTENTS

SUMMARY OF EXPERIMENTAL METHODS S3
GENERAL SYNTHESIS PROCEDURE S6
GENERAL PROCEDURE A: PREPARATION OF *N*-CHLOROSULFONAMIDES WITH TRICHLOROISOCYANURIC ACID. S14
GENERAL PROCEDURE B: PREPARATION OF N-BROMOSULFONAMIDES WITH N-BROMO SUCCINIMIDE. S17
REACTION 7-H WITH PIDA/I_2 IN THE DARK S18
REACTION 7-H WITH PIDA/I_2 UNDER IRRADIATION S19
DEGASSING PROCEDURE AND EPR MEASUREMENTS S23
EPR MEASUREMENTS 7-I S24
EPR MEASUREMENTS 7-BR S25
EPR MEASUREMENTS 7-CL S30
EPR MEASUREMENTS 11-CL S37
EPR MEASUREMENTS 11-BR S38
NMR SPECTRA OF 7-I REACTION MIXTURE UNDER IRRADIATION S39
NMR SPECTRA OF 7-CL REACTION MIXTURE UNDER IRRADIATION S40
NMR SPECTRA OF 11-CL REACTION MIXTURE UNDER IRRADIATION S41
NMR SPECTRA OF 11-BR REACTION MIXTURE UNDER IRRADIATION S43
DFT RESULTS AND OPTIMIZED GEOMETRIES S44
EPR CALCULATIONS OF PBN ADDUCTS S174
REFERENCES S222

SUMMARY OF EXPERIMENTAL METHODS

Purchased compounds were sourced from Kefo (sulfuric acid (98 %), methanol, petroleum ether, p-toulensulfonyl chloride, silica gel, pyridine, silver acetate, ethyl acetate, cyclohexane, triflouroacetic anhydride, toluene, trichloroisocyanuric acid), Ru-Ve (hydrochloric acid (37 %), acetone, silicon oil, petroleum ether, cyclohexane), and Biovit (toulene (anhydrous), acetonitrile (anhydrous), 1,4-dioxane (anhydrous), tetrahydrofuran (anhydrous), N,N-dimethylformamide (anhydrous), N,N-dimethylacetamide, 1,2-dichloroethane (anhydrous), dichloromethane (anhydrous)). All reagents and chemicals were obtained commercially and used without further purification unless otherwise noted.

Moisture-sensitive reactions were performed using flame-dried glassware under an atmosphere of nitrogen (N_2). Air- and moisture-sensitive liquids and solutions were transferred via plastic or glass syringe. Chromatographic purification of the products was carried out using column chromatography filled with silica gel (Macherey-Nagel) 0.063-0.2 mm, and appropriate solvent mixtures were used as eluents: petroleum ether/ethyl acetate. Thin-layer chromatography (TLC) was performed on pre-coated TLC plates ALUGRAM SIL G/UV254, 0.20 mm silica gel 60 with fluorescent indicator UV254 (Macherey-Nagel) in the appropriate solvent system. TLC spots were observed after illumination with UV light at a wavelength of 254 nm, and after immersion in an aqueous solution of $KMnO_4$ (3 g $KMnO_4$, 20 g K_2CO_3, 5 mL aq. NaOH 5%, 300 mL water) followed by heating. If TLC spots were not visible after illumination with UV-light, they were detected utilizing iodine chamber.

Synthetic photocatalyzed reactions were performed in a custom build photoreactor with build in temperature control, as well as standardized luminous intensity for a certain set of high-power LED light sources. The InGaN based, high power single chip LEDs used in this experimental setup were purchased from Roithner Lasertechnik GmbH and mounted on a standard hexagonal aluminum package. As the H2A1-420 LED was used during this project, the technical details are described in detail. The operation temperature of the 420 nm diode ranges between -30 and +70°C, for which reason it always has to be attached to a heat sink. It is important to state, that these LEDs wavelength numbers only describe the peak value, while they cover a broader spectrum around the peak wavelength. The full width at half maximum (FWHM) value $\Delta\lambda$ gives the width of a spectrum curve measured at 50% output and provides a good estimation of the narrowness of the emission peak. In case of the H2A1-420 LED $\Delta\lambda$ at 350mA is 20nm.

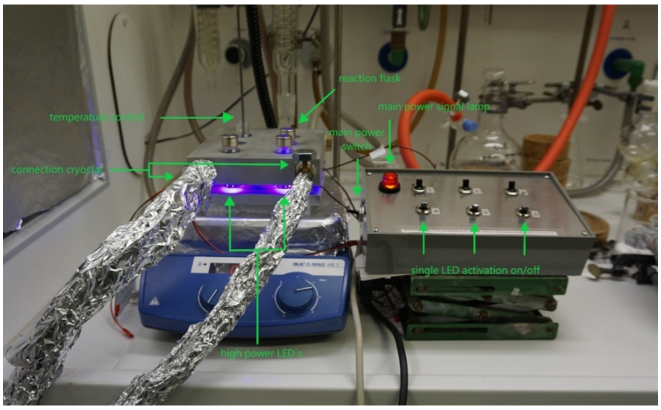

Picture S1. Photochemical synthetic setup

As seen in Picture S1, reaction setup for photoirradiation consists out of an aluminum plate, onto which six high power LED are mounted with heat-conducting tape. The cooling of the LEDs is ensured by the high thermal conductivity of the bottom aluminum plate. On top of this

light source a second aluminum panel with cryostat connectors is mounted by four spacers. The top panel has six voids for 10ml GC vials and is open towards the LED light source on the bottom. This setup always ensures the same irradiation intensity of the reaction mixture for the same LED models in the GC-vials with a distance of 0.6 cm between light source and reaction vessel. The magnetic stir plate provides the steady circulation within the reaction flasks and also connects the control thermometer, that is attached in one of the six vials. During a photo reaction the open spaces are covered with thin foil for eye protection.
The custom-made power-supply unit follows the special requirements of LED lights. All LEDs are connected in series, with the ability to individually trigger each LED, always maintaining the same current (350 mA). This results in a highly flexible setup with the ability to run multiple reactions with different times and light sources.
Irradiation was performed *in situ* (EPR) and *off-site* (NMR) with Kessil PR-160L 370 ± 10 nm gen-2 LED UV, with average intensity 137 mW/cm^2 when sample is 6 cm from the lamp, according to manufacturer.[1]

a) 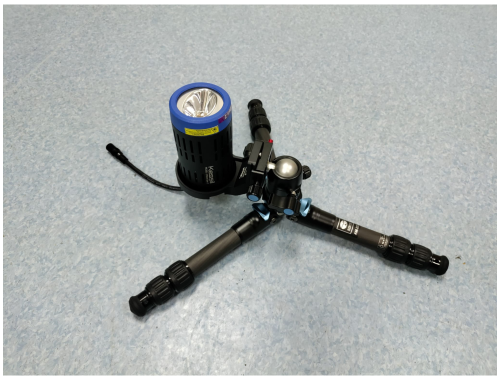 b) 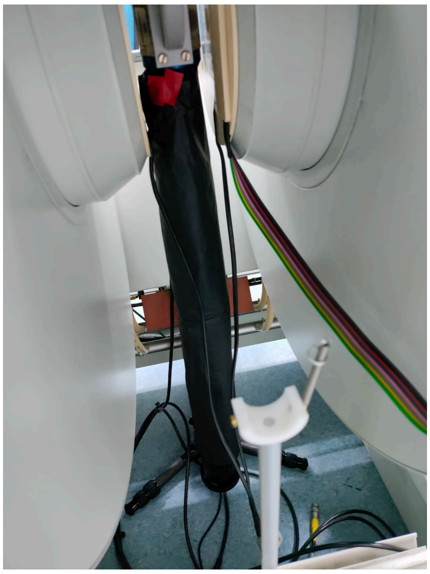

Picture S2. a) Kessil PR-160L on tripod, b) setup for EPR illumination inside cavity

Reactant and products were identified using ^{1}H-NMR spectra, which were recorded on Mercury 200, Varian INOVA 400 and 600 machines in $CDCl_3$ at 400 MHz or 600 MHz at room temperature. All ^{13}C-NMR spectra were recorded respectively at 101 MHz and 151 MHz. The chemical shifts are reported in ppm (δ), relative to the resonance of $CDCl_3$ at δ = 7.27 ppm ^{1}H and for ^{13}C relative to the resonance of $CDCl_3$ δ = 77.16 ppm. Spectra were imported and processed in the MestreNova 11.0.4 program. NMR spectra of reaction mixture were obtained on Varian Inova 400 NMR spectrometer operating at 399.90 MHz for ^{1}H NMR, 100.6 MHz for ^{13}C NMR, and are reported as chemical shifts (δ) in parts per million (ppm). Spectra were referenced internally according to residual solvent signals (^{1}H: $CDCl_3$, 7.26 ppm; ^{13}C: $CDCl_3$, 77.0 ppm;). Spectra were imported and processed in the MestreNova 14.2.0 program. Data for NMR spectra use the following abbreviations to describe multiplicity: s, singlet; br s, broad singlet; d, doublet; t, triplet; q, quartet; dd, doublet of doublets; td, triplet of doublets; ddd, doublet of doublet of doublets; ddt, doublet of doublet of triplets; app dd, apparent doublet of doublets; m, multiplet. Coupling constant (J) are reported in units of Hertz (Hz).
GC measurements were obtained at a Shimadzu GC-2010 Plus Gas Chromatograph with AOC-20i autosampler (with temperature-controlled sample holder) and an Optima 1701-0.25 µm (25 m * 0.25 mm) column.

HR-MS spectra were obtained by using a Thermo Finnigan LTQ FT machine of the MAT 95 type with a direct exposure probe (DEP) and electron impact ionization (EI, 70 eV).
EPR spectroscopy was done using Bruker E500 Elexsys EPR spectrometer with ER4122SHQE cavity resonator. As this cavity resonator doesn't have an optical window for illumination, light source was mounted underneath the cavity, with light coming through the bottom of the EPR 4 mm inner diameter tube.
EPR deconvolution and simulation was done using EasySpin module with MATLAB program package.[2]
EPR visualization and spectra were done using VisualEPR web-page.[3]
Conformational space was sampled and investigated using *Conformer–Rotamer Ensemble Sampling Tool* – CREST[3] coupled with xtb-GFN2 program package and MD simulation using xtb-GFN1[4-5] and xtb-GFN2.[6]
Obtained structures were re-optimized using B3LYP/6-31G(d) level of theory.[7-9] For each structure with stable wavefunction, frequency calculation was performed to identify minima and transition state structures. From transition state structure intrinsic reaction coordinate search (IRC) was performed to characterize corresponding reaction and product complexes/reactive conformers. Improved thermodynamics were obtained using RO-B2PLYP[10-11] with G3MP2-large basis set[12] on geometries obtained at B3LYP/6-31G(d) level of theory, with additional D3 dispersion correction.[13] Additionally, for smaller and model systems G3B3 composite method was used, with results matching the RO-B2PLYP-D3/G3MP2-large results.
Calculations of EPR parameters were done using B3LYP functional and mixed basis set: EPR-III for C, H, O atoms, def2-QZVP for S-atom, and 6-31G(d) for N-atom. Small basis set on N-atom is necessary for the correct calculations of g-factor and hyperfine coupling constants (*hfc*).[14-16] When using larger basis set for N-atom, e.g. EPR-III or def2-QZVP, obtained results systematically underestimate *hfc*. Calculations were performed on Gaussian version 16.C01[17] using the Advanced computing service (clusters Isabella and Supek) provided by University of Zagreb University Computing centre – SRCE.[18] and computational resources of PharmInova project (sw.pharma.hr) at University of Zagreb Faculty of Pharmacy and Biochemistry.[19]

General synthesis procedure

*Synthesis of N-hexyl-4-methylbenzenesulfonamide **7-H***

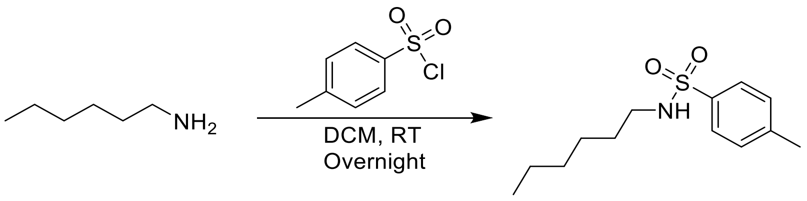

3.1 ml of n-hexylamine (2.4 g, 24.6 mmol, 2.1 eq) was added dropwise to a solution of 2.0 g TsCl (11.7 mmol, 1 eq) in 40 mL of dry DCM at 0°C and stirred for overnight at room temperature. The cooling bath was removed, and the reaction mixture was allowed to stir overnight. The reaction was quenched with 100 ml of water, extracted with DCM (3×50 mL). Organic phases were combined, washed with brine (50 mL), dried over $MgSO_4$ and evaporated. Crude material was purified by recrystallization from *i*-hexane and obtained as colorless solid (69%, 2.1 g) Spectral data are in agreement with literature values.[20]

^{1}H NMR (400 MHz, $CDCl_3$) δ 7.75 (d, J = 8.2 Hz, 2H), 7.31 (d, J = 8.2 Hz, 2H), 4.36 (t, J = 6.2 Hz, 1H), 3.09 – 2.77 (m, 1H), 2.43 (s, 3H), 1.47 – 1.40 (m, 2H), 1.31 – 1.12 (m, 6H), 0.84 (t, J = 7.1 Hz, 3H).
^{13}C NMR (101 MHz, $CDCl_3$) δ 143.5, 137.1, 129.8, 127.3, 43.4, 31.4, 29.7, 26.3, 22.6, 21.7, 14.1.
Elem. Calcd for $C_{13}H_{21}NO_2S$: C 61.15; H 8.29; N 5.48; S 12.55 Found: C 61.41; H 8.67; N 5.36; S 12.78

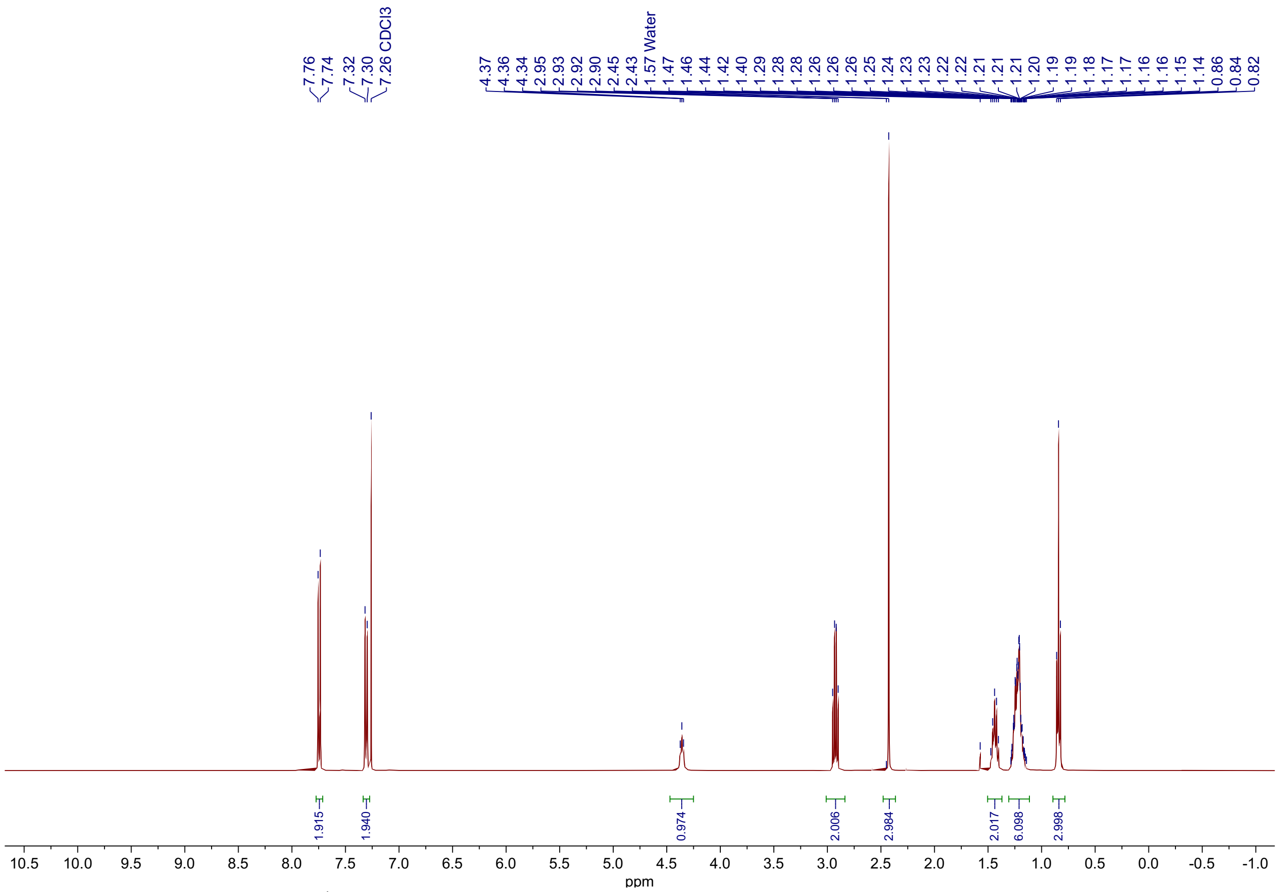

Figure S1. ^{1}H NMR spectra of sulfonamide **7-H**, $CDCl_3$, 400 MHz

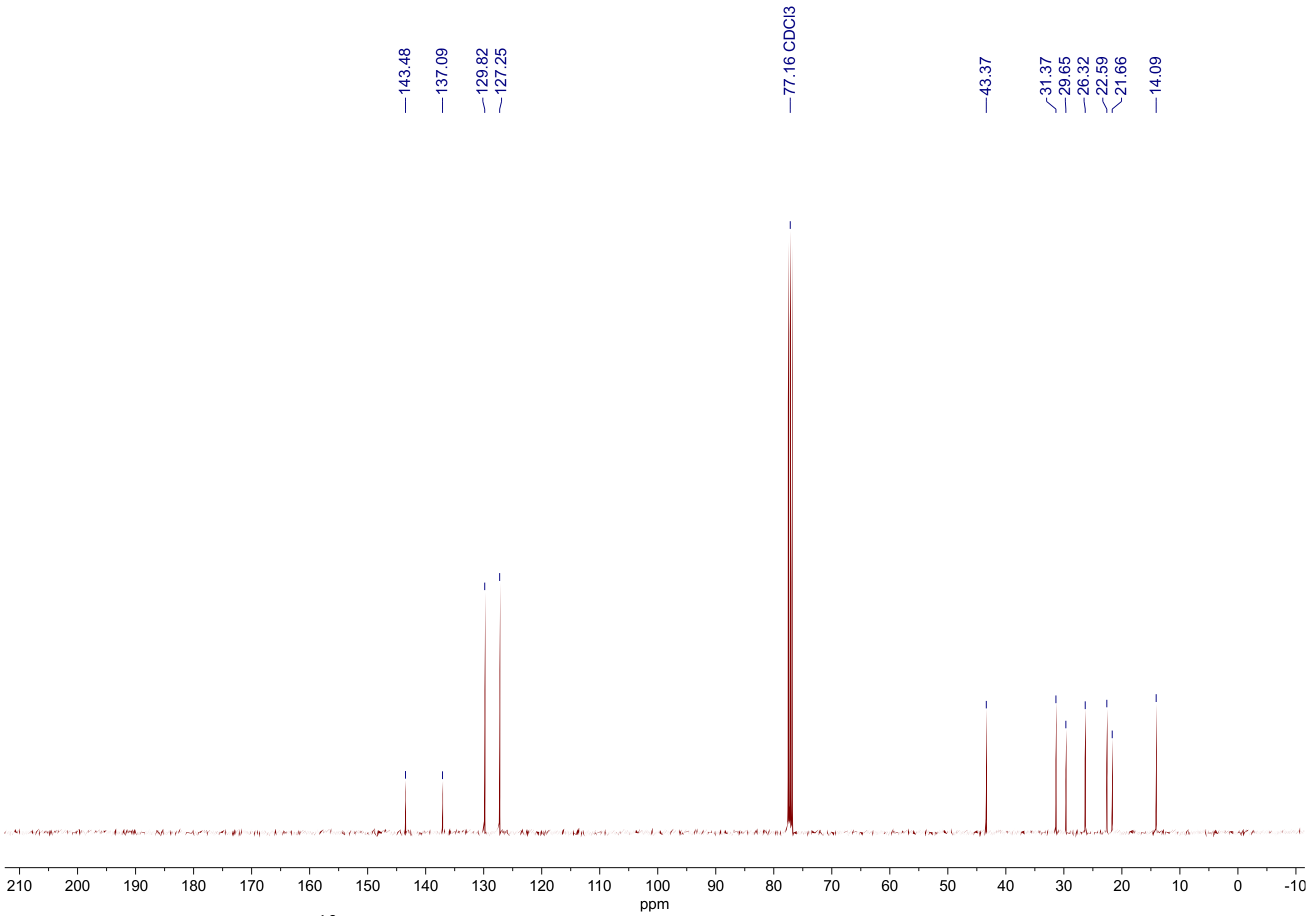

Figure S2. ^{13}C NMR spectra of sulfonamide **7-H**, $CDCl_3$, 101 MHz

Synthesis of 2-Ethylpyrrolidine ***8-H***

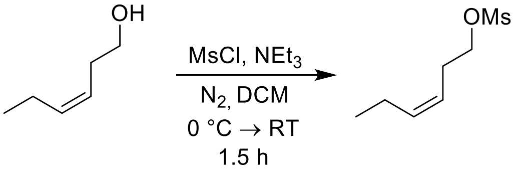

Following the modified literature procedure.[21]

3.5 ml of triethylamine (2.5 g, 25 mmol, 5 eq) was added to the solution of 0.5 g cis-3-Hexenol (5 mmol, 1 eq) in 25 ml of dry DCM. The solution was cooled to 0 °C and 0.46 ml of MsCl (0.69 g, 6 mmol, 1.2 eq) was added dropwise. The solution was stirred for 1.5 hours at room temperature, quenched with 50 ml of saturated $NaHCO_3$ solution, extracted with DCM (3×15 ml). The combined organic layers were dried over $MgSO_4$. Solvents were evaporated under reduced pressure. The crude material was passed through a short silica plug (DCM) to afford a pale-yellow oil of (3Z)-hex-3-en-1-yl methanesulfonate (0.79 g, 88%). Spectral data are in agreement with literature values.[22]

^{1}H NMR (400 MHz, $CDCl_3$) δ 5.61 – 5.53 (m, 1H), 5.37 – 5.25 (m, 1H), 4.20 (t, J = 6.9 Hz, 2H), 3.00 (s, 3H), 2.55 – 2.45 (m, 2H), 2.11 – 2.02 (m, 2H), 0.98 (t, J = 7.5 Hz, 3H).

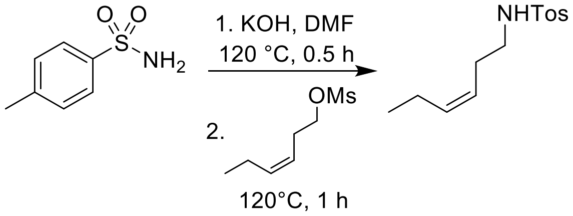

Following the modified literature procedure.[21]

0.5 g of 85% KOH (7.5 mmol, 1.5 eq) was dissolved in 5 ml of dry DMF at 120 °C, followed by addition of 1.28 g of tosylamide (7.5 mmol, 1.5 eq). The resulting solution was stirred for 30 min at 120 °C. After, a solution of the mesylate, obtained above in 5 ml of dry DMF was added in one portion. After 1 h of stirring at 120 °C, the reaction was cooled to room temperature, quenched with water and extracted with DCM (3×10 mL). The combined organic layers were washed with 50 ml of water, dried over $MgSO_4$ and evaporated. The crude material was passed through a short silica plug (iHex/EtOAc = 10/1) to afford N-((3Z)-hex-3-en-1-yl)tosylamine as colourless oil (45%, 0.51 g). Spectral data are in agreement with literature values.[23]

^{1}H NMR (400 MHz, $CDCl_3$) δ 7.78 – 7.70 (m, 2H), 7.35 – 7.27 (m, 2H), 5.56 – 5.44 (m, 1H), 5.19 – 5.08 (m, 1H), 4.32 (t, J = 6.2 Hz, 1H), 2.97 (q, J = 6.6 Hz, 2H), 2.43 (s, 3H), 2.25 – 2.15 (m, 2H), 2.02 – 1.94 (m, 2H), 0.94 (t, J = 7.5 Hz, 3H).

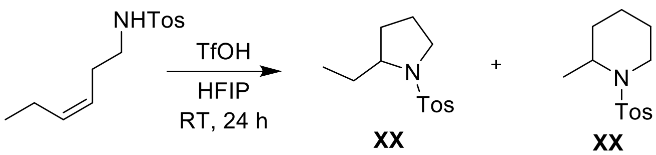

XX **XX**

96 4

Following the modified literature procedure.[24]

0.51 g of the obtained N-((3Z)-hex-3-en-1-yl)tosylamine (2 mmol, 1 eq) was dissolved in 5 ml of HFIP. 0.35 ml of TfOH (0.6 g, 4 mmol, 2 eq) was added dropwise. The resulting solution was stirred for 24 hours at room temperature. Reaction mixture was quenched with saturated aqueous solution of $NaHCO_3$ and extracted with DCM (3×10 mL). The combined organic layers were dried over $MgSO_4$. Solvents were evaporated under reduced pressure. Crude

material was purified by flash chromatography (iHex/EtOAc= 10/1, Rf 0.25) to give XX (508 mg, 99%) as a white solid. Spectral data are in agreement with literature values.[24]

Further ^{1}H NMR and GC-FID analysis showed that the obtained product consists of 5- and 6-membered adduct XX and XX with ratio 96:4, correspondingly.

^{1}H NMR (400 MHz, $CDCl_3$) δ 7.76 – 7.70 (m, 1H), 7.32 – 7.29 (m, 2H), 3.54 (ddd, J = 10.3, 7.9, 5.1 Hz, 1H), 3.38 (ddd, J = 10.4, 7.0, 5.1 Hz, 1H), 3.19 (dt, J = 10.3, 7.2 Hz, 1H), 2.43 (s, 3H), 2.00 – 1.66 (m, 2H), 1.65 – 1.37 (m, 4H), 0.91 (t, J = 7.4 Hz, 1H).

^{13}C NMR (101 MHz, CDCl3) δ 143.3, 135.1, 129.7, 127.6, 61.9, 49.1, 30.2, 29.3, 24.2, 21.6,

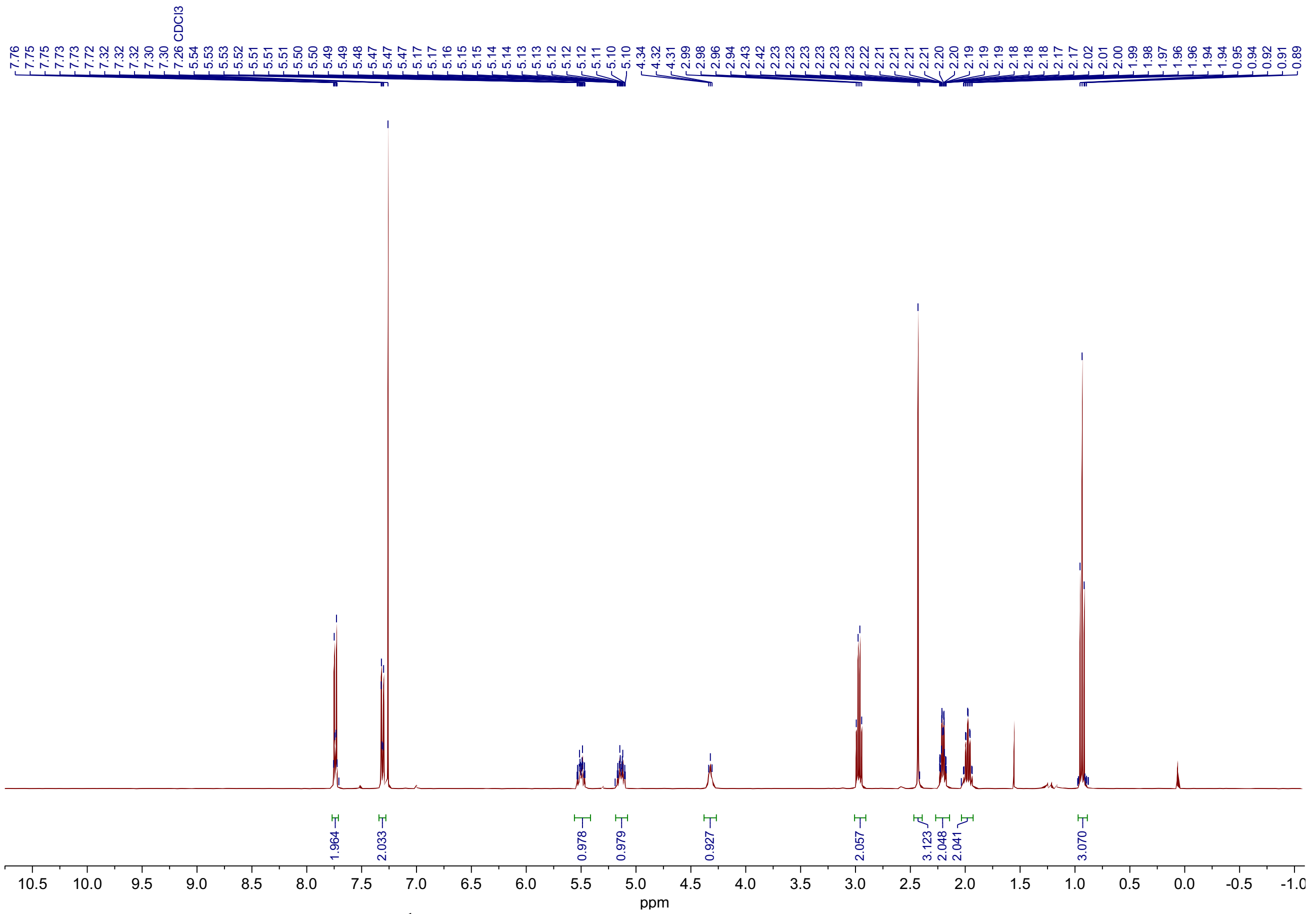

Figure S3. ^{1}H NMR spectra of **8-H**, $CDCl_3$, 400 MHz

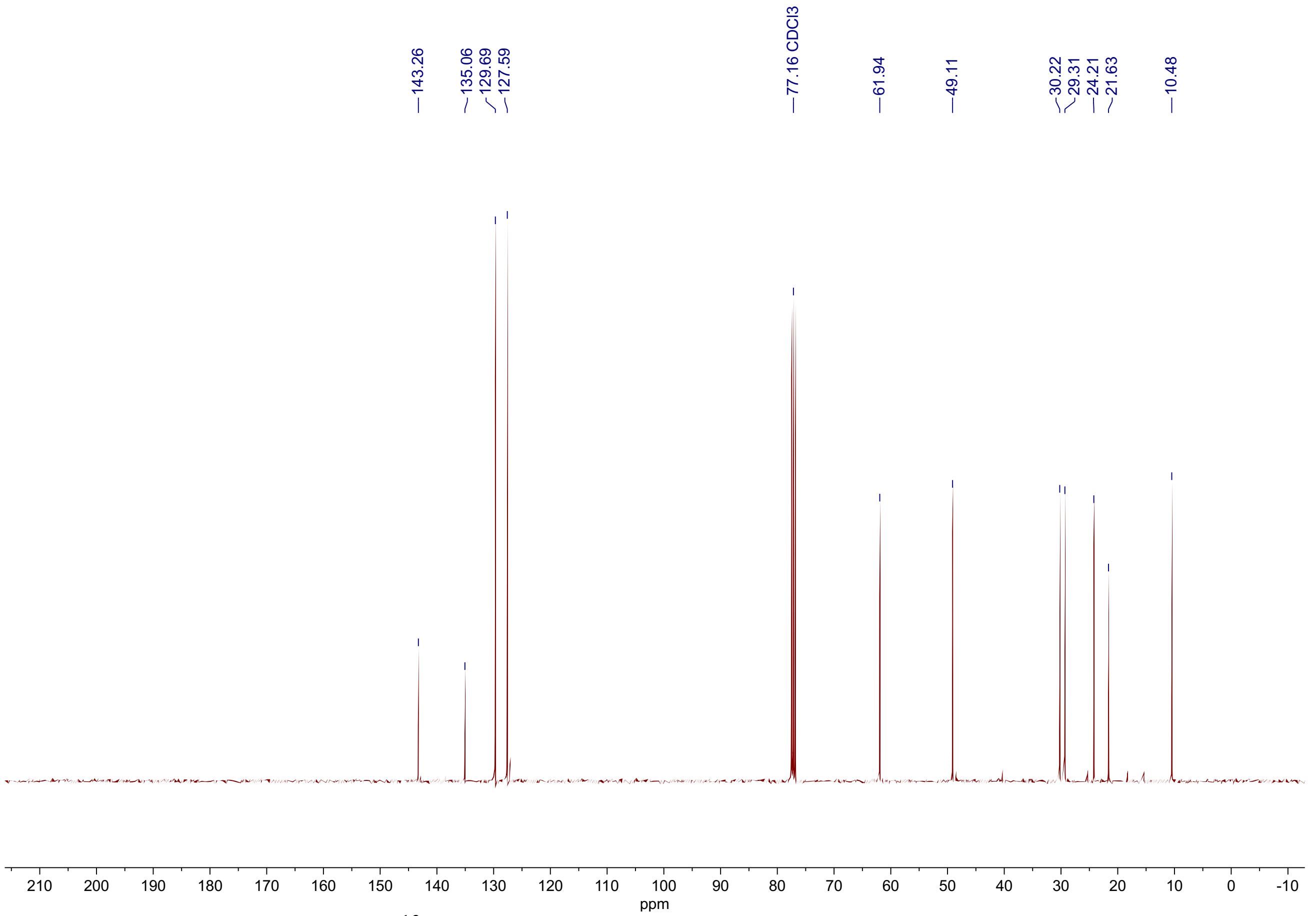

Figure S4. ^{13}C NMR spectra of **8-H**, $CDCl_3$, 101 MHz

*Synthesis of N-tosyl-2-Methylpiperidine **9-H***

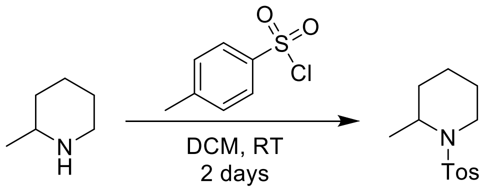

XX

1.4 ml of 2-methylpiperidine (1.15 g, 11.7 mmol, 2.1 eq) was added dropwise to a solution of 1.0 g TsCl (5.8 mmol, 1 eq) in 20 mL of dry DCM at 0°C and stirred for overnight at room temperature. The cooling bath was removed and the reaction mixture was allowed to stir for 2 days. The reaction was quenched with 50 ml of water, extracted with DCM (3×25 mL). Organic phases were combined, washed with brine (50 mL), dried over $MgSO_4$ and evaporated. Crude material was purified by recrystallization from *i*-hexane and obtained as colorless solid (65%, 0.96 g). Spectral data are in agreement with literature values

^{1}H NMR (400 MHz, $CDCl_3$) δ 7.78 – 7.56 (m, 2H), 7.40 – 7.15 (m, 2H), 4.52 – 4.09 (m, 1H), 3.88 – 3.53 (m, 1H), 2.96 (td, J = 13.0, 2.6 Hz, 1H), 2.41 (s, 3H), 1.66 – 1.46 (m, 4H), 1.46 – 1.30 (m, 2H), 1.05 (d, J = 6.9 Hz, 3H).

^{13}C NMR (101 MHz, $CDCl_3$) δ 142.9, 138.5, 129.7, 127.1, 48.6, 40.4, 30.5, 25.3, 21.6, 18.3, 15.4.

Elem. Calcd for $C_{13}H_{19}NO_2S$: C 61.63; H 7.56; N 5.53; S 12.65 Found: C 61.92; H 7.86; N 5.50; S 12.95

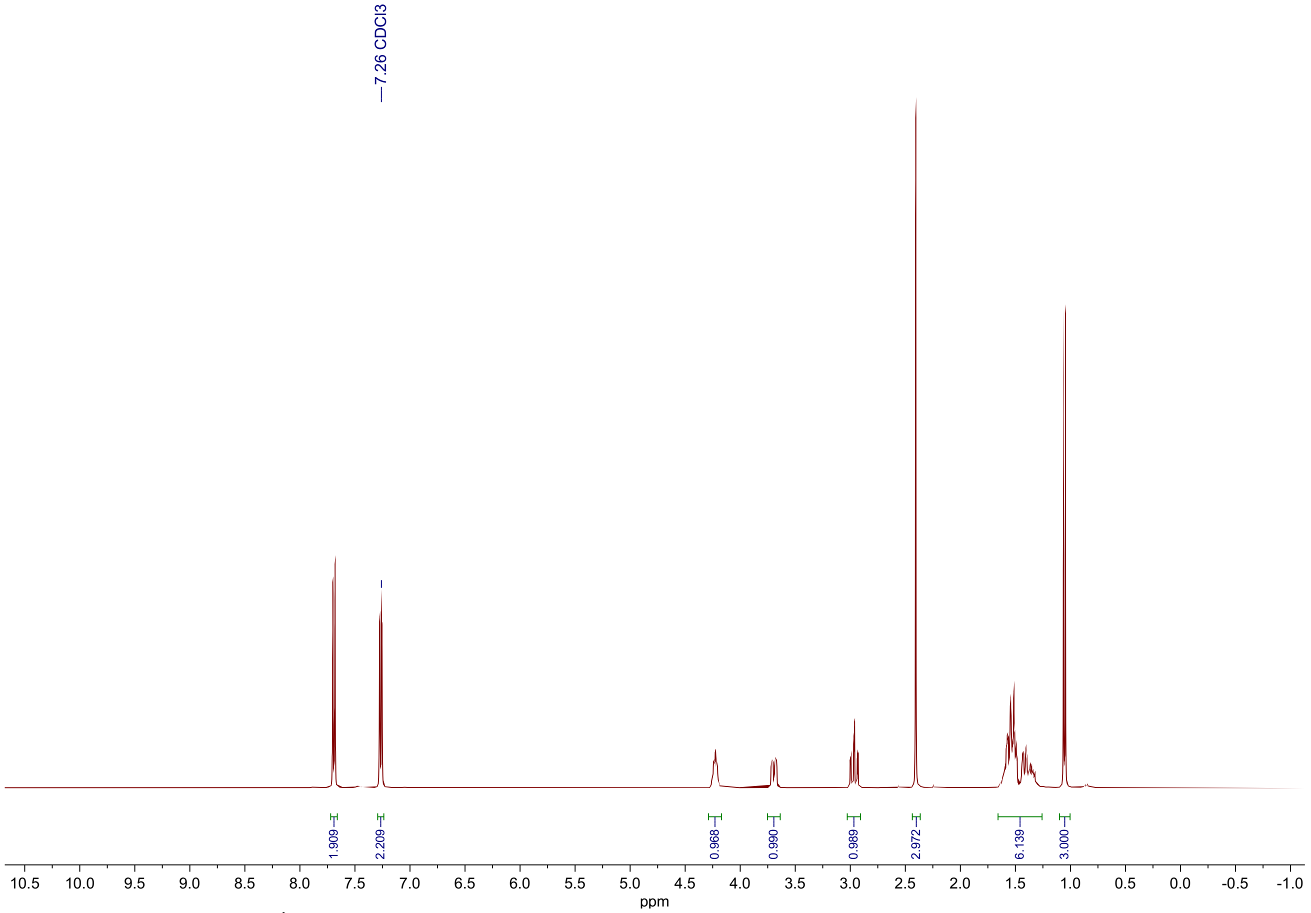

Figure S5. ^{1}H NMR spectra of 2-methylpiperidine **9-H**, $CDCl_3$, 400 MHz

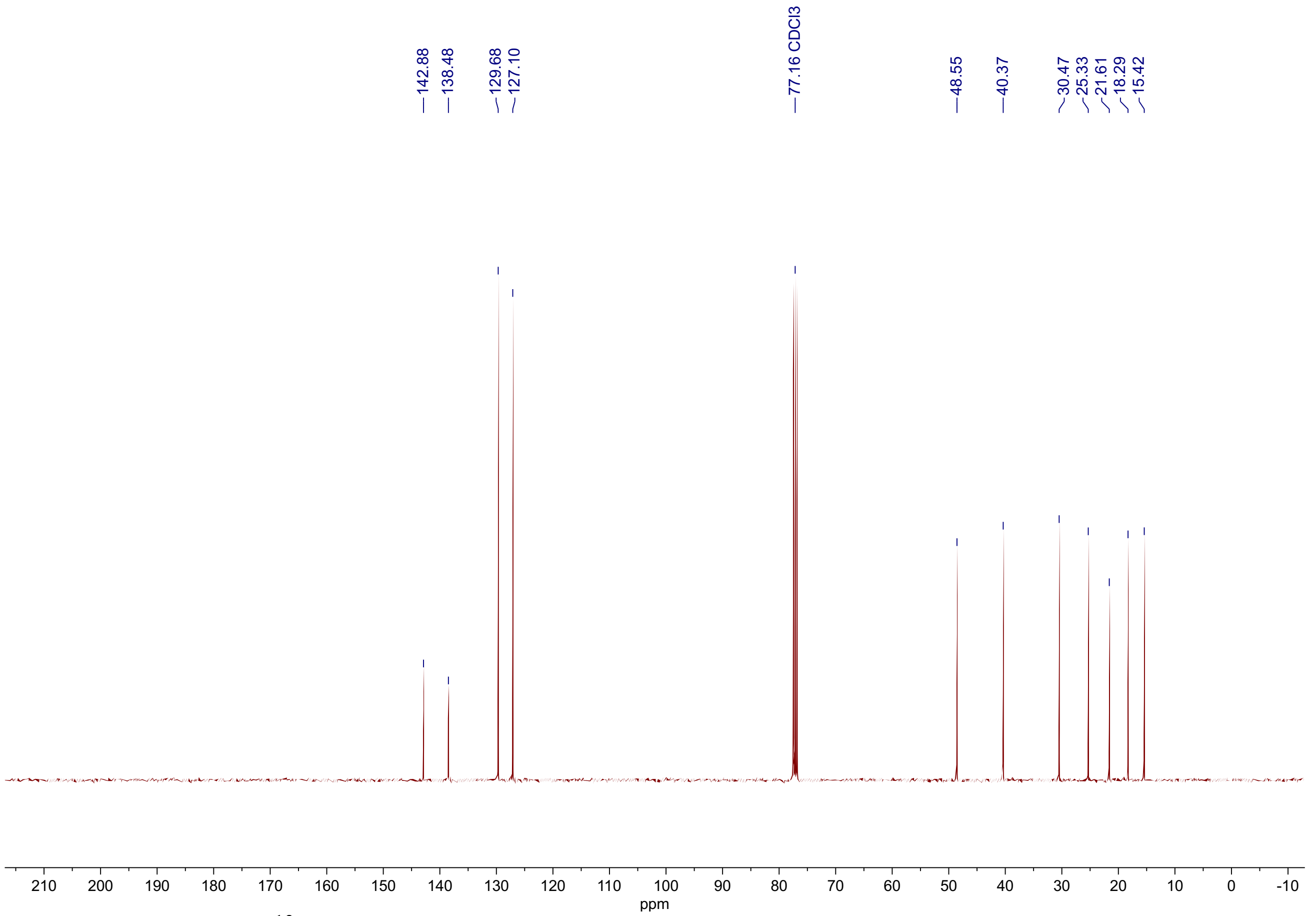

Figure S6. ^{13}C NMR spectra of 2-methylpiperidine **9-H**, $CDCl_3$, 101 MHz

*Synthesis of N-(heptan-2-yl)-4-methylbenzenesulfonamide **11-H***

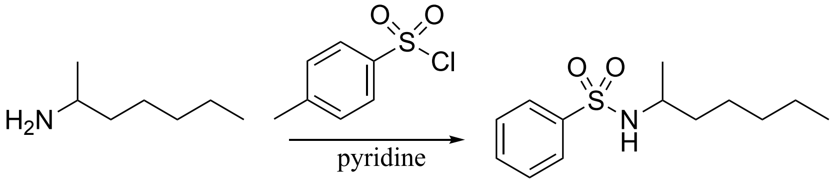

1.31 ml of heptane-2-amine (1.0 g, 8.67 mmol, 2.1 eq) was added dropwise to a solution of 2.48 g TsCl (13 mmol, 1.5 eq) in 50 mL of pyrdine at 0°C. The cooling bath was removed after 30 minutes, and the reaction mixture was allowed to stir overnight. The reaction was quenched with 50 mL of DCM, and extracted with 10% HCl solution until all the pyridine was removed. The combined organic phases were dried with Na_2SO4, filtered, and concentrated under reduced pressure. The crude material was purified by silica gel column chromatography eluting with a petroleum ether:EtOAc (85:15). Pure product was obtained as colorless oil with a yellowish tone to it (85%, 1.98 g)

^1H NMR (400 MHz, $CDCl_3$) δ 7.77 (d, J = 8.2 Hz, 2H), 7.30 (d, J = 8.0 Hz, 2H), 4.43 (d, J = 8.1 Hz, 1H), 3.28 (dq, J = 7.8, 6.4 Hz, 1H), 2.43 (s, 3H), 1.33 (ddt, J = 8.2, 6.2, 2.4 Hz, 2H), 1.27 – 1.05 (m, 6H), 1.03 (d, J = 6.5 Hz, 3H), 0.82 (t, J = 7.0 Hz, 3H).

^{13}C NMR (101 MHz, $CDCl_3$) δ 143.3, 138.4, 129.7, 127.2, 50.1, 37.5, 31.5, 25.3, 22.6, 21.9, 21.6, 14.1.

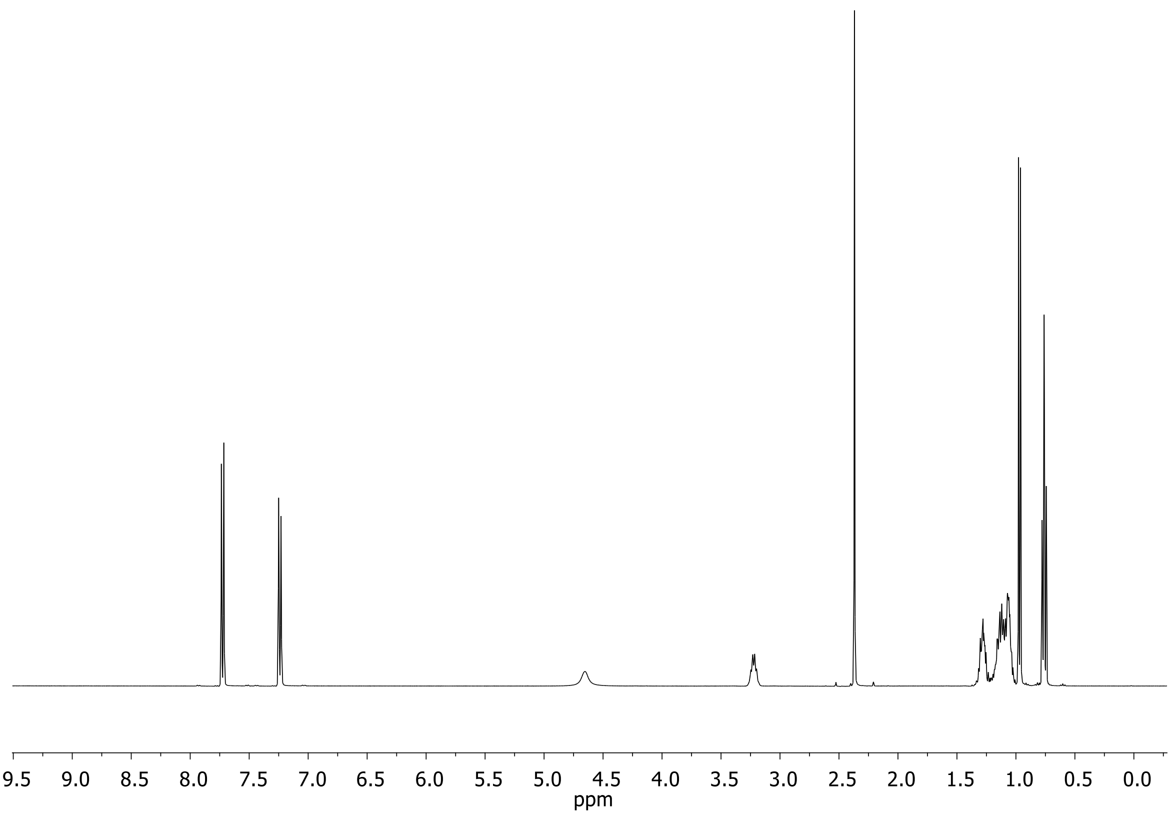

Figure S7. ^{1}H NMR spectra of N-(heptan-2-yl)-4-methylbenzenesulfonamide **11-H**, $CDCl_3$, 400 MHz

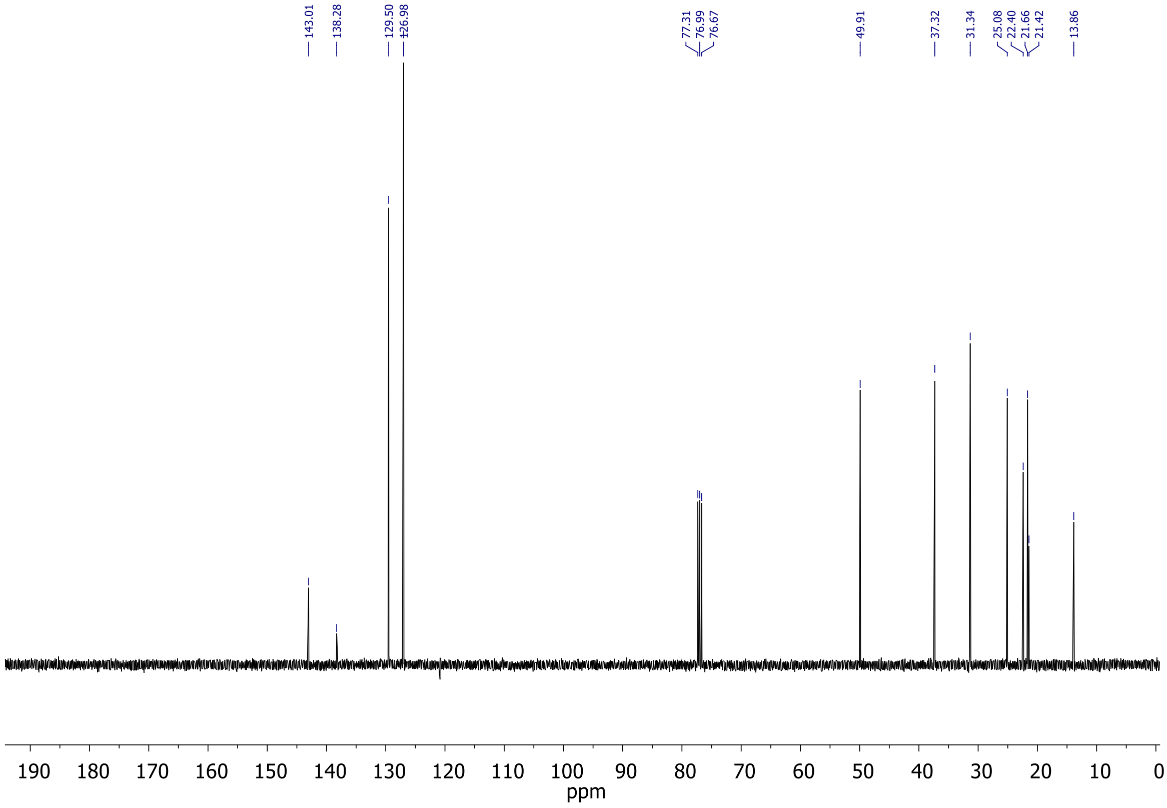

Figure S8. ^{13}C NMR spectra of N-(heptan-2-yl)-4-methylbenzenesulfonamide **11-H**, $CDCl_3$, 400 MHz

General Procedure A: Preparation of *N*-chlorosulfonamides with trichloroisocyanuric acid.

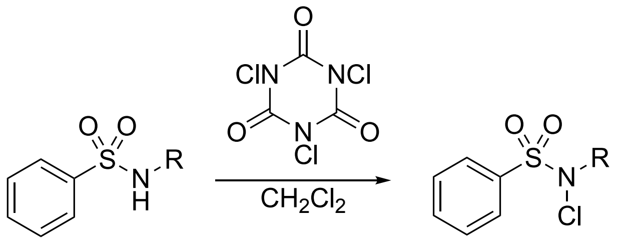

A round bottom flask with a magnetic stir bar was charged with 1 equiv of trichloroisocyanuric acid (TCICA), then evacuated and backfilled with N_2. Anhydrous CH_2Cl_2 was then added to create a suspension. Sulfonamide (1.0 equiv) was then added to the suspension via syringe. The suspension was stirred at room temperature and monitored by TLC until complete consumption of starting material was observed.

Upon complete consumption of starting material as judged by TLC, the reaction was diluted with H_2O and the biphasic suspension was transferred to a separatory funnel. The reaction flask was rinsed with CH_2Cl_2 (5 mL) to ensure quantitative transfer. The organic phase was separated and the aqueous phase was extracted three times with CH_2Cl_2. The combined organic phases were dried with Na_2SO_4, filtered, and concentrated under reduced pressure. The crude material was purified by silica gel column chromatography eluting with a Cyclohexane:EtOAc (85:15) solvent system as noted below.

N-chloro-N-hexyl-4-methylbenzenesulfonamide ***7-Cl***

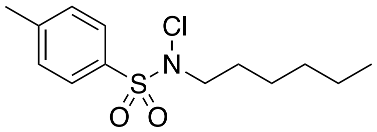

Prepared from *N*-hexyl-4-methyl benzenesulfonamide (985 mg, 3.85 mmol) and TCICA (698 mg, 3 mmol) following general procedure A. The product was obtained as a colorless oil (1054 mg, >94% yield) after silica gel flash column chromatography using cyclohexane:EtOAc (85:15).

^{1}H NMR (400 MHz, $CDCl_3$) δ 7.77 (d, 1H), 7.34 (d, *J* = 8.1 Hz, 1H), 3.17 (t, *J* = 6.9 Hz, 1H), 2.41 (s, 2H), 1.68 – 1.52 (m, 1H), 1.46 – 1.14 (m, 4H), 0.85 (t, 1H).

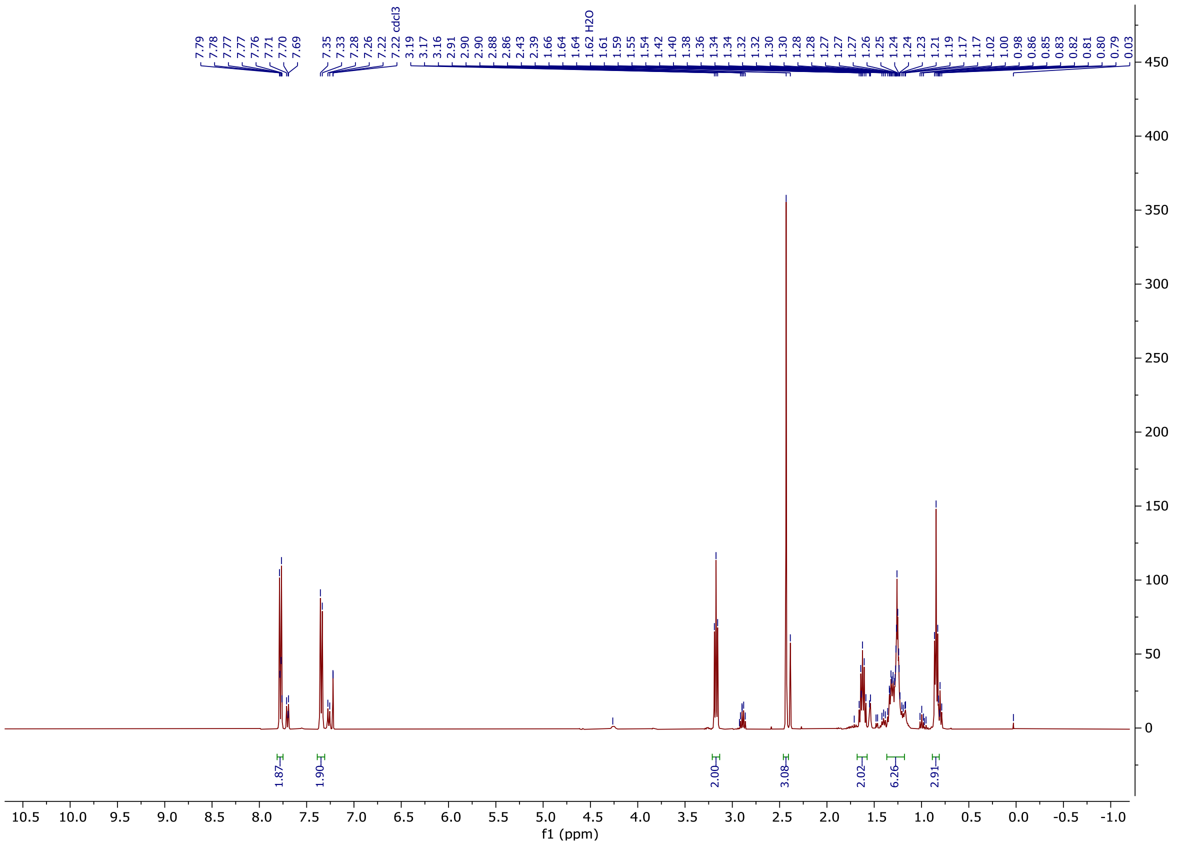

Figure S9. 1H NMR spectra of sulfonamide **7-Cl** in CDCl3, 400 MHz

*N-chloro-N-(heptan-2-yl)-4-methyl-benzenesulfonamide **11-Cl***

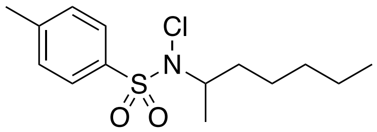

Prepared from *N*-(heptan-2-yl)-4-methyl-benzenesulfonamide (400 mg, 1.48 mmol) and TCICA (400 mg, 1.72 mmol) following general procedure A. The product was obtained as a colorless oil (431 mg, >96% yield) after silica gel flash column chromatography using cyclohexane:EtOAc (85:15).
The structure of the reactant is confirmed from the obtained ^{13}C and ^{1}H spectra which correspond to those found in the literature.[26]

^{1}H NMR (400 MHz, $CDCl_3$) δ 7.78 (d, 1H), 7.31 (d, 1H), 4.43 – 3.83 (m, 0H), 2.41 (s, 2H), 1.32 – 1.19 (m, 1H), 0.99 (d, J = 6.3 Hz, 2H), 0.85 (t, 2H).

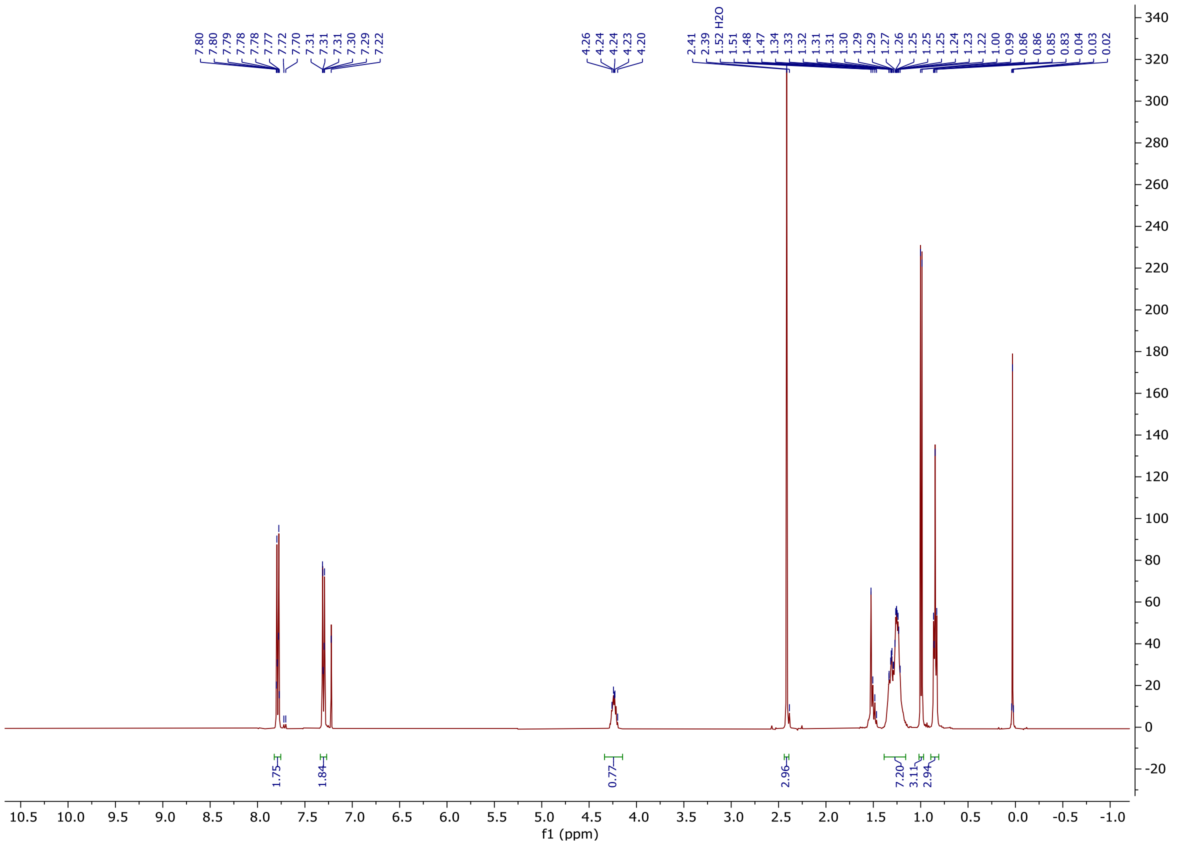

Figure S10. 1H NMR spectra of sulfonamide **11-Cl**, CDCl3, 400 MHz

General Procedure B: Preparation of N-bromosulfonamides with N-bromo succinimide.

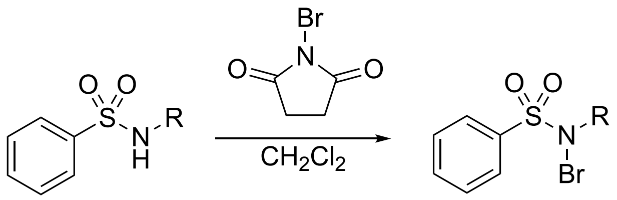

A round bottom flask with a magnetic stir bar was charged with 1.5 equiv of *N*-bromo succinimide (NBS), then evacuated and backfilled with N_2. Anhydrous CH_2Cl_2 was then added to create a suspension. Sulfonamide (1.0 equiv) was then added to the suspension via syringe. The suspension was stirred at room temperature and monitored by TLC until complete consumption of starting material was observed.
Upon complete consumption of starting material as judged by TLC, the reaction was diluted with H_2O and the biphasic suspension was transferred to a separatory funnel. The reaction flask was rinsed with CH_2Cl_2 (5 mL) to ensure quantitative transfer. The organic phase was separated and the aqueous phase was extracted three times with CH_2Cl_2. The combined organic phases were dried with Na_2SO_4, filtered, and concentrated under reduced pressure. The crude material was purified by silica gel column chromatography eluting with a Cyclohexane : EtOAc (85:15) solvent system as noted below.

N-bromo-N-hexylbenzenesulfonamide ***7-Br***

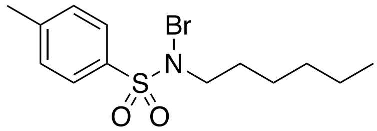

Prepared from *N*-hexyl-4-methylbenzenesulfonamide **7-H** (370 mg, 1.53 mmol) and NBS (408 mg, 2.17 mmol) following general procedure B. The product was obtained as a coloroless oil with a greenish tone to it (323 mg, 66 % yield) after silica gel flash column chromatography using cyclohexane:EtOAc (85:15).

N-bromo-N-(heptan-2-yl)-4-methyl-benzenesulfonamide ***11-Br***

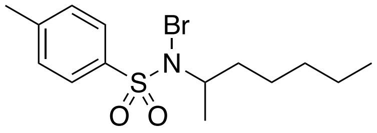

Prepared from *N*-(heptan-2-yl)-4-methyl benzenesulfonamide **11-H** (mg, mmol) and NBS (mg, mmol) following general procedure B. The product was obtained as a colorless oil with a greenish tone to it (, 62 % yield) after silica gel flash column chromatography using cyclohexane:EtOAc (85:15).

Reaction 7-H with PIDA/I_2 in the dark

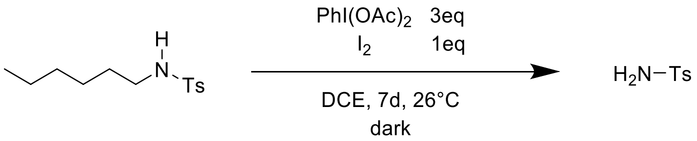

Procedure was modified from Ref 26.

In an oven dried NMR tube under nitrogen atmosphere N-hexyl-4-methylbenzenesulfonamide (25 mg, 0.1 mmol, 1 eq) and phenyliodine(III) diacetate (94.7 mg, 0.3 mmol, 3eq) were dissolved in 1.0 ml of anhydrous $CDCl_3$. After 24.9 mg of iodine (0.1 mmol, 1 eq)) were added to the reaction in the dark, the NMR tube was screw capped and stored under the absence of light for seven days. A crude 400-MHz -^{1}H-NMR spectrum was recorded, after which 1 mL of a sat. aq. sol. of sodium thiosulfate is added to the reaction mixture and mixed until the violet color disappears. The aqueous phase is extracted three times with 3 mL of ethyl acetate, the combined organic phases are dried over anhydrous $MgSO_4$ and the solvent was removed under vacuo to afford the crude product. The crude mixture was purified by silica gel chromatography (9:1/i-Hexane:EtOAc) to afford tosyl amide in almost quantitative yields (15.1 mg, 0.09 mmol, 90%).

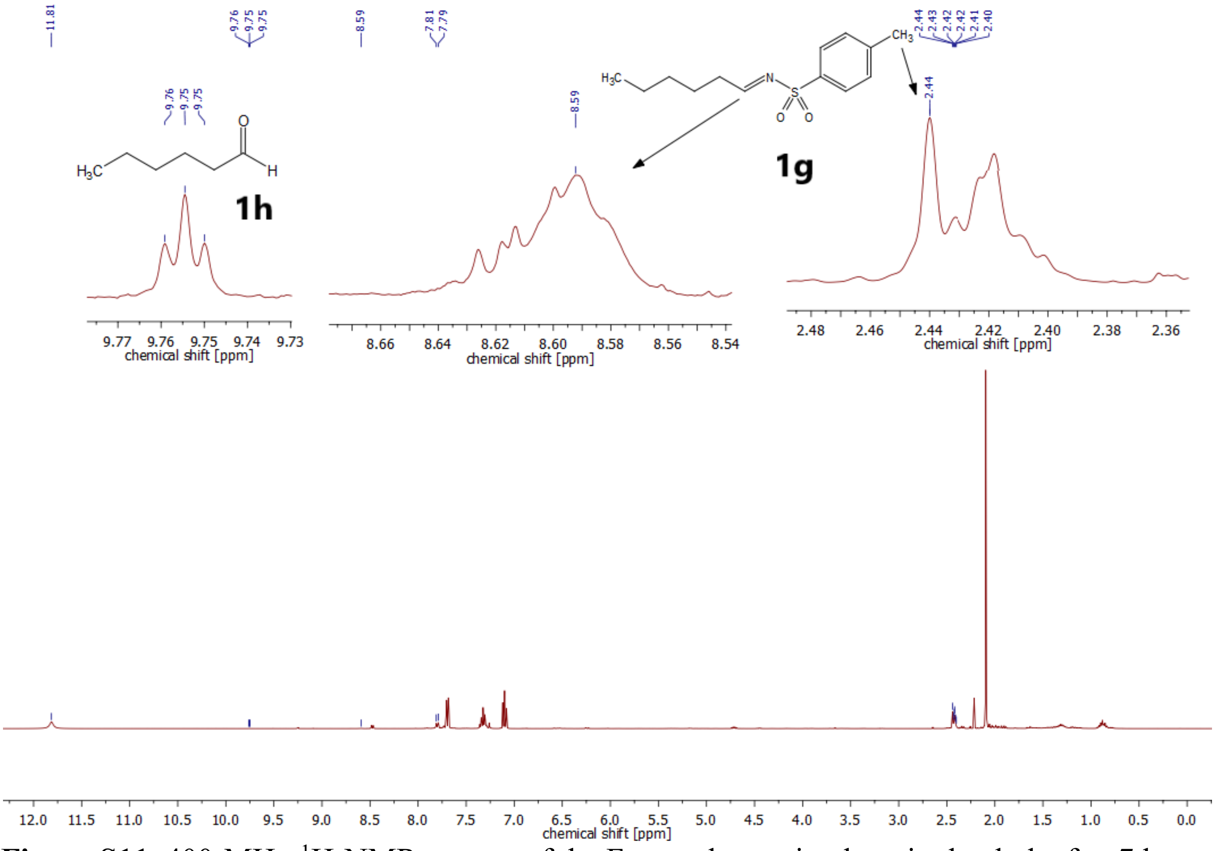

Figure S11. 400-MHz-^{1}H-NMR spectra of the Fan et al. reaction kept in the dark after 7d.

Reaction 7-H with PIDA/I_2 under irradiation

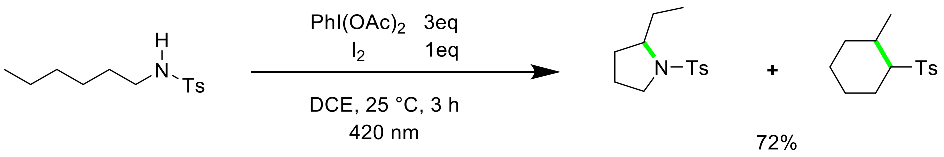

Procedure was modified from Ref. 26 in MS.

General Procedure GP1: An oven dried 10 ml GC vial equipped with a magnetic stir bar is placed in a desiccator that was evacuated and filled with nitrogen for three cycles. To GC-vial is then added under continuous nitrogen flow the N-hexyl-4-methylbenzenesulfonamide (100 mg, 0.39 mmol, 1eq), PIDA (392 mg, 1.17 mmol, 3 eq) and the anhydrous degassed solvent. After 99.4 mg of Iodine (0.39 mmol, 1eq) is added quickly, the GC vial is capped under nitrogen and then transferred to the photoreactor with a preset temperature and light source (420 mn). After 3h of stirring 10 mL of a sat. aq. sol. of sodium thiosulfate is added to the reaction mixture and stirred for 1h. The aqueous phase is extracted three times with 20 mL of ethyl acetate, the combined organic phases are dried over anhydrous $MgSO_4$ and the solvent was removed under vacuo to afford the crude product. The crude mixture was purified by silica gel chromatography (9:1/i-Hexane:EtOAc) to afford 2-Ethyl-1-tosylpyrrolidine and 2-Methyl-1-tosylpiperidine as a product mixture (72%).

Identification by ^{1}H-NMR spectroscopy

As see in Figure 12, the 2-methyl-1-tosylpiperidine is identified by characteristic ^{1}H-NMR signals in the 1,5- and 1,6-HAT cyclisation product mixture besides 2-ethyl-1-tosylpyrrolidine after purification by flash chromatography.

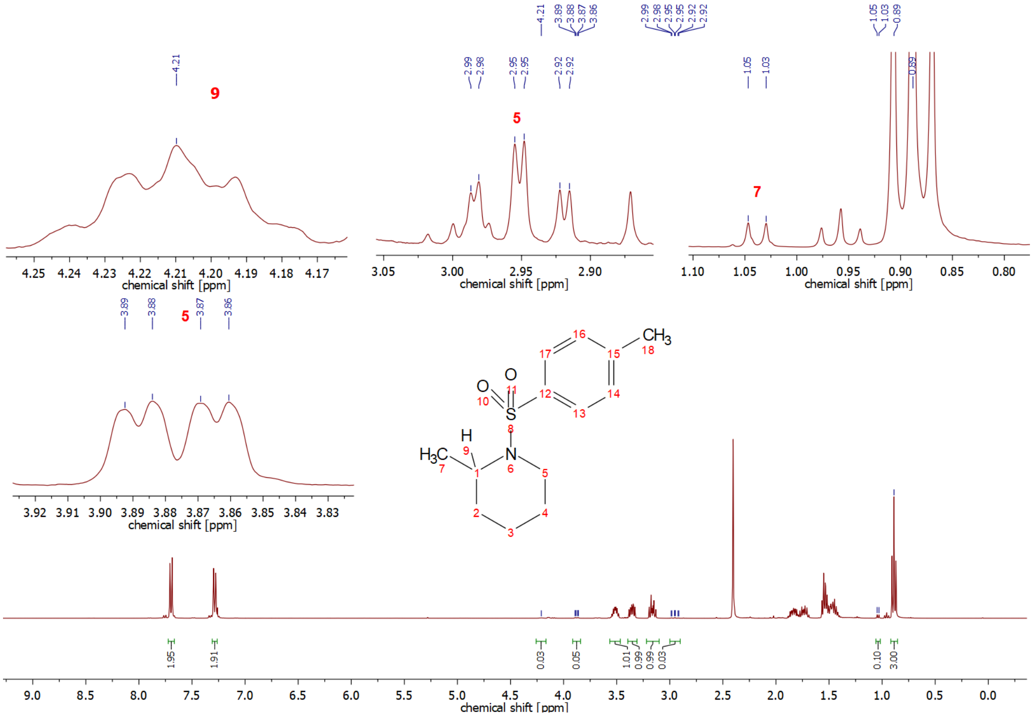

Figure S12. Proof of the existence of 1,6- besides the 1,5-HAT cyclisation product in the Fan et al. reaction after purification by flash chromatography (400MHz-^{1}H-NMR).

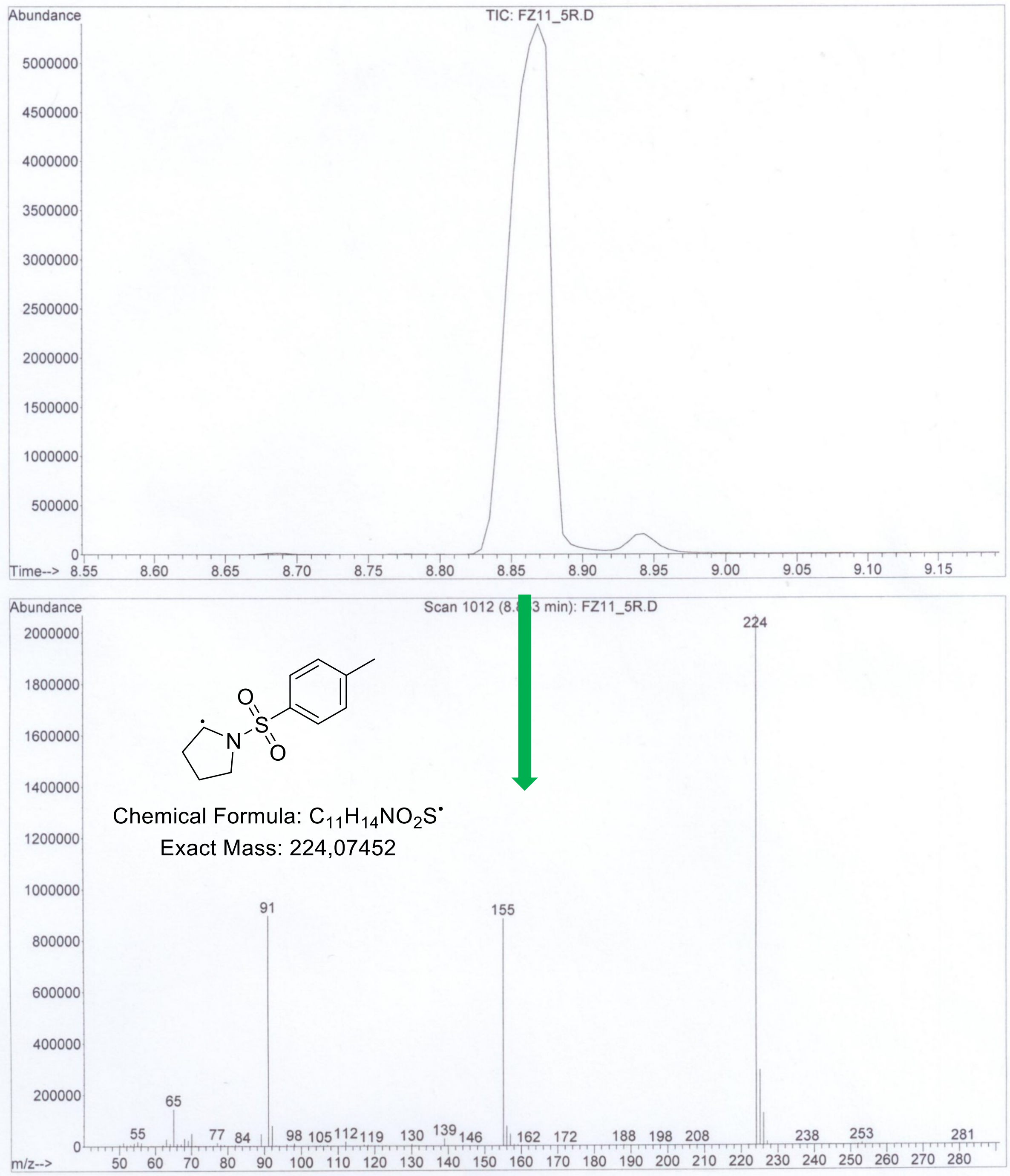

Figure S13. Top: Zoomed-in GC-MS spectrum Bottom: mass spectrum of peak at 8.863 min of retention time with fragment of highest abundance depicted (1,5-HAT product).

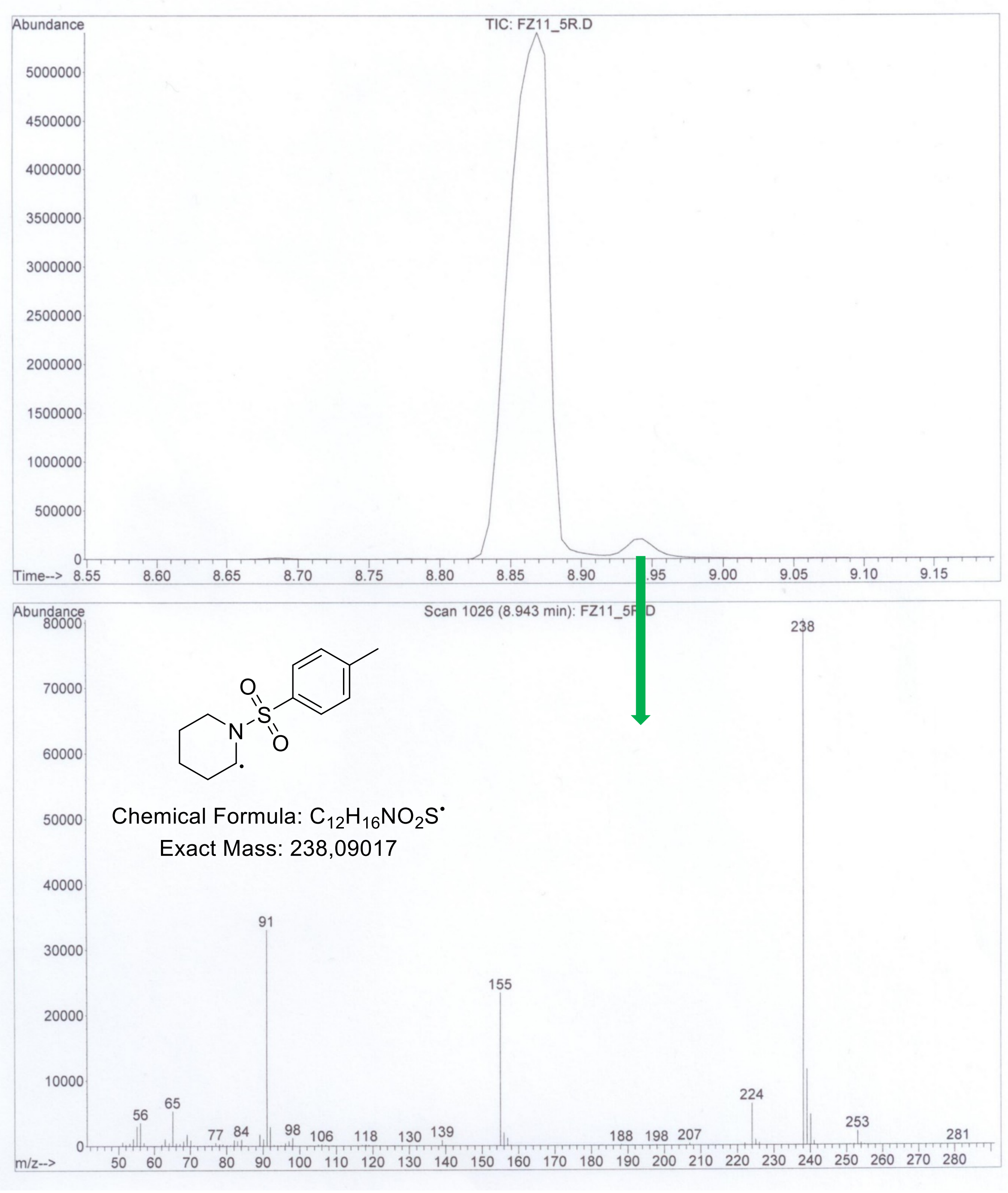

Figure S14: Top: Zoomed-in GC-MS spectrum Bottom: mass spectrum of peak at 8.943 min of retention time with fragment of highest abundance depicted (1,6-HAT product).

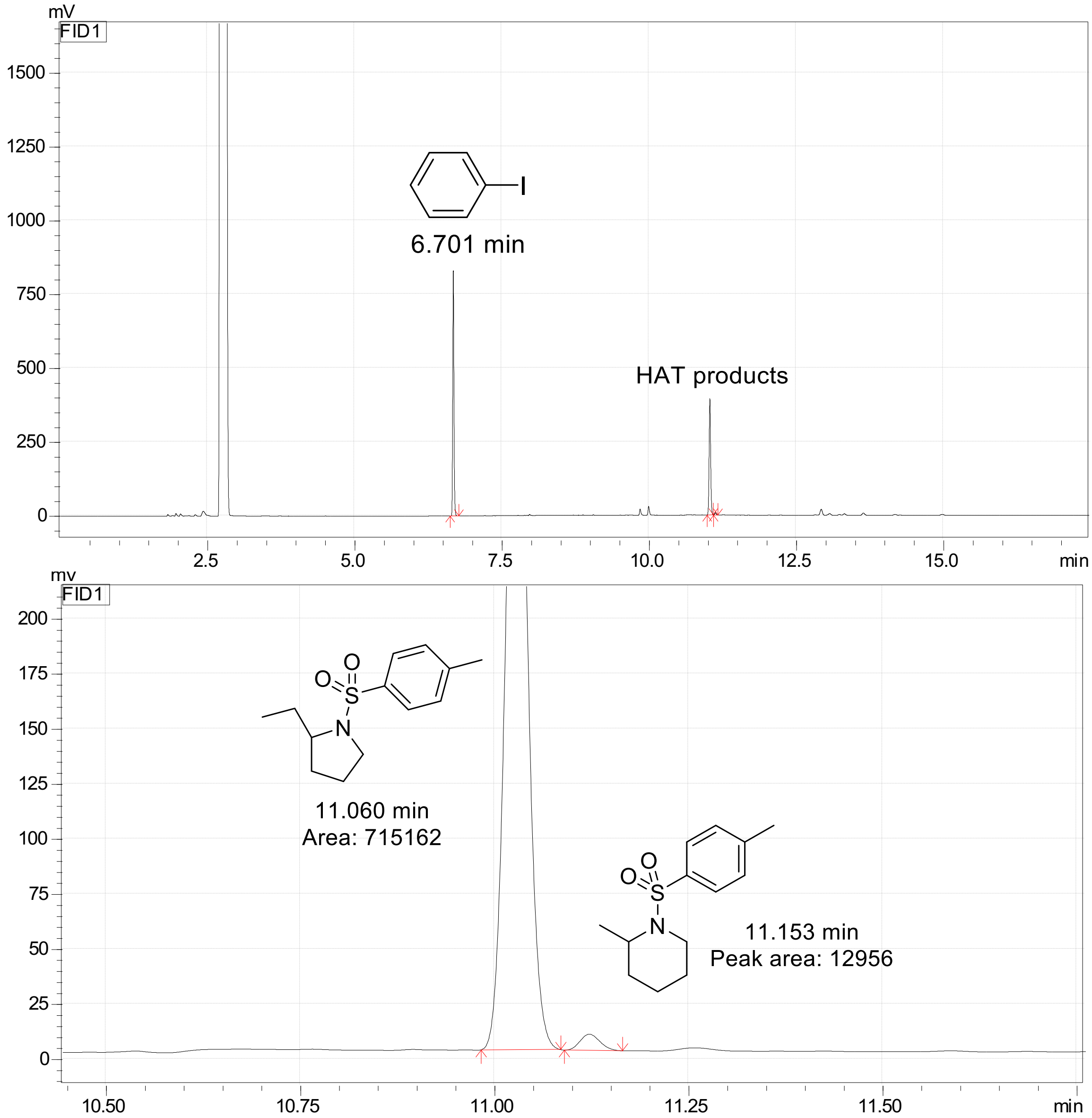

Figure S15. GC-FID under Fan et al. conditions with description of important signals.

Degassing procedure and EPR measurements

The vacuum line was turned on preferably the day before use (this is only necessary for working on high vacuum, 10^{-6} mm Hg, with the use of an additional diffusion pump otherwise it can be turned on the day of use, at least 2 hours before usage). After every usage of the manifold, trap of the vacuum line was disassembled and washed with methanol while the vacuum grease was removed with isopropanol. Trap was then dried in a drying oven. For the preparation of working solutions, Schlenk flasks were used. First step in sample preparation was stock solution preparation in the following manner. Compound (approx. 30 mg) and spin trap (PBN) (approx. 10 mg) were weighed separately on an analytical balance and transferred quantitively to corresponding Schlenk flasks. To each flask approx. 0.3 mL of solvent (toluene or heptane) was added. Schlenk flask was then attached to the vacuum line via a joint that has been greased. The joint was covered with parafilm and fixed with a suitable plastic extension to ensure it was hermetically sealed. The stopcock of the Schlenk flask was then closed. Flask was emersed in liquid nitrogen and evacuated by opening of the main cap on the vacuum line and the cap leading to the flask. Vacuum was then created above the frozen liquid. Liquid nitrogen was removed from underneath the flask and the cap leading to the flask was closed while the solution was left to defrost. Once the solution was defrosted liquid nitrogen was added again. As soon as the solution was frozen again the cap leading to the flask was opened. Whole cycle was repeated as many times as necessary for each stock solution. After degassing, positive pressure of nitrogen gas was created by opening of the stopcock, the flask was then detached from the vacuum line and closed with an appropriate plug. The same procedure was done for the solution of PBN in solvent. Finally, both flasks were transferred to a glovebox in which they were mixed by transferring a small amount to an EPR tube.

EPR measurements **7-I**

Two different stock solutions were prepared. Into the first flask *N*-hexyl-4-methylbenzenesulfonamide **7-H** (8 mg, 0.0313 mmol), (Diacetoxyiodo)benzene (PIDA, 13mg, 0.0404 mmol), I_2 (0.05 mL solution of 1M degassed stock solution made from iodine granules) and toluene or heptane (0.3 mL) were added. Into the second flask PBN (8 mg, 0.0045 mmol) and toluene or heptane (0.3 mL) were added. Inside a glovebox, small volumes from both solutions were transferred to an EPR tube which was then used for the measurement.
The EPR measurements were performed on a Bruker ELEXSYS E500 spectrometer with an ER049X (superX) microwave bridge and an original ER4122SHQE cavity resonator.
Typical measurement parameters are as follows: microwave frequency ~ 9.85 GHz (as recorded by the spectrometer), microwave power 10 mW, modulation frequency 100 kHz, modulation amplitude 0.1 G, lock-in amplifier time constant 10.24 ms, sweep range 60 G, sweep time 20.97 s, one sweep at each time point, and 5s waiting time between points.

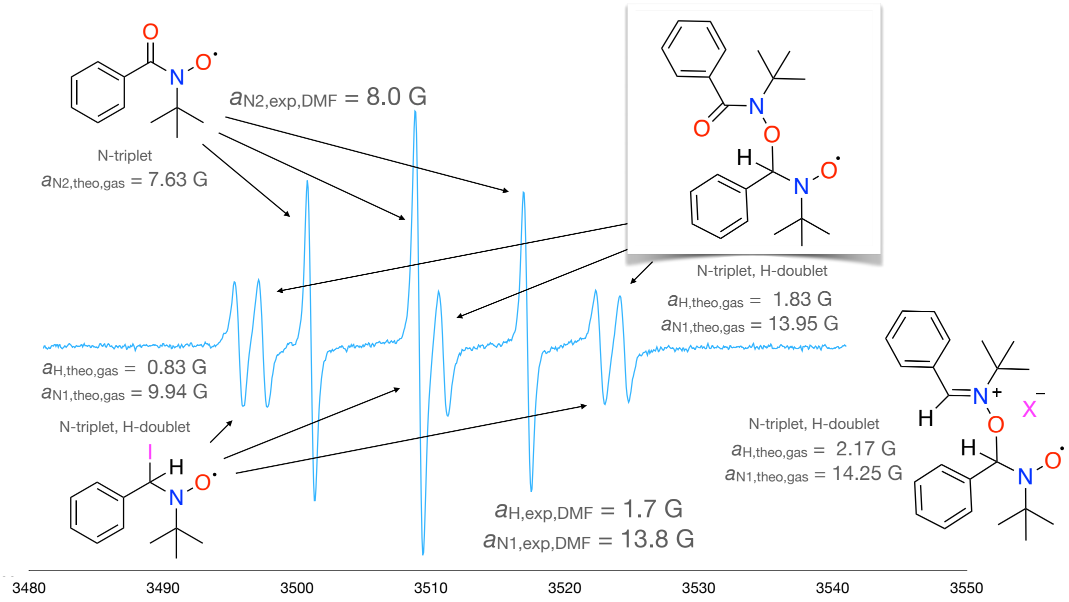

Figure S16. EPR measurement of PBN spin-trapped intermediates of **7-I** in the HLF reaction. Compound **7-I** was generated *in situ* from **7-H**, PIDA and I_2, using procedure described in literature. [27]

Although two distinct radicals are formed, both belong to the oxidation products of spin-trap PBN. Namely, **oxo-PBN** is the compound whose EPR signal has the typical *g*-factor of 2.0069 characteristic of nitroxide radical and hyperfine coupling of 8.0 G from nitrogen atom, making it a triplet. This compound was described earlier in the literature and is observed in larger quantity in other EPR measurements after prolong irradiation. Additional radical, with slightly different *g*-factor (2.0059) is still a nitroxide radical, but with distinct H-atom and N-atom couplings, making it a triplet of doublets. Different options depicted in Figure S16 were investigated using DFT calculations, but only **(oxo-PBN)-PBN** adduct has good enough fit to experimental results. See below for calculation details, geometries and calculated EPR parameters for these compounds. Oxidation pathway, from **X-PBN** (with X being Cl, Br and I) to **oxo-PBN** and **(oxo-PBN)-PBN** will be explored in subsequent research.

EPR measurements **7-Br**

Two different stock solutions were prepared. Into the first flask *N*-bromo-*N*-hexyl-4-methylbenzenesulfonamide **7-Br** (38 mg, 0.012 mmol) and toluene or heptane (0.3 mL) were added. Into the second flask PBN (7 mg, 0.004 mmol) and toluene or heptane (0.3 mL) were added. Inside a glovebox, small volumes from both solutions were transferred to an EPR tube which was then used for the measurement.

The deoxygenated solutions were sealed in the Schlenk flasks and transferred into a glovebox charged with nitrogen gas (an erLab Captair 2200A pyramid glove bag charged with nitrogen. The glove bag was then charged and discharged multiple times until the oxygen concentration reached below one percent of the air level). Small volumes of both solutions, typically 0.1 mL (actually amounts depend on the solution available in each Schlenk flask), were pipetted into one 5 mm PYREX NMR tube under the nitrogen atmosphere and sealed by a Sigma-Aldrich Precision Seal rubber septa plug. The EPR measurements were performed on a Bruker ELEXSYS E500 spectrometer with an ER049X (superX) microwave bridge and an original ER4122SHQE cavity resonator. In-situ UV light illumination was provided by a Kessil 370 nm gen 2 LED UV lamp from the bottom port of the resonator. The spectra were usually collected shortly before, during, and after UV illumination as a two-dimensional time delay experiment. Typical measurement parameters are as follows: microwave frequency ~9.85 GHz (as recorded by the spectrometer), microwave power 10 mW, modulation frequency 100 kHz, modulation amplitude 0.1 G, lock-in amplifier time constant 10.24 ms, sweep range 60 G, sweep time 20.97 s, one sweep at each time point, and 5s waiting time between points. The total number of points was usually set to cover(much) longer than the time needed for the main reaction to finish. (The receiver gain needed to be lowered from 60 dB initially to 40 dB near the later peak of the oxo-PBN signal to avoid saturation.)

The thermal drifts of the magnetic field were compensated in the post processing by aligning the points using Cl-PBN and/or oxo-PBN signals as internal standard(s). Simulations were performed on averages exacted from the relevant period(s) of the aligned spectra using the EASYSPIN package. Experimental and simulated spectra are denoted at the bottom as blue and purple traces, respectively. Another, approximately constant spectrometer bias ($\Delta g \approx$-0.000205) was calibrated by measuring standard samples solid 2,2-diphenyl-1-picrylhydrazyl (DPPH) radical (Bruker, 0103D139) and the aqueous solution of the peroxylamine disulfonate (PADS, i.e., Frémy's salt) radical offline and used to produce the g-factors listed.

Deconvolution procedure:

As we have a complicated EPR spectra of the mixture, we had to use deconvolution techniques to gain insights about this mixture of radicals. There are several modes to deconvolute the same spectra, but we have used one major premise – use deconvolution to gain the least number of radicals. Additionally, we have compared our deconvolution results with results from quantum-chemical calculations.

Our simulation was not a global search over all possible combinations for each compound independently. Instead, we did conditional fitting of EPR spectra based on observations made from EPR spectra without simulations as well as NMR spectra. Our main observations before simulations are:

1) **7-Cl** generated spectra which are, qualitatively, **7-Br** spectra plus **Cl-PBN** and **oxo-PBN**, if the latter is visible.
2) **7-Br** spectra in two different solvents used, namely toluene and n-heptane, are similar to each other.
3) Chlorine was found to have been recombined to C_2 and C_5 positions by the NMR spectroscopy.

We therefore chose to fit first the **7-Br** spectra with the following conditions:

1) There should be just one *N*-centered radical abduct as the design of **7-Br** supported.
2) NMR results suggested that there could be two *C*-centered radical intermediates. We try to use as few as possible *C*-centered abducts in the decomposition.
3) The proposed spin abducts and their hyperfine couplings should explain the majority of the results of **7-Br** spin trapping experiments in both toluene and n-heptane with only minor adjustments.

Resulting spectra can be decomposed in at least two ways:

1) One *N*-centered radical, with triplet from N-atom in PBN, doublet from Hα-atom in PBN, and triplet splitting from N-atom from the **7**. Here splitting from H-atom ($\alpha_{H,exp}$) is larger than splitting from N-atom ($\alpha_{N\prime,exp}$) of **7**, due to larger spin population on H-atom. Two different *C*-centered radical types are formed, which have roughly the same *g*-factor, but different *hfc* parameters. This is shown below and in Figure S17. Additional experiment in *n*-heptane at two time points are shown in Figure S18 and Figure S19. This deconvolution has a strong foundation in extensive quantum-chemical calculations (see EPR calculations on PBN adducts section in the SI)
2) Alternative deconvolution (see Figure S20), where similar parameters for *N*-centered radical is obtained. Again, two different C-centered radical adducts are observed, but here similar *hfc* parameters are used, with different *g*-factors for each type of *C*-centered radical (Δg = 0.0015).

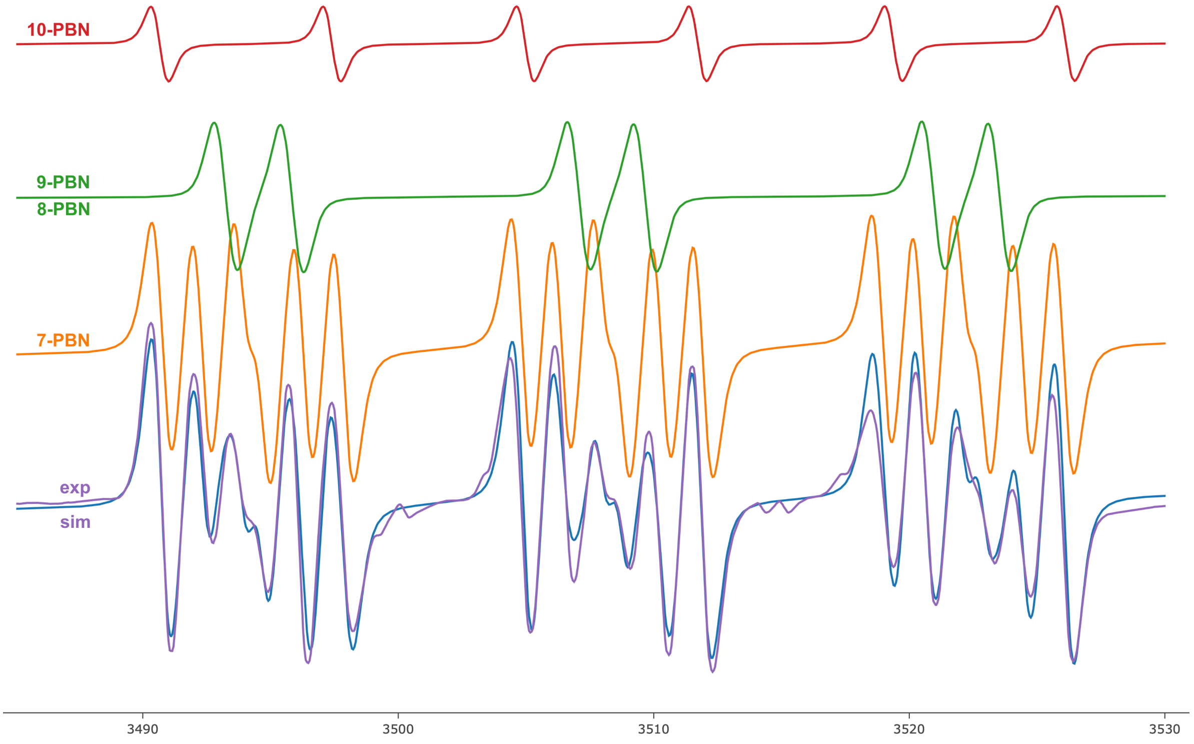

Figure S17. EPR spectra of spin-trapped radical intermediates generated with 370 nm irradiation of **7-Br** in toluene. Experimental spectra is in blue color, while red, green, and orange correspond to simulated spectra for **10-PBN**, **8-PBN** and **7-PBN**, respectively.

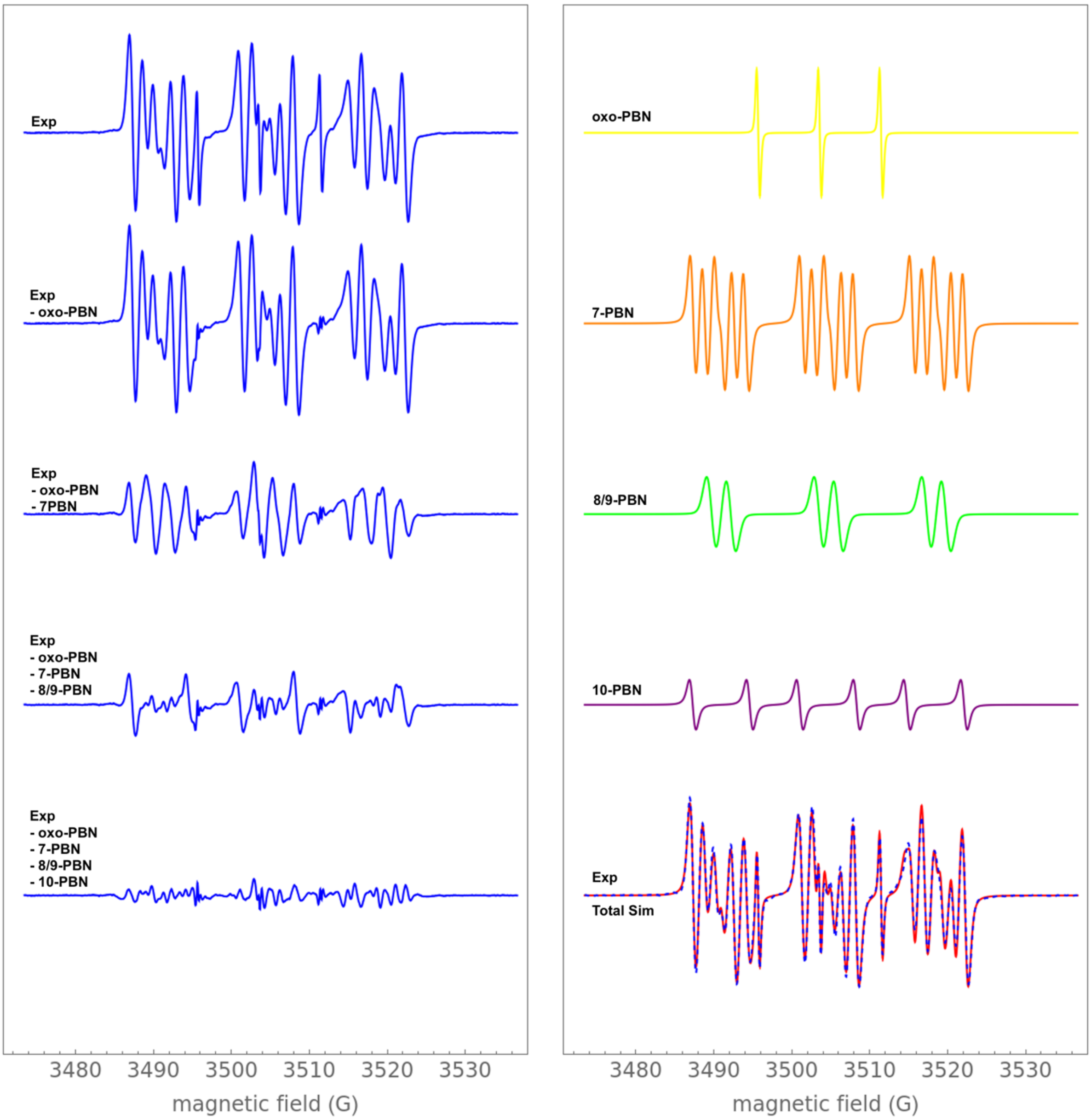

Figure S18. The deconvolution components and residues of the EPR spin abduct spectrum of the **7-Br** spin trapping experiment in *n*-heptane. Left panel shows from upper to lower the experimental spectrum and the residue after each of the deconvolution components on the right panel is subtracted. Right panel shows, from upper to lower, the **oxo-PBN** (yellow), 7-PBN (orange), 8/9-PBN (green), and 10-PBN (purple) components of the deconvolution, as well as a comparison between the total simulated spectrum (red) and the experimental spectrum (blue, dashed) at the bottom.

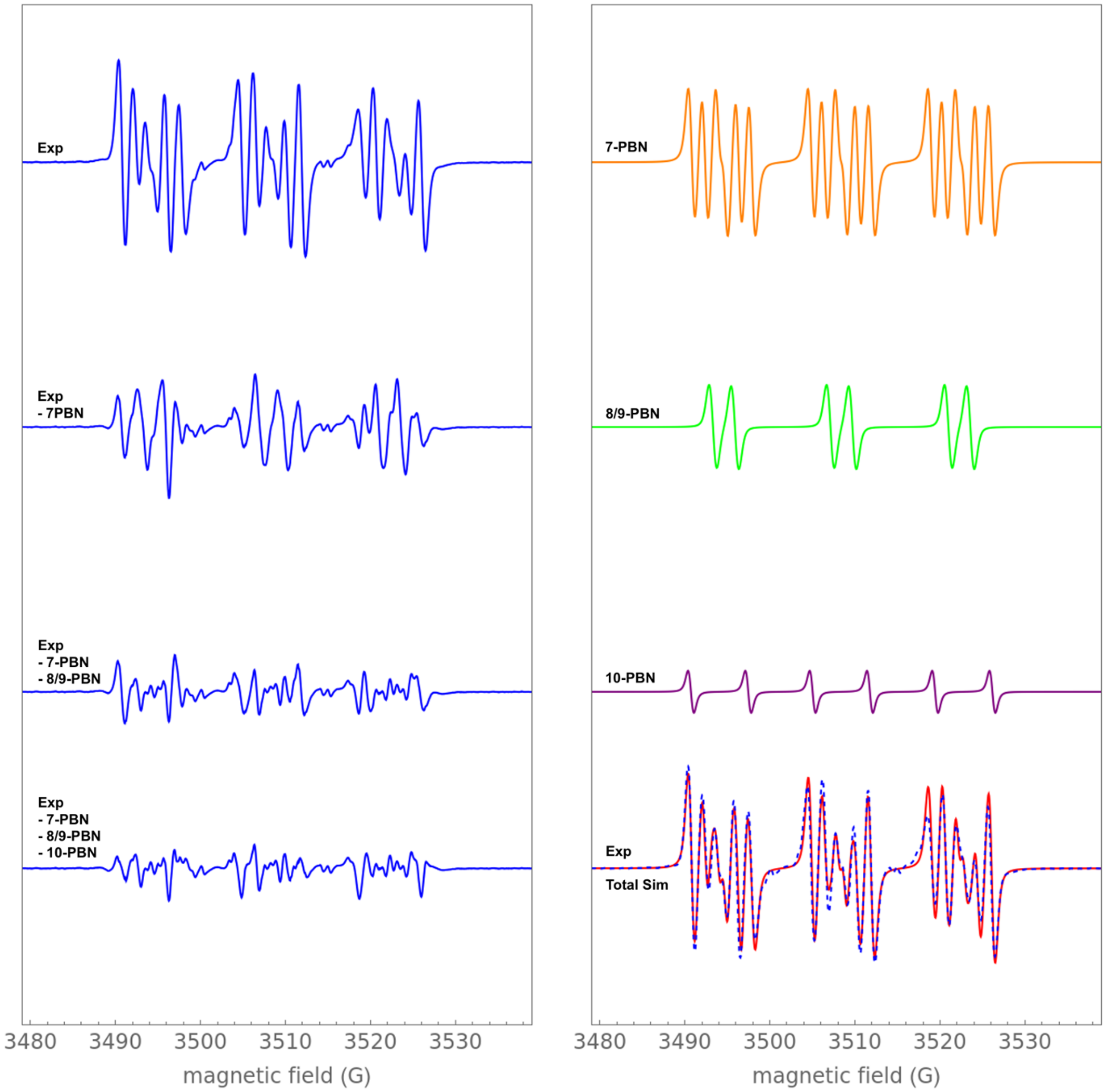

Figure S19. The deconvolution components and residues of the EPR spin adduct spectrum of the **7-Br** spin trapping experiment in *n*-heptane when **oxo-PBN** has not been produced. Left panel shows from upper to lower the experimental spectrum and the residue after each of the deconvolution components on the right panel is subtracted. Right panel shows, from upper to lower, **7-PBN** (orange), **8/9-PBN** (green), and **10-PBN** (purple) components of the deconvolution, as well as a comparison between the total simulated spectrum (red) and the experimental spectrum (blue, dashed) at the bottom.

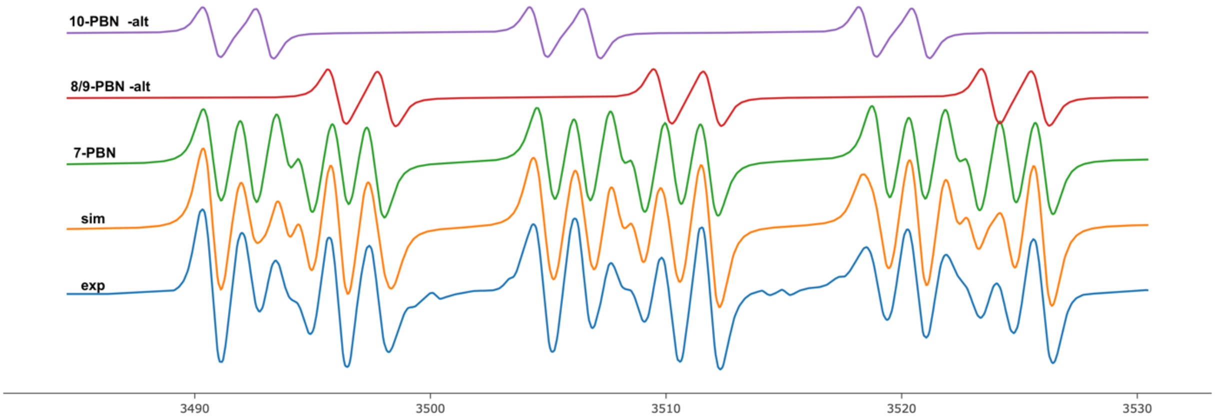

Figure S20. Alternative deconvolution of **7-PBN** spectra from **7-Br**. Red and green lines correspond to two different *C*-centered radical adducts to the PBN, with different g-factors.

Using computational approach (see below in PBN adduct section), we have determined that the first way of deconvolution is more probable. Calculated *g*-factors for two types of *C*-centered radicals differ less than 0.00015, and thus provide a more realistic explanation.
In Figure S21, component kinetic history is presented, where comparison of kinetics in different solvents for different components can be assessed.

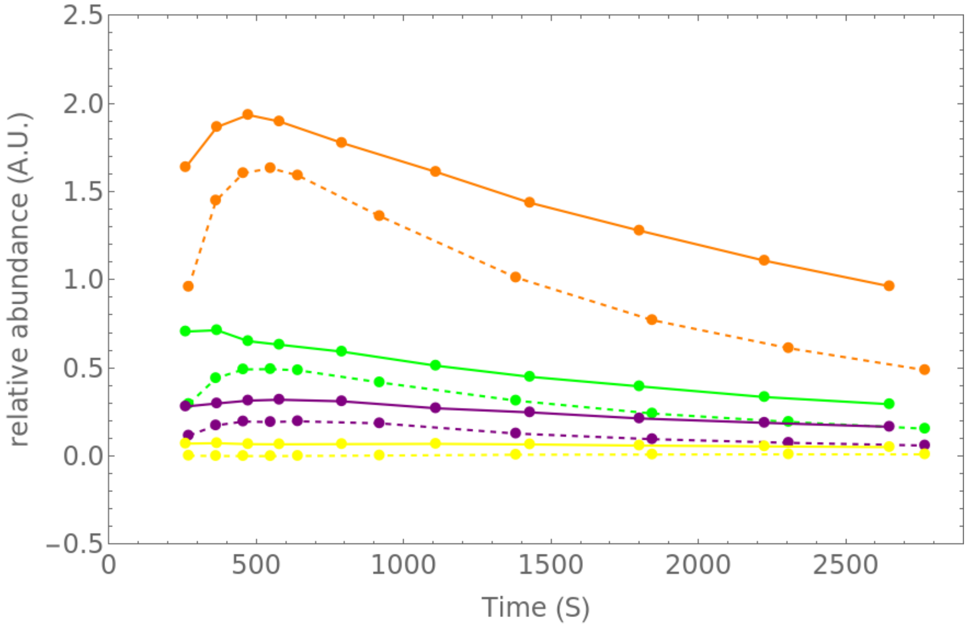

Figure S21. The component kinetic history over the initial period (from light on to 40 minutes later) of **7-Br** spin trapping experiments. Dashed plots are from the experiment in toluene, solid plots are from the experiment in *n*-heptane. The color markers are yellow for **oxo-PBN**, orange for **7-PBN**, green for **8/9-PBN**, and purple for **10-PBN**. The relative weights of the components were not so different during the period studied. The stability of the spin adducts appeared to be lower in toluene than in n-heptane.

EPR measurements **7-Cl**

Two different stock solutions were prepared. Into the first flask *N*-chloro-*N*-hexyl-4-methylbenzenesulfonamide **7-Cl** (42 mg, 0.036 mmol) and toluene or heptane (0.3 mL) were added. Into the second flask PBN (10 mg, 0.0056 mmol) and toluene or heptane (0.3 mL) were added. Both solutions were deoxygenated by the freeze-pump-thaw method already described. We deconvoluted the **7-Cl** spectra by a local least square fit using the template **7-Br** + **Cl-PBN** + **oxo-PBN** if necessary.

The rest of the sample loading, measuring, and analyzing procedures are essentially the same as **7-Br**.

Cl-PBN adduct was first identified in the spectra. EPR parameters of this radical adduct correspond to the literature references.[28] Shown as orange trace in Figure S22.

Simulated values: g_{exp}= 2.0077, $\alpha_{N,exp}$ = 12.36 G, $\alpha_{Cl,exp}$ = 6.24 G, and $\alpha_{H,exp}$ = 0.74 G.

7-PBN is characterized by large triplet splitting coming from N-atom in PBN. Doublet splitting comes from H-atom of PBN, while third triplet splitting comes from N-atom in from fragment stemming from **7**. Shown as green trace.

Simulated values: g_{exp}= 2.0064, $\alpha_{N,exp}$ = 14.12 G, $\alpha_{H,exp}$ = 3.89 G, and $\alpha_{N\prime,exp}$ = 1.60 G

8-PBN and **9-PBN** are both indistinguishable *C*-centered alkyl radicals. Shown as red trace.

Simulated values: g_{exp}= 2.0064, $\alpha_{N,exp}$ = 13.85 G, $\alpha_{H,exp}$ = 2.57 G,

10-PBN is a *C*-centered radical adjacent to amide. Shown as purple trace.

Simulated values: g_{exp}= 2.0064, $\alpha_{N,exp}$ = 14.46 G, $\alpha_{H,exp}$ = 6.78 G,

oxo-PBN is a side product from oxidation of Cl-PBN with another PBN. Shown as brown trace.

Simulated values: g_{exp}= 2.0071, $\alpha_{N,exp}$ = 7.97 G

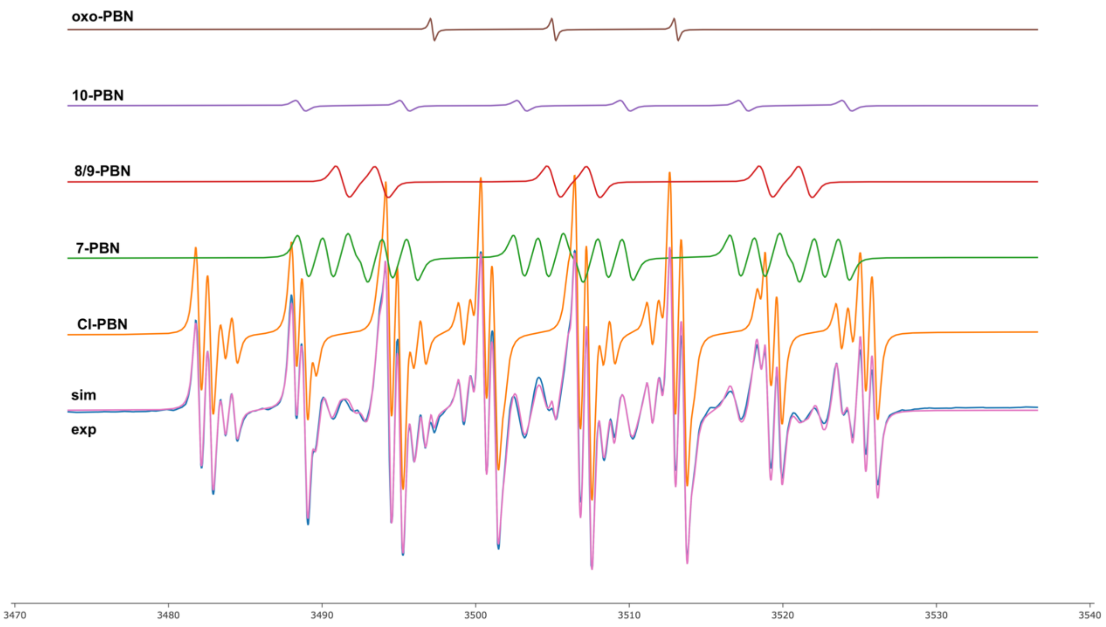

Figure S22. EPR spectra of **7-Cl** in toluene with spin trap PBN and constant illumination

Preliminary SVD procedure (using python3 with pandas, numpy and scipy packages and included SVD analysis, using formula a == U @ S @ Vh) quickly identified **oxo-PBN** spectra in later stages, which introduced correction errors in other singular vectors of spectra (see Figures S23 and S24).

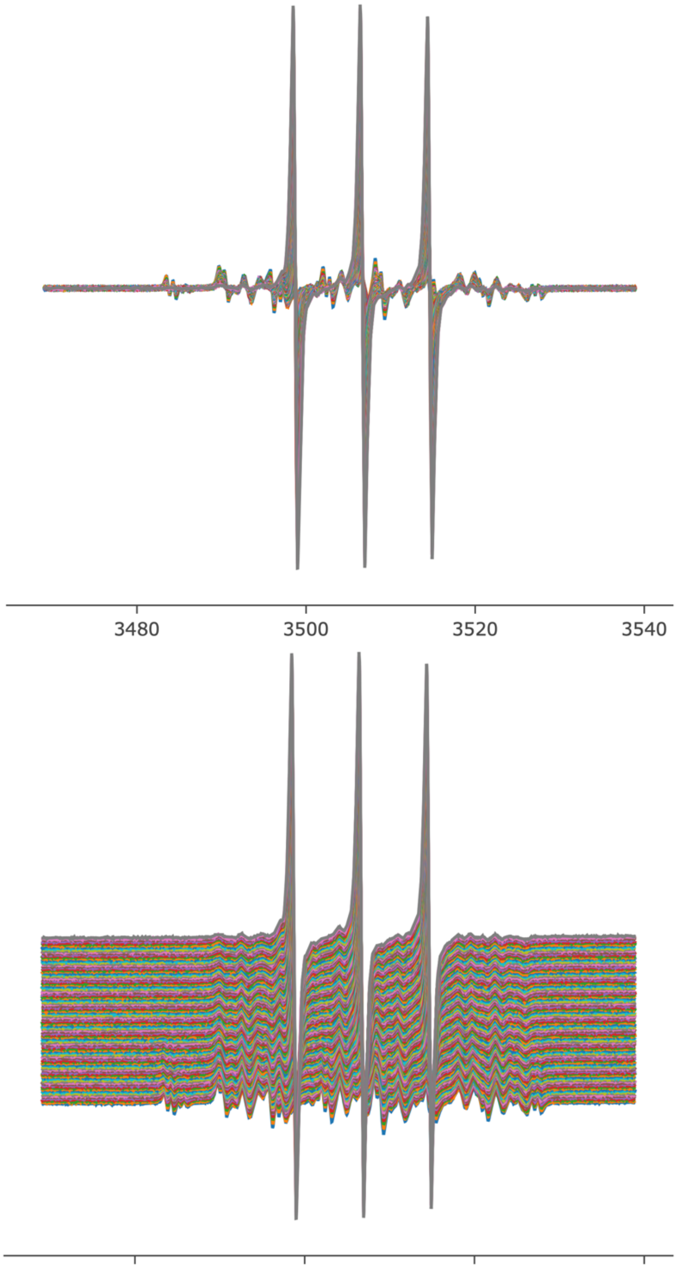

Figure S23. EPR spectra of **7-Cl** recorded in time. Top without offset, bottom with offset. Dominance of late-stage **oxo-PBN** with distinct 8 G separated triplets is observed.

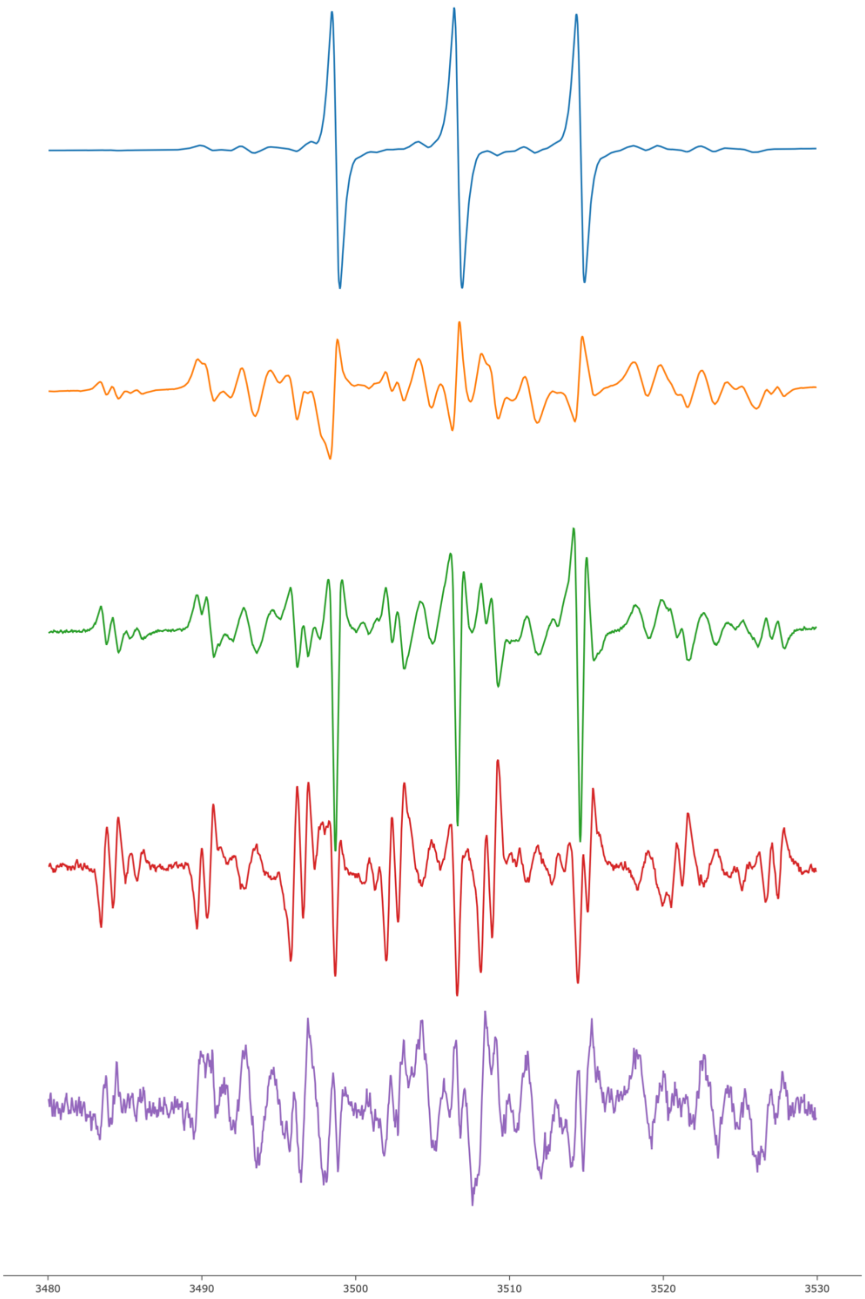

Figure S24. Components of preliminary single value decomposed EPR spectra of **7-Cl** recorded in time. The matrix (U) was reduced to top 5 traces by sigma values. Traces are ordered from top (largest sigma value) to bottom (smallest sigma value). Domination of oxo-PBN triplets with 8 G *hfc* in the first trace introduces artefacts in other traces.

Table S1. Sigma values (S) from SVD

Trace	Raw data	Normalized
1	1036.044995644640	0.763882924167518
2	244.23919749177000	0.180079198452433
3	52.583172429660700	0.0387699257140675
4	17.489236487141000	0.0128949313643873
5	5.931073540171580	0.00437302030159398

Table S2. *Vh values from SVD*

Time Point	Trace1	Trace2	Trace3	Trace4	Trace5
**0**	0.01237384	0.14911143	0.21701243	-0.50493384	-0.25534084
**1**	0.01246286	0.14723518	0.19151665	-0.40932060	-0.12812903
**2**	0.01254871	0.14568244	0.17279813	-0.31232354	-0.03782068
**3**	0.01271418	0.14371488	0.15383632	-0.22674775	0.03915556
**4**	0.01272968	0.14275203	0.14019091	-0.16742894	0.07079576
**5**	0.01279522	0.14185169	0.13015907	-0.12180217	0.09360932
**6**	0.01297452	0.14061708	0.12060964	-0.08471310	0.13524614
**7**	0.01306037	0.13903204	0.11175286	-0.05031603	0.14572053
**8**	0.01303522	0.13881108	0.10658331	-0.01770421	0.18162324
**9**	0.01308496	0.13823551	0.09837032	0.00333782	0.20594003
**10**	0.01323872	0.13814265	0.09307090	0.02657005	0.22228263
**11**	0.01321758	0.13708803	0.08824556	0.04100378	0.18751879
**12**	0.01344025	0.13611398	0.08277337	0.05747156	0.18772244
**13**	0.01337157	0.13588659	0.08070556	0.07411172	0.17667021
**14**	0.01356357	0.13535866	0.07651038	0.08289869	0.16951891
**15**	0.01361022	0.13487199	0.07517489	0.09406482	0.16265139
**16**	0.01375957	0.13484814	0.07411559	0.10162777	0.15130821
**17**	0.01389047	0.13357177	0.06947895	0.10715137	0.13145293
**18**	0.01403980	0.13321430	0.06789417	0.11544218	0.10780295
**19**	0.01420863	0.13347796	0.06735232	0.11553429	0.09187598
**20**	0.01451711	0.13247311	0.06732098	0.10762266	0.06042405
**21**	0.01462383	0.13258015	0.06410409	0.11582228	0.04261502
**22**	0.01478524	0.13202833	0.06203880	0.11021035	0.03498028
**23**	0.01517522	0.13149986	0.05942290	0.10432694	0.01666667
**24**	0.01561649	0.13053734	0.05932060	0.10537433	-0.02496166
**25**	0.01590473	0.13036666	0.05678108	0.10531575	-0.07606041
**26**	0.01660614	0.12873186	0.05226185	0.10105990	-0.08836631
**27**	0.01714470	0.12838853	0.05117091	0.10100412	-0.09648707
**28**	0.01779375	0.12723684	0.04794442	0.10461243	-0.13795916
**29**	0.01850145	0.12658591	0.04417185	0.09355701	-0.17289346
**30**	0.01953433	0.12508199	0.03593186	0.08651258	-0.16659020
**31**	0.02032301	0.12362191	0.03140257	0.08009435	-0.18727377
**32**	0.02132172	0.12274869	0.02699157	0.07987099	-0.17035750
**33**	0.02253502	0.12068050	0.02133543	0.07862353	-0.20453794
**34**	0.02369867	0.11899773	0.01470254	0.08191561	-0.19356203
**35**	0.02568597	0.11651879	0.00499376	0.07287742	-0.18710582
**36**	0.02708183	0.11442535	-0.00431669	0.06863042	-0.19639960
**37**	0.02919557	0.11240698	-0.01656484	0.05344163	-0.15720777
**38**	0.03066602	0.10930704	-0.02456180	0.04654096	-0.18244325
**39**	0.03233567	0.10758694	-0.03116565	0.04397288	-0.14610609
**40**	0.03401924	0.10569322	-0.03746346	0.04756528	-0.13363220
**41**	0.03569414	0.10293358	-0.04377414	0.04205548	-0.10174189
**42**	0.03730574	0.10128047	-0.05060143	0.03655589	-0.10587520
**43**	0.03899716	0.09883119	-0.05779410	0.03206390	-0.08804009
**44**	0.04055110	0.09660254	-0.06655521	0.02538073	-0.07180796

**45**	0.04219906	0.09442028	-0.07122738	0.02492251	-0.05200352
**46**	0.04379206	0.09178199	-0.07181208	0.01263479	-0.06249489
**47**	0.04543934	0.08979478	-0.07384974	0.02758824	-0.05139017
**48**	0.04698352	0.08668127	-0.07772322	0.02782145	-0.04349166
**49**	0.04853960	0.08513582	-0.08127546	0.02044016	-0.03115121
**50**	0.05003393	0.08327113	-0.08148394	0.02622979	-0.00683520
**51**	0.05153932	0.08060560	-0.08134396	0.02725053	-0.01525520
**52**	0.05304808	0.07907516	-0.08343215	0.01962023	0.00501229
**53**	0.05446109	0.07711835	-0.08740963	0.01286311	0.02080814
**54**	0.05571967	0.07506104	-0.09279461	0.00681762	0.00963027
**55**	0.05717695	0.07331641	-0.09475024	0.00671597	-0.00244279
**56**	0.05853724	0.07176890	-0.09179474	0.01439450	0.00390138
**57**	0.05988478	0.06875841	-0.09435483	0.00302102	0.03722988
**58**	0.06113785	0.06765079	-0.10298653	-0.00312101	0.01448660
**59**	0.06255997	0.06626616	-0.10051881	-0.00359575	0.02484566
**60**	0.06377821	0.06352307	-0.09796519	0.00250302	0.04101821
**61**	0.06517310	0.06191267	-0.09583338	0.00502188	0.00862335
**62**	0.06632642	0.06061973	-0.10056038	-0.01477188	0.02623670
**63**	0.06762094	0.05863298	-0.10239897	-0.01383765	0.02196789
**64**	0.06895076	0.05681481	-0.10425987	-0.02058124	0.04169255
**65**	0.07006962	0.05460173	-0.09859830	-0.00750289	0.02119182
**66**	0.07144832	0.05257453	-0.09708031	-0.01125366	0.02521537
**67**	0.07271714	0.05159877	-0.10281104	-0.02463797	0.02825283
**68**	0.07386308	0.04914403	-0.10521025	-0.02673828	0.05440787
**69**	0.07518878	0.04762680	-0.10169523	-0.03403148	0.02191899
**70**	0.07666206	0.04579048	-0.10241461	-0.02665575	0.03578543
**71**	0.07778327	0.04438728	-0.11112768	-0.04436228	0.02442663
**72**	0.07912060	0.04304845	-0.12101717	-0.05996394	0.03874363
**73**	0.08028777	0.04226691	-0.13094158	-0.07936105	0.04884266
**74**	0.08156886	0.03979766	-0.12547439	-0.07297828	0.02038911
**75**	0.08293981	0.03868778	-0.12806992	-0.08194277	0.04135131
**76**	0.08418153	0.03661185	-0.13205916	-0.08162751	0.02318718
**77**	0.08548473	0.03392819	-0.12015610	-0.06850369	0.03495370
**78**	0.08688773	0.03183917	-0.11473775	-0.07474101	0.02741509
**79**	0.08807351	0.03025956	-0.11945372	-0.08282719	0.02130290
**80**	0.08962953	0.02853465	-0.10773532	-0.06943874	0.03976094
**81**	0.09132763	0.02448872	-0.09347224	-0.04464906	0.02804354
**82**	0.09265664	0.02253472	-0.08429441	-0.04692058	0.03884718
**83**	0.09410341	0.01999116	-0.07439220	-0.03080313	0.02813712
**84**	0.09550239	0.01773341	-0.06969327	-0.02587940	0.00766815
**85**	0.09683796	0.01602363	-0.07063641	-0.04015554	0.02299452
**86**	0.09848646	0.01315112	-0.06793643	-0.04072186	0.04960649
**87**	0.10043449	0.01074763	-0.06693328	-0.04459991	0.02965487
**88**	0.10194924	0.00919621	-0.07195120	-0.04546803	0.05045727
**89**	0.10329448	0.00719250	-0.07310076	-0.05917850	0.02032940
**90**	0.10492743	0.00435102	-0.06346622	-0.04305857	0.00681883
**91**	0.10645340	0.00188717	-0.04953575	-0.03435506	0.01881473

**92**	0.10779548	-0.00062358	-0.03953343	-0.02466629	0.00496252
**93**	0.10938899	-0.00297399	-0.02810353	-0.01473382	0.02410704
**94**	0.11089520	-0.00607470	-0.02038075	-0.00305259	0.01195299
**95**	0.11251205	-0.00882967	-0.01111309	0.00870996	0.01985776
**96**	0.11412352	-0.01084286	-0.00693176	0.00802250	0.01450084
**97**	0.11576188	-0.01351330	-0.00426638	0.00579711	0.00071492
**98**	0.11737554	-0.01562150	-0.00193877	0.00125578	0.00652687
**99**	0.11901943	-0.01819244	0.00846358	0.01531943	0.00339873
**100**	0.12054217	-0.02075953	0.01229424	0.01549656	-0.00326425
**101**	0.12233310	-0.02292229	0.00778904	0.00132806	0.00062297
**102**	0.12396068	-0.02457694	0.00261077	-0.01058419	0.01751100
**103**	0.12574145	-0.02629822	-0.01049088	-0.02902140	-0.00184618
**104**	0.12746742	-0.02894951	-0.01181371	-0.04536741	0.02060476
**105**	0.12921991	-0.03151025	-0.00842584	-0.03848117	0.03614411
**106**	0.13106922	-0.03447003	0.00459629	-0.02806377	0.01474467
**107**	0.13291571	-0.03738713	0.01233642	-0.02827234	0.02507973
**108**	0.13465227	-0.04012034	0.01950098	-0.02311585	0.01620264
**109**	0.13653013	-0.04346110	0.03097529	-0.01170987	-0.00549233
**110**	0.13842602	-0.04573184	0.03725228	-0.00673562	0.00748912
**111**	0.14026166	-0.04959419	0.05328229	0.00985287	-0.03561476
**112**	0.14221134	-0.05128353	0.04821519	-0.00230907	-0.00160597
**113**	0.14414577	-0.05375533	0.03842348	-0.01778896	0.00686425
**114**	0.14618433	-0.05703409	0.05288982	-0.00739313	0.01841775
**115**	0.14814173	-0.06112245	0.07836994	0.01546586	-0.02080592
**116**	0.15027657	-0.06404654	0.08587865	0.02717433	-0.01567293
**117**	0.15226172	-0.06697830	0.08986553	0.02754411	-0.01084505
**118**	0.15434510	-0.06943673	0.09163240	0.02201971	-0.02769764
**119**	0.15628743	-0.07301133	0.10376888	0.02595099	-0.01129211
**120**	0.15848865	-0.07628282	0.10697385	0.03331912	-0.02555379
**121**	0.16058037	-0.07973307	0.11835248	0.03741372	-0.01671537
**122**	0.16273549	-0.08345647	0.13726776	0.06288207	-0.03860940
**123**	0.16494066	-0.08701680	0.15165244	0.07446043	-0.02467444
**124**	0.16700842	-0.09036422	0.16192600	0.08210897	-0.03886419
**125**	0.16939213	-0.09349440	0.16771231	0.08370612	-0.03041763
**126**	0.17166990	-0.09675135	0.17280407	0.09138677	-0.04522346
**127**	0.17400181	-0.09993996	0.17114475	0.07402224	-0.03086265

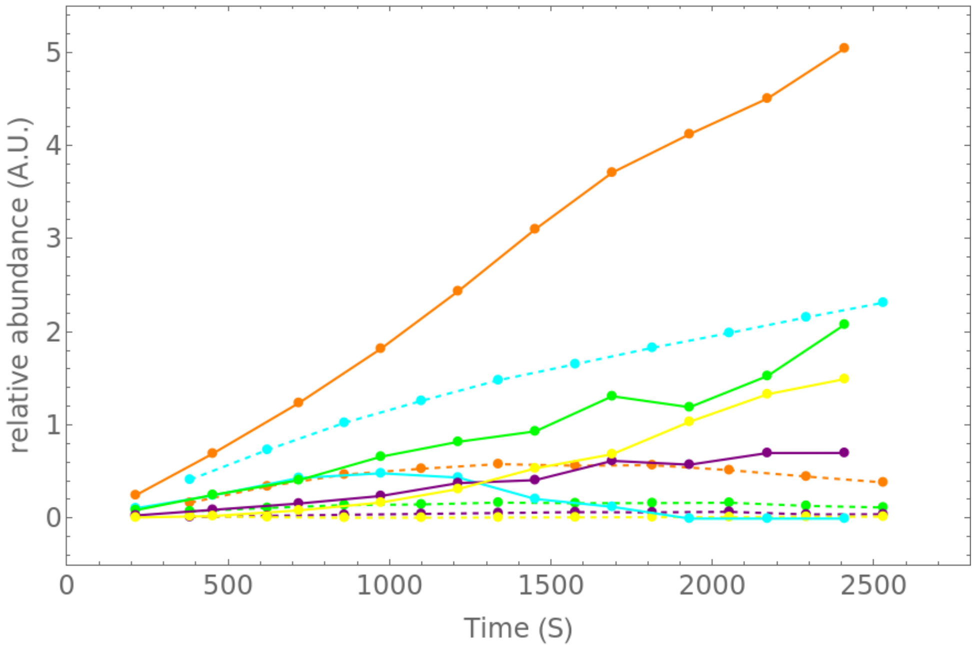

Figure S25. The component kinetic history over the initial period (from light on till about 40 minutes later) of 7-Cl spin trapping experiments. Dashed plots are from the experiment in toluene, solid plots are from the experiment in n-heptane. The color markers are cyan for **Cl-PBN**, yellow for **oxo-PBN**, orange for **7-PBN**, green for **8/9-PBN**, and purple for **10-PBN**. Here we can notice that Cl-PBN is better preserved in toluene than in n-heptane, while components are stabler in n-heptane.

EPR measurements **11-Cl**

Two different stock solutions were prepared. Into the first flask *N*-chloro-*N*-(heptan-2-yl)-4-methylbenzenesulfonamide. (35 mg, 0.012 mmol) and toluene or heptane (0.3 mL) were added. Into the second flask PBN (8 mg 0.0045 mmol) and toluene or heptane (0.3 mL) were added. Inside a glovebox, small volumes from both solutions were transferred to an EPR tube which was then used for the measurement. The deconvolution was performed as a local least square fit using a template inspired by 7-Br.

The rest of the sample loading, measuring, and analyzing procedures are essentially the same as 7-Cl.

Cl-PBN

Simulated values: g_{exp}= 2.0076, $\alpha_{N,exp}$ = 12.28 G, $\alpha_{Cl,exp}$ = 6.26 G, and $\alpha_{H,exp}$ = 0.82 G.

11-PBN

Simulated values: g_{exp}= 2.0063, $\alpha_{N,exp}$ = 14.14 G, $\alpha_{H,exp}$ = 3.87 G, and $\alpha_{N\prime,exp}$ = 1.53 G

12-PBN and **13-PBN**

Simulated values: g_{exp}= 2.0063, $\alpha_{N,exp}$ = 13.80 G, $\alpha_{H,exp}$ = 2.61 G,

14-PBN

Simulated values: g_{exp}= 2.0063, $\alpha_{N,exp}$ = 14.06 G, $\alpha_{H,exp}$ = 7.29 G,

oxo-PBN

Simulated values: g_{exp}= 2.0070, $\alpha_{N,exp}$ = 7.94 G

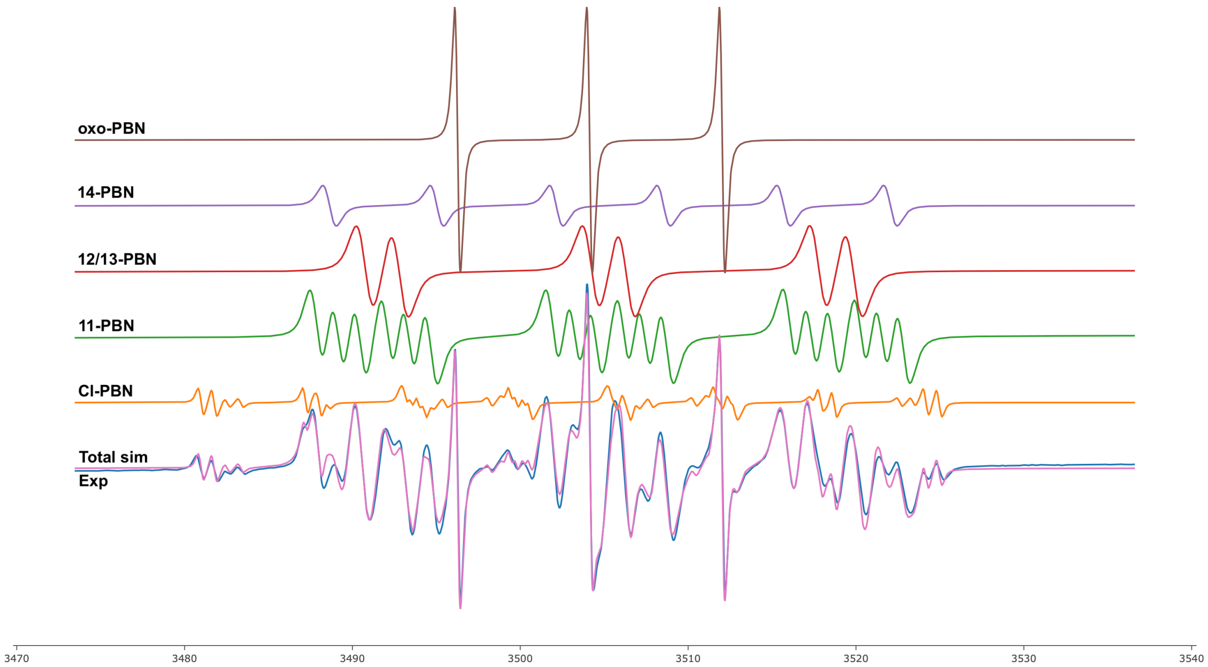

Figure S26. Experimental EPR spectra of **11-Cl** in *n*-heptane.

EPR measurements **11-Br**

Two different stock solutions were prepared. Into the first flask *N*-bromo-*N*-(heptan-2-yl)-4-methylbenzenesulfonamide **11-Br** (45 mg, 0.013 mmol) and toluene or heptane (0.3 mL) were added. Into the second flask PBN (8 mg, 0.0045 mmol) and toluene or heptane (0.3 mL) were added. Inside a glovebox, small volumes from both solutions were transferred to an EPR tube which was then used for the measurement.

The rest of the sample loading, measuring, and analyzing procedures are essentially the same as 7-Cl, except that the spectra were recorded without light illumination. Reaction has already started (and finished) during the mixture preparation in glovebox and/or during the transfer of EPR tube from glovebox to the EPR cavity. The deconvolution followed the visual recognition of one C-centered radical abduct and the oxo-PBN signal, together with the proposed 11-PBN.

11-PBN

Simulated values: g_{exp}= 2.0064, $\alpha_{N,exp}$ = 14.20 G, $\alpha_{H,exp}$ = 4.45 G, and $\alpha_{N\prime,exp}$ = 1.21 G

12-PBN and/or **13-PBN**

Simulated values: g_{exp}= 2.0065, $\alpha_{N,exp}$ = 13.69 G, $\alpha_{H,exp}$ = 1.89 G,

10-PBN

Not discerned in the spectra

oxo-PBN

Simulated values: g_{exp}= 2.0071, $\alpha_{N,exp}$ = 8.00 G

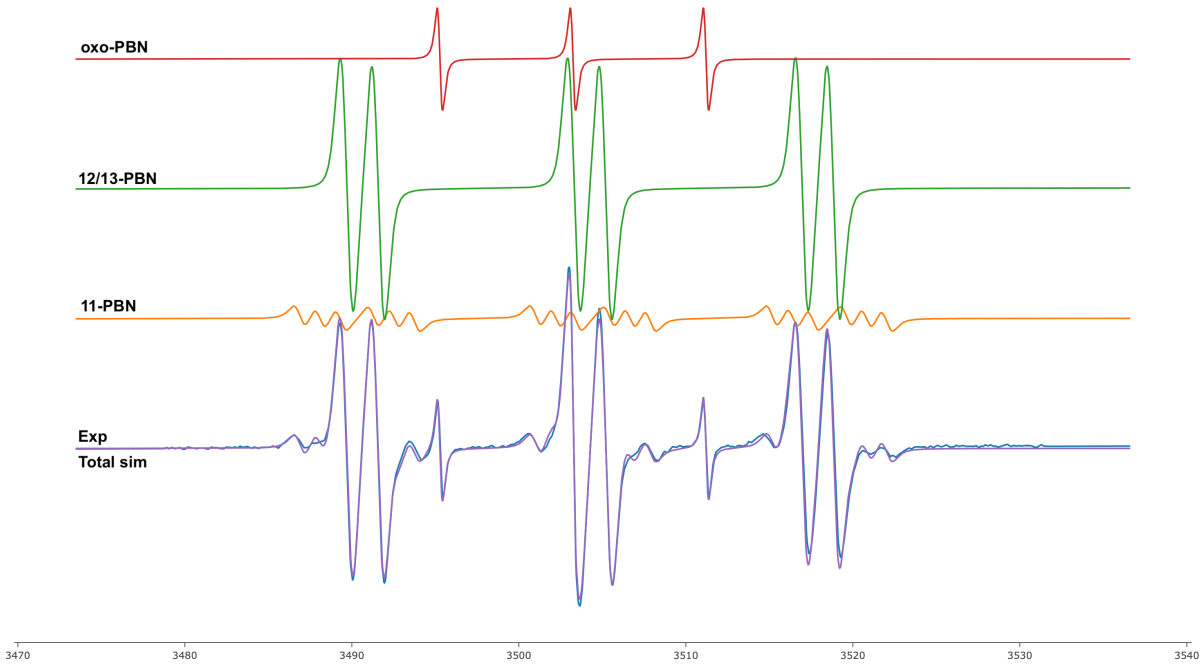

Figure S27. Experimental EPR spectra of **11-Br** in toluene.

Strong triplet-doublet is from *C*-centered radical adducts to the PBN, namely a mixture of **12-PBN** and **13-PBN**. Also observed is the **11-PBN** and **oxo-PBN**.

NMR spectra of **7-I** reaction mixture under irradiation

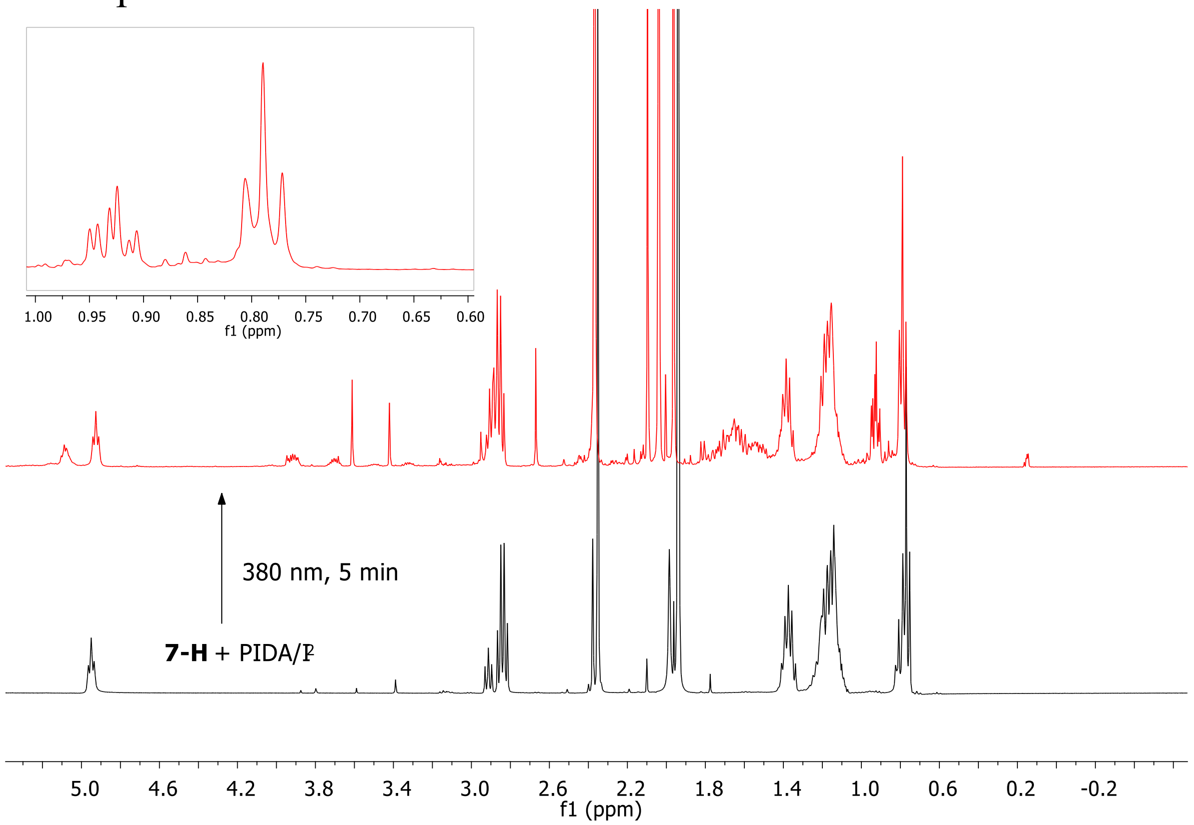

Figure S28. ^{1}H NMR spectra of the **7-H**/PIDA/I_2 mixture (black line) and the resulting reaction mixture after *off-site* irradiation (red line).

In addition to the methyl group signal (0.80 ppm) of the reactant, two methyl triplets appeared in the upfield region (see the inserted inbox). New multiplets appeared, after irradiation, at 1.6 ppm and 5.1 ppm, supporting the formation of two products, namely C_2- and C_5-halogenated structures, as expected (see text).

NMR spectra of **7-Cl** reaction mixture under irradiation

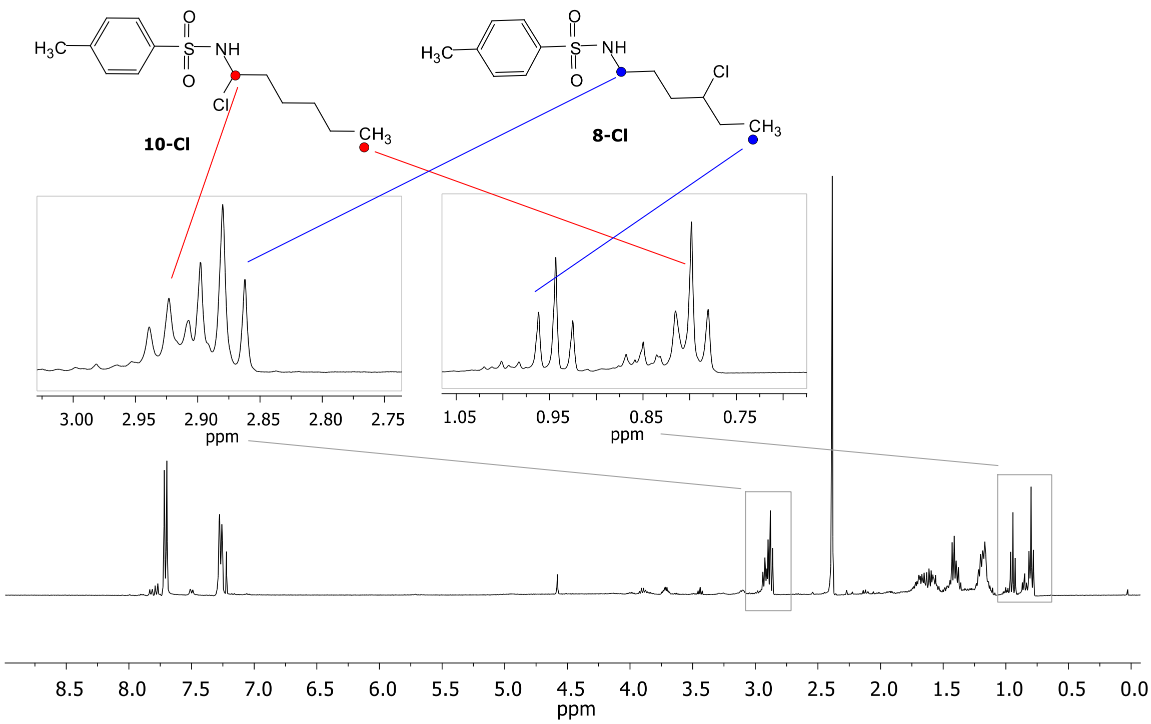

Figure S29. ^{1}H NMR spectra of **7-Cl** reaction mixture under irradiation

After *off-site* irradiation of **7-Cl** the ^{1}H NMR spectra of the corresponding reaction mixture was taken. In the upfield region two triplets were observed, at 0.80 and 0.95 ppm, which correspond to methyl groups in **10-Cl** and **8-Cl**, respectively. Two additional triplets were observed, at 2.88 and 2.93 ppm, which corresponds to C2-H protons in **10-Cl** and **8-Cl**, respectively. All signals observed for **8-Cl** are fully consistent with earlier report.[29] Signals corresponding to the tosyl group of **10-Cl** and **8-Cl** overlap each other.

NMR spectra of **11-Cl** reaction mixture under irradiation

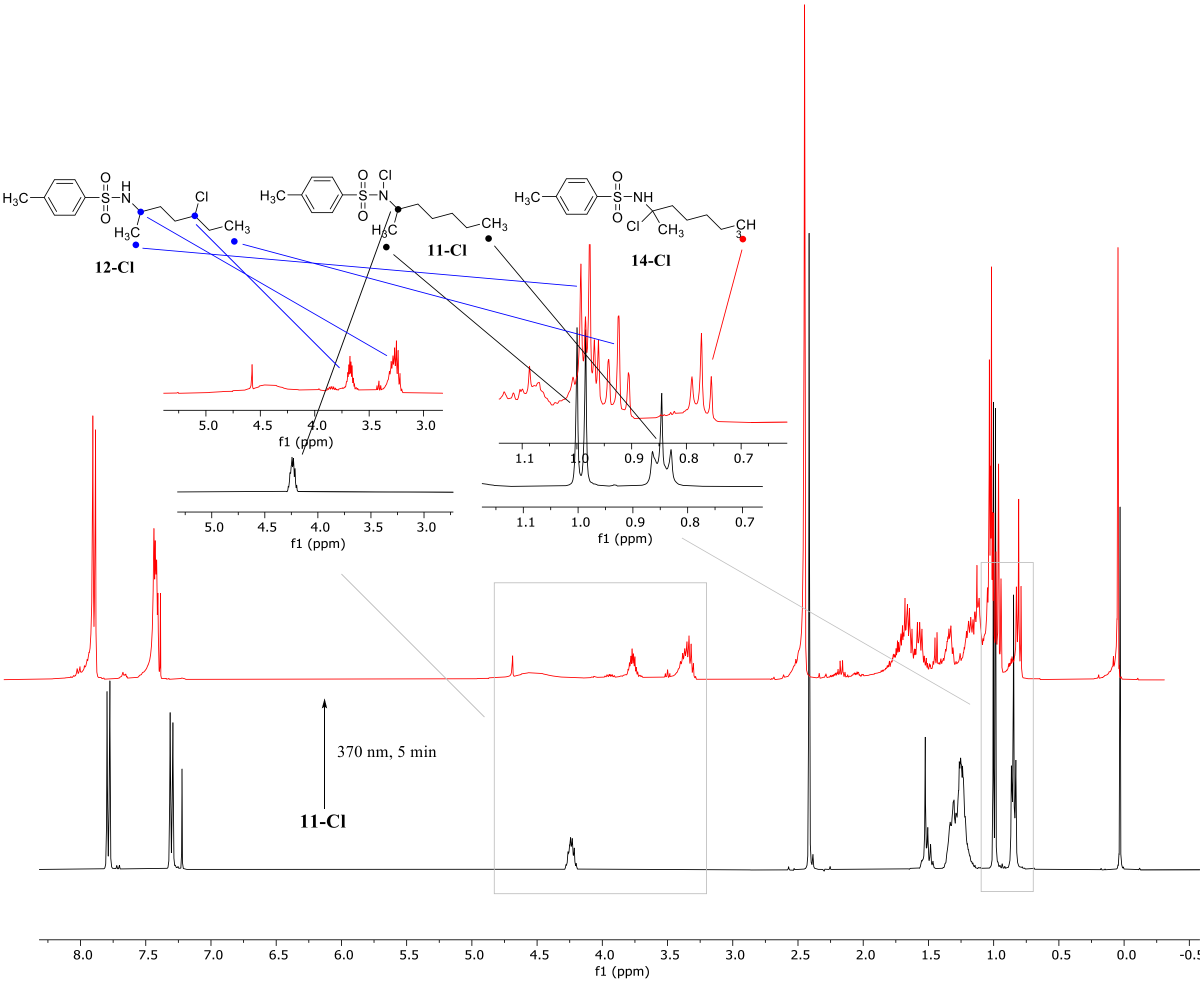

Figure S30. ^{1}H NMR spectra of **7-Cl** reaction mixture under irradiation

Before *off-site* irradiation, ^{1}H NMR spectra of **11-Cl** was taken. All signals observed for **11-Cl** are fully compatible with previous reports.[30]

After *off-site* irradiation of **11-Cl** the ^{1}H NMR spectra of the corresponding reaction mixture was taken. In the upfield region two triplets were observed, at 0.81 and 0.96 ppm, which correspond to methyl groups in **14-Cl** and **12-Cl**, respectively, while the triplet of **11-Cl** at 0.85 ppm is lost. Additionally, there is a doublet corresponding to a methyl group at C2 position of **12-Cl**. Furthermore, two multiplets were observed, at 3.30 and 3.71 ppm, which correspond to C2-H proton and C5-H proton in **12-Cl**. Multiplet at 4.24 ppm which corresponds to C2-H of **11-Cl** is lost**.** All signals observed for **12-Cl** are fully consistent with earlier reports.[31] Signals corresponding to the tosyl group of **12-Cl** and **14-Cl** overlap each and are upfield when compared to analogous signal of **11-Cl**.

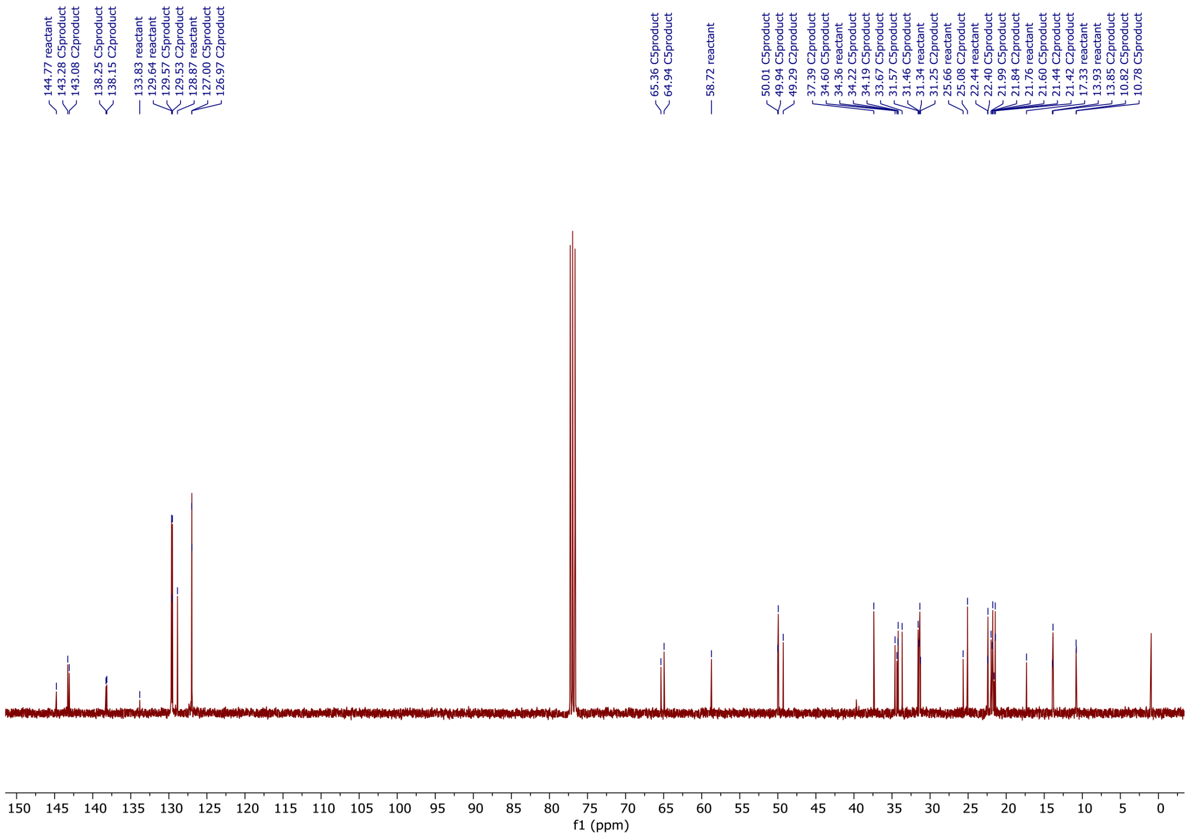

Figure S31. ^{13}C NMR spectra of **7-Cl** reaction mixture under irradiation

After *off-site* irradiation of **11-Cl** the ^{13}C spectra of the coresponding reaction mixture was also taken. Spectra shows only three products in the downfield region (in the aromatic region there are only two products, **14-Cl** and **12-Cl**), two of which are assigned to compound **12-Cl** (diastereomeric mixture), which is in accordance with the literature,[31]and the third to **14-Cl.**

NMR spectra of **11-Br** reaction mixture under irradiation

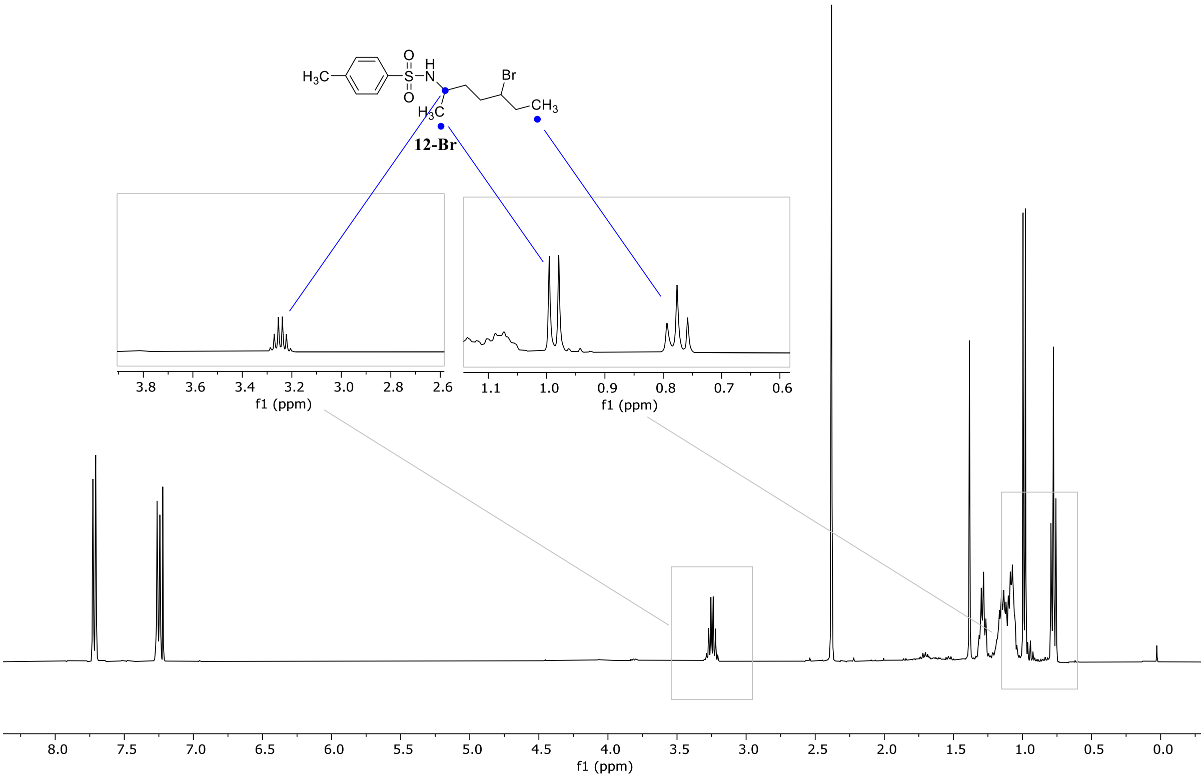

Figure S32. ^{1}H NMR spectra of **11-Br** reaction mixture under irradiation

After *off-site* irradiation of **11-Br** the ^{1}H NMR spectra of the corresponding reaction mixture was taken. In the upfield region one triplet and one doublet were observed, at 0.77 and 0.98 ppm, respectively, which correspond to two methyl groups in **12-Br**. Additionally there is a sextet corresponding to C2-H proton in **12-Br**.

DFT results and optimized geometries

Showing only the top 10 structures obtained after ensemble sorting using CREST. For all calculated structures and energies, please consult Zenodo repository.[32] Energies in the title of .xyz correspond to enthalpies calculated at RO-B2PLYP-D3/G3MP2-large//B3LYP/6-31G(d), using enthalpies and single point energies of B3LYP/6-31G(d) calculations, and single point energies at RO-B2PLYP-D3/G3MP2-large level of theory.

Calculation of reaction enthalpy for system 7’/8’

$$\begin{aligned}\Delta \text{BDE}(7'/8') &= \text{BDE}_{\textit{C-H/propane}} - \text{BDE}_{\textit{N-H/tosylamide}}\\ &= (\text{BDE}_{\textit{C-H/methane,exp}} + \text{RSE}_{\textit{C-H/propane,calc}})\\ &\qquad - (\text{BDE}_{\textit{N-H/ammonia,exp}} + \text{RSE}_{\textit{N-H/tosylamide,calc}})\\ &= \Delta H_{\text{predict,298}}\\ &= -19.8 \text{ kJ/mol}\end{aligned}$$

Calculation of reaction enthalpy for system 8’/10’

$$\begin{aligned}\Delta \text{BDE}(8'/10') &= (\text{BDE}_{\textit{C-H/methane,exp}} + \text{RSE}_{\textit{C-H/N-ethyltosylamide,calc}})\\ &\qquad - (\text{BDE}_{\textit{C-H/methane,exp}} + \text{RSE}_{\textit{C-H/propane,calc}})\\ &= \Delta \text{RSE}\\ &= \Delta H_{\text{predict,298}}\\ &= -15.0 \text{ kJ/mol}\end{aligned}$$

N-H-C angle in 1,*n*-HAT reactions

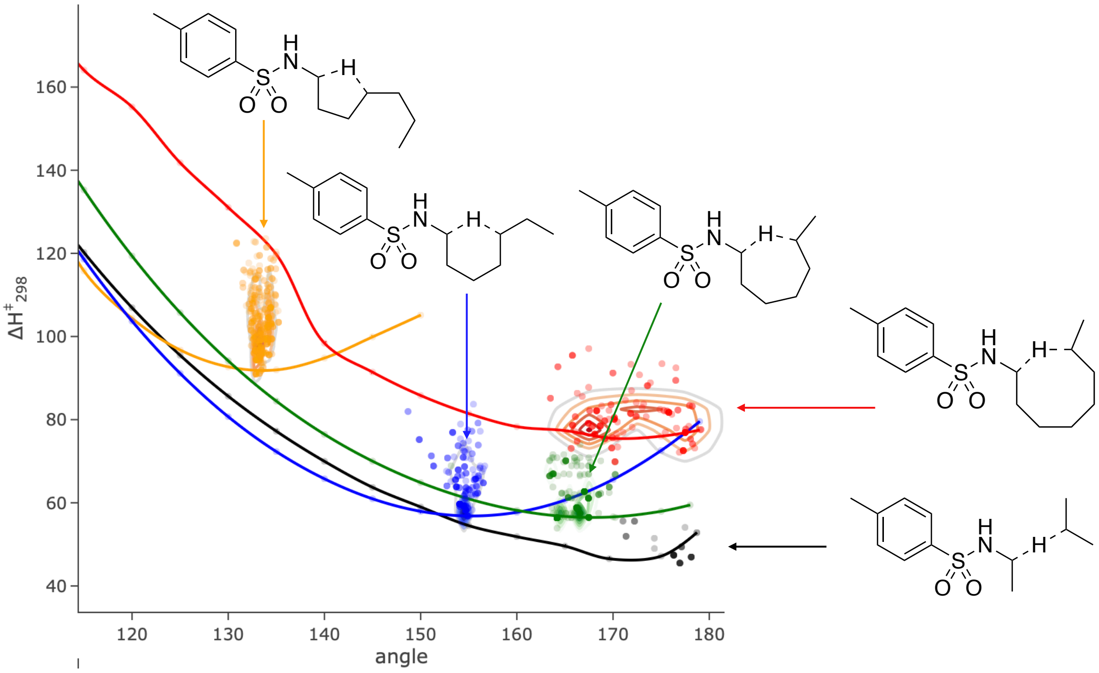

Chart 1. Energy (in kJ/mol) vs. N-H-C angle in transition states for HAT reaction. Constrained (lines) and unconstrained (dots) geometry optimizations and frequency calculation at B3LYP/6-31G(d) level of theory. The orange dots and line are for 1,4-HAT, with blue dots and line for 1,5-HAT, green dots and line for 1,6-HAT, red dots and line for 1,7-HAT, while black dots and line denote intermolecular reaction.

7-H

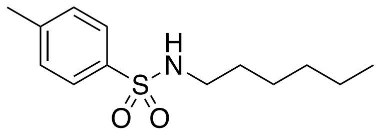

Name	E(B3LYP)	H(B3LYP)	E(RO-B2PLYP-D3)	H(RO-B2PLYP-D3)
Tosyl_NH_hexane_0013	-1111.367419	-1111.020571	-1110.944543	-1110.597695
Tosyl_NH_hexane_0006	-1111.368667	-1111.021649	-1110.944581	-1110.597563
Tosyl_NH_hexane_0004	-1111.36819	-1111.021289	-1110.944123	-1110.597222
Tosyl_NH_hexane_0054	-1111.367553	-1111.020462	-1110.943934	-1110.596843
Tosyl_NH_hexane_0050	-1111.369606	-1111.022753	-1110.94351	-1110.596657
Tosyl_NH_hexane_0042	-1111.367763	-1111.020679	-1110.943108	-1110.596024
Tosyl_NH_hexane_0039	-1111.368296	-1111.021365	-1110.942661	-1110.59573
Tosyl_NH_hexane_0021	-1111.367416	-1111.020487	-1110.942651	-1110.595722
Tosyl_NH_hexane_0034	-1111.368271	-1111.021321	-1110.94263	-1110.59568

38

C 2.5505724597 -1.3574629891 0.9157130193
C 2.9064986839 0.0248498957 0.3543766969
C 4.4182015736 0.2558441600 0.2363836533
C 4.7826798874 1.6392407159 -0.3177440056
C 1.0474554431 -1.6222728757 1.0465025361
N 0.4077900083 -1.6217609013 -0.2826748450
H 2.4685660538 0.7978709724 1.0030777415
H 2.4348691152 0.1504597819 -0.6284068250
H 4.8561285429 -0.5192012210 -0.4105612383
H 4.8873137026 0.1261765214 1.2233870980
H 4.3141710050 1.7681376162 -1.3037619951
H 0.5794543071 -0.8230983805 1.6329070657
C 6.2935025469 1.8638780548 -0.4363597557
H 6.7855071925 1.7755695349 0.5401701856
H 6.5202371875 2.8595736470 -0.8342326356
H 3.0018352536 -2.1417861203 0.2888677421
H 2.9954524553 -1.4790665948 1.9128344568
S -1.2755020506 -1.8184223714 -0.3401786998
O -1.7563579363 -2.5882254938 0.8152298955
O -1.5449502528 -2.2680914282 -1.7104929702
C -1.8594329012 -0.1364363777 -0.1414659314
C -1.6556043266 0.7876356289 -1.1708044151
C -2.5372159830 0.2226427710 1.0213402359
C -2.1309580263 2.0849981088 -1.0186112147
H -1.1369977224 0.4875492436 -2.0753804136
C -3.0074757241 1.5303654207 1.1566040398
H -2.6971563247 -0.5164023828 1.7989625404
C -2.8137916132 2.4778348835 0.1452941328
H -1.9744463992 2.8075355574 -1.8161378613
H -3.5364982314 1.8149015825 2.0626810044
C -3.3387918824 3.8868628006 0.2845890495
H -2.5669916816 4.6263733709 0.0415295220
H -4.1798391296 4.0635743853 -0.3980257522
H -3.6899737809 4.0856674620 1.3015841508
H 6.7529265601 1.1255914679 -1.1051651844
H 4.3466907587 2.4139837204 0.3289575944
H 0.8666778610 -2.5655483007 1.5783131077
H 0.8014809314 -2.3160457540 -0.9184706190
38

C 2.7825180247 -1.9910751917 -0.3787792212
C 3.2122129780 -0.8436342697 0.5498202725
C 3.0935796656 0.5570079162 -0.0655652725
C 3.6375953880 1.6629067157 0.8486883562
C 1.3178242632 -1.9644487468 -0.8311427719
N 0.4177328773 -2.0065880244 0.3357435589
H 4.2564820432 -1.0178041203 0.8447917978
H 2.6197129924 -0.8825074512 1.4727304790
H 2.0416393572 0.7753117717 -0.2948378034
H 3.6314867550 0.5838263588 -1.0257373533
H 3.1128986697 1.6227020988 1.8138113131
H 1.1200202149 -2.7932762077 -1.5234796395
C 3.4953392256 3.0654910158 0.2491193409
H 3.8932571642 3.8320730863 0.9239124524
H 2.4431335462 3.3063535500 0.0531989332
H 3.4035758522 -1.9878992557 -1.2851472383
H 2.9810458206 -2.9518616421 0.1184171164
S -1.2473793639 -1.8882745540 0.0421064305
O -1.8732402745 -2.4061606835 1.2636765339
O -1.5953178365 -2.4406585879 -1.2742211633
C -1.4822187831 -0.1137590381 -0.0457463492
C -1.8823735181 0.4663844221 -1.2475267468
C -1.2944603238 0.6633817054 1.1014517867
C -2.0872318130 1.8463949831 -1.3003763819
H -2.0331727939 -0.1593681521 -2.1205250939
C -1.5018241812 2.0363874692 1.0296936193
H -0.9930015392 0.1943484882 2.0322614434
C -1.9022604459 2.6499785696 -0.1696632436
H -2.3980025676 2.3030008623 -2.2367246990
H -1.3554624400 2.6452879985 1.9187085678
C -2.1467940886 4.1391939419 -0.2228699815
H -2.2182469906 4.4976697817 -1.2542085209
H -3.0838197933 4.4023170698 0.2847841279
H -1.3441477474 4.6945368448 0.2754450670
H 4.0352670146 3.1470865327 -0.7023877452
H 4.6958354376 1.4634816629 1.0697492849
H 1.1037433911 -1.0365334097 -1.3713313492
H 0.5529151412 -2.8297085463 0.9234130439
38

C 2.5756668449 -1.4509318876 1.0826696409
C 2.9919823155 -0.0565893621 0.5970783283
C 4.5149253923 0.1336333057 0.5736942966
C 4.9769964207 1.5411031594 0.1607207188
C 1.0624477650 -1.6857746772 1.1199178818
N 0.4987997506 -1.6233207924 -0.2419157905
H 2.5415365419 0.7008695787 1.2560693678
H 2.5696942456 0.1159921979 -0.3994146943
H 4.9631229194 -0.6066363880 -0.1063435287
H 4.9163752918 -0.0907522709 1.5725300000
H 4.5325825982 2.2808387306 0.8420764768
H 0.5787874920 -0.8977071277 1.7086603331

C 4.6463341026 1.9184239494 -1.2883071451
H 3.5658270475 1.9454440266 -1.4682340249
H 5.0817860140 1.1979991189 -1.9922978367
H 3.0444969360 -2.2207840438 0.4506973652
H 2.9618571883 -1.6215960843 2.0969562283
S -1.1816723439 -1.7874459453 -0.4005622028
O -1.7389063666 -2.5876371595 0.6984784547
O -1.3803889290 -2.1846637647 -1.7988780876
C -1.7498575405 -0.1033419201 -0.1761946011
C -1.4845385390 0.8484929604 -1.1654163640
C -2.4757795282 0.2307611171 0.9648096106
C -1.9473671566 2.1480904038 -0.9946459905
H -0.9294130459 0.5676927164 -2.0543872286
C -2.9323915119 1.5411753478 1.1193213822
H -2.6832842419 -0.5292694323 1.7103048502
C -2.6778506285 2.5159982815 0.1482757006
H -1.7435295658 2.8920725438 -1.7611689512
H -3.4989774499 1.8062360799 2.0084375999
C -3.1884725719 3.9282348982 0.3071450906
H -3.5819388626 4.1020195197 1.3131832644
H -2.3962154057 4.6626156824 0.1210846297
H -3.9956187364 4.1382061170 -0.4062936890
H 5.0454279158 2.9082814319 -1.5376138467
H 6.0633930026 1.6090620285 0.3067429622
H 0.8326085892 -2.6431743643 1.6055084655
H 0.9162345843 -2.3008143607 -0.8807078249
38

C 2.6746903846 -1.4569190136 -0.7251050313
C 3.0258140114 -0.1526766299 0.0022026061
C 4.5280641710 -0.0174906123 0.2871315374
C 4.9194721427 1.2484085026 1.0676674067
C 1.1843211311 -1.6319547067 -1.0325663051
N 0.4080365100 -1.7210170692 0.2185908154
H 2.4659165030 -0.0997859928 0.9448171227
H 2.6796305113 0.6936366439 -0.6067538018
H 5.0838295069 -0.0423862869 -0.6625461714
H 4.8603080753 -0.8982602009 0.8554378759
H 4.3672721236 1.2707969964 2.0181045538
H 1.0224101894 -2.5120284138 -1.6686080573
C 4.6842979202 2.5605691544 0.3097544108
H 5.0323049038 3.4198823648 0.8943183619
H 3.6230685517 2.7222027787 0.0896677217
H 3.2166262103 -1.5068377049 -1.6795129266
H 3.0255358215 -2.3184290669 -0.1364391962
S -1.2784381097 -1.8383221608 0.0852611968
O -1.7092036351 -2.4002644249 1.3702612719
O -1.6642010342 -2.4715648004 -1.1831789297
C -1.7712378951 -0.1182744193 -0.0052919319
C -2.3018308915 0.3830430079 -1.1918385942
C -1.6460693889 0.6925283220 1.1267964858
C -2.7023622494 1.7194718487 -1.2450519420
H -2.4038235322 -0.2698790989 -2.0519346169
C -2.0504074678 2.0206812218 1.0553074623
H -1.2424986336 0.2824541559 2.0467055877
C -2.5847917534 2.5557449095 -0.1292086671
H -3.1161266949 2.1149156909 -2.1693145351
H -1.9544112601 2.6555837377 1.9329531301
C -3.0388858596 3.9949907544 -0.1816302795
H -3.2267807540 4.3194918811 -1.2095603105
H -3.9680667340 4.1356771846 0.3855294995
H -2.2908230142 4.6663299212 0.2553237554
H 5.2259646290 2.5670198702 -0.6446538731
H 5.9821793659 1.1788233323 1.3359829519
H 0.8140667100 -0.7596572896 -1.5835698696
H 0.7041106420 -2.4892048907 0.8214891462
38

C 1.9252487988 -1.2560893458 0.9141239454
C 2.6368408347 -0.3747849565 -0.1186761868
C 3.2466009606 0.8943653626 0.4897283037
C 3.9675358346 1.7786017215 -0.5358645929
C 1.2980726077 -2.5268382581 0.3326465655
N 0.2435329680 -2.2466240059 -0.6676568330
H 1.9254825453 -0.0990447643 -0.9074103369
H 3.4294289891 -0.9603888759 -0.6092641769
H 3.9521591931 0.6170554511 1.2875022357
H 2.4526282262 1.4796279488 0.9770492673
H 4.7614335559 1.1946303500 -1.0230668193
H 0.9076038437 -3.1588524339 1.1391909498
C 4.5704467886 3.0468313341 0.0768745753
H 5.0774435677 3.6561566542 -0.6800963361
H 5.3054474620 2.8012565018 0.8533812607
H 2.6374860250 -1.5680201127 1.6911306288
H 1.1512155953 -0.6737209414 1.4308288096
S -1.3442029001 -1.9833693396 -0.1294985257
O -1.5064333418 -2.4748458191 1.2450826681
O -2.1857900475 -2.4824818337 -1.2240666383
C -1.4882491205 -0.1970310502 -0.0751291452
C -1.7251185061 0.4344794893 1.1433657768
C -1.4100327688 0.5372804899 -1.2623786268
C -1.8711157899 1.8229660087 1.1718474166
H -1.7965107981 -0.1575397322 2.0493719805
C -1.5560763942 1.9190136949 -1.2147395527
H -1.2411002706 0.0300600963 -2.2066684165
C -1.7885845466 2.5846313040 0.0010235639
H -2.0557724239 2.3191042347 2.1213225759
H -1.4934647052 2.4938922493 -2.1357386729
C -1.9576370724 4.0849481878 0.0307360244
H -1.1175294683 4.5907163628 -0.4598493667
H -2.8694478025 4.3904684892 -0.4977584827
H -2.0255271670 4.4607671006 1.0559853368
H 3.7958494932 3.6693857704 0.5417114876
H 3.2624690191 2.0555863058 -1.3325054928
H 2.0626659725 -3.1032548182 -0.2009563719
H 0.1724174415 -2.9562729375 -1.3936323443
38

C 2.3593724481 -2.2131741388 0.4942304574
C 3.1917131324 -0.9384904070 0.3069997275
C 4.6568053953 -1.2305987799 -0.0435044054
C 5.4991184860 0.0365319289 -0.2393457173
C 0.8991790366 -1.9893144130 0.9122662394
N 0.0637722175 -1.2970765388 -0.0856305397
H 3.1439182943 -0.3360958840 1.2242790531
H 2.7488031039 -0.3187034760 -0.4835090077
H 4.6997068232 -1.8384488369 -0.9602178170
H 5.1079890858 -1.8468200556 0.7490733737
H 5.0481191847 0.6509001678 -1.0309416525
H 0.8481657628 -1.4017404136 1.8323552610
C 6.9611287625 -0.2584468342 -0.5896049970
H 7.0379930377 -0.8341975257 -1.5204350972
H 7.4488946875 -0.8427623263 0.2006793085
H 2.3795264777 -2.8083210156 -0.4322256401
H 2.8235845192 -2.8465345832 1.2643270175
S -0.1434632505 0.3774183746 -0.0383612112
O 0.3701521648 0.9339337881 -1.2978687573
O 0.3386435398 0.8399885664 1.2680674953
C -1.9255012339 0.5825415766 -0.0648341985
C -2.5728948287 0.8300152303 -1.2744855793
C -2.6450737674 0.4964467891 1.1291480044
C -3.9596664634 0.9788797056 -1.2847117639
H -1.9947665542 0.9179594094 -2.1881120069
C -4.0281764148 0.6483434228 1.0998059308
H -2.1240520047 0.3262506311 2.0654571271
C -4.7078748411 0.8909234911 -0.1040202748
H -4.4670940318 1.1726904705 -2.2265762736
H -4.5910241508 0.5829713836 2.0279812058
C -6.2057124970 1.0834527851 -0.1162956120
H -6.4739628075 2.1044250141 0.1857746975
H -6.6235927048 0.9165867215 -1.1140044117
H -6.7032560917 0.3996455907 0.5800505136
H 7.5351810693 0.6657569053 -0.7221754383
H 5.4563085591 0.6433621791 0.6759811131
H 0.4210349144 -2.9561544754 1.1135986995
H 0.2292464401 -1.5851948692 -1.0470866262
38

C 2.4929971808 -1.8790645682 -0.8098547513
C 3.8389760149 -1.2679113725 -1.2369917074

C 4.1249299935 0.1511144027 -0.7116403148
C 4.1405177135 0.2787925932 0.8185019132
C 1.2738038348 -1.1153321917 -1.3408090887
N -0.0249366241 -1.6969465615 -0.9561609192
H 3.8984962560 -1.2590394654 -2.3351876970
H 4.6409777522 -1.9373264385 -0.8969164732
H 3.3945674223 0.8618700164 -1.1252147555
H 5.1003788625 0.4699621264 -1.1061076635
H 3.1511149243 0.0345737507 1.2252997246
H 1.2934213317 -1.0769158537 -2.4375902984
C 4.5487167228 1.6781728927 1.2914310659
H 3.8579435730 2.4418554053 0.9122406032
H 5.5546347206 1.9403505565 0.9402352834
H 2.4428648356 -2.9134422757 -1.1807994891
H 2.4239482102 -1.9338914262 0.2811918255
S -0.6786590387 -1.3102611083 0.5533550463
O 0.3667555159 -0.8807901147 1.4946365480
O -1.5619950719 -2.4381519602 0.8705677951
C -1.6717287503 0.1368392835 0.1866771162
C -1.3775380664 1.3457200884 0.8123428324
C -2.7595907280 0.0246541478 -0.6849742545
C -2.1788963668 2.4591459742 0.5507422952
H -0.5362964532 1.4056654553 1.4941207715
C -3.5462633133 1.1430075667 -0.9333696974
H -2.9813889771 -0.9256701794 -1.1593742674
C -3.2700401558 2.3776811480 -0.3207507593
H -1.9508891451 3.4046500293 1.0363387897
H -4.3919036991 1.0601915911 -1.6121210045
C -4.1424833261 3.5790689555 -0.5961435569
H -3.7512104334 4.4785540863 -0.1115768726
H -4.2162834099 3.7797829109 -1.6716840545
H -5.1638044792 3.4187648620 -0.2285895740
H 4.5487457787 1.7454513540 2.3852140148
H 4.8358710019 -0.4646754860 1.2347542740
H 1.2769437984 -0.0784978809 -0.9927499404
H -0.0793989683 -2.7090079129 -1.0598212153
38

C 1.7806071473 -0.8417433299 1.1988871554
C 2.7796636583 -0.3611907301 0.1383293932
C 3.4654952359 0.9612444537 0.5055662325
C 4.4615560511 1.4498371028 -0.5538227784
C 1.1311544441 -2.1978918241 0.8825733272
N 0.3278698358 -2.2581041845 -0.3428911472
H 2.2655951379 -0.2346755445 -0.8262437031
H 3.5448650443 -1.1363707385 -0.0196814626
H 3.9867994741 0.8440141583 1.4672087497
H 2.6993285007 1.7336936996 0.6676400852
H 5.2255054494 0.6763366727 -0.7166568354
H 0.4752313760 -2.5167824575 1.6970309834
C 5.1433269940 2.7692968390 -0.1777428248
H 5.7032237224 2.6729088816 0.7606282944
H 4.4073681246 3.5714158179 -0.0420369573
H 2.2991036385 -0.9426272108 2.1631704240
H 1.0005508068 -0.0836760629 1.3433924246
S -1.3192768736 -1.9809246321 -0.3777148888
O -1.8730319214 -2.6452795555 0.8044242470
O -1.7236342979 -2.3170310346 -1.7446192929
C -1.6197213537 -0.2180210405 -0.1691451001
C -1.5475577612 0.6290692909 -1.2775849276
C -1.9147197445 0.2903861597 1.0970304073
C -1.7565123636 1.9954925509 -1.1062007664
H -1.3523556380 0.2167800120 -2.2623871886
C -2.1215153794 1.6606602857 1.2504219153
H -1.9969151454 -0.3852024769 1.9418415343
C -2.0451386026 2.5338291678 0.1565889618
H -1.7032711342 2.6551093865 -1.9691128507
H -2.3544059102 2.0570129967 2.2358672159
C -2.3047006699 4.0119130645 0.3254226553
H -1.7224694015 4.6057119730 -0.3869687028
H -3.3637518089 4.2467158722 0.1550842932
H -2.0549722281 4.3504238736 1.3361614806
H 5.8472095523 3.0911890265 -0.9535212777
H 3.9396251440 1.5680688813 -1.5141826377
H 1.9080901265 -2.9678025235 0.7868573882
H 0.7772658272 -1.9992974622 -1.2148085786
38

C 2.2590590764 -2.5050459099 1.1548380529
C 3.0885303866 -1.9888761963 -0.0320383447
C 3.9725497996 -0.7744766325 0.2856610651
C 4.8478161855 -0.3484857229 -0.9005909719
C 1.1403049490 -1.5817689078 1.6622172077
N 0.0262807389 -1.3793991325 0.7156036347
H 2.4240589201 -1.7481422486 -0.8726060548
H 3.7276841800 -2.8128016632 -0.3798803390
H 4.6176023054 -1.0093658945 1.1467808418
H 3.3457703743 0.0754329677 0.5829596440
H 5.4768156197 -1.1949925838 -1.2126956590
H 1.5227954544 -0.5919994208 1.9199974768
C 5.7355305296 0.8606569412 -0.5892534337
H 6.3467228360 1.1421957020 -1.4546055227
H 6.4167676581 0.6494728166 0.2447894993
H 1.8111941312 -3.4750997651 0.8908347274
H 2.9250096849 -2.7068915272 2.0065272279
S -0.0155249208 -0.0395490790 -0.3093532412
O 0.1645029708 -0.4838598746 -1.6991216257
O 0.8593848687 0.9771070660 0.2878062203
C -1.7110736826 0.5250551355 -0.1584202557
C -2.6134550493 0.2762150773 -1.1900171942
C -2.1038718868 1.2235363661 0.9869265542
C -3.9298447317 0.7234495407 -1.0635470353
H -2.2828633289 -0.2490018026 -2.0795391698
C -3.4188089718 1.6620764532 1.0950826524
H -1.3846549986 1.4265345123 1.7736645457
C -4.3532337901 1.4191807375 0.0743711333
H -4.6363205385 0.5314301289 -1.8672255456
H -3.7275729436 2.2067687666 1.9842739056
C -5.7729484476 1.9178362577 0.2020456530
H -5.8145055315 3.0126185550 0.1367903403
H -6.4125475834 1.5141696706 -0.5886077181
H -6.2095117410 1.6367719401 1.1675253722
H 5.1308873087 1.7320809291 -0.3103434148
H 4.2002501739 -0.1156717583 -1.7569623189
H 0.7059088389 -2.0083707025 2.5750465882
H -0.2324709399 -2.2126050116 0.1916168325
38

C 2.8322277630 -1.8817880995 -0.5601813834
C 3.2717069792 -0.6357946920 0.2263461892
C 3.1222549037 0.6825278444 -0.5472081286
C 3.6904936420 1.9110360222 0.1834931136
C 1.3429555076 -1.9422144548 -0.9204988089
N 0.5191056820 -1.9375712469 0.3021868533
H 4.3264290927 -0.7636862070 0.5086854224
H 2.7025767926 -0.5869377038 1.1617351806
H 2.0620757906 0.8664746202 -0.7697754702
H 3.6288729616 0.5835548877 -1.5186194497
H 4.7555904896 1.7438840354 0.3995522949
H 1.1376438188 -2.8240619110 -1.5414145726
C 2.9561658583 2.2707606617 1.4809493694
H 3.3568765723 3.1932609587 1.9167680272
H 3.0515595710 1.4828921176 2.2364484621
H 3.3995550884 -1.9486510492 -1.4988381839
H 3.0920479078 -2.7835575655 0.0134740616
S -1.1636641942 -1.8679190322 0.1140106689
O -1.6962775357 -2.3616640936 1.3887222585
O -1.5784879689 -2.4683075102 -1.1610517158
C -1.4541757382 -0.1037087494 -0.0080032186
C -1.9024640203 0.4374509335 -1.2108221715
C -1.2570754143 0.7045988143 1.1158713904
C -2.1458085816 1.8102268312 -1.2892411175
H -2.0627989532 -0.2123743286 -2.0643546823
C -1.5048251440 2.0694884492 1.0194243215
H -0.9201342372 0.2651533427 2.0488942878
C -1.9527408117 2.6444629015 -0.1825983468
H -2.4948076745 2.2362971016 -2.2264954636
H -1.3550044297 2.7017719723 1.8914179171
C -2.2370162802 4.1253508518 -0.2625030898
H -3.1465459956 4.3823596646 0.2955734772

H -1.4185239967 4.7131949924 0.1684953082
H -2.3805284932 4.4512272685 -1.2970901434
H 1.8852915939 2.4238534043 1.2967155445

H 3.6506625090 2.7713697961 -0.4981489478
H 1.0574513261 -1.0633923549 -1.5074309156
H 0.7115327435 -2.7226946589 0.9246326071

7-Cl

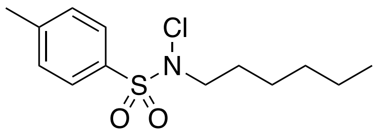

Name	E(B3LYP)	H(B3LYP)	E(RO-B2PLYP-D3)	H(RO-B2PLYP-D3)
Tosyl_NCl_hexane_0040	-1570.919326	-1570.581502	-1570.405971	-1570.068147
Tosyl_NCl_hexane_0066	-1570.917971	-1570.580070	-1570.405171	-1570.067270
Tosyl_NCl_hexane_0054	-1570.918619	-1570.580894	-1570.403791	-1570.066066
Tosyl_NCl_hexane_0013	-1570.916379	-1570.578758	-1570.402871	-1570.065250
Tosyl_NCl_hexane_0019	-1570.918203	-1570.580435	-1570.402272	-1570.064504
Tosyl_NCl_hexane_0003	-1570.917262	-1570.579568	-1570.402167	-1570.064473
Tosyl_NCl_hexane_0034	-1570.917962	-1570.580363	-1570.401630	-1570.064031
Tosyl_NCl_hexane_0029	-1570.916886	-1570.579186	-1570.401296	-1570.063596
Tosyl_NCl_hexane_0084	-1570.916702	-1570.579031	-1570.400938	-1570.063267
Tosyl_NCl_hexane_0040	-1570.919326	-1570.581502	-1570.405971	-1570.068147
Tosyl_NCl_hexane_0066	-1570.917971	-1570.580070	-1570.405171	-1570.067270
Tosyl_NCl_hexane_0054	-1570.918619	-1570.580894	-1570.403791	-1570.066066
Tosyl_NCl_hexane_0013	-1570.916379	-1570.578758	-1570.402871	-1570.065250
Tosyl_NCl_hexane_0019	-1570.918203	-1570.580435	-1570.402272	-1570.064504
Tosyl_NCl_hexane_0003	-1570.917262	-1570.579568	-1570.402167	-1570.064473

38
-1570.068147
C 2.6437842443 0.2095445881 -0.1251751840
C 4.0302707124 -0.0086939550 -0.7474272058
C 5.1613919593 -0.0048475960 0.2890240153
C 6.5510637021 -0.2220761233 -0.3232278569
C 1.5452354851 0.1951457046 -1.1920515614
N 0.1465085084 0.4479085366 -0.7559500409
H 4.2229542191 0.7730338406 -1.4975120302
H 4.0418381439 -0.9642512503 -1.2921733195
H 4.9684058600 -0.7858753618 1.0388136346
H 5.1501333283 0.9498641198 0.8353082173
H 6.5608485743 -1.1761381832 -0.8692771952
H 1.4975852013 -0.7789047992 -1.6883846219
C 7.6754919686 -0.2181659709 0.7174066405
H 8.6541636147 -0.3753454205 0.2497797292
H 7.5300800832 -1.0115475711 1.4608162119
H 2.4442604674 -0.5695984385 0.6197606713
H 2.6239206512 1.1678650080 0.4078993357
S -0.5186356098 -0.7798764294 0.3055173409
O -0.1259784330 -0.5981340197 1.7054555505
O -0.1947031201 -2.0190950709 -0.4097168264
C -2.2642062882 -0.4348245547 0.1547644351
C -2.9158122699 -0.7167292385 -1.0488834487
C -2.9591496914 0.0575936430 1.2576066489
C -4.2835961038 -0.4844903718 -1.1422970924
H -2.3594145446 -1.1159802354 -1.8900823536
C -4.3310599424 0.2807783002 1.1438960045
H -2.4309652324 0.2542925479 2.1839829199
C -5.0115609105 0.0199178546 -0.0519939035
H -4.7986172209 -0.7026208906 -2.0746035114
H -4.8806266522 0.6621378075 2.0006071632
C -6.4918948091 0.2882160986 -0.1783747527
H -6.9671563862 0.3874867320 0.8021915864
H -6.6758137467 1.2191282045 -0.7305208773
H -6.9988685675 -0.5156054374 -0.7233670056
H 7.7132916066 0.7363591111 1.2567303874
Cl -0.0420403648 2.0708764456 -0.0562356154
H 1.7378802263 0.9488131222 -1.9632039190
H 6.7429435151 0.5589911883 -1.0728193758
38
-1570.067270
C 3.0143393603 1.7395165255 0.1954701632
C 3.2722116210 0.8274629916 -1.0139587721
C 3.8113905764 -0.5668577171 -0.6647903156
C 4.1652718729 -1.4004002250 -1.9037912884
C 1.9720543679 1.2213492394 1.1904822971
N 0.6641660629 0.9663264413 0.5278525801
H 3.9906470710 1.3348658333 -1.6728238917
H 2.3445284889 0.7251710386 -1.5912760010
H 3.0712369258 -1.1168956868 -0.0676642120
H 4.7057626603 -0.4658083912 -0.0307262433
H 3.2747984610 -1.4935759097 -2.5419545621
H 1.8537369666 1.9133285460 2.0336510940
C 4.6960438694 -2.7960833328 -1.5611234775
H 3.9570935783 -3.3720234956 -0.9907253475
H 5.6069689551 -2.7351956376 -0.9528448227
H 3.9451510467 1.8777799006 0.7636519384
H 2.7166813401 2.7358920406 -0.1524777953
S -0.3851059634 -0.0674405825 1.4986940824
O -0.9351692446 0.6369503397 2.6596373276
O 0.4344869202 -1.2723534086 1.6695672411
C -1.7025736226 -0.4026921762 0.3436816602
C -2.9660957325 0.1387538182 0.5738836229
C -1.4579938602 -1.2434808652 -0.7452007852
C -3.9987234523 -0.1636087537 -0.3126206393
H -3.1295712815 0.7821495698 1.4313237622
C -2.5009711022 -1.5289869643 -1.6197627107
H -0.4701432325 -1.6642589747 -0.8984937627
C -3.7851154922 -0.9974380706 -1.4178312550
H -4.9865219955 0.2564419155 -0.1417547111
H -2.3186739162 -2.1783908658 -2.4724006429
C -4.9131711586 -1.3411997251 -2.3604402388
H -5.3153178859 -2.3392998482 -2.1426956721

H -5.7388213055 -0.6282001424 -2.2753972426
H -4.5750866350 -1.3495481823 -3.4023298885
H 4.9377749859 -3.3660209343 -2.4656570682
Cl -0.1535942510 2.5051735505 0.1573848883
H 2.2751372296 0.2493465556 1.5880108103
H 4.9122987175 -0.8604851173 -2.5031486023
38
-1570.066066
C 2.2484718608 1.9609606471 -0.6996793161
C 3.5319648399 1.1162715170 -0.7120525512
C 4.0124012435 0.6556381904 0.6712555703
C 5.3632786043 -0.0709796049 0.6236153058
C 1.0176632683 1.2605579929 -0.1138442184
N 0.7290466558 -0.0147209596 -0.8102321340
H 4.3227644470 1.7129880007 -1.1877797355
H 3.3788102715 0.2391080860 -1.3528651349
H 3.2676174576 -0.0145165261 1.1211940667
H 4.0942274544 1.5262934551 1.3408772441
H 5.2830156057 -0.9342649676 -0.0516616924
H 0.1443962812 1.9272992154 -0.1339522704
C 5.8423092283 -0.5432646946 1.9999886585
H 6.8074642232 -1.0583509526 1.9326259241
H 5.1239211096 -1.2382492415 2.4513119525
H 2.4034270470 2.8697295775 -0.1006859366
H 2.0201634381 2.2992347600 -1.7174886869
S -0.2180711505 -1.1853442455 0.0768515990
O 0.4846164616 -1.2583248458 1.3604044474
O -0.3604522095 -2.3382966622 -0.8032774508
C -1.8398911968 -0.4831140582 0.3582475568
C -2.0696402247 0.2823871555 1.5040172355
C -2.8546001160 -0.7014752577 -0.5779146668
C -3.3314298479 0.8386639644 1.7042034709
H -1.2795019769 0.4174987036 2.2347159284
C -4.1078082023 -0.1360862192 -0.3588534132
H -2.6628635496 -1.3160161415 -1.4502423970
C -4.3670995148 0.6407802794 0.7803209408
H -3.5164549120 1.4304675412 2.5971077778
H -4.9007375566 -0.3046565872 -1.0832888094
C -5.7402737203 1.2208391848 1.0195573372
H -6.2072907609 1.5432458296 0.0829514530
H -6.4061050614 0.4756338821 1.4743204901
H -5.7009903076 2.0806155450 1.6955570516
H 5.9633739359 0.3016192537 2.6895864551
Cl 0.0772249228 0.2696678141 -2.4319488029
H 1.2093538242 0.9722688983 0.9221942451
H 6.1181544344 0.5941905564 0.1799997067
38
-1570.065250
C 2.7257960847 0.6428495207 -0.0172710370
C 4.0634575555 1.1240147794 0.5659087593
C 5.3017962575 0.4848256145 -0.0842680005
C 5.4486685986 -1.0244823290 0.1554508291
C 1.5474944279 1.3780425680 0.6282770182
N 0.1785917935 1.0631016690 0.1410580215
H 4.0823802466 0.9287234900 1.6483539334
H 4.1296498043 2.2157121862 0.4515449443
H 6.1980899401 0.9913889124 0.3012387020
H 5.2846204611 0.6811717028 -1.1666891685
H 5.4293572847 -1.2204965286 1.2372418259
H 1.6458750152 2.4619553204 0.5043953379
C 6.7366017723 -1.5980948233 -0.4447807489
H 6.8190075291 -2.6749320062 -0.2588922677
H 7.6239065364 -1.1162493301 -0.0154877426
H 2.7141845928 0.8136557138 -1.1009865577
H 2.6080104310 -0.4351146986 0.1343812363
S -0.3472539762 -0.5799180590 0.4579691267
O -0.0292593108 -0.7403379502 1.8810775008
O 0.1480099809 -1.5389734221 -0.5331097558
C -2.1124300250 -0.4078098931 0.2507332687
C -2.7373647451 -1.0368019254 -0.8240462516
C -2.8448385238 0.3139856183 1.1972076667
C -4.1219283370 -0.9300234578 -0.9539853020
H -2.1457000439 -1.5968058553 -1.5397055725
C -4.2237577658 0.4105118941 1.0478349084
H -2.3399390659 0.7876842182 2.0322436277
C -4.8839081718 -0.2092239379 -0.0262665395
H -4.6165065572 -1.4156474305 -1.7912081695
H -4.8006179424 0.9736920307 1.7773208495
C -6.3847797114 -0.1134891793 -0.1589797780
H -6.7366772217 0.9090755492 0.0174550794
H -6.7203905932 -0.4219111516 -1.1536199654
H -6.8858081894 -0.7583386558 0.5746121904
H 6.7704260587 -1.4445120579 -1.5305273207
Cl -0.0219134175 1.4802599477 -1.5750212397
H 1.5018402236 1.1723465968 1.7021045353
H 4.5867048669 -1.5572671077 -0.2662461783
38
-1570.064504
C 2.6353188455 -0.1533011723 -1.0536274291
C 4.1348105641 0.1676413359 -0.9281172408
C 4.7168580956 -0.0004741636 0.4832661474
C 6.2333276190 0.2263658125 0.5395815469
C 1.7547864384 0.8336631609 -0.2792024263
N 0.2806833747 0.7075128335 -0.4288957466
H 4.3201163823 1.1935668877 -1.2808250285
H 4.6827974488 -0.4900424696 -1.6164384074
H 4.4858863679 -1.0109492421 0.8512528105
H 4.2283815211 0.6945970977 1.1811106479
H 6.7284095998 -0.4745344187 -0.1475742274
H 1.9780920284 1.8663512929 -0.5694743000
C 6.8169846225 0.0611552191 1.9463679648
H 6.3673154551 0.7738391112 2.6487463206
H 7.9003616678 0.2268226690 1.9520518199
H 2.3497241602 -0.1286872196 -2.1115023428
H 2.4433780645 -1.1732266737 -0.6997656321
S -0.3912852745 -0.7805629246 0.2120007994
O 0.2092652287 -0.8436896317 1.5490282845
O -0.2723747294 -1.9074283434 -0.7169223827
C -2.1148669620 -0.3335459990 0.3469249392
C -2.5066595373 0.5955764789 1.3145466102
C -3.0472622118 -0.9548679298 -0.4813606362
C -3.8557794474 0.9086330176 1.4373679536
H -1.7657838882 1.0603126026 1.9561905928
C -4.3959119996 -0.6287993597 -0.3400391443
H -2.7165261669 -1.6777539584 -1.2188565912
C -4.8205527805 0.3024422456 0.6157099181
H -4.1686747324 1.6336475464 2.1847158680
H -5.1290352641 -1.1070692041 -0.9843876932
C -6.2837866046 0.6361246134 0.7800865314
H -6.4365562122 1.7145745492 0.8987175823
H -6.8709749405 0.3017941113 -0.0804704915
H -6.6991653932 0.1510001251 1.6729589384
H 6.6317295664 -0.9472396822 2.3361817829
Cl -0.2396267586 0.9747281459 -2.1077527461
H 1.9153194553 0.7517214941 0.7988716690
H 6.4634299588 1.2334982875 0.1631905956
38
-1570.064473
C 2.6480195454 -0.6450299176 -0.4469034339
C 4.0941040130 -0.7885964174 0.0605641613
C 4.9129859808 0.5109529098 0.0705812046
C 6.3735730217 0.2978985190 0.4900794420
C 1.7683410840 0.1776999111 0.5010910630
N 0.3434580435 0.3747357275 0.1239417474
H 4.6061405369 -1.5211835062 -0.5778122948
H 4.0862927508 -1.2245721380 1.0705579146
H 4.4550328579 1.2460803612 0.7478053405
H 4.8858105514 0.9633523808 -0.9320486820
H 6.3998556296 -0.1611544544 1.4886180533
H 2.1583143028 1.1906471528 0.6366416770
C 7.1944217268 1.5913597948 0.5006942907
H 7.2147943122 2.0562707467 -0.4927059759
H 8.2314297425 1.4045402888 0.8019954315
H 2.6496923206 -0.1802209032 -1.4404909506
H 2.2087488966 -1.6413072736 -0.5672946180
S -0.6177867302 -1.0921082454 0.0981901978
O -0.2839280225 -1.7062891348 1.3879804375
O -0.4774783076 -1.8469323941 -1.1499148942
C -2.2652205373 -0.4061154785 0.1651660481
C -3.1044804711 -0.5397698096 -0.9390174649

C -2.7049896844 0.2035860111 1.3432843181
C -4.4047130520 -0.0413415604 -0.8605054449
H -2.7420581305 -1.0298550664 -1.8358594221
C -4.0039689637 0.6961820817 1.3999895834
H -2.0411803273 0.2822194883 2.1975520075
C -4.8722090060 0.5853801114 0.3012253664
H -5.0661889814 -0.1440152968 -1.7168314194
H -4.3545235717 1.1706241053 2.3132687063
C -6.2724741947 1.1452288988 0.3732935832
H -6.7515582495 0.9015574270 1.3280494851
H -6.2623846616 2.2395740850 0.2870673780
H -6.9023120812 0.7565110408 -0.4325161531
H 6.7717565882 2.3240394779 1.1993377572
Cl 0.1831315216 1.2737665592 -1.4006192753
H 1.7200849980 -0.2926369027 1.4882371430
H 6.8415297829 -0.4281149226 -0.1898347730
38
-1570.064031
C 3.1350685130 1.5430512739 0.5090273446
C 3.3973290733 0.7279959455 -0.7672350624
C 3.8959460700 -0.7031730016 -0.5143328956
C 4.3107594088 -1.4586137096 -1.7884442213
C 2.0447834622 0.9791973233 1.4248841825
N 0.7497172163 0.8393763540 0.7056982937
H 4.1465222686 1.2660739576 -1.3652469942
H 2.4800437845 0.7067792708 -1.3668791937
H 3.1203999265 -1.2842572257 0.0034851186
H 4.7584342070 -0.6628737566 0.1673558574
H 5.0863618309 -0.8835004792 -2.3146522044
H 1.9265613709 1.5959270444 2.3244949998
C 3.1553376247 -1.7564887058 -2.7517369372
H 2.6967181775 -0.8388944139 -3.1370549834
H 2.3695255943 -2.3378907779 -2.2523351682
H 4.0522796485 1.5978606483 1.1125030660
H 2.8817085567 2.5757303288 0.2408429736
S -0.3616555917 -0.2552398812 1.5274183523
O -0.9350714120 0.3404032701 2.7369135625
O 0.4175484593 -1.4964496093 1.5986509804
C -1.6433829762 -0.4249600895 0.2983470313
C -2.9081556444 0.0995834237 0.5564671133
C -1.3722322617 -1.1266799731 -0.8796545222
C -3.9157267864 -0.0771794820 -0.3913834647
H -3.0922826195 0.6335431426 1.4821504010
C -2.3896481687 -1.2874824208 -1.8134804564
H -0.3833957938 -1.5361767637 -1.0561545336
C -3.6751033115 -0.7687220339 -1.5850907988
H -4.9047395093 0.3301946623 -0.1985633011
H -2.1865185266 -1.8277866552 -2.7348260315
C -4.7728071843 -0.9730721534 -2.6012387528
H -5.1113557125 -2.0171207982 -2.6093876100
H -5.6419347588 -0.3446504660 -2.3853815169
H -4.4267557115 -0.7389553973 -3.6143522830
H 3.5010290922 -2.3383126287 -3.6139983315
Cl -0.0073969361 2.4345682375 0.4733103534
H 2.2990375049 -0.0361326150 1.7395896000
H 4.7813885175 -2.4066221555 -1.4955457205
38
-1570.063596
C 2.7687664415 1.2472887472 -0.0119491298
C 4.2400234727 1.1701698916 -0.4593498841
C 4.7931693661 -0.2437862531 -0.7179456784
C 4.7122917693 -1.1969077984 0.4832306982
C 1.8047707821 0.6907190477 -1.0639331695
N 0.3500859573 0.7418292885 -0.7603237408
H 4.8525209166 1.6522908087 0.3145922963
H 4.3713630460 1.7749898594 -1.3683488504
H 5.8442128749 -0.1458272100 -1.0250932786
H 4.2783759988 -0.7014446922 -1.5753064669
H 5.1724898337 -0.7150292354 1.3581513259
H 1.9924138249 -0.3688938422 -1.2548747305
C 5.4000952100 -2.5407181060 0.2198367074
H 5.3245631829 -3.2042770665 1.0885791995
H 6.4651600674 -2.4053938659 -0.0066069309
H 2.6328912409 0.7082847954 0.9316022252
H 2.5125731521 2.2938644984 0.1895577689
S -0.1419389891 -0.2480752427 0.6016155733
O 0.0425415419 0.4213529915 1.8916582463
O 0.5240933102 -1.5252104603 0.3178092946
C -1.8927066341 -0.3993449055 0.2845921241
C -2.7968261235 0.2140193896 1.1492381587
C -2.3285643249 -1.1685728557 -0.7978305035
C -4.1630669521 0.0582206717 0.9155894869
H -2.4313555730 0.7992102333 1.9857879826
C -3.6946332900 -1.3094051642 -1.0143698287
H -1.6081181994 -1.6459149955 -1.4533423205
C -4.6323372257 -0.7012426970 -0.1631293457
H -4.8746740317 0.5357958787 1.5840862816
H -4.0422249389 -1.9029867072 -1.8563508205
C -6.1119929953 -0.8841657558 -0.4008969021
H -6.4336650888 -1.8943303197 -0.1162269312
H -6.7034138265 -0.1722056483 0.1825016882
H -6.3655697075 -0.7514594483 -1.4586216234
H 4.9437421363 -3.0584013984 -0.6330210803
Cl -0.2330535571 2.4109836407 -0.5819614513
H 1.9127850740 1.2211023433 -2.0164543684
H 3.6627087460 -1.3778653077 0.7483080765
38
-1570.063267
C 2.7513169707 0.8092869159 -1.3618207841
C 3.2956228495 -0.2158891886 -0.3590262817
C 4.7504236319 0.0694703107 0.0384641892
C 5.3128013578 -0.9385083826 1.0488499307
C 1.3573152668 0.5278033400 -1.9352394154
N 0.1648494842 0.6626165575 -1.0584253546
H 3.2248449234 -1.2238265606 -0.7920516196
H 2.6720100051 -0.2248567388 0.5420735314
H 4.8203146660 1.0827753478 0.4619792722
H 5.3840495249 0.0728183407 -0.8617045698
H 4.6799640600 -0.9398003475 1.9470680765
H 1.2901152108 -0.4933656441 -2.3217079707
C 6.7644172679 -0.6500604001 1.4449228912
H 6.8609730519 0.3423856568 1.9025908704
H 7.4274553712 -0.6760681680 0.5710666737
H 2.7650859863 1.8135469813 -0.9192707689
H 3.4246647414 0.8551353384 -2.2306158717
S -0.2163229480 -0.6290076753 0.0469732479
O 0.3513348365 -0.4318733558 1.3834913065
O 0.0947966326 -1.8288493643 -0.7375098644
C -1.9889843496 -0.4367956135 0.1701303108
C -2.5470169801 0.0124455571 1.3653452321
C -2.7911210629 -0.7825921501 -0.9205363614
C -3.9339141770 0.1251788110 1.4610014575
H -1.9027920664 0.2660393477 2.1998029424
C -4.1716226432 -0.6629296233 -0.8048863116
H -2.3382729623 -1.1400384948 -1.8391800528
C -4.7649572770 -0.2094232129 0.3847513108
H -4.3756711149 0.4786790549 2.3891158045
H -4.8020622039 -0.9272256558 -1.6503149858
C -6.2668938418 -0.1110949574 0.5029408066
H -6.7112198353 -1.0979068756 0.6878416360
H -6.5625587460 0.5425469705 1.3292178678
H -6.7159636877 0.2778251600 -0.4174051694
H 7.1363706928 -1.3864645441 2.1665214444
Cl 0.0409086058 2.2503817272 -0.2955515062
H 1.1555710878 1.2101787018 -2.7681859608
H 5.2420517450 -1.9510049501 0.6268800432
38
-1570.068147
C 3.2541835719 -0.0927292268 -1.6119951250
C 3.8111308207 -0.2390391290 -0.1839684012
C 3.2122688194 0.7116836780 0.8630467675
C 3.8573500565 0.5571929143 2.2465099125
C 1.9155070247 -0.7827224122 -1.8920263410
N 0.7837407164 -0.2079892991 -1.1140165374
H 4.8962369447 -0.0681351265 -0.2258009869
H 3.6838543046 -1.2798193124 0.1485486349
H 2.1316019683 0.5448169407 0.9400139473
H 3.3381142863 1.7493696443 0.5193716253
H 3.7347724937 -0.4801823989 2.5899318375
H 1.9653757069 -1.8274619619 -1.5695991160

C 3.2740583456 1.5123856686 3.2929171710
H 2.1966714602 1.3494364199 3.4206010786
H 3.4149837498 2.5590500600 2.9961530409
H 3.1922484521 0.9670420147 -1.8893457139
H 3.9625879572 -0.5474046136 -2.3179249688
S -0.6362345474 -1.2631684475 -1.1460668042
O -0.1133314034 -2.5147889223 -0.5889985879
O -1.3062156950 -1.2416940037 -2.4488220530
C -1.6810401823 -0.4667815174 0.0608961602
C -1.3579940719 -0.5717403852 1.4161912505
C -2.8301474311 0.1988387765 -0.3625461598
C -2.1985627347 0.0188412692 2.3539212764
H -0.4717381377 -1.1155474923 1.7251363724
C -3.6611854759 0.7801060073 0.5939819867
H -3.0655962861 0.2505442837 -1.4197952406
C -3.3581817077 0.7058947639 1.9597968876
H -1.9564544565 -0.0599116681 3.4108359172
H -4.5611853410 1.2978641083 0.2725513315
C -4.2466029764 1.3630416028 2.9883753584
H -5.2509027476 1.5452139570 2.5939326373
H -4.3398436919 0.7467967032 3.8890489564
H -3.8341901277 2.3317217506 3.2998988096
H 3.7503427701 1.3763617460 4.2707495306
Cl 0.3574024547 1.4035680548 -1.7461328227
H 1.6899229349 -0.7648729609 -2.9661495916
H 4.9414572945 0.7225581379 2.1645720003

7

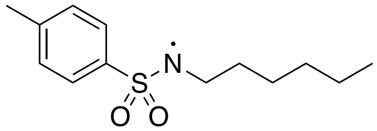

Name	E(B3LYP)	H(B3LYP)	E(RO-B2PLYP-D3)	H(RO-B2PLYP-D3)
tos_Nhexan_react.conf003	-1110.71069972	-1110.377888	-1110.27640585210000	-1109.94359413210000
tos_Nhexan_react.conf001	-1110.71069971	-1110.377889	-1110.27640457360000	-1109.94359386360000
tos_Nhexan_react.conf002	-1110.71069971	-1110.377888	-1110.27640499380000	-1109.94359328380000
tos_Nhexan_react.conf000	-1110.71069971	-1110.377889	-1110.27640366850000	-1109.94359295850000
tos_Nhexan_react.conf014	-1110.70883204	-1110.376086	-1110.27626669770000	-1109.94352065770000
tos_Nhexan_react.conf015	-1110.70883203	-1110.376085	-1110.27626764810000	-1109.94352061810000
tos_Nhexan_react.conf026	-1110.70891722	-1110.375792	-1110.27461793430000	-1109.94149271430000
tos_Nhexan_react.conf020	-1110.70891722	-1110.375792	-1110.27461791830000	-1109.94149269830000
tos_Nhexan_react.conf025	-1110.70891722	-1110.375792	-1110.27461784000000	-1109.94149262000000
tos_Nhexan_react.conf022	-1110.70891722	-1110.375792	-1110.27461771590000	-1109.94149249590000
NH_3	-56.5479477876	-56.509611	-56.53264223626600	-56.49430544866600
NH_2 radical	-55.8726196871	-55.849856	-55.85180557635200	-55.83180557635200
**7-H**	-1111.367419	-1111.020571	-1110.944543	-1110.597695
RSE (**7**/**7-H**)				-22.06

37
-1109.94359413
C 2.8198955362 -1.2508224884 1.7126965727
C 3.2545249098 0.2118802039 1.5267701808
C 4.0126686215 0.4994240166 0.2229863401
C 4.5275350146 1.9425087496 0.1356576521
C 1.8353297369 -1.7567233551 0.6519384011
N 0.5872427098 -1.0099453464 0.7201274987
H 3.8884107013 0.4915665076 2.3797084040
H 2.3651555212 0.8544376245 1.5817997785
H 3.3622541088 0.3043419256 -0.6407169799
H 4.8632690820 -0.1936689993 0.1331830321
H 5.1870781753 2.1449345462 0.9917862886
H 1.5829132316 -2.8137516376 0.8430226602
H 2.2738905632 -1.7281064502 -0.3552023128
C 5.2756013479 2.2379458884 -1.1681976055
H 6.1494995575 1.5842789490 -1.2807707849
H 5.6295811445 3.2747870659 -1.2004905826
H 3.6999678142 -1.9084261102 1.6912648594
H 2.3572172295 -1.3707700900 2.7000686300
H 3.6789964147 2.6342012581 0.2354247324
S -0.3086280725 -1.1069923125 -0.7199348560
O -0.6664994359 -2.5130755459 -0.9604196398
O 0.4310420202 -0.3392923697 -1.7361444546
C -1.7896130037 -0.2077253957 -0.2915324783
C -1.8181045554 1.1796546589 -0.4425623315
C -2.9007304729 -0.9063634973 0.1810631607
C -2.9774832837 1.8712964124 -0.1016960624
H -0.9493461653 1.7017699296 -0.8290812960
C -4.0517368832 -0.1962533695 0.5161459338
H -2.8620925059 -1.9863820500 0.2742772241
C -4.1095669789 1.1978941937 0.3809772953
H -3.0061189329 2.9521131193 -0.2158040334
H -4.9209400018 -0.7345344328 0.8857361278
C -5.3718895334 1.9555164430 0.7163571330
H -5.1488983785 2.9587908735 1.0939730072
H -6.0048898969 2.0756978583 -0.1728047718
H -5.9662156832 1.4307520541 1.4711010544
H 4.6294005891 2.0783402828 -2.0398808593
37
-1109.94352066
C 3.5764748501 -0.8247101297 -0.8911608087
C 4.0262872906 -0.4951177780 0.5437412989
C 3.1148176362 0.4425415175 1.3533082285
C 2.9774861451 1.8603593271 0.7835516220
C 2.2572447754 -1.6015979343 -0.9895282621
N 1.1086852792 -0.7311673463 -0.7889024310
H 5.0316973304 -0.0536625007 0.4891607728
H 4.1408493395 -1.4389549676 1.0957881730
H 3.5177002197 0.5076564059 2.3745659910
H 2.1167095913 -0.0052239544 1.4540668544
H 2.5409952236 1.8140804034 -0.2215548300
H 2.2463035542 -2.4599154916 -0.3005375920
H 2.1492113223 -2.0225148739 -2.0044119284
C 2.1104400892 2.7692794539 1.6612680259
H 1.0905554531 2.3744017122 1.7469339099
H 2.0404384326 3.7804538276 1.2439354952
H 3.4920504720 0.0864255793 -1.4950753601
H 4.3532030265 -1.4403476547 -1.3636593732
H 3.9778055023 2.3037765748 0.6704578766
S -0.3086711632 -1.5900331952 -0.4011240938
O -0.1120158718 -2.2295345310 0.9095448103
O -0.6817832410 -2.3911239001 -1.5773291672
C -1.4899539726 -0.2643785836 -0.2133225864
C -2.1987422670 0.1841719552 -1.3283206291
C -1.6922487053 0.2997954867 1.0469726752
C -3.1136219305 1.2229484800 -1.1729507582
H -2.0396321270 -0.2806925045 -2.2954503201
C -2.6114727174 1.3379468291 1.1817990831
H -1.1460423004 -0.0785837392 1.9044240584
C -3.3346334163 1.8149517787 0.0789875202
H -3.6683037640 1.5779756565 -2.0379476124
H -2.7737038342 1.7819700367 2.1607962103
C -4.3522256582 2.9184821832 0.2421138456
H -4.4510416322 3.5113085875 -0.6729461793
H -5.3436519497 2.5064618198 0.4725100398
H -4.0828898505 3.5946408946 1.0599056497
H 2.5207047257 2.8544121921 2.6754178206
37
-1109.94311607
C 3.0046118589 -1.4516705419 1.6357919858
C 3.9088723118 -0.4552644144 0.8916484048
C 3.2430804315 0.8640347789 0.4789651392
C 4.2252808670 1.8488075160 -0.1702383589

C 1.8415674148 -2.0151851019 0.8075015182
N 0.7327676305 -1.0752551394 0.7349148098
H 4.3231746633 -0.9470464945 -0.0011373288
H 4.7694442894 -0.2329990340 1.5387974258
H 2.7837630944 1.3330989601 1.3611868937
H 2.4217435479 0.6641393158 -0.2216784385
H 4.6897752537 1.3727928385 -1.0454703793
H 1.4389366481 -2.9163721024 1.3015100530
H 2.1824713238 -2.3407759366 -0.1868919676
C 3.5650415238 3.1636409302 -0.5969253183
H 2.7681782400 2.9855642924 -1.3294915285
H 4.2903752025 3.8466697804 -1.0538042145
H 3.6156405841 -2.3031776741 1.9630710678
H 2.5940069112 -0.9902030367 2.5430338572
H 5.0447293491 2.0607999532 0.5317679011
S -0.2805120796 -1.3606108179 -0.6008518025
O -0.7981688768 -2.7351665855 -0.5254705791
O 0.4518026595 -0.9029152682 -1.7935825409
C -1.6255085852 -0.2300649035 -0.2838950802
C -2.7795673744 -0.7010113860 0.3418876445
C -1.5087530490 1.1048988276 -0.6756090952
C -3.8256737500 0.1870281576 0.5854205322
H -2.8545963475 -1.7460471995 0.6226206859
C -2.5639613508 1.9767228243 -0.4221715254
H -0.6100345593 1.4479803719 -1.1769550678
C -3.7362582416 1.5342073436 0.2094191562
H -4.7278213840 -0.1736771261 1.0731186492
H -2.4787843591 3.0179870825 -0.7231275904
C -4.8862611693 2.4829164718 0.4489882658
H -5.5269750979 2.5548198074 -0.4398383710
H -5.5160012883 2.1485127082 1.2794246330
H -4.5312408932 3.4938763451 0.6750561409
H 3.1167208536 3.6790726237 0.2616006848
37
-1109.94195574
C 2.9886100405 -1.3873370911 1.5670632594
C 3.8268940976 -0.2335745669 0.9914163204
C 3.0664250730 1.0827668593 0.7758983807
C 3.9600845939 2.2572327590 0.3405833735
C 1.8825443597 -1.9094274789 0.6393707489
N 0.7103949882 -1.0470474978 0.6656600667
H 4.2781102674 -0.5652579525 0.0460363175
H 4.6655376417 -0.0498681864 1.6788012722
H 2.5531119195 1.3516070539 1.7094613517
H 2.2774670046 0.9377855243 0.0259385256
H 4.7471836099 2.4122221743 1.0930542390
H 1.5372223129 -2.8954029159 0.9956037581
H 2.2628490179 -2.0705269914 -0.3806118261
C 4.6007075771 2.0897849693 -1.0427860124
H 5.1699737321 2.9835860718 -1.3231211655
H 3.8369403702 1.9246812059 -1.8127675422
H 3.6559232913 -2.2314207474 1.7856867809
H 2.5324629192 -1.0896272163 2.5199852520
H 3.3545479689 3.1736059038 0.3426295718
S -0.2558692951 -1.2153523500 -0.7238663817
O -0.6912824263 -2.6160526842 -0.8323450785
O 0.4693510562 -0.5704153697 -1.8312070488
C -1.6723008882 -0.2120681735 -0.3056418189
C -1.6448357031 1.1589111955 -0.5665589252
C -2.7915130223 -0.8162155877 0.2672939721
C -2.7549473363 1.9309645482 -0.2347117207
H -0.7719879797 1.6063230143 -1.0298616193
C -3.8929233401 -0.0266961054 0.5915026664
H -2.7976341462 -1.8862235282 0.4451513946
C -3.8935442078 1.3536107521 0.3470812055
H -2.7395990644 2.9995993496 -0.4342805129
H -4.7680912189 -0.4912808307 1.0388298044
C -5.1035575444 2.1962598785 0.6722977756
H -5.7620970657 2.2903761231 -0.2013145090
H -5.6948527137 1.7542016385 1.4804516570
H -4.8172547675 3.2094112588 0.9732302384
H 5.2912161774 1.2394273049 -1.0746316862
37
-1109.94184673
C 3.4665158047 -0.5560600395 -0.9566399546
C 3.9860783965 -0.4535892862 0.4885525682
C 3.1015596590 0.3157716410 1.4859106440
C 2.8491839770 1.7974057956 1.1580525422
C 2.1649718300 -1.3520803845 -1.1195289648
N 1.0009228260 -0.5599221106 -0.7525023056
H 4.9842070601 0.0029794275 0.4566686877
H 4.1379713996 -1.4717096890 0.8747321068
H 3.5767137270 0.2483770503 2.4755631628
H 2.1348071655 -0.1949876349 1.5822568966
H 2.1743750830 2.2038935724 1.9236993773
H 2.2082406505 -2.3031597835 -0.5668073126
H 2.0257332678 -1.6239807889 -2.1802717003
C 4.1151156866 2.6611863294 1.1049148050
H 4.6800855236 2.5958168668 2.0436506050
H 3.8651555551 3.7158031659 0.9410607199
H 3.3249070205 0.4390279943 -1.3953393623
H 4.2323065744 -1.0571533912 -1.5633444651
H 2.3039868042 1.8775045415 0.2094031054
S -0.3640317536 -1.5194796051 -0.4175717628
O -0.1159737017 -2.1850589707 0.8718910327
O -0.6916188344 -2.3077091828 -1.6153707429
C -1.6300016942 -0.2816210132 -0.1888595030
C -2.4245300941 0.0911190097 -1.2731954627
C -1.8134264066 0.2886169224 1.0719327760
C -3.4091425927 1.0592777578 -1.0870100581
H -2.2761875781 -0.3779200389 -2.2399539790
C -2.8021761050 1.2548456402 1.2375780821
H -1.1974234005 -0.0289684183 1.9064437539
C -3.6138335011 1.6547448677 0.1654460475
H -4.0313230035 1.3548258576 -1.9280775409
H -2.9495947717 1.7038526469 2.2167046356
C -4.7039653419 2.6801924616 0.3655015377
H -4.9379119216 3.2041249922 -0.5666575756
H -5.6300201649 2.2048453834 0.7150612161
H -4.4196610362 3.4263974226 1.1147347903
H 4.7874638074 2.3563487023 0.2944069140
37
-1109.94181742
C 3.1980458668 -1.6105212056 0.4818268984
C 3.9368190502 -0.4493129636 1.1640023836
C 3.3351320258 0.9558976305 0.9945287061
C 3.3489220032 1.4879309083 -0.4454531057
C 1.8247206484 -1.9985196190 1.1119369970
N 0.7932645721 -1.0001118948 0.8852121268
H 4.9654365870 -0.4397391504 0.7742919046
H 4.0249819948 -0.6748838607 2.2366174709
H 3.9042407872 1.6481387157 1.6322100079
H 2.3051252443 0.9622713710 1.3745600752
H 2.7480548465 0.8365905034 -1.0915521034
H 1.9505003625 -2.0622273695 2.2003401636
H 1.5268516096 -2.9846952692 0.7340077601
C 2.8177169681 2.9219462725 -0.5484645470
H 3.4160793523 3.6144868069 0.0569195702
H 1.7820127328 2.9852997198 -0.1907644278
H 3.0525991130 -1.4230552256 -0.5862153622
H 3.8155880856 -2.5146191195 0.5629464262
H 4.3767817425 1.4512919959 -0.8355437958
S -0.1405111817 -1.2947192007 -0.5076182479
O -0.6553854906 -2.6725392060 -0.4825735404
O 0.6537453812 -0.8219531286 -1.6564605166
C -1.5093716830 -0.1765524477 -0.2591448608
C -2.6874377237 -0.6556549874 0.3139770479
C -1.3855243711 1.1584205151 -0.6492163512
C -3.7508818256 0.2242056607 0.5062077793
H -2.7671832970 -1.7005128704 0.5941680298
C -2.4588800558 2.0217132396 -0.4480992951
H -0.4675504313 1.5073450986 -1.1101071213
C -3.6553789836 1.5710401423 0.1302087151
H -4.6716461770 -0.1427295486 0.9526876959
H -2.3687420998 3.0627055267 -0.7485041487
C -4.8230731506 2.5105996537 0.3132483165
H -4.4873598475 3.5248182128 0.5537210732
H -5.4215057606 2.5757095829 -0.6050818313
H -5.4886429023 2.1723981243 1.1136419535
H 2.8358357819 3.2823858173 -1.5834425914

37
-1109.94160795
C 3.0572983983 -1.2164853148 1.4361988246
C 3.8616301860 -0.2053827330 0.6015516019
C 3.0999379630 1.0649678103 0.1983399530
C 3.9417143192 2.0727973473 -0.6041086543
C 1.8749831178 -1.8686338520 0.7062432527
N 0.7156033645 -0.9895732496 0.6705789868
H 4.2332526427 -0.7061222667 -0.3053329939
H 4.7525445145 0.0678229858 1.1819910460
H 2.7058982145 1.5550201513 1.1007238232
H 2.2248071705 0.7867063834 -0.4019580990
H 3.2759927117 2.8637718239 -0.9745945189
H 1.5529716636 -2.7695014041 1.2567609405
H 2.1683264795 -2.2140697812 -0.2966810521
C 5.0872477014 2.7206976615 0.1835016835
H 4.7107427624 3.2286591801 1.0804763339
H 5.6098952824 3.4670620937 -0.4257902029
H 3.7305844243 -2.0241230940 1.7523485300
H 2.6821969812 -0.7417985643 2.3519877743
H 4.3458439715 1.5756172910 -1.4975377589
S -0.3780621188 -1.4018995145 -0.5655961933
O -0.8479366556 -2.7769471682 -0.3374849995
O 0.2507483866 -1.0287546889 -1.8434396696
C -1.7294124737 -0.2799924644 -0.2444498011
C -2.7906344701 -0.7008728235 0.5573606406
C -1.7107566963 0.9970892358 -0.8072152756
C -3.8408654124 0.1807199908 0.8042578422
H -2.7930883954 -1.7040717752 0.9699260448
C -2.7694689671 1.8637620660 -0.5483409465
H -0.8852040115 1.2981230729 -1.4432531479
C -3.8483161098 1.4718579563 0.2580510233
H -4.6705321848 -0.1407836762 1.4289006625
H -2.7606193956 2.8603317202 -0.9828505507
C -5.0062915416 2.4090009017 0.5046885234
H -5.7601185158 2.3180061121 -0.2885465916
H -5.5044952858 2.1898756123 1.4544529382
H -4.6794146580 3.4538348553 0.5234325324
H 5.8321131882 1.9851642244 0.5077563507
37
-1109.94149271
C 3.0796139176 -1.6776380444 -0.5179073309
C 3.9448298081 -0.4302542778 -0.2951813907
C 3.2859502370 0.7032675900 0.5017664952
C 4.2399823463 1.8725989037 0.7796780852
C 1.8834476160 -1.5036204883 -1.5039384059
N 0.8394223401 -0.6245345515 -1.0081970983
H 4.2862111434 -0.0467860002 -1.2685916494
H 4.8532671408 -0.7522153487 0.2349656951
H 2.9066554092 0.3032980356 1.4528045763
H 2.4061850250 1.0707063951 -0.0430487915
H 4.6239797882 2.2635796325 -0.1736927039
H 1.4814097038 -2.4979587126 -1.7388777113
H 2.2562246655 -1.0436625117 -2.4271309638
C 3.5828486039 3.0097910391 1.5684969901
H 4.2872818784 3.8287850336 1.7542053980
H 3.2176689242 2.6569855877 2.5408917091
H 3.6998037641 -2.4707345682 -0.9555471601
H 2.6989745063 -2.0572839257 0.4360226835
H 5.1167150793 1.5039143057 1.3315632818
S -0.2893404851 -1.4021037402 0.0047514430
O 0.2954421533 -1.3969674823 1.3575840221
O -0.7392329505 -2.6640719000 -0.6034630065
C -1.6459649328 -0.2425061976 -0.0374993465
C -2.7376491357 -0.4972328095 -0.8668849910
C -1.5998275799 0.8976382596 0.7681853983
C -3.7935083177 0.4127088146 -0.8917947567
H -2.7576227116 -1.3966787487 -1.4728006453
C -2.6625580938 1.7948688700 0.7275781847
H -0.7487980669 1.0696951286 1.4185443204
C -3.7741035446 1.5680373205 -0.0994036557
H -4.6476662755 0.2199114137 -1.5359894404
H -2.6324666520 2.6852655624 1.3508380876
C -4.9332696147 2.5355174205 -0.1098260440
H -5.5317346723 2.4351770203 -1.0205683765
H -5.6006890854 2.3574211909 0.7436238097
H -4.5893087503 3.5730808221 -0.0399706785
H 2.7253449640 3.4245120489 1.0242403657
37
-1109.94075148
C 3.1547876490 -1.4731055935 1.5864844255
C 4.0652102640 -0.2924605702 1.2012236586
C 3.3813231795 1.0821751095 1.0159925272
C 3.0367652885 1.4369152827 -0.4385539744
C 2.0955295572 -1.8986327477 0.5635657224
N 0.9413065096 -1.0135655470 0.5796191406
H 4.6223828254 -0.5496296099 0.2882943311
H 4.8204425889 -0.2118959766 1.9937415364
H 4.0480997762 1.8646276146 1.4045392097
H 2.4715390134 1.1275613628 1.6290229022
H 2.4005867274 0.6612393101 -0.8815040883
H 1.7313631487 -2.9133982343 0.7989131588
H 2.5147828008 -1.9657201154 -0.4532989554
C 2.3379764414 2.7939269157 -0.5684032806
H 2.9527513692 3.6006928308 -0.1493654985
H 1.3802730167 2.7963140912 -0.0332016215
H 3.7919482429 -2.3487253105 1.7704518912
H 2.6460458217 -1.2557907968 2.5346102510
H 3.9633144877 1.4443165665 -1.0306339933
S -0.1934030337 -1.4012510641 -0.6325204432
O -0.7188858147 -2.7456384916 -0.3481798026
O 0.4181774650 -1.0947172620 -1.9358856979
C -1.4817215748 -0.2073892134 -0.3123916226
C -2.5090519504 -0.5316193996 0.5744621494
C -1.4471384654 1.0291636502 -0.9583367196
C -3.5075617718 0.4074282008 0.8222055980
H -2.5264945203 -1.5055588556 1.0517990039
C -2.4548206420 1.9547747759 -0.6969658393
H -0.6496044641 1.2539158437 -1.6582390375
C -3.4976413794 1.6609152166 0.1935012182
H -4.3103787990 0.1616204386 1.5128364823
H -2.4333701520 2.9203320489 -1.1961040650
C -4.6022307803 2.6592922311 0.4445540792
H -5.4212828578 2.5267067390 -0.2746976281
H -5.0273140288 2.5435393294 1.4467423621
H -4.2432692949 3.6885143635 0.3423869512
H 2.1363865702 3.0407549568 -1.6174009018
37
-1109.94030329
C 3.1753871992 -1.3834520748 0.4218418958
C 3.8278987282 -0.2499873573 1.2272881761
C 3.1495761173 1.1301731543 1.1617185374
C 3.1154093227 1.7941754526 -0.2265232345
C 1.8197191684 -1.9156256170 0.9809045556
N 0.7318408404 -0.9637701860 0.8323239277
H 4.8676929192 -0.1586292581 0.8848019157
H 3.8909781135 -0.5614839391 2.2799465417
H 3.6774731926 1.7982363668 1.8580747461
H 2.1224335878 1.0409583656 1.5365096757
H 2.5569454366 2.7362212734 -0.1349161343
H 1.9336235225 -2.0788916668 2.0602858065
H 1.5905481266 -2.8762866890 0.5027467266
C 4.4962689821 2.0970476602 -0.8212196283
H 4.4046805150 2.6349873532 -1.7717231716
H 5.0679141922 1.1830640404 -1.0200331996
H 3.0351496946 -1.1037756100 -0.6268472536
H 3.8472564860 -2.2517282855 0.4300740786
H 2.5410098726 1.1732641976 -0.9252127464
S -0.1741448085 -1.1882878605 -0.5915316363
O -0.6053979710 -2.5907692610 -0.6965653749
O 0.5985913763 -0.5683980231 -1.6836445289
C -1.6091049077 -0.1801830388 -0.2596559738
C -2.7746789125 -0.7848810959 0.2104865247
C -1.5480136393 1.1968425102 -0.4836751452
C -3.8907476173 0.0094404805 0.4674341024
H -2.8034950127 -1.8584606471 0.3625489035
C -2.6726746471 1.9729922514 -0.2194711832
H -0.6373223583 1.6461692465 -0.8651913690
C -3.8590637476 1.3946875235 0.2578370951
H -4.8023058948 -0.4556179369 0.8342524607

H -2.6315679116 3.0459858456 -0.3899677125
C -5.0814003921 2.2444949677 0.5098288166
H -4.8096608891 3.2322579750 0.8968623572
H -5.6463668121 2.4036811014 -0.4181452437
H -5.7583902312 1.7709321006 1.2276871865
H 5.0936025887 2.7194465012 -0.1424363428

8

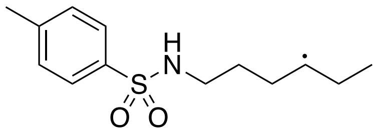

Name	E(B3LYP)	H(B3LYP)	E(RO-B2PLYP-D3)	H(RO-B2PLYP-D3)
tos_Nhexan_15HAT_prod.conf001	-1110.70358121	-1110.371290	-1110.28022547080000	-1109.94793426080000
tos_Nhexan_15HAT_prod.conf002	-1110.70358119	-1110.371290	-1110.28022523770000	-1109.94793404770000
tos_Nhexan_15HAT_prod.conf003	-1110.70358121	-1110.371289	-1110.28022454960000	-1109.94793233960000
tos_Nhexan_15HAT_prod.conf009	-1110.70316421	-1110.370844	-1110.28008446920000	-1109.94776425920000
tos_Nhexan_15HAT_prod.conf060	-1110.70177335	-1110.369075	-1110.27926966650000	-1109.94657131650000
tos_Nhexan_15HAT_prod.conf082	-1110.70039032	-1110.367947	-1110.27891470290000	-1109.94647138290000
tos_Nhexan_15HAT_prod.conf000	-1110.70477491	-1110.372357	-1110.27881190570000	-1109.94639399570000
tos_Nhexan_15HAT_prod.conf069	-1110.70097343	-1110.368720	-1110.27861403580000	-1109.94636060580000
tos_Nhexan_15HAT_prod.conf068	-1110.70097344	-1110.368720	-1110.27861358100000	-1109.94636014100000
tos_Nhexan_15HAT_prod.conf071	-1110.70096255	-1110.368709	-1110.27859177670000	-1109.94633822670000
CH_4	-40.518383	-40.469372	-40.483361	-40.434350
CH_3 radical	-39.838292	-39.804414	-39.807032	-39.773154
**7-H**	-1111.367419	-1111.020571	-1110.944543	-1109.597695
RSE (**8**/**7-H**)				-30.02

37
-1109.94793426
C 2.7208404823 2.2120883319 0.1560672543
C 1.6022038772 2.8887381958 -0.6594206141
C 0.3903492600 3.2265948636 0.1500114777
C -0.9794217032 3.3071591045 -0.4437971648
C 2.3442979861 0.8321030448 0.7042973824
N 2.1244723883 -0.1138708724 -0.4050595898
H 0.5457586752 3.6704586411 1.1349333738
H -1.1102011961 2.4948184636 -1.1740954652
H 1.4035401282 0.9074375591 1.2599227713
H 3.1110190929 0.4664434553 1.3996361348
C -2.1051104575 3.2500003153 0.5985812234
H -2.0837966174 2.3000697589 1.1441955567
H -3.0887300570 3.3490949727 0.1253314857
H 3.6280098222 2.1264649021 -0.4589510176
H 2.9911961687 2.8481783109 1.0097612186
H -1.0878704978 4.2406423418 -1.0303451783
S 1.4993896903 -1.6415778906 -0.0220272422
O 1.8719548185 -2.0378790946 1.3426016053
O 1.8495047858 -2.4803841036 -1.1738155463
C -0.2664189306 -1.3365935657 -0.0259843379
C -0.9728723736 -1.3677462779 1.1743977313
C -0.9192677202 -1.1014611206 -1.2394833107
C -2.3512696524 -1.1459428552 1.1571622426
H -0.4480359131 -1.5763567638 2.1004098551
C -2.2927632368 -0.8830744626 -1.2377979109
H -0.3571772667 -1.1035185713 -2.1676012378
C -3.0299913993 -0.8963163543 -0.0414712513
H -2.9070128978 -1.1751604609 2.0910727624
H -2.8062682534 -0.7068980643 -2.1800179440
C -4.5169871354 -0.6338103035 -0.0544010207
H -4.7256104960 0.4385922866 -0.1628133114
H -5.0061856506 -1.1442751131 -0.8912879920
H -4.9916656342 -0.9691698886 0.8727937301
H -2.0057665055 4.0602432291 1.3311312665
H 2.9553157302 -0.2728457892 -0.9752948579
H 1.3208769942 2.2384796295 -1.4979684816
H 2.0251114644 3.8026443000 -1.1185240737
37
-1109.94776426
C 1.2484780459 2.4983831177 1.8285007518
C 2.3928793109 3.0725829925 0.9615692156
C 1.9185766358 3.8168063332 -0.2480817253
C 2.3294597345 3.4480229854 -1.6386450688
C 0.4136845472 1.4287682540 1.1149792129
N 1.2343682690 0.2462338572 0.8169488716
H 1.3240381848 4.7178980960 -0.0948015097
H 2.1586306586 2.3717558875 -1.7995539116
H 0.0635070760 1.8225712217 0.1570190207
H -0.4679644458 1.1651476020 1.7186440585
C 1.6091104891 4.2507617286 -2.7279746202
H 0.5256886832 4.0897111167 -2.6812492506
H 1.9478478618 3.9561909427 -3.7270803179
H 1.6610759059 2.0830504572 2.7596466682
H 0.5681013392 3.3071048354 2.1275680286
H 3.4218362524 3.5805600050 -1.7558437594
S 0.8103153671 -0.8011641591 -0.4243427912
O 1.8153241066 -1.8653245334 -0.3803132664
O 0.5913340798 0.0309883765 -1.6103819638
C -0.7732073277 -1.5374291862 0.0042057440
C -0.8020879151 -2.7090988828 0.7648895551
C -1.9588534348 -0.9225548222 -0.4028124356
C -2.0307717243 -3.2574321547 1.1244246768
H 0.1276445184 -3.1910514531 1.0495735664
C -3.1796553767 -1.4848977531 -0.0326011576
H -1.9207356541 -0.0301433778 -1.0184669888
C -3.2369499771 -2.6567168963 0.7340874998
H -2.0545702454 -4.1720110408 1.7121884651
H -4.1036785552 -1.0088266244 -0.3517051107
C -4.5631184941 -3.2787886239 1.1012034406
H -4.8580586675 -4.0377327033 0.3645194816

H -4.5174190196 -3.7746672378 2.0764999585
H -5.3615777030 -2.5307220497 1.1358170590
H 1.7922305969 5.3264405702 -2.6144844248
H 1.5527445806 -0.2776138219 1.6289201893
H 3.0519380378 2.2500072178 0.6592546252
H 2.9944643782 3.7336573684 1.6084296066
37
-1109.94707624
C 0.9975473272 2.6472084853 1.7346118666
C 2.2562439304 3.1838778065 1.0224253920
C 1.9695469576 3.8429397090 -0.2891898961
C 2.8325708155 3.7129416969 -1.5039275046
C 0.2634168098 1.5503376349 0.9548791704
N 1.1202441485 0.3665138316 0.7971652969
H 1.1379253840 4.5487665595 -0.3327097317
H 3.6196382538 2.9701656699 -1.3201767153
H 0.0353015820 1.9103631331 -0.0517688798
H -0.6855819633 1.3003112192 1.4537768906
C 2.0466181699 3.3181959372 -2.7708312841
H 1.5884774010 2.3311014738 -2.6516163125
H 2.7069570520 3.2885364972 -3.6457535340
H 1.2678384391 2.2730220739 2.7326492676
H 0.2859734098 3.4678148259 1.8992467439
H 3.3553652580 4.6657755577 -1.7041018521
S 0.7792341383 -0.7751949359 -0.3872660518
O 1.8001355116 -1.8114179555 -0.2201863503
O 0.6015368456 -0.0344453905 -1.6386693934
C -0.8087436408 -1.5147690334 0.0185484703
C -0.8513976572 -2.6357628562 0.8515174053
C -1.9854822686 -0.9512357926 -0.4788277393
C -2.0852856121 -3.1849699874 1.1914533966
H 0.0729693529 -3.0793195651 1.2072022088
C -3.2117389955 -1.5134773627 -0.1268525841
H -1.9351774848 -0.0994487442 -1.1487685426
C -3.2829622928 -2.6350203725 0.7105978618
H -2.1196138135 -4.0605652632 1.8353902073
H -4.1286497073 -1.0775954522 -0.5161836361
C -4.6132415568 -3.2593680800 1.0585195383
H -4.6026715971 -3.6917670526 2.0645543179
H -5.4253540197 -2.5268978896 1.0104193942
H -4.8616446381 -4.0690338561 0.3596407694
H 1.2493455792 4.0425846427 -2.9788321956
H 1.3812383589 -0.0959432961 1.6654068481
H 2.9655943645 2.3603679235 0.8750119682
H 2.7502801652 3.8896761021 1.7165798037
37
-1109.94657132
C 1.1342488355 1.4700261555 -1.7268011217
C 0.8983535613 2.8318575842 -1.0418779593
C 0.2868820551 2.7671474746 0.3257121799
C 0.4218362020 3.8911376316 1.3073266403
C 2.2540072722 0.6271000119 -1.0960434985
N 2.0099049593 0.1630262144 0.2750329019
H -0.5480587285 2.0773663230 0.4619280305
H 1.4436695716 4.2946701375 1.2739253262
H 3.1833922413 1.2119769451 -1.0753721843
H 2.4613238399 -0.2611845612 -1.6990548689
C -0.5739500242 5.0495753492 1.0558115155
H -0.4237022090 5.4924828431 0.0649051649
H -0.4445712356 5.8412046358 1.8030437360
H 0.1995459329 0.8957339119 -1.7441492181
H 1.4096405166 1.6497743070 -2.7747332406
H 0.2649435452 3.5164639048 2.3280433214
S 1.4194579429 -1.3659032236 0.6089254998
O 1.4479821619 -1.4699032595 2.0692485326
O 2.1436089613 -2.2887643664 -0.2690959992
C -0.3131001699 -1.4406102933 0.1218375120
C -0.6600754350 -1.9276049828 -1.1394395542
C -1.2973626547 -0.9942057650 1.0075318672
C -2.0022860191 -1.9495233485 -1.5172341790
H 0.1128145048 -2.3004035863 -1.8032438825
C -2.6334285682 -1.0226124098 0.6136898003
H -1.0167952430 -0.6521239513 1.9985372568
C -3.0081396978 -1.4979650559 -0.6519545810
H -2.2732023011 -2.3321303010 -2.4984133498
H -3.4001833540 -0.6792289215 1.3042315088
C -4.4622841667 -1.5568631765 -1.0553735899
H -4.9253451373 -2.4945215306 -0.7205590668
H -5.0355516412 -0.7361161227 -0.6115318264
H -4.5796469494 -1.5059420949 -2.1426208952
H -1.6098349721 4.6963895576 1.1123544323
H 1.6286282516 0.8650167219 0.9079069213
H 0.2459385046 3.4291173393 -1.7078475147
H 1.8473595243 3.3885104360 -0.9981064040
37
-1109.94647138
C 1.5259474952 1.6863836606 0.9459883828
C 1.2796041494 2.6094730200 -0.2749600150
C 0.2315366839 3.6511615308 -0.0407395376
C -1.2312881899 3.3571390426 -0.1496640270
C 2.6358801743 0.6464323762 0.7248828184
N 2.4279300116 -0.3044989110 -0.3715458370
H 0.5286382299 4.5742975313 0.4567089184
H -1.5995475325 2.8798353062 0.7791089624
H 2.7911989577 0.0485699657 1.6271638420
H 3.5859514225 1.1555555269 0.5167880472
C -2.0864695343 4.6033156152 -0.4250270082
H -1.9555531485 5.3514932333 0.3660361780
H -3.1520050737 4.3517527237 -0.4725454480
H 1.8131008978 2.3025376437 1.8087655338
H 0.5915850809 1.1819427107 1.2173107484
H -1.3985749056 2.6066890525 -0.9368759549
S 1.6226617408 -1.7575612892 -0.1924649574
O 2.0499598356 -2.3078988144 1.0958592117
O 1.8251283038 -2.4541217801 -1.4646260597
C -0.1429995160 -1.4304374662 -0.0593910927
C -0.7487675597 -1.3924023879 1.1971362203
C -0.8935526901 -1.2179289631 -1.2187429642
C -2.1135359878 -1.1196819475 1.2884494857
H -0.1585962774 -1.5971008439 2.0841023084
C -2.2548928007 -0.9450535695 -1.1092433947
H -0.4207299446 -1.2960658885 -2.1926277850
C -2.8856265468 -0.8840899254 0.1429554634
H -2.5882450754 -1.1006432388 2.2664808718
H -2.8416239778 -0.7903319119 -2.0116597608
C -4.3573986027 -0.5633202249 0.2504411357
H -4.7835530614 -0.9435764806 1.1841362347
H -4.5265496362 0.5214958486 0.2308792252
H -4.9232704663 -0.9939836503 -0.5824414242
H -1.8047188382 5.0717452300 -1.3746324507
H 2.3387502836 0.0689566261 -1.3104738601
H 0.9830761853 1.9846326646 -1.1313533860
H 2.2327458743 3.0883276218 -0.5449249989
37
-1109.94639400
C 1.7615100271 1.9760252704 -2.0922378410
C 2.5444365099 3.0174465827 -1.2595097445
C 1.7956919538 3.5949490039 -0.0960729712
C 2.4616510930 4.0065317027 1.1794543267
C 1.5293486905 0.6396281047 -1.3774097190
N 0.5834697060 0.8141617030 -0.2606497700
H 0.8007707926 3.9984433457 -0.2959354362
H 2.9162131397 5.0090429644 1.0589641385
H 2.4866025654 0.2253313382 -1.0326091506
H 1.0921621725 -0.0815121284 -2.0778975034
C 1.5093566982 4.0408078162 2.3856177471
H 0.6714472556 4.7250576623 2.2051906515
H 2.0304455478 4.3823900070 3.2866593730
H 0.7958665294 2.3950165935 -2.4038831754
H 2.3255802370 1.7650425046 -3.0092667495
H 3.3046906249 3.3327589679 1.3888978487
S 0.4129805404 -0.4498339866 0.8464238653
O -0.0806449922 0.1960032364 2.0700293732
O 1.6146339537 -1.2962083613 0.8709859274
C -0.9086588430 -1.4269478019 0.1311657297
C -0.6242145983 -2.6778133316 -0.4116878598
C -2.2149940599 -0.9290283789 0.1450601794
C -1.6638272699 -3.4343845101 -0.9561786831
H 0.3942676476 -3.0505229426 -0.3960481681
C -3.2376014293 -1.6964116434 -0.4008438609

H -2.4209366984 0.0416194047 0.5840869278
C -2.9801038716 -2.9597288281 -0.9599464669
H -1.4458174348 -4.4112764308 -1.3805666668
H -4.2553734404 -1.3132788494 -0.3916472618
C -4.1067882270 -3.7867915030 -1.5320111529
H -3.7290070186 -4.6561897517 -2.0785216802
H -4.7685401477 -4.1554770479 -0.7377875532
H -4.7261044932 -3.1977119731 -2.2183958455
H 1.0935021093 3.0477915619 2.5905199430
H 0.7760674887 1.6647830785 0.2775892642
H 2.8452200155 3.8302652356 -1.9471719652
H 3.4841920346 2.5662792725 -0.9101140739
37
-1109.94636061
C 0.4974782789 1.6071588631 1.5067001667
C -0.1186900798 2.5970425516 0.5009986189
C 0.7560321940 3.7641321185 0.1671201129
C 0.5238450844 4.6154906013 -1.0406092514
C 1.8138076957 0.9649998335 1.0400402670
N 1.7561484934 0.2183932763 -0.2198782516
H 1.3830219962 4.1727939488 0.9609760958
H -0.3490548744 5.2769563181 -0.8756310712
H 2.1958422580 0.2729026091 1.7951208240
H 2.5766418773 1.7401869739 0.9023545543
C 1.7304954108 5.4840195427 -1.4237123227
H 2.6028355000 4.8643893102 -1.6598977700
H 2.0101673748 6.1519330660 -0.6001984512
H 0.7042692586 2.1329042805 2.4496684834
H -0.2346560402 0.8253466329 1.7388119211
H 0.2361317808 3.9801395430 -1.8924497252
S 1.3882954504 -1.4053420313 -0.3229125785
O 2.0623642635 -2.0527425085 0.8050753730
O 1.6514683580 -1.7598527875 -1.7195351474
C -0.3802909460 -1.6230933703 -0.0624657866
C -0.8568147138 -1.9800440094 1.1998130476
C -1.2630640009 -1.4280579011 -1.1276947744
C -2.2300223489 -2.1248299815 1.3947614300
H -0.1554040066 -2.1568888003 2.0083140601
C -2.6315811221 -1.5755317002 -0.9151038760
H -0.8778181528 -1.1868139390 -2.1133053470
C -3.1377428593 -1.9244236594 0.3459933840
H -2.6016523043 -2.4061469088 2.3771895691
H -3.3189567858 -1.4260989363 -1.7444402068
C -4.6209724978 -2.1153681090 0.5563946293
H -4.9285268393 -3.1339595283 0.2851307881
H -4.9014763663 -1.9580119183 1.6027620392
H -5.2059772728 -1.4254808787 -0.0610341464
H 1.5095824270 6.1063508446 -2.2977338064
H 1.5054896538 0.7279560506 -1.0601656581
H -1.0816122067 2.9480942232 0.9195660456
H -0.4014788010 2.0643567204 -0.4205612106
37
-1109.94633415
C 1.2043597333 1.7227679960 -1.5534598002
C 1.2929469354 3.0592081630 -0.7701139870
C 0.9858670915 2.9831001820 0.6983528782
C -0.4203656774 2.8873494440 1.2084961846
C 2.2746131918 0.6925646366 -1.1639554948
N 2.1236527701 0.1069413648 0.1736588748
H 1.7586678116 3.2785940037 1.4073049787
H -0.4398218098 2.3381342126 2.1604243577
H 3.2678678369 1.1597434345 -1.2071465752
H 2.2957964760 -0.1382753953 -1.8747002451
C -1.0735036100 4.2716704409 1.4345014812
H -1.1089076896 4.8484163424 0.5033144420
H -0.5092603583 4.8566992408 2.1693341575
H 0.2068433852 1.2814391024 -1.4396057403
H 1.3275117610 1.9483945602 -2.6215568188
H -1.0407303771 2.3132412157 0.5070929672
S 1.4638521053 -1.4108592697 0.4115051309
O 1.5735631194 -1.6455603549 1.8528546688
O 2.0780414423 -2.2891861603 -0.5874892239
C -0.2950280313 -1.3504985261 0.0278738194
C -1.2037911880 -0.9675973775 1.0174234444
C -0.7375880090 -1.6762754714 -1.2556194111
C -2.5603403834 -0.8970233040 0.7078742225
H -0.8498190822 -0.7504705845 2.0199487030
C -2.0985899125 -1.5990423201 -1.5487186304
H -0.0241676903 -2.0054321916 -2.0036706028
C -3.0299887828 -1.2089238314 -0.5767106178
H -3.2684939350 -0.6039312236 1.4794143794
H -2.4437389240 -1.8557549270 -2.5474496544
C -4.5063175581 -1.1630342719 -0.8915928038
H -4.9925529355 -2.1135167028 -0.6349073827
H -5.0137329750 -0.3761145942 -0.3236445988
H -4.6842938396 -0.9836921782 -1.9567956636
H -2.1000548135 4.1623852263 1.8038517560
H 1.8577297119 0.7612881109 0.9077511710
H 0.5912849205 3.7615003560 -1.2523103235
H 2.2961862658 3.4793421908 -0.9196708800
37
-1109.94612570
C 1.3223505466 2.2071892899 -2.0713336178
C 2.3845409658 3.0637431568 -1.3426324112
C 1.9186075005 3.6611881221 -0.0518186288
C 2.6926575888 3.5967939127 1.2253294684
C 1.0982805872 0.8112825404 -1.4784906484
N 0.7015768606 0.8877150473 -0.0679781520
H 1.0492305236 4.3205847228 -0.0895788097
H 3.3851907055 4.4580514010 1.2967060247
H 2.0267047625 0.2337183145 -1.5153986190
H 0.3482339726 0.2774978003 -2.0845329723
C 1.8065775710 3.5792770683 2.4815458837
H 1.1359928363 4.4477529625 2.5034203612
H 2.4161482391 3.6083601254 3.3918217868
H 0.3683977682 2.7538083445 -2.0999992374
H 1.6201487376 2.0625142616 -3.1181864296
H 3.3341399780 2.7051559445 1.2180075353
S 0.5143231695 -0.5090681324 0.8401057071
O 0.1124079167 -0.0433182145 2.1702707411
O 1.7159409704 -1.3140422467 0.6120724158
C -0.8752617875 -1.4182874185 0.1499475141
C -0.6567012153 -2.3620327020 -0.8558754618
C -2.1680448256 -1.1508582881 0.6078916925
C -1.7466256721 -3.0325103813 -1.4097231497
H 0.3554107067 -2.5809621105 -1.1794212022
C -3.2444882432 -1.8314252552 0.0444422150
H -2.3192992603 -0.4346427503 1.4090621045
C -3.0535642293 -2.7803101733 -0.9712316973
H -1.5773702127 -3.7699393715 -2.1906879924
H -4.2504370455 -1.6284496970 0.4041187672
C -4.2250071006 -3.5366248459 -1.5511197212
H -4.0233181351 -3.8649242884 -2.5758158116
H -4.4452413997 -4.4341400258 -0.9580905032
H -5.1326955981 -2.9241840317 -1.5613014748
H 1.1949981256 2.6716346162 2.5136550380
H -0.0254291006 1.5644166408 0.1486292718
H 2.6905054899 3.8618332701 -2.0428376713
H 3.2812621922 2.4529803935 -1.1719057026
37
-1109.94609332
C 1.8128339350 1.7074310046 0.7063484971
C 1.2209566900 2.5617268516 -0.4427554333
C 0.1929698757 3.5496668818 0.0099150944
C -1.2472712879 3.1854347881 0.1878475229
C 2.8590207004 0.6895139061 0.2414761861
N 2.3088729122 -0.3180343330 -0.6921741869
H 0.5382682465 4.4838574215 0.4536759762
H -1.3930772716 2.6331429468 1.1366029112
H 3.3224754399 0.2016355348 1.1074008348
H 3.6516020247 1.2047395914 -0.3135808352
C -2.1910050781 4.3968912727 0.1820679133
H -2.1324032454 4.9365546231 -0.7699325349
H -1.9290349944 5.1025151056 0.9798154576
H 2.2907281585 2.3644170193 1.4452100029
H 1.0049984250 1.1875092273 1.2352357691
H -1.5456226134 2.4740322277 -0.5968629490
S 1.5957302555 -1.7205916336 -0.0558958095
O 2.0017183667 -1.9106085840 1.3427433849
O 1.8493596820 -2.7489241648 -1.0723699872

C -0.1581978625 -1.3483551081 -0.0531598659
C -0.8455586244 -1.2968720797 1.1574286386
C -0.8253114172 -1.1611882201 -1.2671995739
C -2.2179633328 -1.0406297601 1.1490889786
H -0.3090423474 -1.4587790102 2.0860662758
C -2.1923205828 -0.9052806963 -1.2563925252
H -0.2799449127 -1.2189841194 -2.2035379330
C -2.9111647337 -0.8421323580 -0.0505443111
H -2.7572222620 -0.9991997764 2.0921159134
H -2.7145162428 -0.7571466144 -2.1986728489
C -4.4000393216 -0.5901410629 -0.0587237172
H -4.7690508678 -0.3335423671 0.9388950920
H -4.6638422906 0.2270334951 -0.7394979961
H -4.9477306514 -1.4798459087 -0.3955404472
H -3.2322218338 4.0917109859 0.3364353781
H 2.9793951098 -0.6414772315 -1.3859612533
H 0.7881096973 1.8791019905 -1.1856875465
H 2.0519033404 3.0853181425 -0.9397747891

9

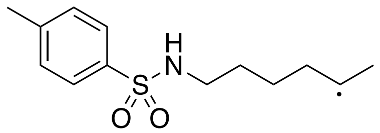

Name	E(B3LYP)	H(B3LYP)	E(RO-B2PLYP-D3)	H(RO-B2PLYP-D3)
tos_Nhexan_16HAT_prod.conf053	-1110.70099866	-1110.368939	-1110.27977059700000	-1109.94771093700000
tos_Nhexan_16HAT_prod.conf063	-1110.70099783	-1110.368396	-1110.27972325810000	-1109.94712142810000
tos_Nhexan_16HAT_prod.conf014	-1110.70185137	-1110.369760	-1110.27917456000000	-1109.94708319000000
tos_Nhexan_16HAT_prod.conf013	-1110.70185131	-1110.369760	-1110.27917418630000	-1109.94708287630000
tos_Nhexan_16HAT_prod.conf010	-1110.70185136	-1110.369760	-1110.27917382750000	-1109.94708246750000
tos_Nhexan_16HAT_prod.conf012	-1110.70185135	-1110.369760	-1110.27917364630000	-1109.94708229630000
tos_Nhexan_16HAT_prod.conf009	-1110.70185136	-1110.369760	-1110.27917363670000	-1109.94708227670000
tos_Nhexan_16HAT_prod.conf011	-1110.70185135	-1110.369760	-1110.27917337060000	-1109.94708202060000
tos_Nhexan_16HAT_prod.conf083	-1110.70019281	-1110.367889	-1110.27908153260000	-1109.94677772260000
tos_Nhexan_16HAT_prod.conf084	-1110.70019282	-1110.367889	-1110.27908084940000	-1109.94677702940000
CH_4	-40.518383	-40.469372	-40.483361	-40.434350
CH_3 radical	-39.838292	-39.804414	-39.807032	-39.773154
**7-H**				-1109.597695
RSE (**9**/**7-H**)				-29.44

37
-1109.94771094
C -0.3720815583 -2.3064477027 0.7750004602
C -1.0926813411 -2.7808645233 -0.4936481378
C -2.6260568202 -2.6909550576 -0.3967730635
C -3.1506527276 -1.2955276699 -0.2729144051
C 1.1584712525 -2.4189803418 0.7065504669
N 1.8190466504 -1.6299053652 -0.3391079028
H -2.9767148126 -3.2966972965 0.4544721622
H -2.5894684393 -0.4933876068 -0.7504093971
H 1.4483668729 -3.4644357228 0.5373474760
H 1.6145789575 -2.1184286627 1.6536592885
C -4.5530454599 -1.0234860646 0.1597584341
H -4.8424240681 -1.6514917175 1.0138634158
H -5.2873220225 -1.2354982259 -0.6395584798
H -0.6607352610 -1.2729453871 0.9990541081
H -0.7098895167 -2.9141232358 1.6269950032
S 2.3699285620 -0.0701020368 -0.1050479862
O 3.0978477135 0.2545492464 -1.3337906024
O 2.9923962492 -0.0475431198 1.2206022695
C 0.9561947999 1.0428216967 -0.0329660504
C 0.3755483450 1.4969472782 -1.2195225448
C 0.4518496020 1.4442664012 1.2051428558
C -0.7301444323 2.3426229483 -1.1577210768
H 0.8050419998 1.2151454926 -2.1756548036
C -0.6539763244 2.2920839928 1.2488108478
H 0.9381206118 1.1120184892 2.1161550353
C -1.2671331646 2.7478132953 0.0735445308
H -1.1752341384 2.7061985976 -2.0808608427
H -1.0408748377 2.6130575330 2.2129698710
C -2.4814672241 3.6438332667 0.1309026782
H -3.4065924251 3.0524720883 0.1545167933
H -2.4744723023 4.2713862999 1.0279824342
H -2.5376343206 4.3001165425 -0.7436289912
H -4.6953291275 0.0248135336 0.4470947064
H 1.5384172861 -1.7937355845 -1.3001769406
H -0.8044375873 -3.8191078633 -0.7110828480
H -0.7645839238 -2.1810193707 -1.3554973184
H -3.0594976446 -3.1820020384 -1.2900993343
37
-1109.94712143
C 0.0690821693 -3.3330841039 -1.2547345754
C -0.8799757752 -3.9486793150 -0.2104645095
C -2.2715207254 -3.2816259135 -0.1009485125
C -2.3136391725 -2.0556441274 0.7587824644
C 0.3750015876 -1.8364040709 -1.1164622395
N 0.9702773400 -1.5376698616 0.1941821023
H -2.9730439703 -4.0335051422 0.3070092529
H -1.9101116946 -2.1498229118 1.7688149528
H -0.5310361062 -1.2374196776 -1.2904140091
H 1.1138653509 -1.5555971099 -1.8716385836
C -3.2337279459 -0.9113398985 0.4864278498
H -2.8950239675 0.0099031339 0.9751717533
H -4.2582910674 -1.1059520724 0.8534758194
H 1.0186710060 -3.8810972578 -1.2296672948
H -0.3587110312 -3.4832408677 -2.2561692805
S 1.8751917221 -0.1333545801 0.3925228458
O 2.8587241789 -0.1131816301 -0.6921493357
O 2.2568980924 -0.1241840889 1.8062500394
C 0.7683847536 1.2616618497 0.1325777552
C 0.0365195234 1.7681785563 1.2100786458
C 0.6297857067 1.8117318842 -1.1427130141

C -0.8452133076 2.8255567584 0.9984712269
H 0.1783992778 1.3510619017 2.2018860597
C -0.2578361630 2.8697494735 -1.3367211276
H 1.2271219172 1.4282127213 -1.9631116374
C -1.0078320403 3.3925029156 -0.2749524608
H -1.4091602111 3.2257398291 1.8378646607
H -0.3632566061 3.3013005960 -2.3291990623
C -1.9407767915 4.5613268449 -0.4850702063
H -1.4434291473 5.5096082226 -0.2420312086
H -2.2755768419 4.6258216832 -1.5252459386
H -2.8267539512 4.4876696078 0.1545550496
H -3.3218022819 -0.7119131754 -0.5893411718
H 0.3106326720 -1.6284524317 0.9650631895
H -0.3990884439 -3.9475424092 0.7774858105
H -1.0125398571 -5.0058894194 -0.4727143932
H -2.6504560734 -3.0476758785 -1.1066567772
37
-1109.94708319
C -0.2080473466 -2.5242494358 -0.5313004973
C -1.1282869578 -2.6351862365 0.6896452624
C -2.6236581444 -2.5390668447 0.3379224293
C -3.0418025790 -1.2094134465 -0.2052872411
C 1.2840797869 -2.6181483795 -0.2000218975
N 1.7530139503 -1.5206513474 0.6764526649
H -3.2086358474 -2.7768230634 1.2484231139
H -2.5369257383 -0.3221568241 0.1740702758
H 1.8763121097 -2.6527983965 -1.1220665433
H 1.4811827918 -3.5429504066 0.3547991356
C -4.3261079196 -1.0341078591 -0.9456810601
H -5.2037460136 -1.0417026337 -0.2723859909
H -4.4905640007 -1.8450313916 -1.6689421700
H -0.4389486700 -3.3309176959 -1.2418770329
H -0.4085248094 -1.5865087394 -1.0640670805
S 2.2990199245 -0.0802155714 -0.0345298523
O 2.6005789257 -0.2933761246 -1.4560601385
O 3.3182716369 0.4196552293 0.8965496799
C 0.8774359703 1.0092970283 0.0480196068
C 0.3952028973 1.4198214736 1.2941662059
C 0.2999679050 1.4727227297 -1.1318120165
C -0.6858884732 2.2931538205 1.3479536476
H 0.8637820492 1.0616188863 2.2049910125
C -0.7830830678 2.3506551691 -1.0589928730
H 0.6984655839 1.1490260273 -2.0871605521
C -1.2911363867 2.7746665763 0.1745226527
H -1.0655961816 2.6129284917 2.3154605313
H -1.2365854960 2.7133106390 -1.9780172526
C -2.4444629367 3.7465946187 0.2506379739
H -2.9695399894 3.8220484586 -0.7064322495
H -3.1706694599 3.4473775780 1.0146474904
H -2.0938314310 4.7526539305 0.5152980009
H -4.3563227173 -0.0832354111 -1.4903043311
H 2.4858013040 -1.8002881405 1.3246849237
H -0.8718490959 -1.8464010542 1.4080763015
H -0.9398069402 -3.5911006563 1.1991371884
H -2.8877510787 -3.3321403394 -0.3803882029
37
-1109.94677772
C -0.3908596177 -2.3296255600 0.9319311898
C -1.1726921625 -2.9006174608 -0.2574103072
C -2.7086303995 -2.9511587099 -0.0289538576
C -3.3687430678 -1.6188592021 0.1401372231
C 1.1348128196 -2.3958300234 0.7645636109
N 1.6938063986 -1.6278806496 -0.3524823181
H -2.9022955482 -3.5794984198 0.8516405724
H -3.4817532270 -1.2139035407 1.1435140306
H 1.4465864376 -3.4386199244 0.6185668823
H 1.6431884388 -2.0427351220 1.6657835280
C -3.6407672081 -0.7149452831 -1.0176168545
H -2.7924404317 -0.0425034951 -1.2391787992
H -4.5057754139 -0.0657965894 -0.8294427863
H -0.6991158002 -1.2942136674 1.1201657680
H -0.6463631667 -2.9011737126 1.8362793599
S 2.2876695066 -0.0751196228 -0.1990820922
O 2.9163478006 0.2126725723 -1.4901478900
O 3.0176947853 -0.0378379309 1.0702382168
C 0.9046832418 1.0656031629 -0.0337698748
C 0.2733309916 1.5538404855 -1.1808308829
C 0.4771244950 1.4584493606 1.2351615226
C -0.8043157388 2.4261715719 -1.0469778767
H 0.6448836532 1.2764673128 -2.1620430359
C -0.6040279995 2.3319834673 1.3509872300
H 1.0038673050 1.1027552851 2.1143280431
C -1.2654812082 2.8235352663 0.2179113554
H -1.2869177990 2.8177022633 -1.9394488341
H -0.9310679456 2.6461240217 2.3391815035
C -2.4495495154 3.7515173406 0.3491283823
H -2.4326067456 4.5332724989 -0.4179932609
H -3.3939762240 3.2038174773 0.2320296165
H -2.4716028613 4.2375959605 1.3294022633
H -3.8397003568 -1.2842359033 -1.9354123927
H 1.3352915337 -1.8131878690 -1.2830797858
H -0.8190309382 -3.9204163037 -0.4702787302
H -0.9719621945 -2.3053151223 -1.1595080873
H -3.1532941501 -3.4723927716 -0.8906130141
37
-1109.94676553
C -1.6076978706 -2.2097770587 -1.7219471887
C -1.3071396975 -3.7056010649 -1.5229851829
C -0.9268397341 -4.1429085667 -0.0927443101
C -1.9357288821 -3.7881865668 0.9543437234
C -0.4473133401 -1.2343649239 -1.4825576820
N -0.1141947442 -1.1549737339 -0.0535335096
H 0.0457491250 -3.7183764595 0.1801701536
H -2.9894586095 -3.9447953592 0.7204278149
H -0.7217226881 -0.2454442669 -1.8835387599
H 0.4527160886 -1.5675133002 -2.0084262304
C -1.5442897689 -3.6228164239 2.3868862043
H -0.6498493270 -2.9930075197 2.4895528722
H -1.2949851799 -4.5896320458 2.8621968154
H -1.9393589635 -2.0579439610 -2.7574958536
H -2.4564027186 -1.9152598298 -1.0881875557
S 1.1510771204 -0.1962883353 0.4803701254
O 2.3005688025 -0.5206891388 -0.3665856093
O 1.1656102424 -0.3670403931 1.9357872084
C 0.7089852575 1.5139358134 0.1448042120
C 1.0723185477 2.0993281118 -1.0695873523
C -0.0203942869 2.2351905967 1.0939646767
C 0.6906092836 3.4144904587 -1.3334531837
H 1.6649203553 1.5368914154 -1.7833127140
C -0.3911733534 3.5478087188 0.8142827746
H -0.2718157710 1.7756269884 2.0444086322
C -0.0441642324 4.1582398123 -0.4006840359
H 0.9757287822 3.8725041123 -2.2774976294
H -0.9536229159 4.1119708954 1.5545202351
C -0.4242382158 5.5931857086 -0.6782752500
H -0.4601865967 5.7983393582 -1.7528779660
H -1.4023407072 5.8388571108 -0.2511181186
H 0.3052366486 6.2851515774 -0.2370322757
H -2.3493176549 -3.1787332038 2.9839638889
H -0.9126028251 -1.0945440855 0.5739831905
H -0.5030608815 -4.0044054277 -2.2092654436
H -2.1995689620 -4.2648854130 -1.8379931130
H -0.7782779740 -5.2402214131 -0.1143262038
37
-1109.94667356
C -0.4658302018 -1.8032588515 -1.9281477775
C -1.7600994800 -2.5121505505 -1.4808012932
C -2.3825328836 -2.0724040849 -0.1373247445
C -1.7883953962 -2.6752228006 1.0984006682
C 0.8376419278 -2.1836579705 -1.2043130212
N 1.0004657950 -1.7073627450 0.1719226967
H -3.4579666422 -2.3368197179 -0.1653613352
H -1.4229821424 -3.7009404641 1.0299007190
H 1.7005140207 -1.8098211264 -1.7623812651
H 0.9376117071 -3.2771648147 -1.1770945849
C -2.1414517495 -2.1557237777 2.4551004728
H -2.1414701280 -1.0582839455 2.4815355715
H -1.4510897661 -2.5167454310 3.2253009381
H -0.3104065534 -2.0476559578 -2.9884707534
H -0.6067472990 -0.7156728346 -1.8850957573

S 1.7770696761 -0.2836180608 0.5540340747
O 2.9500848527 -0.1982463179 -0.3208041904
O 1.8754135841 -0.2866560821 2.0156359430
C 0.7091229848 1.1009912292 0.1180425809
C 0.8574275604 1.7314823086 -1.1182550268
C -0.2716722759 1.5247327953 1.0184503214
C 0.0035379706 2.7808733451 -1.4569200677
H 1.6441394070 1.4117664228 -1.7934414751
C -1.1170864307 2.5738539831 0.6640264816
H -0.3504794585 1.0514658738 1.9919952957
C -0.9956653529 3.2175609963 -0.5766157637
H 0.1215002650 3.2742610700 -2.4187897307
H -1.8778692949 2.9063966255 1.3664704644
C -1.8944233206 4.3767653579 -0.9363601534
H -1.9985848337 4.4824082004 -2.0209985397
H -2.8951033003 4.2569783768 -0.5077710828
H -1.4886426401 5.3225787748 -0.5535850949
H -3.1551768614 -2.4739362916 2.7604246414
H 0.2420014765 -1.9328869342 0.8187986142
H -1.5947221768 -3.5998406024 -1.4641281604
H -2.5084545198 -2.3376548681 -2.2642027909
H -2.3680479380 -0.9740209775 -0.0622846031
37
-1109.94666414
C 0.0867829477 -3.4396020258 0.4507122409
C -1.1367119724 -3.1476962904 -0.4320760964
C -2.1861450199 -2.2269683164 0.2152755779
C -3.4217142799 -2.0643444922 -0.6112650591
C 0.9189788627 -2.2157844369 0.8519710262
N 1.4071049909 -1.5087230316 -0.3456832172
H -1.7499899798 -1.2365169005 0.4083019126
H -3.7958616405 -2.9359320173 -1.1467266809
H 1.7458739896 -2.5178116149 1.5080895489
H 0.3044917340 -1.5075449487 1.4171585097
C -4.3152675972 -0.8775687717 -0.4665600259
H -4.9203846198 -0.9168671011 0.4583768982
H -5.0213691077 -0.7940948453 -1.3007013927
H -0.2324000648 -3.9349590304 1.3781748752
H 0.7399455321 -4.1582303784 -0.0653824574
S 2.2361735852 -0.0502619055 -0.1098033490
O 2.9663565978 0.1638274520 -1.3640000676
O 2.9391175750 -0.0397840540 1.1804323325
C 0.8873281363 1.1242336405 0.0095852170
C 0.0585775522 1.3381707705 -1.0961197219
C 0.7051346422 1.8394223999 1.1913694054
C -0.9667527384 2.2731178562 -1.0023633093
H 0.2176608729 0.7782463467 -2.0117277488
C -0.3278807495 2.7754433086 1.2665943082
H 1.3670011932 1.6618731964 2.0321001330
C -1.1755856795 3.0078887882 0.1774899762
H -1.6146338218 2.4418056606 -1.8592586326
H -0.4742160994 3.3339540513 2.1877209225
C -2.2758241731 4.0395405146 0.2524465695
H -3.2063659783 3.6654788340 -0.1889563519
H -2.4830824959 4.3309698447 1.2865925101
H -2.0002991140 4.9483666069 -0.2982195213
H -3.7368827765 0.0557600578 -0.4133885182
H 1.9927774766 -2.0855433224 -0.9499027606
H -1.6126204749 -4.1049972840 -0.6854614300
H -0.8095129288 -2.7030584429 -1.3791035676
H -2.4421144229 -2.6331440631 1.2152804583
37
-1109.94650268
C -0.4421519776 -1.9390016330 -1.4377512680
C -1.5507038117 -3.0043886354 -1.4669661875
C -2.2936095752 -3.2035557549 -0.1311738849
C -3.0221320427 -1.9905467350 0.3540563354
C 0.7358228121 -2.3159681685 -0.5284622119
N 1.8648016635 -1.3806185563 -0.5546198433
H -1.5976183202 -3.5523682118 0.6468119031
H -3.4545559462 -1.3256748594 -0.3932199941
H 1.1286577729 -3.3004736575 -0.8162814641
H 0.4297414619 -2.3976287519 0.5173142122
C -3.5089468417 -1.8833561460 1.7616121512
H -4.3994738830 -2.5133415701 1.9435108609
H -3.7943026509 -0.8567951995 2.0192730591
H -0.0641137785 -1.7924868283 -2.4598702828
H -0.8596879603 -0.9777219530 -1.1166558223
S 2.0491532251 -0.1396204830 0.5517842669
O 3.3847870158 0.4004577161 0.2890643168
O 1.6533913793 -0.6907566077 1.8501207235
C 0.8796059913 1.1727069346 0.1631330910
C -0.3787866202 1.1844014127 0.7680266335
C 1.2407719096 2.1669938518 -0.7489177302
C -1.2822407238 2.1944346723 0.4411685965
H -0.6372499778 0.4198151809 1.4926885408
C 0.3254535687 3.1690083476 -1.0633520314
H 2.2337434352 2.1633657119 -1.1865346182
C -0.9478768491 3.2001198367 -0.4763909918
H -2.2616391908 2.2055297630 0.9134717979
H 0.6073382998 3.9452481557 -1.7708604294
C -1.9198876069 4.3104125093 -0.7975697958
H -2.9573686942 3.9857909530 -0.6676664955
H -1.7629826932 5.1734759680 -0.1370467593
H -1.7994192543 4.6640169726 -1.8269290475
H -2.7475127272 -2.2158509716 2.4802670366
H 2.2407550913 -1.1354157655 -1.4657069417
H -1.1243801909 -3.9656780244 -1.7863327685
H -2.2827950527 -2.7257420854 -2.2369171007
H -3.0042977278 -4.0429790698 -0.2626711609
37
-1109.94639100
C 0.0178961727 -3.5464179848 0.0278891642
C -1.1171000485 -3.3125257067 -0.9844295182
C -2.4400290014 -2.7959036483 -0.3839565146
C -2.4419295904 -1.3608625515 0.0391515462
C 0.6576675299 -2.2944294220 0.6387531886
N 1.3127973641 -1.4930855358 -0.4130615010
H -2.7338284570 -3.4359617805 0.4638758749
H -1.8713306565 -0.6551452914 -0.5619862638
H 1.3691763724 -2.5808899447 1.4242930310
H -0.1120665594 -1.6660658759 1.0981301823
C -3.4893334854 -0.8206980838 0.9564323979
H -3.2130871748 0.1647039552 1.3483062022
H -3.6687099308 -1.4906616653 1.8088410551
H -0.3540086054 -4.1637521337 0.8572833536
H 0.8089825182 -4.1405217397 -0.4538582755
S 2.1033234510 -0.0702791689 0.0628500630
O 3.0607982567 0.1964669497 -1.0167693204
O 2.5438413844 -0.1574576055 1.4615750213
C 0.7912428794 1.1480517593 -0.0068454666
C 0.3329289513 1.5917797997 -1.2509087519
C 0.2595257139 1.6559517102 1.1763034544
C -0.6776301641 2.5460058039 -1.2987283756
H 0.7712188370 1.1985613579 -2.1622961338
C -0.7542952804 2.6138909648 1.1091003082
H 0.6445731568 1.3108018236 2.1297119389
C -1.2374564051 3.0723692049 -0.1220491993
H -1.0366007854 2.8950771928 -2.2640647745
H -1.1699491050 3.0148047241 2.0302121534
C -2.3168460386 4.1260707183 -0.1932894534
H -3.0951082165 3.8529213998 -0.9149743986
H -2.7943319622 4.2781465966 0.7794016646
H -1.9042477191 5.0911651560 -0.5145034804
H -4.4658668010 -0.7000948646 0.4508146080
H 1.9899305961 -2.0252882735 -0.9606173081
H -1.3185909071 -4.2681496135 -1.4854603634
H -0.7760202802 -2.6206498840 -1.7650276026
H -3.2339462122 -2.9547464012 -1.1412234762
37
-1109.94637510
C -0.0391454657 -2.3816003885 0.9815139581
C -1.2379897750 -2.4214244937 0.0272661642
C -2.5820364985 -2.1367110617 0.7462780633
C -3.7730075023 -2.1598534195 -0.1584841766
C 1.3158351680 -2.5912698147 0.2983303261
N 1.6244056329 -1.5470279250 -0.7042102828
H -2.5026053552 -1.1496090120 1.2284474724
H -4.2778715545 -3.1100985584 -0.3245407896
H 1.3063657622 -3.5359350193 -0.2575582969

H 2.1112810022 -2.6548253659 1.0507601500
C -4.0871884943 -1.0288269946 -1.0822712523
H -3.7776599361 -0.0650819388 -0.6566100870
H -5.1599614277 -0.9736174478 -1.3080671687
H -0.0266391218 -1.4269906174 1.5233589467
H -0.1460664262 -3.1619527782 1.7481476198
S 2.3100569374 -0.0864884294 -0.1777101901
O 3.1395841994 0.3527822254 -1.3064989877
O 2.8729068724 -0.2415248061 1.1700564728
C 0.9054348256 1.0202149526 -0.0426896575
C 0.5736100504 1.5572724906 1.1990434945
C 0.1880003635 1.3677708203 -1.1910086459
C -0.5015553457 2.4432439526 1.2908718811
H 1.1530013169 1.2830947220 2.0740226044
C -0.8812165105 2.2501528936 -1.0805287566
H 0.4666772200 0.9529843214 -2.1542227717
C -1.2433385268 2.8027597915 0.1598064425
H -0.7639707408 2.8628946978 2.2587625954
H -1.4434939325 2.5206585139 -1.9711366272
C -2.3925244091 3.7776504907 0.2575314403
H -2.1234739334 4.7484157325 -0.1783955833
H -3.2726702358 3.4143234401 -0.2851680705
H -2.6836324245 3.9516962517 1.2977855418
H -3.5711983037 -1.1206499363 -2.0555306847
H 2.2229027198 -1.8741194857 -1.4597272112
H -1.2894039869 -3.4058843552 -0.4596601198
H -1.0905865971 -1.6852824849 -0.7712907374
H -2.7095888070 -2.8708646828 1.5545527827

10

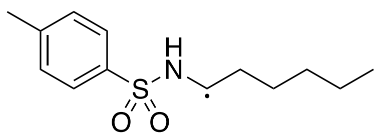

Name	E(B3LYP)	H(B3LYP)	E(RO-B2PLYP-D3)	H(RO-B2PLYP-D3)
Tosyl_NH_C2_hexane_radical_0000	-1110.710641	-1110.37804	-1110.288512	-1109.955911
Tosyl_NH_C2_hexane_radical_0002	-1110.711385	-1110.378686	-1110.288535	-1109.955836
Tosyl_NH_C2_hexane_radical_0001	-1110.710645	-1110.378025	-1110.288422	-1109.955802
Tosyl_NH_C2_hexane_radical_0013	-1110.711593	-1110.379122	-1110.286883	-1109.954412
Tosyl_NH_C2_hexane_radical_0009	-1110.710467	-1110.377875	-1110.286986	-1109.954394
Tosyl_NH_C2_hexane_radical_0019	-1110.710652	-1110.37785	-1110.287071	-1109.954269
Tosyl_NH_C2_hexane_radical_0021	-1110.710652	-1110.377851	-1110.287069	-1109.954268
Tosyl_NH_C2_hexane_radical_0057	-1110.711656	-1110.378951	-1110.286729	-1109.954024
Tosyl_NH_C2_hexane_radical_0064	-1110.710273	-1110.377756	-1110.285792	-1109.953275
CH_4	-40.518383	-40.469372	-40.483361	-40.434350
CH_3 radical	-39.838292	-39.804414	-39.807032	-39.773154
**7-H**				-1109.597695
RSE (**10/10-H**)				-50.97

37

C -1.5451074808 -2.4640860773 0.2832153462
C -2.2133474080 -1.0657544723 0.2528254365
C -3.7075551029 -1.1259870855 -0.0878125636
C -4.3838185963 0.2508992909 -0.1185258281
C -0.0860296819 -2.4572738097 0.6006820327
N 0.8567939224 -2.1799312155 -0.3848747627
H -1.6923707784 -0.4322078000 -0.4785381389
H -2.0728581249 -0.5817389718 1.2285613904
H -4.2221427498 -1.7662324930 0.6442653959
H -3.8397835731 -1.6165059504 -1.0640361300
H -4.2535298667 0.7393386162 0.8575590039
H 0.3072672904 -2.5610147671 1.6033979448
C -5.8757829593 0.1826173741 -0.4605700367
H -6.0362619253 -0.2697443390 -1.4469671055
H -6.3292869807 1.1802505873 -0.4742884156
H -2.0603862711 -3.0879884788 1.0247740693
H -1.7165448412 -2.9514312480 -0.6911698933
S 2.2113928665 -1.1627171280 -0.1327372097
O 2.9892977115 -1.2804420024 -1.3672910886
O 2.7364627219 -1.5346126712 1.1806470897
C 1.6015867957 0.5179392580 -0.0186806251
C 1.5277881291 1.3029186495 -1.1728814417
C 1.1533215813 1.0079831243 1.2104415915
C 1.0003322380 2.5886347995 -1.0867102537
H 1.8984202701 0.9128901504 -2.1152233831
C 0.6248328749 2.2961655057 1.2751162900
H 1.2410420086 0.3964321347 2.1018436543
C 0.5400989221 3.1057891634 0.1340329026
H 0.9505126672 3.2048384772 -1.9813051922
H 0.2798486134 2.6823564321 2.2311988519
C -0.0004383794 4.5131085314 0.2201031645
H 0.8133596210 5.2394755496 0.3461911695
H -0.5435267322 4.7906156953 -0.6895410075
H -0.6781824487 4.6315221980 1.0714672299
H -6.4234189021 -0.4220428583 0.2727597298
H -3.8688609580 0.8906179754 -0.8492801417
H 0.5322398542 -2.1304320956 -1.3472038060
37

C -1.4897250195 -2.6321002994 -0.9353773738
C -2.8946127498 -2.3696260257 -0.3614947683
C -3.2553646154 -0.8828016083 -0.2539671840
C -4.6420425582 -0.6356201528 0.3528855600
C -0.3950297645 -2.1733147091 -0.0265114862
N 0.8994984466 -2.0412123260 -0.5416008865
H -2.9673709649 -2.8392521649 0.6301290847
H -3.6330097785 -2.8803996442 -0.9939815805
H -3.2108425474 -0.4280763579 -1.2553062852
H -2.4932099300 -0.3657719518 0.3456110338
H -5.4003223079 -1.1583587349 -0.2475990692
H -0.4354740224 -2.3725547309 1.0396899737
C -5.0027544986 0.8503850790 0.4478053693
H -5.9964156861 0.9943275607 0.8871021474
H -5.0048305283 1.3219279896 -0.5427261179
H -1.3914508113 -3.7131663870 -1.1468160104
H -1.3965066465 -2.1305347056 -1.9121421539
S 2.0811884921 -1.1013347116 0.2560855061
O 3.2917516425 -1.2768111910 -0.5471515544
O 1.9979766057 -1.4810026647 1.6655657371
C 1.5629761037 0.6076126764 0.1143518851
C 0.6311447664 1.1239813230 1.0182730781
C 2.0602727856 1.3913878248 -0.9293778068
C 0.1944563276 2.4383811502 0.8642034581
H 0.2746543094 0.5119648883 1.8394628221
C 1.6156499768 2.7045994095 -1.0630641990
H 2.7962639342 0.9777495717 -1.6109243562
C 0.6775111397 3.2484593351 -0.1732528722
H -0.5282888407 2.8449261867 1.5674629941
H 2.0072633501 3.3199041364 -1.8695241600
C 0.2257704872 4.6828944638 -0.3092384978
H 0.2194363781 5.0040023530 -1.3560527090
H -0.7800224708 4.8272997798 0.0981773653
H 0.8984391244 5.3595308456 0.2343564209
H -4.2812633042 1.3939066616 1.0703968687
H -4.6846145625 -1.0871542336 1.3543171837
H 0.9886294642 -1.9604195661 -1.5516509147
37

C -1.5359649524 -3.0146434373 -0.1521803335
C -2.4198017236 -2.0081632526 0.6229830008
C -2.8928731681 -0.8201658670 -0.2220339904
C -3.7439334151 0.1862756143 0.5621692732
C -0.2096215078 -2.4846850356 -0.5980221431
N 0.7605307594 -2.2022535445 0.3692244325

H -1.8714151240 -1.6340360461 1.5010648516
H -3.2893845387 -2.5474638438 1.0230909222
H -3.4719172760 -1.1967927771 -1.0788453169
H -2.0190449817 -0.3071371000 -0.6461083962
H -4.6217458575 -0.3255807098 0.9820078212
H 0.1810091407 -2.6504031185 -1.5944572846
C -4.2029134631 1.3752671852 -0.2881797316
H -3.3430541219 1.9267822683 -0.6880096159
H -4.8080650067 2.0771879822 0.2969789429
H -1.3968030053 -3.9103733672 0.4802575566
H -2.0795554164 -3.3576357382 -1.0406275458
S 2.0495592374 -1.1264451026 0.0751626750
O 2.5512053092 -1.4695410293 -1.2546415618
O 2.8715557456 -1.2039605489 1.2835689838
C 1.3497558075 0.5209316036 -0.0126830375
C 1.3089035961 1.3121747599 1.1381628000
C 0.8143775664 0.9808086932 -1.2182396062
C 0.7245980689 2.5745915020 1.0735574448
H 1.7469708788 0.9453987466 2.0605969645
C 0.2291788478 2.2455792535 -1.2614571453
H 0.8770891181 0.3658537200 -2.1093165049
C 0.1755695528 3.0614876320 -0.1224091324
H 0.6993137410 3.1959823949 1.9655189553
H -0.1832740556 2.6091072636 -2.1994859443
C -0.4242248692 4.4458233453 -0.1878463512
H -0.9166302156 4.7143632926 0.7526732843
H 0.3508759024 5.2006220071 -0.3755832569
H -1.1605954555 4.5270226648 -0.9936568594
H -4.8087914048 1.0434896621 -1.1404011628
H -3.1663916913 0.5548638652 1.4219665285
H 0.4608654691 -2.1638399159 1.3399933526
37

C -1.4968488263 -2.9712527046 -0.7578953683
C -2.5815730494 -1.8879290424 -0.9940986779
C -2.6326162428 -0.8038731292 0.0879671521
C -3.7258946276 0.2454426666 -0.1526709493
C -0.0802313828 -2.5036827936 -0.8583963751
N 0.6620420807 -2.2294920146 0.2910050840
H -3.5586185347 -2.3891276906 -1.0534640173
H -2.4110219442 -1.4220360837 -1.9741672796
H -1.6598106303 -0.2976800662 0.1474499870
H -2.7986114435 -1.2775726939 1.0685241248
H -3.5562717737 0.7233295400 -1.1278977245
H 0.4974975759 -2.5729917427 -1.7713231208
C -3.7802243190 1.3187843741 0.9393442502
H -3.9933042655 0.8761504093 1.9203230473
H -4.5605809259 2.0605237409 0.7336745878
H -1.6724894037 -3.4316293567 0.2277767580
H -1.6487991098 -3.7722180209 -1.4936249021
S 1.9748750349 -1.1393737544 0.3026752115
O 2.7353281138 -1.4342983592 -0.9111145058
O 2.5376212702 -1.2624267904 1.6481620923
C 1.2987157874 0.5108480764 0.1337501674
C 0.9894066310 1.0038860502 -1.1368133057
C 1.0466003003 1.2714560905 1.2785161540
C 0.4168870783 2.2692528943 -1.2521938079
H 1.2222947494 0.4163776257 -2.0181246479
C 0.4797282929 2.5364506345 1.1418010529
H 1.3170924646 0.8821455455 2.2544578614
C 0.1490444482 3.0528505504 -0.1206662045
H 0.1855632691 2.6603855148 -2.2399470206
H 0.3003468782 3.1381553570 2.0295425253
C -0.4912053704 4.4140026141 -0.2543280128
H -0.1328102772 5.1027942596 0.5178117785
H -0.2839497979 4.8603945381 -1.2320627733
H -1.5822533543 4.3473748144 -0.1494646210
H -2.8231128989 1.8491098150 1.0180534792
H -4.7019358989 -0.2551173196 -0.2246425413
H 0.1725928437 -2.2372522486 1.1818701240
37

C -1.5076427723 -2.9822658922 0.7238025777
C -2.5983534751 -1.9002638987 0.9373736876
C -2.6362501739 -0.8225376053 -0.1515715935
C -3.7396184657 0.2217972035 0.0632737280
C -0.0933205935 -2.5101829324 0.8357013999
N 0.6500588830 -2.2183198116 -0.3090737275
H -2.4436789886 -1.4280878877 1.9170326948
H -3.5748505868 -2.4038117410 0.9848960457
H -2.7820685807 -1.3025143970 -1.1323564233
H -1.6654999197 -0.3108849820 -0.1964645108
H -4.7141267910 -0.2840018598 0.1173563404
H 0.4848657444 -2.5981434827 1.7467082009
C -3.7772336190 1.2911951821 -1.0332559908
H -3.9646682088 0.8436709204 -2.0172704588
H -2.8231691167 1.8293247849 -1.0909217743
H -1.6647731915 -3.7766360713 1.4652700301
H -1.6713903307 -3.4524976407 -0.2594798966
S 1.9665828765 -1.1336213638 -0.3011597284
O 2.5299301856 -1.2354322753 -1.6481644789
O 2.7250521229 -1.4521565771 0.9079062346
C 1.2986453162 0.5170173437 -0.1043035806
C 0.9861360502 0.9877061315 1.1741132634
C 1.0502009218 1.2979249729 -1.2359393491
C 0.4176816044 2.2525398473 1.3107359095
H 1.2072163894 0.3804227024 2.0449990474
C 0.4870777448 2.5623407486 -1.0780388652
H 1.3142675310 0.9219524514 -2.2188369919
C 0.1617355994 3.0599909167 0.1931014032
H 0.1775771173 2.6235062559 2.3042024336
H 0.3042644594 3.1771695072 -1.9560316509
C -0.4138174683 4.4465755927 0.3559840737
H -1.0234472936 4.7324286317 -0.5073639129
H -1.0370633262 4.5195966307 1.2530345368
H 0.3851225953 5.1937512095 0.4521155676
H -4.5675843503 2.0275509987 -0.8475825006
H -3.5921972107 0.7039649949 1.0399963248
H 0.1607125415 -2.2089858742 -1.1999570191
37

C -1.5331330661 -2.5757769010 0.3908315748
C -2.2106429201 -1.1813304671 0.3924584096
C -3.7144644440 -1.2517578591 0.0944445419
C -4.4410631021 0.1021913860 0.1603125432
C -0.0618979941 -2.5535800314 0.6484354568
N 0.8347859589 -2.2455972485 -0.3705587028
H -1.7068483639 -0.5425488797 -0.3438534281
H -2.0484380037 -0.7070228829 1.3699909151
H -4.1856439984 -1.9410315989 0.8097748371
H -3.8672521475 -1.6961540853 -0.9008168058
H -5.5194186126 -0.0751724756 0.0510210374
H 0.3689911540 -2.6232276644 1.6388493379
C -3.9938923976 1.1163738309 -0.8996119810
H -2.9424066388 1.4000269153 -0.7781501569
H -4.5908060168 2.0337696665 -0.8404945356
H -2.0129109050 -3.2075242339 1.1493655298
H -1.7380544451 -3.0601814455 -0.5784322643
S 2.1419900749 -1.1534439309 -0.1785688389
O 2.8737157349 -1.2364246262 -1.4438622480
O 2.7407821097 -1.4856531357 1.1136952258
C 1.4436267978 0.4917758881 -0.0466896773
C 1.2388004532 1.2471133399 -1.2049666749
C 1.0531689306 0.9778604969 1.2033012775
C 0.6371693859 2.4986335990 -1.1018769729
H 1.5664635368 0.8627246823 -2.1654593074
C 0.4503254010 2.2321128044 1.2856442844
H 1.2416305253 0.3901577142 2.0951494619
C 0.2329357166 3.0113638964 0.1408912853
H 0.4855599869 3.0923883519 -2.0001577590
H 0.1509109343 2.6158139219 2.2579257181
C -0.3864177721 4.3847892452 0.2432387937
H 0.3878511538 5.1621079869 0.2879568654
H -1.0173256471 4.6060907634 -0.6241201593
H -0.9995212086 4.4824813539 1.1446829587
H -4.1137833225 0.7080434199 -1.9112478878
H -4.3049157620 0.5357145470 1.1614297660
H 0.4740211227 -2.2203015304 -1.3210121600
37

C -1.6517340742 -2.7315959537 -0.4895561617
C -2.9154974228 -2.5255672142 0.3698017484
C -3.2466666567 -1.0607421232 0.6987651634
C -3.6664353122 -0.2113919676 -0.5088242054
C -0.4039614811 -2.2813031870 0.1985308557
N 0.7555239908 -2.0808915050 -0.5577343205
H -2.7897910199 -3.0840860482 1.3074723281
H -3.7679001235 -2.9870251277 -0.1469758584
H -2.3776724598 -0.5939162053 1.1830243346
H -4.0584790129 -1.0442847792 1.4396732613
H -2.8604499471 -0.1963443388 -1.2546554018
H -0.2258726695 -2.5096468895 1.2444094394
C -4.0169713837 1.2307544392 -0.1275384359
H -4.8364175118 1.2608043811 0.6012858462
H -4.3280655543 1.8125185505 -1.0028297768
H -1.5866047339 -3.8031587779 -0.7550663913
H -1.7587733025 -2.2004352130 -1.4474871876
S 2.0310502230 -1.0986431862 0.0150998053
O 3.0633242828 -1.2007951330 -1.0171279708
O 2.2475842227 -1.5069688429 1.4021618097
C 1.4202877573 0.5857295274 0.0259408298
C 0.6714748879 1.0421229635 1.1133403991
C 1.6588177162 1.4095399733 -1.0768793443
C 0.1557461710 2.3370770223 1.0853971536
H 0.5181771098 0.4008721735 1.9743272232
C 1.1394321633 2.7017146472 -1.0837713792
H 2.2575739984 1.0429668585 -1.9041963047
C 0.3804379969 3.1859790799 -0.0077438110
H -0.4217763040 2.6981873271 1.9329256358
H 1.3307804501 3.3480149975 -1.9370204879
C -0.1495492726 4.5999517635 -0.0126964172
H 0.6047694660 5.3029613638 0.3648266207
H -0.4171739163 4.9242799249 -1.0238083035
H -1.0351375905 4.6991973972 0.6229370323
H -3.1541629067 1.7384103656 0.3209607538
H -4.5299617058 -0.6827189581 -1.0002074023
H 0.6391206498 -1.9777826956 -1.5629746903
37

C -1.6778277389 -2.7370673577 0.3246558729
C -2.8868056963 -2.4975625778 -0.6023644267
C -3.1858859092 -1.0219705801 -0.9134714872
C -3.6650104508 -0.1971608971 0.2890345904
C -0.3878975384 -2.2798255597 -0.2754319626
N 0.7231788227 -2.0990066721 0.5546592048
H -3.7720028423 -2.9633975092 -0.1484427576
H -2.7105327570 -3.0339280503 -1.5448123252
H -3.9546573534 -0.9796068299 -1.6979805222
H -2.2869642907 -0.5527033337 -1.3368344269
H -4.5582822111 -0.6714540681 0.7207564221
H -0.1467624464 -2.4857215512 -1.3132849602
C -3.9820419412 1.2567780689 -0.0766154952
H -4.3351099360 1.8212203460 0.7941449878
H -4.7606423024 1.3117893940 -0.8475275039
H -1.8379950294 -2.2302028087 1.2882863092
H -1.6366665056 -3.8157269428 0.5649468408
S 2.0306373164 -1.1037329454 0.0843531372
O 2.3352935191 -1.4821089510 -1.2946213896
O 2.9957666470 -1.2271985463 1.1774341990
C 1.4162617469 0.5793097225 0.0711190746
C 0.7435254064 1.0604051672 -1.0550752069
C 1.5806319728 1.3791971401 1.2042015862
C 0.2263597587 2.3545359264 -1.0340356826
H 0.6568003511 0.4409300143 -1.9409302332
C 1.0605481621 2.6715458792 1.2040819697
H 2.1296805979 0.9974697875 2.0587135802
C 0.3697954381 3.1770935729 0.0929490011
H -0.2853150534 2.7372878439 -1.9138098602
H 1.2005739208 3.3018636072 2.0788789652
C -0.2172599687 4.5681603732 0.1143420625
H -0.2355318047 5.0111725467 -0.8867118808
H -1.2517708277 4.5523089906 0.4823494610
H 0.3518957482 5.2335785527 0.7714438225
H -3.0919223086 1.7657968390 -0.4665736671
H -2.9014098755 -0.2071447375 1.0782680396
H 0.5449774388 -2.0180196477 1.5528611818
37

C -1.7602511952 -2.0789107404 0.0791530899
C -2.2482274350 -0.6140701380 -0.0713117019
C -3.6917229851 -0.4815827382 -0.5836322590
C -4.7694478281 -0.9936963209 0.3826425176
C -0.3345084228 -2.2171514895 0.5014319386
N 0.6992181319 -2.0512685112 -0.4158010579
H -1.5733743941 -0.0899531164 -0.7610830784
H -2.1485024204 -0.1036341294 0.8960955669
H -3.7868317458 -1.0085244314 -1.5453139866
H -3.8889161751 0.5781521784 -0.7998351050
H -4.6233134024 -2.0647920583 0.5753542850
H -0.0256347029 -2.3648433366 1.5277913005
C -6.1923780026 -0.7683439034 -0.1394982586
H -6.3894008835 0.2979877356 -0.3055116455
H -6.9420979944 -1.1397171597 0.5682559386
H -2.3856536712 -2.6030414104 0.8106149043
H -1.9222028067 -2.5952540487 -0.8826679209
S 2.1333994105 -1.1821019607 -0.0675616145
O 2.9772946304 -1.3854059738 -1.2464560613
O 2.5253718107 -1.6030128617 1.2771341229
C 1.6971134474 0.5536881865 0.0110608643
C 1.7855217054 1.3380688208 -1.1424840640
C 1.2182440142 1.0918988990 1.2080604284
C 1.3899345342 2.6719935791 -1.0882227486
H 2.1776835867 0.9082328986 -2.0584182998
C 0.8233461407 2.4282732519 1.2408075411
H 1.1797987310 0.4776625774 2.1010924233
C 0.9024818979 3.2383686774 0.0996241922
H 1.4664390353 3.2869037452 -1.9818084402
H 0.4549708894 2.8515375993 2.1721601245
C 0.5063541018 4.6944973933 0.1534280139
H 1.3791892778 5.3321917127 0.3465175597
H 0.0667236166 5.0258895763 -0.7932743708
H -0.2188222770 4.8841328010 0.9511717007
H -6.3507624265 -1.2855074497 -1.0939026069
H -4.6507533701 -0.4899494245 1.3528409381
H 0.4495473529 -1.9713856260 -1.3982466098
37

C -1.7059140523 -2.1259862817 -0.6190905011
C -2.2200110969 -0.6676462147 -0.7473297540
C -3.7504873948 -0.5313620661 -0.6878216254
C -4.3799670099 -0.8686860997 0.6715754371
C -0.2238581905 -2.2727568061 -0.7339698680
N 0.6022263275 -2.0681712324 0.3674085666
H -1.8578645970 -0.2572651837 -1.6984889754
H -1.7637041959 -0.0535983637 0.0416854854
H -4.2023031905 -1.1663591114 -1.4647737896
H -4.0177613117 0.5022533212 -0.9503182748
H -4.1717977194 -1.9144257582 0.9342241333
H 0.2880157941 -2.4124781336 -1.6772598406
C -5.8947664402 -0.6384525254 0.6988815434
H -6.1402151336 0.4081667791 0.4804347446
H -6.3196134933 -0.8832422032 1.6789444811
H -2.0461339766 -2.5458392945 0.3399334343
H -2.1793075153 -2.7356158288 -1.4001985209
S 2.0642549538 -1.1782301939 0.2983705987
O 2.7224131999 -1.6083950612 -0.9348528699
O 2.6587383700 -1.3528800834 1.6247583895
C 1.6294331167 0.5493540945 0.1082310528
C 1.4649443361 1.3463091402 1.2447824700
C 1.4043057918 1.0685595989 -1.1690163651
C 1.0722920361 2.6733700221 1.0922291174
H 1.6632610072 0.9317571162 2.2278064023
C 1.0081024857 2.3985297063 -1.3005232197
H 1.5613246132 0.4448928376 -2.0423314603
C 0.8367151007 3.2207832513 -0.1783855286
H 0.9527257353 3.2981068828 1.9742173812
H 0.8372315948 2.8071243304 -2.2935518244
C 0.4423464463 4.6702938723 -0.3318579996
H -0.0928608754 4.8435881638 -1.2708447213
H -0.1985305362 5.0011260499 0.4923212958

H 1.3275709177 5.3200088052 -0.3350405107
H -6.4031499777 -1.2595014154 -0.0488753034
H -3.9025768022 -0.2565431418 1.4506323887
H 0.1595384744 -1.9799067594 1.2785196938

TS 7 → 8

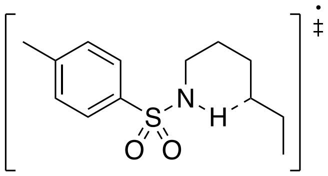

Name	E(B3LYP)	H(B3LYP)	E(RO-B2PLYP-D3)	H(RO-B2PLYP-D3)	NImag
tos_Nhexan_15HAT_ts.conf054	-1110.68208233	-1110.354755	-1110.25745003380000	-1109.93012270380000	-1612.3601
tos_Nhexan_15HAT_ts.conf053	-1110.68208240	-1110.354755	-1110.25744803410000	-1109.93012063410000	-1612.3838
tos_Nhexan_15HAT_ts.conf052	-1110.68208240	-1110.354755	-1110.25744776770000	-1109.93012036770000	-1612.3931
tos_Nhexan_15HAT_ts.conf020	-1110.68402560	-1110.356788	-1110.25713145580000	-1109.92989385580000	-1581.8940
tos_Nhexan_15HAT_ts.conf019	-1110.68402559	-1110.356788	-1110.25713067150000	-1109.92989308150000	-1581.9230
tos_Nhexan_15HAT_ts.conf004	-1110.68429341	-1110.357017	-1110.25711119540000	-1109.92983478540000	-1676.5275
tos_Nhexan_15HAT_ts.conf008	-1110.68429353	-1110.357016	-1110.25711138580000	-1109.92983385580000	-1676.4128
tos_Nhexan_15HAT_ts.conf003	-1110.68429350	-1110.357017	-1110.25710989860000	-1109.92983339860000	-1676.5442
tos_Nhexan_15HAT_ts.conf012	-1110.68429354	-1110.357016	-1110.25711006310000	-1109.92983252310000	-1676.4739
tos_Nhexan_15HAT_ts.conf010	-1110.68429354	-1110.357016	-1110.25710999950000	-1109.92983245950000	-1676.4646

37
-1109.93091650
C 2.3906540783 2.5248232458 -0.6234641288
C 3.6410673242 1.6873072123 -0.2965915715
C 3.4831842979 0.2508678271 -0.7651370585
C 4.2257467712 -0.8354284953 -0.0168759768
C 1.1476518416 1.9261043095 0.0523311664
N 0.9514296074 0.5522811637 -0.4376042622
H 2.2356835579 2.5581593279 -1.7094845124
H 2.5180042931 3.5579692046 -0.2803958701
H 3.8146574029 1.7050375232 0.7881011756
H 4.5257035606 2.1447826770 -0.7656344670
H 3.5309424585 0.1467104223 -1.8565182337
H 2.1807193778 0.1093258324 -0.6107005250
H 3.9415677332 -0.8051806342 1.0439671993
H 5.3021252475 -0.5894909934 -0.0425188762
H 0.2589227395 2.5143509932 -0.2171042056
H 1.2577621906 1.9612747243 1.1443196676
C 4.0056766876 -2.2436602915 -0.5792384416
H 4.5965923204 -2.9804043146 -0.0240241461
H 2.9515360524 -2.5275291457 -0.5073933722
S 0.2043691234 -0.5281525451 0.6384686906
O 0.3900185138 -0.1281183504 2.0442584413
O 0.6066765753 -1.8686917236 0.1934324278
C -1.5251273504 -0.2885698953 0.2288606968
C -2.0303679121 -0.8325072225 -0.9558402951
C -2.3453007482 0.4326780377 1.0951080655
C -3.3720455556 -0.6426363707 -1.2685011822
H -1.3786644348 -1.4005154536 -1.6112623457
C -3.6890755841 0.6122484749 0.7646889719
H -1.9352393390 0.8310392284 2.0170352491
C -4.2224294510 0.0808331629 -0.4158916458
H -3.7703874613 -1.0648528569 -2.1881378398
H -4.3336485642 1.1706559148 1.4390855643
C -5.6824342502 0.2566534662 -0.7592563221
H -6.2417703840 -0.6725335761 -0.5890752540
H -6.1471811624 1.0373930450 -0.1493694905
H -5.8160662158 0.5239207463 -1.8135957025
H 4.3078401614 -2.2989699561 -1.6325240443
37
-1109.93051504
C 2.5634955749 2.2653934434 -0.3602249406
C 3.7301423472 1.4814043049 0.2658164369
C 3.4848297473 -0.0165257243 0.1999394450
C 4.1211803518 -0.9108303512 1.2429067853
C 1.2379138196 1.9353505683 0.3585511282
N 0.9813240695 0.4866884326 0.4526821723
H 2.4625661111 2.0097075102 -1.4211200313
H 2.7457600300 3.3452200460 -0.2970729662
H 3.8642020401 1.7910129950 1.3120077226
H 4.6644349695 1.7333284737 -0.2596983447
H 3.5397984735 -0.4202519264 -0.8171789616
H 2.1866327222 -0.0321053513 0.4177521410
H 3.8357965993 -0.5605252810 2.2449577642
H 5.2169101821 -0.7874640527 1.1873249642
H 0.4061232256 2.4296371888 -0.1562050353
H 1.2681303055 2.3131224888 1.3880495832
C 3.7634135140 -2.3921266005 1.0779018440
H 2.6791284732 -2.5409384849 1.1113718267
H 4.1151587618 -2.7759782644 0.1133438874
S 0.1713816105 -0.2186295318 -0.8671342166
O 0.6310827900 -1.6129111926 -0.9085112947
O 0.2336313566 0.6206756328 -2.0773777090
C -1.5246119326 -0.1858592709 -0.2817974278
C -1.9032195699 -1.0178646536 0.7765677150
C -2.4493623404 0.6524683867 -0.9011488257
C -3.2223189004 -0.9970931556 1.2147075460
H -1.1720147456 -1.6707242208 1.2414004078
C -3.7696907834 0.6591915552 -0.4479569196
H -2.1359979287 1.2772236728 -1.7306509056
C -4.1764547519 -0.1602277585 0.6112734464
H -3.5213871638 -1.6422416437 2.0375604734
H -4.4949861744 1.3099959295 -0.9300890426
C -5.6092276704 -0.1653636189 1.0883112146
H -6.1790041496 0.6630991856 0.6566628475
H -6.1152902604 -1.0980440008 0.8075250576
H -5.6671970603 -0.0845971166 2.1799470694
H 4.2207775578 -2.9974422776 1.8682830210
37
-1109.93019693
C 2.4105391580 2.2279150797 0.2991541605
C 3.5821070303 1.7248874700 -0.5646545849
C 3.4953803451 0.2271144346 -0.8397040765
C 4.0339142382 -0.7188028671 0.2184607349
C 1.0696109312 1.9129323837 -0.3908584359
N 0.9266099219 0.4890546009 -0.7498598779

H 2.4887643988 3.3110776070 0.4544488152
H 2.4253978981 1.7520450244 1.2852279499
H 4.5315053780 1.9533605182 -0.0568134777
H 3.5887245386 2.2771746233 -1.5132324529
H 3.7922099631 -0.0556272544 -1.8560407587
H 2.1795456123 0.0934098002 -0.9112288660
H 3.4959374296 -0.5733291275 1.1645097832
H 5.0787512546 -0.4259612005 0.4209138481
H 0.9921763980 2.4775491009 -1.3284680903
H 0.2362934368 2.2220145727 0.2513300967
C 3.9889805693 -2.1963539444 -0.1853669709
H 4.4436741676 -2.8230054725 0.5898243939
H 2.9572715701 -2.5293747840 -0.3271478758
S 0.2593390542 -0.5063623934 0.4558022409
O 0.5444275564 -0.0188728284 1.8185537126
O 0.6186845974 -1.8788410175 0.0783439342
C -1.4931360155 -0.2651590633 0.1547717165
C -2.0639571455 -0.7707389325 -1.0176833895
C -2.2685775135 0.4020324067 1.1012156168
C -3.4256167143 -0.5953082161 -1.2363297232
H -1.4458519163 -1.2941733129 -1.7394435624
C -3.6341872951 0.5679072838 0.8643506007
H -1.8059841297 0.7706069508 2.0105754877
C -4.2324843990 0.0750457223 -0.3012113434
H -3.8744514560 -0.9862747545 -2.1464655569
H -4.2436762105 1.0853600292 1.6010838796
C -5.7135109143 0.2381762472 -0.5465879456
H -5.9129915730 0.5985000004 -1.5623406001
H -6.2391551682 -0.7190547512 -0.4357854821
H -6.1618023090 0.9450937032 0.1581093917
H 4.5419801495 -2.3652152855 -1.1180599343
37
-1109.93012270
C 2.3457788244 2.3159508286 -0.1605232816
C 2.1604254969 1.7139526486 -1.5714407111
C 2.2248273369 0.1912480255 -1.5555707437
C 3.5886060681 -0.4723800338 -1.5517869390
C 1.3081945122 1.7509098118 0.8200618154
N 1.4875883017 0.2964294151 0.8887190525
H 2.2465430405 3.4067404611 -0.1991597672
H 3.3513471517 2.0945316969 0.2164518731
H 2.9313107101 2.1204511551 -2.2436336670
H 1.1909176378 2.0389540801 -1.9695089966
H 1.5070575667 -0.2965011570 -2.2236017242
H 1.7684008086 -0.0394883055 -0.3540409103
H 4.2137076004 -0.0232522990 -0.7685897674
H 4.0892601413 -0.2421652424 -2.5077056205
H 0.2956510758 2.0438717928 0.5000504472
H 1.4711315874 2.1582634506 1.8255929774
C 3.5203279263 -1.9909281493 -1.3534130713
H 4.5227571422 -2.4324702985 -1.3517248726
H 3.0344100171 -2.2404614128 -0.4040861412
S 0.2255173916 -0.6407607378 1.4909238515
O -0.0427412715 -0.1442990400 2.8475711851
O 0.6147424284 -2.0308278641 1.2390293161
C -1.2447957075 -0.3140794522 0.5063000947
C -1.4560516875 -1.0363717387 -0.6718857042
C -2.1573063251 0.6589518790 0.9174210612
C -2.5842515404 -0.7686504920 -1.4423071591
H -0.7541356371 -1.8116157035 -0.9615490523
C -3.2821638823 0.9147087633 0.1326927763
H -1.9958784782 1.1870023734 1.8512963217
C -3.5138080382 0.2093713090 -1.0551255309
H -2.7531220158 -1.3345049549 -2.3555827430
H -3.9952490764 1.6700562451 0.4539797724
C -4.7498502845 0.4669608894 -1.8837134194
H -5.1748059610 1.4537239403 -1.6748457074
H -4.5332621585 0.4089108462 -2.9559520436
H -5.5281326899 -0.2774904711 -1.6702724465
H 2.9473751962 -2.4674025139 -2.1585455922
37
-1109.92989386
C 2.5363905233 2.3896820504 0.2168223402
C 3.7430167233 1.5523149432 -0.2614547299
C 3.5810091977 0.0762692749 0.0776120285
C 3.9522618260 -0.3887017389 1.4720916526
C 1.2270893308 1.8471067129 -0.3746663131
N 1.0470460701 0.4604034162 0.0811817786
H 2.6582082201 3.4365299684 -0.0842067150
H 2.4736392586 2.3724282939 1.3117672542
H 4.6631362920 1.9490265548 0.1941855006
H 3.8480274406 1.6730846884 -1.3465558544
H 3.9042756436 -0.6100760537 -0.7109565405
H 2.2725462339 -0.0133701759 0.0582433759
H 3.4462728878 0.2398001264 2.2177341189
H 5.0325072571 -0.2169874211 1.6183284632
H 1.2400026128 1.9231160204 -1.4702727163
H 0.3786607864 2.4409942666 -0.0055357947
C 3.6256499526 -1.8649491817 1.7240875568
H 3.8900827728 -2.1576982309 2.7461800041
H 2.5594026116 -2.0623238620 1.5721546383
S 0.1479788188 -0.5651878040 -0.9326933075
O 0.5825099291 -1.9252179157 -0.5908189238
O 0.1611363707 -0.1085143536 -2.3329919725
C -1.5105765185 -0.3159033081 -0.2968325620
C -1.8818314469 -0.9234018948 0.9061314021
C -2.4102230661 0.4766536412 -1.0084766097
C -3.1687435592 -0.7251538175 1.3950476972
H -1.1714184770 -1.5468150644 1.4386021401
C -3.6968871862 0.6638722992 -0.5024416454
H -2.1061307002 0.9234624206 -1.9490787521
C -4.0960699734 0.0694153294 0.7010871824
H -3.4629187059 -1.1969252969 2.3296844253
H -4.4030796587 1.2780489664 -1.0558279746
C -5.4977272985 0.2527480675 1.2322475576
H -5.9982984517 1.1034536767 0.7596107508
H -6.1105157394 -0.6380456064 1.0415271088
H -5.4969429384 0.4170165070 2.3155755023
H 4.1808413643 -2.5124114892 1.0350313393
37
-1109.92983479
C 3.3785550588 1.5763802625 0.5624981302
C 3.8844371262 0.1933405727 0.1152534932
C 2.7769180354 -0.6403073669 -0.5078823666
C 2.8745826726 -2.1481436356 -0.4148812506
C 2.2030245174 1.4220470868 1.5483642016
N 1.1031111347 0.5919462162 1.0122294379
H 3.0507412262 2.1562766962 -0.3080157401
H 4.1848562768 2.1396247500 1.0482845104
H 4.3043785089 -0.3381819621 0.9812008682
H 4.7077930142 0.3190354754 -0.6052605551
H 2.4576367819 -0.2707880503 -1.4881416699
H 1.7423180658 -0.2358573023 0.2371364562
H 2.9901915855 -2.4433920621 0.6374912886
H 3.8056118481 -2.4680777929 -0.9147209793
H 1.8025266331 2.3993212163 1.8379473775
H 2.5471742994 0.9187426467 2.4604197785
C 1.6858343568 -2.8862994007 -1.0399593439
H 1.8080751142 -3.9718183027 -0.9569808392
H 0.7463391931 -2.6139854588 -0.5459622310
S -0.0020336330 1.4472020088 0.0380422260
O 0.4938281169 1.6414285632 -1.3373191964
O -0.4340638096 2.6170650898 0.8193834787
C -1.3442432535 0.2623517043 -0.0319275566
C -1.6820413722 -0.3160575615 -1.2529186578
C -2.0657412867 -0.0329150388 1.1286374554
C -2.7512248619 -1.2127854321 -1.3069547814
H -1.1172523490 -0.0591974121 -2.1425299995
C -3.1261468478 -0.9289393388 1.0562500626
H -1.7956594790 0.4344043393 2.0697156349
C -3.4866339606 -1.5328105091 -0.1604409443
H -3.0181976317 -1.6665757427 -2.2581103498
H -3.6884326582 -1.1639141559 1.9569999651
C -4.6505054469 -2.4932401753 -0.2215294745
H -5.5981949321 -1.9796273691 -0.0159608861
H -4.7330272033 -2.9615261198 -1.2069425510
H -4.5492372595 -3.2905442206 0.5243369513
H 1.5852359095 -2.6409919621 -2.1039364139
37
-1109.92967719

C 3.2494932339 2.1924071277 0.2573167525
C 3.7472784325 1.0036960608 -0.5855942656
C 2.6092450838 0.3609141221 -1.3593242808
C 2.7001828180 -1.1180546761 -1.6666262589
C 2.1889798911 1.7320123669 1.2653578689
N 1.0417539770 1.1606985208 0.5244629174
H 2.8212162542 2.9627883414 -0.3971407614
H 4.0807286107 2.6539226903 0.8026797963
H 4.2086371500 0.2592132927 0.0775506150
H 4.5342720904 1.3415613690 -1.2772528546
H 2.2597222228 0.9735078421 -2.2000369759
H 1.6088994264 0.5947547372 -0.5015763871
H 2.7263819277 -1.6790900083 -0.7234262491
H 3.6691879892 -1.3091865070 -2.1599978772
H 1.8192726923 2.5816200603 1.8508422253
H 2.6382876426 1.0159968503 1.9676923390
C 1.5679209339 -1.6401891327 -2.5564402610
H 1.6905658852 -2.7096597482 -2.7596565753
H 0.5920171902 -1.5008207017 -2.0787060894
S 0.2113288404 -0.0238674649 1.4057852330
O -0.1389920124 0.5870636497 2.6970990973
O 0.9229357400 -1.3152915576 1.3927324709
C -1.2686624468 -0.2048041790 0.4129160052
C -1.6163624799 -1.4677008236 -0.0611476507
C -2.0947281611 0.9003972067 0.1863619602
C -2.8011241670 -1.6202201049 -0.7844890409
H -0.9664413913 -2.3126466629 0.1393815346
C -3.2685993809 0.7304463367 -0.5389474362
H -1.8143798199 1.8760595744 0.5687580899
C -3.6414261949 -0.5301180595 -1.0357740372
H -3.0752501106 -2.6044131929 -1.1559881594
H -3.9114101821 1.5884728081 -0.7215968500
C -4.9211883528 -0.6939110727 -1.8206448336
H -4.9057338620 -0.0897682263 -2.7362691927
H -5.0834122022 -1.7364010451 -2.1102954962
H -5.7900293681 -0.3681896840 -1.2358980014
H 1.5466999715 -1.1167982356 -3.5202481511
37
-1109.92962961
C 1.2369127025 2.2856274090 -0.4934748035
C 2.3181590910 1.6838033611 -1.4083483496
C 2.2526297382 0.1646118192 -1.4059848820
C 3.5121407811 -0.6264714992 -1.6916096304
C 1.4136687243 1.7997425580 0.9621829729
N 1.5782103807 0.3402882433 1.0571694830
H 0.2424430438 2.0005295868 -0.8585135747
H 1.2846313883 3.3812062897 -0.5082030176
H 3.3106576320 2.0131707815 -1.0707499764
H 2.1897856991 2.0679199197 -2.4320315430
H 1.3641015610 -0.2238749822 -1.9180306668
H 1.9662501014 -0.0290485431 -0.1468517222
H 4.3063862883 -0.2979703221 -1.0073028687
H 3.8663181353 -0.3722562869 -2.7056518587
H 0.5732372487 2.1283855458 1.5843056136
H 2.3218944435 2.2470939409 1.3851050951
C 3.3152232868 -2.1426419520 -1.5778275784
H 2.5786609959 -2.4998056356 -2.3080791513
H 4.2536263926 -2.6760266077 -1.7640327352
S 0.2802966298 -0.6056335799 1.5648323709
O -0.0631722601 -0.1314633297 2.9123962215
O 0.6930495042 -1.9884321789 1.3094771945
C -1.1557104242 -0.2858887971 0.5263541463
C -1.3329717255 -1.0222875164 -0.6483503788
C -2.0781279651 0.6951173809 0.8959515658
C -2.4316590010 -0.7555607197 -1.4617561084
H -0.6284388447 -1.8075300227 -0.9018733777
C -3.1710332927 0.9509900669 0.0683003191
H -1.9511304235 1.2291014787 1.8317160998
C -3.3654077530 0.2346402405 -1.1204667716
H -2.5731526377 -1.3321876312 -2.3729102599
H -3.8908981348 1.7131465730 0.3569620737
C -4.5704062658 0.4923727657 -1.9935091086
H -4.3339521707 0.3567462588 -3.0542359641
H -5.3860789009 -0.2018334843 -1.7518421147
H -4.9554012938 1.5079487266 -1.8569601365
H 2.9559700573 -2.4178543634 -0.5803178103
37
-1109.92956266
C 2.4466719983 2.4924422099 -0.3898029541
C 3.6780092013 1.5666506370 -0.3243806186
C 3.4117417132 0.2539436595 -1.0426823679
C 4.1219872903 -1.0228609278 -0.6297473817
C 1.2212779477 1.8157869670 0.2462949452
N 0.9247624485 0.5811665926 -0.4969379387
H 2.2207867253 2.7437221586 -1.4340104901
H 2.6457667790 3.4331410700 0.1363038020
H 3.9282860646 1.3832277065 0.7271046032
H 4.5453389826 2.0763169877 -0.7720731230
H 3.3668483077 0.3752479788 -2.1318297967
H 2.1221600569 0.1389036433 -0.8221055823
H 5.1576101679 -0.9747684204 -1.0095251412
H 3.6499178380 -1.8597413085 -1.1590359132
H 0.3492353944 2.4803906154 0.1680351868
H 1.4041091933 1.6220430265 1.3116449189
C 4.1511124108 -1.3263348116 0.8736122692
H 4.6149307187 -2.3028571967 1.0498789072
H 4.7329076039 -0.5843726720 1.4320738898
S 0.2062097978 -0.6847854114 0.3748929612
O 0.4803746465 -0.5881805415 1.8205339392
O 0.5533075553 -1.9081939160 -0.3588760573
C -1.5368644540 -0.3417391931 0.1310139532
C -2.2844976314 0.2069533463 1.1719006644
C -2.1251786013 -0.6313812692 -1.1036514235
C -3.6394100386 0.4706227706 0.9678891543
H -1.8106069096 0.4075570385 2.1268680556
C -3.4768588457 -0.3612402834 -1.2883415246
H -1.5290378327 -1.0692503691 -1.8972875137
C -4.2553334811 0.1926836114 -0.2585745266
H -4.2275882410 0.8948619006 1.7779425751
H -3.9397607401 -0.5861934811 -2.2463565253
C -5.7273465695 0.4566244508 -0.4682506608
H -6.1318523844 1.1143156244 0.3072272038
H -5.9155076654 0.9228656044 -1.4419379322
H -6.3027198301 -0.4779898624 -0.4419279299
H 3.1423715003 -1.3564497097 1.2985234105
37
-1109.92951648
C 2.4755049547 2.3505100877 -0.6214138044
C 3.6521155038 1.3925627779 -0.3473524985
C 3.3447341050 -0.0095091467 -0.8481347657
C 3.9315261158 -1.2008925593 -0.1206368614
C 1.1930152871 1.8486606237 0.0619645244
N 0.8627013461 0.5138330463 -0.4615782049
H 2.3046311367 2.4316063468 -1.7025434900
H 2.7038736975 3.3567210158 -0.2516721013
H 3.8493881161 1.3636062745 0.7331115153
H 4.5614946592 1.7858218724 -0.8260121965
H 3.3896651112 -0.0952741191 -1.9411141361
H 2.0409783759 -0.0177812733 -0.6934904283
H 3.4164145147 -2.1124899581 -0.4448492038
H 3.7375510465 -1.1027788866 0.9553397785
H 0.3594649445 2.5248367406 -0.1756168857
H 1.3224232945 1.8418963482 1.1521129494
C 5.4468497380 -1.3536026139 -0.3568810382
H 5.8314996265 -2.2327679775 0.1722840720
H 5.6712736573 -1.4783372023 -1.4229375939
S 0.0981816484 -0.5583358801 0.6123596224
O 0.3097005001 -0.1744775745 2.0191631446
O 0.4653185471 -1.9023637060 0.1485498590
C -1.6296868549 -0.2729459265 0.2271074144
C -2.1644676981 -0.7984796194 -0.9528270052
C -2.4196730558 0.4654721909 1.1068442217
C -3.5047121573 -0.5724590868 -1.2472350249
H -1.5365085208 -1.3807798559 -1.6188192206
C -3.7624303033 0.6814343275 0.7947816809
H -1.9874426857 0.8492883342 2.0247964600
C -4.3248060893 0.1692158027 -0.3808150653
H -3.9258280378 -0.9803067492 -2.1632169601
H -4.3833718085 1.2536523194 1.4797140227
C -5.7841657787 0.3843133606 -0.7041128096

H -6.2192393106 1.1773332053 -0.0881720775
H -5.9251171015 0.6550929906 -1.7565923857

H -6.3660761916 -0.5293818412 -0.5257922811
H 5.9998710261 -0.4778634629 0.0021304686

TS 7 → 9

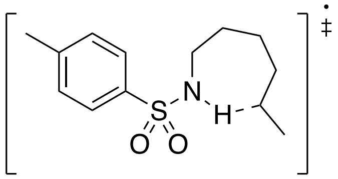

Name	E(B3LYP)	H(B3LYP)	E(RO-B2PLYP-D3)	H(RO-B2PLYP-D3)	NImag
tos_Nhexan_16HAT_ts.conf029	-1110.68356675	-1110.356477	-1110.25880083380000	-1109.93171108380000	-1560.6141
tos_Nhexan_16HAT_ts.conf001	-1110.68585926	-1110.358786	-1110.25866530360000	-1109.93159204360000	-1531.2994
tos_Nhexan_16HAT_ts.conf000	-1110.68585925	-1110.358786	-1110.25866521000000	-1109.93159196000000	-1531.3176
tos_Nhexan_16HAT_ts.conf005	-1110.68439445	-1110.357191	-1110.25792439550000	-1109.93072094550000	-1605.2872
tos_Nhexan_16HAT_ts.conf010	-1110.68439446	-1110.357191	-1110.25792437010000	-1109.93072091010000	-1605.3274
tos_Nhexan_16HAT_ts.conf002	-1110.68439445	-1110.357191	-1110.25792429790000	-1109.93072084790000	-1605.3260
tos_Nhexan_16HAT_ts.conf008	-1110.68439446	-1110.357191	-1110.25792422230000	-1109.93072076230000	-1605.3253
tos_Nhexan_16HAT_ts.conf007	-1110.68439453	-1110.357191	-1110.25792422480000	-1109.93072069480000	-1605.1614
tos_Nhexan_16HAT_ts.conf006	-1110.68439445	-1110.357191	-1110.25792403740000	-1109.93072058740000	-1605.3590
tos_Nhexan_16HAT_ts.conf029	-1110.68356675	-1110.356477	-1110.25880083380000	-1109.93171108380000	-1560.6141

37
-1109.93171108
C -2.7016587199 -0.5521637480 -2.0753735917
C -2.9174427761 -1.8999767996 -1.3593518551
C -3.3046377544 -1.8118467089 0.1287927342
C -2.1969726922 -1.3170186196 1.0416421202
C -1.4349371560 0.2250524047 -1.6908585902
N -1.5524497816 0.7845855377 -0.3379820364
H -2.0131255770 -2.5176303542 -1.4666551159
H -3.7148894238 -2.4373540120 -1.8882850802
H -3.6239764537 -2.8086270969 0.4746236907
H -4.1814715894 -1.1583191837 0.2385548393
H -1.8335811544 -0.2120469465 0.4795619943
H -1.3054431909 1.0755051878 -2.3719887520
H -0.5510791955 -0.4232781978 -1.8066646087
C -2.5785867617 -0.9507004356 2.4588793704
H -1.7473639733 -0.4795453536 2.9920641164
H -2.8798034391 -1.8477359973 3.0220150031
H -2.6407008594 -0.7442254636 -3.1545688685
H -3.5733651530 0.0966110493 -1.9202567956
H -1.2872071264 -1.9269254197 0.9747079006
S -0.2229230866 1.6290902917 0.2692501089
O -0.4924858741 1.8108145430 1.6984695542
O -0.0208921659 2.7724421367 -0.6309844141
C 1.2387178807 0.5869369487 0.1317514843
C 2.0300701109 0.6466666682 -1.0171328540
C 1.5674998628 -0.2802919237 1.1769932625
C 3.1498878296 -0.1780535911 -1.1188483846
H 1.7808947998 1.3477979058 -1.8066500770
C 2.6900029713 -1.0963884231 1.0593579036
H 0.9623987469 -0.2926363526 2.0774362756
C 3.4977787455 -1.0603119194 -0.0871839975
H 3.7679949790 -0.1292699203 -2.0121122647
H 2.9496772322 -1.7668689012 1.8752538384
C 4.7317738677 -1.9248674067 -0.1890600297
H 5.5937466622 -1.4360544003 0.2839549769
H 4.5899211521 -2.8876619840 0.3131895082
H 4.9999609194 -2.1204410250 -1.2321967917
H -3.4224397476 -0.2521593717 2.4718064654
37
-1109.93159204
C -2.4013244132 -2.0595124893 -1.1786178590
C -3.8545555113 -1.6550735267 -0.8635668552
C -4.1720904129 -0.1536141686 -0.9875314326
C -3.5036526832 0.7179293371 0.0591882689
C -1.3428286923 -1.6341322471 -0.1511994903
N -1.0825154241 -0.1865446612 -0.2283487835
H -4.1146089598 -2.0004516317 0.1478812986
H -4.5130599526 -2.2027786802 -1.5499371189
H -5.2631187520 -0.0121285804 -0.9155308415
H -3.8874167006 0.1993962069 -1.9890905091
H -2.2516486283 0.4012010829 -0.0614941804
H -0.3949025803 -2.1421572098 -0.3800173237
H -1.6351522023 -1.9484019692 0.8604448040
C -3.5711067747 2.2165348082 -0.1277644098
H -4.6049860570 2.5728424162 0.0020605703
H -3.2464123279 2.5062789540 -1.1337937091
H -2.3593966230 -3.1546542338 -1.2436112166
H -2.1124307952 -1.6758230071 -2.1660663637
H -3.7212149565 0.3967054686 1.0849583254
S -0.1101426747 0.4338862953 1.0262109307
O -0.1879790013 -0.3897618344 2.2447429629
O -0.4094202405 1.8704985203 1.0835741163
C 1.5266291310 0.2175917575 0.3258198987
C 2.3516950118 -0.8001638584 0.8024817366
C 1.9573489589 1.0787762558 -0.6878855431
C 3.6239064448 -0.9562849341 0.2508324101
H 2.0024729583 -1.4448221547 1.6020735301
C 3.2283882607 0.9076157576 -1.2255024851
H 1.3047658037 1.8719535489 -1.0370789341
C 4.0814164183 -0.1098605566 -0.7665239067
H 4.2724319867 -1.7461720142 0.6217360561
H 3.5689754508 1.5756486785 -2.0131406325
C 5.4678659387 -0.2657144789 -1.3443004899
H 5.9033450969 -1.2361411468 -1.0871172970
H 5.4587344552 -0.1754398451 -2.4363519957
H 6.1431084664 0.5110751010 -0.9623382622
H -2.9375562818 2.7330753438 0.5983687103
37
-1109.93072095
C -3.4587572622 0.8295861290 -1.1179863278
C -4.1988788797 -0.3471102904 -0.4565008198
C -3.4717831028 -1.0134159293 0.7272753912
C -2.2391004822 -1.8062832335 0.3413257383
C -2.1852884677 0.4697435721 -1.9049319307
N -1.0267377653 -0.0319465411 -1.1364836367
H -4.4293926911 -1.1071036855 -1.2189252765
H -5.1671769585 0.0239794201 -0.0973150988
H -4.1739755419 -1.6944880228 1.2363282786
H -3.1908897321 -0.2494603766 1.4632208289
H -1.5057352739 -0.9827324553 -0.3605845140
H -1.8383613451 1.3462863472 -2.4625068985
H -2.4095369352 -0.3163624216 -2.6371287206
C -1.3618894614 -2.3036065519 1.4689103259

H -0.4593998653 -2.7988042475 1.0950243488
H -1.9095728394 -3.0339932036 2.0846392314
H -4.1433708731 1.3050973808 -1.8334504404
H -3.2216625398 1.5903974390 -0.3644905148
H -2.4410418877 -2.5680076918 -0.4232588873
S -0.2718536241 1.1236338535 -0.1494138222
O -0.9015283220 1.2235049832 1.1814479818
O -0.1184732291 2.3410979765 -0.9625539179
C 1.3343175458 0.3588370251 0.0777269940
C 2.1032153430 0.0013818763 -1.0340684836
C 1.8308223672 0.2044913977 1.3698529366
C 3.3728531034 -0.5298136409 -0.8370661239
H 1.7046644213 0.1299891912 -2.0346748021
C 3.1091976886 -0.3275697766 1.5487047504
H 1.2210357089 0.4986748749 2.2171544200
C 3.8967884202 -0.7035637486 0.4551742274
H 3.9715813719 -0.8136721591 -1.6994658920
H 3.4988473364 -0.4495849800 2.5562739365
C 5.2751514411 -1.2894352500 0.6490547897
H 5.3121089265 -2.3348115897 0.3172041738
H 6.0259942710 -0.7414527493 0.0675282128
H 5.5779703357 -1.2628913350 1.7001071894
H -1.0614393013 -1.4786715261 2.1232608053
37
-1109.93035554
C -2.4744075854 -1.3522510504 -1.8510871151
C -3.8959650914 -1.1630732286 -1.2869483147
C -4.0501380144 -1.4143749344 0.2266810005
C -3.3472963626 -0.4098287786 1.1277992134
C -1.4472594927 -0.2744140352 -1.4744698744
N -1.0351757683 -0.4040965423 -0.0674219989
H -4.5623233052 -1.8593009614 -1.8122397826
H -4.2545183238 -0.1557361088 -1.5400388117
H -3.6803278420 -2.4225261870 0.4557239979
H -5.1237976661 -1.4171956753 0.4762929622
H -2.1446817704 -0.3853102595 0.6508565937
H -1.8352197040 0.7279924457 -1.7029147242
H -0.5432270790 -0.4110718246 -2.0855823496
C -3.8644525678 1.0124194739 1.1603278335
H -4.8722262725 1.0392194995 1.6027713783
H -3.9358425246 1.4560543270 0.1610772878
H -2.0830064131 -2.3351356865 -1.5567903726
H -2.5410688765 -1.3570091641 -2.9469102713
H -3.1356724623 -0.8028632027 2.1275277819
S -0.1229008473 0.8995844235 0.5414382030
O -0.3300660659 0.8806577967 1.9950852986
O -0.3505658846 2.1371856883 -0.2243438313
C 1.5455952966 0.3427943247 0.1913432615
C 2.2586292707 0.9202471453 -0.8582570950
C 2.1127871218 -0.6621824079 0.9806260313
C 3.5566515737 0.4796891069 -1.1196463487
H 1.8041748148 1.7108258909 -1.4458817873
C 3.4075224505 -1.0882004806 0.7049790009
H 1.5458128587 -1.0909601284 1.8001725735
C 4.1499230764 -0.5260607929 -0.3469173634
H 4.1181532457 0.9295450610 -1.9347721327
H 3.8541229601 -1.8684056520 1.3169268298
C 5.5634927727 -0.9836736956 -0.6167202286
H 5.9116272211 -0.6535364925 -1.6002437757
H 6.2576428571 -0.5803144900 0.1320702356
H 5.6451832688 -2.0756928066 -0.5757672963
H -3.2141242210 1.6485715422 1.7680431929
37
-1109.93025671
C -3.7368358708 0.0993209961 1.0198344742
C -4.2155601277 -0.8980124514 -0.0519966349
C -3.1955551631 -1.9730412686 -0.4673823103
C -1.9874904861 -1.4396967809 -1.2128848864
C -2.6592791407 1.0986801147 0.5802938186
N -1.3407160554 0.4357882245 0.4695075167
H -4.5391997911 -0.3410499162 -0.9435745963
H -5.1108646733 -1.4041103654 0.3314043190
H -3.7010149509 -2.7156326770 -1.1072722792
H -2.8593465774 -2.5237204633 0.4232034607
H -1.5157049493 -0.5255800141 -0.3851166324
H -2.5513123945 1.8865479353 1.3337064160
H -2.9585381863 1.5879697673 -0.3592191986
C -0.8594805404 -2.4101327777 -1.4800667618
H -1.1941225527 -3.2105522725 -2.1585347715
H -0.5149289157 -2.8888997747 -0.5559465026
H -4.6034318308 0.6881146525 1.3478218258
H -3.3750397412 -0.4438633005 1.9030949460
H -2.2473099588 -0.8198284497 -2.0783701919
S -0.1514468752 1.4641932071 -0.1798878392
O -0.1207650738 2.6572084721 0.6801234987
O -0.3016611029 1.6248610305 -1.6366587435
C 1.3228713393 0.4888354010 0.1124324274
C 2.1399711026 0.1572941256 -0.9661409159
C 1.6735400484 0.1294215218 1.4171016885
C 3.3172982952 -0.5558608447 -0.7332615536
H 1.8518142703 0.4575935901 -1.9679302024
C 2.8477842353 -0.5846437851 1.6298036275
H 1.0298167372 0.4001757076 2.2472423145
C 3.6892818453 -0.9365315528 0.5611484418
H 3.9565898581 -0.8176764133 -1.5727475835
H 3.1207674952 -0.8727116170 2.6423285183
C 4.9724624878 -1.6916202945 0.8140881707
H 5.4088053080 -2.0649532889 -0.1173284763
H 5.7199226011 -1.0482170604 1.2959079492
H 4.8085321408 -2.5473155841 1.4791498904
H -0.0036714373 -1.9162477164 -1.9504419938
37
-1109.92977047
C -2.5816685597 -0.2446784111 -2.0244228182
C -3.9401558869 -0.6131235404 -1.4014794652
C -4.1618066958 -0.1330011525 0.0456314477
C -3.3178511783 -0.8539879165 1.0787820524
C -1.3526954844 -0.9838393224 -1.4599150781
N -0.9753754360 -0.7188068542 -0.0596433206
H -4.0849162868 -1.7033389410 -1.4491724336
H -4.7258268382 -0.1759963901 -2.0307948123
H -5.2231742238 -0.2736043486 0.3081990029
H -3.9746312248 0.9480710715 0.1086500987
H -2.1029707227 -0.7649806838 0.6568816851
H -0.4794897052 -0.7577756536 -2.0858613642
H -1.5121419262 -2.0682383076 -1.5210246950
C -3.3353329090 -0.3114497733 2.4906477914
H -3.0368724201 0.7404000480 2.5173942116
H -2.6571259020 -0.8690488047 3.1439599825
H -2.6213137733 -0.4863042284 -3.0955101693
H -2.4140384709 0.8348408079 -1.9519615085
H -3.4439200156 -1.9441354009 1.0323662376
S -0.2243908353 0.7704554879 0.2631764735
O -0.5016766102 1.0550628718 1.6771762605
O -0.4981953031 1.7936990846 -0.7615946149
C 1.5002416986 0.3020901473 0.0955814226
C 2.2461479440 0.7945869629 -0.9735903883
C 2.0812299521 -0.5363998367 1.0526468353
C 3.5910106393 0.4371232979 -1.0850344383
H 1.7781538712 1.4565143610 -1.6943319744
C 3.4216910929 -0.8812394963 0.9254011672
H 1.4873626062 -0.9040787157 1.8827515574
C 4.1981807743 -0.4017267191 -0.1434733396
H 4.1773300014 0.8217056741 -1.9158963384
H 3.8780841302 -1.5319260465 1.6677454589
C 5.6571990543 -0.7744348083 -0.2559127303
H 6.2477704672 -0.3020394341 0.5395048499
H 5.8010818781 -1.8572225671 -0.1625515753
H 6.0794934900 -0.4584488733 -1.2145686042
H -4.3497984857 -0.3918678389 2.9118723272
37
-1109.92909228
C -3.3703244376 -0.2678985280 -1.7404790806
C -3.9651811275 -1.2518248442 -0.7152658525
C -2.9585899201 -2.2043283586 -0.0392274380
C -1.9645805701 -1.5414957425 0.9034463342
C -2.4646961037 0.8366366793 -1.1792808691
N -1.1570847500 0.2785350423 -0.7751668713
H -4.7133027271 -1.8659432214 -1.2329280557
H -4.5183158216 -0.6878491477 0.0482039682

H -2.4069533488 -2.7486484077 -0.8170557701
H -3.5205304476 -2.9655203590 0.5270158991
H -1.4297993853 -0.6134021878 0.1315913688
H -2.9672107149 1.3613245485 -0.3522231217
H -2.2622345309 1.5814923484 -1.9570650924
C -2.4954779320 -0.8772947384 2.1547930971
H -1.7116151996 -0.3174904482 2.6723471557
H -2.8878460340 -1.6370317727 2.8490110117
H -2.8118604579 -0.8202162388 -2.5078935964
H -4.2022252101 0.2302946377 -2.2555144620
H -1.0812377183 -2.1615773088 1.0927745071
S -0.1308420421 1.4296999392 -0.0625792516
O -0.5517928303 1.7773443868 1.3065634782
O 0.0450343582 2.4994278210 -1.0567800191
C 1.3823867123 0.4780105662 0.0541526096
C 1.9073919559 0.1800586288 1.3093712773
C 2.0423012756 0.0844426488 -1.1138222445
C 3.1042172438 -0.5338096350 1.3932154810
H 1.3854221638 0.5101492240 2.2010578182
C 3.2316777830 -0.6284151251 -1.0112103955
H 1.6252730619 0.3341432856 -2.0837023173
C 3.7825079956 -0.9479668368 0.2413119845
H 3.5172817547 -0.7685254136 2.3711022819
H 3.7464680426 -0.9405481282 -1.9168911818
C 5.0862394614 -1.7046841251 0.3329041666
H 5.0795461357 -2.5916705843 -0.3111556631
H 5.9286793100 -1.0794506120 0.0103759836
H 5.2896117058 -2.0314404459 1.3571798814
H -3.3116540990 -0.1790302360 1.9418389421
37
-1109.92887987
C -3.1218441193 -0.3244341701 1.6032428739
C -3.4574196781 -1.6107371868 0.8254929835
C -3.5745971955 -1.4547857457 -0.7044574019
C -2.2659090757 -1.1928907212 -1.4383779760
C -1.7164933698 0.2562529302 1.3895456239
N -1.5919316780 0.8274114376 0.0439671629
H -4.4183511779 -1.9860609297 1.2000613848
H -2.7190216492 -2.3873707639 1.0694197087
H -4.2769899069 -0.6412127957 -0.9260140636
H -4.0249232122 -2.3715847481 -1.1187832392
H -1.8083379541 -0.1508301257 -0.8249852420
H -0.9520066663 -0.5109982161 1.5966283798
H -1.5523184912 1.0778892562 2.0977477009
C -1.2233526214 -2.2910889096 -1.4250018517
H -0.3067036588 -1.9829941099 -1.9366485496
H -1.6074604751 -3.1828733389 -1.9440367927
H -3.8566277648 0.4554205681 1.3659028168
H -3.2220502570 -0.5371815668 2.6758726027
H -2.4182975220 -0.7659411551 -2.4353451306
S -0.1802073511 1.6669140558 -0.3356682909
O -0.1048386280 2.7731637905 0.6291722533
O -0.2402524455 1.9041004728 -1.7797097211
C 1.2470780174 0.6134558412 -0.0314801518
C 1.7890009155 0.5232753356 1.2530285529
C 1.8109503790 -0.0998528982 -1.0917901437
C 2.8887930878 -0.3042799000 1.4735894001
H 1.3698751726 1.1147818107 2.0601837212
C 2.9132818280 -0.9185275437 -0.8547637971
H 1.4000457739 0.0115244923 -2.0896458024
C 3.4676515236 -1.0370185747 0.4279332310
H 3.3113653640 -0.3736810922 2.4731276685
H 3.3566866998 -1.4684460554 -1.6815562262
C 4.6824033818 -1.9014879160 0.6682383146
H 5.6074931831 -1.3327909287 0.5053664856
H 4.7096213269 -2.2791329751 1.6955266561
H 4.7051984278 -2.7595456370 -0.0115879370
H -0.9526636495 -2.6012541029 -0.4096341604
37
-1109.92840248
C -2.6622014649 -1.6587838042 0.9487547931
C -3.9922987617 -1.2477346969 0.2913110233
C -4.1464427191 0.2604112490 -0.0001820639
C -3.2500214981 0.8023942771 -1.1024114723
C -1.4170026223 -1.6322030085 0.0403064577
N -0.9817432037 -0.3253961833 -0.4829570830
H -4.8047914895 -1.5411155414 0.9681532111
H -4.1451087890 -1.8290643919 -0.6295963789
H -3.9561583086 0.8182818346 0.9254768231
H -5.1953213020 0.4611658211 -0.2722085024
H -2.0803116323 0.3312053351 -0.8628056432
H -1.5904976349 -2.2607568198 -0.8427272592
H -0.5708130095 -2.0737103170 0.5825034034
C -3.5590376022 0.3894174367 -2.5275268045
H -3.6454286425 -0.6974620468 -2.6372447397
H -2.7868321367 0.7398220720 -3.2202319334
H -2.4724753545 -1.0419763254 1.8336324465
H -2.7611811678 -2.6945925849 1.3014047359
H -3.0425576367 1.8717271727 -1.0030769640
S -0.1747122287 0.7052541766 0.6038205016
O -0.4196413059 0.3568979047 2.0146506659
O -0.4347997245 2.0683888479 0.1241244329
C 1.5318537371 0.2813746969 0.2427279226
C 2.0941301720 0.6710576153 -0.9770853927
C 2.2827186704 -0.4136877632 1.1888744515
C 3.4207241671 0.3521093030 -1.2430434145
H 1.4973911581 1.2196589028 -1.6981365420
C 3.6132666896 -0.7253897852 0.9040354426
H 1.8302330743 -0.6904554511 2.1350656289
C 4.2016067697 -0.3502410575 -0.3092751265
H 3.8626091744 0.6539390033 -2.1898217438
H 4.2034001309 -1.2649820013 1.6406633943
C 5.6464958304 -0.6716204075 -0.6081979723
H 6.2652871759 0.2346803175 -0.5848693661
H 6.0607549840 -1.3740076881 0.1214149227
H 5.7597504240 -1.1121849557 -1.6055183512
H -4.5165468097 0.8265397792 -2.8496194759
37
-1109.92836698
C -1.4877340022 -1.7972557097 1.0821555538
C -2.3933447108 -2.3267175670 -0.0461444298
C -2.2290772707 -1.6348247595 -1.4135207017
C -2.7340691926 -0.2049664587 -1.4555420574
C -1.8550498720 -0.4155620356 1.6593256245
N -1.7509214193 0.7416338655 0.7589524316
H -3.4454503627 -2.2667149235 0.2704058323
H -2.1768743611 -3.3946238416 -0.1766011500
H -2.7702900107 -2.2199639856 -2.1747512259
H -1.1704534243 -1.6595555476 -1.7094113295
H -2.2058165322 0.3840091101 -0.4473231346
H -1.2407511372 -0.2039063792 2.5427866023
H -2.8978204005 -0.4384205633 2.0024574862
C -2.4078189629 0.6194406462 -2.6817495018
H -1.3421719268 0.5698604667 -2.9297654206
H -2.6710940115 1.6716833994 -2.5393423316
H -1.5423567667 -2.5033423943 1.9218762734
H -0.4435513819 -1.7984942837 0.7477137747
H -3.7892837625 -0.1273701642 -1.1623371791
S -0.3157068647 1.6090469441 0.6561346270
O -0.0667155834 2.1150670840 2.0139963912
O -0.4943463255 2.5096883954 -0.4861554648
C 1.0748206395 0.5339984951 0.2592817876
C 1.3947030359 0.2832040759 -1.0770102246
C 1.8242237284 -0.0400644934 1.2884982731
C 2.4576165110 -0.5661102725 -1.3775912152
H 0.8312524282 0.7705898980 -1.8654929439
C 2.8838247085 -0.8882633886 0.9704546630
H 1.5888200473 0.1941254572 2.3212690577
C 3.2164430764 -1.1672696690 -0.3626326524
H 2.7093545738 -0.7578471323 -2.4180202235
H 3.4685280012 -1.3338092612 1.7717837557
C 4.3875940103 -2.0596565973 -0.6981905489
H 4.5652180051 -2.8012653857 0.0873780269
H 4.2288200011 -2.5928081867 -1.6413340752
H 5.3095208074 -1.4732335546 -0.8072857948
H -2.9666939454 0.2446813034 -3.5531773536

TS 7 → 10

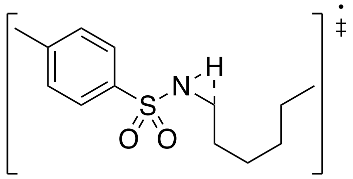

Name	E(B3LYP)	H(B3LYP)	E(RO-B2PLYP-D3)	H(RO-B2PLYP-D3)	NImag
tos_Nhexan_nrad_12HAT_ts.conf103	-1110.64040532	-1110.312512	-1110.21480592950000	-1109.88691260950000	-1998.1093
tos_Nhexan_nrad_12HAT_ts.conf099	-1110.64040528	-1110.312513	-1110.21480347210000	-1109.88691119210000	-1998.1306
tos_Nhexan_nrad_12HAT_ts.conf041	-1110.63977388	-1110.311960	-1110.21441413100000	-1109.88660025100000	-1999.6791
tos_Nhexan_nrad_12HAT_ts.conf120	-1110.64052033	-1110.312607	-1110.21380694010000	-1109.88589361010000	-2002.7381
tos_Nhexan_nrad_12HAT_ts.conf131	-1110.64052033	-1110.312607	-1110.21380630910000	-1109.88589297910000	-2002.7286
tos_Nhexan_nrad_12HAT_ts.conf245	-1110.64051724	-1110.312480	-1110.21387369140000	-1109.88583645140000	-1993.2617

37
-1109.88806594
S 0.4461664928 -1.9767465079 -0.0767052365
N 1.5714524206 -0.8200260205 -0.7025760179
C -1.0190832384 -0.9588602792 0.0266135845
C -1.4291269363 -0.4707648205 1.2676977646
H -0.8652792683 -0.7229695885 2.1593672609
C -2.5729101902 0.3238506419 1.3370501783
H -2.8993481176 0.7041618883 2.3017658915
C -3.3113855959 0.6349925397 0.1874953659
C -4.5665140678 1.4699652782 0.2733745700
C -2.8744910073 0.1267082582 -1.0468447360
H -3.4377339171 0.3551347928 -1.9483995952
C -1.7376154698 -0.6703696369 -1.1369480761
H -1.4114970516 -1.0723483191 -2.0903160263
O 0.2545889267 -2.9790203027 -1.1295812869
O 0.8250864160 -2.3830643717 1.2862899286
C 2.7882354820 -0.6512101437 0.0822570046
C 3.5788225943 0.6071943811 -0.1314457038
C 3.1798224388 1.7676296057 0.8155276414
H 3.4833998596 0.9242135903 -1.1760770615
H 4.6389380882 0.3772277616 0.0378545404
H -4.5844197097 2.0752323994 1.1849937817
H -5.4611817056 0.8334928357 0.2836865733
H -4.6590345688 2.1435528209 -0.5853193992
H 2.6300017490 -1.3875920873 -0.9970670985
H 2.8448979116 -1.1872965687 1.0293553504
C 1.7509233760 2.3087589557 0.6498363685
C 1.4501869737 2.9262995723 -0.7226766840
C 0.0552999390 3.5568567276 -0.7947400614
H 3.8971809496 2.5852802529 0.6553111414
H 3.3213839020 1.4354839188 1.8528955335
H 1.5877796471 3.0714571491 1.4251259803
H 1.0224171914 1.5126673229 0.8528758452
H 1.5346637523 2.1569191522 -1.5000585702
H 2.2096870186 3.6883407808 -0.9521374753
H -0.7247251353 2.8095830234 -0.6057968823
H -0.1340428367 3.9942118692 -1.7818490162
H -0.0595660184 4.3540997924 -0.0495232216
37
-1109.88698775
S 0.3099188868 -1.2743453912 0.2932857580
N 1.0736589706 -0.1751252686 -0.7874984345
C -1.4075561700 -0.8605776933 0.0260588586
C -1.9316750494 0.2995518451 0.6007070275
H -1.2987047314 0.9417012352 1.2039504665
C -3.2720733766 0.6088895362 0.3913201776
H -3.6857597287 1.5124219927 0.8325615432
C -4.1001413491 -0.2276623920 -0.3738089561
C -5.5605120805 0.1052007738 -0.5653061813
C -3.5469796712 -1.3880922252 -0.9305426756
H -4.1747323133 -2.0492797257 -1.5226557622
C -2.2050276242 -1.7132921816 -0.7355570295
H -1.7787907480 -2.6172372495 -1.1573674711
O 0.5348281040 -2.6622470719 -0.1410768624
O 0.6994166652 -0.8584027980 1.6480364701
C 2.4597331932 -0.5376660325 -1.0864214962
C 3.3827593263 0.5720445262 -1.4929896749
C 4.1779941725 1.1752187644 -0.3060482687
H 2.8040841129 1.3535877019 -1.9982185413
H 4.1025410972 0.1799749513 -2.2237998235
H -6.1486457525 -0.1886780307 0.3140083605
H -5.9811259374 -0.4164538529 -1.4305448686
H -5.7104050822 1.1806843308 -0.7092740946
H 1.3969993479 -0.7278290734 -1.8414483016
H 2.8604938059 -1.4443143762 -0.6339406103
C 3.3355345215 1.8075745716 0.8132127013
C 2.4883749398 3.0119962159 0.3802722311
C 1.7554990190 3.6663600932 1.5563743423
H 4.8661111452 1.9271396271 -0.7186176963
H 4.8090227045 0.3857870851 0.1240762879
H 4.0227556775 2.1267029181 1.6101728957
H 2.6821173739 1.0489289840 1.2630683558
H 1.7513437299 2.6950472131 -0.3681632304
H 3.1332537587 3.7562306057 -0.1105337989
H 1.0896734292 2.9484402742 2.0508367586
H 1.1475793227 4.5172482812 1.2274326648
H 2.4621363838 4.0331685083 2.3112574599
37
-1109.88691261
S 0.4165024889 -2.0827645285 0.1547885477
N 1.5048357536 -1.2012263519 -0.8600984080
C -1.0377993296 -1.0518625130 0.0200628232
C -1.3922531196 -0.2228605166 1.0844817049
H -0.7926551580 -0.2202582447 1.9883982009
C -2.5306197027 0.5752461239 0.9700070373
H -2.8160535540 1.2180030421 1.7991001509
C -3.3165572557 0.5559804100 -0.1895733664
C -4.5621134038 1.4024107574 -0.2987529626
C -2.9350751983 -0.2908862468 -1.2430973803
H -3.5360272293 -0.3212244259 -2.1487893139
C -1.8056290995 -1.0969485507 -1.1474743875
H -1.5221498255 -1.7596181867 -1.9583240016
O 0.1653934408 -3.3547940248 -0.5268931995
O 0.8716998948 -2.0570849335 1.5547849676
C 2.6629571406 -0.6300985879 -0.1860135364
C 3.3234692599 0.5308576023 -0.8726396984
C 2.5358624148 1.8562382592 -0.7329612850
H 3.4372268036 0.3037826568 -1.9398574391
H 4.3317308855 0.6530712442 -0.4560879441
H -4.5672007800 2.2069631024 0.4429634110
H -5.4638134157 0.7976086184 -0.1358563074
H -4.6526013337 1.8545819429 -1.2925591580
H 2.6171905494 -1.7473286053 -0.8744970322
H 2.7197835305 -0.7578196986 0.8939066670
C 2.4113036421 2.3643549512 0.7098643364

C 1.7736561647 3.7583857854 0.8342379631
C 0.3216525894 3.8392230582 0.3474317836
H 1.5429987100 1.7121117128 -1.1754052676
H 3.0447052989 2.6164393694 -1.3425386132
H 3.4120635543 2.3928889013 1.1651395922
H 1.8257719571 1.6488685793 1.3049611490
H 2.3850293986 4.4852628304 0.2802403015
H 1.8160201675 4.0670713594 1.8873626364
H -0.1000462497 4.8332001879 0.5359603307
H -0.3105605622 3.1041093569 0.8609223127
H 0.2401454636 3.6469695547 -0.7281438614
37
-1109.88672778
S 0.2956522799 -1.5205021038 0.5997762790
N 1.1136724337 -0.9824272501 -0.8246557401
C -1.3783252250 -1.0530872999 0.1880441150
C -2.1654645268 -1.9212653577 -0.5707417513
H -1.7642385483 -2.8760831522 -0.8934071815
C -3.4708570058 -1.5500455503 -0.8818975760
H -4.0912027631 -2.2243430645 -1.4672305887
C -4.0026993809 -0.3277649390 -0.4443538952
C -5.4105871724 0.0757316774 -0.8119156556
C -3.1919327941 0.5159925606 0.3278728028
H -3.5935440554 1.4591402115 0.6900598913
C -1.8822147738 0.1641716783 0.6472891323
H -1.2601300975 0.8104940300 1.2570524842
O 0.3772186992 -2.9866647816 0.6562762708
O 0.7440936856 -0.7057009847 1.7397237167
C 2.5132635911 -0.6449475406 -0.6033287356
C 3.1199109438 0.3160628258 -1.5849308588
C 2.6682019397 1.7782957548 -1.3520866961
H 2.8423905631 0.0204893865 -2.6043165642
H 4.2130005238 0.2480490758 -1.5111609339
H -5.4325526651 0.5787215516 -1.7878606052
H -5.8339339536 0.7690494331 -0.0781949856
H -6.0720633460 -0.7941697917 -0.8806853884
H 2.0307183209 -1.7599164172 -1.1135197333
H 2.8836946816 -0.6878936526 0.4193005952
C 3.0679012960 2.3574254414 0.0109701903
C 2.6765800757 3.8321835457 0.1738138355
C 3.0687332547 4.4121868842 1.5365390495
H 1.5775883062 1.8257919475 -1.4697859035
H 3.0968664494 2.3963594913 -2.1532922478
H 4.1553592634 2.2547418571 0.1475510635
H 2.6000056587 1.7717012743 0.8144189317
H 1.5915651196 3.9353140507 0.0302665449
H 3.1462233061 4.4230276074 -0.6257062812
H 2.7751077418 5.4645933977 1.6228422739
H 4.1528268937 4.3549908549 1.6944223709
H 2.5867700134 3.8625816056 2.3541717001
37
-1109.88660025
S 0.4261915127 -2.0707765956 0.1254089520
N 1.5563334283 -1.1086967057 -0.7646008317
C -1.0234229549 -1.0301053671 0.0200152172
C -1.3968734415 -0.2598698848 1.1219527134
H -0.8163932417 -0.3103373218 2.0368606233
C -2.5286972658 0.5498322517 1.0283132006
H -2.8283693342 1.1474122045 1.8856876785
C -3.2904945697 0.5990466323 -0.1466588053
C -4.5304004040 1.4563267065 -0.2333659245
C -2.8907392657 -0.1899102720 -1.2373864062
H -3.4727079660 -0.1667381119 -2.1555884647
C -1.7664891571 -1.0058850454 -1.1637402794
H -1.4684050262 -1.6238496844 -2.0040658691
O 0.2022262875 -3.2797662755 -0.6719143273
O 0.8233325559 -2.1661670753 1.5398371431
C 2.7309027723 -0.6801013031 -0.0159244944
C 3.5008441828 0.4984447803 -0.5383765599
C 2.9867226777 1.8651964047 -0.0200120475
H 3.4809278629 0.4936299844 -1.6355071255
H 4.5480243983 0.3776757526 -0.2320114238
H -4.5362917851 2.2331507790 0.5374279952
H -5.4365891548 0.8513110524 -0.0982785678
H -4.6116307412 1.9448808324 -1.2105180549
H 2.6407723557 -1.6987255641 -0.8433167025
H 2.7605527503 -0.9318408242 1.0440287163
C 1.6380867312 2.2949612668 -0.6130004226
C 1.1606522378 3.6807835239 -0.1461231341
C 0.7745650464 3.7510893105 1.3365024832
H 3.7444829187 2.6232108170 -0.2657787760
H 2.9330185854 1.8306623676 1.0760455578
H 0.8683030897 1.5496781878 -0.3760136329
H 1.7254410362 2.2986339182 -1.7085152029
H 0.2917137660 3.9707548134 -0.7517529197
H 1.9410169980 4.4259327045 -0.3586513886
H -0.0116749668 3.0221512507 1.5698069030
H 0.3957543606 4.7457433608 1.5983537377
H 1.6262263510 3.5424992845 1.9939979849
37
-1109.88598521
S 0.2975065654 -2.1792995578 0.1459477391
N 1.3171064155 -1.1346499342 -0.7830837816
C -1.2367764639 -1.2677121970 0.0524137356
C -1.6292278772 -0.4706496826 1.1281470240
H -1.0112284459 -0.4229686821 2.0183245846
C -2.8259121377 0.2394410697 1.0416595080
H -3.1389714691 0.8593858396 1.8781266569
C -3.6343881489 0.1629545266 -0.1001779277
C -4.9435643223 0.9114817009 -0.1761657288
C -3.2135266025 -0.6492610718 -1.1653845889
H -3.8304904427 -0.7228397795 -2.0577317659
C -2.0237242182 -1.3671116714 -1.0983581744
H -1.7079169565 -2.0041721885 -1.9176463865
O 0.1614990872 -3.4198807121 -0.6229912929
O 0.7333001370 -2.2107242352 1.5515715354
C 2.5046874332 -0.6488662984 -0.0945859046
C 3.0976664946 0.6266700528 -0.6162969820
C 2.3474922256 1.8736628987 -0.0960888146
H 3.0720146921 0.6226084125 -1.7133408507
H 4.1519994575 0.6775931527 -0.3145861917
H -5.1144351583 1.3187618355 -1.1786056506
H -4.9744954287 1.7400740251 0.5381662416
H -5.7888143576 0.2488678321 0.0520347438
H 2.4314239925 -1.6467690888 -0.9489610621
H 2.6177535355 -0.9263172180 0.9532100380
C 2.9360352519 3.1813356162 -0.6437325965
C 2.1834490109 4.4497806761 -0.2077458076
C 2.2563595534 4.7482464246 1.2945478245
H 2.3744227341 1.8747651959 1.0012328122
H 1.2910431421 1.7927472841 -0.3852197760
H 2.9401371528 3.1323635824 -1.7418207495
H 3.9898848477 3.2627589657 -0.3377177610
H 1.1311248874 4.3678316636 -0.5149506063
H 2.5949762507 5.3049693801 -0.7604259024
H 1.7823140037 3.9620240531 1.8928059085
H 1.7499908309 5.6906076981 1.5324723645
H 3.2977127253 4.8373395733 1.6289304104
37
-1109.88589361
S 0.2733209831 -1.5907706493 0.2288061245
N 1.0031560452 -0.7038208414 -1.0653375468
C -1.4228939892 -1.0633317553 0.0418336836
C -1.9149047466 -0.0374151829 0.8492381575
H -1.2692211065 0.4251878778 1.5878903125
C -3.2402816612 0.3650702852 0.6964593593
H -3.6304391581 1.1625087725 1.3240698982
C -4.0793494188 -0.2424865572 -0.2477770441
C -5.5227563862 0.1788606765 -0.3859768307
C -3.5558586319 -1.2733047420 -1.0432025731
H -4.1936850429 -1.7578709615 -1.7784431094
C -2.2357856037 -1.6921758146 -0.9038889184
H -1.8393351247 -2.5002042206 -1.5094438898
O 0.3639213160 -3.0194408725 -0.0989005025
O 0.7804021319 -1.0859087793 1.5148602454
C 2.3435023705 -0.2153510366 -0.7734406042
C 2.8002913800 0.9724531593 -1.5703168775
C 2.1415686740 2.2952663959 -1.1029508546
H 2.5537452707 0.8203627863 -2.6283077805
H 3.8922339546 1.0417883019 -1.5005380191

H -5.8626049224 0.1102910856 -1.4247732774
H -5.6751281579 1.2075408190 -0.0442923838
H -6.1790546197 -0.4659178691 0.2134629469
H 2.0176811360 -1.2853140668 -1.4687499283
H 2.7243958627 -0.3826661257 0.2326196971
C 2.4016730232 2.6870138127 0.3607559741
C 3.8722383227 2.9649677372 0.7034972729
C 4.0657280267 3.4429292104 2.1465080696
H 1.0601593876 2.2059821031 -1.2652594241
H 2.4930582802 3.0992948711 -1.7645961394
H 2.0127930277 1.9058253323 1.0293433627
H 1.8135853368 3.5894005714 0.5805591545
H 4.2686187721 3.7206537594 0.0096774415
H 4.4737912560 2.0597714708 0.5429542727
H 3.5131107768 4.3720527335 2.3329731917
H 5.1221863980 3.6332994502 2.3671550264
H 3.7048961840 2.6946100883 2.8625664481
37
-1109.88583645
S -0.0956911314 -0.4683989310 -0.3583466384
N 0.6582349188 -0.7432531182 1.1560859881
C -1.7776491930 -0.9095588391 0.0589175570
C -2.5956055410 0.0282538360 0.6921441697
H -2.2102053906 1.0152462266 0.9247365861
C -3.9075960618 -0.3197906020 0.9995249850
H -4.5511932466 0.4072775014 1.4888110399
C -4.4165629907 -1.5885053855 0.6808474052
C -5.8345123026 -1.9604150896 1.0434010715
C -3.5754544164 -2.5025814426 0.0328491152
H -3.9579361194 -3.4840579850 -0.2360108304
C -2.2574781204 -2.1732327004 -0.2813176026
H -1.6116016782 -2.8754213113 -0.7975894404
O -0.0454479320 0.9744969609 -0.6363213093
O 0.4032799307 -1.4279554045 -1.3584126125
C 2.1158013717 -0.6370063200 1.2071782851
C 3.0184460925 -0.0099863161 0.1840268020
C 3.1393007974 1.5245965664 0.3385906717
H 2.6803560602 -0.2567611392 -0.8274980552
H 4.0132659322 -0.4609911224 0.3054832022
H -5.9059621491 -2.2538614579 2.0990727660
H -6.1945923759 -2.8022415800 0.4439169341
H -6.5196432837 -1.1187311512 0.8952124060
H 1.3920910077 -1.7357422533 1.0893445607
H 2.4333403863 -0.6240196813 2.2492698958
C 4.1068220207 2.1361807223 -0.6826196242
C 4.2498086123 3.6574327029 -0.5439374956
C 5.2111599150 4.2674917590 -1.5692091068
H 3.4810284678 1.7602966803 1.3566817866
H 2.1455375372 1.9700285713 0.2208633954
H 3.7584504157 1.8959723486 -1.6974724669
H 5.0973168794 1.6674020290 -0.5791791758
H 4.5960635952 3.8981120741 0.4714549770
H 3.2598368201 4.1229049029 -0.6468310204
H 4.8717661890 4.0737928436 -2.5941166081
H 6.2187168800 3.8448634925 -1.4694373474
H 5.2919421742 5.3534583831 -1.4449313084
37
-1109.88582749
S 0.1724309009 -2.0447279564 -0.1775488604
N 1.0570883056 -0.5648567800 -0.3263595224
C -1.4729198527 -1.3924923477 0.0677045763
C -2.2054191075 -0.9480457052 -1.0367977730
H -1.7795434263 -1.0147520064 -2.0322913968
C -3.4859480954 -0.4429596006 -0.8371741736
H -4.0633842937 -0.1018577118 -1.6931054189
C -4.0495211350 -0.3751868778 0.4473655226
C -5.4329408347 0.1955619977 0.6483440374
C -3.2949458150 -0.8382349234 1.5330552776
H -3.7215946111 -0.8081191162 2.5325275821
C -2.0087011022 -1.3462705757 1.3546742600
H -1.4313352608 -1.7197842704 2.1935710498
O 0.2343864633 -2.7005826131 -1.4873256773
O 0.5733721864 -2.7646433188 1.0420256363
C 2.1723258565 -0.4159182161 0.5952389709
C 2.6375608537 0.9816745929 0.8908282870
C 2.7990010513 1.8965374869 -0.3335594398
H 3.5871908188 0.9123698335 1.4372956170
H 1.9141396741 1.4401532393 1.5878139740
H -5.8389331414 -0.0715386501 1.6287818757
H -6.1289843481 -0.1641311351 -0.1175571156
H -5.4200707961 1.2914943718 0.5834124987
H 2.2254027518 -0.7943034418 -0.6670336902
H 2.3111995537 -1.2066049596 1.3317671817
C 3.2498087412 3.3135968204 0.0405530125
C 3.4208552777 4.2366240238 -1.1727879479
C 3.8675666829 5.6524992684 -0.7949152246
H 1.8479041432 1.9403397494 -0.8777982569
H 3.5308657366 1.4487889229 -1.0217769047
H 4.1995474463 3.2626109802 0.5939447884
H 2.5185739169 3.7571616086 0.7327071969
H 2.4720524284 4.2862169049 -1.7253764009
H 4.1520144323 3.7939168366 -1.8639332529
H 4.8312223657 5.6379504760 -0.2708919044
H 3.1385629172 6.1349047910 -0.1321290340
H 3.9808553806 6.2858998652 -1.6819886977
37
-1109.88574642
S 0.0208380183 -1.9215045124 0.0331990898
N 0.6461679304 -0.5098707611 -0.7498253564
C -1.7087947115 -1.4817376479 0.1186850971
C -2.2381364361 -1.0077040777 1.3191003906
H -1.6014828604 -0.9220003072 2.1931196220
C -3.5887028999 -0.6660434084 1.3735560240
H -4.0079646958 -0.2991440269 2.3071384250
C -4.4152026604 -0.7922925510 0.2492866323
C -5.8833373883 -0.4468497448 0.3218404474
C -3.8535423386 -1.2742043839 -0.9439810913
H -4.4813667474 -1.3815098837 -1.8252670502
C -2.5089191430 -1.6229644623 -1.0185629645
H -2.0814876901 -2.0078956025 -1.9383150152
O 0.1840387848 -3.0304077602 -0.9121874403
O 0.5426536361 -2.0226386881 1.4058047077
C 1.7370294776 0.1520079519 -0.0515240391
C 1.9678750279 1.5983286657 -0.3769491458
C 3.4006970931 2.0623334481 -0.0741823214
H 1.2524915709 2.2018211264 0.2086516100
H 1.7180029499 1.7789181308 -1.4302105373
H -6.1003033000 0.2077191213 1.1716508539
H -6.4946504073 -1.3514052579 0.4386834760
H -6.2219831821 0.0556088488 -0.5907929764
H 1.8299239416 -0.6837498861 -1.0653005328
H 2.0200323754 -0.2467859278 0.9225619579
C 3.6135611819 3.5579105661 -0.3382377183
C 5.0424323957 4.0312391132 -0.0423554592
C 5.2489361417 5.5259810136 -0.3072475261
H 4.1053127779 1.4766336710 -0.6806605673
H 3.6416565067 1.8390450761 0.9750924561
H 2.9051722167 4.1395388251 0.2703043882
H 3.3671543520 3.7811702471 -1.3869459740
H 5.7494162338 3.4499812942 -0.6510683463
H 5.2880241418 3.8072136693 1.0054095810
H 6.2777668585 5.8322810197 -0.0869522277
H 4.5799943576 6.1349720695 0.3132531616
H 5.0445558346 5.7749505903 -1.3558565473

TS 8 → 10

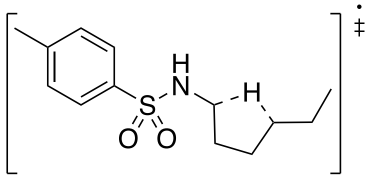

Name	E(B3LYP)	H(B3LYP)	E(RO-B2PLYP-D3)	H(RO-B2PLYP-D3)	NImag
Tosyl_NH_C2_radical_hexane_1_4_HAT_TS_0008	-1110.665019	-1110.338356	-1110.242550	-1109.915887	-1856.2352
Tosyl_NH_C2_radical_hexane_1_4_HAT_TS_0009	-1110.667653	-1110.340116	-1110.243386	-1109.915849	-1867.1380
Tosyl_NH_C2_radical_hexane_1_4_HAT_TS_0005	-1110.667121	-1110.339428	-1110.242794	-1109.915101	-1850.8593
Tosyl_NH_C2_radical_hexane_1_4_HAT_TS_0014	-1110.665787	-1110.338142	-1110.242251	-1109.914606	-1859.6486
Tosyl_NH_C2_radical_hexane_1_4_HAT_TS_0015	-1110.666159	-1110.338826	-1110.240506	-1109.913173	-1852.7169
Tosyl_NH_C2_radical_hexane_1_4_HAT_TS_0041	-1110.667037	-1110.339608	-1110.240060	-1109.912631	-1851.3668
Tosyl_NH_C2_radical_hexane_1_4_HAT_TS_0021	-1110.665602	-1110.338112	-1110.239984	-1109.912494	-1853.0208
Tosyl_NH_C2_radical_hexane_1_4_HAT_TS_0084	-1110.666135	-1110.338595	-1110.239106	-1109.911566	-1865.8489

37
-1109.930149
C 3.5171741546 -0.5299732175 -0.5723809573
C 2.6665096946 1.7697863396 -0.4755606147
H 2.3902241454 1.9268415962 -1.5254780318
H 1.8500561630 0.6606824556 -0.1225998048
C 3.9318518850 0.9369610752 -0.3039183081
C 2.4584372252 2.9887318713 0.3927325177
H 2.5652715334 2.7059317130 1.4503653440
H 3.2643097817 3.7201986359 0.2038958090
H 4.2959857064 1.0307419894 0.7284227073
H 4.7532488833 1.2508832705 -0.9609868113
H 4.2425040352 -1.2621102676 -0.1992276897
H 3.4243804993 -0.6912639802 -1.6562646910
C 1.1044151123 3.6737172146 0.1740014550
H 0.9954920751 4.5538941684 0.8179396139
H 0.2764073262 2.9890230377 0.3903622728
N 1.2938905706 -1.6575925196 -0.5075617283
C 2.1421893274 -0.6664308763 0.0746220051
H 2.1649443829 -0.7856992991 1.1595176586
H 1.2254424816 -1.6627189129 -1.5220479575
S -0.1570279315 -2.1346365223 0.2104711049
O 0.1394693153 -2.3327229582 1.6294488318
O -0.6613607833 -3.2014020589 -0.6558168100
C -1.3025592196 -0.7574297694 0.0895536047
C -2.1122638456 -0.6347484242 -1.0421690488
C -1.3667154300 0.1830688571 1.1196098822
C -2.9845693655 0.4465386000 -1.1403764033
H -2.0709784730 -1.3894307948 -1.8208149336
C -2.2464519722 1.2584984839 1.0040944166
H -0.7524721381 0.0580422937 2.0047868855
C -3.0664983460 1.4088818747 -0.1232349580
H -3.6189569600 0.5412238227 -2.0183879294
H -2.3031792350 1.9884149948 1.8080022398
C -4.0412712760 2.5577556893 -0.2231567453
H -3.6862454499 3.4343118017 0.3282529520
H -4.2070957662 2.8535343062 -1.2642204469
H -5.0177559342 2.2815049385 0.1963161149
H 0.9956485365 4.0063784891 -0.8656585487
37
-1109.930067
C 2.6499507163 0.2179860606 1.9468122547
C 3.8956806789 0.9886954086 -0.0225005060
H 4.5041543837 0.1059612248 -0.2527953103
H 2.5907017226 0.4825550595 -0.1318997431
C 3.7235804952 1.2339660014 1.4733143778
C 4.1310121065 2.1481684285 -0.9617304189
H 3.4053656450 2.9453170230 -0.7434020284
H 5.1238838664 2.5929444241 -0.7714365946
H 3.3567317112 2.2562049404 1.6391397686
H 4.6539753203 1.1327017869 2.0464772492
H 2.2035227412 0.4795853970 2.9133708059
H 3.1118943633 -0.7732188101 2.0629579710
C 4.0305432644 1.7505194144 -2.4399674736
H 3.0426292012 1.3330013448 -2.6650567559
H 4.7779202557 0.9888798138 -2.6940200275
N 0.9355402869 -1.0686012485 0.6493635758
C 1.6445968383 0.1686396814 0.8025100647
H 0.9535392166 1.0159634802 0.7742397319
H 1.5170763974 -1.9037201079 0.7112360226
S -0.0933228656 -1.2652322452 -0.6966794295
O -0.2413057164 -2.7193629405 -0.8206286542
O 0.3564862396 -0.4439215454 -1.8266687245
C -1.6291481172 -0.5618801145 -0.1019468212
C -2.3809346854 -1.2598211025 0.8478622824
C -2.0676688201 0.6591789316 -0.6096839071
C -3.5789959348 -0.7142604815 1.2946577051
H -2.0315918209 -2.2168923258 1.2209888975
C -3.2729465175 1.1916098117 -0.1483231335
H -1.4761679553 1.1729486711 -1.3597446471
C -4.0443643796 0.5182764877 0.8056011161
H -4.1678818852 -1.2531808470 2.0330925041
H -3.6191585552 2.1441676105 -0.5413981683
C -5.3574163150 1.0858524016 1.2893036557
H -5.4208094521 1.0686531820 2.3834584144
H -5.4942202818 2.1196444770 0.9581637680
H -6.2039973755 0.5001606386 0.9087171367
H 4.1971620652 2.6122810529 -3.0962897688
37
-1109.929365
C 3.3619745047 -0.3651979487 -0.6547517020
C 2.2748397275 1.8212137393 -0.4054718599
H 1.9073535081 1.9861896818 -1.4255608950
H 1.6168875844 0.6181898537 -0.0489517930
C 3.6350331040 1.1318771823 -0.3587835939
C 1.9733811528 2.9631570172 0.5380215243
H 0.8922687410 3.1595171896 0.5370337159
H 2.2335797507 2.6679606018 1.5647571423
H 4.0631085363 1.2327476207 0.6482442901
H 4.3678523150 1.5501936014 -1.0602568218
H 4.1871635895 -1.0223921867 -0.3574941189
H 3.2144035779 -0.5013751523 -1.7360393105
C 2.7177891419 4.2669163981 0.1895103720
H 2.4603895140 5.0686776381 0.8923449351
H 2.4601175889 4.6091585898 -0.8199464669
N 1.2664666490 -1.7156658341 -0.4997180216
C 2.0569909647 -0.6714231211 0.0723481530
H 2.1615115472 -0.8258099568 1.1480718070

H 1.1789387120 -1.7075259754 -1.5133004829
S -0.1789442875 -2.2125930045 0.2263461330
O 0.1241397609 -2.3928064218 1.6461241772
O -0.6686972428 -3.2959297409 -0.6274257623
C -1.3368207478 -0.8474908274 0.0891576827
C -2.1784277153 -0.7704172803 -1.0232329279
C -1.3681932319 0.1371008266 1.0790520245
C -3.0515792209 0.3081645986 -1.1415201324
H -2.1587100902 -1.5569951039 -1.7704847562
C -2.2468542996 1.2105500317 0.9420921111
H -0.7281641468 0.0492864329 1.9502592860
C -3.1002268016 1.3143055394 -0.1652337986
H -3.7119504009 0.3669428325 -2.0034188206
H -2.2760247471 1.9762286900 1.7135358466
C -4.0743526571 2.4616930049 -0.2873896282
H -4.2462088419 2.7323809501 -1.3344228660
H -5.0488366957 2.1969199563 0.1439274886
H -3.7153940189 3.3509766099 0.2405489061
H 3.8042621189 4.1260759375 0.2258840306
37
-1109.928900
C 1.2659627390 1.8025637027 0.2473282205
C 3.7191357077 1.7331518519 0.3002308749
H 3.9034096234 1.7405111176 1.3813313836
H 2.9464796033 0.5565318702 0.1516532651
C 2.5395843059 2.6035493957 -0.1204426505
C 4.9805336263 1.7237278873 -0.5302006678
H 4.7205736927 1.5599285732 -1.5859818977
H 5.4536776284 2.7211590917 -0.4953562673
H 2.5717477278 2.7634961911 -1.2068387525
H 2.5440172313 3.5959494947 0.3484045918
H 0.3664032862 2.1611073665 -0.2676975653
H 1.0751092639 1.8889883275 1.3266883267
C 6.0003361168 0.6711118072 -0.0792722047
H 5.5809021118 -0.3389385055 -0.1507977359
H 6.3001401209 0.8333995668 0.9632546876
N 1.0586123507 -0.7080861754 0.6867953645
C 1.6207472151 0.3514809502 -0.0804262717
H 1.6013236599 0.0945318176 -1.1406003785
H 0.9573174589 -0.5556833258 1.6862769657
S -0.1390888516 -1.7492938274 0.0956023527
O -0.3069618391 -2.7588695082 1.1426409209
O 0.2562612314 -2.0788457592 -1.2744900486
C -1.6705218099 -0.8150198680 -0.0030399415
C -2.5212909424 -0.7694673160 1.1039288252
C -1.9802100744 -0.1141878096 -1.1707552769
C -3.6852904548 -0.0073903942 1.0365396829
H -2.2798127213 -1.3407367303 1.9944007255
C -3.1477495965 0.6455699656 -1.2186880236
H -1.3250805995 -0.1842681493 -2.0325921600
C -4.0171182231 0.7122808728 -0.1207999456
H -4.3515647439 0.0243878342 1.8953473260
H -3.3925794831 1.1888405306 -2.1282721907
C -5.2977798213 1.5091073339 -0.1942452964
H -5.2119297779 2.3430471470 -0.8983059518
H -6.1324012892 0.8809160493 -0.5327115898
H -5.5741244804 1.9156330968 0.7842257083
H 6.9055514107 0.7056192861 -0.6956636874
37
-1109.927424
C 3.1833386884 -0.5616079017 0.6093225003
C 3.7145747581 1.5977940469 -0.4277417865
H 4.1528041704 1.2681356943 -1.3776904907
H 2.4235118187 1.0453759671 -0.5047681021
C 4.0744331591 0.6964047108 0.7501023569
C 3.7126529896 3.0981345732 -0.2516970998
H 3.1271236999 3.3597471547 0.6415775651
H 4.7394025570 3.4463009834 -0.0418076199
H 3.8384127781 1.2119270955 1.6911686329
H 5.1406634227 0.4389495328 0.7866614155
H 3.1013423261 -1.1485526439 1.5310843201
H 3.6002801880 -1.2251168186 -0.1618309635
C 3.1677216474 3.8561064108 -1.4681979875
H 2.1296775712 3.5717310177 -1.6764516486
H 3.7580645229 3.6372504089 -2.3663120606
N 1.0105911589 -0.8139844029 -0.7321215843
C 1.8389928698 -0.0232056071 0.1241385146
H 1.2388630203 0.4555550828 0.9012163173
H 1.5113085399 -1.3540717538 -1.4362724658
S -0.1680034649 -1.8555705812 -0.0523200305
O 0.2106603977 -2.2466218542 1.3115344154
O -0.4108971111 -2.8580944690 -1.0965407695
C -1.5973485376 -0.7836725445 0.0807910261
C -2.2486805909 -0.3585565875 -1.0810959719
C -2.0607099309 -0.4112837168 1.3404475134
C -3.3687562155 0.4566818445 -0.9683175900
H -1.8825119680 -0.6679599405 -2.0544923084
C -3.1873139306 0.4082583699 1.4340173709
H -1.5486620135 -0.7673676937 2.2277572375
C -3.8562945253 0.8536209968 0.2887172060
H -3.8783814334 0.7903502556 -1.8691662469
H -3.5521200932 0.7014473124 2.4152021772
C -5.0834919441 1.7275986240 0.3909448702
H -5.9807360648 1.1883726759 0.0615552219
H -5.2546638989 2.0604353323 1.4190324066
H -4.9930511062 2.6177122973 -0.2426503245
H 3.1954341637 4.9396052787 -1.3070322069
37
-1109.926830
C 2.4584101107 0.6809872467 1.7761569622
C 3.8224451203 1.0056665815 -0.2378701541
H 4.4584510264 0.1128698079 -0.2037482851
H 2.5351177218 0.4422737766 -0.3073396109
C 3.5387505994 1.5945265325 1.1417126532
C 4.1313571321 1.9235942521 -1.3988332138
H 4.0699770668 1.3565726818 -2.3372175949
H 3.3623950147 2.7070049311 -1.4605909535
H 3.1378544409 2.6118409933 1.0308526508
H 4.4259155981 1.6698492344 1.7830717142
H 1.9387797285 1.1475785241 2.6213532287
H 2.9329154411 -0.2352329860 2.1565645110
C 5.5182030306 2.5897591979 -1.3071611092
H 6.3147477988 1.8367921548 -1.2737259201
H 5.6054039057 3.2066801876 -0.4050268869
N 0.8656346699 -0.9423271437 0.7394285966
C 1.5350102727 0.3194763355 0.6185298298
H 0.8274570616 1.1071049529 0.3449075216
H 1.4635285654 -1.7240128142 1.0044557533
S -0.1533097615 -1.4753611898 -0.5171732876
O -0.2490205283 -2.9234509881 -0.3035059025
O 0.2682956454 -0.9179811452 -1.8065805052
C -1.7143627037 -0.7094066986 -0.0877372570
C -2.4434939051 -1.1995981662 0.9997748823
C -2.1944373766 0.3489752265 -0.8561036760
C -3.6607105068 -0.6086736217 1.3186776053
H -2.0620933819 -2.0351123886 1.5774805807
C -3.4184048903 0.9304183609 -0.5198736502
H -1.6202883459 0.6991397354 -1.7071042850
C -4.1676466812 0.4646853720 0.5663840546
H -4.2321225038 -0.9866956934 2.1631485844
H -3.7969702967 1.7565609280 -1.1164627221
C -5.4998708483 1.0825724748 0.9180261958
H -6.3243693620 0.3907207679 0.7035425069
H -5.5563690308 1.3288043298 1.9847278774
H -5.6797433415 1.9995377076 0.3489213892
H 5.7032183554 3.2358933076 -2.1739162605
37
-1109.926778
C 3.5051871383 -0.2860245490 -0.1753263911
C 2.1523372944 1.7214357043 -0.6471360383
H 1.3607524966 1.9974024744 -1.3491410836
H 1.6611895948 0.6503760786 0.1457530364
C 3.2668412369 0.8451298841 -1.2169013336
C 2.5457191494 2.8443622207 0.2897601267
H 3.2159135558 2.4605691663 1.0730293745
H 3.1418107552 3.5891694003 -0.2656794036
H 4.1898549926 1.4053819761 -1.4168665759
H 2.9531707052 0.4118571599 -2.1758747676
H 4.1555326338 0.0796483330 0.6273330339
H 3.9907678423 -1.1705395990 -0.6041399481

C 1.3470771680 3.5445190303 0.9398654951
H 0.7514528728 2.8392000428 1.5307111929
H 0.6857716080 3.9771088737 0.1790996249
N 1.4116652506 -1.6115475580 -0.2911379009
C 2.1351891365 -0.6011326653 0.4250390317
H 2.0964341892 -0.8033785786 1.4946437676
H 1.4467002492 -1.5600485091 -1.3065215491
S -0.1081078337 -2.1350939104 0.2302190023
O 0.0273282124 -2.4089268682 1.6609913300
O -0.4986325792 -3.1565360459 -0.7429681835
C -1.2425858785 -0.7545507710 0.0539466085
C -1.4353897195 0.1243265429 1.1235150755
C -1.9120569479 -0.5654334040 -1.1558023576
C -2.2994406935 1.2049714973 0.9671932722
H -0.9325581605 -0.0566707145 2.0673481262
C -2.7736616458 0.5221079309 -1.2936907634
H -1.7777450862 -1.2752268361 -1.9656346180
C -2.9781724539 1.4244257841 -0.2414662071
H -2.4574740575 1.8852011464 1.8008669506
H -3.3034280604 0.6662949892 -2.2319982151
C -3.9117920925 2.6017965679 -0.3926349853
H -3.3760454916 3.5512295822 -0.2700621356
H -4.3893459710 2.6116411172 -1.3769043708
H -4.7041351301 2.5802511924 0.3652747308
H 1.6697319840 4.3553444390 1.6026264247
37
-1109.925829
C 3.4000897468 -0.3770666875 -0.7561786658
C 2.3481667258 1.8369808202 -0.7488949456
H 1.9905988783 1.8617034785 -1.7847338576
H 1.6692754778 0.6918219411 -0.2514436397
C 3.6971537346 1.1372098084 -0.6169328229
C 2.0543466339 3.1163751469 0.0076252240
H 2.7431283965 3.9116156342 -0.3292425354
H 1.0493088725 3.4630301150 -0.2672352284
H 4.1331291913 1.3319063828 0.3708669236
H 4.4320928783 1.4725659795 -1.3603842036
H 4.2145302429 -1.0137810045 -0.3921564651
H 3.2486056013 -0.6222429020 -1.8175250559
C 2.1407111701 3.0064595857 1.5367733084
H 3.1411111962 2.7107088020 1.8721384153
H 1.4291442093 2.2644850086 1.9177567351
N 1.2870612101 -1.6740273279 -0.4664389339
C 2.0909767489 -0.5871200038 -0.0015847797
H 2.1962004440 -0.6358274286 1.0839228996
H 1.1890711768 -1.7607026221 -1.4752890026
S -0.1482745210 -2.1022387464 0.3185894570
O 0.1726014506 -2.1609770610 1.7447136730
O -0.6507603486 -3.2533009284 -0.4331828056
C -1.3077803637 -0.7520024766 0.0818029567
C -2.1437068408 -0.7530099374 -1.0374861578
C -1.3478822478 0.2966389548 1.0029737118
C -3.0188262227 0.3125727097 -1.2332019302
H -2.1188696850 -1.5884328531 -1.7296055946
C -2.2288313098 1.3555931670 0.7894703436
H -0.7131048190 0.2681037029 1.8820772064
C -3.0758714963 1.3820149989 -0.3271151465
H -3.6745157105 0.3110867640 -2.1006550918
H -2.2643279790 2.1714886650 1.5072796305
C -4.0525031839 2.5154753418 -0.5315980964
H -4.2239929568 2.7103863703 -1.5953862979
H -5.0267497090 2.2798899143 -0.0831516267
H -3.6960932972 3.4409955011 -0.0681153567
H 1.9094194412 3.9677533427 2.0094396261

TS 9 → 10

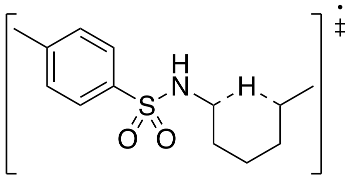

Name	E(B3LYP)	H(B3LYP)	E(RO-B2PLYP-D3)	H(RO-B2PLYP-D3)	NImag
tos_Nhex_crad15crad.conf.001	-1110.67621467	-1110.348821	-1110.25372702870000	-1109.92633335870000	-1744.2053
tos_Nhex_crad15crad.conf.003	-1110.67612545	-1110.348661	-1110.25315835040000	-1109.92569390040000	-1736.2726
tos_Nhex_crad15crad.conf.042	-1110.67630769	-1110.349076	-1110.25075515960000	-1109.92352346960000	-1732.8162
tos_Nhex_crad15crad.conf.040	-1110.67630790	-1110.349076	-1110.25075521740000	-1109.92352331740000	-1732.6999
tos_Nhex_crad15crad.conf.043	-1110.67630773	-1110.349076	-1110.25075494620000	-1109.92352321620000	-1732.8127
tos_Nhex_crad15crad.conf.057	-1110.67630798	-1110.349076	-1110.25075514690000	-1109.92352316690000	-1732.6545
tos_Nhex_crad15crad.conf.063	-1110.67630807	-1110.349076	-1110.25075495560000	-1109.92352288560000	-1732.6402
tos_Nhex_crad15crad.conf.030	-1110.67630800	-1110.349076	-1110.25075486860000	-1109.92352286860000	-1732.6580
tos_Nhex_crad15crad.conf.032	-1110.67630792	-1110.349076	-1110.25075474810000	-1109.92352282810000	-1732.6970
tos_Nhex_crad15crad.conf.084	-1110.67630805	-1110.349076	-1110.25075478770000	-1109.92352273770000	-1732.6606

37
-1109.92633336
S 0.1688863244 -2.0200361760 0.0741049550
N 1.6015009249 -1.2127565324 -0.2849217532
C -1.1746060007 -0.8288741273 0.0048731725
C -1.5851525237 -0.1789244261 1.1697260597
H -1.1130726787 -0.4192388328 2.1161960224
C -2.6197166199 0.7539067438 1.1000547451
H -2.9463773232 1.2552525601 2.0079695954
C -3.2529491988 1.0473088243 -0.1154130582
C -4.3951909853 2.0330773645 -0.1774091584
C -2.8228957020 0.3759788107 -1.2702668944
H -3.3085131109 0.5827392389 -2.2210159733
C -1.7936478160 -0.5603599367 -1.2190367445
H -1.4852825542 -1.0970149317 -2.1105010210
O -0.0174566369 -2.9501208030 -1.0413838228
O 0.2850731995 -2.4581607680 1.4655400800
C 2.1671263687 -0.2277882030 0.6015535907
C 3.5879549386 0.1693690597 0.2154662267
H -4.3884451264 2.5960279022 -1.1168119240
H -4.3530413628 2.7490441070 0.6494511932
H -5.3626464443 1.5174654764 -0.1152803965
H 4.0759746032 0.5911257005 1.1036359250
C 3.6139642696 1.2282945888 -0.9068585508
H 4.1666835672 -0.7187542760 -0.0740641930
H 1.5495376409 0.9608749665 0.4486749145
H 2.0467725540 -0.5578858857 1.6335858939
C 2.8608620247 2.5059501228 -0.4869630267
C 1.4096361803 2.2275342879 -0.1262550921
C 0.7310642500 3.1809239270 0.8306105592
H 3.1649872540 0.8194914953 -1.8253089573
H 4.6533725359 1.4688280867 -1.1602705049
H 2.9294760804 3.2569544149 -1.2905234358
H 3.3730957737 2.9437785510 0.3828273874
H 1.6775791668 -1.0287164643 -1.2818724358
H 0.7941690637 1.9810779802 -0.9995484195
H -0.2852310690 2.8506900426 1.0715947729
H 0.6542549969 4.1958707611 0.4074047933
H 1.2910211824 3.2699524440 1.7705828254
37
-1109.92569390
S 0.1126286607 -2.0385835797 0.1255459115
N 1.4959144453 -1.3006724692 -0.4916816394
C -1.2387636999 -0.8608771195 0.0051854981
C -1.5437031255 -0.0427858520 1.0941945143
H -0.9870346105 -0.1471842583 2.0192070115
C -2.5824926995 0.8808122385 0.9815955092
H -2.8262073060 1.5141569264 1.8312001031
C -3.3244142797 0.9990609446 -0.2014669120
C -4.4693215062 1.9782577147 -0.3047386900
C -3.0000741098 0.1602222794 -1.2785602699
H -3.5708253624 0.2301652023 -2.2015161615
C -1.9674852905 -0.7689873945 -1.1835858990
H -1.7394189846 -1.4330800224 -2.0111047896
O -0.1718709838 -3.1268294884 -0.8117821650
O 0.3684513908 -2.2601479173 1.5492649135
C 2.1228829659 -0.1859450059 0.1647049901
C 3.5047886920 0.1387801339 -0.3879296598
H -4.5507164529 2.3950592603 -1.3142408778
H -4.3519760207 2.8086920826 0.3986232556
H -5.4259528808 1.4894025042 -0.0776520973
H 4.2476888049 -0.5817725748 -0.0180326333
C 3.9144836777 1.5790403925 -0.0336214514
H 3.4914312171 0.0342593702 -1.4839569293
H 1.5123771688 1.0050492301 -0.0001881681
H 2.1110291151 -0.3461837812 1.2448607241
C 2.9281062490 2.6120038270 -0.6235826344
C 1.4998431580 2.3945577828 -0.1372963853
C 1.1445893980 2.9621283594 1.2209092001
H 4.9266646243 1.7755790153 -0.4067933620
H 3.9609215708 1.6877464062 1.0589671581
H 2.9623135276 2.5360943543 -1.7190994049
H 3.2749932697 3.6276717623 -0.3740922238
H 1.4732157672 -1.2725227860 -1.5080164574
H 0.7353419050 2.5852167073 -0.8956615746
H 1.1729098589 4.0633637100 1.2160430378
H 1.8432918586 2.6294863701 1.9993240047
H 0.1366051044 2.6614221925 1.5281559764
37
-1109.92352347
S 0.0939991157 -1.1406752412 -0.5615891842
N 1.0525311415 -0.5146163832 0.6963650903
C -1.5513778784 -0.6373087639 -0.0637921461
C -2.2094999165 0.3630932288 -0.7758019880
H -1.7202237205 0.8292634582 -1.6241567042
C -3.4971813204 0.7372349022 -0.3869938811
H -4.0144260652 1.5176429488 -0.9394424444
C -4.1342280474 0.1219760325 0.6964644776
C -5.5342802653 0.5143696383 1.1041997783
C -3.4475617432 -0.8879979160 1.3917070987
H -3.9291460339 -1.3798171843 2.2335874051
C -2.1646693844 -1.2732245795 1.0197448242

H -1.6428606367 -2.0605344418 1.5536894567
O 0.3871115560 -0.4759655015 -1.8375848503
O 0.2205951810 -2.5954543614 -0.4174648705
C 1.4965616045 0.8599949701 0.6224249227
C 2.4859339293 1.2228163222 1.7225004046
H -5.8633944182 1.4211739958 0.5879529003
H -5.5998001825 0.6955070633 2.1833029873
H -6.2516674467 -0.2818162156 0.8675221391
H 2.4597643155 2.3116545559 1.8578537049
C 3.9305452526 0.8077222030 1.3643702198
H 2.1831796590 0.7787180689 2.6820676355
H 2.2721332657 1.0382184318 -0.4648789182
H 0.6362303596 1.5279920849 0.5388010152
C 4.3904282302 1.4534115195 0.0416662435
C 3.4865313949 1.0700799840 -1.1195091691
C 3.4109720775 2.0003918609 -2.3073746897
H 4.0000445017 -0.2876907646 1.2808548503
H 4.6045509219 1.0924135207 2.1813102851
H 5.4368170430 1.1734882090 -0.1607888757
H 4.3859543025 2.5471857823 0.1632784113
H 1.7926710877 -1.1929225334 0.8730605808
H 3.5805779525 0.0161161339 -1.4038427523
H 4.3720662861 2.0567363249 -2.8439549585
H 3.1560220907 3.0232556551 -2.0005854141
H 2.6542422335 1.6615679983 -3.0229822057
37
-1109.92338639
S 0.0789243689 -1.7117458170 0.2992577964
N 1.2844734657 -0.5474745329 0.4917017975
C -1.4926089488 -0.8601561502 0.1072040172
C -2.2419643670 -0.5343557227 1.2406386088
H -1.8965959148 -0.8414422715 2.2224958374
C -3.4394647414 0.1602608335 1.0885825760
H -4.0263716050 0.4095463742 1.9694776368
C -3.9060573374 0.5357974597 -0.1800648508
C -5.2220527923 1.2601600748 -0.3351007049
C -3.1376304806 0.1916990931 -1.3007480786
H -3.4872708381 0.4656313033 -2.2932779595
C -1.9379462702 -0.5058361590 -1.1680658406
H -1.3603748824 -0.7924703175 -2.0404190123
O 0.3613791892 -2.3811887173 -0.9715299513
O 0.0360972722 -2.4198716564 1.5806793357
C 1.7224426517 0.3099106932 -0.5754833365
C 1.2450486806 1.7607970027 -0.5585698399
H -5.2476544438 1.8539138948 -1.2544456881
H -5.4130484166 1.9305908262 0.5094921058
H -6.0583522397 0.5500801881 -0.3813835637
H 1.3676160901 2.1775796487 -1.5674402868
C 2.0617977657 2.6210626992 0.4290801174
H 0.1732571215 1.8163212943 -0.3214975068
H 3.0327848123 0.4950622443 -0.3818164738
H 1.6279624366 -0.2153934851 -1.5252222818
C 3.5636167681 2.6031889349 0.0777074389
C 4.1393916475 1.1959062221 0.0879359504
C 5.3471750247 0.9168492904 -0.7761629576
H 1.9197616837 2.2507764712 1.4563112768
H 1.6853463883 3.6509400445 0.4229619709
H 4.1130058006 3.2564729235 0.7744280201
H 3.6957729146 3.0430693884 -0.9219642340
H 1.2768961878 -0.1595504245 1.4305309324
H 4.2183100237 0.7741960327 1.0967541379
H 5.1629788893 1.1919018420 -1.8224800111
H 5.6251953364 -0.1425530685 -0.7497796278
H 6.2253425289 1.4933322420 -0.4428392342
37
-1109.92298508
S 0.0506542788 -1.7866757521 0.0958819164
N 1.2309416089 -0.7304170550 0.6848005679
C -1.5057865738 -0.8906102927 0.0125971331
C -1.8563409328 -0.2180123264 -1.1601070696
H -1.2148337030 -0.2848126382 -2.0324131751
C -3.0445803379 0.5097414413 -1.1983152282
H -3.3206788184 1.0310348607 -2.1118244012
C -3.8950105956 0.5716513036 -0.0854611085
C -5.1976659850 1.3330869673 -0.1473615066
C -3.5228842861 -0.1203174463 1.0765238079
H -4.1740951876 -0.0924677344 1.9469729363
C -2.3379827215 -0.8503057412 1.1338533098
H -2.0662365240 -1.4016125976 2.0282304621
O 0.4396085553 -2.0990650438 -1.2804643894
O -0.0899601204 -2.8081280669 1.1359894177
C 1.7593191182 0.3617347029 -0.0769497762
C 1.2784299598 1.7724425296 0.2501173246
H -5.1473989000 2.1568536821 -0.8666939703
H -5.4662585078 1.7489327317 0.8293725098
H -6.0215565328 0.6775056629 -0.4591416245
H 0.3012400560 1.9732705414 -0.2128992687
C 2.3134701628 2.8208608474 -0.1961577307
H 1.1287858593 1.8579986744 1.3373579986
H 3.0714232813 0.4967980979 0.1455186704
H 1.7352224877 0.1079956454 -1.1382579034
C 3.6770427802 2.6064612435 0.5006457781
C 4.2557996678 1.2221681204 0.2343412809
C 4.9843869915 1.0169211141 -1.0765404642
H 1.9394565336 3.8281139701 0.0228094058
H 2.4401979736 2.7681936664 -1.2866446967
H 3.5396919352 2.7486043457 1.5812389911
H 4.3779095472 3.3906169503 0.1723870805
H 1.1363570064 -0.5928317077 1.6870431047
H 4.7711280395 0.7785011233 1.0905801617
H 5.2494146940 -0.0352394387 -1.2291020638
H 5.9180184879 1.6003354614 -1.1097855525
H 4.3789278946 1.3342208287 -1.9350061694
37
-1109.92297441
S 0.0734112357 -1.4861393963 -0.0892367847
N 1.0522747020 -0.5473452405 0.9383652655
C -1.5320154475 -0.7176853192 0.1130719226
C -2.2332810043 -0.8978587980 1.3090698771
H -1.8035584001 -1.4957674075 2.1060121311
C -3.4847258774 -0.3101705354 1.4539275431
H -4.0344497083 -0.4478975076 2.3820491150
C -4.0535085312 0.4543174982 0.4209221907
C -5.4214679712 1.0709189152 0.5907282691
C -3.3307336805 0.6130371687 -0.7667253749
H -3.7566485299 1.1970791717 -1.5786774111
C -2.0729744475 0.0295320112 -0.9308781963
H -1.5182498021 0.1397071265 -1.8563677081
O 0.4665745172 -1.3143943334 -1.4930108390
O 0.0586479209 -2.8106939294 0.5416264056
C 1.6567557436 0.6527644980 0.4133487821
C 2.5439029805 1.3804359147 1.4112860934
H -5.4954656631 1.6242435522 1.5341581544
H -6.2023022370 0.2999020364 0.6080417508
H -5.6548013830 1.7610013387 -0.2256160506
H 1.9428971926 1.9351336910 2.1462971904
C 3.5174225691 2.3233851476 0.6792053710
H 3.1277629365 0.6422045963 1.9828458914
H 2.5299472886 0.4539296260 -0.5930950899
H 0.8917861811 1.2934045941 -0.0354476153
C 4.4400009005 1.5508150371 -0.2916575291
C 3.6565752868 0.7551604656 -1.3304381263
C 3.1673927467 1.5016292358 -2.5535703342
H 4.1222283505 2.8698558123 1.4128312055
H 2.9434070032 3.0800485854 0.1259875812
H 5.0693646083 0.8705803413 0.2984467159
H 5.1268914682 2.2616613752 -0.7783454947
H 1.7025015232 -1.1828217900 1.3998452663
H 4.0887878299 -0.2204513997 -1.5678185892
H 2.4911728560 0.8800573209 -3.1506581611
H 4.0043344631 1.8036237436 -3.2028841581
H 2.6267849888 2.4182762132 -2.2836007532
37
-1109.92275794
S 0.2359435582 -1.9105887057 0.1993609630
N 1.4260160642 -1.1729919334 1.1460699486
C -1.1704324817 -0.7960298260 0.0853177464
C -2.0594752263 -0.6985287694 1.1598603172
H -1.9141208544 -1.3116279421 2.0438132987
C -3.1498916136 0.1611335865 1.0657693086

H -3.8468605268 0.2310632228 1.8975239383
C -3.3753712210 0.9252789613 -0.0899957071
C -4.5501004867 1.8705373519 -0.1709609967
C -2.4793094393 0.7949034806 -1.1589908941
H -2.6493998299 1.3610694356 -2.0715370686
C -1.3781015828 -0.0577446484 -1.0797290824
H -0.6972864411 -0.1727960352 -1.9160880754
O 0.7812580913 -2.0545221319 -1.1515173526
O -0.1872605476 -3.0580114276 1.0052824233
C 2.1650066290 0.0118799757 0.7798041035
C 3.1990768400 -0.1333005463 -0.3291163582
H -4.3232974553 2.8239070118 0.3245206503
H -5.4349429932 1.4530799360 0.3214378928
H -4.8128548786 2.0958095283 -1.2092413626
H 4.0594951776 -0.7151362617 0.0334587622
C 3.6547877328 1.2502155541 -0.8243532126
H 2.7647700675 -0.7000518231 -1.1587917178
H 1.4619910766 1.0764961011 0.3344159357
H 2.5945743018 0.4228739574 1.6987106139
C 2.4723136357 2.0790937597 -1.3760199847
C 1.3768005383 2.2944783805 -0.3395293600
C 1.6071424903 3.3795378280 0.6919168177
H 4.4142358599 1.1279651573 -1.6057649124
H 4.1425436582 1.7976689554 -0.0046765152
H 2.0568521826 1.5512952621 -2.2448021434
H 2.8486160822 3.0452814976 -1.7496884085
H 1.1563720624 -1.2357815857 2.1230620699
H 0.3648259661 2.3010893767 -0.7513916892
H 2.5693059364 3.2600389864 1.2061173103
H 0.8205806884 3.3843675476 1.4557072573
H 1.6172572634 4.3779410848 0.2267558169
37
-1109.92191280
S 0.1491791965 -1.3488189198 0.5061029199
N 1.1183345963 -1.0918951029 -0.8739762640
C -1.4306100673 -0.6387126939 0.0421647008
C -2.1137094168 -1.1352370177 -1.0720430729
H -1.6709305541 -1.9175709874 -1.6796555744
C -3.3619834801 -0.6124730907 -1.3892743037
H -3.8946956852 -0.9938907871 -2.2572334648
C -3.9488084814 0.3965019096 -0.6064175366
C -5.3040279776 0.9546129562 -0.9699766009
C -3.2468859600 0.8664854168 0.5087674204
H -3.6868804818 1.6420380986 1.1305561389
C -1.9910261055 0.3546858135 0.8413737458
H -1.4485399637 0.7153219840 1.7085456829
O 0.0021319653 -2.8084682522 0.5674748674
O 0.6534354568 -0.5809266058 1.6511038852
C 2.0497969702 0.0160007049 -0.9308267196
C 1.4495708912 1.4115966798 -1.0026301412
H -5.6498104047 1.6822205365 -0.2297473528
H -6.0561337967 0.1594620158 -1.0378795936
H -5.2777862695 1.4560408214 -1.9455488644
H 1.0479130132 1.6114532682 -2.0072937832
C 2.5011994228 2.4677982244 -0.6161034351
H 0.6047530946 1.4874310728 -0.3076778136
H 2.8704768394 0.1490298009 0.1360544849
H 2.7508397865 -0.1844256592 -1.7468034649
C 3.0252159661 2.2460249329 0.8215478899
C 3.6612561774 0.8727956605 1.0023032075
C 5.0968404380 0.7152644837 0.5461778885
H 2.0644171113 3.4700742590 -0.7030022615
H 3.3379283769 2.4331740168 -1.3288662069
H 2.1826926558 2.3506667249 1.5167220986
H 3.7419346744 3.0447019040 1.0721239429
H 1.5453473124 -1.9927162570 -1.0802281198
H 3.4537953653 0.4093227055 1.9688190621
H 5.2377027763 1.0478250837 -0.4907391877
H 5.4288984341 -0.3277933314 0.6082241938
H 5.7826542820 1.3130164683 1.1673820644
37
-1109.92173788
S 0.1102449675 -1.5935152894 -0.1590857556
N 1.0827430258 -0.2595583866 -0.5906228676
C -1.5073903824 -0.8395586728 0.0036836362
C -2.1140275926 -0.7798262009 1.2560943127
H -1.5971995016 -1.1791372445 2.1219726799
C -3.3849399978 -0.2124021467 1.3683561005
H -3.8614764223 -0.1629690182 2.3442028888
C -4.0560613632 0.2886254354 0.2475795424
C -5.4372161013 0.8873709014 0.3671639948
C -3.4210655989 0.2096849898 -1.0038246119
H -3.9291979864 0.5915422905 -1.8862171452
C -2.1561373333 -0.3513346870 -1.1346935569
H -1.6741077223 -0.4153633808 -2.1046600530
O 0.1289381294 -2.4566844874 -1.3464529402
O 0.4776839109 -2.1179260354 1.1637594369
C 1.6384611090 0.5840785740 0.4415455676
C 2.9362891261 0.1427425695 1.1110905567
H -5.7309674693 1.0129009958 1.4136069833
H -6.1876826392 0.2479504492 -0.1148703510
H -5.4905025446 1.8681513749 -0.1197514901
H 3.0445667026 0.6936100967 2.0553362033
C 4.1643425457 0.4375283492 0.2251240634
H 2.8841996905 -0.9230420453 1.3683726786
H 2.0708031383 1.6822955446 -0.2111822742
H 0.8498136940 0.9137217354 1.1194021888
C 4.2687338774 1.9407564781 -0.1051258418
C 3.0322534459 2.4577110049 -0.8245971591
C 2.6926674831 3.9242554154 -0.6843446979
H 4.1021322028 -0.1414718393 -0.7092373338
H 5.0763279051 0.0986459101 0.7304839308
H 5.1741761530 2.1264711754 -0.7045646676
H 4.4028272962 2.4989415152 0.8332837362
H 1.7527357768 -0.5941261734 -1.2816863602
H 2.9570310227 2.0922370850 -1.8557736832
H 2.6054792353 4.2152601373 0.3701398391
H 1.7475331065 4.1698056067 -1.1816600697
H 3.4715064920 4.5635500420 -1.1301666155
37
-1109.92119826
S 0.0743341133 -1.7259146152 -0.1827825380
N 1.0917150945 -0.5154946493 -0.8346859469
C -1.4908678523 -0.8665863390 -0.0279252220
C -2.1856046041 -0.4895391960 -1.1812158741
H -1.7707212579 -0.7035061937 -2.1607069993
C -3.4099297740 0.1557939451 -1.0525778420
H -3.9533831667 0.4518231800 -1.9468026985
C -3.9592478452 0.4287978569 0.2119636356
C -5.2986505522 1.1159515015 0.3311921097
C -3.2439362883 0.0354516334 1.3482600152
H -3.6543018001 0.2350538621 2.3349627442
C -2.0127082354 -0.6139989668 1.2385091654
H -1.4625451747 -0.9298273721 2.1182036407
O -0.0287580325 -2.7305507729 -1.2483306717
O 0.5011955650 -2.0944479178 1.1741298683
C 1.8042197506 0.3655107814 0.0520322347
C 3.1492447345 -0.0847017543 0.6085304213
H -5.5145154941 1.3975581905 1.3661889804
H -6.1099880027 0.4611893755 -0.0115901281
H -5.3375742951 2.0236598980 -0.2822535085
H 3.0014775950 -0.7702495301 1.4540865590
C 4.0048503457 1.1263939270 1.0196278080
H 3.6837310904 -0.6552739675 -0.1663349263
H 2.1739283981 1.4844135213 -0.6024757359
H 1.1237840459 0.7605909062 0.8106012992
C 4.2738783768 2.0642033048 -0.1803024888
C 2.9888761639 2.5775431372 -0.8199948768
C 2.3126887763 3.7591828524 -0.1565404980
H 4.9569356875 0.7832988757 1.4414657699
H 3.4951540273 1.6812736911 1.8200492924
H 4.8618120670 1.5118567514 -0.9257479602
H 4.9044222951 2.9050950483 0.1499440120
H 1.6703494426 -0.9684363773 -1.5408466023
H 3.0344259050 2.6630939460 -1.9094212708
H 1.3401760895 3.9761002651 -0.6130745969
H 2.9264712627 4.6695010790 -0.2418702783
H 2.1461749154 3.5856772851 0.9141172477
37
-1109.92073574

S 0.2150578432 -1.3584498335 0.5114596920
N 1.1902051832 -1.1606565250 -0.8677088409
C -1.3441078595 -0.6210606195 0.0205178562
C -1.9823123583 -1.0525641233 -1.1435899653
H -1.5094890017 -1.7897200512 -1.7840015713
C -3.2244409719 -0.5166862550 -1.4750231365
H -3.7212415541 -0.8459945971 -2.3840920676
C -3.8462898608 0.4371195598 -0.6562906439
C -5.1952453341 1.0130328704 -1.0161955406
C -3.1867887662 0.8444716484 0.5119269562
H -3.6533184604 1.5810917581 1.1618030326
C -1.9425446017 0.3213611836 0.8580834600
H -1.4336722783 0.6339239550 1.7632157293
O 0.0439731789 -2.8137664043 0.6043160065
O 0.7171263380 -0.5852983997 1.6552186645
C 2.0835342476 -0.0331688335 -1.0662193668
C 1.4459697648 1.3485826613 -1.2355850291
H -5.5626957150 0.6121916224 -1.9655379778
H -5.1501063229 2.1050785099 -1.1070524854
H -5.9413969350 0.7853677614 -0.2450715072
H 1.9443401686 1.8509154502 -2.0753462111
C 1.5851723882 2.2499199629 0.0129312233
H 0.3900175732 1.2489381943 -1.5173001466
H 2.9409839555 0.1945585139 -0.0469288717
H 2.7401220857 -0.3085715043 -1.8949168310
C 3.0464464669 2.3293847809 0.4952978254
C 3.5762398047 0.9650333376 0.9088466533
C 5.0713112285 0.7476113149 0.8544441833
H 0.9610424372 1.8594160496 0.8243906059
H 1.2113196959 3.2529082489 -0.2284734034
H 3.1204935249 3.0454852090 1.3296116926
H 3.6722013270 2.7388647229 -0.3131773604
H 1.6400958518 -2.0621539373 -1.0122210326
H 3.1019152404 0.5796276541 1.8148476541
H 5.3404601323 -0.2843050407 1.1081437630
H 5.6023316811 1.4035673384 1.5634083489
H 5.4753403530 0.9670744182 -0.1429568966
37
-1109.92064525
S 0.3003930302 -1.8608173416 0.1920971301
N 1.4477290999 -1.1653455017 1.2109532729
C -1.1115268499 -0.7543854505 0.0670207505
C -1.9740554840 -0.6132583319 1.1591365203
H -1.8007554740 -1.1796311747 2.0690885622
C -3.0703408467 0.2366085498 1.0568900868
H -3.7410558788 0.3461254744 1.9059010363
C -3.3335492474 0.9444484098 -0.1273179090
C -4.5436764860 1.8415640532 -0.2319068882
C -2.4629382060 0.7742944973 -1.2104727617
H -2.6558175398 1.3041681118 -2.1400345916
C -1.3545572511 -0.0694993722 -1.1228548583
H -0.6914196332 -0.2128443919 -1.9687954024
O 0.9012569087 -1.9474670045 -1.1404151703
O -0.1504283201 -3.0412422678 0.9332903455
C 2.2712466668 -0.0123875828 0.9277701461
C 3.2927992256 -0.1365139889 -0.2106812253
H -4.6492043841 2.4761357636 0.6553304806
H -5.4654311703 1.2516151118 -0.3175824870
H -4.4858408481 2.4924907110 -1.1095383938
H 4.2701857317 0.1890727297 0.1705788067
C 2.9425517508 0.7213747925 -1.4451860596
H 3.3916395327 -1.1863361579 -0.5033587712
H 1.6115921696 1.1047835128 0.5797643596
H 2.7217557599 0.2810224541 1.8787340161
C 2.6342557366 2.1812612693 -1.0607894226
C 1.4140144045 2.2743210013 -0.1584455383
C 1.3139648888 3.4566827953 0.7782621689
H 2.0769843972 0.2811503779 -1.9549085881
H 3.7776461089 0.6859238221 -2.1554818421
H 2.4909614506 2.7841898050 -1.9719737739
H 3.5066802532 2.6118451607 -0.5456688144
H 1.1508744322 -1.2672222935 2.1761025466
H 0.4789412419 2.0499543878 -0.6802327523
H 1.2428413661 4.4074462708 0.2254315188
H 2.1980872824 3.5324772074 1.4249837405
H 0.4292830662 3.3908086472 1.4223349504
37
-1109.91990957
S 0.2288298250 -1.4165699494 0.3041202585
N 1.1640552574 -1.0150166041 -1.0583840940
C -1.3436146585 -0.6226585982 -0.0303535579
C -1.9130774022 0.1929787349 0.9449499938
H -1.3773178854 0.3792283787 1.8691682892
C -3.1695816969 0.7552721858 0.7121840173
H -3.6155740316 1.3928797126 1.4712834184
C -3.8643273999 0.5105121262 -0.4773375257
C -5.2164017045 1.1315808271 -0.7347734075
C -3.2697757607 -0.3242068638 -1.4390112412
H -3.7970643660 -0.5308710588 -2.3673508302
C -2.0205408532 -0.8946259109 -1.2235004740
H -1.5694550019 -1.5386873247 -1.9710427393
O 0.0602638985 -2.8704717450 0.1874106282
O 0.7634682252 -0.8167114171 1.5338013854
C 2.0519119647 0.1325948066 -1.1151229832
C 1.4105445633 1.5219459398 -1.0623681139
H -5.9448522153 0.3796509621 -1.0597176773
H -5.1596244340 1.8882209216 -1.5278017381
H -5.6109666786 1.6192285561 0.1616257843
H 1.8846558344 2.1433530946 -1.8337207730
C 1.5864033698 2.2318348535 0.2998676027
H 0.3468014334 1.4606190105 -1.3251251019
H 2.9384677033 0.2119580838 -0.0984225891
H 2.6842565515 -0.0162354226 -1.9935780945
C 3.0610271242 2.2450402628 0.7466596758
C 3.6013304284 0.8367361391 0.9412988885
C 5.0940634542 0.6346138469 0.8133873889
H 0.9856762623 1.7248609993 1.0631421795
H 1.2064819888 3.2580096380 0.2183602616
H 3.1596386436 2.8319660805 1.6741040626
H 3.6636039471 2.7702271907 -0.0109380180
H 1.6088541319 -1.8839263620 -1.3466916258
H 3.1527261048 0.3215427767 1.7943481901
H 5.4697073088 0.9990137311 -0.1521766955
H 5.3694587913 -0.4224155752 0.9056008403
H 5.6456000052 1.1815283412 1.5954232496
37
-1109.91985205
S 0.1907366453 -2.0175848986 0.0615813317
N 1.6198518779 -1.1633335081 -0.2058307173
C -1.1672396480 -0.8446577701 -0.0204836961
C -1.6240908279 -0.2312401145 1.1470545199
H -1.1858182785 -0.4972533413 2.1028412601
C -2.6561829619 0.7025470967 1.0656792061
H -3.0159906257 1.1790502214 1.9743741508
C -3.2430350294 1.0315635807 -0.1639080240
C -4.3835795441 2.0183815990 -0.2396315138
C -2.7682607422 0.3952839349 -1.3211662266
H -3.2177095660 0.6300933352 -2.2831121581
C -1.7388784508 -0.5404207402 -1.2587927390
H -1.3932122056 -1.0479039388 -2.1536759607
O 0.0728996155 -2.9084276635 -1.0945270698
O 0.2591596840 -2.5060853951 1.4391586562
C 2.0937986632 -0.1788615816 0.7314103888
C 3.4677877560 0.3755134771 0.3888452219
H -4.3636176241 2.7202332037 0.6001428952
H -5.3521186424 1.5018126726 -0.2112698613
H -4.3525250138 2.5969877575 -1.1689193949
H 3.8153039902 0.9037842894 1.2838095148
C 3.4748196142 1.3617315043 -0.8180019678
H 4.1826758155 -0.4397506657 0.2140715526
H 1.4001283320 0.9744084776 0.6525597390
H 2.0036975168 -0.5673671626 1.7463366682
C 2.1021990154 2.0303016019 -1.1174350312
C 1.2422277676 2.2549227818 0.1154018520
C 1.7073323285 3.2831770835 1.1238054696
H 3.8140703859 0.8464614311 -1.7249993901
H 4.2285425722 2.1328131626 -0.6221682951
H 1.5364651983 1.3943421356 -1.8121725717
H 2.2791021750 2.9721563470 -1.6605365386
H 1.7340184785 -0.9474086543 -1.1926621219

H 0.1740010660 2.3192912706 -0.1078529540
H 1.6902635296 4.2996461337 0.6980532468
H 2.7353538364 3.0992769243 1.4602833240
H 1.0650286833 3.2931972411 2.0119441635
37
-1109.91922626
S 0.0401678751 -1.3411924388 0.1758842044
N 0.9004477731 0.1386881320 0.0357875699
C -1.6475629981 -0.7736990991 -0.0187335706
C -2.3728078003 -1.1578461254 -1.1442182525
H -1.9060013411 -1.7859456771 -1.8953233980
C -3.6951225433 -0.7300976035 -1.2775479870
H -4.2651214748 -1.0276783117 -2.1540999172
C -4.2994908727 0.0702277781 -0.3017075835
C -5.7339012941 0.5215126109 -0.4402218056
C -3.5440974137 0.4380080190 0.8250061840
H -3.9988501687 1.0573328649 1.5945966748
C -2.2267645744 0.0207406552 0.9756343974
H -1.6505216387 0.3023979165 1.8507298354
O 0.3400338523 -2.2354556534 -0.9546985239
O 0.2509622345 -1.7918934586 1.5549986341
C 2.2122388130 0.1981154857 0.6477800636
C 3.3959594792 -0.3557674036 -0.1415885814
H -5.8250358853 1.6063639151 -0.3103837181
H -6.3706435344 0.0522717856 0.3204408556
H -6.1429448853 0.2631200678 -1.4215216465
H 4.2190264239 -0.5519522048 0.5586232954
C 3.8878735771 0.6384409040 -1.2143685667
H 3.1331956322 -1.3182456147 -0.6019508705
H 2.5278682080 1.5086896760 0.6615374389
H 2.1446600113 -0.1282644709 1.6843915203
C 4.2984768064 1.9842602245 -0.5827279347
C 3.1503798351 2.6371183534 0.1718080839
C 3.4923588616 3.5811580152 1.3021071238
H 3.0968899767 0.8083372071 -1.9613054640
H 4.7329014954 0.2053429486 -1.7623986973
H 4.6848608425 2.6580413068 -1.3641103688
H 5.1346054741 1.8075467568 0.1099095360
H 0.9115521622 0.4015032197 -0.9500661569
H 2.3701736352 3.0237980654 -0.4948555085
H 4.1392311191 3.0974216152 2.0448307001
H 2.5932203736 3.9361163149 1.8182678161
H 4.0315664362 4.4701308756 0.9372111231

TS_INTER 7' → 10'

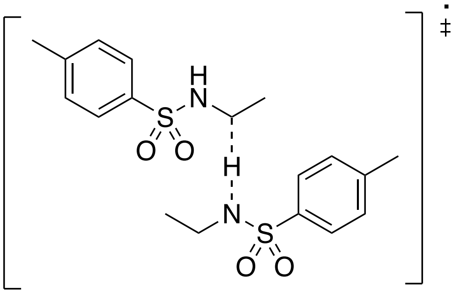

Name	E(B3LYP)	H(B3LYP)	E(RO-B2PLYP-D3)	H(RO-B2PLYP-D3)	NImag
di_tos_Net_HAT.conf004	-1907.559959	-1907.123372	-1906.927839	-1906.491252	-1311.2132
di_tos_Net_HAT.conf011	-1907.559959	-1907.123372	-1906.927839	-1906.491252	-1311.2097
di_tos_Net_HAT.conf066	-1907.558689	-1907.121849	-1906.926747	-1906.489907	-1275.5743
di_tos_Net_HAT.conf018	-1907.558689	-1907.121849	-1906.926746	-1906.489906	-1275.5503
di_tos_Net_HAT.conf006	-1907.557028	-1907.120320	-1906.924909	-1906.488201	-1168.6136
di_tos_Net_HAT.conf019	-1907.555325	-1907.118505	-1906.923362	-1906.486543	-1337.5053
di_tos_Net_HAT.conf058	-1907.558255	-1907.121737	-1906.921773	-1906.485255	-1559.9359
di_tos_Net_HAT.conf090	-1907.559143	-1907.122684	-1906.920910	-1906.484451	-1464.1228
di_tos_Net_HAT.conf084	-1907.553134	-1907.116549	-1906.919881	-1906.483297	-1264.7311
di_tos_Net_HAT.conf022	-1907.552314	-1907.115438	-1906.918998	-1906.482122	-1155.1299
Reactant					
di_tos_Net_HAT.conf058_opt_rev	-1907.594475	-1907.152423	-1906.961005	-1906.518953	
di_tos_Net_HAT.conf011_opt_fwd	-1907.583240	-1907.140782	-1906.952708	-1906.510250	
di_tos_Net_HAT.conf006_opt_rev	-1907.582123	-1907.139718	-1906.952143	-1906.509737	
di_tos_Net_HAT.conf075_opt_fwd	-1907.584251	-1907.142159	-1906.949298	-1906.507207	
di_tos_Net_HAT.conf019_opt_rev	-1907.582060	-1907.140048	-1906.948017	-1906.506005	
di_tos_Net_HAT.conf093_opt_rev	-1907.582690	-1907.140520	-1906.947944	-1906.505773	
di_tos_Net_HAT.conf062_opt_fwd	-1907.582515	-1907.140182	-1906.946525	-1906.504192	
Product					
di_tos_Net_HAT.conf011_opt_rev	-1907.584550	-1907.143341	-1906.940877	-1906.499669	
di_tos_Net_HAT.conf058_opt_fwd	-1907.584574	-1907.142375	-1906.941619	-1906.499420	
di_tos_Net_HAT.conf066_opt_fwd	-1907.583824	-1907.141014	-1906.940868	-1906.498058	
di_tos_Net_HAT.conf027_opt_rev	-1907.584383	-1907.141616	-1906.938218	-1906.495451	
di_tos_Net_HAT.conf090_opt_fwd	-1907.582489	-1907.140438	-1906.936788	-1906.494737	
di_tos_Net_HAT.conf006_opt_fwd	-1907.583644	-1907.140922	-1906.936943	-1906.494221	
di_tos_Net_HAT.conf019_opt_fwd	-1907.579113	-1907.137176	-1906.936022	-1906.494084	
di_tos_Net_HAT.conf075_opt_rev	-1907.581979	-1907.139236	-1906.936505	-1906.493762	

51

S -0.910499 -1.808715 -0.515367
N -0.128073 -1.321268 0.901027
C -2.427104 -0.856204 -0.463434
C -3.636134 -1.507820 -0.221662
H -3.647140 -2.582946 -0.077666
C -4.814094 -0.760970 -0.188015
H -5.759397 -1.265121 -0.003170
C -4.800040 0.624112 -0.394469
C -6.080817 1.423784 -0.388258
C -3.566689 1.252356 -0.634366
H -3.538682 2.327029 -0.799530
C -2.381160 0.525637 -0.671102
H -1.432417 1.016232 -0.860833
O -1.276751 -3.236144 -0.501368
O -0.078963 -1.292557 -1.622189
C -0.550019 -2.048358 2.108705
C 0.205486 -1.516401 3.324064
H -5.946296 2.392904 0.104600
H -6.885660 0.889156 0.125543
H -6.421875 1.626571 -1.412009
H -1.628584 -1.877225 2.244127
H -0.150772 -2.007878 4.235927
H 0.058388 -0.436880 3.427376
H 1.281428 -1.706218 3.240009
S 3.499131 0.349858 -0.653004
N 2.723307 -1.148371 -0.827271

C 2.264596 1.549168 -0.149779
C 1.522193 2.217755 -1.125279
H 1.705961 2.022333 -2.176327
C 0.572324 3.156392 -0.727346
H 0.004447 3.691892 -1.484572
C 0.349248 3.434895 0.629577
C -0.706054 4.429101 1.051403
C 1.118838 2.756959 1.585267
H 0.975356 2.975501 2.640617
C 2.074215 1.816607 1.206723
H 2.685197 1.313189 1.947995
O 4.435741 0.181059 0.459733
O 3.926937 0.702940 -2.005258
C 2.394088 -1.966778 0.283661
C 2.329088 -3.449478 -0.047737
H 1.992526 -4.018606 0.824769
H 3.323968 -3.809841 -0.336391
H 1.635839 -3.647776 -0.870497
H -0.437825 4.925800 1.989472
H -1.671076 3.929841 1.212254
H -0.859347 5.200058 0.289263
H 2.039077 -1.163731 -1.587333
H 1.209603 -1.627288 0.654984
H -0.421713 -3.134535 2.002363
H 3.040750 -1.730198 1.131615

51

S -0.910470 -1.808708 -0.515404
N -0.128073 -1.321278 0.901014
C -2.427089 -0.856221 -0.463464
C -2.381163 0.525632 -0.671110
H -1.432427 1.016238 -0.860844
C -3.566701 1.252325 -0.634396
H -3.538711 2.326997 -0.799581
C -4.800052 0.624055 -0.394517
C -6.080789 1.423790 -0.388027
C -4.814090 -0.761028 -0.188128
H -5.759393 -1.265206 -0.003362
C -3.636110 -1.507862 -0.221753
H -3.647108 -2.582996 -0.077819
O -1.276698 -3.236144 -0.501440
O -0.078924 -1.292513 -1.622201
C -0.550035 -2.048397 2.108671
C 0.205441 -1.516453 3.324054
H -6.420364 1.629397 -1.411697
H -5.946894 2.391568 0.107660
H -6.886383 0.887866 0.123233
H -1.628604 -1.877280 2.244075
H -0.150827 -2.007954 4.235900
H 0.058325 -0.436937 3.427386
H 1.281387 -1.706253 3.240015
S 3.499143 0.349886 -0.652945
N 2.723337 -1.148350 -0.827237
C 2.264581 1.549185 -0.149760
C 1.522210 2.217772 -1.125284
H 1.706019 2.022356 -2.176326
C 0.572321 3.156401 -0.727382
H 0.004469 3.691901 -1.484627
C 0.349193 3.434896 0.629533
C -0.706134 4.429091 1.051324
C 1.118749 2.756960 1.585249
H 0.975226 2.975495 2.640595
C 2.074146 1.816615 1.206736
H 2.685103 1.313196 1.948029
O 4.435719 0.181093 0.459823
O 3.926990 0.702972 -2.005184
C 2.394103 -1.966766 0.283684
C 2.329117 -3.449464 -0.047724
H 1.992547 -4.018599 0.824774
H 3.324005 -3.809819 -0.336365
H 1.635881 -3.647763 -0.870495
H -0.437961 4.925764 1.989422
H -1.671166 3.929826 1.212104
H -0.859383 5.200068 0.289195
H 2.039122 -1.163710 -1.587313
H 1.209608 -1.627286 0.654989
H -0.421713 -3.134569 2.002311
H 3.040747 -1.730186 1.131651

51

S -0.874724 -1.905412 -0.474027
N -0.074062 -1.447267 0.942992
C -2.319113 -0.844139 -0.504113
C -3.586411 -1.413609 -0.383290
H -3.682603 -2.487498 -0.264124
C -4.709058 -0.586904 -0.430452
H -5.698901 -1.027185 -0.339062
C -4.583010 0.797763 -0.599543
C -5.802373 1.684599 -0.681024
C -3.293639 1.341621 -0.719303
H -3.177265 2.414439 -0.855293
C -2.161629 0.534014 -0.675129
H -1.170420 0.962887 -0.774895
O -1.350110 -3.299366 -0.430141
O 0.011162 -1.480672 -1.578007
C -0.392774 -2.230018 2.151108
C -1.529486 -1.593884 2.961142
H -5.637982 2.640830 -0.172440
H -6.677850 1.205873 -0.231493
H -6.052868 1.912741 -1.725448
H -0.628633 -3.274779 1.913355
H -1.656402 -2.133472 3.906968
H -2.478770 -1.631690 2.418869
H -1.303188 -0.546173 3.182182
S 3.550517 0.376232 -0.533948
N 2.795863 -1.122785 -0.770980
C 2.290378 1.556235 -0.047362
C 1.644911 2.311155 -1.028354
H 1.916823 2.189463 -2.071384
C 0.679108 3.239517 -0.642247
H 0.186549 3.841322 -1.402241
C 0.345016 3.421846 0.707614
C -0.724661 4.406356 1.115840
C 1.017897 2.656346 1.671159
H 0.786139 2.797601 2.723952
C 1.987439 1.726727 1.305078
H 2.524037 1.157985 2.056646
O 4.446797 0.186770 0.608068
O 4.024154 0.767080 -1.860335
C 2.457787 -1.987839 0.300935
C 2.438033 -3.457802 -0.087560
H 2.080365 -4.067088 0.748583
H 3.450281 -3.786944 -0.351962
H 1.781180 -3.633019 -0.944651
H -0.485311 4.885813 2.070826
H -1.692490 3.902284 1.240115
H -0.856352 5.191315 0.364337
H 2.141144 -1.136024 -1.555471
H 1.263186 -1.692303 0.650507
H 0.521302 -2.224264 2.757812
H 3.076208 -1.767284 1.174171

51

S -0.874766 -1.905385 -0.474094
N -0.074141 -1.447360 0.942986
C -2.319137 -0.844086 -0.504128
C -3.586451 -1.413557 -0.383484
H -3.682666 -2.487463 -0.264486
C -4.709083 -0.586829 -0.430611
H -5.698939 -1.027110 -0.339369
C -4.583002 0.797863 -0.599477
C -5.802345 1.684741 -0.680817
C -3.293615 1.341718 -0.719068
H -3.177214 2.414555 -0.854892

C -2.161621 0.534089 -0.674930
H -1.170398 0.962963 -0.774569
O -1.350174 -3.299336 -0.430331
O 0.011158 -1.480575 -1.578016
C -0.392899 -2.230203 2.151029
C -1.529646 -1.594134 2.961065
H -5.638109 2.640670 -0.171607
H -6.677953 1.205753 -0.231818
H -6.052526 1.913506 -1.725176
H -0.628747 -3.274946 1.913188
H -1.303368 -0.546435 3.182182
H -1.656589 -2.133786 3.906851
H -2.478911 -1.631915 2.418757
S 3.550532 0.376144 -0.533855
N 2.795831 -1.122842 -0.770923
C 2.290418 1.556208 -0.047350
C 1.645076 2.311179 -1.028378
H 1.917048 2.189475 -2.071390
C 0.679301 3.239603 -0.642331
H 0.186829 3.841439 -1.402356
C 0.345131 3.421948 0.707503
C -0.724419 4.406608 1.115702
C 1.017876 2.656373 1.671093
H 0.786038 2.797620 2.723869
C 1.987378 1.726696 1.305074
H 2.523870 1.157895 2.056674
O 4.446739 0.186651 0.608213
O 4.024262 0.766970 -1.860215
C 2.457722 -1.987916 0.300965
C 2.437956 -3.457870 -0.087566
H 2.080268 -4.067172 0.748557
H 3.450205 -3.787018 -0.351960
H 1.781115 -3.633059 -0.944672
H -1.692021 3.902449 1.241352
H -0.856938 5.190811 0.363558
H -0.484318 4.887062 2.070005
H 2.141123 -1.136047 -1.555423
H 1.263119 -1.692380 0.650521
H 0.521153 -2.224491 2.757770
H 3.076130 -1.767390 1.174218

51

S -0.869846 -1.910023 -0.490444
N -0.033368 -1.343665 0.855918
C -2.336190 -0.876031 -0.471097
C -2.219294 0.495001 -0.718931
H -1.245843 0.934140 -0.908150
C -3.366756 1.281199 -0.718353
H -3.281981 2.347517 -0.915255
C -4.632874 0.723310 -0.474752
C -5.871992 1.585526 -0.508471
C -4.718524 -0.652164 -0.226443
H -5.689721 -1.102570 -0.036928
C -3.579271 -1.457761 -0.224162
H -3.645443 -2.525858 -0.047021
O -1.321896 -3.308657 -0.378123
O -0.040626 -1.521120 -1.649895
C -0.443717 -1.870315 2.167741
C 0.013796 -3.290476 2.524461
H -6.197325 1.764909 -1.541688
H -5.691914 2.565288 -0.052594
H -6.705298 1.112039 0.019813
H -0.056336 -1.156063 2.903732
H 1.107372 -3.353167 2.552739
H -0.362487 -4.017476 1.801529
H -0.359315 -3.558243 3.519942
S 3.436714 0.602649 -0.598452
N 2.733114 -0.912870 -0.874282
C 2.149310 1.707011 -0.016429
C 1.882668 1.799862 1.350728
H 2.467147 1.220685 2.057411
C 0.885498 2.666848 1.790090
H 0.680787 2.747583 2.854777
C 0.149577 3.445487 0.885441
C -0.953244 4.356041 1.369369
C 0.451242 3.345428 -0.480729
H -0.088836 3.962158 -1.195258
C 1.443605 2.480950 -0.939817
H 1.690214 2.423198 -1.994640
O 4.382163 0.400830 0.501038
O 3.844748 1.071014 -1.921650
C 2.505733 -1.860492 0.157146
C 2.558938 -3.300585 -0.331285
H 3.572838 -3.535425 -0.677025
H 1.863178 -3.462860 -1.159485
H 2.296480 -3.987689 0.478410
H -1.910847 3.819723 1.410008
H -1.086636 5.214911 0.703577
H -0.749661 4.733771 2.376568
H 2.047675 -0.926462 -1.631943
H 1.330389 -1.647264 0.580729
H -1.540389 -1.813085 2.227097
H 3.161698 -1.655622 1.006544

51

S 1.108232 -1.747302 -0.420645
N 0.266310 -1.119845 0.906109
C 2.620043 -0.785147 -0.406463
C 2.567493 0.588634 -0.661382
H 1.613841 1.069733 -0.851174
C 3.750543 1.319983 -0.662085
H 3.717221 2.388959 -0.859518
C 4.988591 0.703334 -0.416096
C 6.266508 1.506782 -0.451216
C 5.009330 -0.673539 -0.161435
H 5.958099 -1.168056 0.031393
C 3.833349 -1.424353 -0.154628
H 3.847808 -2.492855 0.031955
O 0.317080 -1.355045 -1.605085
O 1.473897 -3.164289 -0.243352
C 0.697888 -1.665666 2.203589
C -0.023317 -0.939623 3.336383
H 7.081451 0.987794 0.062635
H 6.136595 2.487643 0.019194
H 6.589826 1.684714 -1.485257
H 0.544687 -2.752769 2.262337
H 0.335554 -1.301125 4.306444
H -1.104601 -1.107656 3.293197
H 0.153519 0.138614 3.275793
S -3.441793 -0.050829 -1.011112
N -2.531579 -1.487228 -0.945126
C -2.422171 1.270396 -0.354064
C -1.472085 1.874412 -1.182181
H -1.360148 1.547526 -2.210365
C -0.697988 2.914210 -0.675490
H 0.027579 3.401409 -1.323022
C -0.850580 3.358576 0.647722
C 0.016328 4.469246 1.190536
C -1.818407 2.741635 1.450356
H -1.967845 3.087328 2.470210
C -2.607097 1.701829 0.959092
H -3.378397 1.249532 1.573089
O -3.600910 0.183741 -2.445270
O -4.583079 -0.221926 -0.112134
C -2.100617 -2.164549 0.230114
C -3.075011 -2.327070 1.379622
H -3.360102 -1.374940 1.831166
H -2.615992 -2.958071 2.147149
H -3.995604 -2.811576 1.034146
H -0.436355 4.939273 2.069061
H 0.188127 5.247254 0.438780
H 1.000311 4.086854 1.492823
H -1.813211 -1.484545 -1.673108
H -1.034147 -1.575238 0.649892
H 1.782126 -1.505257 2.296666
H -1.669196 -3.119341 -0.089770

51

S -2.528690 -1.262639 -1.131036
N -1.976014 -2.151942 0.179160
C -3.172105 0.322426 -0.574750
C -2.307416 1.398085 -0.359476
H -1.244308 1.312286 -0.552948
C -2.830315 2.606811 0.094981
H -2.154051 3.441251 0.262903
C -4.203065 2.764302 0.333689
C -4.758387 4.090407 0.795657
C -5.048367 1.669819 0.104736
H -6.116961 1.772757 0.277833
C -4.544521 0.451682 -0.347358
H -5.204050 -0.387087 -0.542778
O -3.664131 -1.997098 -1.703428
O -1.331860 -1.003709 -1.959907
C -2.700909 -2.031967 1.448023
C -2.200770 -3.080311 2.439368
H -5.717120 3.967533 1.309591
H -4.926667 4.763663 -0.055335
H -4.067593 4.597730 1.477435
H -2.621589 -1.023361 1.880495
H -1.148067 -2.914616 2.694029
H -2.301648 -4.086338 2.020916
H -2.780166 -3.026640 3.367511
S 1.616232 0.585000 0.428847
N 1.100664 -0.793202 -0.405153
C 3.389576 0.599727 0.186182
C 4.223752 0.090990 1.181235
H 3.797507 -0.285887 2.104686
C 5.603364 0.101383 0.978314
H 6.257898 -0.286701 1.754650
C 6.160088 0.611488 -0.201606
C 7.652990 0.598106 -0.426783
C 5.295248 1.127840 -1.179636
H 5.709638 1.543427 -2.094895
C 3.916084 1.126809 -0.996107
H 3.254670 1.544911 -1.747691
O 1.055869 1.726639 -0.304561
O 1.360758 0.374985 1.857289
C 0.664511 -1.968036 0.271039
C 0.919270 -3.251021 -0.500757
H 0.471906 -3.196006 -1.497793
H 0.473156 -4.100118 0.025504
H 1.997492 -3.425359 -0.608470
H 8.199484 0.465862 0.511969
H 7.995635 1.528645 -0.892325
H 7.942080 -0.223262 -1.095486
H 0.571999 -0.571093 -1.253810
H -0.625428 -1.952754 0.330909
H -3.765961 -2.205707 1.238058
H 1.009671 -1.972680 1.306375

51

S 2.365676 1.341140 -0.668987
N 1.369554 1.448936 0.689154
C 3.432756 -0.024293 -0.214517
C 2.871962 -1.233067 0.214037
H 1.796935 -1.332833 0.327351
C 3.718812 -2.293741 0.516056
H 3.290127 -3.234448 0.853281
C 5.113659 -2.172360 0.396970
C 6.012722 -3.343832 0.712693
C 5.643480 -0.948495 -0.028160
H 6.720700 -0.834208 -0.120883
C 4.812551 0.129908 -0.335045
H 5.220956 1.079954 -0.662439
O 3.206466 2.535394 -0.852739
O 1.502932 0.905321 -1.788851
C 1.677757 2.555974 1.604115
C 0.760607 2.495156 2.823023
H 7.064510 -3.044263 0.751077
H 5.918356 -4.129224 -0.048263
H 5.753624 -3.797966 1.676156
H 2.723428 2.442404 1.922849
H 1.036878 3.275403 3.540778
H 0.836767 1.520832 3.314881
H -0.287697 2.652902 2.545066
S -1.626830 -0.977638 -0.502150
N -1.270888 0.557285 -1.154436
C -3.370557 -0.816796 -0.127406
C -3.790327 -0.801987 1.201176
H -3.057554 -0.877714 1.997355
C -5.154596 -0.700765 1.479378
H -5.487292 -0.688314 2.514169
C -6.099880 -0.620606 0.450325
C -7.576499 -0.530316 0.752470
C -5.646335 -0.643975 -0.879662
H -6.367329 -0.586542 -1.691588
C -4.291977 -0.744528 -1.176769
H -3.948375 -0.772272 -2.205652
O -1.445219 -1.895764 -1.627747
O -0.920723 -1.190098 0.768243
C -1.089551 1.679204 -0.292511
C -1.123430 3.004955 -1.030825
H -0.897227 3.828959 -0.346051
H -2.116217 3.178308 -1.464699
H -0.386322 3.016586 -1.839784
H -8.052090 0.275329 0.181386
H -7.758447 -0.345654 1.815380
H -8.090747 -1.461927 0.483942
H -0.512195 0.458679 -1.837885
H 0.080700 1.570232 0.231229
H 1.618946 3.535857 1.109278
H -1.767221 1.628915 0.564879

51

S -1.270400 -1.247205 -0.528841
N -0.419375 -0.672174 0.791145
C -2.960819 -0.631583 -0.460346
C -3.950196 -1.378884 0.181925
H -3.706798 -2.351119 0.597117
C -5.250049 -0.876950 0.249858
H -6.020514 -1.460272 0.748184
C -5.582432 0.358017 -0.321666
C -6.998462 0.880539 -0.274945
C -4.572730 1.084357 -0.972215
H -4.814250 2.037580 -1.436630
C -3.269432 0.599347 -1.047492
H -2.501176 1.149882 -1.580640
O -1.347445 -2.710864 -0.393554
O -0.639517 -0.629542 -1.703499
C -0.942272 -0.956736 2.134255
C -0.383074 -2.272229 2.691166
H -7.530294 0.657292 -1.209167
H -7.020643 1.967635 -0.142785
H -7.567428 0.426019 0.542114
H -0.607369 -0.126775 2.770011
H -0.728700 -2.416789 3.721978
H 0.710982 -2.251448 2.686328
H -0.712116 -3.117860 2.082955
S 2.682009 2.248019 -0.183857
N 1.363845 2.240099 0.893282
C 3.294139 0.571477 -0.276274
C 4.452062 0.234903 0.427414
H 4.972325 0.990038 1.006544
C 4.929256 -1.072667 0.353877
H 5.833594 -1.340354 0.894730
C 4.265775 -2.044572 -0.407070
C 4.795424 -3.455403 -0.499071
C 3.103380 -1.674089 -1.100835
H 2.567311 -2.413973 -1.689409
C 2.611948 -0.373455 -1.048235

H 1.707993 -0.112866 -1.587399
O 2.114034 2.615210 -1.479439
O 3.689862 3.058148 0.501627
C 0.025319 1.947807 0.535144
C -1.004228 2.656765 1.400982
H -2.012455 2.326855 1.132667
H -0.941539 3.742645 1.259499
H -0.852327 2.446583 2.466848
H 5.288040 -3.627589 -1.464931
H 3.987152 -4.190134 -0.414774
H 5.528676 -3.662096 0.286665
H 1.636501 2.236263 1.871025
H -0.184109 0.700980 0.634745
H -2.041493 -0.956260 2.173666
H -0.105536 2.113801 -0.537082

51

S 1.182149 -1.701611 -0.517169
N 0.337063 -1.269947 0.875576
C 2.607468 -0.615246 -0.428546
C 3.883816 -1.173029 -0.363377
H 3.996752 -2.251718 -0.352957
C 4.992848 -0.327450 -0.322515
H 5.989681 -0.758615 -0.273849
C 4.844096 1.064745 -0.346041
C 6.048031 1.975820 -0.330201
C 3.545688 1.597568 -0.406360
H 3.412399 2.676904 -0.420538
C 2.427752 0.771289 -0.449671
H 1.427665 1.189386 -0.490642
O 0.322499 -1.264146 -1.636920
O 1.689125 -3.086259 -0.521274
C 0.818854 -1.830514 2.149545
C 0.590622 -3.326514 2.406455
H 5.878114 2.854122 0.302264
H 6.275266 2.343548 -1.339469
H 6.938779 1.457902 0.038427
H 1.893802 -1.607574 2.224422
H -0.477251 -3.570173 2.396710
H 0.980524 -3.588774 3.396966
H 1.093711 -3.939406 1.656381
S -3.415098 0.021209 -0.998959
N -2.491960 -1.403875 -0.957484
C -2.459179 1.330137 -0.229907
C -2.731111 1.706855 1.085409
H -3.524032 1.211982 1.635808
C -2.003315 2.748624 1.658785
H -2.220214 3.050513 2.680430
C -1.011411 3.423599 0.935830
C -0.211900 4.537054 1.568880
C -0.770830 3.033925 -0.391384
H -0.025557 3.565959 -0.978362
C -1.482932 1.991985 -0.979425
H -1.303922 1.708296 -2.010992
O -3.502565 0.321989 -2.426974
O -4.599489 -0.205537 -0.170973
C -2.109474 -2.157753 0.188762
C -3.080481 -2.255695 1.349290
H -2.685328 -2.961834 2.085653
H -4.057266 -2.613795 1.007173
H -3.239362 -1.294991 1.844052
H 0.028388 5.320956 0.842753
H 0.739270 4.159722 1.967529
H -0.754120 4.997969 2.400413
H -1.760044 -1.379694 -1.671895
H -1.009599 -1.690196 0.605363
H 0.327277 -1.237048 2.929054
H -1.781270 -3.141032 -0.167330

51

S 1.987409 -1.037245 1.066496
N 1.807693 -2.052912 -0.234309
C 2.700186 0.505749 0.478446
C 2.043225 1.245850 -0.511289
H 1.109543 0.892033 -0.939411
C 2.611120 2.438288 -0.949640
H 2.106593 3.013445 -1.722473
C 3.821539 2.910453 -0.416165
C 4.408817 4.219474 -0.887429
C 4.457795 2.145767 0.568687
H 5.398888 2.490761 0.989850
C 3.907533 0.944996 1.019085
H 4.401444 0.350280 1.779795
O 2.950043 -1.639169 1.990370
O 0.607081 -0.740812 1.511618
C 2.998769 -2.533519 -0.951605
C 2.605475 -3.677482 -1.883570
H 5.457191 4.319205 -0.590061
H 3.862557 5.071965 -0.462983
H 4.354075 4.313016 -1.977805
H 3.478111 -1.723882 -1.522961
H 1.874372 -3.346915 -2.630232
H 2.166308 -4.504168 -1.317247
H 3.488210 -4.046145 -2.417073
S -1.929406 -1.136089 -1.068787
N -1.692182 -2.112597 0.288081
C -2.716522 0.387622 -0.537553
C -4.008957 0.673155 -0.974813
H -4.512609 -0.013163 -1.646686
C -4.626788 1.847849 -0.543024
H -5.633267 2.075922 -0.885080
C -3.974595 2.735078 0.320866
C -4.638559 4.013375 0.774056
C -2.676519 2.414530 0.752930
H -2.157292 3.086979 1.431783
C -2.041432 1.250428 0.333669
H -1.044479 1.007851 0.689502
O -0.564556 -0.798454 -1.527332
O -2.861966 -1.837236 -1.951166
C -2.739307 -2.785600 0.917889
C -2.854998 -2.764151 2.403730
H -1.879927 -2.904279 2.890088
H -3.517814 -3.565132 2.746493
H -3.264066 -1.811729 2.785537
H -5.703232 4.023840 0.521855
H -4.176425 4.888376 0.298910
H -4.545114 4.149520 1.857602
H -0.912315 -1.772758 0.863671
H 1.024440 -1.751619 -0.824394
H 3.712227 -2.880561 -0.199487
H -3.529543 -3.136742 0.267497

51

S -1.129558 -1.641973 -0.201756
N -0.429311 -0.974850 1.178211
C -2.647511 -0.708010 -0.353745
C -2.597361 0.684956 -0.463291
H -1.644354 1.201475 -0.411638
C -3.784744 1.390083 -0.626456
H -3.754460 2.474115 -0.707250
C -5.022698 0.728460 -0.690425
C -6.298836 1.508720 -0.896122
C -5.040019 -0.666817 -0.578369
H -5.988562 -1.195942 -0.621279
C -3.859887 -1.392765 -0.412562
H -3.870548 -2.473899 -0.326932
O -1.492655 -3.053846 -0.007766
O -0.218762 -1.275443 -1.303922
C -0.913773 -1.469156 2.482018
C -0.202613 -0.715784 3.602733
H -6.405173 1.817104 -1.944335
H -6.313158 2.420553 -0.288864
H -7.179695 0.913434 -0.637348
H -1.992142 -1.281467 2.526064

H -0.567899 -1.056988 4.577069
H -0.375541 0.361683 3.519971
H 0.879133 -0.891310 3.569639
S 3.499595 -0.054100 -1.126506
N 2.700580 -1.504629 -0.776417
C 2.529496 1.271380 -0.400570
C 1.519577 1.874846 -1.152789
H 1.339094 1.552271 -2.172362
C 0.776724 2.907545 -0.583968
H 0.007392 3.397577 -1.176616
C 1.016764 3.340356 0.729061
C 0.187430 4.443018 1.343363
C 2.038349 2.717784 1.459302
H 2.254314 3.053631 2.470713
C 2.798161 1.689203 0.904351
H 3.612097 1.235858 1.459942
O 4.771831 -0.109908 -0.404486
O 3.415902 0.085493 -2.579081
C 2.750999 -2.084825 0.500970
C 2.231663 -3.485265 0.634032
H 2.208016 -3.778939 1.688802
H 2.878423 -4.205169 0.104813
H 1.220277 -3.605653 0.227930
H 0.748554 4.987638 2.109516
H -0.712692 4.038027 1.825060
H -0.144028 5.163554 0.588303
H 1.822881 -1.601260 -1.294894
H 0.590134 -1.053188 1.081040
H -0.765210 -2.552183 2.582087
H 3.632819 -1.800133 1.067045

51

S -1.140436 -1.630597 -0.229755
N -0.400725 -0.973897 1.127585
C -2.615912 -0.628451 -0.384049
C -2.506217 0.761940 -0.484053
H -1.533583 1.238219 -0.415182
C -3.660380 1.517709 -0.657758
H -3.582968 2.599960 -0.731296
C -4.924545 0.909870 -0.741364
C -6.163641 1.745460 -0.955968
C -5.002037 -0.483910 -0.638820
H -5.971381 -0.972311 -0.697089
C -3.855636 -1.260210 -0.463780
H -3.913427 -2.340412 -0.386264
O -1.576952 -3.020184 -0.026091
O -0.220859 -1.322074 -1.341318
C -0.906726 -1.336919 2.465342
C -0.401869 -2.672928 3.012898
H -6.228838 2.090541 -1.996006
H -6.159846 2.637943 -0.320231
H -7.073473 1.177576 -0.739492
H -0.618782 -0.514488 3.130364
H 0.692481 -2.676954 3.079349
H -0.713990 -3.499819 2.370122
H -0.799148 -2.841469 4.020710
S 3.474851 0.180744 -1.154199
N 2.719572 -1.319224 -0.963665
C 2.498142 1.381805 -0.243753
C 2.732570 1.574714 1.119320
H 3.527651 1.027801 1.614919
C 1.963713 2.505090 1.817041
H 2.152358 2.665203 2.875889
C 0.968499 3.253131 1.173325
C 0.128227 4.247340 1.938916
C 0.765290 3.049435 -0.200097
H 0.018508 3.642194 -0.723845
C 1.516345 2.116163 -0.912100
H 1.367607 1.971242 -1.976710
O 4.769106 0.069716 -0.479518
O 3.344043 0.498902 -2.575041
C 2.827799 -2.058254 0.225302
C 2.338329 -3.474521 0.186051
H 2.990007 -4.106585 -0.440556
H 1.324009 -3.558340 -0.220605
H 2.335051 -3.902580 1.193764
H -0.815417 3.793419 2.270008
H -0.128207 5.114811 1.321482
H 0.647636 4.607987 2.832379
H 1.827497 -1.381727 -1.461649
H 0.615153 -1.061931 1.024405
H -2.000629 -1.325910 2.414913
H 3.724757 -1.830550 0.793471

51

S 2.555396 -1.160240 -0.828082
N 1.717825 -1.559146 0.563472
C 3.367137 0.368269 -0.367659
C 2.605691 1.453199 0.081956
H 1.529158 1.369443 0.198137
C 3.255578 2.644160 0.387067
H 2.670115 3.489693 0.740276
C 4.647743 2.775577 0.248321
C 5.328613 4.081288 0.582918
C 5.379717 1.673519 -0.207389
H 6.457742 1.755458 -0.321322
C 4.748831 0.468300 -0.519850
H 5.313311 -0.386678 -0.875611
O 1.500272 -0.876934 -1.824489
O 3.607209 -2.130943 -1.160553
C 2.415335 -2.204031 1.688271
C 2.595960 -3.716775 1.549092
H 4.969337 4.891795 -0.063263
H 6.413656 4.011617 0.460444
H 5.124513 4.382144 1.617672
H 3.383493 -1.705234 1.802500
H 3.091835 -4.122136 2.439029
H 3.200487 -3.956622 0.670443
H 1.624846 -4.216754 1.450971
S -1.648967 0.122942 0.414769
N -1.280254 -0.803690 -0.972139
C -3.387116 0.500066 0.200552
C -4.281837 0.184333 1.220091
H -3.925540 -0.323978 2.109471
C -5.626185 0.531017 1.074051
H -6.327861 0.286256 1.867405
C -6.084628 1.188674 -0.072897
C -7.541881 1.548557 -0.237102
C -5.156765 1.501234 -1.081006
H -5.493708 2.016487 -1.977331
C -3.813682 1.166295 -0.952227
H -3.103487 1.413585 -1.734433
O -1.516444 -0.678708 1.638617
O -0.878489 1.362514 0.234979
C -1.360845 -2.210218 -0.973089
C -2.611313 -2.912013 -0.558712
H -3.468661 -2.645785 -1.200348
H -2.894218 -2.677581 0.474098
H -2.467559 -3.994501 -0.628741
H -7.662746 2.599277 -0.525155
H -8.102278 1.384951 0.688207
H -8.012210 0.943550 -1.022776
H -0.397554 -0.459234 -1.365523
H 0.810070 -1.964694 0.328603
H 1.830257 -1.962354 2.582326
H -0.711365 -2.648217 -1.724164

51

S 1.424204 -0.232170 0.467764
N 1.053733 1.399793 0.265446
C 3.165353 -0.297478 0.068405
C 3.580005 -0.040935 -1.242228
H 2.851749 0.212494 -2.005431
C 4.933430 -0.122243 -1.548124

H 5.262478 0.075609 -2.565311
C 5.882460 -0.459912 -0.567953
C 7.345441 -0.566401 -0.925120
C 5.436352 -0.713257 0.734324
H 6.155710 -0.977321 1.505172
C 4.081618 -0.637845 1.061562
H 3.733067 -0.845333 2.067425
O 0.660791 -0.917311 -0.592546
O 1.269535 -0.659389 1.866371
C 1.341165 2.299396 1.399928
C 1.048202 3.741845 0.997685
H 7.975400 -0.604735 -0.031436
H 7.670668 0.283766 -1.535211
H 7.541469 -1.475351 -1.508301
H 0.768680 2.015905 2.292374
H 1.280055 4.417156 1.827966
H -0.010585 3.873691 0.746092
H 1.647355 4.035403 0.130169
S -3.105172 -1.736625 -0.893236
N -1.884223 -2.336638 0.141558
C -3.132977 0.048330 -0.667412
C -2.340452 0.855601 -1.487151
H -1.754833 0.403456 -2.280735
C -2.335230 2.236177 -1.283391
H -1.740373 2.869570 -1.938143
C -3.107869 2.824562 -0.268848
C -3.141700 4.324303 -0.089641
C -3.882972 1.988261 0.546584
H -4.489574 2.424871 1.336595
C -3.904006 0.607619 0.353651
H -4.529128 -0.030120 0.969147
O -2.622676 -2.000180 -2.249591
O -4.386072 -2.255066 -0.408352
C -1.985842 -2.293225 1.539947
C -3.011613 -3.094609 2.272010
H -4.018369 -2.951525 1.869188
H -3.011475 -2.821174 3.332486
H -2.796382 -4.175571 2.207229
H -3.983528 4.765526 -0.639514
H -2.227907 4.797339 -0.464498
H -3.263404 4.601324 0.962991
H -0.977191 -2.032198 -0.222977
H 0.088877 1.456200 -0.068232
H 2.403159 2.187230 1.643552
H -1.067823 -1.993157 2.035525

51

S 2.525320 -1.259944 -0.880979
N 1.749977 -1.732854 0.534720
C 3.330970 0.280295 -0.451820
C 4.700885 0.406243 -0.675013
H 5.261850 -0.435504 -1.066366
C 5.324706 1.621339 -0.388682
H 6.393109 1.724906 -0.560996
C 4.597557 2.706996 0.112847
C 5.275956 4.015970 0.439653
C 3.216738 2.550214 0.318302
H 2.633822 3.384266 0.701587
C 2.573485 1.348825 0.039264
H 1.504461 1.248978 0.202315
O 1.426897 -0.960547 -1.824917
O 3.570136 -2.218345 -1.262980
C 2.446846 -2.637190 1.473815
C 3.433863 -1.903819 2.378744
H 6.300825 4.043744 0.057184
H 5.321271 4.177176 1.524453
H 4.730796 4.865080 0.011612
H 1.651328 -3.083132 2.079113
H 3.856528 -2.603522 3.108703
H 2.934851 -1.092758 2.917913
H 4.263724 -1.477285 1.806931
S -1.699028 0.058670 0.442650
N -1.336093 -0.887000 -0.932004
C -3.428732 0.464158 0.213471
C -4.339548 0.152257 1.219752
H -4.000459 -0.366947 2.109551
C -5.677483 0.517758 1.060529
H -6.391705 0.276086 1.843555
C -6.113489 1.190501 -0.086317
C -7.563458 1.572561 -0.264308
C -5.169580 1.498762 -1.080880
H -5.488979 2.025625 -1.976882
C -3.832783 1.144982 -0.938975
H -3.110150 1.389751 -1.710540
O -1.587508 -0.734615 1.673115
O -0.906118 1.283038 0.255911
C -1.411318 -2.293950 -0.915907
C -2.654358 -2.995148 -0.478373
H -2.925821 -2.748556 0.554502
H -2.507297 -4.077890 -0.536814
H -3.519684 -2.740132 -1.113893
H -8.141141 1.390260 0.646744
H -8.027633 0.995965 -1.074527
H -7.666922 2.632491 -0.524367
H -0.461868 -0.542119 -1.342394
H 0.807339 -2.043531 0.294217
H 2.949767 -3.448426 0.932724
H -0.774639 -2.735939 -1.675714

51

S -1.981582 -0.603274 1.307700
N -1.990992 -1.837986 0.183696
C -2.633230 0.790829 0.392750
C -3.694410 1.516396 0.929265
H -4.135460 1.208097 1.871005
C -4.171514 2.630324 0.236026
H -5.000269 3.199789 0.649245
C -3.601768 3.024415 -0.979851
C -4.129573 4.219104 -1.737976
C -2.530512 2.272944 -1.492737
H -2.075759 2.564873 -2.436461
C -2.039310 1.159242 -0.819861
H -1.215513 0.580453 -1.227466
O -0.554870 -0.341285 1.600605
O -2.905498 -0.882611 2.413368
C -3.162388 -2.725897 0.113254
C -3.021351 -3.657509 -1.087666
H -4.845349 4.790546 -1.139309
H -4.639045 3.908742 -2.659247
H -3.318100 4.894692 -2.032528
H -3.289475 -3.299349 1.040928
H -3.893343 -4.316638 -1.155648
H -2.130032 -4.289317 -0.995438
H -2.938138 -3.086402 -2.017283
S 2.043942 -1.118964 -0.944786
N 1.687497 -1.911216 0.540239
C 2.921455 0.309090 -0.316183
C 2.211907 1.371484 0.249583
H 1.130498 1.333082 0.330794
C 2.918677 2.476844 0.716528
H 2.373662 3.305701 1.161437
C 4.316517 2.543433 0.616522
C 5.070437 3.738264 1.149629
C 4.996851 1.467402 0.030331
H 6.079074 1.506658 -0.065644
C 4.310524 0.346991 -0.434216
H 4.834526 -0.485352 -0.891466
O 2.975375 -1.971400 -1.688210
O 0.805051 -0.661607 -1.595943
C 1.112188 -3.200853 0.551103
C 1.715585 -4.335391 -0.213561
H 2.767088 -4.503248 0.070644
H 1.711260 -4.167367 -1.294962
H 1.157575 -5.253114 -0.002337
H 5.271542 3.627584 2.223434
H 4.498572 4.663265 1.021065

H 6.035326 3.860792 0.647516
H 1.138432 -1.262095 1.115163
H -1.081047 -2.305819 0.147820
H -4.051192 -2.096673 -0.001156
H 0.640906 -3.406119 1.509524

51

S -1.805486 1.439162 1.019349
N -1.240234 2.462129 -0.222154
C -2.996196 0.435542 0.158474
C -2.564597 -0.520946 -0.766752
H -1.510926 -0.673395 -0.979345
C -3.521296 -1.296169 -1.413504
H -3.191930 -2.043666 -2.130601
C -4.892074 -1.137365 -1.152124
C -5.908578 -2.001644 -1.858131
C -5.288350 -0.172657 -0.216868
H -6.344821 -0.038724 0.000694
C -4.349830 0.618142 0.444093
H -4.654654 1.360248 1.173770
O -2.493860 2.203927 2.067919
O -0.622797 0.624325 1.379684
C -0.598295 3.663287 0.285274
C -0.124140 4.541571 -0.868831
H -5.811960 -3.051044 -1.552629
H -5.769345 -1.970304 -2.945027
H -6.931484 -1.682162 -1.637687
H -1.296078 4.200927 0.945813
H 0.362807 5.442695 -0.480775
H -0.967521 4.847487 -1.495462
H 0.590791 4.004647 -1.500213
S 1.526268 -2.048829 -0.539255
N 0.833791 -1.987470 0.977680
C 2.824893 -0.804563 -0.602113
C 2.490889 0.529874 -0.851171
H 1.458238 0.804230 -1.040642
C 3.499296 1.489124 -0.875468
H 3.244026 2.526288 -1.081709
C 4.840845 1.142371 -0.651401
C 5.920544 2.198443 -0.655746
C 5.148464 -0.202537 -0.411055
H 6.183641 -0.494423 -0.251261
C 4.151280 -1.177832 -0.383648
H 4.394979 -2.222375 -0.221406
O 2.175089 -3.355735 -0.668371
O 0.472290 -1.613366 -1.466412
C 1.641284 -2.345916 2.153242
C 0.738625 -2.431380 3.382306
H 1.329316 -2.699940 4.264857
H -0.039643 -3.187145 3.238301
H 0.251549 -1.469812 3.580331
H 6.907444 1.762309 -0.839472
H 5.966902 2.719562 0.309794
H 5.735945 2.957307 -1.423894
H 0.284220 -1.131815 1.088570
H 2.449573 -1.618292 2.327925
H 0.246560 3.367402 0.930989
H 2.102593 -3.315211 1.946872

51

S -2.622303 -1.395324 -0.618949
N -1.882222 -1.908299 0.812190
C -3.361105 0.225924 -0.428290
C -2.550820 1.366067 -0.431742
H -1.473512 1.279268 -0.531491
C -3.152132 2.613499 -0.285916
H -2.528221 3.503828 -0.282076
C -4.542534 2.743375 -0.145805
C -5.178792 4.107132 -0.023911
C -5.326042 1.581344 -0.145223
H -6.404841 1.662407 -0.037959
C -4.746550 0.321238 -0.283720
H -5.353356 -0.577799 -0.295865
O -3.698981 -2.360668 -0.866823
O -1.508796 -1.269562 -1.580135
C -2.004127 -1.090050 2.002930
C -1.443361 -1.833936 3.211638
H -6.164199 4.050095 0.448965
H -5.314897 4.565380 -1.012379
H -4.555525 4.788331 0.565097
H -1.440637 -0.156364 1.834364
H -0.383076 -2.051078 3.058564
H -1.981327 -2.772929 3.375532
H -1.545449 -1.213396 4.108667
S 1.640643 -0.307649 0.513790
N 1.399298 -1.235607 -0.856130
C 3.258461 0.410109 0.227509
C 4.242531 0.283325 1.205078
H 4.032530 -0.282461 2.106206
C 5.484310 0.888233 1.001702
H 6.255077 0.790169 1.762059
C 5.753329 1.616809 -0.162188
C 7.102088 2.257169 -0.388725
C 4.740181 1.730717 -1.129443
H 4.930942 2.294123 -2.039877
C 3.497612 1.135570 -0.943823
H 2.722014 1.221500 -1.697643
O 0.622340 0.758528 0.445848
O 1.743000 -1.122860 1.735996
C 1.912657 -2.619719 -0.843104
C 1.661436 -3.259768 -2.205812
H 0.589906 -3.283630 -2.432150
H 2.032692 -4.290247 -2.210342
H 2.168173 -2.701907 -2.999860
H 7.721849 2.216439 0.512034
H 7.000153 3.308384 -0.682445
H 7.650423 1.750359 -1.193151
H 0.407074 -1.179841 -1.113150
H 1.442776 -3.207731 -0.044901
H -3.052158 -0.786059 2.159612
H 2.987273 -2.577677 -0.633714

51

S -1.822498 1.163448 1.168023
N -0.933037 2.064619 0.036842
C -2.991638 0.296062 0.144434
C -4.333079 0.676161 0.199478
H -4.641062 1.484656 0.853301
C -5.257693 -0.004937 -0.590546
H -6.304934 0.283689 -0.552552
C -4.859881 -1.054786 -1.428320
C -5.865474 -1.802921 -2.269716
C -3.502185 -1.412131 -1.457902
H -3.173011 -2.225637 -2.099290
C -2.559888 -0.750486 -0.677040
H -1.518034 -1.054063 -0.713858
O -2.551846 2.060337 2.076049
O -0.849046 0.206859 1.739260
C -0.158902 3.138680 0.649374
C -0.767762 4.494479 0.240617
H -5.516866 -1.912026 -3.302919
H -6.833306 -1.292905 -2.288865
H -6.029665 -2.814709 -1.877469
H -0.093766 3.072378 1.741543
H -0.124652 5.302563 0.605138
H -1.761493 4.610483 0.681228
H -0.850705 4.571518 -0.847374
S 1.693454 -2.111475 -0.357123
N 1.167929 -1.974182 1.217248
C 2.745236 -0.697359 -0.726930
C 2.177476 0.478583 -1.223765
H 1.110697 0.529103 -1.415558
C 3.001184 1.569624 -1.495516
H 2.563608 2.477124 -1.905805

C 4.385000 1.512272 -1.272486
C 5.264012 2.709859 -1.544720
C 4.929362 0.318359 -0.780542
H 6.002378 0.245552 -0.619834
C 4.121845 -0.785324 -0.509151
H 4.553278 -1.718041 -0.161535
O 2.543902 -3.301792 -0.427987
O 0.485716 -1.969181 -1.182570
C 2.133486 -2.044075 2.322507
C 1.383407 -2.173830 3.646884
H 2.095053 -2.224097 4.478210
H 0.769715 -3.079745 3.655242
H 0.726349 -1.312862 3.813046
H 6.274383 2.407084 -1.838370
H 5.360805 3.340091 -0.650455
H 4.851779 3.337210 -2.341815
H 0.459770 -1.242754 1.311100
H 2.789625 -1.159404 2.344543
H 0.852358 3.053303 0.231451
H 2.761309 -2.922298 2.149892

51

S 2.942022 -1.201254 0.318635
N 2.465211 -1.564221 -1.269888
C 3.415386 0.507228 0.145364
C 2.475427 1.448228 -0.290737
H 1.463719 1.152247 -0.553308
C 2.869423 2.778097 -0.393364
H 2.148790 3.515785 -0.737682
C 4.173115 3.184560 -0.063311
C 4.569473 4.637678 -0.167517
C 5.086498 2.216328 0.371697
H 6.100315 2.512375 0.628810
C 4.718345 0.875558 0.478311
H 5.424539 0.122555 0.810749
O 4.113844 -1.982345 0.740363
O 1.702717 -1.293417 1.126724
C 2.503339 -2.977211 -1.606806
C 1.155726 -3.657012 -1.262403
H 5.648669 4.770113 -0.045096
H 4.070465 5.238079 0.603962
H 4.282985 5.059656 -1.137698
H 2.655542 -3.019510 -2.691204
H 1.159705 -4.670850 -1.676663
H 0.317806 -3.097417 -1.686533
H 1.023406 -3.718606 -0.179093
S -1.686408 -0.314678 -0.570025
N -1.258334 -0.810120 0.977249
C -3.336756 0.337678 -0.312337
C -4.400980 -0.184372 -1.043623
H -4.225781 -1.003420 -1.732727
C -5.675432 0.359404 -0.871665
H -6.509018 -0.047509 -1.438617
C -5.897549 1.417306 0.016804
C -7.273643 2.014230 0.192107
C -4.804661 1.924695 0.740132
H -4.959352 2.745153 1.437039
C -3.528419 1.397014 0.580245
H -2.688675 1.791256 1.142861
O -0.773326 0.795569 -0.884488
O -1.810266 -1.446009 -1.505702
C -1.790148 -2.106852 1.439217
C -1.338969 -2.353548 2.876620
H -1.734812 -3.309587 3.236305
H -1.693924 -1.557480 3.538895
H -0.246017 -2.392472 2.939785
H -7.548084 2.080597 1.251424
H -8.037068 1.419367 -0.318256
H -7.317382 3.032241 -0.215631
H -0.235242 -0.768009 1.040507
H -1.468617 -2.927795 0.784953
H 3.328835 -3.499730 -1.107391
H -2.883562 -2.056181 1.387753

51

S -3.124621 -0.897386 -0.694505
N -3.696953 -1.580531 0.758041
C -3.050127 0.832208 -0.285014
C -2.031358 1.304309 0.547213
H -1.280533 0.637133 0.955624
C -1.981160 2.663227 0.836808
H -1.181037 3.033883 1.471560
C -2.927970 3.555705 0.309988
C -2.838879 5.031367 0.615476
C -3.936895 3.050170 -0.520286
H -4.675961 3.728187 -0.939384
C -4.005665 1.692082 -0.826648
H -4.778402 1.301756 -1.479834
O -4.106370 -1.095245 -1.769973
O -1.751002 -1.423785 -0.854133
C -4.107156 -2.966825 0.603074
C -4.573436 -3.535726 1.939826
H -3.745563 5.561638 0.308622
H -1.990907 5.488339 0.089579
H -2.686026 5.208173 1.686156
H -4.899965 -3.023611 -0.159659
H -4.884611 -4.578805 1.817250
H -5.422332 -2.965137 2.328983
H -3.768545 -3.498790 2.680229
S 1.688782 0.336922 0.029815
N 1.262525 -1.152746 -0.633135
C 3.479003 0.239258 0.036420
C 4.167251 0.286694 1.246397
H 3.611811 0.353633 2.175649
C 5.563045 0.249865 1.236073
H 6.103953 0.284430 2.178506
C 6.276290 0.170821 0.034811
C 7.786254 0.146331 0.023331
C 5.555148 0.127606 -1.170651
H 6.093328 0.066358 -2.113688
C 4.165413 0.164462 -1.179628
H 3.613558 0.134265 -2.113327
O 1.254227 1.340479 -0.948718
O 1.255135 0.457466 1.435678
C 1.321897 -2.320848 0.268356
C 1.019601 -3.589246 -0.525088
H 1.077881 -4.465583 0.130154
H 1.735178 -3.717286 -1.343504
H 0.011190 -3.547811 -0.950225
H 8.167452 -0.677683 -0.591113
H 8.193515 0.032119 1.032446
H 8.193056 1.075137 -0.396587
H 0.328585 -1.041354 -1.039917
H 0.624511 -2.212117 1.108753
H -3.263911 -3.549119 0.195325
H 2.334755 -2.367462 0.683498

51

S -2.040388 1.607225 0.484632
N -2.350900 2.796654 -0.697407
C -2.940091 0.217011 -0.137269
C -2.442801 -0.497518 -1.228529
H -1.500189 -0.225400 -1.689501
C -3.157760 -1.600612 -1.682619
H -2.771665 -2.168289 -2.524968
C -4.347836 -2.005834 -1.058221
C -5.084242 -3.232786 -1.537502
C -4.816653 -1.269164 0.039993
H -5.732617 -1.575880 0.538888
C -4.123096 -0.156138 0.508645
H -4.473931 0.404006 1.368454
O -2.585225 1.977148 1.806058
O -0.587258 1.322604 0.367959
C -2.185593 4.164973 -0.216494

C -0.750931 4.667442 -0.515695
H -4.650049 -4.141365 -1.098662
H -5.022227 -3.338261 -2.625308
H -6.140979 -3.204771 -1.254527
H -2.903579 4.759676 -0.790367
H -0.519037 4.557641 -1.578606
H -0.017474 4.101400 0.065118
H -0.683997 5.726016 -0.244233
S 1.582858 -1.896030 -0.053736
N 0.733757 -1.223061 1.216066
C 3.015024 -0.868862 -0.354777
C 4.273298 -1.294976 0.071013
H 4.376442 -2.264287 0.547832
C 5.379970 -0.475994 -0.149008
H 6.363127 -0.811621 0.172176
C 5.247615 0.765675 -0.785485
C 6.450338 1.655290 -0.996115
C 3.970495 1.166299 -1.205889
H 3.851366 2.121894 -1.711283
C 2.854286 0.359884 -0.997770
H 1.872198 0.676625 -1.334596
O 2.069122 -3.211435 0.383822
O 0.684998 -1.762154 -1.214724
C 1.336894 -1.184351 2.554221
C 0.281963 -0.809760 3.584296
H -0.527628 -1.547066 3.589701
H -0.152531 0.175063 3.378001
H 0.733624 -0.776597 4.581828
H 6.723192 2.177789 -0.070413
H 6.257739 2.416783 -1.758438
H 7.326180 1.076576 -1.309439
H 0.283794 -0.355957 0.927184
H 2.194152 -0.487743 2.583426
H -2.401511 4.274168 0.853495
H 1.737699 -2.177454 2.757591

51

S 2.318041 -0.995322 0.119632
N 3.571052 -1.191071 1.251765
C 2.498446 0.725877 -0.303143
C 2.910513 1.663276 0.648126
H 3.191236 1.345903 1.645903
C 2.976308 3.005270 0.282341
H 3.304612 3.739620 1.013632
C 2.627392 3.424862 -1.009694
C 2.725405 4.878621 -1.404531
C 2.203643 2.460480 -1.935006
H 1.913717 2.767882 -2.936233
C 2.137278 1.112001 -1.596184
H 1.791137 0.374868 -2.310838
O 1.071441 -1.211387 0.897053
O 2.547502 -1.811505 -1.077981
C 3.878375 -2.587651 1.509844
C 4.954354 -2.707898 2.584462
H 3.672572 5.081713 -1.921633
H 2.681751 5.536384 -0.530755
H 1.917051 5.161875 -2.086748
H 2.955934 -3.112507 1.809647
H 5.187521 -3.762461 2.766250
H 4.617136 -2.261483 3.524959
H 5.872412 -2.200060 2.273063
S -2.003700 -0.711982 -1.395041
N -1.396116 -2.079646 -0.668844
C -2.770201 0.278170 -0.098491
C -1.969096 0.877717 0.879359
H -0.891356 0.749353 0.858732
C -2.570829 1.634767 1.879330
H -1.949784 2.099866 2.641643
C -3.963736 1.811046 1.922124
C -4.597343 2.657463 3.000822
C -4.741607 1.199309 0.932820
H -5.821912 1.322400 0.948787
C -4.155213 0.433887 -0.077057
H -4.756340 -0.032982 -0.849787
O -0.834679 0.030578 -1.881230
O -3.082101 -1.128516 -2.296894
C -2.310736 -3.058358 -0.054166
C -2.738410 -4.149226 -1.034551
H -3.275141 -3.711737 -1.880058
H -3.395525 -4.871561 -0.534912
H -1.863365 -4.681604 -1.421123
H -5.678364 2.495520 3.054558
H -4.432761 3.726487 2.812327
H -4.171560 2.435482 3.986029
H -0.542239 -1.868807 -0.150095
H -3.192305 -2.556642 0.369456
H 4.193131 -3.062490 0.567031
H -1.766142 -3.494159 0.790241

51

S -2.998213 -1.199252 0.419753
N -2.554059 -1.620098 -1.163983
C -3.455249 0.508141 0.197643
C -2.513187 1.422260 -0.288337
H -1.510864 1.103453 -0.559651
C -2.893452 2.752669 -0.428231
H -2.171357 3.469691 -0.811121
C -4.185584 3.186142 -0.086863
C -4.567485 4.639269 -0.235692
C -5.101174 2.244601 0.398816
H -6.105951 2.561768 0.665989
C -4.746743 0.903592 0.543456
H -5.454862 0.171042 0.915166
O -1.747997 -1.280607 1.211075
O -4.172508 -1.951487 0.885028
C -2.613506 -3.043045 -1.453223
C -1.256640 -3.718799 -1.140462
H -4.017183 5.266432 0.477201
H -5.636178 4.794418 -0.059804
H -4.331126 5.011581 -1.239333
H -3.421427 -3.545079 -0.906520
H -1.282365 -4.746842 -1.517244
H -1.079588 -3.742016 -0.062202
H -0.433328 -3.181531 -1.618856
S 1.650890 -0.425919 -0.536790
N 1.205874 -0.915302 1.009862
C 3.254294 0.341116 -0.297472
C 4.330023 -0.071595 -1.080021
H 4.197637 -0.882824 -1.787698
C 5.562905 0.566875 -0.931591
H 6.405569 0.244786 -1.538261
C 5.732027 1.612440 -0.017271
C 7.063321 2.308633 0.136695
C 4.627812 2.009928 0.755732
H 4.741032 2.819263 1.473344
C 3.392388 1.386805 0.620246
H 2.545120 1.695457 1.223596
O 1.852720 -1.575015 -1.435764
O 0.685697 0.627157 -0.891731
C 1.609665 -2.262471 1.470977
C 3.009609 -2.287817 2.080574
H 3.092731 -1.558122 2.892454
H 3.778854 -2.059059 1.336401
H 3.221260 -3.284570 2.484456
H 7.003644 3.353794 -0.192323
H 7.843128 1.818083 -0.453518
H 7.388853 2.319829 1.183701
H 0.192034 -0.783673 1.072923
H 1.532285 -2.993206 0.656311
H -2.807977 -3.118908 -2.529188
H 0.867102 -2.536077 2.226897

TS_{INTER} *8’/9’ → 10*

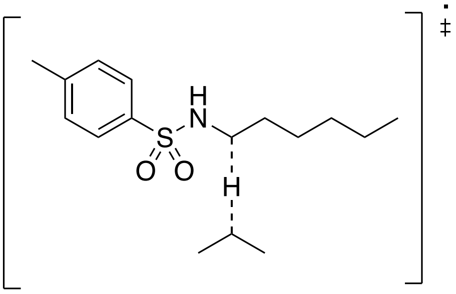

Name	E(B3LYP)	H(B3LYP)	E(RO-B2PLYP-D3)	H(RO-B2PLYP-D3)	NImag
Tosyl_NH_CH_pentane_H_propane_TS_0010	-1229.827045	-1229.388211	-1229.317937	-1228.879103	-1726.3755
Tosyl_NH_CH_pentane_H_propane_TS_0067	-1229.826444	-1229.388597	-1229.316865	-1228.879018	-1730.8136
Tosyl_NH_CH_pentane_H_propane_TS_0034	-1229.826444	-1229.387653	-1229.316908	-1228.878117	-1730.8057
Tosyl_NH_CH_pentane_H_propane_TS_0025	-1229.826447	-1229.387652	-1229.316611	-1228.877816	-1730.9027
Tosyl_NH_CH_pentane_H_propane_TS_0021	-1229.826203	-1229.387456	-1229.316186	-1228.877439	-1723.8266
Tosyl_NH_CH_pentane_H_propane_TS_0076	-1229.827836	-1229.389077	-1229.315937	-1228.877178	-1731.1853
Tosyl_NH_CH_pentane_H_propane_TS_0036	-1229.826525	-1229.387686	-1229.315177	-1228.876338	-1729.5088
Tosyl_NH_CH_pentane_H_propane_TS_0091	-1229.827234	-1229.388533	-1229.314998	-1228.876297	-1733.8578
Tosyl_NH_CH_pentane_H_propane_TS_0037	-1229.826498	-1229.38767	-1229.315087	-1228.876259	-1732.8139
Tosyl_NH_CH_pentane_H_propane_TS_0075	-1229.827006	-1229.388318	-1229.314666	-1228.875978	-1727.6535
Propyl radical (8’/9’)	-118.4781564	-118.383468	-118.3888396	-118.2941512	
Propane	-119.1442426	-119.034683	-119.054390	-118.9448304	
7-H	-1111.367419	-1111.020571	-1110.944543	-1110.597695	
10	-1110.710641	-1110.37804	-1110.288512	-1109.955911	

48

H 2.8908824757 -3.5552340019 1.1251404557
C 1.8150390584 -3.6212968945 0.9225889423
C 1.4968325633 -4.4307053498 -0.3174936729
H 1.5116881693 -2.3081647226 0.6146221326
H 0.4317282071 -4.3629974001 -0.5666108757
H 2.0766746186 -4.0940164197 -1.1864129704
H 1.7311122735 -5.4972628633 -0.1699860340
C 0.9984096361 -3.9506591072 2.1548400538
H -0.0726554196 -3.8422347328 1.9502102993
H 1.1703545635 -4.9877874458 2.4842148241
H 1.2538882974 -3.2965738046 2.9976619490
C 2.5868409513 -0.3002729279 0.2332952925
H 3.1383356110 -0.6898838732 -0.6365573693
H 3.1811663290 -0.5796932968 1.1140852227
H 0.6528515507 -0.6817835982 1.1934023073
C 1.2468342169 -1.0097794104 0.3352735265
N 0.4610950683 -0.8828278906 -0.8743755078
H 0.8934441401 -1.2626243724 -1.7154904071
S -1.1738726955 -1.3209439931 -0.8844919596
O -1.4889652468 -1.5021811425 -2.3057314612
O -1.4455753638 -2.3833699681 0.0931812492
C -1.9742005885 0.1649152503 -0.2831779804
C -2.0346361725 1.2923619459 -1.1079639265
C -2.5489168200 0.1723374222 0.9857512790
C -2.6704114675 2.4373960474 -0.6422877896
H -1.5960907133 1.2641501401 -2.1000700084
C -3.1821059761 1.3321236044 1.4369801513
H -2.5067793535 -0.7204936475 1.6000419068
C -3.2530953848 2.4772492526 0.6360967507
H -2.7208844391 3.3164246162 -1.2805115706
H -3.6314841367 1.3422801808 2.4267663547
C -3.9522829635 3.7257446128 1.1184346144
H -4.2284899802 3.6486119342 2.1742616735
H -3.3163393872 4.6106446761 0.9983357418
H -4.8705720802 3.9097785759 0.5464398855
C 2.4972576416 1.2328660089 0.1372676594
H 1.9703261669 1.6166303082 1.0229295404
H 1.8778724900 1.5038127022 -0.7275669107
C 3.8688863458 1.9107646205 0.0253354591
H 4.4873681595 1.6311725198 0.8915144827
H 4.3956477017 1.5236382714 -0.8598940724
C 3.7885215041 3.4402784657 -0.0647674230
H 3.2635860403 3.8273701364 0.8201739188
H 3.1706812542 3.7191851074 -0.9301777238
C 5.1608014989 4.1120093537 -0.1789284017
H 5.7895547674 3.8795605385 0.6894566193
H 5.6957911610 3.7714824544 -1.0740700153
H 5.0690996736 5.2024124970 -0.2409158015
48

H 1.0061578075 -4.3991125243 -0.2441136812
C 1.6505485444 -3.9803458838 0.5369820198
C 1.0614343124 -4.1405811144 1.9222712967
H 1.5504039362 -2.6316567947 0.2451315967
H 1.6534007176 -3.5990876684 2.6725936793
H 0.0340298743 -3.7651762616 1.9572444610
H 1.0449008292 -5.1981758796 2.2300078610
C 3.1051387852 -4.3708476307 0.3754623458
H 3.4759691970 -4.1682509045 -0.6366073314
H 3.7465200920 -3.8300170871 1.0837752155
H 3.2548678695 -5.4455053658 0.5666599197
C 2.7473221032 -0.7618175175 -0.4483128177
H 3.1136188111 -1.3045295124 -1.3324698342
H 3.4529545421 -0.9930533382 0.3612671249
H 0.9990152883 -0.9089647620 0.8800748630
C 1.3899310026 -1.3219195404 -0.0521175656

N 0.4016149646 -1.1783825460 -1.0995329703
H 0.5919999750 -1.6903031378 -1.9595210180
S -1.2466906276 -1.3200169305 -0.7507768868
O -1.8628894917 -1.6588769225 -2.0381285650
O -1.4583838741 -2.1509124504 0.4409908230
C -1.6951025000 0.3580387852 -0.3080640103
C -1.8057356682 1.3252615740 -1.3116806476
C -1.9383964395 0.6781638117 1.0259558181
C -2.1515388244 2.6259046312 -0.9630694254
H -1.6351734844 1.0539505739 -2.3483794001
C -2.2820443363 1.9905352070 1.3576076940
H -1.8736610051 -0.0940310778 1.7848277382
C -2.3900168836 2.9817808825 0.3755776572
H -2.2448553359 3.3796617847 -1.7413371232
H -2.4766873046 2.2430162957 2.3968891366
C -2.7502415792 4.4032262306 0.7365044766
H -3.5642846561 4.7802917977 0.1066830793
H -3.0655790007 4.4842598891 1.7810264089
H -1.8948081385 5.0758218286 0.5938938111
C 2.7774590972 0.7518331826 -0.7462905568
H 2.0769096971 0.9672551317 -1.5634825167
H 3.7793514130 1.0098338323 -1.1182246558
C 2.4433197448 1.6437033274 0.4562731546
H 1.4335068560 1.4125493869 0.8218568277
H 3.1305300438 1.4139656318 1.2850511654
C 2.5192959656 3.1422228186 0.1356725529
H 1.8448429860 3.3639231154 -0.7034942937
H 3.5320309101 3.3897040093 -0.2131912363
C 2.1560842014 4.0322822496 1.3286666974
H 2.8284281829 3.8539511581 2.1770305491
H 2.2218334999 5.0956458312 1.0707102124
H 1.1327792179 3.8332469415 1.6707635085
48

H 0.9924842207 -4.1516388358 -0.2713373483
C 1.5290262264 -3.7777021264 0.6070993803
C 0.8200895113 -4.1110282477 1.9024057947
H 1.3631152931 -2.4161373739 0.4341542800
H 1.2905758572 -3.6073270589 2.7578715121
H -0.2317414271 -3.8114393561 1.8600617336
H 0.8528136919 -5.1922969339 2.1105037412
C 3.0156844245 -4.0646468812 0.5617373191
H 3.2187501950 -5.1432082142 0.6588611358
H 3.4698508317 -3.7348346749 -0.3805984895
H 3.5466119348 -3.5666566540 1.3838257662
C 2.5124726655 -0.4081108508 0.1873467582
H 3.0491981309 -0.7516728397 -0.7104725995
H 3.1065041805 -0.7562571311 1.0432457125
H 0.5752617771 -0.8040114400 1.1353153655
C 1.1560711850 -1.0898056692 0.2536888784
N 0.3671620729 -0.8679015377 -0.9401691711
H 0.7853840105 -1.2108833546 -1.8040043244
S -1.2764395066 -1.2756415726 -0.9632086673
O -1.5886371222 -1.4328900817 -2.3880865760
O -1.5672482544 -2.3426780669 0.0027329085
C -2.0569382199 0.2164297504 -0.3510428473
C -2.1066594916 1.3496002134 -1.1685781415
C -2.6242632830 0.2244939709 0.9212404947
C -2.7239042459 2.5005503506 -0.6924919472
H -1.6747836723 1.3213023087 -2.1636100661
C -3.2387665448 1.3901038212 1.3830282704
H -2.5914809807 -0.6724660617 1.5300430233
C -3.2985890708 2.5409338476 0.5893947342
H -2.7658463148 3.3839529743 -1.3252861834
H -3.6822895700 1.4004895885 2.3754774458
C -3.9792266113 3.7953637299 1.0827949610
H -3.3423821418 4.6765182729 0.9424898590
H -4.9108198396 3.9810404453 0.5331137706
H -4.2297265866 3.7245748987 2.1454921487
C 2.4570617966 1.1302562764 0.1918151457
H 1.9469348993 1.4670487453 1.1059549705
H 1.8361929636 1.4701459670 -0.6471492239
C 3.8428843790 1.7829589409 0.1099235391
H 4.4628102280 1.4329685278 0.9490691413
H 4.3522600135 1.4433059717 -0.8044990307
C 3.7972858415 3.3165377787 0.1214421439
H 3.2904087281 3.6562008047 1.0358828608
H 3.1774656925 3.6657946587 -0.7165813934
C 5.1836740699 3.9630530004 0.0358216219
H 5.8155028514 3.6597852263 0.8797923696
H 5.7015889081 3.6701481878 -0.8858222052
H 5.1168538467 5.0569677698 0.0465408993
48

H 0.4171536450 -4.0619457110 0.8575107841
C 1.4658398349 -3.7581283493 0.7966492454
C 2.2076959106 -3.9053301127 2.1086948186
H 1.2982162335 -2.4042351587 0.5855954834
H 3.2139158485 -3.4683180160 2.0552373981
H 1.6720515531 -3.4205300275 2.9335947098
H 2.3346394423 -4.9649053076 2.3824741194
C 2.1693036928 -4.3014899969 -0.4298553577
H 1.5963921601 -4.1091715338 -1.3451157330
H 3.1674480559 -3.8597325134 -0.5526413522
H 2.3099249705 -5.3921457310 -0.3633597290
C 2.4733317240 -0.4299656772 0.2451402941
H 2.9947356190 -0.8202789851 -0.6425167040
H 3.0695739867 -0.7520869829 1.1097950580
H 0.5392620526 -0.7545433475 1.2278207811
C 1.1066300505 -1.0853308596 0.3530929311
N 0.3087426604 -0.9023660323 -0.8411382645
H 0.7161551326 -1.2792317828 -1.6958062464
S -1.3402846660 -1.2899359654 -0.8356009560
O -1.6637400571 -1.5003037590 -2.2512343110
O -1.6371158027 -2.3121286456 0.1752673234
C -2.0970525918 0.2362708149 -0.2800970215
C -2.1367547153 1.3361527032 -1.1425046772
C -2.6578390337 0.3027566686 0.9932875456
C -2.7371050183 2.5130641735 -0.7105451344
H -1.7107909178 1.2620413620 -2.1377300208
C -3.2553687485 1.4938587061 1.4104420452
H -2.6336963759 -0.5695889079 1.6372919923
C -3.3045502270 2.6124636756 0.5714444905
H -2.7716334691 3.3705430531 -1.3784986825
H -3.6941819307 1.5497444321 2.4034626198
C -3.9641728994 3.8955014007 1.0175910310
H -4.2446620837 3.8561208737 2.0743742965
H -3.2995338060 4.7558319633 0.8756072990
H -4.8747367707 4.0937803029 0.4381375778
C 2.4447965540 1.1079682188 0.1852268957
H 1.9481822510 1.4918981271 1.0881213653
H 1.8232475123 1.4233935968 -0.6627528778
C 3.8411319153 1.7318522272 0.0655733984
H 4.4613412628 1.4070689405 0.9145950512
H 4.3373257980 1.3444305990 -0.8370344180
C 3.8224449925 3.2650867107 0.0116848279
H 3.3281361354 3.6524554744 0.9139553340
H 3.2028861651 3.5892040307 -0.8365581536
C 5.2193292190 3.8825366141 -0.1103402698
H 5.1718013777 4.9769269655 -0.1460295045
H 5.8516489365 3.6043073922 0.7418428176
H 5.7254617547 3.5413494888 -1.0218642758
48

H 3.0377715464 -3.8473828218 0.8266051214
C 1.9424931246 -3.7962219016 0.8426765800
C 1.2928806459 -4.7009472280 -0.1837523577
H 1.7182341835 -2.5022007185 0.4116026331
H 1.7150277272 -4.5535100028 -1.1860409774
H 1.4369143239 -5.7632146021 0.0711772080
H 0.2124950569 -4.5231989469 -0.2349939350
C 1.3643465229 -3.8614083323 2.2410147490
H 0.2915636735 -3.6373641048 2.2277890628
H 1.4882918064 -4.8642067496 2.6798282678
H 1.8543923062 -3.1492601093 2.9163139573
C 2.8494047651 -0.6650390554 -0.4192469859
H 3.2362597975 -1.2622564149 -1.2586218120
H 3.5523146238 -0.8214960929 0.4105768279
H 1.0927735786 -0.7561028788 0.9015744260
C 1.5028035480 -1.2294530505 0.0065138498

N 0.5215774838 -1.1857167497 -1.0564698561
H 0.7428775290 -1.7336984730 -1.8864016396
S -1.1231828673 -1.3754142448 -0.7144068198
O -1.7257477885 -1.7160568554 -2.0076193284
O -1.3242823229 -2.2282806470 0.4647272882
C -1.6069093265 0.2869391073 -0.2505753138
C -1.8935880552 0.5756433557 1.0819897294
C -1.7046351083 1.2721985560 -1.2378523687
C -2.2682637359 1.8752357297 1.4294571079
H -1.8357210788 -0.2106902462 1.8267621287
C -2.0813654461 2.5599419186 -0.8735354506
H -1.4988480618 1.0250001000 -2.2741792378
C -2.3637410085 2.8845128417 0.4645598031
H -2.4958600178 2.1036947500 2.4675877560
H -2.1644826069 3.3279514232 -1.6388625981
C -2.7534136217 4.2932527388 0.8442095003
H -3.1349948216 4.3417100902 1.8684819905
H -1.8932195068 4.9718304106 0.7776726328
H -3.5266894518 4.6873752790 0.1749904839
C 2.8473944961 0.8230858262 -0.8252056426
H 2.1478061926 0.9633815100 -1.6594861316
H 3.8455295214 1.0772603805 -1.2096535935
C 2.4848465599 1.7909850317 0.3083773877
H 1.4792011433 1.5616080048 0.6863270548
H 3.1731807912 1.6389485013 1.1539261087
C 2.5255963146 3.2638179606 -0.1196517365
H 1.8492345743 3.4077231857 -0.9741020841
H 3.5332898627 3.5103413370 -0.4834410902
C 2.1362824026 4.2287792580 1.0048891969
H 1.1173890055 4.0287457617 1.3596138316
H 2.8102433125 4.1299031952 1.8648509542
H 2.1756508126 5.2718736792 0.6702472676
48

H 2.8539239247 -3.6962663516 1.1315336933
C 1.7814150763 -3.7192921661 0.9034934711
C 1.4653325794 -4.4743622103 -0.3709505175
H 1.5277693446 -2.3869524887 0.6363515207
H 0.4087401135 -4.3635753763 -0.6401847985
H 2.0748304997 -4.1269430224 -1.2149802750
H 1.6621369613 -5.5524689638 -0.2562842024
C 0.9264265927 -4.0636577940 2.1052612508
H 1.1837296238 -3.4481278289 2.9760894461
H -0.1355829145 -3.9123401176 1.8813772130
H 1.0566046989 -5.1167701480 2.4013504375
C 2.6650039822 -0.3931135435 0.3676030657
H 3.2313108953 -0.7556474033 -0.5044429048
H 3.2279493733 -0.7272934349 1.2499901950
H 0.6923093428 -0.7625902037 1.2503790275
C 1.3059985862 -1.0722833294 0.3994583553
N 0.5635585233 -0.8802436146 -0.8288516879
H 1.0102520207 -1.2456186683 -1.6687907401
S -1.0833713552 -1.2635081583 -0.9041180879
O -1.3548405808 -1.4069417468 -2.3386184241
O -1.4214057491 -2.3341065932 0.0433327523
C -1.8577260318 0.2358789594 -0.3021742914
C -2.4570756147 0.2451073759 0.9553228818
C -1.8702963905 1.3738717755 -1.1144652364
C -3.0660768182 1.4171937724 1.4080385431
H -2.4530532490 -0.6556643417 1.5593802628
C -2.4828771639 2.5310769479 -0.6476271378
H -1.4139422067 1.3441719020 -2.0984720682
C -3.0890324051 2.5729833707 0.6197085775
H -3.5345953394 1.4287299619 2.3888966662
H -2.4965121988 3.4182033151 -1.2764431194
C -3.7624520326 3.8351114766 1.1032288893
H -4.6666396703 4.0486070841 0.5191090011
H -4.0569878593 3.7556546947 2.1539218026
H -3.1016716371 4.7039234361 1.0013018974
C 2.6133035775 1.1448164514 0.3408670536
H 2.0818014155 1.5001393449 1.2360717696
H 2.0129283388 1.4636945778 -0.5192426782
C 4.0073892600 1.7858468913 0.2896961398
H 4.6043734546 1.4043227129 1.1306236231
H 4.5252199143 1.4563552827 -0.6238438098
C 4.0069579182 3.3228392891 0.3425046863
H 5.0451364025 3.6698484416 0.4328193394
H 3.4964388000 3.6518281089 1.2590600323
C 3.3610425811 4.0001176275 -0.8721920848
H 2.2975840105 3.7517652525 -0.9615500311
H 3.4388327971 5.0911325453 -0.8016730763
H 3.8530889095 3.6917322551 -1.8033690123
48

H 2.9215653047 -3.6115270804 1.0691321440
C 1.8363754403 -3.6508421210 0.9153739317
C 1.4399427957 -4.4937463439 -0.2790268308
H 1.5592574280 -2.3405591894 0.5724400535
H 0.3672895700 -4.4021633387 -0.4842661304
H 1.9903356934 -4.2064079964 -1.1840666719
H 1.6491802386 -5.5610852679 -0.1023860136
C 1.0651287550 -3.9100373535 2.1928012841
H 1.3767005297 -3.2345158837 2.9990490988
H -0.0101822879 -3.7754501366 2.0302532235
H 1.2203379070 -4.9395816111 2.5528451212
C 2.6740820882 -0.3794862045 0.0757071710
H 3.1780317654 -0.8154072834 -0.8010257997
H 3.2965383046 -0.6450972217 0.9412653638
H 0.7723292309 -0.6705222950 1.1274461382
C 1.3202578338 -1.0452202002 0.2578906866
N 0.4881054440 -0.9369043583 -0.9221453951
H 0.8730223933 -1.3589291586 -1.7661360950
S -1.1580584170 -1.3234973825 -0.8488361070
O -1.5376374938 -1.5481377624 -2.2479432890
O -1.4199529753 -2.3390944032 0.1799023254
C -1.8885086848 0.2081737459 -0.2738632576
C -1.9509603861 1.3048300608 -1.1389962609
C -2.4080008906 0.2816663655 1.0166237517
C -2.5322221689 2.4859815735 -0.6925351020
H -1.5561928580 1.2254319790 -2.1465944342
C -2.9868165315 1.4768685692 1.4479685871
H -2.3659029211 -0.5880460585 1.6632326380
C -3.0582965817 2.5924471511 0.6063885398
H -2.5839270765 3.3413113750 -1.3620927425
H -3.3927921701 1.5386183660 2.4545026231
C -3.6999231876 3.8791244320 1.0679407167
H -3.0506602335 4.7408048241 0.8736966727
H -4.6435334942 4.0616399374 0.5381389943
H -3.9200088326 3.8560353039 2.1394163368
C 2.6232731637 1.1516365570 -0.0717222957
H 2.1379077861 1.5746711710 0.8179590909
H 1.9809764824 1.4101269205 -0.9239913723
C 4.0122703297 1.7766004465 -0.2641948392
H 4.6445776825 1.5349981247 0.6036135160
H 4.4949014599 1.3034961464 -1.1315574651
C 4.0049651094 3.3001455865 -0.4745363022
H 3.3757207666 3.5409569186 -1.3433925612
H 5.0216364901 3.6214664742 -0.7381742772
C 3.5291756323 4.1070686246 0.7395164772
H 3.5980316618 5.1839504594 0.5473619713
H 2.4870267623 3.8856473640 0.9957110752
H 4.1418551424 3.8877109956 1.6231520957
48

H 1.0107650087 -4.4005542647 -0.2452546157
C 1.6508143457 -3.9810745841 0.5390350772
C 1.0554288883 -4.1427189135 1.9214823793
H 1.5500909696 -2.6324619128 0.2471871059
H 0.0273843832 -3.7686721306 1.9518651046
H 1.0388898274 -5.2004776330 2.2286529138
H 1.6431854502 -3.6007792255 2.6747830739
C 3.1067360446 -4.3693393429 0.3842342335
H 3.2571844778 -5.4438172221 0.5758697839
H 3.4820078319 -4.1659130740 -0.6260276314
H 3.7439546822 -3.8277196884 1.0956953031
C 2.7468033770 -0.7614255648 -0.4437060536
H 3.1161823620 -1.3045980311 -1.3262943023
H 3.4505779536 -0.9908167125 0.3680109533
H 0.9957427483 -0.9101949532 0.8808259222
C 1.3890700729 -1.3228546670 -0.0504848674

N 0.4028514702 -1.1803188422 -1.0999952574
H 0.5946530409 -1.6930935160 -1.9591582443
S -1.2461737999 -1.3203543953 -0.7542706359
O -1.8605312694 -1.6566754624 -2.0431544715
O -1.4613283736 -2.1526599984 0.4359115622
C -1.6924218492 0.3578326309 -0.3097464034
C -1.7975651926 1.3272225963 -1.3116004606
C -1.9399594096 0.6757009908 1.0242187592
C -2.1416991452 2.6280944257 -0.9613945595
H -1.6246406254 1.0576467205 -2.3483631371
C -2.2818297596 1.9879373224 1.3574080259
H -1.8801626880 -0.0984401630 1.7815112044
C -2.3833616842 2.9818113571 0.3770379495
H -2.2313191574 3.3835188354 -1.7384283594
H -2.4803164080 2.2384764642 2.3964601070
C -2.7353852534 4.4043388123 0.7417455891
H -3.4885077731 4.8165571638 0.0608048816
H -3.1266842401 4.4719593800 1.7613286745
H -1.8542780357 5.0562484626 0.6811131613
C 2.7756057642 0.7519188023 -0.7434026764
H 2.0772596068 0.9652129905 -1.5630278494
H 3.7782395127 1.0110108349 -1.1125654768
C 2.4362344648 1.6448304806 0.4569192950
H 1.4260047752 1.4118756816 0.8201629636
H 3.1217753635 1.4180123856 1.2878783928
C 2.5094316563 3.1430215010 0.1341153779
H 1.8370203173 3.3616080567 -0.7074972985
H 3.5226097069 3.3925745714 -0.2119627470
C 2.1401203786 4.0341128633 1.3244694789
H 2.2039866217 5.0972212471 1.0649699694
H 1.1162262565 3.8328893212 1.6636141798
H 2.8101190975 3.8589280090 2.1753387543
48

H 0.9732142874 -4.3883846217 -0.2302609906
C 1.6443007514 -3.9734646145 0.5300118454
C 1.0937524585 -4.1218347941 1.9323412492
H 1.5491799488 -2.6246982246 0.2353892933
H 1.0763315488 -5.1777587117 2.2457431470
H 1.7116506534 -3.5823021687 2.6629453587
H 0.0710965656 -3.7370674570 1.9944801941
C 3.0895293797 -4.3797121647 0.3284090704
H 3.2335640794 -5.4552451648 0.5191388849
H 3.4332543901 -4.1844867063 -0.6946338306
H 3.7566433183 -3.8432946774 1.0160164772
C 2.7514070022 -0.7649397811 -0.4729897715
H 3.0998042282 -1.3059438651 -1.3654150597
H 3.4659401463 -1.0084034030 0.3251286405
H 1.0172011252 -0.8981256096 0.8752273311
C 1.3944633692 -1.3143449666 -0.0611350169
N 0.3958582419 -1.1635602587 -1.0977837686
H 0.5801147377 -1.6682770688 -1.9633445875
S -1.2487859862 -1.3171958131 -0.7349006289
O -1.8726148880 -1.6707575663 -2.0146572133
O -1.4408106006 -2.1409390104 0.4650353971
C -1.7142091643 0.3586123037 -0.3018430309
C -1.8735320116 1.3087519534 -1.3148268062
C -1.9176272770 0.6949603399 1.0351450757
C -2.2301662080 2.6086626481 -0.9736260759
H -1.7288928981 1.0255425451 -2.3522600148
C -2.2720133126 2.0061473308 1.3590341570
H -1.8119887066 -0.0636296593 1.8031092584
C -2.4337312775 2.9796306044 0.3663507792
H -2.3576171590 3.3501322198 -1.7588120548
H -2.4321026217 2.2721180107 2.4008905267
C -2.8425628408 4.3905636717 0.7164289382
H -3.8880760514 4.5776555593 0.4393261156
H -2.7458964030 4.5820262654 1.7893812211
H -2.2315679059 5.1277224576 0.1832788567
C 2.7937972565 0.7502470349 -0.7613538306
H 2.0835153408 0.9797223860 -1.5662572216
H 3.7929180906 0.9993186613 -1.1466312510
C 2.4889454700 1.6370143345 0.4527352030
H 1.4784622315 1.4219211056 0.8264261235
H 3.1797796530 1.3859516475 1.2722846039
C 2.5918244654 3.1367981374 0.1458475083
H 1.9141365652 3.3807384938 -0.6844778195
H 3.6059856178 3.3669066540 -0.2107146103
C 2.2589496040 4.0221089059 1.3511253760
H 1.2353880234 3.8408321321 1.7018724877
H 2.9364116070 3.8217741376 2.1904687310
H 2.3433934797 5.0863870390 1.1027301655
48

H 0.5069654418 -4.0923213557 1.0530585730
C 1.5519949857 -3.9246636942 0.7770938230
C 2.5003591357 -4.0050444418 1.9548878317
H 1.4804457245 -2.5928853184 0.4185054599
H 2.1700948048 -3.3688722003 2.7846290589
H 2.5733126140 -5.0334125805 2.3432179178
H 3.5171424446 -3.6973477427 1.6761682912
C 1.9666738541 -4.6859571323 -0.4649600353
H 2.0084295405 -5.7707122356 -0.2770659229
H 1.2609754958 -4.5326608491 -1.2905190383
H 2.9652103012 -4.3846948135 -0.8092046154
C 2.7205283043 -0.8062118125 -0.3985813476
H 3.0517703279 -1.4064370624 -1.2591196457
H 3.4328476888 -1.0189537371 0.4103324916
H 0.9911991781 -0.8220925229 0.9626104749
C 1.3537354714 -1.2994982505 0.0495817294
N 0.3527790219 -1.1847021610 -0.9890723101
H 0.5326508478 -1.7226701882 -1.8352686145
S -1.2917585403 -1.3187790076 -0.6134769946
O -1.9224088495 -1.7054820996 -1.8804305279
O -1.4834842836 -2.1059607871 0.6104136869
C -1.7428203249 0.3725958608 -0.2282906191
C -1.9002283273 1.2936042799 -1.2681019335
C -1.9376970812 0.7495080063 1.0991213038
C -2.2462557774 2.6054808724 -0.9637320413
H -1.7621842266 0.9789131497 -2.2973280819
C -2.2813991624 2.0721141313 1.3858378745
H -1.8336170893 0.0129248410 1.8884548870
C -2.4409809643 3.0170741803 0.3656391881
H -2.3720532284 3.3244991300 -1.7697914640
H -2.4344489520 2.3698847987 2.4201282645
C -2.8384697738 4.4405246287 0.6759097549
H -2.2288698717 5.1573403487 0.1142104536
H -3.8857007630 4.6250530443 0.4035394854
H -2.7300792985 4.6644289884 1.7414511238
C 2.7967261529 0.6881556307 -0.7753968692
H 2.0880176551 0.8868842412 -1.5898447245
H 3.7994356936 0.8907730450 -1.1781770111
C 2.5186527144 1.6514768807 0.3855396156
H 1.5050539382 1.4830390511 0.7744312764
H 3.2071122909 1.4322762900 1.2161489055
C 2.6563509333 3.1276671406 -0.0095704083
H 1.9802556019 3.3391209767 -0.8500537939
H 3.6738144443 3.3111577972 -0.3833752157
C 2.3523512778 4.0904784327 1.1427538099
H 2.4612078661 5.1358226741 0.8314552165
H 1.3268598152 3.9556463972 1.5084567727
H 3.0298315569 3.9233215897 1.9893120631

8-Cl

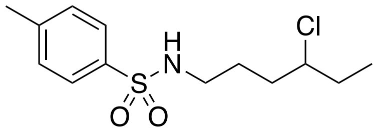

Name	E(B3LYP)	H(B3LYP)	E(RO-B2PLYP-D3)	H(RO-B2PLYP-D3)
Tosyl_NH_C5_Cl_hexane_0052	-1570.968771	-1570.629623	-1570.444011	-1570.104863
Tosyl_NH_C5_Cl_hexane_0024	-1570.968041	-1570.628793	-1570.443405	-1570.104157
Tosyl_NH_C5_Cl_hexane_0026	-1570.968031	-1570.628781	-1570.443392	-1570.104142
Tosyl_NH_C5_Cl_hexane_0097	-1570.967415	-1570.62833	-1570.441987	-1570.102902
Tosyl_NH_C5_Cl_hexane_0038	-1570.967956	-1570.628973	-1570.44157	-1570.102587
Tosyl_NH_C5_Cl_hexane_0022	-1570.966898	-1570.628056	-1570.441329	-1570.102487
Tosyl_NH_C5_Cl_hexane_0076	-1570.96704	-1570.628088	-1570.440447	-1570.101495
Tosyl_NH_C5_Cl_hexane_0093	-1570.967785	-1570.628946	-1570.439594	-1570.100755
Tosyl_NH_C5_Cl_hexane_0040	-1570.96711	-1570.628327	-1570.439135	-1570.100352

38

C -1.8996369729 -0.6583278678 2.0929518655
C -3.0467772220 0.1742646109 1.5017019026
C -3.4047090965 -0.0954263799 0.0392754869
C -4.5715048350 0.7635339267 -0.4489496978
C -0.5827900777 -0.5857493648 1.3100280003
H -2.7799108145 1.2375125294 1.5577882309
H -3.9420483580 0.0346154061 2.1205212469
Cl -3.8168710118 -1.8878024489 -0.1808420823
H -2.5371387954 0.0590405115 -0.6067002973
H -0.7330617855 -0.9683577495 0.2978618031
C -4.8712677449 0.6506462205 -1.9455284825
H -5.1736345139 -0.3657661316 -2.2149536443
H -3.9908199909 0.9126978438 -2.5438933081
H -1.7280146506 -0.3265638168 3.1263863110
H -2.1922228573 -1.7128729847 2.1501381760
S 0.7295627398 1.3064174758 -0.1771464665
O 1.0340658926 2.7170055744 0.0702107662
O -0.0558577531 0.8573423801 -1.3309980421
C 2.2880913766 0.4134684334 -0.2178278570
C 2.3747392791 -0.8026392492 -0.8982948862
C 3.3952200588 0.9353909837 0.4563305962
C 3.5803858632 -1.5025326588 -0.8895307842
H 1.5159398202 -1.1805359962 -1.4427575670
C 4.5915941849 0.2227908067 0.4529930372
H 3.3206788231 1.8958293314 0.9560416902
C 4.7046128849 -1.0048895190 -0.2167362142
H 3.6502719019 -2.4482346477 -1.4213610796
H 5.4554515373 0.6297217953 0.9731436453
C 6.0164679451 -1.7522935898 -0.2409766406
H 6.6596361284 -1.3894768444 -1.0535787142
H 6.5707186619 -1.6202541481 0.6942133521
H 5.8646173473 -2.8246745609 -0.3993183649
H -5.6823460181 1.3314844676 -2.2262153214
H 0.2743571472 1.2176504724 2.0247882828
H -5.4652017131 0.5224452011 0.1407681408
N -0.1158622077 0.8025305175 1.1825606648
H 0.1755169438 -1.2204699689 1.7906330233
H -4.3085036514 1.8041766018 -0.2102485157
38

C -2.1602532127 -0.0753595782 2.2344396782
C -2.7644345258 0.8395737769 1.1586780091
C -3.3413294403 0.1495504766 -0.0793651528
C -3.8555940906 1.1456597081 -1.1186611557
C -0.9289090852 -0.8980923681 1.8258503165
H -1.9999759460 1.5421981248 0.8007255485
H -3.5534614575 1.4477129613 1.6185293560
Cl -4.7112852242 -0.9886770874 0.4309770642
H -2.6031344289 -0.5106873391 -0.5381224658
H -1.1340865727 -1.5271142728 0.9570827667
C -4.3235514853 0.5193272252 -2.4342462124
H -5.1771590582 -0.1470220344 -2.2776231968
H -3.5190570776 -0.0622614535 -2.8996854317
H -1.8935217978 0.5433591153 3.1036227989
H -2.9241455062 -0.7792768083 2.5859887252
S 0.6049045238 0.3962270960 -0.0608858083
O 0.4111673064 1.8512323861 -0.1615398599
O -0.1183794322 -0.5221948373 -0.9509226486
C 2.3616019472 0.0874821366 -0.2271177721
C 3.2473228036 1.1619749958 -0.2627729663
C 2.8202266192 -1.2282411176 -0.3424190351
C 4.6132778789 0.9103367215 -0.4038901339
H 2.8679945531 2.1754068135 -0.1897898599
C 4.1836836352 -1.4589734701 -0.4833158458
H 2.1166469315 -2.0542195057 -0.3283976993
C 5.1022816135 -0.3959430115 -0.5156391862
H 5.3077066588 1.7462091462 -0.4317786705
H 4.5446919428 -2.4807383030 -0.5735069884
C 6.5781755402 -0.6656469554 -0.6860395923
H 6.9276091175 -1.4311724011 0.0164077521
H 6.7979636782 -1.0322147398 -1.6968808556
H 7.1726471168 0.2385581112 -0.5246526226
H -4.6263315929 1.2982356403 -3.1427329875
H 0.4691932378 0.6441727146 2.1640297795
H -4.6586092417 1.7470440971 -0.6732185758
N 0.2854347572 -0.1176123762 1.5141379870
H -0.6606351545 -1.5712398965 2.6489621091
H -3.0210673816 1.8315591896 -1.3215159401
38

C -2.1061789729 0.5999331740 1.3169866740
C -2.2039395839 -0.6318205942 0.4097460817
C -3.6186594311 -1.1786405779 0.2171554133
C -3.6519672581 -2.4939448037 -0.5630048493
C -0.6732893016 1.1030578081 1.5172184447
H -1.6067747504 -1.4448868097 0.8492604516
H -1.7614931148 -0.4147485230 -0.5685954755
Cl -4.6559870643 0.0812855392 -0.6477226236
H -4.1148299270 -1.3036991334 1.1853653537
H -0.0376518515 0.2903156404 1.8877370289
C -5.0305074967 -3.1507545670 -0.6657669638
H -4.9603907707 -4.1080098660 -1.1934960505
H -5.7346831892 -2.5145241126 -1.2096014179
H -2.7247034104 1.4107338813 0.9121156829
H -2.5188843798 0.3660599128 2.3078519354
S 1.5122305703 2.0660089832 0.2331876716
O 1.8989321133 2.5753492555 1.5556826782
O 1.6313245279 2.9031550137 -0.9651187940
C 2.3975983018 0.5303653215 -0.0272728829
C 2.3312705035 -0.0970007312 -1.2750149243

C 3.1659332957 -0.0039549648 1.0047410967
C 3.0377613369 -1.2772160330 -1.4767022982
H 1.7397881141 0.3407891772 -2.0724278837
C 3.8682798167 -1.1902692963 0.7839133203
H 3.2160496442 0.5105489234 1.9583083294
C 3.8171859254 -1.8426839767 -0.4530715454
H 2.9897393724 -1.7687838239 -2.4455283940
H 4.4689047234 -1.6107005627 1.5866124222
C 4.5967487157 -3.1125713808 -0.6980249991
H 5.4650792634 -2.9231187086 -1.3421855692
H 4.9670409579 -3.5431916770 0.2372260844
H 3.9825278653 -3.8683048405 -1.2007661195
H -5.4508917547 -3.3472094224 0.3281201984
H -0.6470267232 2.2953133867 -0.2097274517
H -3.2350674938 -2.3226335251 -1.5633446097
N -0.1051030989 1.5526539222 0.2331986795
H -0.6514771055 1.9013685985 2.2701864500
H -2.9609138012 -3.1781741051 -0.0494148343
38

C -1.9773613298 -0.5456607554 2.0913614551
C -3.1011905814 0.1390751934 1.2994388671
C -3.3649857740 -0.4000570949 -0.1062822268
C -4.4807171297 0.3284308209 -0.8601556954
C -0.6037191842 -0.5140334449 1.4082421628
H -2.8499213921 1.2004896579 1.1872842929
H -4.0310889667 0.0871200063 1.8797206498
Cl -3.8014768095 -2.1981276053 -0.0083654015
H -2.4534019913 -0.3798149056 -0.7068548262
H -0.6561915103 -1.0276213992 0.4454628547
C -4.1190624257 1.7828927673 -1.1985270136
H -4.8828634889 2.2175645948 -1.8525116719
H -3.1557839043 1.8412207811 -1.7181312633
H -1.9019244487 -0.0625342676 3.0752556005
H -2.2332847949 -1.5949072769 2.2781970098
S 0.6379857754 1.2745821821 -0.2665005334
O 0.9092158807 2.7079203549 -0.1427123303
O -0.1552015659 0.7125907924 -1.3652368003
C 2.2182982425 0.4199496949 -0.2678308299
C 2.3284574994 -0.8384533464 -0.8628840332
C 3.3194810024 1.0156305563 0.3524687606
C 3.5517121411 -1.5055057983 -0.8217437004
H 1.4734265053 -1.2750451291 -1.3679194106
C 4.5339199443 0.3346696475 0.3825859143
H 3.2265013375 2.0066685798 0.7848289535
C 4.6707375495 -0.9337682958 -0.2008048634
H 3.6396496972 -2.4842649609 -1.2869509281
H 5.3930319961 0.7987047279 0.8610342541
C 6.0007085724 -1.6487447592 -0.1915134770
H 5.8724112435 -2.7353632272 -0.2261452318
H 6.6053195060 -1.3635553413 -1.0625952740
H 6.5828041921 -1.4018079566 0.7023809082
H -4.0554722586 2.4126437238 -0.3049184767
H 0.2042413491 1.3776035794 1.9396628638
H -4.6771715647 -0.2190350495 -1.7883954890
N -0.1687233673 0.8657799807 1.1448544160
H 0.1348441241 -1.0491344257 2.0212184789
H -5.4047526290 0.2890143380 -0.2691926958
38

C -1.9674434270 -0.4964556489 2.1104219074
C -3.0961821791 0.1642764794 1.3052136460
C -3.3631751333 -0.4115926572 -0.0852322842
C -4.4839709007 0.2937031842 -0.8535258042
C -0.5962873717 -0.4779928276 1.4218689726
H -2.8486469141 1.2232226033 1.1649748211
H -4.0238842707 0.1243834521 1.8899519570
Cl -3.7933302292 -2.2079363882 0.0603777233
H -2.4537955245 -0.4038123403 -0.6894272095
H -0.6503660942 -1.0162342529 0.4727365938
C -4.1285230478 1.7402874618 -1.2301089686
H -4.8960012952 2.1555262504 -1.8923604127
H -3.1671720943 1.7886304618 -1.7542800568
H -1.8901428625 0.0119797038 3.0813335269
H -2.2193443905 -1.5413640821 2.3249871548
S 0.6337897245 1.2708237656 -0.3025662926
O 0.9020431752 2.7074635663 -0.2161450327
O -0.1621055631 0.6793613186 -1.3837472207
C 2.2157584295 0.4193695772 -0.2869954609
C 3.3187397941 1.0342395290 0.3113595818
C 2.3279056529 -0.8511313128 -0.8549453170
C 4.5361095200 0.3592492320 0.3492706358
H 3.2262840425 2.0378148538 0.7138791101
C 3.5544305788 -1.5120487899 -0.8063908798
H 1.4738598479 -1.2991210013 -1.3515963927
C 4.6731388583 -0.9244544481 -0.2002206056
H 5.3983077743 0.8412519594 0.8038056629
H 3.6457436272 -2.4971851232 -1.2571436677
C 5.9910506432 -1.6579307451 -0.1284086284
H 6.8382239269 -0.9671419603 -0.1927038405
H 6.0868477891 -2.2022017747 0.8205408153
H 6.0867325312 -2.3908406469 -0.9357064784
H -4.0641896190 2.3928395247 -0.3530668089
H 0.2076471087 1.4291075677 1.9018017784
H -4.6816531473 -0.2779210285 -1.7668250206
N -0.1666204890 0.8959785033 1.1217522581
H 0.1460988896 -0.9949524169 2.0457094341
H -5.4058685516 0.2662321225 -0.2585627816
38

C -1.7348853264 -0.9923208239 1.8696791160
C -3.1009071594 -0.3090708750 1.6963515061
C -3.5672477113 0.0012586730 0.2705245276
C -4.9518542275 0.6473197089 0.2330761279
C -0.4997690269 -0.1533443245 1.5193691731
H -3.0915697737 0.6496676556 2.2357362082
H -3.8640917478 -0.9316597393 2.1808197019
Cl -3.5851305891 -1.5617169307 -0.7252760835
H -2.8347250486 0.6236186218 -0.2449046280
H 0.4019931363 -0.6919006754 1.8516326273
C -5.4083036067 1.1117356078 -1.1521396401
H -6.3813419352 1.6107293947 -1.0876000510
H -5.5050139637 0.2683924291 -1.8422095503
H -1.6308895976 -1.2736037545 2.9254813399
H -1.7168807395 -1.9321738700 1.3044703042
S 0.7540882049 1.1864948230 -0.4957934733
O 0.7277222412 2.3635979226 0.3749570735
O 0.5279357205 1.2658127413 -1.9394805093
C 2.3314460827 0.3641024009 -0.2400329055
C 3.0431239600 0.5779232017 0.9418724380
C 2.8200294016 -0.5037877433 -1.2206982894
C 4.2489257263 -0.0937636986 1.1416828660
H 2.6658761398 1.2779145166 1.6798526615
C 4.0260678852 -1.1647524777 -1.0043442286
H 2.2692781796 -0.6384948782 -2.1460928340
C 4.7588639511 -0.9726802526 0.1770587200
H 4.8062319325 0.0743159262 2.0600153149
H 4.4100176047 -1.8363680970 -1.7685178994
C 6.0812923632 -1.6714691979 0.3849313069
H 6.0677335531 -2.6860485512 -0.0274165074
H 6.8945883697 -1.1293579540 -0.1152622052
H 6.3375497837 -1.7383393377 1.4469193123
H -4.6946999156 1.8224987987 -1.5852916683
H -0.5519736194 -0.6518581921 -0.5325521901
H -5.6841976525 -0.0467779025 0.6650931774
N -0.4439253696 0.1535280331 0.0818597672
H -0.5261256807 0.8047480535 2.0469354395
H -4.9064151836 1.5141481019 0.9086842299
38

C -2.1168386630 -0.7493709694 1.4392095964
C -2.5994720185 0.1873960422 0.3260261887
C -4.0638386744 0.0056800671 -0.0724365699
C -4.4682944993 0.8705307347 -1.2670857946
C -0.6456787032 -0.5663819599 1.8384664177
H -2.0086210590 0.0140891294 -0.5830445560
H -2.4281324993 1.2321613804 0.6124031148
Cl -5.1605837450 0.3866651238 1.3682625295
H -4.2731101458 -1.0484827719 -0.2829483152
H -0.4311354224 -1.1787843184 2.7225017656

C -5.8811508849 0.6193295570 -1.7990162007
H -6.6385078475 0.8556734179 -1.0458129028
H -6.0133144975 -0.4291434074 -2.0934810182
H -2.7229309807 -0.5915861188 2.3394025644
H -2.2815085579 -1.7963741019 1.1406707632
S 0.8881852843 0.1788781742 -0.3219995733
O 0.6196380579 1.5073241934 0.2400285743
O 0.3872904239 -0.2049297555 -1.6510561027
C 2.6612952076 -0.0818211109 -0.3363887954
C 3.2457201343 -0.7850873102 -1.3876988430
C 3.4396647211 0.4383650585 0.7013961624
C 4.6278664271 -0.9789411188 -1.3890697426
H 2.6263530508 -1.1620024527 -2.1943803735
C 4.8152959406 0.2359789479 0.6825607304
H 2.9721281630 1.0004854844 1.5031326306
C 5.4320161910 -0.4751107524 -0.3601580987
H 5.0872432794 -1.5269829941 -2.2079018574
H 5.4243284141 0.6397431308 1.4878969068
C 6.9299300753 -0.6656085777 -0.3764620077
H 7.4428261440 0.2674046881 -0.6437066233
H 7.3051448073 -0.9685381822 0.6075945533
H 7.2298247997 -1.4267791577 -1.1031002106
H -6.0739545935 1.2398008935 -2.6809036003
H 0.1568678877 -1.8309114624 0.3549640769
H -4.3435968723 1.9265606580 -0.9974928354
N 0.3501997310 -0.9455299856 0.8183403424
H -0.4436520938 0.4735027356 2.1070897597
H -3.7371236293 0.6638141482 -2.0615049974
38

C -2.2019120831 -1.0978296154 1.2359606571
C -2.6908602950 -0.3084828143 0.0159327794
C -4.1732963925 0.0630136481 0.0468262316
C -4.6424047488 0.7527350196 -1.2348126550
C -0.7568461235 -1.6062970095 1.1295640487
H -2.5311032137 -0.9093156136 -0.8902660061
H -2.0985076831 0.6047455445 -0.1136866581
Cl -4.5140170420 1.1569650880 1.4994572257
H -4.7852643420 -0.8234333927 0.2441958553
H -0.6333554461 -2.2473482100 0.2532182048
C -6.1482325489 1.0141729661 -1.3145528244
H -6.4081566438 1.4637895965 -2.2789822903
H -6.4764350591 1.6950488717 -0.5237222587
H -2.3062190357 -0.4837796357 2.1394938343
H -2.8453337587 -1.9762447806 1.3907117075
S 0.8166959141 -0.0052907129 -0.4626346943
O 0.4880695193 1.4225009916 -0.5766455565
O 0.3715709643 -0.9814021770 -1.4649346248
C 2.6007890529 -0.1247963798 -0.3321054828
C 3.3450838920 1.0100087401 -0.0135200859
C 3.2256219512 -1.3537651617 -0.5570689148
C 4.7319281297 0.9040845041 0.0927531028
H 2.8439592858 1.9605137548 0.1348062731
C 4.6100290097 -1.4399782947 -0.4473699896
H 2.6341659343 -2.2228010299 -0.8256081076
C 5.3852530245 -0.3160085079 -0.1209183695
H 5.3153227015 1.7873500027 0.3407252499
H 5.0998541476 -2.3948618168 -0.6227847993
C 6.8893561292 -0.4188289423 -0.0338053879
H 7.3437919624 -0.3979405052 -1.0330817930
H 7.3158615031 0.4112690287 0.5378188265
H 7.1998399617 -1.3553023348 0.4423291875
H -6.7186009866 0.0822515854 -1.2160939299
H 0.1208662202 0.2398855971 1.6543146584
H -4.0840722840 1.6888514137 -1.3573476820
N 0.2721995639 -0.5556038532 1.0381828151
H -0.5191652176 -2.2108722630 2.0134148429
H -4.3412470030 0.1000018758 -2.0667931813
38

C -2.5903562060 0.7772834282 -1.6500950407
C -2.8563228037 -0.6616878394 -1.1800591876
C -2.5943322073 -0.9884176910 0.2928884433
C -2.8937442782 -2.4485529887 0.6333719859
C -1.1284360766 1.2394771048 -1.6564142707
H -2.2293747738 -1.3490950300 -1.7677349977
H -3.8982560733 -0.9179976707 -1.4110747442
Cl -3.6198120096 0.1194975030 1.3619107067
H -1.5697185044 -0.7285472961 0.5633583786
H -1.0417012647 2.1805584322 -2.2144721519
C -2.5093527767 -2.8702938180 2.0535946172
H -2.7087467139 -3.9367105824 2.2051014544
H -3.0768881092 -2.3095986955 2.8020253789
H -2.9501504973 0.8625097871 -2.6833630029
H -3.1957683497 1.4781987187 -1.0621346889
S 0.9841899603 1.9693395175 -0.1201741210
O 1.0360274636 2.5266804723 1.2349590111
O 1.3621035348 2.7713169430 -1.2911646781
C 1.9551474491 0.4638168134 -0.1683715365
C 2.6840913006 0.1487082024 -1.3134035119
C 1.9826298017 -0.3686263746 0.9543385473
C 3.4392496923 -1.0251503293 -1.3355428370
H 2.6662195142 0.8213188384 -2.1642441383
C 2.7415968055 -1.5334243113 0.9143301509
H 1.4268575504 -0.0956753492 1.8456194978
C 3.4810270914 -1.8810438655 -0.2286589220
H 4.0093854197 -1.2746967134 -2.2269420530
H 2.7681666310 -2.1829830037 1.7860733627
C 4.3219278946 -3.1352311506 -0.2452787834
H 5.2496793558 -2.9938241863 0.3242968559
H 3.7910771119 -3.9794261934 0.2086802186
H 4.6016855290 -3.4176707748 -1.2648072031
H -1.4423546188 -2.6990681547 2.2410086838
H -1.1951775185 1.9485001703 0.3222776086
H -3.9571041092 -2.6487957300 0.4502528072
N -0.6011178369 1.3839330907 -0.2860039915
H -0.5040637209 0.4992560045 -2.1717834678
H -2.3324040888 -3.0580751428 -0.0900874631
38

C -1.6333264578 0.2832470201 1.3269543305
C -2.1291483597 0.3083061669 -0.1222723202
C -2.4269419748 -1.0635650130 -0.7247498097
C -2.7864132569 -1.0026970309 -2.2101458979
C -1.2285850252 1.6624386458 1.8554020538
H -1.3578506989 0.7718878352 -0.7512215492
H -3.0253982317 0.9381115756 -0.2043193318
Cl -3.7995177170 -1.8727581912 0.2119637964
H -1.5753707170 -1.7343918211 -0.5715482190
H -0.9644438416 1.5918167595 2.9170918775
C -2.9565870897 -2.3624334179 -2.8917480313
H -3.1622033024 -2.2332246001 -3.9599378438
H -3.7846840630 -2.9263874118 -2.4528347873
H -2.4162455907 -0.1109850483 1.9846192431
H -0.7822751563 -0.4040107138 1.4170309093
S 1.4724135567 1.8607594452 1.4566914907
O 1.5354223200 1.2336947640 2.7828766684
O 2.2473623533 3.0662879926 1.1399664771
C 1.8776635583 0.5963286328 0.2509546529
C 2.1404020391 -0.7028142706 0.6808775332
C 1.9655878642 0.9374104078 -1.1016349872
C 2.4802740991 -1.6746884441 -0.2617719061
H 2.0815253043 -0.9405963290 1.7375559476
C 2.3043756819 -0.0443606841 -2.0271767164
H 1.7760765834 1.9574365773 -1.4198727389
C 2.5684164126 -1.3640669338 -1.6241534174
H 2.6846081417 -2.6894452511 0.0704347385
H 2.3719784758 0.2165307820 -3.0806687657
C 2.9662008326 -2.4111233937 -2.6369274920
H 4.0258640188 -2.3130372915 -2.9067243522
H 2.8193256823 -3.4231058669 -2.2472154055
H 2.3878875322 -2.3145190348 -3.5623337771
H -2.0476545272 -2.9692157464 -2.7971303065
H -0.1540404218 3.2984056320 1.0850745925
H -3.6950777647 -0.3996288781 -2.3311478084
N -0.1302420202 2.2806684529 1.0789019962
H -2.0743266764 2.3541116836 1.7677795170
H -1.9745289479 -0.4481005273 -2.7025895467

9-Cl

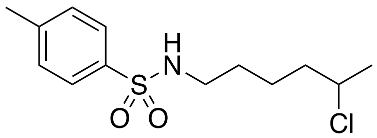

Name	E(B3LYP)	H(B3LYP)	E(RO-B2PLYP-D3)	H(RO-B2PLYP-D3)
Tosyl_NH_C6_Cl_hexane_0086	-1570.968404	-1570.629286	-1570.442276	-1570.103158
Tosyl_NH_C6_Cl_hexane_0025	-1570.967421	-1570.628416	-1570.441344	-1570.102339
Tosyl_NH_C6_Cl_hexane_0039	-1570.967557	-1570.628539	-1570.441318	-1570.1023
Tosyl_NH_C6_Cl_hexane_0029	-1570.96716	-1570.628018	-1570.441336	-1570.102194
Tosyl_NH_C6_Cl_hexane_0000	-1570.967114	-1570.628258	-1570.440984	-1570.102128
Tosyl_NH_C6_Cl_hexane_0044	-1570.967077	-1570.628205	-1570.440337	-1570.101465
Tosyl_NH_C6_Cl_hexane_0001	-1570.967712	-1570.628727	-1570.440225	-1570.10124
Tosyl_NH_C6_Cl_hexane_0071	-1570.967042	-1570.62793	-1570.440056	-1570.100944
Tosyl_NH_C6_Cl_hexane_0054	-1570.966933	-1570.627925	-1570.438295	-1570.099287

38

C -1.6023685582 1.8044320773 1.1843103487
C -2.8051758806 0.8541351423 1.0759316509
C -3.1583143753 0.4558604412 -0.3624323727
C -4.4017909925 -0.4224594630 -0.4923038208
C -0.2993748338 1.2785587756 0.5659086065
H -3.6700091374 1.3397767185 1.5418830649
H -2.5992673699 -0.0542476376 1.6555615681
H -2.3314672766 -0.1231543784 -0.7959863870
H -3.2892737992 1.3491441212 -0.9890315785
H 0.5023106482 2.0221842734 0.6880090560
C -4.6304054701 -0.9386040674 -1.9066393474
H -5.5340113034 -1.5516262180 -1.9638237745
H -3.7739048021 -1.5528109990 -2.2105175643
H -1.8329550779 2.7604412933 0.6937460002
H -1.4289349571 2.0420080497 2.2433758983
S 0.9645429046 -1.1306250069 0.2603927800
O 0.2978999744 -1.2382630056 -1.0410327482
O 1.1391168727 -2.2755827293 1.1557013059
C 2.5895555563 -0.4212621116 -0.0333018654
C 2.8200012399 0.3372882470 -1.1825622770
C 3.6029821248 -0.6150526574 0.9090533499
C 4.0745543156 0.9130540167 -1.3780887785
H 2.0332021997 0.4535204851 -1.9202816377
C 4.8501639408 -0.0331903736 0.6965048165
H 3.4177401901 -1.2310971866 1.7830614699
C 5.1068557157 0.7392409108 -0.4462642754
H 4.2564674173 1.5011821606 -2.2743006077
H 5.6410967845 -0.1870916011 1.4266635953
C 6.4731401136 1.3375402191 -0.6818667538
H 6.9486357306 1.6344621866 0.2588527472
H 7.1399708844 0.6133729868 -1.1682045249
H 6.4195619593 2.2180248704 -1.3299713150
H -4.7294017005 -0.1089791568 -2.6143958860
H 0.3810073133 0.0022551706 2.1161448974
H -4.3484957679 -1.2565930278 0.2128794760
N 0.0747157586 -0.0177688925 1.1466004416
Cl -5.9044073083 0.5108801468 0.0444983871
H -0.4330632669 1.1136827338 -0.5056959469
38

C -2.0558703129 1.9353927123 1.3512423185
C -2.7270298248 0.5711164853 1.1264503265
C -3.2510854597 0.3581443266 -0.2995300480
C -3.9336783518 -0.9893173376 -0.5272984951
C -0.7513794711 2.1993803203 0.5823372513
H -3.5618745339 0.4819458998 1.8308905802
H -2.0239062014 -0.2338813475 1.3777348798
H -2.4145577463 0.3948918892 -1.0091137152
H -3.9473935648 1.1621175161 -0.5752461857
H -0.4229861997 3.2284252392 0.7741888069
C -4.2934964213 -1.2443618071 -1.9847108361
H -4.7929797986 -2.2093747620 -2.1069718773
H -3.3763125195 -1.2493999089 -2.5863448419
H -2.7576305343 2.7390523546 1.0848447382
H -1.8552144285 2.0602792788 2.4255863478
S 0.7025982695 -0.0832940797 0.0639677942
O 0.0977381656 0.1052989493 -1.2619657534
O 0.3856837906 -1.2650346612 0.8795015105
C 2.4848861371 -0.0339204209 -0.1165110914
C 3.0611039337 0.8767914677 -1.0069717805
C 3.2740480007 -0.9170583390 0.6167938765
C 4.4437971557 0.9020629935 -1.1484636233
H 2.4323167438 1.5475584797 -1.5833143540
C 4.6608923548 -0.8762518901 0.4621503279
H 2.8050883154 -1.6280013949 1.2883083302
C 5.2662746510 0.0282222485 -0.4173916192
H 4.8959377452 1.6087458604 -1.8403456233
H 5.2799028123 -1.5638050799 1.0329661142
C 6.7651691853 0.0575459195 -0.5980288795
H 7.1541389232 1.0807871643 -0.5424808828
H 7.2730697808 -0.5398019334 0.1650603825
H 7.0520733729 -0.3430585106 -1.5787722365
H -4.9554160103 -0.4606656637 -2.3679476046
H 0.4532000779 1.1226273763 1.9384047440
H -3.3050999409 -1.7972027802 -0.1433777069
N 0.3782171549 1.3203896740 0.9427291587
Cl -5.4744770945 -1.1079295671 0.4890952801
H -0.8930254342 2.1055598801 -0.4963449483
38

C -2.3731736259 0.0650009968 2.0627770054
C -2.7487611350 -0.1872252161 0.5935412829
C -2.1979119009 0.8611461012 -0.3799804942
C -2.6050786399 0.6546476987 -1.8386326255
C -0.8705750821 0.1085224065 2.3662444792
H -3.8415787523 -0.2121929937 0.5163448923
H -2.3869519408 -1.1782293808 0.2916435626
H -1.0996101514 0.8342448599 -0.3656712330
H -2.4955653627 1.8715122921 -0.0678434324
H -0.7044811007 0.3019536193 3.4340184568
C -1.8940247508 1.5965592961 -2.8017732825
H -2.2205881354 1.4311550554 -3.8321317215
H -0.8122929026 1.4206685359 -2.7467792088
H -2.8020590689 1.0189333998 2.3987911978
H -2.8400411722 -0.7106005935 2.6864017942
S 1.4722366920 -1.2155252692 2.0328670814
O 1.7788362199 -2.6491944276 1.9952671902
O 1.9756937075 -0.3618235642 3.1168638943

C 1.9695557945 -0.4592371265 0.4850804735
C 2.6146374671 0.7763555102 0.4996681966
C 1.7298141201 -1.1313157430 -0.7168968379
C 3.0142008853 1.3483940229 -0.7090251139
H 2.8029267741 1.2714265286 1.4462457766
C 2.1349183936 -0.5458573394 -1.9123942220
H 1.2394019384 -2.0992647176 -0.7096814112
C 2.7846934892 0.7000448788 -1.9287999823
H 3.5167219187 2.3124022225 -0.7013509860
H 1.9534002129 -1.0662103742 -2.8498197393
C 3.2535448159 1.3056718126 -3.2303384574
H 4.2267480750 0.8921789596 -3.5259125292
H 2.5532223136 1.0974167849 -4.0462694095
H 3.3705600495 2.3908390605 -3.1493107494
H -2.0879203035 2.6421831675 -2.5411753357
H -0.5791105106 -1.9729454001 2.3964346945
H -2.4360251496 -0.3855579696 -2.1316134072
N -0.2185230217 -1.1396857453 1.9309723950
Cl -4.4262900714 0.8762376485 -2.0316957861
H -0.3960407519 0.9285550437 1.8173352233
38

C -2.7661677379 -1.9030651084 0.2054972590
C -3.0417423644 -0.3924026634 0.2866379935
C -2.5641600675 0.3959901630 -0.9388932389
C -2.9941047671 1.8626061349 -0.9681640085
C -1.2896804620 -2.3049960388 0.1010560759
H -2.5711998852 0.0189195408 1.1850834339
H -4.1247465491 -0.2488159989 0.4057186540
H -2.9747097101 -0.0644191553 -1.8513128546
H -1.4721224048 0.3516810003 -1.0266454926
H -0.8440053289 -1.8897637585 -0.8089772531
C -2.6076449193 2.5787488350 -2.2557614484
H -3.0970833700 2.0888885383 -3.1068872666
H -1.5247700759 2.5409188436 -2.4126245966
H -3.2034248104 -2.3899263020 1.0888899869
H -3.2848216656 -2.3338409167 -0.6622010201
S 1.1498058961 -2.0111026073 1.2458414811
O 1.5112095851 -3.2553141759 0.5519243584
O 1.5461414042 -1.7967375957 2.6417559020
C 1.7161357150 -0.6509618322 0.2253169562
C 2.5862804879 -0.9179205337 -0.8309063716
C 1.3329370826 0.6590539753 0.5283481834
C 3.0742912850 0.1437031643 -1.5933442141
H 2.8657162487 -1.9429473201 -1.0490871969
C 1.8235548091 1.7042955723 -0.2483490971
H 0.6476551346 0.8540052364 1.3460707939
C 2.7054839964 1.4656909412 -1.3151311580
H 3.7505468065 -0.0611454511 -2.4196441266
H 1.5137865277 2.7217829178 -0.0239127415
C 3.2569384375 2.6137061061 -2.1265485503
H 2.5004226726 3.3887558885 -2.2904909618
H 4.0998939589 3.0900222687 -1.6088037813
H 3.6198056010 2.2781205953 -3.1031125419
H -2.9192028734 3.6266141779 -2.2357849063
H -0.8717789079 -2.0832851720 2.1488003066
H -4.0710628817 1.9443892607 -0.7948209941
N -0.5275427383 -1.7688948768 1.2414830559
Cl -2.2543200155 2.7758161419 0.4557688095
H -1.2009476444 -3.3971142547 0.0305709340
38

C -1.7306567677 -1.7114155637 -0.2887263116
C -2.3353739798 -0.3456698975 0.0579004731
C -2.7050309428 0.4633547859 -1.1902165132
C -3.3822122107 1.8067548393 -0.9210408555
C -1.3220294039 -2.5405170342 0.9333112316
H -1.6256180113 0.2218398973 0.6677659279
H -3.2343024571 -0.4924077261 0.6758913140
H -3.4064634814 -0.1193390139 -1.8072652695
H -1.8136427443 0.6323028034 -1.8085620404
H -1.0041176266 -3.5417217467 0.6185592700
C -3.8659197274 2.5046449548 -2.1857847787
H -3.0358424078 2.6778955229 -2.8783707569
H -4.3306358747 3.4670487749 -1.9549837633
H -2.4530385044 -2.3076497565 -0.8642072435
H -0.8589808975 -1.5777640891 -0.9423965926
S 1.3538433601 -2.1231950728 1.3154565107
O 1.4892077295 -3.2991229000 0.4450757406
O 2.0805432947 -2.0316053213 2.5875547466
C 1.7368478734 -0.6806818335 0.3239411060
C 2.2134103725 -0.8532270937 -0.9735624310
C 1.6048097015 0.5961932326 0.8784456171
C 2.5483546878 0.2727138362 -1.7285798952
H 2.3173339957 -1.8540291384 -1.3785186835
C 1.9385386524 1.7054442073 0.1102732634
H 1.2403658243 0.7159334783 1.8933381905
C 2.4143004875 1.5631375493 -1.2044014324
H 2.9203292900 0.1423884410 -2.7417426401
H 1.8266593687 2.7001314419 0.5343490312
C 2.7664087543 2.7816112246 -2.0236118832
H 3.1387522939 2.5053260918 -3.0146340664
H 1.8932375702 3.4313013519 -2.1601211674
H 3.5394965019 3.3823303248 -1.5292557116
H -4.6092101888 1.8736968611 -2.6891298810
H -0.3285882017 -2.1099760954 2.7377036308
H -4.2102319741 1.6777342086 -0.2179627804
N -0.2722201446 -1.8890840139 1.7458728084
Cl -2.2265029112 2.9383667057 -0.0320041361
H -2.1824336545 -2.6595876048 1.6019616401
38

C -1.4214312734 0.5758044052 1.5619218005
C -2.0504631194 0.0868895915 0.2521617096
C -2.3885660655 1.2389078479 -0.7002341065
C -2.9747019141 0.8177177801 -2.0472204211
C -1.0482108551 -0.5504469899 2.5306359291
H -2.9621061066 -0.4820824475 0.4738814088
H -1.3592004948 -0.6070989539 -0.2424383468
H -1.4695229583 1.8022784009 -0.9226959262
H -3.0781871200 1.9451503651 -0.2187478897
H -1.9311610012 -1.1663247966 2.7358378916
C -3.1564146716 1.9778676048 -3.0176098597
H -3.8115250471 2.7446652098 -2.5915689421
H -3.5892172923 1.6402485067 -3.9632457486
H -0.5298300183 1.1803136021 1.3495549943
H -2.1215684600 1.2414505170 2.0859063514
S 1.6059324670 -1.0870757356 2.1344389121
O 2.2817206536 -2.3862172415 2.2340635865
O 1.7854765916 -0.0484280085 3.1570532428
C 2.0283970262 -0.3527442594 0.5535178068
C 2.4333518813 0.9793744260 0.5027858890
C 1.9929497392 -1.1375854965 -0.6025205679
C 2.7941610980 1.5335280305 -0.7266933742
H 2.4667244166 1.5647027761 1.4153583061
C 2.3543806149 -0.5683907869 -1.8190875299
H 1.6902321222 -2.1781225664 -0.5459411650
C 2.7624041161 0.7734402288 -1.9018121868
H 3.1103375362 2.5728413245 -0.7696123649
H 2.3259307021 -1.1752569868 -2.7209885273
C 3.1824761431 1.3673356165 -3.2252028312
H 2.5208311715 1.0461961756 -4.0373471451
H 3.1778246433 2.4612424684 -3.1942252661
H 4.1987090754 1.0499026298 -3.4929845404
H -2.1809039912 2.4346681416 -3.2268725080
H -0.1292858390 -2.4320394622 2.3166074710
H -2.3557356824 0.0369910374 -2.4986870142
N -0.0378627823 -1.4778169232 1.9748602393
Cl -4.6086721663 -0.0036084254 -1.8023543600
H -0.7060571830 -0.1295280669 3.4834445334
38

C -2.6277126088 -0.4262053894 2.0368206705
C -2.9140507716 -0.3872848670 0.5261695387
C -2.4068607256 0.8916036974 -0.1628800161
C -2.8675788524 1.0085768890 -1.6175087939
C -1.1431321864 -0.4099707089 2.4205206138
H -4.0002588334 -0.4715690835 0.3814193386
H -2.4625764442 -1.2684735890 0.0577680107
H -1.3114269165 0.9288331434 -0.1432192105

H -2.7669380680 1.7698063447 0.3875193669
H -1.0330151819 -0.3864510131 3.5125906649
C -2.3341199151 -0.0736749336 -2.5469011161
H -2.6492627558 0.1051896330 -3.5785220753
H -2.7196122214 -1.0523324672 -2.2379896013
H -3.1060970420 0.4308744177 2.5300522356
H -3.0969037257 -1.3231740363 2.4655774396
S 1.2507851264 -1.5858761260 1.9190685326
O 1.6097539874 -2.9916018682 1.7041636389
O 1.7196661329 -0.8548476088 3.1033549736
C 1.7092467427 -0.6257834112 0.4782605444
C 2.1680629892 0.6814240867 0.6375467372
C 1.6061775406 -1.2025885110 -0.7902840850
C 2.5055014858 1.4248887305 -0.4938899280
H 2.2702915994 1.0975639253 1.6340888279
C 1.9511508512 -0.4469134905 -1.9069318734
H 1.2746818609 -2.2306339527 -0.8932918983
C 2.3946075558 0.8800004031 -1.7792656543
H 2.8634285123 2.4442550787 -0.3738032341
H 1.8823575272 -0.8940328010 -2.8957134976
C 2.7213056622 1.7038502047 -3.0012110543
H 3.4476001786 2.4901644033 -2.7726337132
H 3.1301652614 1.0839549861 -3.8059362277
H 1.8194468217 2.1936709361 -3.3913026359
H -1.2401069350 -0.1088252965 -2.5148936149
H -0.7699705215 -2.4639145320 2.1781736682
H -3.9596847936 1.0457236227 -1.6649940001
N -0.4445613789 -1.5632415624 1.8264824820
Cl -2.3441465594 2.6546610314 -2.2677938795
H -0.6632894465 0.4946375284 2.0341307761
38

C -2.1450192661 0.4283880051 1.7576860000
C -3.2316524869 1.3657881704 1.2046273958
C -3.2120390542 1.5778571182 -0.3194466545
C -3.3222905243 0.3205146904 -1.1846400859
C -0.7173068339 0.9660018938 1.5941601592
H -3.1470786894 2.3499101571 1.6869879066
H -4.2112796766 0.9673821415 1.4899752827
H -2.2786605488 2.0766253398 -0.6193633684
H -4.0238298518 2.2636442835 -0.5934483827
H -0.5853458530 1.8869182909 2.1760911955
C -3.2684535008 0.6223426563 -2.6769201738
H -4.0693411282 1.3093590782 -2.9697279659
H -3.3625037444 -0.2927465428 -3.2676923309
H -2.3347356000 0.2645209056 2.8281191343
H -2.2105528766 -0.5536261011 1.2784099657
S 0.8652307804 -1.1434501730 0.9236667716
O -0.1332488148 -1.3589565370 -0.1358579031
O 1.3321317603 -2.2540460518 1.7602278049
C 2.2745832731 -0.3495276383 0.1507222758
C 2.2842267793 -0.1662266570 -1.2297964716
C 3.3713212125 0.0257784924 0.9331218410
C 3.4042321458 0.4118478068 -1.8310387381
H 1.4275024833 -0.4760769803 -1.8180407406
C 4.4771969544 0.5998931790 0.3177768823
H 3.3528833524 -0.1280773009 2.0070491770
C 4.5131112952 0.8021850355 -1.0728231748
H 3.4140961378 0.5576027948 -2.9082513526
H 5.3305443869 0.8957882117 0.9234151167
C 5.7284300428 1.4178549698 -1.7239921032
H 5.5671810189 1.5906804904 -2.7921918845
H 5.9850034757 2.3782337019 -1.2611489939
H 6.6052149956 0.7665457122 -1.6195097108
H -2.3054522510 1.0898067936 -2.9187559446
H 0.1428992626 -0.4466299578 2.9025392006
H -2.5479862032 -0.4034039311 -0.9219828447
N 0.3360669701 0.0281030186 2.0220080104
Cl -4.9038798217 -0.5569762097 -0.8185494131
H -0.5088967324 1.2238235275 0.5520186568
38

C -1.9277082182 1.6257639518 1.2871245460
C -2.5014672147 0.3493953888 0.6583445462
C -3.9964148007 0.4860846064 0.3268481598
C -4.5989187604 -0.8001030136 -0.2430178647
C -0.4507834399 1.5362910412 1.6990150093
H -2.3479126347 -0.4934502258 1.3461146152
H -1.9388578479 0.1101863750 -0.2500470133
H -4.1507966421 1.2998961854 -0.3943704583
H -4.5495523190 0.7598943138 1.2334023636
H -0.2928995508 0.7077269748 2.3939342099
C -4.0472485806 -1.2217761353 -1.5985653089
H -4.1832404102 -0.4255954967 -2.3383029799
H -4.5513260724 -2.1211188740 -1.9627627210
H -2.0579244790 2.4705035531 0.5933686328
H -2.5017535177 1.8864608758 2.1879065981
S 0.9406739506 -0.1955324911 0.0751127008
O 0.3942496799 -0.4252555022 -1.2714500181
O 0.6415940336 -1.1076687842 1.1850440389
C 2.7206084897 -0.0771858706 -0.0974624377
C 3.5249055126 -0.1175413992 1.0448468945
C 3.2833621492 0.0378485878 -1.3669354745
C 4.9054829400 -0.0289917937 0.9039317437
H 3.0727940812 -0.2266644425 2.0251754500
C 4.6708770479 0.1266241891 -1.4884806805
H 2.6427157376 0.0457099099 -2.2420908072
C 5.5012750676 0.0950609809 -0.3621265007
H 5.5346899632 -0.0607597904 1.7902672008
H 5.1133643174 0.2164359536 -2.4774088546
C 7.0034587287 0.1665391680 -0.4984010426
H 7.4393328494 0.8531242977 0.2360816866
H 7.4612655934 -0.8171250165 -0.3315559854
H 7.3003680703 0.5035396613 -1.4961896802
H -2.9761556471 -1.4393222304 -1.5174289957
H 0.3347340895 1.9462888570 -0.2142839933
H -4.4932386379 -1.6141393247 0.4795353104
N 0.5096241670 1.3540601564 0.5948647657
Cl -6.4256034480 -0.5764934099 -0.3901334691
H -0.1589379453 2.4576131565 2.2178148475
38

C -1.9464624072 1.8756371013 0.7079016290
C -2.5206966259 0.5850923951 0.1076635762
C -4.0015396652 0.3915307457 0.4746611161
C -4.5596498455 -0.9594215998 0.0222951231
C -0.4991932329 2.1935934795 0.3049411021
H -1.9407644227 -0.2765276262 0.4626743369
H -2.3951825791 0.6104966034 -0.9812371881
H -4.6088211253 1.1937546909 0.0347780566
H -4.1200176785 0.4686153412 1.5624297787
H -0.2144504594 3.1736095228 0.7072122356
C -4.5974631771 -1.1661933959 -1.4858332720
H -5.1718823203 -0.3718460633 -1.9744471983
H -5.0530773159 -2.1276311293 -1.7377146232
H -2.5643281931 2.7317208998 0.4004018765
H -2.0152801213 1.8356560039 1.8057405977
S 0.9509878807 -0.0966523586 -0.1739940408
O 0.4933391991 0.1856347925 -1.5397158868
O 0.5562699640 -1.3242223297 0.5322881794
C 2.7426096977 -0.0399531159 -0.1652506611
C 3.4058728921 0.8394818781 -1.0248804952
C 3.4540829228 -0.8786120725 0.6908682207
C 4.7962421391 0.8806441806 -1.0106120649
H 2.8383255514 1.4708137912 -1.7005593660
C 4.8482043987 -0.8226745360 0.6917007982
H 2.9215647022 -1.5706897781 1.3342724038
C 5.5398776454 0.0530096176 -0.1539405170
H 5.3158807693 1.5631290806 -1.6789468093
H 5.4062559683 -1.4758426466 1.3579368334
C 7.0494034595 0.0908193007 -0.1697004593
H 7.4718450259 -0.3720920900 0.7273928707
H 7.4476197177 -0.4495943290 -1.0384120804
H 7.4233749083 1.1188555545 -0.2305625681
H -3.5779083486 -1.1568700887 -1.8880676103
H 0.3996206498 0.9347775130 1.7391784931
H -4.0061595220 -1.7714356355 0.5017393182
N 0.5153546094 1.2327860235 0.7731621692
Cl -6.2720442934 -1.1378256885 0.6879506934
H -0.4052132433 2.2482032390 -0.7824538498

10-Cl

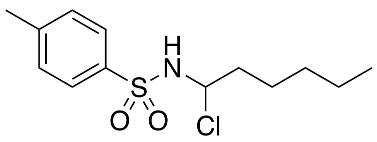

Name	E(B3LYP)	H(B3LYP)	E(RO-B2PLYP-D3)	H(RO-B2PLYP-D3)
Tosyl_NH_C2_Cl_hexane_0000	-1570.969918	-1570.631246	-1570.456882	-1570.118210
Tosyl_NH_C2_Cl_hexane_0014	-1570.970284	-1570.631769	-1570.456124	-1570.117609
Tosyl_NH_C2_Cl_hexane_0002	-1570.968564	-1570.629824	-1570.456105	-1570.117365
Tosyl_NH_C2_Cl_hexane_0053	-1570.968844	-1570.630287	-1570.455780	-1570.117223
Tosyl_NH_C2_Cl_hexane_0038	-1570.969623	-1570.631161	-1570.455600	-1570.117138
Tosyl_NH_C2_Cl_hexane_0066	-1570.969282	-1570.630653	-1570.455519	-1570.116890
Tosyl_NH_C2_Cl_hexane_0025	-1570.969005	-1570.630402	-1570.455321	-1570.116718
Tosyl_NH_C2_Cl_hexane_0027	-1570.968936	-1570.630338	-1570.455309	-1570.116711
Tosyl_NH_C2_Cl_hexane_0029	-1570.968989	-1570.630386	-1570.455297	-1570.116694

38
-1570.118210
C 2.5335823973 -1.1911137092 -0.2109849540
C 3.5771900226 -0.1116452698 0.1145637470
C 4.9900906856 -0.5112952552 -0.3314716174
C 6.0480712549 0.5531979553 -0.0130762110
C 1.1312918467 -0.8226229046 0.2713090336
N 0.6199650144 0.3211061737 -0.3885694770
H 3.5759741598 0.0797779572 1.1969960531
H 3.2908044165 0.8335735858 -0.3623970666
H 4.9890893050 -0.7089150103 -1.4138845400
H 5.2726450269 -1.4592917365 0.1498428896
H 1.0997388473 -0.6419323311 1.3445824442
C 7.4574867154 0.1538741589 -0.4614933359
H 7.7828029187 -0.7718239755 0.0290738066
H 8.1888549330 0.9332897214 -0.2197891238
H 2.5005320423 -1.3805226807 -1.2924849030
H 2.8106604409 -2.1394717413 0.2613788677
S -0.5103701822 1.3842093109 0.3026426265
O -0.4257267207 2.5807089742 -0.5337455657
O -0.2060993308 1.3851846155 1.7338503856
C -2.1456330069 0.6894912338 0.0798411584
C -2.6902109313 -0.1306430056 1.0696013825
C -2.8624544917 0.9916986937 -1.0798992400
C -3.9641404046 -0.6602435624 0.8824101279
H -2.1298629920 -0.3352076446 1.9748645882
C -4.1342821158 0.4498469382 -1.2495558262
H -2.4392475665 1.6613972823 -1.8211184722
C -4.7019906832 -0.3873757598 -0.2785252141
H -4.3946020661 -1.2938125383 1.6537488018
H -4.6994119623 0.6891679365 -2.1468905081
C -6.0686833256 -0.9947344445 -0.4845570301
H -6.5938715606 -1.1322486008 0.4662140048
H -6.6922533436 -0.3703005598 -1.1322978078
H -5.9902086257 -1.9820194110 -0.9587806706
H 7.4965387541 -0.0149795691 -1.5447396106
Cl 0.0302093057 -2.3447288010 0.0434740617
H 6.0489291622 0.7496566756 1.0682824181
H 0.5914992852 0.3077177418 -1.4042283416
H 5.7641958810 1.5000349064 -0.4934006172
38
-1570.117609
C 2.4877187762 -1.5968299330 -1.0486641521
C 3.6400553790 -0.5764226694 -1.0282906602
C 4.1292627602 -0.1860206712 0.3736672552
C 5.3730719303 0.7122808774 0.3414602473
C 1.2224347667 -1.1075613339 -0.3424248917
N 0.6969826666 0.0680445680 -0.9328996124
H 3.3387719070 0.3268850755 -1.5731943446
H 4.4740896208 -1.0161006359 -1.5925748914
H 4.3547192054 -1.0964418159 0.9500531806
H 3.3300575641 0.3367625332 0.9163117973
H 1.3864043395 -0.9140079040 0.7151014008
C 5.8591427067 1.1158622226 1.7370224575
H 6.7467696658 1.7561863939 1.6807635736
H 6.1213422918 0.2349114610 2.3360692267
H 2.2325197464 -1.8557631203 -2.0843629102
H 2.7972980640 -2.5281936433 -0.5621670413
S -0.1697265355 1.2460270790 -0.0728854550
O -0.1617800084 2.4086966972 -0.9597307132
O 0.4286834301 1.2554391034 1.2634856069
C -1.8670978181 0.6977160766 0.0823007914
C -2.2557762673 -0.0450699282 1.1985803660
C -2.7853174064 1.0336351128 -0.9147876009
C -3.5797226448 -0.4628301319 1.3040728425
H -1.5358599590 -0.2754884880 1.9757863946
C -4.1045090940 0.6042322570 -0.7917939652
H -2.4744180477 1.6431626295 -1.7566973737
C -4.5212350468 -0.1539152552 0.3117288994
H -3.8881962908 -1.0354944938 2.1750241283
H -4.8248117236 0.8705917926 -1.5612436275
C -5.9460595070 -0.6406855389 0.4218310270
H -6.2681892577 -0.7051421154 1.4662323183
H -6.6384650333 0.0202365522 -0.1092591643
H -6.0513582024 -1.6431469152 -0.0137553237
H 5.0833755795 1.6675726340 2.2814167917
Cl -0.0152240440 -2.5350365627 -0.3417184433
H 5.1493817354 1.6154670945 -0.2433405837
H 0.4779026613 0.0467742380 -1.9247823621
H 6.1813786712 0.1937901004 -0.1940660832
38
-1570.117365
C 2.6642267107 0.2167394245 -0.4906626221
C 3.6877967685 -0.7063271357 0.1798654592
C 5.1042868263 -0.5095471270 -0.3768077154
C 6.1454592381 -1.4227214438 0.2832019279
C 1.2494130758 0.1322306735 0.0823489826
N 0.4041544326 1.1233447451 -0.4764339067
H 3.3826269950 -1.7508584895 0.0494261345
H 3.6916418977 -0.5149629159 1.2627242504
H 5.4084402010 0.5397404659 -0.2474605937
H 5.0977668406 -0.6931774756 -1.4615876558
H 1.2379288401 0.2427228165 1.1657542245
C 7.5583317772 -1.2272097418 -0.2758246337
H 8.2776207593 -1.8921602210 0.2154210558
H 7.9039273337 -0.1964284100 -0.1294720242
H 2.9738599739 1.2630836741 -0.3679967755
H 2.6252527822 0.0185793689 -1.5703018950
S -0.9331303093 1.7868047433 0.3324249469
O -1.2289356825 3.0066461791 -0.4177619903

O -0.5589923246 1.7880338606 1.7471616130
C -2.3273592402 0.6817628393 0.1262071923
C -2.5733802859 -0.3080908761 1.0793841466
C -3.1622869607 0.8372225610 -0.9822453314
C -3.6638568885 -1.1561841252 0.9065435038
H -1.9293743617 -0.3988133816 1.9467551895
C -4.2464899255 -0.0231394842 -1.1387019116
H -2.9788141951 1.6362496775 -1.6928167027
C -4.5110380326 -1.0346435898 -0.2044864296
H -3.8629952709 -1.9237096415 1.6501765658
H -4.9039821895 0.0995811814 -1.9957335916
C -5.6708263819 -1.9817953350 -0.3969831374
H -5.3495847154 -2.8921773109 -0.9202166038
H -6.0979671360 -2.2926545493 0.5620693945
H -6.4670867497 -1.5258363381 -0.9938790517
H 7.5917943353 -1.4372671119 -1.3520012417
Cl 0.5827649389 -1.6151208729 -0.1936405120
H 5.8409202180 -2.4707345682 0.1534033944
H 0.3242489341 1.1628120579 -1.4884210822
H 6.1515060218 -1.2397665578 1.3670296561
38
-1570.117223
C 2.7005227899 -0.3962273481 1.0411185073
C 3.3218718768 -0.3973995196 -0.3609245946
C 4.8492342133 -0.5452173282 -0.3208296627
C 5.4968323681 -0.5329783788 -1.7113436156
C 1.1737106310 -0.3180204121 1.1024185060
N 0.6554662038 0.8150685096 0.4312728008
H 3.0748045401 0.5392436618 -0.8820405197
H 2.8899648942 -1.2153232654 -0.9510605746
H 5.1076707870 -1.4833252557 0.1917801693
H 5.2798211458 0.2638391652 0.2874219935
H 0.8208824838 -0.2941476855 2.1315787832
C 7.0202107708 -0.6887912867 -1.6665428084
H 7.4853581740 0.1227900603 -1.0936342203
H 7.4525091093 -0.6756211972 -2.6733723546
H 3.0061649082 -1.2977193730 1.5833497540
H 3.0711670871 0.4670990103 1.6103759097
S -0.7947467739 1.5830832746 0.8694636222
O -0.7146098564 2.8869207545 0.2124155143
O -0.8874920122 1.4227853759 2.3209687615
C -2.1542138203 0.6894273754 0.1214118777
C -2.5781786285 1.0427154875 -1.1611517196
C -2.7835164044 -0.3352371606 0.8309824014
C -3.6370843420 0.3464356597 -1.7395340738
H -2.1028049897 1.8666499497 -1.6828465965
C -3.8416605715 -1.0170648496 0.2366970655
H -2.4574152452 -0.5801552274 1.8354513274
C -4.2800511780 -0.6952003574 -1.0562100705
H -3.9759902741 0.6235634597 -2.7345841571
H -4.3387852850 -1.8109128656 0.7884602238
C -5.4081105925 -1.4643366033 -1.7005331189
H -5.9000411562 -0.8768343076 -2.4820724401
H -5.0364375487 -2.3860443387 -2.1674167218
H -6.1651410776 -1.7565009420 -0.9651877799
H 7.3082155486 -1.6352307847 -1.1927191663
Cl 0.4638612046 -1.9500773920 0.4463712503
H 5.2396134409 0.4057526584 -2.2223389693
H 0.8987766819 0.9458149756 -0.5454704238
H 5.0631469351 -1.3395374193 -2.3191708701
38
-1570.117138
C 2.5478766743 -1.4036482297 -0.1397199101
C 3.6389259461 -0.3663201325 0.1674943006
C 5.0321237291 -0.8504188353 -0.2605468516
C 6.1727697745 0.1232796100 0.0798332600
C 1.1606877764 -0.9566821739 0.3193398111
N 0.7089241961 0.1913388609 -0.3756973702
H 3.6437080006 -0.1531530571 1.2461819332
H 3.3922378427 0.5777687565 -0.3302605263
H 5.0329464918 -1.0433928584 -1.3439363448
H 5.2364020814 -1.8173287598 0.2208282875
H 1.1288041611 -0.7455064451 1.3870083712
C 6.1161556969 1.4576291449 -0.6735045463
H 5.2185899475 2.0335102575 -0.4224527199
H 6.1161081775 1.2978226528 -1.7592411750
H 2.5148193590 -1.6193969905 -1.2163245612
H 2.7755742874 -2.3515473541 0.3592480616
S -0.3747668738 1.3251370499 0.2766853974
O -0.2241420870 2.4946510766 -0.5881122115
O -0.0841750195 1.3477816958 1.7105719685
C -2.0395120352 0.7045946685 0.0535936110
C -2.6320573469 -0.0646853797 1.0567593818
C -2.7302479126 1.0131947200 -1.1200255726
C -3.9281672067 -0.5366962724 0.8689803656
H -2.0904461133 -0.2743505447 1.9722029940
C -4.0253035502 0.5291359994 -1.2899936860
H -2.2684030071 1.6438328450 -1.8723370213
C -4.6415310260 -0.2562700711 -0.3055975159
H -4.3957206495 -1.1302998343 1.6504862923
H -4.5699740291 0.7737899613 -2.1984446982
C -6.0343088686 -0.8017672744 -0.5100527051
H -5.9992399077 -1.8182314828 -0.9239166653
H -6.5864238631 -0.8552033719 0.4340309467
H -6.6091991919 -0.1844541497 -1.2075609956
H 6.9835510650 2.0816740220 -0.4304424113
Cl -0.0088674147 -2.4314856211 0.1223183817
H 7.1280980316 -0.3709768966 -0.1417353688
H 0.6881945189 0.1519587950 -1.3908702446
H 6.1732721562 0.3133236180 1.1625738669
38
-1570.116890
C 2.5466163699 -1.2882680277 -0.4122409680
C 3.6264043362 -0.2239484338 -0.1628371068
C 5.0043070630 -0.6707952394 -0.6731801125
C 6.1116691414 0.3842908188 -0.5136400972
C 1.1789289929 -0.8833063532 0.1354787227
N 0.6574086733 0.2610102900 -0.5151183486
H 3.6741759117 -0.0078811377 0.9124318043
H 3.3381145404 0.7150502889 -0.6516014093
H 4.9189934736 -0.9352900061 -1.7369540726
H 5.3049398130 -1.5927877881 -0.1535142159
H 1.2062176817 -0.6863758115 1.2059933589
C 6.4656975700 0.7197862107 0.9400497490
H 6.7663802370 -0.1804930731 1.4908629551
H 5.6220084986 1.1703477205 1.4744854599
H 2.4533187518 -1.4974160155 -1.4865811334
H 2.8301551741 -2.2330195672 0.0636484149
S -0.4132574166 1.3581643805 0.2163048831
O -0.3450323128 2.5413507241 -0.6402803777
O -0.0372516342 1.3714154057 1.6303286558
C -2.0722214305 0.6971204619 0.0855392658
C -2.5831371759 -0.0999066346 1.1116236611
C -2.8405025633 1.0021587176 -1.0398772814
C -3.8758021913 -0.6035400036 0.9955773330
H -1.9819974518 -0.3067118953 1.9898034096
C -4.1306925207 0.4864514866 -1.1383055518
H -2.4413040043 1.6540912953 -1.8096895523
C -4.6658930721 -0.3273180226 -0.1297854492
H -4.2798447169 -1.2190230334 1.7953485687
H -4.7354967257 0.7281650439 -2.0087117041
C -6.0539885942 -0.9068817217 -0.2581573001
H -6.0187532732 -1.9117341487 -0.6994813659
H -6.5420630860 -0.9983503570 0.7177060939
H -6.6891656781 -0.2896502857 -0.9013871428
H 7.2985301478 1.4305098189 0.9862427391
Cl 0.0371107752 -2.3854908195 -0.0115240966
H 5.8131713949 1.3024212873 -1.0392231469
H 0.5781134575 0.2346601541 -1.5277978563
H 7.0129668419 0.0208378006 -1.0251153582
38
-1570.116718
C 2.6599606168 -0.9219021424 0.1398048364
C 3.5983608449 0.2034626003 0.6066447566
C 5.0871604715 -0.0985470140 0.3665193252
C 5.5005211234 -0.1793374454 -1.1101168419
C 1.2016224070 -0.6570798010 0.5109082258
N 0.6818648448 0.4882455476 -0.1391674916
H 3.4388629943 0.3732341759 1.6800959637
H 3.3256000624 1.1412910043 0.1073195742

H 5.3567054179 -1.0380223273 0.8717111846
H 5.6796873839 0.6877865960 0.8544234582
H 1.0655299500 -0.5334895204 1.5841087417
C 7.0067218517 -0.3931604337 -1.2938244473
H 7.2768946762 -0.4458153458 -2.3545278509
H 7.3351168651 -1.3263358925 -0.8198160145
H 2.7258731875 -1.0681018045 -0.9454031848
H 2.9538776856 -1.8714753541 0.6002748407
S -0.5677105025 1.4489337790 0.4940481789
O -0.4800450812 2.6911436728 -0.2724488504
O -0.3943742324 1.3905152558 1.9457611879
C -2.1313375067 0.6773002629 0.0867022310
C -2.7130103118 -0.2231313712 0.9810825124
C -2.7572501490 1.0006269560 -1.1189551542
C -3.9306880031 -0.8116097427 0.6507804464
H -2.2254689634 -0.4441804938 1.9238252480
C -3.9738273267 0.3990044228 -1.4322620589
H -2.3096990571 1.7311312430 -1.7845814809
C -4.5757443617 -0.5185739072 -0.5595759178
H -4.3903753830 -1.5080535346 1.3474541124
H -4.4688560970 0.6540188971 -2.3659326266
C -5.8796625880 -1.1886220201 -0.9201830166
H -6.4687725883 -0.5761013318 -1.6100767093
H -5.7002894290 -2.1544090589 -1.4109220392
H -6.4888269154 -1.3846122549 -0.0317132633
H 7.5806818962 0.4258670579 -0.8433592116
Cl 0.2184450166 -2.2258649482 0.1161991967
H 5.1969862778 0.7466708684 -1.6195188752
H 0.7466723520 0.5277178066 -1.1524471752
H 4.9594709452 -0.9947324232 -1.6088414522
38
-1570.116711
C 2.6584261888 -0.6928674520 -0.3060607215
C 3.5834400460 0.4832052948 0.0481808054
C 5.0176129576 0.3193583042 -0.4829586036
C 5.8179581341 -0.8293228729 0.1475625599
C 1.2327852941 -0.4914019299 0.2065257259
N 0.5896027415 0.6096009046 -0.4092989778
H 3.6111410649 0.6059090971 1.1403449711
H 3.1540296271 1.4088082623 -0.3518084703
H 5.5570722954 1.2605443717 -0.3079838514
H 4.9891418976 0.1865837207 -1.5749801271
H 1.2003563943 -0.3477234415 1.2853812606
C 7.2641966116 -0.8905336596 -0.3552589688
H 7.8148066143 -1.7156850832 0.1103014023
H 7.8016054445 0.0387556817 -0.1297723888
H 2.6283259739 -0.8409238895 -1.3944563215
H 3.0333493751 -1.6257244899 0.1244615247
S -0.6355076729 1.5235697122 0.3318953621
O -0.6938106822 2.7474581492 -0.4662156805
O -0.3082185754 1.5130779107 1.7579832630
C -2.1906720750 0.6643075666 0.1095673418
C -2.9550494179 0.9220529246 -1.0303347294
C -2.6279041247 -0.2382723187 1.0806692516
C -4.1644485084 0.2521827579 -1.1995283983
H -2.6183113548 1.6548917969 -1.7559710504
C -3.8409722902 -0.8956106176 0.8943964200
H -2.0338250468 -0.4080076615 1.9715046414
C -4.6231561355 -0.6692782047 -0.2473978499
H -4.7668843099 0.4558885933 -2.0812439322
H -4.1883837750 -1.5940310338 1.6514913227
C -5.9205880414 -1.4132115451 -0.4533531065
H -5.7445176814 -2.3753826603 -0.9523397480
H -6.4154203980 -1.6276751487 0.4994830448
H -6.6153923061 -0.8435671813 -1.0786745307
H 7.3002155744 -1.0374015495 -1.4418078061
Cl 0.2971732753 -2.1156937768 -0.0546789396
H 5.3262212993 -1.7895358009 -0.0578620242
H 0.5441804247 0.6232338131 -1.4243413237
H 5.8146477166 -0.7118592644 1.2407806426
38
-1570.116694
C 2.6292904254 0.1901397051 -1.4826197022
C 3.7742096338 -0.7530589688 -1.0865563564
C 4.3362332869 -0.5225196880 0.3229697518
C 5.5605772334 -1.3962098391 0.6274092378
C 1.3770649324 0.1319335514 -0.6054295705
N 0.4231486191 1.0959768307 -1.0144993355
H 4.5796473787 -0.6117905145 -1.8201934855
H 3.4435523309 -1.7933160520 -1.1873771664
H 3.5613707131 -0.7282405725 1.0749239567
H 4.6075497694 0.5371783980 0.4426033618
H 1.5956478850 0.2945387751 0.4478428530
C 6.1201332191 -1.1793505618 2.0368506277
H 6.9932416596 -1.8152976677 2.2218211586
H 5.3695378656 -1.4132668358 2.8017105626
H 2.9740842742 1.2317696753 -1.4351118701
H 2.3351144634 -0.0024404939 -2.5227140045
S -0.7182329984 1.7962733336 0.0290990601
O -1.1729981015 2.9844557928 -0.6918296079
O -0.0556795503 1.8557367421 1.3325436354
C -2.1170775448 0.6863088348 0.1650483892
C -2.1527769672 -0.2608386827 1.1899619628
C -3.1654578362 0.7957426124 -0.7508693406
C -3.2497425519 -1.1129484105 1.2844061846
H -1.3417781952 -0.3155433195 1.9073050330
C -4.2526990493 -0.0679932861 -0.6412997514
H -3.1393162641 1.5629645506 -1.5174931269
C -4.3103480116 -1.0375034988 0.3699465626
H -3.2847241009 -1.8471082251 2.0853591636
H -5.0749503844 0.0191499757 -1.3471033239
C -5.4785983894 -1.9891632802 0.4638250109
H -5.2680090056 -2.9214355202 -0.0768130301
H -5.6937342320 -2.2585113462 1.5030652257
H -6.3849923465 -1.5567408878 0.0282966273
H 6.4292370426 -0.1373726719 2.1852497463
Cl 0.6843791487 -1.6276468083 -0.6467976805
H 6.3448977604 -1.1900593514 -0.1148250628
H 0.1333630440 1.0945712575 -1.9881873499
H 5.2898168151 -2.4535909694 0.4986672031

8pyrrolidine

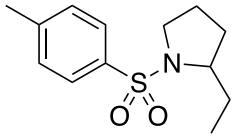

Name	E(B3LYP)	H(B3LYP)	E(RO-B2PLYP-D3)	H(RO-B2PLYP-D3)
Tosyl_2_ethyl_pyrrolidine_0035	-1110.159549	-1109.835395	-1109.743836	-1109.419682
Tosyl_2_ethyl_pyrrolidine_0041	-1110.158436	-1109.834118	-1109.743432	-1109.419114
Tosyl_2_ethyl_pyrrolidine_0099	-1110.158392	-1109.8343	-1109.743055	-1109.418963
Tosyl_2_ethyl_pyrrolidine_0025	-1110.157388	-1109.833185	-1109.742485	-1109.418282
Tosyl_2_ethyl_pyrrolidine_0067	-1110.158323	-1109.834324	-1109.740392	-1109.416393
Tosyl_2_ethyl_pyrrolidine_0032	-1110.158393	-1109.834389	-1109.740212	-1109.416208
Tosyl_2_ethyl_pyrrolidine_0071	-1110.157212	-1109.833077	-1109.740145	-1109.41601
Tosyl_2_ethyl_pyrrolidine_0023	-1110.157082	-1109.832986	-1109.739689	-1109.415593
Tosyl_2_ethyl_pyrrolidine_0043	-1110.157042	-1109.832995	-1109.739574	-1109.415527

36

C 2.3822434009 0.1569136653 2.4143873411
C 2.1656617946 1.4062391288 1.5449346204
C 1.9716691090 0.8509402934 0.1142831415
C 3.2489749378 0.8355568026 -0.7406010453
C 1.4040997842 -0.8521328414 1.8081831185
N 1.4850112459 -0.5402179944 0.3661698203
H 2.9963248548 2.1157823676 1.6012744182
H 1.2594279578 1.9312595634 1.8697014646
C 3.6719940318 2.2287340458 -1.2185472869
H 3.8908256042 2.9067829395 -0.3846363793
H 2.8854627367 2.6931703538 -1.8263681597
H 3.4096513616 -0.2106296534 2.3138929044
H 2.1888606334 0.3332395457 3.4767821473
S 0.3809890804 -1.2465036471 -0.6705784614
O 0.2553419222 -2.6413259040 -0.2399865393
O 0.7890061220 -0.8804832877 -2.0301889115
C -1.2054727575 -0.4540937935 -0.3681880051
C -2.0556257612 -0.9599999604 0.6192166223
C -1.5663943131 0.6789855796 -1.1027539128
C -3.2633438297 -0.3139308631 0.8773634231
H -1.7823247020 -1.8631970910 1.1543256050
C -2.7778896070 1.3117825407 -0.8311288898
H -0.9160495099 1.0371872619 -1.8936149537
C -3.6435534377 0.8296020912 0.1609603130
H -3.9265127251 -0.7105845426 1.6424873226
H -3.0606561916 2.1901709444 -1.4065037048
C -4.9703497648 1.5016845909 0.4231309583
H -4.9242007651 2.5757149041 0.2152246435
H -5.7575340270 1.0800152229 -0.2158596772
H -5.2893512827 1.3686423025 1.4620350118
H 4.5739702939 2.1687165383 -1.8370345661
H 3.0633850873 0.1897118072 -1.6039405512
H 1.2021523195 1.4267148657 -0.4160173649
H 4.0584232886 0.3679458755 -0.1635339795
H 1.6755875834 -1.8952196079 1.9813394257
H 0.3889557040 -0.6862611453 2.2016551754
36

C 2.7197861619 2.2295303028 0.1623535267
C 3.4608495540 1.0565981286 0.8157396361
C 2.6231358553 -0.1865711058 0.4445240075
C 2.6424928405 -1.3080991537 1.4872013103
C 1.2529905232 1.8514130135 0.3851240319
N 1.2323164457 0.3679935975 0.2693280251
H 3.4751397050 1.1816017109 1.9068514538
H 4.4975698097 0.9604522026 0.4801035831
C 4.0342469998 -1.9201417341 1.6834906642
H 4.7565958851 -1.1849662174 2.0575810460
H 4.4250922954 -2.3234464720 0.7412484634
H 2.9786670410 3.2013780866 0.5949493246
H 2.9284115590 2.2644495517 -0.9132218668
S 0.3557415915 -0.2540402442 -1.0372967129
O 0.7256922521 -1.6717500291 -1.1316687199
O 0.4664867211 0.6045364036 -2.2291759029
C -1.3314399656 -0.1123487679 -0.4429868790
C -1.7018846587 -0.7256102940 0.7575841336
C -2.2717900068 0.5558958504 -1.2241478072
C -3.0261852308 -0.6539854971 1.1752344839
H -0.9580664635 -1.2398374831 1.3571316673
C -3.5971309460 0.6169219220 -0.7899672803
H -1.9620254407 1.0215523213 -2.1534572242
C -3.9954581114 0.0149674821 0.4088815585
H -3.3163805724 -1.1247904825 2.1116470472
H -4.3319422603 1.1415693015 -1.3955382037
C -5.4332475020 0.0653879795 0.8679538527
H -5.5064750562 0.3538426325 1.9228765440
H -6.0173963239 0.7797820203 0.2800194899
H -5.9132486317 -0.9167422800 0.7688141927
H 3.9983382312 -2.7436392667 2.4049380923
H 1.9425422488 -2.0853458516 1.1635701619
H 2.9770341178 -0.5911595364 -0.5114815228
H 2.2659163711 -0.9079728641 2.4387686488
H 0.9173263054 2.1286151670 1.3921192285
H 0.5763701454 2.3082023865 -0.3399754682
36

C 2.4499040517 2.3522373979 -0.5568008624
C 3.4814098781 1.2344142122 -0.3376187233
C 2.6701107274 0.0629520042 0.2642890975
C 2.7339675482 -0.0366806242 1.7952768977
C 1.2173800048 1.5777688600 -1.0250621299
N 1.2623246220 0.3642067111 -0.1774756266
H 4.3069087374 1.5382151900 0.3129137188
H 3.9123127769 0.9349921150 -1.2999379222
C 4.0860043848 -0.5384501383 2.3139838998
H 4.0780466057 -0.6170241067 3.4066172209
H 4.9101732950 0.1309391597 2.0395877396
H 2.2346395261 2.8720766199 0.3838463286
H 2.7721316103 3.0984112885 -1.2894744674
S 0.3238494193 -0.9419918487 -0.6880016793
O 0.7044404289 -2.0738091643 0.1660495539
O 0.3567961415 -1.0841451977 -2.1526953391
C -1.3296034678 -0.4017768148 -0.2465044758
C -2.2639450832 -0.1611242267 -1.2513907756
C -1.6750250682 -0.2687821451 1.1016831563
C -3.5575927354 0.2275460636 -0.8983423103
H -1.9762269613 -0.2823431206 -2.2901363108
C -2.9677438218 0.1197101454 1.4351488711

H -0.9390334611 -0.4678370799 1.8737119750
C -3.9298203331 0.3729310666 0.4427954627
H -4.2885178961 0.4176307426 -1.6803778181
H -3.2393949253 0.2264035615 2.4827879754
C -5.3351906296 0.7733180068 0.8238455322
H -5.8856656620 -0.0761860077 1.2483017149
H -5.3338601063 1.5659355917 1.5808933223
H -5.8988713441 1.1328267824 -0.0423640318
H 4.3164576100 -1.5324107139 1.9118246318
H 1.9459597241 -0.7259227174 2.1160679954
H 3.0008047436 -0.8917133120 -0.1582263433
H 2.4974252534 0.9445734709 2.2298019739
H 0.2768305373 2.1141628044 -0.8632815554
H 1.2943953358 1.3218824493 -2.0903423843
36

C 1.4812546595 0.4483616734 2.4527541412
C 2.3754586269 1.2812136959 1.5250253268
C 2.0279640105 0.7939239614 0.0996906401
C 3.1976628421 0.8577302870 -0.8899218222
C 1.4832044238 -0.9248883552 1.7739736446
N 1.5605822671 -0.6094196840 0.3258681518
H 3.4324644357 1.0714394630 1.7348067685
H 2.2248476029 2.3590804639 1.6342127619
C 3.6732990370 2.2900777868 -1.1586423676
H 4.4816077869 2.2966213770 -1.8976900636
H 4.0519519266 2.7765169668 -0.2517483149
H 1.8477307454 0.4096503803 3.4835279551
H 0.4627809758 0.8548240143 2.4694100176
S 0.4142748718 -1.2726764835 -0.7011552753
O 0.2697038743 -2.6747275184 -0.3021187460
O 0.8004948783 -0.8815395574 -2.0599413259
C -1.1568824340 -0.4629010411 -0.3577707162
C -1.5008780360 0.7037268810 -1.0465821034
C -2.0148143262 -0.9927827296 0.6103718200
C -2.6992926931 1.3482109374 -0.7443712800
H -0.8488631712 1.0797524134 -1.8276190996
C -3.2087891965 -0.3351518217 0.8995167339
H -1.7602509011 -1.9239800661 1.1052634499
C -3.5697296392 0.8440195891 0.2318600117
H -2.9675887815 2.2535313781 -1.2837678654
H -3.8776726431 -0.7511096430 1.6493073908
C -4.8826464431 1.5292707132 0.5276323799
H -5.6875179071 1.1243200548 -0.1000202294
H -5.1828970365 1.3880452380 1.5711413474
H -4.8271898231 2.6049132271 0.3311826518
H 2.8602839723 2.9116243967 -1.5550690110
H 2.8838073705 0.3862953183 -1.8253275665
H 1.1945493615 1.3965300505 -0.2913112039
H 4.0217278860 0.2521551805 -0.4895985289
H 2.3653226097 -1.5120765729 2.0573235027
H 0.6034135257 -1.5307893168 1.9993690242
36

C 2.4092971873 0.3396678272 2.3165590263
C 2.1269551887 1.6130931751 1.5010935007
C 1.8267603835 1.1216141408 0.0612521534
C 2.9728819733 1.3148458151 -0.9478337218
C 1.4573676995 -0.6850210739 1.6968071594
N 1.4893824017 -0.3221338597 0.2640382258
H 2.9573828983 2.3254089099 1.5216828104
H 1.2462753946 2.1242705415 1.9069420513
C 4.3004514288 0.6470683061 -0.5790196921
H 4.7246050829 1.0496224712 0.3490721578
H 5.0398906694 0.8088461828 -1.3710304448
H 3.4458568016 0.0144321547 2.1827310450
H 2.2302141827 0.4700945624 3.3882321040
S 0.4021458729 -1.0794815565 -0.7604794256
O 0.3943655091 -2.4913517242 -0.3690992960
O 0.7342593048 -0.6492179838 -2.1216679484
C -1.2263508252 -0.4147952914 -0.3834605833
C -1.6933013171 0.7085567175 -1.0717164934
C -2.0050097095 -1.0114295185 0.6123213428
C -2.9395447312 1.2402311996 -0.7454109430
H -1.0967484293 1.1371459884 -1.8699256868
C -3.2487730425 -0.4660036627 0.9252343017
H -1.6486170794 -1.9064323000 1.1110541219
C -3.7353485625 0.6657929797 0.2558206029
H -3.3047185171 2.1110328406 -1.2847383851
H -3.8563395086 -0.9333304813 1.6966008811
C -5.0997354103 1.2277607783 0.5774020786
H -5.8750348603 0.7541397729 -0.0393228993
H -5.3680311587 1.0555333858 1.6248863730
H -5.1454556631 2.3048242892 0.3860576144
H 4.1728688060 -0.4332813162 -0.4566631524
H 3.1200623219 2.3982606164 -1.0655843069
H 0.9492936681 1.6547446126 -0.3277955601
H 2.6315663983 0.9347319980 -1.9155117259
H 1.7741457774 -1.7215666125 1.8253636218
H 0.4460272606 -0.5741645829 2.1191313366
36

C 1.4817366956 0.5292301831 2.4442336211
C 2.2705112903 1.4937190586 1.5491173796
C 1.8911926472 1.0879786907 0.1076100145
C 2.9709597228 1.3712986909 -0.9516074697
C 1.5597293286 -0.7880738338 1.6651287974
N 1.5602695085 -0.3652991581 0.2419637712
H 3.3459323472 1.3570934925 1.7093087369
H 2.0404841961 2.5472222369 1.7361833499
C 4.2614764901 0.5591860903 -0.8077365681
H 4.7814424835 0.7575010818 0.1370600273
H 4.9554236474 0.8069429429 -1.6183739963
H 1.8921463856 0.4441773752 3.4554752765
H 0.4368508707 0.8519093469 2.5276897587
S 0.4495197145 -1.0591715293 -0.8026224979
O 0.4294679530 -2.4899843386 -0.4885804646
O 0.7733159142 -0.5570705685 -2.1410184275
C -1.1737107922 -0.4041801831 -0.3814107230
C -1.6270994382 0.7701342239 -0.9888900490
C -1.9626771183 -1.0612186286 0.5673386873
C -2.8667210805 1.2936639066 -0.6249320991
H -1.0260418763 1.2462077804 -1.7561223653
C -3.1990961638 -0.5235441793 0.9188236720
H -1.6213022679 -1.9963081637 0.9985379418
C -3.6702717210 0.6604184406 0.3331590271
H -3.2200939355 2.2052094221 -1.1010834007
H -3.8142011169 -1.0383261141 1.6531792987
C -5.0272907621 1.2152892848 0.6956648300
H -5.2842069947 0.9983068483 1.7377864309
H -5.0685864573 2.2999510687 0.5528810191
H -5.8129331108 0.7735202629 0.0684875560
H 4.0505891821 -0.5132824023 -0.8610462861
H 3.1943462859 2.4472236347 -0.9064892277
H 0.9818631988 1.6373486012 -0.1769754143
H 2.5372797172 1.1787745384 -1.9371025367
H 2.4927454434 -1.3248128558 1.8759031693
H 0.7355291047 -1.4733199033 1.8731369275
36

C 2.6291478944 -2.2773776555 0.2178588590
C 3.6388861526 -1.1313257073 0.0371841537
C 2.7884409643 0.1696916075 -0.0579614351
C 3.2005181716 1.0520668688 -1.2446490526
C 1.3940680198 -1.7460504911 -0.5164017924
N 1.3626570973 -0.2955169588 -0.2011836238
H 4.2035128869 -1.2676432181 -0.8936098031
H 4.3659345194 -1.0777826625 0.8531842292
C 2.4410682345 2.3749324039 -1.3540151026
H 2.8242635043 2.9747648566 -2.1875451845
H 2.5320536510 2.9659566582 -0.4363773573
H 2.9875557543 -3.2303246611 -0.1854225101
H 2.3830012657 -2.4185047301 1.2748351378
S 0.3287375667 0.1175363012 1.0920050404
O 0.6705925672 1.4962868021 1.4617303160
O 0.3001283323 -0.9333709958 2.1246658531
C -1.2771163598 0.1095343932 0.2906761357
C -2.1943631687 -0.8912119378 0.6042926867
C -1.6092229833 1.1330031189 -0.6018984413
C -3.4521580518 -0.8755387000 -0.0015300233

H -1.9263174495 -1.6589898033 1.3221293422
C -2.8668956757 1.1336258802 -1.1946098037
H -0.8957771630 1.9222583996 -0.8130450573
C -3.8066815582 0.1287834273 -0.9090600129
H -4.1702409290 -1.6543800743 0.2425931364
H -3.1293548855 1.9300923796 -1.8870276332
C -5.1637756910 0.1404654622 -1.5713208757
H -5.0794665670 -0.0437323170 -2.6499100140
H -5.8209723435 -0.6277406416 -1.1529900872
H -5.6581602445 1.1113462657 -1.4491521726
H 1.3756920308 2.1963349618 -1.5283558692
H 3.0810410531 0.4743109751 -2.1722013633
H 2.8688790239 0.7595909678 0.8586539325
H 4.2774066834 1.2500137681 -1.1434580191
H 1.5005280761 -1.8480825347 -1.6036110529
H 0.4590698338 -2.2287875376 -0.2252722179
36

C 2.7620572866 2.0952699138 0.0402225121
C 3.6821286985 0.8675530824 0.1466013287
C 2.7372674945 -0.3425345645 0.3599968750
C 2.6797640814 -0.8807420777 1.7995076019
C 1.5349872845 1.5357033750 -0.6801478101
N 1.3999568617 0.1853523898 -0.0872065059
H 4.4171954514 0.9554283637 0.9527353745
H 4.2363540106 0.7397259688 -0.7900414686
C 2.2631861202 0.1300617052 2.8719635348
H 2.2104617578 -0.3533223003 3.8539823777
H 1.2769707203 0.5520518190 2.6512577692
H 2.4821572056 2.4603504442 1.0338154297
H 3.2169742573 2.9247021215 -0.5100896292
S 0.3870289883 -0.8857741787 -0.9135299651
O 0.6189586155 -2.2096981435 -0.3234626908
O 0.4965592321 -0.6971710565 -2.3688351859
C -1.2419575675 -0.3125744276 -0.4261660880
C -1.7094274472 -0.5883417376 0.8620517397
C -2.0338860536 0.3762065866 -1.3425958939
C -2.9786621756 -0.1549804097 1.2297496204
H -1.0879922077 -1.1424468229 1.5578081945
C -3.3050758372 0.8045335477 -0.9559881825
H -1.6587286990 0.5615374012 -2.3433652665
C -3.7965505019 0.5476253122 0.3290897701
H -3.3458816844 -0.3679815038 2.2309790320
H -3.9251890688 1.3428294358 -1.6685110510
C -5.1812652189 0.9906650634 0.7376463319
H -5.1763206571 1.4537139318 1.7309481785
H -5.5971335372 1.7129215216 0.0285838174
H -5.8703545065 0.1374154344 0.7834835691
H 2.9750914592 0.9600864501 2.9566949891
H 3.6749295340 -1.2837586872 2.0360454510
H 3.0447908431 -1.1709250986 -0.2867268497
H 1.9921478883 -1.7329037711 1.8066447142
H 0.6237110837 2.1162777219 -0.5028202615
H 1.7049092308 1.4774968971 -1.7635819561
36

C 2.9922731771 -0.8984906141 -1.7216054894
C 3.7209659891 0.0055926480 -0.7193593203
C 2.7286210540 0.1629368226 0.4541494102
C 2.8140497437 1.4910041509 1.2223656633
C 1.5505469473 -0.3909454537 -1.6246615573
N 1.3789249188 -0.0324241241 -0.1909097037
H 3.9301200508 0.9787078729 -1.1786939648
H 4.6741623701 -0.4078202175 -0.3751503674
C 2.4779868408 2.7473208819 0.4125869902
H 1.4769587159 2.6722603384 -0.0255228488
H 3.1892203228 2.9229203866 -0.4029338959
H 3.3970281856 -0.8363487601 -2.7370391577
H 3.0324206461 -1.9448106990 -1.3973954636
S 0.2886204870 -0.9928752200 0.6793365031
O 0.5171294189 -0.6781330318 2.0949851694
O 0.3198727223 -2.3946288800 0.2281564737
C -1.2917725252 -0.3115207589 0.1721241835
C -2.1959638609 -1.1171979128 -0.5159115452
C -1.6196738915 1.0035808418 0.5157749644
C -3.4385787401 -0.5916781219 -0.8755944923
H -1.9256434764 -2.1391521709 -0.7588701388
C -2.8614016078 1.5104470718 0.1498713801
H -0.9107190021 1.6155623509 1.0636417417
C -3.7907984145 0.7229134691 -0.5508258909
H -4.1452870825 -1.2175794370 -1.4147693099
H -3.1187086617 2.5336787737 0.4135251822
C -5.1412798310 1.2853695977 -0.9254526670
H -5.7645532151 1.4411084533 -0.0356338822
H -5.0449647217 2.2568913938 -1.4241279138
H -5.6837879739 0.6129237787 -1.5967504717
H 2.4993494008 3.6333150600 1.0568908194
H 3.8311984534 1.5712416906 1.6319779843
H 2.9011483655 -0.6462048729 1.1724216256
H 2.1395165647 1.4185738620 2.0821332339
H 1.4043818029 0.5127111289 -2.2292653480
H 0.8112976366 -1.1330763273 -1.9323963281
36

C 3.0071776338 2.0507423787 -0.2396870895
C 3.7543139147 0.7157969163 -0.1046464754
C 2.7141693493 -0.2184805539 0.5436383214
C 2.7381395640 -0.1538908743 2.0804721028
C 1.5959731370 1.6160510677 -0.6599815868
N 1.4113307190 0.2894946176 -0.0096406934
H 4.6696729310 0.7938358342 0.4908058953
H 4.0272475842 0.3373478046 -1.0968583424
C 1.6531653421 -0.9798501936 2.7736585139
H 1.6934866212 -2.0303826428 2.4668027913
H 0.6581593152 -0.5974255494 2.5259995545
H 2.9793761370 2.5765393433 0.7211569136
H 3.4572919540 2.7256943631 -0.9740290791
S 0.4345786140 -0.8108316325 -0.8530953446
O 0.6755300745 -2.1300374128 -0.2563346454
O 0.5718398559 -0.6171220403 -2.3056395367
C -1.2230364950 -0.2836060923 -0.4118647809
C -1.8287579509 -0.8086299761 0.7320355309
C -1.9008708400 0.6221033295 -1.2278011872
C -3.1171142895 -0.4004387250 1.0659988856
H -1.3017559742 -1.5417704817 1.3327824842
C -3.1893672550 1.0230777212 -0.8751348950
H -1.4336841250 0.9860881965 -2.1366626210
C -3.8145887904 0.5260276611 0.2756317711
H -3.5942402786 -0.8147025921 1.9510141599
H -3.7214849808 1.7252589575 -1.5122765225
C -5.2010748221 0.9805150858 0.6651871405
H -5.7421701566 1.3967956137 -0.1902660086
H -5.7933557288 0.1543590589 1.0732545875
H -5.1577035408 1.7598102182 1.4375916008
H 1.7703493285 -0.9352445895 3.8625304013
H 2.6625606161 0.8939800678 2.4018514381
H 2.8529547512 -1.2571813045 0.2351336488
H 3.7291952099 -0.5054935464 2.4010974165
H 0.8172612081 2.3111487425 -0.3284172693
H 1.5319505471 1.5150465381 -1.7490661749

$9_{piperidine}$

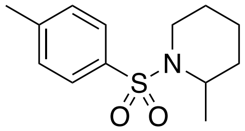

Name	E(B3LYP)	H(B3LYP)	E(RO-B2PLYP-D3)	H(RO-B2PLYP-D3)
Tosyl_2_methyl_piperidine_0000	-1110.160569	-1109.836057	-1109.745886	-1109.421374
Tosyl_2_methyl_piperidine_0006	-1110.162169	-1109.837717	-1109.745064	-1109.420612
Tosyl_2_methyl_piperidine_0002	-1110.161216	-1109.836794	-1109.744465	-1109.420043
Tosyl_2_methyl_piperidine_0007	-1110.161175	-1109.836736	-1109.744302	-1109.419863
Tosyl_2_methyl_piperidine_0003	-1110.16096	-1109.836543	-1109.743867	-1109.41945
Tosyl_2_methyl_piperidine_0021	-1110.157808	-1109.833379	-1109.743076	-1109.418647
Tosyl_2_methyl_piperidine_0001	-1110.158901	-1109.834506	-1109.742643	-1109.418248
Tosyl_2_methyl_piperidine_0012	-1110.15903	-1109.834711	-1109.741866	-1109.417547
Tosyl_2_methyl_piperidine_0028	-1110.156685	-1109.832395	-1109.739301	-1109.415011

36

C 2.0077020883 2.2430328836 0.9621452128
C 3.3558354046 2.1586386546 0.2352263449
C 2.6428348798 -0.0915492352 -0.7648029179
C 3.2224634119 1.3101854998 -1.0375502833
C 1.4327911965 0.8484130798 1.2299990712
N 1.3532898087 0.0977768132 -0.0391464933
H 4.1087756000 1.7211411533 0.9046307296
H 3.7142045000 3.1632343046 -0.0200304643
H 2.1097700491 2.7754476284 1.9158611600
H 1.2928320038 2.8061102300 0.3479787235
S 0.3239020021 -1.2489008869 0.0009862873
O 0.4351914622 -1.9703656300 1.2788105260
O 0.5313735213 -1.9603680735 -1.2665125780
C -1.2951242524 -0.4720973651 -0.0301595988
C -2.1602167560 -0.6467336912 1.0473754900
C -1.6920482822 0.2499773614 -1.1600864549
C -3.4355271905 -0.0798656963 0.9947688264
H -1.8347142969 -1.2227848289 1.9069555407
C -2.9649709016 0.8072783163 -1.1954095378
H -1.0106572912 0.3682269266 -1.9961743153
C -3.8572334252 0.6520593043 -0.1204206606
H -4.1125690145 -0.2134161887 1.8347831565
H -3.2769521358 1.3703529175 -2.0720033619
C -5.2403242816 1.2552092050 -0.1824365232
H -5.8400787183 0.7884050397 -0.9738722950
H -5.7770215331 1.1259262117 0.7621512462
H -5.1978836738 2.3285763139 -0.4027821467
H 2.3770207074 -0.5466002512 -1.7214272301
H 2.5588815788 1.8256351776 -1.7437859143
H 2.0384341418 0.3125214697 1.9746594521
H 0.4214721721 0.9325636431 1.6390751780
C 3.6303038274 -1.0302892938 -0.0513150999
H 4.1953331013 1.1979073437 -1.5317251201
H 4.5407403470 -1.1343761874 -0.6531312413
H 3.1950979557 -2.0257070067 0.0757140230
H 3.9248149327 -0.6617067781 0.9365001297
36

C 3.1931208674 1.5219200024 -1.1878704164
C 3.7956415124 1.7108978262 0.2106671346
C 2.0358707821 0.1063305599 1.1609669886
C 3.5326830705 0.4739766517 1.0829085662
C 1.7061064420 1.1531799973 -1.1109389531
N 1.5306917199 -0.0096829142 -0.2282619885
H 3.3634656531 2.6052108461 0.6807036475
H 4.8747516904 1.8921874902 0.1375396158
H 3.3047748042 2.4341699326 -1.7873530855
H 3.7225785369 0.7168050975 -1.7127826616
S 0.4016499350 -1.1802689882 -0.6268793549
O 0.6593987677 -2.3198374962 0.2593094811
O 0.4396959522 -1.3089361150 -2.0870228020
C -1.2392570701 -0.5624728864 -0.2153974321
C -1.9086076649 0.2767128994 -1.1095197284
C -1.8280777817 -0.9191506084 0.9995005908
C -3.1656847258 0.7724379320 -0.7700427253
H -1.4598355196 0.5171043388 -2.0675290832
C -3.0876885809 -0.4164851249 1.3217938369
H -1.3088020947 -1.5964796701 1.6691595562
C -3.7751604350 0.4367038847 0.4474235355
H -3.6869405215 1.4243909195 -1.4670771306
H -3.5477315958 -0.6982053418 2.2659950776
C -5.1532869373 0.9507620476 0.7898973284
H -5.3306380673 1.9415396612 0.3586053332
H -5.9311636790 0.2818034235 0.3984748109
H -5.2982463839 1.0194651666 1.8728978643
H 1.9548050368 -0.8935381934 1.5920084399
H 4.0721015277 -0.3838631462 0.6626133222
H 1.1194877736 2.0119654547 -0.7474507894
H 1.3342338882 0.8827682611 -2.1000846592
C 1.2243605400 1.0831862370 2.0296763316
H 3.9134519500 0.6308736744 2.0998736410
H 1.2547408920 2.1085371071 1.6468046256
H 1.6303509885 1.0982393647 3.0477991818
H 0.1752148488 0.7793134741 2.0898910093
36

C 3.7563303416 -0.6617215261 0.2025122791
C 4.0448787583 0.8414195379 0.0785400430
C 1.5620203324 1.2440714351 -0.4235170925
C 2.7902942194 1.6644993083 0.4119286807
C 2.5451331530 -1.0548541979 -0.6517760041
N 1.3491384600 -0.2303023148 -0.3726745665
H 4.3868683473 1.0648441395 -0.9422484793
H 4.8644305412 1.1298355135 0.7477410837
H 4.6265600634 -1.2521404648 -0.1122869977
H 3.5555072891 -0.9179672880 1.2510498194
S 0.3263241268 -0.7794349722 0.8599737229
O 0.5265462723 -0.0345325665 2.1146119890
O 0.4284853606 -2.2441755213 0.8582292631
C -1.2865608758 -0.3064672696 0.2256953148
C -1.7346552167 -0.8333188819 -0.9898481412
C -2.1020820874 0.5279392761 0.9863985115
C -3.0069597290 -0.5049328795 -1.4435781870
H -1.0910876699 -1.4877693420 -1.5685826643
C -3.3772558595 0.8477120456 0.5151283342
H -1.7371971645 0.9162209894 1.9311534639
C -3.8491785775 0.3395506811 -0.6998378301
H -3.3571860102 -0.9107624071 -2.3898570841
H -4.0141967038 1.5017378562 1.1054075610
C -5.2329074095 0.6749257245 -1.2031758061

H -5.8764738365 -0.2138582386 -1.2115658595
H -5.7162100393 1.4294290566 -0.5753228006
H -5.2021142566 1.0585909991 -2.2298678851
H 0.6733060178 1.7017600313 0.0228923316
H 2.5404065688 1.5269264025 1.4700119998
H 2.7762612459 -0.9226501015 -1.7165037873
H 2.2692584303 -2.0997357869 -0.5048779118
C 1.6224425314 1.7039447521 -1.8866987652
H 2.9797409228 2.7347184881 0.2581927702
H 0.7211899170 1.3887720164 -2.4213920843
H 2.4909081423 1.3000636308 -2.4168908567
H 1.6881572302 2.7969952256 -1.9303977572
36

C 2.8752137335 1.7994329590 1.1930896488
C 3.2898641189 2.1251572624 -0.2488698914
C 1.7876515588 0.2246426140 -1.0797137587
C 2.1914949032 1.7053640391 -1.2379461834
C 2.4706999809 0.3284167793 1.3400141214
N 1.4272407372 0.0161980483 0.3458718939
H 4.2285572787 1.6065783750 -0.4865159944
H 3.4947572783 3.1975237268 -0.3533857398
H 3.6934834873 2.0167590213 1.8910307790
H 2.0231445076 2.4285245609 1.4827359578
S 0.3213567191 -1.2050038845 0.6995001439
O 0.2888714306 -1.3188727645 2.1624848947
O 0.5494222512 -2.3991735907 -0.1290536296
C -1.2278806373 -0.4822409059 0.1446820523
C -1.9218360787 -1.0669645846 -0.9120930138
C -1.7461378110 0.6317916543 0.8125976501
C -3.1429514861 -0.5190933559 -1.3114098947
H -1.5087015183 -1.9401953530 -1.4052422006
C -2.9637584609 1.1630594246 0.4034878045
H -1.2016040458 1.0696005963 1.6429504337
C -3.6821231410 0.5978114057 -0.6642564856
H -3.6847125184 -0.9724428644 -2.1377876348
H -3.3689293512 2.0295305727 0.9210565513
C -5.0113534497 1.1787166449 -1.0840994400
H -4.9469070422 2.2641350432 -1.2238650619
H -5.3640997648 0.7373857984 -2.0210941881
H -5.7796667959 0.9978764147 -0.3215067917
H 0.8688926245 0.0653319628 -1.6555388759
H 1.3007039972 2.3242177170 -1.0702793078
H 3.3487587212 -0.3223052964 1.2096028424
H 2.0595280041 0.1295003307 2.3300307189
C 2.8521176443 -0.7538906863 -1.6039933555
H 2.5171660635 1.8775045907 -2.2712771590
H 3.0267032860 -0.5712457562 -2.6709018354
H 2.5148472387 -1.7849667768 -1.4777646522
H 3.8112002020 -0.6386178046 -1.0887823015
36

C 2.5044055501 1.9345380907 -0.8764874321
C 3.8306119062 1.4010108276 -0.3150151494
C 2.5403070287 -0.4540835833 0.8985472218
C 3.7297202106 -0.1041626021 -0.0207779760
C 1.3367290893 1.5787069851 0.0527629013
N 1.2719173191 0.1389691303 0.3861691575
H 4.0906889849 1.9484012905 0.6021589892
H 4.6450384536 1.5871352121 -1.0254658358
H 2.5458828200 3.0246340018 -0.9995727542
H 2.3137531357 1.4984156368 -1.8634031578
S 0.3563502637 -0.8094729653 -0.6772981479
O 0.5938413389 -2.2073300086 -0.2952153533
O 0.5362554566 -0.3936906969 -2.0785698437
C -1.3188299626 -0.3576400460 -0.2115891512
C -2.1662613538 0.2022652924 -1.1646354885
C -1.7750942695 -0.6308312432 1.0816975791
C -3.4835405865 0.5032193285 -0.8113850980
H -1.7937578668 0.3939572115 -2.1651106004
C -3.0893372274 -0.3258244433 1.4158854825
H -1.1057585018 -1.0738706818 1.8118358042
C -3.9650779846 0.2454833379 0.4764871386
H -4.1460541484 0.9430587353 -1.5526560301
H -3.4465057962 -0.5349808898 2.4216077768

C -5.3927701200 0.5597984934 0.8551878425
H -5.9128401428 1.0914861942 0.0527720407
H -5.9564281961 -0.3573430087 1.0678477221
H -5.4386441163 1.1811898015 1.7574861945
H 2.3910665292 -1.5351891333 0.8838298880
H 3.6054580910 -0.6471251562 -0.9667655499
H 1.4275577650 2.1195767860 1.0032606560
H 0.3784806958 1.8755741921 -0.3830383190
C 2.7491705357 -0.0321697838 2.3596984190
H 4.6579661034 -0.4705537142 0.4362839262
H 3.6343701066 -0.5349176441 2.7655551315
H 1.8833061159 -0.3178268800 2.9649509125
H 2.9019645711 1.0461236207 2.4737713057
36

C 1.6707540932 1.7395269336 1.4863856841
C 2.3391988807 2.3866628329 0.2649198775
C 2.2232745738 0.1350477433 -0.9640632475
C 1.9381636983 1.6516163323 -1.0237745603
C 1.9860801441 0.2394142931 1.5450824087
N 1.6672482102 -0.4465006484 0.2823053083
H 3.4308282714 2.3622893310 0.3898603746
H 2.0602230670 3.4447273718 0.1916274913
H 2.0121475755 2.2161681659 2.4142133093
H 0.5831651652 1.8806408981 1.4308887736
S 0.3833740419 -1.5145496775 0.2075339800
O 0.2938975484 -2.1480286429 1.5268434415
O 0.5667195114 -2.2945175220 -1.0205463142
C -1.1328886618 -0.5659205403 -0.0102126615
C -1.8349100276 -0.1166697712 1.1108690502
C -1.5855502974 -0.2670654976 -1.2972098321
C -2.9847328910 0.6506159003 0.9348058909
H -1.4937928041 -0.3863242586 2.1048143763
C -2.7372612778 0.5022628171 -1.4549952079
H -1.0533108490 -0.6537297743 -2.1598366994
C -3.4536865355 0.9744691463 -0.3464494562
H -3.5327967126 0.9965677070 1.8080632637
H -3.0908302877 0.7317838929 -2.4574322884
C -4.7198236955 1.7776819029 -0.5268059926
H -4.8663212811 2.4858809295 0.2954059350
H -4.7068006876 2.3422453752 -1.4647939679
H -5.6008473378 1.1227595469 -0.5527487296
H 1.7056948596 -0.3545586247 -1.7916207069
H 0.8631123007 1.7957227229 -1.1948039040
H 3.0565677465 0.0924407524 1.7445662253
H 1.4481269055 -0.2646806193 2.3491572304
C 3.7127050770 -0.2177990283 -1.0928460576
H 2.4588960309 2.0805664821 -1.8892265068
H 4.1078331654 0.1640882965 -2.0413817829
H 3.8396382969 -1.3039342092 -1.0755183178
H 4.3162328270 0.2101710493 -0.2854874064
36

C 3.2756613836 1.6894243759 -1.0463629741
C 3.8387778027 1.7989922301 0.3716240266
C 1.9869781403 0.1999031177 1.1639808373
C 2.7282444922 1.5330206112 1.3902657835
C 2.5809936895 0.3433461348 -1.2429665876
N 1.4867841361 0.1784704399 -0.2475229122
H 4.2837845354 2.7866789233 0.5432047205
H 4.6440121099 1.0616652602 0.5019446759
H 2.5539856867 2.4966402968 -1.2286049317
H 4.0704264538 1.7864663736 -1.7964013440
S 0.4291915559 -1.1061616114 -0.6703758348
O 0.6945757116 -2.3080123153 0.1370128789
O 0.4612299127 -1.1994101195 -2.1356959037
C -1.1874079426 -0.4765940101 -0.2066055565
C -1.5923713812 0.7950956808 -0.6200337735
C -2.0635054227 -1.3195929362 0.4739858615
C -2.8833225149 1.2239677815 -0.3282969465
H -0.8980982316 1.4419669457 -1.1456134955
C -3.3560538745 -0.8747019812 0.7542570422
H -1.7295461973 -2.3036413533 0.7853310671
C -3.7868564006 0.3972688354 0.3592490947
H -3.1976633996 2.2176035541 -0.6390384360

H -4.0391558933 -1.5286441617 1.2906215876
C -5.1928987700 0.8681997952 0.6454003502
H -5.6766981729 0.2481475901 1.4063104170
H -5.2043323680 1.9065716853 0.9952707579
H -5.8139281857 0.8256497969 -0.2589002495
H 2.7011021455 -0.6300700410 1.2910726855
H 3.1361505214 1.5211804672 2.4086044578
H 2.1432993497 0.2711613934 -2.2375083127
H 3.3180438883 -0.4706795740 -1.1426872871
C 0.8847786772 0.0579029076 2.2184300542
H 1.9894048356 2.3452995522 1.3498502886
H 1.3398316480 0.1709535582 3.2086958393
H 0.4008145934 -0.9188552881 2.1867034220
H 0.1250148469 0.8378091611 2.1036948648
36

C 3.5573334715 -0.0695705479 1.4388148975
C 4.1869958381 0.6924391464 0.2634512941
C 1.8531510357 1.3210109475 -0.5218044632
C 3.2286595225 0.7661426753 -0.9362293980
C 2.1697587706 0.4886774673 1.7768094236
N 1.2641536009 0.5280945727 0.6028401902
H 5.1322956317 0.2228411539 -0.0343286856
H 4.4351701959 1.7141842547 0.5890454226
H 3.4587583328 -1.1343760138 1.1918674331
H 4.1989406697 -0.0058714687 2.3273848372
S 0.4480613938 -0.9237908340 0.2701042417
O 0.4459215169 -1.6892261943 1.5240730693
O 0.9367882679 -1.5572959808 -0.9660690628
C -1.2365256457 -0.3829769682 -0.0448737889
C -1.8857689389 0.4574603712 0.8637905971
C -1.9115397689 -0.8945916036 -1.1507758267
C -3.2171726513 0.7947759035 0.6455117635
H -1.3484171505 0.8513481934 1.7201044407
C -3.2484338593 -0.5476012926 -1.3533897693
H -1.3904304224 -1.5470797020 -1.8429124188
C -3.9213433151 0.2972175366 -0.4637604485
H -3.7214390488 1.4556415982 1.3467870577
H -3.7747899285 -0.9416822943 -2.2191709408
C -5.3716946953 0.6606678155 -0.6759988099
H -5.7278720365 0.3327382501 -1.6572155804
H -6.0112264755 0.1924926255 0.0832103048
H -5.5273678513 1.7434280456 -0.6037610772
H 2.0140462600 2.3073577707 -0.0615515299
H 3.6521968950 1.4119488128 -1.7168079140
H 1.6885158273 -0.0901707340 2.5645882894
H 2.2526215610 1.5279795869 2.1233018844
C 0.9095678934 1.5324971173 -1.7067889404
H 3.0872575102 -0.2278294528 -1.3758214959
H -0.0551103652 1.9330215803 -1.3814665953
H 0.7393948384 0.6081642549 -2.2638221524
H 1.3660633458 2.2584424876 -2.3886312774
36

C 2.9276430831 1.3962553243 1.7460833269
C 3.2472406992 2.2756706441 0.5373052229
C 1.8330157926 0.6872683865 -0.9187051478
C 3.1702944997 1.4402795334 -0.7417803873
C 1.5858866777 0.6920549209 1.5494356005
N 1.5994277769 -0.1289788777 0.3132126693
H 4.2399041094 2.7320294198 0.6334116763
H 2.5229317509 3.1017078673 0.4839494097
H 3.7102627998 0.6392491095 1.8813431344
H 2.8813484645 1.9895159553 2.6678587717
S 0.4206428612 -1.3658613163 0.3227452202
O 0.3931745445 -1.8716493270 1.6986650524
O 0.6957219367 -2.2663870960 -0.7968635548
C -1.1765926350 -0.5910083823 0.0163339978
C -1.8576859773 0.0211951496 1.0733929388
C -1.7254193186 -0.6009250635 -1.2671032966
C -3.0807066242 0.6416316348 0.8304099829
H -1.4460766496 -0.0144750883 2.0761994367
C -2.9524062324 0.0219361827 -1.4919265530
H -1.2099774946 -1.1158624104 -2.0702535941
C -3.6472275564 0.6547127899 -0.4527030475
H -3.6101109701 1.1152291208 1.6538251262
H -3.3805895160 0.0084503480 -2.4913133368
C -4.9903202000 1.3004969680 -0.6967793012
H -5.8084248974 0.6083268629 -0.4575039505
H -5.1266740108 2.1904332770 -0.0731843183
H -5.1089529498 1.5964429158 -1.7439230188
H 1.0240481049 1.4357036440 -0.9985430822
H 3.3127021114 2.0774156853 -1.6232245490
H 1.3811642710 0.0267400894 2.3869286314
H 0.7806086581 1.4465335358 1.4988704129
C 1.8985137864 -0.1111363011 -2.2251446362
H 3.9784781461 0.6967466351 -0.7470829151
H 0.9412797852 -0.5386149432 -2.5198251426
H 2.6187500301 -0.9296141280 -2.1469183033
H 2.2237333885 0.5696091213 -3.0201410731
36

C 1.8552437830 2.4592597640 -0.6149920679
C 3.3480921164 2.2640611383 -0.3475619780
C 2.8588472118 -0.1933122871 0.2431821762
C 3.5515008353 1.1233782105 0.6521689866
C 1.2150432801 1.1347677143 -1.0267876407
N 1.3996431243 0.1129590330 0.0400458722
H 3.8037742883 3.1877315853 0.0295124642
H 3.8581265920 2.0215327362 -1.2909488569
H 1.3546126933 2.8332316048 0.2873516559
H 1.6883684940 3.1952490137 -1.4113338863
S 0.3550636753 -1.2269460240 -0.2450910968
O 0.5131040792 -2.1830026804 0.8548310359
O 0.4742011433 -1.6925215485 -1.6374707438
C -1.2570853374 -0.4543844197 -0.0533588247
C -2.1449930351 -0.4504711789 -1.1269644246
C -1.6341998020 0.0495075206 1.1946490099
C -3.4244035472 0.0781856360 -0.9481775343
H -1.8315877691 -0.8577029041 -2.0825302076
C -2.9112160873 0.5776929919 1.3527353293
H -0.9343092486 0.0278922709 2.0233196006
C -3.8270419900 0.5982348565 0.2876062343
H -4.1197799379 0.0843786276 -1.7838390613
H -3.2073471077 0.9768261181 2.3200150194
C -5.2189911481 1.1496849504 0.4848469789
H -5.2002821330 2.0948681064 1.0390898213
H -5.7199084791 1.3267027404 -0.4717745430
H -5.8410076320 0.4512876235 1.0596168370
H 3.2803529266 -0.5241701832 -0.7213626140
H 4.6206845775 0.9132450483 0.7775030088
H 0.1440603798 1.2765923864 -1.1876093490
H 1.6471076447 0.7789416454 -1.9749634216
C 3.1538357376 -1.2603470697 1.3029156171
H 3.1638242329 1.4177288835 1.6369379651
H 2.6489600515 -1.0250406224 2.2447435848
H 4.2351742774 -1.2678702510 1.4826851965
H 2.8475042405 -2.2603322313 1.0000036521

10imine

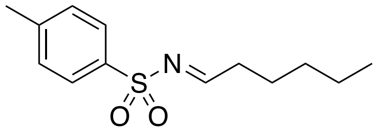

Name	E(B3LYP)	H(B3LYP)	E(RO-B2PLYP-D3)	H(RO-B2PLYP-D3)
Tosyl_imine_hexane_0044	-1110.152647	-1109.83051	-1109.727493	-1109.405356
Tosyl_imine_hexane_0002	-1110.151649	-1109.829299	-1109.727274	-1109.404924
Tosyl_imine_hexane_0055	-1110.152738	-1109.830539	-1109.726976	-1109.404777
Tosyl_imine_hexane_0083	-1110.151245	-1109.829033	-1109.726615	-1109.404403
Tosyl_imine_hexane_0089	-1110.151817	-1109.829443	-1109.726634	-1109.40426
Tosyl_imine_hexane_0075	-1110.15114	-1109.828939	-1109.72628	-1109.404079
Tosyl_imine_hexane_0008	-1110.150415	-1109.828194	-1109.726161	-1109.40394
Tosyl_imine_hexane_0077	-1110.151357	-1109.829098	-1109.725951	-1109.403692
Tosyl_imine_hexane_0067	-1110.150382	-1109.827956	-1109.726076	-1109.40365

36

C 3.0601229899 -1.3918718058 -0.9208074569
C 3.9611945806 -0.3208032816 -0.2704138800
C 3.3659381048 1.0926531116 -0.3060477775
C 4.2868504638 2.1503373918 0.3153295965
C 1.7897072923 -1.6182448202 -0.1578498145
N 0.6457286614 -1.5164061102 -0.7202959567
H 4.9271437374 -0.3252253940 -0.7918830873
H 4.1742535724 -0.6070545449 0.7695777353
H 3.1465090955 1.3659797930 -1.3485218116
C 3.6944814233 3.5625851810 0.2744845188
H 4.3747905602 4.2939954535 0.7250543667
H 2.7445625295 3.6098765700 0.8209192524
H 2.8163208757 -1.1293235968 -1.9554701577
H 3.6033648352 -2.3485415355 -0.9399061669
S -0.7087781226 -1.8853585025 0.3009776165
O -0.2965622975 -2.0031447456 1.7100398415
O -1.4120326582 -2.9862533068 -0.3624544229
C -1.6654409757 -0.3864578153 0.1166501782
C -2.3582163936 -0.1609878133 -1.0755269199
C -1.7171961052 0.5298979080 1.1660109076
C -3.1026853683 1.0065859037 -1.2119041470
H -2.3168499102 -0.8935508085 -1.8746199856
C -2.4723220874 1.6920597771 1.0117198512
H -1.1813800677 0.3253796672 2.0867690392
C -3.1738050526 1.9488350348 -0.1734885318
H -3.6423517401 1.1896118774 -2.1379437771
H -2.5194293676 2.4093567786 1.8273461264
C -4.0115816933 3.1959624347 -0.3251418116
H -3.9298787200 3.6127866679 -1.3348771999
H -5.0735353283 2.9793388921 -0.1496499055
H -3.7124422952 3.9700150393 0.3883797089
H 3.4984240405 3.8805512614 -0.7568350895
H 5.2527814935 2.1426157255 -0.2095652544
H 2.3985757739 1.1014742535 0.2166408569
H 4.5048752890 1.8742414724 1.3568136864
H 1.8813828391 -1.8727065483 0.9067587929
36

C 3.1405079587 -1.5048346821 -0.8706249110
C 3.6934716905 -0.3452353646 -0.0330661317
C 3.2200801336 1.0409216434 -0.4922592499
C 3.8167091796 2.1826334236 0.3406149474
C 1.6902712524 -1.8436356873 -0.6782781968
N 0.9729354424 -1.2842898156 0.2200215486
H 4.7899896593 -0.3846221058 -0.0797189904
H 3.4168023439 -0.4994679017 1.0167868262
H 3.4907171006 1.1851261560 -1.5495729220
C 3.3544325985 3.5686136906 -0.1203342779
H 3.7939394943 4.3622150650 0.4946519634
H 2.2634023619 3.6628637559 -0.0570188215
H 3.2973521299 -1.3243927667 -1.9454602672
H 3.6971533662 -2.4323372171 -0.6609994202
S -0.6488384295 -1.8840366876 0.3467121620
O -0.7852887297 -2.3554964823 1.7270904798
O -0.9764798877 -2.7708942723 -0.7822599459
C -1.5694054996 -0.3635773665 0.1558518635
C -2.2435879126 -0.1130952243 -1.0385150815
C -1.6098386506 0.5475548447 1.2142947997
C -2.9642095130 1.0731723343 -1.1716464091
H -2.2070189388 -0.8421326335 -1.8408955647
C -2.3308691062 1.7277574264 1.0604835554
H -1.0913886183 0.3265526787 2.1412887191
C -3.0197504824 2.0086921748 -0.1301100652
H -3.4943803140 1.2734169598 -2.0994122072
H -2.3650157665 2.4422071540 1.8794215615
C -3.8250461212 3.2783844262 -0.2698237991
H -3.3225338439 4.1261014430 0.2083292141
H -3.9962009153 3.5318335648 -1.3206599479
H -4.8083144435 3.1738470788 0.2074449934
H 3.6422168975 3.7568948426 -1.1621623358
H 4.9142101569 2.1296988839 0.2984917048
H 2.1253846456 1.0890305278 -0.4401696775
H 3.5451329783 2.0382217588 1.3957034777
H 1.2720592501 -2.6145571302 -1.3384804565
36

C 2.7620039515 0.5548215495 1.5824505273
C 4.1450843065 -0.0978847854 1.3480563805
C 4.4927575507 -0.3733587705 -0.1242911682
C 4.5749892020 0.8765237372 -1.0116082923
C 1.6378929544 -0.3210910392 1.1200884694
N 0.8292755463 0.0620099386 0.2057904971
H 4.1927346547 -1.0392163001 1.9123442017
H 4.9041418295 0.5623010428 1.7879264877
H 5.4596399211 -0.8950985175 -0.1522992163
C 5.0359792982 0.5576717479 -2.4377436956
H 6.0318855641 0.0974157816 -2.4400442288
H 5.0855073051 1.4635147364 -3.0524341750
H 2.6351853646 0.7208746395 2.6622909309
H 2.6885748647 1.5261380913 1.0855766585
S -0.3591587596 -1.1134379293 -0.2628757844
O -0.3790797359 -2.2520132831 0.6710880380
O -0.1468096258 -1.3317541125 -1.6963668556
C -1.8578606783 -0.1672830478 -0.0322617460
C -2.1852511420 0.8288965903 -0.9555194835
C -2.6840972454 -0.4425491687 1.0564178253
C -3.3545591961 1.5603107484 -0.7714044749
H -1.5361967670 1.0175211096 -1.8040699880
C -3.8537340461 0.2984524911 1.2212622314
H -2.4139713650 -1.2295463621 1.7523510262

C -4.2067789278 1.3070635763 0.3151771260
H -3.6143872212 2.3387171922 -1.4847329630
H -4.5038797744 0.0871703816 2.0666080191
C -5.4883713841 2.0876725779 0.4835492308
H -6.2780871444 1.6909490310 -0.1679682588
H -5.8567017484 2.0377702356 1.5129150315
H -5.3523517462 3.1422697941 0.2204942811
H 4.3462735928 -0.1397974797 -2.9278210709
H 5.2653361916 1.6009483912 -0.5547131023
H 3.7606380230 -1.0713626443 -0.5544017440
H 3.5939598196 1.3665938701 -1.0545431409
H 1.5413721853 -1.3096292280 1.5887775692
36

C 3.2942183716 -1.2873815813 0.3736427028
C 3.9933629824 -0.3954258465 -0.6781491598
C 3.3183008700 0.9634190887 -0.9285282688
C 3.2674176327 1.8934450451 0.2915819130
C 1.8977828964 -1.6517043850 -0.0297031643
N 0.8946273940 -1.3527154781 0.7053591253
H 4.0545038874 -0.9467040677 -1.6263631357
H 5.0285034668 -0.2362330604 -0.3488578011
H 3.8601101053 1.4690813921 -1.7400802875
C 2.6733316163 3.2670224355 -0.0377607414
H 2.6480669010 3.9131027848 0.8469585297
H 1.6460262449 3.1730619896 -0.4104724453
H 3.8664909869 -2.2212882690 0.4736686296
H 3.2705850066 -0.8093312575 1.3568501656
S -0.6432876661 -1.8908111943 0.1050777965
O -1.2048554301 -2.7371757287 1.1607849787
O -0.5287145233 -2.3924829734 -1.2748982350
C -1.5288678192 -0.3379752793 0.0693800531
C -2.0042927426 0.2039142586 1.2658157337
C -1.7362223213 0.3083110235 -1.1487332892
C -2.6885844454 1.4153398854 1.2322254635
H -1.8448569533 -0.3224486047 2.2009145121
C -2.4264943054 1.5196636245 -1.1620615170
H -1.3701810396 -0.1401712242 -2.0661855894
C -2.9119067100 2.0906435474 0.0220505086
H -3.0599788767 1.8436858911 2.1600287065
H -2.5942283704 2.0273211345 -2.1087164826
C -3.6842114935 3.3880042942 -0.0025331082
H -3.4643117361 3.9693276525 -0.9034240834
H -4.7660403282 3.2008605901 0.0115594766
H -3.4523337415 4.0088135164 0.8695137632
H 3.2608635412 3.7800108488 -0.8092820567
H 4.2832557938 2.0189396083 0.6938185330
H 2.2961616185 0.8051238208 -1.3020524350
H 2.6738540906 1.4287453033 1.0891840006
H 1.7674756712 -2.1722514083 -0.9879839112
36

C 3.0993549077 -1.0869897542 0.9763639096
C 3.9319097249 -0.0345204387 0.2135980478
C 3.2704796861 1.3503070440 0.1676718423
C 4.1266607514 2.4375685435 -0.5022237970
C 1.8154129750 -1.4236629326 0.2794699031
N 0.6883553254 -1.3365492911 0.8774115403
H 4.1256081913 -0.3996831272 -0.8036535860
H 4.9116818670 0.0453000638 0.7029118932
H 2.3031493952 1.2810741507 -0.3509172947
C 4.3715796455 2.2220939775 -2.0007385813
H 4.9366627977 3.0572926095 -2.4296500475
H 3.4238722239 2.1460993722 -2.5485136358
H 3.6834014978 -2.0167472157 1.0488396802
H 2.8783028284 -0.7551144671 1.9961684348
S -0.6814539453 -1.8386631949 -0.0629905138
O -1.3045994880 -2.9236690561 0.6993386178
O -0.3147093816 -2.0330445853 -1.4761875608
C -1.7047412307 -0.3778514764 0.0582917731
C -1.8397441020 0.4608888031 -1.0470778476
C -2.3647568405 -0.1042674723 1.2589483110
C -2.6461478240 1.5936597083 -0.9413821570
H -1.3274667940 0.2193450362 -1.9722548876
C -3.1614630942 1.0331806539 1.3460207220

H -2.2581272998 -0.7774471158 2.1029729327
C -3.3168309549 1.8974500011 0.2505299562
H -2.7582299632 2.2502820682 -1.8006087029
H -3.6761715396 1.2534671610 2.2781384190
C -4.2103241980 3.1106477271 0.3510117687
H -3.9759599956 3.8473443002 -0.4236494960
H -5.2655967607 2.8320221177 0.2316225947
H -4.1140042313 3.5991275752 1.3267603853
H 4.9421049351 1.3072761185 -2.1971703302
H 5.0913899651 2.5104131665 0.0202737754
H 3.0390374858 1.6617019771 1.1958820949
H 3.6308146780 3.4066714770 -0.3586359968
H 1.8819373456 -1.7471086119 -0.7681629281
36

C 3.1200499600 -1.4691683283 -0.9772445364
C 3.6747580535 -0.1929648915 -0.3319861348
C 3.1314996888 1.1013923640 -0.9562614742
C 3.7478954141 2.3848499525 -0.3753241349
C 1.6940687068 -1.8175983620 -0.6602941649
N 1.0109622103 -1.1688887569 0.2039023699
H 4.7684882105 -0.2075578141 -0.4335002624
H 3.4556808015 -0.2180895639 0.7408665330
H 3.3237244095 1.0769401579 -2.0390817202
C 3.3963308319 2.6479242237 1.0940310890
H 3.8137715341 3.6031767234 1.4326437860
H 3.7866574740 1.8666478727 1.7555786922
H 3.2147318427 -1.4301584537 -2.0735429460
H 3.7179722301 -2.3448747007 -0.6775773089
S -0.5813531546 -1.7863645515 0.5000342906
O -0.6231150443 -2.0913189611 1.9323908129
O -0.9475962836 -2.8100028491 -0.4931535100
C -1.5551800198 -0.3203999895 0.1865086041
C -1.5462403812 0.7179458221 1.1215681315
C -2.3183638157 -0.2374378936 -0.9771356414
C -2.3083394711 1.8552922018 0.8729634974
H -0.9580654465 0.6270805346 2.0286417495
C -3.0790944506 0.9086887961 -1.2059554061
H -2.3180269950 -1.0623255988 -1.6815573572
C -3.0867071592 1.9687774081 -0.2900666312
H -2.3045107666 2.6680426508 1.5952630051
H -3.6785695013 0.9786195800 -2.1101991075
C -3.9332953475 3.1959162284 -0.5300623537
H -4.1974689441 3.3011965995 -1.5868789223
H -4.8703521283 3.1438199769 0.0397182984
H -3.4145653497 4.1080062931 -0.2155131544
H 2.3094432247 2.6877799994 1.2368235879
H 3.4095441392 3.2371483488 -0.9798247950
H 2.0417885200 1.1358559968 -0.8327454535
H 4.8410653573 2.3472069168 -0.4900732880
H 1.2643479467 -2.6744396519 -1.1953001570
36

C 3.2610592367 -1.2652800707 -0.4161956929
C 3.6407031372 -0.3126342207 0.7262614662
C 3.1827633747 1.1460004444 0.5502259704
C 3.8451781140 1.8939596197 -0.6156666583
C 1.8104107825 -1.6354899279 -0.5303883530
N 0.9443818584 -1.2806469889 0.3407068582
H 4.7335939656 -0.3339340175 0.8338444515
H 3.2249279869 -0.7083582371 1.6596628934
H 2.0918522086 1.1766268455 0.4387577067
C 3.4169348529 3.3632714635 -0.6964498323
H 3.9014173750 3.8769517096 -1.5346328803
H 3.6792017069 3.9024872908 0.2221072439
H 3.5795478369 -0.8743429550 -1.3934295754
H 3.8066727919 -2.2169429965 -0.3120712961
S -0.6561534956 -1.8940047922 0.0800741674
O -0.9872882079 -2.6506256528 1.2901138650
O -0.7760624906 -2.5183197394 -1.2482434976
C -1.5871883457 -0.3679558930 0.0726508630
C -1.8092932859 0.3023382678 1.2783452729
C -2.0895530080 0.1251572843 -1.1306948200
C -2.5380599889 1.4876507785 1.2661260022
H -1.4229312793 -0.1060669911 2.2061766082

C -2.8213730949 1.3121151538 -1.1215472038
H -1.9138305947 -0.4204081406 -2.0516321013
C -3.0566552425 2.0097458732 0.0704342756
H -2.7127349400 2.0157062045 2.2004203752
H -3.2183562856 1.7006541543 -2.0560920670
C -3.8733118761 3.2798553166 0.0781001525
H -3.4649100500 4.0143404438 0.7806809281
H -3.9091466778 3.7402891959 -0.9140996625
H -4.9081746163 3.0791198541 0.3851126058
H 2.3320951034 3.4518626132 -0.8324391759
H 3.6052290763 1.4009866545 -1.5679709564
H 3.4067791522 1.6846879772 1.4817457993
H 4.9383872860 1.8365783090 -0.5101861116
H 1.5247000521 -2.2476001755 -1.3958296133
36

C 2.7555346629 1.3822785188 1.0774909906
C 4.2108946456 0.8613256653 1.1120115168
C 4.7957948983 0.4151254440 -0.2392139282
C 4.2460454940 -0.9018197884 -0.8071644522
C 1.7152251082 0.3161054107 0.9134299692
N 0.8010474240 0.4077754125 0.0232640324
H 4.2864125088 0.0391699355 1.8383465343
H 4.8319707784 1.6735956467 1.5095270261
H 4.6533240586 1.2192178648 -0.9762231345
C 4.9732482183 -1.3381189780 -2.0838837599
H 4.5645300636 -2.2778658036 -2.4706155531
H 6.0447892948 -1.4872615913 -1.9006979028
H 2.5478845159 1.8709528473 2.0423429756
H 2.6246348394 2.1371502065 0.2949153704
S -0.2774491877 -0.9466088973 -0.0589965020
O -0.1459538923 -1.8088932010 1.1278242975
O -0.0965443173 -1.5027114175 -1.4035753576
C -1.8553133423 -0.1112043273 0.0207062939
C -2.3099077345 0.5972608180 -1.0944916046
C -2.6156440845 -0.1878142824 1.1866175849
C -3.5407090439 1.2425561193 -1.0270126736
H -1.7095766142 0.6326569603 -1.9973967913
C -3.8486210027 0.4620076205 1.2330768726
H -2.2459086013 -0.7542606605 2.0345974247
C -4.3292997000 1.1843729440 0.1332130015
H -3.8995546575 1.7978729437 -1.8902234293
H -4.4477470457 0.4042001434 2.1384090002
C -5.6770174528 1.8638605664 0.1796364181
H -6.4401156040 1.2574168407 -0.3256978894
H -6.0121060154 2.0213380744 1.2095075311
H -5.6524847495 2.8365434200 -0.3236323544
H 4.8735401276 -0.5829532416 -2.8732069252
H 3.1755432848 -0.8094392076 -1.0238892249
H 5.8831995151 0.3114127661 -0.1144956699
H 4.3411683355 -1.6902486016 -0.0464658955
H 1.7573243446 -0.5376806582 1.6027327345
36

C 3.1539182216 -1.3746505302 -0.9983616489
C 4.2138450676 -0.4405089958 -0.3736331760
C 3.9183528278 1.0659483875 -0.4691226683
C 2.7711776876 1.5788563361 0.4130573800
C 1.9027666239 -1.5422122907 -0.1892626658
N 0.7455402362 -1.4708410705 -0.7298848798
H 5.1631531984 -0.6403683149 -0.8855883224
H 4.3725070349 -0.7211601217 0.6777491779
H 4.8354142094 1.6070468715 -0.1956872720
C 2.6152157862 3.1022450996 0.3487342585
H 1.7890306270 3.4448107932 0.9816895900
H 2.4093918738 3.4357939070 -0.6757500136
H 2.8880435198 -1.0562290719 -2.0121220928
H 3.5952884364 -2.3801628017 -1.0830137428
S -0.5837092899 -1.7979369591 0.3352709608
O -0.1356049804 -1.8819209270 1.7358966385
O -1.3161401312 -2.9081225717 -0.2790520306
C -1.5297786458 -0.2938253321 0.1354760709
C -2.2783105785 -0.1079580742 -1.0294147030
C -1.5144574060 0.6681444225 1.1448712536
C -3.0113868671 1.0652293250 -1.1795935766
H -2.2883516661 -0.8753870074 -1.7961275085
C -2.2583795983 1.8357443929 0.9770452300
H -0.9377219760 0.4936599303 2.0468927151
C -3.0152419723 2.0528832797 -0.1818095104
H -3.5948336519 1.2170636164 -2.0844844952
H -2.2537260938 2.5881907200 1.7617550216
C -3.8409563903 3.3064520730 -0.3460927409
H -4.8950391180 3.1166965479 -0.1044823905
H -3.4920973905 4.1066089917 0.3140525929
H -3.8075662993 3.6756218268 -1.3768803328
H 3.5276954643 3.6096890453 0.6853490068
H 2.9514719089 1.2736654729 1.4539993158
H 3.7161788506 1.3272165937 -1.5183327506
H 1.8252786540 1.1112615566 0.1138789590
H 2.0197148030 -1.7552373915 0.8817032535
36

C 3.2322494347 -1.0629643157 -0.4134357088
C 3.9562928974 -0.1091267135 0.5632564113
C 3.2506891696 1.2393433318 0.7923054640
C 3.0498749108 2.1124719051 -0.4587106842
C 1.8769938058 -1.4695387376 0.0804114509
N 0.8247048586 -1.2604595615 -0.6159703141
H 4.9714185609 0.0555913431 0.1827184132
H 4.0750773631 -0.6147544638 1.5312386130
H 2.2702075037 1.0582151783 1.2541937679
C 4.3502273810 2.5341399097 -1.1530537522
H 4.8996032554 1.6752230783 -1.5558805917
H 5.0189986839 3.0576016389 -0.4580668767
H 3.1335345922 -0.6236462966 -1.4102773071
H 3.8337053410 -1.9783919653 -0.5154223327
S -0.6487068483 -1.8571713291 0.0824797490
O -0.4445370781 -2.2715080925 1.4809581897
O -1.1958478740 -2.7969426744 -0.8993986786
C -1.6338866817 -0.3655063022 0.0738252219
C -2.1593412911 0.0982695270 -1.1347328131
C -1.8737672770 0.3075409943 1.2710305655
C -2.9270238561 1.2588700477 -1.1346483581
H -1.9732552311 -0.4483745645 -2.0531350096
C -2.6489828726 1.4664358293 1.2510678093
H -1.4657742034 -0.0803338692 2.1983031707
C -3.1857530914 1.9591146142 0.0543132288
H -3.3372599497 1.6267736289 -2.0719924199
H -2.8423979746 1.9944587110 2.1815313789
C -4.0478548417 3.1988164217 0.0429626145
H -5.1137277391 2.9355029461 0.0322494226
H -3.8723229774 3.8182618202 0.9280096458
H -3.8575270431 3.8108545682 -0.8452827407
H 4.1458190034 3.2108518605 -1.9903389073
H 2.4982895151 3.0128077707 -0.1573907618
H 3.8336137179 1.8057741647 1.5321459856
H 2.3998298160 1.5924540313 -1.1743993385
H 1.8217687860 -1.9474465463 1.0677850735

11-H

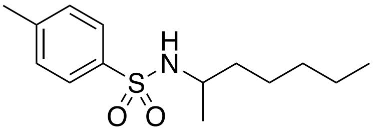

Name	E(B3LYP)	H(B3LYP)	E(RO-B2PLYP-D3)	H(RO-B2PLYP-D3)
Tosyl_NH_2_heptane_0001	-1150.685000	-1150.308726	-1150.238282	-1149.862008
Tosyl_NH_2_heptane_0042	-1150.683928	-1150.307602	-1150.237004	-1149.860678
Tosyl_NH_2_heptane_0034	-1150.685700	-1150.309556	-1150.236135	-1149.859991
Tosyl_NH_2_heptane_0016	-1150.684617	-1150.308369	-1150.236043	-1149.859795
Tosyl_NH_2_heptane_0018	-1150.684370	-1150.308139	-1150.235461	-1149.85923
Tosyl_NH_2_heptane_0037	-1150.683485	-1150.307295	-1150.235149	-1149.858959
Tosyl_NH_2_heptane_0096	-1150.684388	-1150.308166	-1150.235065	-1149.858843
Tosyl_NH_2_heptane_0083	-1150.684257	-1150.308057	-1150.235018	-1149.858818
Tosyl_NH_2_heptane_0069	-1150.684008	-1150.30788	-1150.234142	-1149.858014

41

C -2.1985687704 -1.8344673263 -0.3936386589
C -2.4712166156 -0.3328122100 -0.2536533971
C -3.9481406171 -0.0105579965 0.0078506921
C -4.2312972168 1.4915528261 0.1388540232
C -0.7228450140 -2.1998136099 -0.6306888169
N 0.0647039277 -1.8043194952 0.5614268788
H -2.1445296690 0.1797986018 -1.1707185245
H -1.8546443091 0.0719709453 0.5584514281
H -4.2752389372 -0.5211018132 0.9261887470
H -4.5646121584 -0.4249639883 -0.8041442440
H -3.6153384841 1.9056046807 0.9497620577
H -0.3426156857 -1.5916598678 -1.4599066123
C -5.7065972976 1.8078151612 0.4047544613
H -5.8756942224 2.8871559699 0.4926227226
H -6.0509663489 1.3400754201 1.3354125512
H -2.5532364442 -2.3629319224 0.5045071643
H -2.7835858776 -2.2384782964 -1.2315826307
S 1.7060866428 -1.4352564656 0.4083481386
O 2.2787213730 -2.0483541873 -0.7983038473
O 2.2670832280 -1.7072726139 1.7370854861
C 1.6918660568 0.3367324212 0.1336669994
C 1.3190507365 1.1912966534 1.1759620893
C 2.0855492443 0.8443330222 -1.1019299586
C 1.3318243368 2.5647974096 0.9631224523
H 1.0320390320 0.7813521067 2.1388278325
C 2.0918426906 2.2270868671 -1.2979163052
H 2.3893797114 0.1634011706 -1.8894994361
C 1.7165834401 3.1055365433 -0.2759278945
H 1.0433790977 3.2325922745 1.7715541575
H 2.3985529857 2.6265215209 -2.2613454945
C 1.7331103446 4.6010710731 -0.4846632394
H 2.4752403107 5.0830359233 0.1640453855
H 1.9774001141 4.8590167569 -1.5194492437
H 0.7602480939 5.0473675863 -0.2461134635
H -6.3443969080 1.4360025448 -0.4067131789
H -3.9068464092 2.0019306292 -0.7791973501
H -0.0296904248 -2.4434121100 1.3500106928
C -0.5420092287 -3.6824204432 -0.9799697098
H -1.1093623286 -3.9395748348 -1.8818687827
H -0.9080278025 -4.3198737518 -0.1640930221
H 0.5122935092 -3.9101096787 -1.1556564387
41

C -2.2957261229 -2.2352703402 0.2907944648
C -2.6646739810 -0.8244207541 0.7733955550
C -2.8146427325 0.2181864569 -0.3433164565
C -3.2449724131 1.5963119330 0.1764756390
C -0.8961893644 -2.3861538093 -0.3346524158
N 0.1210595568 -2.0110582087 0.6756285501
H -1.9087301076 -0.4829546019 1.4914983244
H -3.6123766524 -0.8884428140 1.3264096289
H -3.5501649889 -0.1378076451 -1.0810045361
H -1.8654293663 0.3294948903 -0.8850447904
H -4.2038116931 1.5019057687 0.7060309044
H -0.7898266607 -1.6652370232 -1.1519752881
C -3.3723182473 2.6462359310 -0.9319082754
H -3.6876824092 3.6166295452 -0.5309587478
H -4.1102578101 2.3427906754 -1.6848878598
H -2.3711310103 -2.9379074441 1.1339247024
H -3.0310239878 -2.5757719616 -0.4517232941
S 1.6305118946 -1.4345134416 0.1860279250
O 1.9589960794 -1.8829551892 -1.1744471139
O 2.5188581096 -1.7297085739 1.3162931139
C 1.3648670548 0.3374330292 0.0964665068
C 1.1434372743 1.0610955008 1.2719830267
C 1.4210166146 0.9791312557 -1.1389879892
C 0.9621613130 2.4378809002 1.1965437834
H 1.1252142733 0.5507740692 2.2294714028
C 1.2361487996 2.3620818482 -1.1960737728
H 1.6186582543 0.4006582416 -2.0348721831
C 1.0001277719 3.1103626995 -0.0369032340
H 0.7959002812 3.0048050586 2.1094733741
H 1.2844024372 2.8660424935 -2.1581693965
C 0.7784264921 4.6025976674 -0.1041659186
H 1.1315274239 5.0179958799 -1.0529883616
H -0.2881746000 4.8463738384 -0.0137163824
H 1.2983360288 5.1212505530 0.7088429122
H -2.4148346038 2.7917875694 -1.4473588374
H -2.5154000481 1.9405096970 0.9228046438
H 0.2820711309 -2.7212905433 1.3889341757
C -0.6701823835 -3.7967969806 -0.8933202063
H -0.7578029657 -4.5474482258 -0.0965611867
H 0.3225812122 -3.8780277329 -1.3427672729
H -1.4195902587 -4.0348314690 -1.6570364065
41

C -2.2172344552 -1.9421187646 -0.5104304961
C -2.5080114031 -0.4388805426 -0.4389848172
C -3.9946281489 -0.1239114597 -0.2228510464
C -4.3364305000 1.3753289833 -0.2431273693
C -0.7266535829 -2.2913756450 -0.6651836327
N -0.0045735830 -1.8538393998 0.5534787170
H -2.1694933733 0.0374528584 -1.3716811953
H -1.9063791788 -0.0012608165 0.3652617763
H -4.3210404991 -0.5556099066 0.7354371616
H -4.5852786682 -0.6300904696 -1.0001341305
H -4.0339648310 1.8004745621 -1.2110035693
H -0.3146451266 -1.6991225102 -1.4905695941

C -3.6994586321 2.1882696304 0.8906847678
H -4.0293372810 3.2331229381 0.8576100118
H -2.6053125464 2.1863658183 0.8307515188
H -2.6054444553 -2.4410465689 0.3906640093
H -2.7591853211 -2.3842675585 -1.3580465465
S 1.6221623888 -1.4111109407 0.4612732690
O 2.2686932114 -2.0059530752 -0.7167768671
O 2.1430266633 -1.6467619687 1.8130455816
C 1.5341243815 0.3563882917 0.1697333135
C 1.9695970165 0.8756559635 -1.0468087392
C 1.0573545541 1.1972800270 1.1803810339
C 1.9133076082 2.2553131012 -1.2563957931
H 2.3512030589 0.2060586351 -1.8098954344
C 1.0082392640 2.5682164235 0.9543614634
H 0.7371697091 0.7796969725 2.1294131133
C 1.4342892582 3.1198917020 -0.2664278698
H 2.2516080392 2.6634438442 -2.2055136520
H 0.6393929961 3.2253282921 1.7384730653
C 1.3896542398 4.6130368816 -0.4874548940
H 1.6031500976 4.8709668318 -1.5290850692
H 0.4069297819 5.0253778459 -0.2306694139
H 2.1282737488 5.1272072191 0.1406874119
H -3.9793004698 1.7831149481 1.8715307858
H -5.4279479193 1.4861457095 -0.1912551852
H -0.1030420126 -2.4887626609 1.3447546812
C -0.5018940435 -3.7783000277 -0.9652616477
H -0.8941950256 -4.4021658347 -0.1510825267
H 0.5641137992 -3.9886010724 -1.0845058558
H -1.0196505859 -4.0705781216 -1.8860325982
41

C -2.2909773264 -1.4329925117 -0.1445279866
C -2.3967462941 0.0849907008 0.0487149475
C -3.7716659791 0.5680336229 0.5387453753
C -4.9262815398 0.3525864932 -0.4502645735
C -0.8814592966 -1.9265932476 -0.5173227599
N 0.0371802089 -1.6487660079 0.6124342034
H -2.1540377911 0.5862576465 -0.9003291257
H -1.6293602568 0.3997304527 0.7655533029
H -3.7031663944 1.6409815513 0.7685614343
H -4.0132644656 0.0710562865 1.4905805465
H -4.6677720630 0.8192370512 -1.4119831808
H -0.5148046613 -1.3283117039 -1.3597759579
C -6.2564686687 0.9233033560 0.0527401049
H -6.5566792170 0.4542537287 0.9979609797
H -7.0629963189 0.7588177273 -0.6708966915
H -2.6132686217 -1.9474822250 0.7741384455
H -2.9771445121 -1.7649492298 -0.9340563537
S 1.6878334392 -1.4283916602 0.3290131101
O 2.0970276194 -2.0482100231 -0.9391436312
O 2.3286192447 -1.7991328064 1.5964003266
C 1.8203686821 0.3459941226 0.1062134343
C 1.6147188678 1.1937447792 1.1993360622
C 2.1614880692 0.8586708360 -1.1427756184
C 1.7413357420 2.5668381198 1.0247952986
H 1.3680477893 0.7780818303 2.1708953375
C 2.2840652296 2.2410417119 -1.2996991703
H 2.3360477449 0.1810136780 -1.9713547874
C 2.0757589452 3.1134944986 -0.2262833120
H 1.5837296631 3.2291272365 1.8728804905
H 2.5508390796 2.6443822290 -2.2733221275
C 2.2115539701 4.6080142353 -0.3949218713
H 2.4638648723 4.8741013332 -1.4256834979
H 1.2797622942 5.1239802749 -0.1327305668
H 2.9960213837 5.0110743086 0.2573170424
H -6.1824120490 2.0034196899 0.2299478560
H -5.0497312624 -0.7192111634 -0.6552341137
H -0.0519951184 -2.3036870609 1.3885146914
C -0.8733597765 -3.4065735838 -0.9202266955
H -1.2313379075 -4.0348669637 -0.0937335674
H 0.1362754563 -3.7258834785 -1.1903280311
H -1.5343283973 -3.5780399026 -1.7776382866
41

C -2.2461984599 -1.9215408311 -0.2466725006
C -2.5508279086 -0.4352434661 -0.0279470260
C -4.0231887444 -0.1753213948 0.3209754410
C -4.3612079420 1.2962746423 0.6128531972
C -0.7772264377 -2.2334333023 -0.5814553018
N 0.0676275849 -1.8561213505 0.5767491593
H -2.2765609766 0.1201376167 -0.9354427564
H -1.9086533258 -0.0492662100 0.7738246866
H -4.2889759167 -0.7783482062 1.2012011959
H -4.6633434058 -0.5366895729 -0.4981887065
H -5.3951698812 1.3515165636 0.9792461476
H -0.4638293706 -1.5849840561 -1.4081294459
C -4.2108949593 2.2341616646 -0.5910283528
H -4.8324026293 1.8996195337 -1.4313781872
H -3.1748228489 2.2840322283 -0.9441117315
H -2.5350610181 -2.4966763037 0.6464127682
H -2.8667983028 -2.3078109856 -1.0672861718
S 1.6846102171 -1.4280074043 0.3420852713
O 2.2070843812 -1.9872472152 -0.9125439576
O 2.3294745144 -1.7178292546 1.6282751895
C 1.5964676424 0.3493941813 0.1208019941
C 1.2569870851 1.1618741879 1.2071516993
C 1.8995081040 0.9037206585 -1.1203468958
C 1.2111796756 2.5402884766 1.0338239488
H 1.0403298109 0.7161589291 2.1725191987
C 1.8476250222 2.2905628958 -1.2763129280
H 2.1788001938 0.2553751640 -1.9436950736
C 1.5042096349 3.1274573958 -0.2091629913
H 0.9481461120 3.1754757158 1.8764646001
H 2.0830467288 2.7263364133 -2.2440529255
C 1.4616973644 4.6279012298 -0.3745510060
H 2.2515711626 5.1128190137 0.2129236900
H 1.5978846388 4.9203042871 -1.4200177384
H 0.5060745392 5.0402626148 -0.0297075309
H -4.5201516658 3.2543005484 -0.3357790007
H -3.7274397453 1.6546570040 1.4366957108
H 0.0392404028 -2.5248845455 1.3456151119
C -0.5751592071 -3.6965495967 -0.9953162194
H -1.1878352726 -3.9391481332 -1.8712285854
H -0.8733573862 -4.3732795466 -0.1834921666
H 0.4728438641 -3.8854534067 -1.2408504457
41

C -2.2606237184 -1.4631975187 -0.6047020651
C -2.3855195462 0.0654748192 -0.5821680889
C -3.8333994614 0.5837244956 -0.5690311434
C -4.6196772136 0.2751301013 0.7134737639
C -0.8129831720 -1.9799104672 -0.6768275116
N -0.0994195680 -1.5767675784 0.5585355162
H -1.8747079259 0.4700452041 -1.4676376671
H -1.8442040047 0.4596643032 0.2869383748
H -4.3774659479 0.1752741594 -1.4342331635
H -3.8145807117 1.6735191496 -0.7127307081
H -4.6973412342 -0.8111139517 0.8547875741
H -0.3019741698 -1.4689842790 -1.5014009035
C -6.0269348736 0.8815261467 0.7095682528
H -6.5662219170 0.6497064900 1.6351060347
H -6.6220562001 0.4964043550 -0.1277428450
H -2.7430048911 -1.9021401690 0.2801867285
H -2.8026724092 -1.8614947098 -1.4741685916
S 1.5771240001 -1.3718096886 0.5403993438
O 2.1923510455 -2.1189756951 -0.5655269727
O 1.9880184491 -1.5989326472 1.9310179729
C 1.7612946293 0.3700394307 0.1558211653
C 2.3060061854 0.7502840269 -1.0682277003
C 1.3866699626 1.3270713341 1.1040509142
C 2.4639731739 2.1090986649 -1.3491496175
H 2.6070277217 -0.0098892325 -1.7808076310
C 1.5518898791 2.6750547544 0.8076586719
H 0.9797042856 1.0146897105 2.0602541491
C 2.0916384056 3.0887261558 -0.4223934884
H 2.8882375972 2.4092064562 -2.3040239910
H 1.2621222455 3.4222917012 1.5427576696
C 2.2766308240 4.5575134854 -0.7205968790
H 3.0464247580 4.9980181368 -0.0743255709
H 2.5811548365 4.7208943924 -1.7586973754

H 1.3514467513 5.1195458371 -0.5467986751
H -5.9875574393 1.9734367504 0.6109586335
H -4.0586472373 0.6559375605 1.5791426100
H -0.3265089048 -2.1466243687 1.3726956075
C -0.7488676452 -3.4936291359 -0.9160944479
H -1.2464824290 -4.0360702434 -0.1011627512
H 0.2893690109 -3.8292876172 -0.9766742285
H -1.2565764182 -3.7597542128 -1.8503647415
41

C -2.0652865713 -2.2782549343 -0.4961625498
C -3.0482928849 -1.2147970909 0.0097116534
C -4.4140273478 -1.8011452124 0.3918276533
C -5.4042082356 -0.7487782968 0.9072004878
C -0.6980732148 -1.7462848021 -0.9652141241
N 0.0403604379 -1.0696471442 0.1254410710
H -3.1795985557 -0.4444686005 -0.7618498021
H -2.6229133907 -0.6973497274 0.8797723507
H -4.2777494054 -2.5768346294 1.1608705267
H -4.8503277561 -2.3129741949 -0.4793645948
H -4.9683521853 -0.2383639699 1.7773033665
H -0.8632823091 -0.9840300212 -1.7325249597
C -6.7667805480 -1.3368814672 1.2875957827
H -7.2433031568 -1.8239461526 0.4276232293
H -7.4501389416 -0.5608199863 1.6508777370
H -1.8992556029 -3.0346976442 0.2880678940
H -2.5171672327 -2.8196166857 -1.3392717165
S 0.0787874774 0.6060710425 0.2793148996
O -0.2525860328 0.9033979976 1.6791522823
O -0.6692552348 1.1885594717 -0.8411194026
C 1.8048648210 1.0363716617 0.0407576908
C 2.2681999275 1.3399625864 -1.2400137196
C 2.6667227201 1.0608031073 1.1380175978
C 3.6133749936 1.6567869697 -1.4183096257
H 1.5794204656 1.3417003875 -2.0781151855
C 4.0084458600 1.3801854039 0.9408854173
H 2.2850354828 0.8471877745 2.1308219766
C 4.5036768864 1.6820499467 -0.3356410832
H 3.9766267774 1.8948865859 -2.4151958812
H 4.6817222218 1.4009791893 1.7945158764
C 5.9524743899 2.0596783010 -0.5337279709
H 6.0961423611 3.1423444962 -0.4194477430
H 6.5992824609 1.5671673874 0.1997524398
H 6.3043795489 1.7890588319 -1.5345832203
H -6.6672166815 -2.0897619646 2.0794844425
H -5.5401013874 0.0254802968 0.1391608081
H -0.0751878487 -1.4847042881 1.0463795845
C 0.1798077440 -2.8623032621 -1.5455249172
H 1.1383068818 -2.4602571344 -1.8872395226
H -0.3198196828 -3.3452086106 -2.3933065998
H 0.3852528165 -3.6331337318 -0.7925833918
41

C -1.9889333375 -2.2046539298 -1.3851415142
C -2.9879117031 -2.3256749756 -0.2250421427
C -3.8561907361 -1.0813713389 0.0090311501
C -4.9367819244 -1.3005652718 1.0764085276
C -0.9039231734 -1.1213661135 -1.2248170950
N -0.1355527721 -1.3790435001 0.0081229388
H -2.4418468941 -2.5643802821 0.6959987981
H -3.6411510322 -3.1855421500 -0.4312614334
H -4.3375109118 -0.7857572544 -0.9366669697
H -3.2226609844 -0.2398824519 0.3173996774
H -5.5803694121 -2.1404390078 0.7765333872
H -1.3932527378 -0.1560450952 -1.0671362438
C -5.7985056665 -0.0585339928 1.3249253070
H -6.3176982424 0.2540386939 0.4100469006
H -5.1852082911 0.7856086952 1.6624555065
H -1.4894747825 -3.1734576577 -1.5372818108
H -2.5303155049 -1.9937247990 -2.3183976769
S 0.3517063136 -0.1808065454 1.0644256037
O 0.9373240821 -0.8960756472 2.2004841787
O -0.7862724628 0.7331685911 1.2021161783
C 1.6816208686 0.7619252138 0.3015641667
C 2.9976773167 0.3056302259 0.4092039638
C 1.3890653357 1.9319493070 -0.4016067724
C 4.0202363182 1.0228281888 -0.2069883003
H 3.2151510223 -0.5872991792 0.9863858499
C 2.4251689595 2.6370273946 -1.0127225520
H 0.3661094619 2.2898045335 -0.4499317615
C 3.7529945123 2.1964190226 -0.9275552349
H 5.0452108471 0.6698991360 -0.1210086293
H 2.1986068695 3.5500134635 -1.5581414587
C 4.8737134080 2.9840413321 -1.5632751790
H 5.3189449303 3.6841925588 -0.8440630040
H 5.6762707501 2.3272951855 -1.9151526720
H 4.5176314202 3.5735741138 -2.4141621374
H -6.5594062640 -0.2457418637 2.0914655402
H -4.4556920547 -1.6048111295 2.0163888172
H 0.5228529475 -2.1529340208 -0.0177789883
C -0.0032378286 -1.0397293657 -2.4653825148
H -0.5991288883 -0.8290952127 -3.3607016319
H 0.5177905877 -1.9916369279 -2.6320145793
H 0.7491765472 -0.2519836941 -2.3630369088
41

C -2.0605652025 -2.2934008650 -1.3222903654
C -2.8241395847 -2.4301377670 0.0050974124
C -3.7969372229 -1.2818253876 0.3083724940
C -4.6271533428 -1.5222998619 1.5763731460
C -1.0625629452 -1.1239783630 -1.4190752079
N 0.0204486477 -1.2828486103 -0.4221753525
H -2.1134098725 -2.5302013113 0.8376064734
H -3.3843344080 -3.3753285005 -0.0251762879
H -4.4752625793 -1.1452715948 -0.5482667674
H -3.2399786320 -0.3437999571 0.4237975770
H -5.1882914158 -2.4624787260 1.4727677246
H -1.5819174885 -0.1925948490 -1.1743933996
C -5.5979319971 -0.3779518302 1.8845829039
H -5.0600812521 0.5666094652 2.0297395879
H -6.1748074224 -0.5768484781 2.7951365443
H -1.5141103101 -3.2268496080 -1.5293419139
H -2.7816245653 -2.1828600794 -2.1436616216
S 0.2985066895 -0.1618249831 0.7960021479
O 0.7774421966 -0.9508412453 1.9372785540
O -0.8562704683 0.7399629167 0.9053948794
C 1.6694485749 0.8296201859 0.1981103097
C 1.4208031541 2.0652386606 -0.3980060757
C 2.9741852950 0.3504469230 0.3369080896
C 2.4944957901 2.8194603102 -0.8719797013
H 0.4030436316 2.4325197534 -0.4739651182
C 4.0329707767 1.1174258067 -0.1397722236
H 3.1526963272 -0.6027549931 0.8234996585
C 3.8124000890 2.3612853571 -0.7518981018
H 2.3034140643 3.7836343530 -1.3367413799
H 5.0498429338 0.7475212555 -0.0315075782
C 4.9734511208 3.1951243924 -1.2391156008
H 5.4510513448 3.7293505385 -0.4072454584
H 5.7440566949 2.5735320959 -1.7080875780
H 4.6509794951 3.9449791098 -1.9681988795
H -6.3114215981 -0.2305376408 1.0640000940
H -3.9478543144 -1.6635270563 2.4285021186
H 0.1500711121 -2.2164963854 -0.0437846900
C -0.4627088371 -1.0051616691 -2.8260324601
H 0.0676693783 -1.9237754208 -3.1036437876
H 0.2512395168 -0.1768838096 -2.8724629106
H -1.2493720888 -0.8267194803 -3.5686618327
41

C -2.0136707610 -1.2860789215 1.3325697439
C -3.4698339801 -1.1135316291 0.8682038289
C -3.6873767824 0.0024714507 -0.1635573316
C -5.1659626698 0.2242361830 -0.5092022297
C -1.0012120775 -1.6720153794 0.2368090911
N 0.3419523301 -1.6825130528 0.8646743140
H -4.0805504291 -0.8970954192 1.7557911007
H -3.8540600317 -2.0615669010 0.4678557817
H -3.1377617083 -0.2247442783 -1.0882958174
H -3.2598058772 0.9406691883 0.2210247696
H -5.5943530210 -0.7156061226 -0.8857093896

H -0.9717579055 -0.8749051173 -0.5140362154
C -5.3853250755 1.3346893817 -1.5415358148
H -4.9975339251 2.2948803683 -1.1794450049
H -6.4498563983 1.4686441415 -1.7648358939
H -1.6644232681 -0.3551443262 1.7965663227
H -1.9802275700 -2.0543592413 2.1192680510
S 1.7135082284 -1.3845439045 -0.0730629318
O 1.4931276869 -1.7322042352 -1.4844117866
O 2.8135764222 -1.9899199323 0.6867419400
C 1.8479007547 0.4022796894 0.0055348721
C 2.0683477242 1.0247448841 1.2383240103
C 1.7813642209 1.1462375511 -1.1698773968
C 2.2109041179 2.4065111351 1.2835562702
H 2.1252542680 0.4311421882 2.1448387570
C 1.9264230155 2.5337343495 -1.1054829194
H 1.6211864282 0.6401664348 -2.1156187079
C 2.1429316657 3.1836371444 0.1142237403
H 2.3804342383 2.8943231571 2.2406480731
H 1.8726183003 3.1174508183 -2.0210175502
C 2.3134500323 4.6825650057 0.1820675946
H 1.6353477949 5.1283635116 0.9193890371
H 3.3345616604 4.9508633700 0.4813268160
H 2.1162530199 5.1530693549 -0.7858184655
H -4.8735050722 1.1064935767 -2.4845968029
H -5.7196152524 0.4630601521 0.4099872614
H 0.5314894231 -2.5080749242 1.4317146741
C -1.3245097752 -2.9996019869 -0.4594834032
H -2.2934524199 -2.9555990976 -0.9682447309
H -1.3692344422 -3.8182049215 0.2714808150
H -0.5582504711 -3.2344801451 -1.2021801126

11-Cl

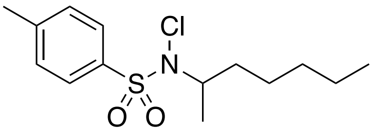

Name	E(B3LYP)	H(B3LYP)	E(RO-B2PLYP-D3)	H(RO-B2PLYP-D3)
Tosyl_NCl_2_heptane_0016	-1610.234968	-1609.867873	-1609.681383	-1609.314288
Tosyl_NCl_2_heptane_0055	-1610.235691	-1609.868733	-1609.680824	-1609.313866
Tosyl_NCl_2_heptane_0028	-1610.23371	-1609.866553	-1609.680925	-1609.313768
Tosyl_NCl_2_heptane_0041	-1610.233673	-1609.866493	-1609.680767	-1609.313587
Tosyl_NCl_2_heptane_0057	-1610.233498	-1609.866338	-1609.680204	-1609.313044
Tosyl_NCl_2_heptane_0094	-1610.234351	-1609.867313	-1609.680047	-1609.313009
Tosyl_NCl_2_heptane_0022	-1610.232445	-1609.865469	-1609.679539	-1609.312563
Tosyl_NCl_2_heptane_0030	-1610.232379	-1609.865244	-1609.679694	-1609.312559
Tosyl_NCl_2_heptane_0074	-1610.233025	-1609.866005	-1609.678783	-1609.311763

41

C -2.9304937633 -0.9972843251 0.1741737231
C -3.0377559172 0.4515728162 -0.3150838580
C -4.3413581716 1.1328715496 0.1208929994
C -4.4602067948 2.5851899652 -0.3589841990
C -1.6730017086 -1.7466649679 -0.2929315684
N -0.4552264320 -0.9826204692 0.1322799461
H -2.9654247925 0.4703252826 -1.4126235163
H -2.1802093299 1.0249399880 0.0579302934
H -4.4160038988 1.1076934158 1.2183585908
H -5.2005682046 0.5567177925 -0.2544827220
H -3.6026947515 3.1609403367 0.0173551302
H -1.6141147304 -1.6723978643 -1.3843132050
C -5.7624719321 3.2621833454 0.0803005626
H -5.8169448490 4.2966048799 -0.2778613388
H -5.8490656735 3.2833197916 1.1737090382
H -2.9865854665 -1.0293181871 1.2702548609
H -3.7920699513 -1.5733483256 -0.1911635981
S 1.0167496865 -1.4013068576 -0.7286755585
O 0.5958212076 -1.3469648548 -2.1330429527
O 1.6713163023 -2.5971068301 -0.1899124003
C 2.0331963815 0.0190326459 -0.3559144426
C 1.7196240404 1.2533433348 -0.9312905796
C 3.1509504807 -0.1369989258 0.4612059944
C 2.5392183769 2.3450760677 -0.6668511569
H 0.8511306464 1.3506572751 -1.5738346964
C 3.9616585213 0.9698593267 0.7114644945
H 3.3751627533 -1.1080188241 0.8886189712
C 3.6716577928 2.2218605511 0.1550828932
H 2.3001849051 3.3099448358 -1.1073008839
H 4.8338786252 0.8567156241 1.3501019716
C 4.5652650130 3.4118032573 0.4099436941
H 5.2246154176 3.2425876626 1.2665731120
H 5.2008899017 3.6188844236 -0.4608520187
H 3.9795093285 4.3170938084 0.6040861737
H -6.6380056510 2.7284101917 -0.3096222676
Cl -0.1842298471 -1.0382361723 1.8896839321
C -1.6817304057 -3.2249876201 0.1110911387
H -4.3857089268 2.6110138492 -1.4555142198
H -1.7460908837 -3.3385540475 1.1978924098
H -0.7826278929 -3.7418825893 -0.2349634167
H -2.5540517140 -3.7171593274 -0.3328101500
41

C -2.9311691308 -1.2019512079 1.0392377068
C -3.1364943798 0.3202622413 1.0171997909
C -3.5797684904 0.8896389824 -0.3379794042
C -3.8816782404 2.3934664002 -0.2877280886
C -1.8341970769 -1.7429516108 0.1071996199
N -0.5490312180 -1.0268644562 0.3939571720
H -2.2100880089 0.8142924775 1.3355477168
H -3.8945402417 0.5690110291 1.7731744304
H -4.4767380255 0.3535166421 -0.6845729730
H -2.8032621930 0.7089546175 -1.0939828164
H -4.6636523337 2.5816726108 0.4618243993
H -2.0499588054 -1.4188932734 -0.9153098323
C -4.3197711875 2.9673266599 -1.6390272575
H -4.5255285033 4.0417736030 -1.5705568919
H -5.2316092756 2.4763590462 -2.0008712435
H -2.7178338459 -1.5290032392 2.0648636060
H -3.8644980775 -1.7033046929 0.7469377279
S 0.6381946110 -1.1030037837 -0.8961401596
O -0.1386791408 -0.6842938428 -2.0690541570
O 1.3892747532 -2.3618956376 -0.9081728443
C 1.7441875616 0.2131375492 -0.4122845837
C 1.3186819626 1.5395734752 -0.5227569031
C 3.0338210757 -0.1014489958 0.0117079055
C 2.2026008261 2.5588411824 -0.1857811571
H 0.3173388843 1.7639079638 -0.8741526712
C 3.9056904184 0.9355753248 0.3419051835
H 3.3430821373 -1.1389115958 0.0737293839
C 3.5058891540 2.2749694466 0.2543657989
H 1.8800633197 3.5936234383 -0.2708638564
H 4.9140878832 0.6981211701 0.6707705555
C 4.4449814314 3.3931629899 0.6378609114
H 5.4837955885 3.0512246697 0.6718027326
H 4.3834175381 4.2273278012 -0.0696489728
H 4.1956048337 3.7916903665 1.6299590102
H -3.5441005549 2.8235088555 -2.4011874594
Cl 0.1784190783 -1.5073334189 1.9443955515
C -1.7438972197 -3.2726076042 0.1328586087
H -2.9888236037 2.9291078478 0.0658932630
H -1.5415406316 -3.6419974953 1.1431702502
H -0.9568705417 -3.6395718564 -0.5314422570
H -2.6987203691 -3.6966857945 -0.1969645892
41

C -3.0006916477 -1.1199254764 0.1557784219
C -3.1561803077 0.3250482511 -0.3325786013
C -4.4904518822 0.9511417298 0.0976341511
C -4.7190325283 2.3825549674 -0.4160256090
C -1.7143982924 -1.8236818779 -0.3039378116
N -0.5270098511 -1.0159545198 0.1266296864
H -3.0823788402 0.3472961926 -1.4302802796
H -2.3174451949 0.9207743972 0.0440482732
H -4.5557308545 0.9475161751 1.1961251901
H -5.3133491668 0.3139080903 -0.2572609511
H -4.6520609981 2.3869714161 -1.5135137434
H -1.6525300931 -1.7483411065 -1.3950730471

C -3.7531341429 3.4257054393 0.1586980077
H -3.8029348332 3.4470103614 1.2546906160
H -3.9978522289 4.4304978242 -0.2045606604
H -3.0623437577 -1.1550093719 1.2515027376
H -3.8389305137 -1.7261328756 -0.2151381449
S 0.9641277539 -1.3843129877 -0.7245427149
O 0.5497374274 -1.3496153110 -2.1314686728
O 1.6579287121 -2.5540433904 -0.1778049070
C 1.9266778571 0.0730022826 -0.3511931657
C 1.5760549597 1.2917358181 -0.9382654600
C 3.0403102124 -0.0381103054 0.4788230001
C 2.3534455958 2.4135759161 -0.6723844477
H 0.7121076306 1.3538525276 -1.5912244433
C 3.8084092754 1.0984476588 0.7304037583
H 3.2943385831 -0.9978457164 0.9149543858
C 3.4802767182 2.3358760297 0.1626952779
H 2.0853869951 3.3665141594 -1.1220039210
H 4.6770399096 1.0203989518 1.3791087894
C 4.3280234004 3.5584959001 0.4195356809
H 3.7082181461 4.4434720430 0.6012186539
H 4.9832572862 3.4182485208 1.2845137039
H 4.9656530352 3.7827267006 -0.4455234904
H -2.7139327850 3.2193530471 -0.1206224232
Cl -0.2648650124 -1.0581461132 1.8858044273
C -1.6719241221 -3.3009418324 0.1017981689
H -5.7481675912 2.6805225318 -0.1742582024
H -1.7362858374 -3.4153932722 1.1885035761
H -0.7536307429 -3.7858250740 -0.2401564370
H -2.5243966386 -3.8244816366 -0.3447609551
41

C -3.0182148116 -1.2291822329 0.8756985982
C -3.2753804366 0.2764799366 0.7099111220
C -3.6933113831 0.6952847314 -0.7084072031
C -4.1130297148 2.1700470842 -0.8310538333
C -1.8538041711 -1.8020763437 0.0507004372
N -0.6073718110 -1.0282312622 0.3589817967
H -2.3785271495 0.8256728191 1.0179449213
H -4.0714030193 0.5624183542 1.4122658068
H -4.5339116256 0.0630268390 -1.0305773892
H -2.8741143143 0.5008797767 -1.4145515358
H -4.5338246738 2.3329097301 -1.8322710887
H -2.0161758302 -1.5587234806 -1.0035998628
C -2.9756324063 3.1730868867 -0.6035794691
H -2.1555956236 3.0015238333 -1.3131547075
H -2.5620598731 3.1004725618 0.4085559237
H -2.8480386916 -1.4594758204 1.9351241483
H -3.9163854885 -1.7902036522 0.5815068624
S 0.6511858781 -1.1494293930 -0.8575943517
O -0.0684059792 -0.8184564736 -2.0934109455
O 1.4282785888 -2.3885521364 -0.7565091314
C 1.7007033635 0.2180703906 -0.3908000647
C 1.2374259222 1.5260031804 -0.5569416645
C 2.9858853834 -0.0412989891 0.0811270229
C 2.0792177207 2.5832070723 -0.2294087859
H 0.2366217675 1.7078391224 -0.9337075228
C 3.8156477865 1.0332254662 0.4000354230
H 3.3219190584 -1.0660008892 0.1946871085
C 3.3793569780 2.3555990418 0.2510985950
H 1.7243543017 3.6037323851 -0.3504273994
H 4.8184528479 0.8387454330 0.7715026238
C 4.2888139208 3.5158173680 0.5769457827
H 5.1388971471 3.1999586285 1.1891502433
H 4.6907680540 3.9686030343 -0.3387985882
H 3.7530708747 4.3036827802 1.1178663897
H -3.3236767485 4.2030313699 -0.7437285542
Cl 0.0457364037 -1.3852560375 1.9747070379
C -1.7243159627 -3.3224569393 0.1934192027
H -4.9277023820 2.3747338843 -0.1214465521
H -1.5796639309 -3.6127801518 1.2388357871
H -0.8851657251 -3.7101034724 -0.3904345182
H -2.6433676107 -3.7987343336 -0.1654543763
41

C -2.8967571800 -0.7844420527 1.1668686460
C -2.9259350678 0.7513569027 1.2391768737
C -3.2791410267 1.4818514775 -0.0685679090
C -4.6793203477 1.1792829828 -0.6213128224
C -1.8997375871 -1.3860714769 0.1632276417
N -0.5351596508 -0.8256581338 0.4289907051
H -1.9487551043 1.1045995614 1.5886416094
H -3.6523764198 1.0370474723 2.0130567954
H -2.5281633993 1.2580083010 -0.8390875715
H -3.2028047277 2.5632121652 0.1156095664
H -4.7643419778 0.1120035220 -0.8677182913
H -2.1175010415 -0.9862613274 -0.8318852174
C -5.0193053397 2.0066977892 -1.8655733658
H -4.9879269757 3.0812803822 -1.6467194665
H -6.0217535348 1.7723494935 -2.2416653413
H -2.6826263540 -1.1873274957 2.1649658420
H -3.8871326509 -1.1706481014 0.8932068076
S 0.5888996362 -0.9583152259 -0.9117871077
O -0.1834324160 -0.4053295605 -2.0309535980
O 1.2047182890 -2.2848226374 -1.0128344921
C 1.8420593889 0.2131148707 -0.4149395265
C 1.5489239336 1.5793140864 -0.4367753153
C 3.1100120672 -0.2508939450 -0.0704801198
C 2.5455585359 2.4855001682 -0.0915914421
H 0.5610235129 1.9211019704 -0.7262328758
C 4.0962580056 0.6746662526 0.2693167026
H 3.3143803446 -1.3158010031 -0.0759855694
C 3.8312248952 2.0498042817 0.2694292292
H 2.3262918075 3.5502673174 -0.1076680529
H 5.0885133888 0.3204940425 0.5364985301
C 4.8948655716 3.0466465127 0.6622523143
H 4.8974535842 3.9133168562 -0.0079755841
H 4.7221365668 3.4245576031 1.6783933036
H 5.8925900678 2.5979634457 0.6424731476
H -4.3055251044 1.8137901541 -2.6757305966
Cl 0.1932430362 -1.4596525015 1.9224625622
C -1.9722539828 -2.9159440239 0.1078324625
H -5.4266863116 1.3704326716 0.1626004116
H -2.9794338774 -3.2196674738 -0.1982738108
H -1.7693669594 -3.3590663873 1.0879091927
H -1.2565309807 -3.3261620490 -0.6097342019
41

C -2.9829386676 -1.0861570554 0.8023235292
C -3.1580549434 0.4318026548 0.6390397456
C -3.4712385487 0.8871168558 -0.7943327929
C -3.7030365807 2.4014716760 -0.9389730752
C -1.8231545598 -1.7184196752 0.0149120128
N -0.5541477380 -0.9894176994 0.3385697671
H -2.2510602545 0.9397844847 0.9905908889
H -3.9681368196 0.7442517207 1.3103999436
H -4.3599622516 0.3511020104 -1.1621433070
H -2.6440794129 0.6009139503 -1.4567640033
H -2.8248143478 2.9386375102 -0.5519950710
H -1.9511332120 -1.4882584297 -1.0469164713
C -4.9666137440 2.9248197509 -0.2453380714
H -5.8613140803 2.4089652672 -0.6163648459
H -5.1013894884 3.9967567569 -0.4304566427
H -2.8619172094 -1.3283933239 1.8658602941
H -3.8981992971 -1.5991803112 0.4751020230
S 0.7322477833 -1.1957306157 -0.8367809141
O 0.0586790791 -0.8867416659 -2.1038148870
O 1.4646311504 -2.4542663710 -0.6676510152
C 1.8134527402 0.1543243356 -0.3920671904
C 1.4135957071 1.4669735363 -0.6571224511
C 3.0601833042 -0.1253927388 0.1637143205
C 2.2793715818 2.5089492722 -0.3440112697
H 0.4439415485 1.6628100085 -1.1020433653
C 3.9149025421 0.9339805410 0.4674899128
H 3.3487129341 -1.1536982273 0.3512316554
C 3.5414978150 2.2608495365 0.2206485753
H 1.9744228537 3.5333208517 -0.5434992780
H 4.8880707554 0.7238517747 0.9035695749
C 4.4779073959 3.4035433660 0.5318017482
H 3.9524795393 4.2257189751 1.0304214235
H 5.3005354247 3.0838216410 1.1784106842

H 4.9180325823 3.8120158606 -0.3871185656
H -4.9287899135 2.7828970919 0.8405177977
Cl 0.0373920372 -1.3276822295 1.9814613042
C -1.7587924101 -3.2399710801 0.1865058905
H -3.7597710330 2.6449540634 -2.0083685390
H -0.9290752064 -3.6730849064 -0.3786305821
H -2.6920236316 -3.6845531831 -0.1764997999
H -1.6400298957 -3.5167719076 1.2388392322
41

C -2.6786583933 -1.3962836143 0.5862544964
C -3.1747091960 -0.3587803103 -0.4281585718
C -4.6671950070 -0.0428581993 -0.2588092841
C -5.1850508666 0.9990761381 -1.2585926401
C -1.2325710303 -1.8894021124 0.4099197635
N -0.1476341860 -0.8712477505 0.5858709468
H -2.9931576210 -0.7280775129 -1.4476616978
H -2.5973024146 0.5674376960 -0.3324350386
H -4.8494027618 0.3170676240 0.7651003897
H -5.2537676633 -0.9684545826 -0.3633745609
H -4.5999543011 1.9229790673 -1.1522856922
H -1.0858269009 -2.2092842610 -0.6265929135
C -6.6747007437 1.3126270673 -1.0858911749
H -6.8836470773 1.7066004369 -0.0834407740
H -7.2883958069 0.4131665480 -1.2210896817
H -2.8060120919 -1.0143208312 1.6077137976
H -3.3139326072 -2.2909640736 0.5193583533
S 0.3219599627 0.1288329884 -0.7595664640
O -0.3268914230 1.4424154743 -0.7554434063
O 0.1925272393 -0.7746251680 -1.9084635747
C 2.0543280616 0.3756026747 -0.3910064980
C 2.4982969342 1.6373373986 -0.0017077462
C 2.9450551115 -0.6898483339 -0.5481157066
C 3.8582091471 1.8270997598 0.2457360785
H 1.7894120792 2.4517761705 0.0972079384
C 4.2961883299 -0.4803386186 -0.2968872074
H 2.5827619679 -1.6603379878 -0.8702066324
C 4.7741160250 0.7775157857 0.1073221894
H 4.2112576768 2.8095844516 0.5482463749
H 4.9952159292 -1.3039215918 -0.4205128835
C 6.2419501738 0.9811541903 0.3963391486
H 6.5243074243 0.5105253474 1.3470344536
H 6.8683989196 0.5332415212 -0.3832196870
H 6.4937884108 2.0435921256 0.4659056237
H -7.0137332194 2.0588561253 -1.8135967260
Cl -0.2513206677 0.0535009418 2.0858993979
C -0.9135973100 -3.0674924402 1.3381100327
H -5.0018339261 0.6407108330 -2.2814000703
H 0.1127131637 -3.4132501341 1.1855510583
H -1.5970877614 -3.8979799901 1.1307275267
H -1.0320133490 -2.7878307854 2.3903757820
41

C -3.1401526257 -1.4524940968 0.0805501981
C -3.4991335219 -0.0874119630 -0.5387157210
C -2.9664204777 1.1455094850 0.2060645680
C -3.4097068872 2.4672469756 -0.4346053413
C -1.7786477811 -2.0498573548 -0.3150611975
N -0.6677614952 -1.1399293024 0.1141428849
H -4.5949596306 -0.0140048300 -0.5837503772
H -3.1550333801 -0.0625239354 -1.5831000500
H -1.8721727650 1.1089458632 0.2490771313
H -3.3164264207 1.1145971239 1.2487739382
H -3.0718775505 2.4942798068 -1.4808913092
H -1.6948902906 -2.0070898446 -1.4064878601
C -2.8787589528 3.7006252101 0.3034387028
H -1.7818730316 3.7046731682 0.3273829952
H -3.2297521624 3.7220446159 1.3425508115
H -3.2177440871 -1.4067856174 1.1748521912
H -3.8846823371 -2.1924658227 -0.2415498298
S 0.8585447545 -1.3951128084 -0.7216432698
O 0.4566755992 -1.4044111195 -2.1326588492
O 1.6388326150 -2.5005262394 -0.1577549370
C 1.6959442257 0.1387336340 -0.3527228443
C 1.2783105566 1.3128540173 -0.9851817314
C 2.7837932900 0.1319184013 0.5179443863
C 1.9583477118 2.4968941522 -0.7208497963
H 0.4442165554 1.2915922523 -1.6783393725
C 3.4539271181 1.3292996185 0.7671889283
H 3.0974922802 -0.7963821332 0.9823543262
C 3.0517174893 2.5258231434 0.1605283326
H 1.6420690576 3.4140425538 -1.2116146251
H 4.3050957528 1.3314620693 1.4431406891
C 3.7639924014 3.8240668164 0.4546032459
H 4.7482360810 3.6501268090 0.8998405971
H 3.9012028819 4.4197445097 -0.4544949774
H 3.1864697241 4.4371704886 1.1587757314
H -3.2064819343 4.6293499584 -0.1779182807
Cl -0.4169369585 -1.1440298466 1.8753187291
C -1.6249910186 -3.5061401299 0.1408499951
H -4.5080719266 2.5066140618 -0.4711428484
H -0.6471928726 -3.9108715538 -0.1337993557
H -2.3975517140 -4.1196715910 -0.3352014308
H -1.7414555016 -3.5972701502 1.2254048508
41

C -2.6377358496 -1.0766028191 0.8430920949
C -4.1021470628 -1.3074439015 0.4217479886
C -4.5869109928 -0.5184823418 -0.8082756461
C -4.4920974710 1.0083915225 -0.6754937859
C -1.5976193190 -1.4088867706 -0.2422973716
N -0.1900693461 -1.0153093021 0.0984003367
H -4.7351488030 -1.0423400173 1.2797920201
H -4.2721974998 -2.3782732314 0.2478640063
H -5.6345104083 -0.7946785490 -0.9957904699
H -4.0347746575 -0.8364750128 -1.7051954096
H -4.9913334400 1.3208300860 0.2532834411
H -1.7857601998 -0.7932710348 -1.1272144218
C -5.1183497997 1.7449277992 -1.8644552459
H -4.6207293919 1.4739082950 -2.8038555765
H -5.0356760333 2.8313689566 -1.7482357801
H -2.5074100475 -0.0337956220 1.1507552879
H -2.4274945864 -1.6861061941 1.7305257702
S 0.1068775980 0.7137470546 0.1577966367
O -0.2304289784 1.3121300749 1.4525393087
O -0.5297896777 1.1946225973 -1.0752427413
C 1.8822910163 0.7495062215 -0.0362588803
C 2.6760289215 1.1630679380 1.0315056056
C 2.4431407996 0.4090423422 -1.2701486407
C 4.0584670997 1.2265868566 0.8576458317
H 2.2140128694 1.4284163990 1.9759232083
C 3.8235092003 0.4769954125 -1.4220270272
H 1.8066008247 0.0991564060 -2.0919700683
C 4.6520816590 0.8870332588 -0.3642675899
H 4.6845813897 1.5455626531 1.6868840245
H 4.2682250098 0.2102677971 -2.3776396790
C 6.1470242754 0.9779733874 -0.5548471973
H 6.5359388342 0.1018522109 -1.0855971819
H 6.4140407480 1.8608526060 -1.1500800042
H 6.6698807274 1.0552586131 0.4031476182
H -6.1825935306 1.4992341547 -1.9703004988
Cl 0.4144406070 -1.7953073948 1.5794307997
C -1.5883158763 -2.8779633463 -0.6715738158
H -3.4426065523 1.3132487076 -0.5799039578
H -1.4318077463 -3.5445319129 0.1827399251
H -0.7956305893 -3.0582349266 -1.4035176329
H -2.5469058515 -3.1368358774 -1.1318057753
41

C -2.6829592849 -1.7626187219 0.8084965055
C -3.3751086163 -0.3988681566 0.6606720895
C -3.9035937315 -0.0926182251 -0.7484076045
C -4.6411809823 1.2506895448 -0.8264038167
C -1.3644446790 -1.9813306617 0.0438326965
N -0.2129419493 -1.1009436927 0.4287960998
H -2.6993401842 0.4024275438 0.9808061204
H -4.2192789459 -0.3813420371 1.3645025095
H -4.5815178141 -0.8998882987 -1.0672998390
H -3.0755237020 -0.0788542983 -1.4690651867
H -5.4688722122 1.2542870104 -0.1022163552

H -1.5025394480 -1.7387092895 -1.0135480633
C -5.1841691803 1.5595597985 -2.2252019570
H -4.3739066240 1.5974876984 -2.9634290573
H -5.7022269354 2.5253326137 -2.2493678550
H -2.5037686084 -1.9696693140 1.8708548410
H -3.3682918558 -2.5486668171 0.4610010740
S -0.0072756395 0.4383724808 -0.3531240951
O -0.6203328380 1.5512838549 0.3779057930
O -0.3856194332 0.1562509977 -1.7433597335
C 1.7675340146 0.6256763076 -0.2483758563
C 2.3029666894 1.6208567304 0.5661976107
C 2.5888027893 -0.1973550169 -1.0238631674
C 3.6878444844 1.7839459480 0.6093310075
H 1.6432895191 2.2518102607 1.1513206022
C 3.9662758435 -0.0190247089 -0.9665126803
H 2.1522363026 -0.9597366308 -1.6602285634
C 4.5378413445 0.9725424335 -0.1515703285
H 4.1124229030 2.5563043795 1.2454116777
H 4.6116277143 -0.6573031374 -1.5650902150
C 6.0348531002 1.1657134851 -0.1183858738
H 6.5589871798 0.2077422198 -0.0268019956
H 6.3904461924 1.6409513544 -1.0417626074
H 6.3391664755 1.8009694165 0.7187952134
H -5.8959993237 0.7918133410 -2.5541066487
Cl 0.0870809672 -1.0011580329 2.1658459106
C -0.8858092378 -3.4342738412 0.1517741775
H -3.9560255911 2.0503267527 -0.5139055897
H 0.0411773872 -3.5791175194 -0.4101617792
H -1.6489823066 -4.1076797147 -0.2532085046
H -0.7063847975 -3.7162511093 1.1949382820

11

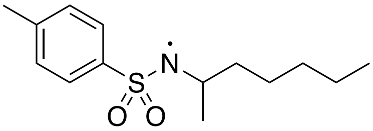

Name	E(B3LYP)	H(B3LYP)	E(RO-B2PLYP-D3)	H(RO-B2PLYP-D3)
tos_2Nheptan_react.conf.003	-1150.02755235	-1149.664752	-1149.56914619230000	-1149.20634584230000
tos_2Nheptan_react.conf.005	-1150.02592708	-1149.663243	-1149.56863120320000	-1149.20594712320000
tos_2Nheptan_react.conf.004	-1150.02682300	-1149.664083	-1149.56773163430000	-1149.20499163430000
tos_2Nheptan_react.conf.014	-1150.02566685	-1149.662824	-1149.56740616260000	-1149.20456331260000
tos_2Nheptan_react.conf.024	-1150.02458455	-1149.661836	-1149.56730961230000	-1149.20456106230000
tos_2Nheptan_react.conf.040	-1150.02452692	-1149.661744	-1149.56724513300000	-1149.20446221300000
tos_2Nheptan_react.conf.062	-1150.02324210	-1149.660399	-1149.56676738210000	-1149.20392428210000
tos_2Nheptan_react.conf.069	-1150.02270363	-1149.659901	-1149.56445667130000	-1149.20165404130000
tos_2Nheptan_react.conf.003	-1150.02755235	-1149.664752	-1149.56914619230000	-1149.20634584230000
tos_2Nheptan_react.conf.005	-1150.02592708	-1149.663243	-1149.56863120320000	-1149.20594712320000
NH_3	-56.5479477876	-56.509611	-56.53264223626600	-56.49430544866600
NH_2 radical	-55.8726196871	-55.849856	-55.85180557635200	-55.83180557635200
**11-H**	-1150.685000	-1150.308726	-1150.238282	-1149.862008
RSE (**11**/**11-H**)				-17.97

40
-1149.20634584
C -2.6829479246 1.2400449845 -1.5415171802
C -3.0513731388 -0.2494134045 -1.4551055260
C -3.8955961875 -0.6367620236 -0.2330403619
C -4.3205347834 -2.1110647182 -0.2445593619
C -1.7376480695 1.7514018174 -0.4338286483
N -0.4414140670 1.0915982952 -0.6026950909
H -3.4213158853 -2.7415561226 -0.2816845743
H -2.1789145473 1.5606448782 0.5524931122
C -1.4900525585 3.2710305122 -0.5668890164
C -5.1671361044 -2.5033484012 0.9704533616
H -5.4528806734 -3.5610097103 0.9347781806
H -6.0894682897 -1.9110011600 1.0197532119
H -2.2072361855 1.4463239282 -2.5088697268
H -3.5961752775 1.8502236060 -1.4979160649
S 0.2048089659 0.4923182092 0.8425732985
O -0.5895746575 -0.6936756899 1.2087886236
O 0.3587911248 1.6012817768 1.7983660651
C 1.8286766398 -0.0453266008 0.3307271889
C 1.9878381836 -1.3152139852 -0.2268450643
C 2.9173835000 0.8095774113 0.5029059429
C 3.2581608467 -1.7223832426 -0.6254601844
H 1.1315726352 -1.9722576580 -0.3355840320
C 4.1809376036 0.3833162884 0.0985987310
H 2.7731620245 1.7856051498 0.9536016989
C 4.3720697139 -0.8837890056 -0.4695257326
H 3.3886793773 -2.7096927538 -1.0615753361
H 5.0334116961 1.0451127302 0.2297306332
C 5.7498294266 -1.3495795187 -0.8754290619
H 6.4005393943 -0.5060740640 -1.1270139046
H 6.2327824842 -1.9031862366 -0.0593299694
H 5.7078729320 -2.0187571774 -1.7410839375
H -4.6169304344 -2.3377122804 1.9045426960
H -3.6035878633 -0.5137036433 -2.3680457948
H -3.3308783688 -0.4425684378 0.6876159004
H -2.4489616123 3.7969014178 -0.4998312250
H -1.0276658451 3.5027572274 -1.5317421540
H -0.8368826252 3.6240431657 0.2350091027
H -4.8814548285 -2.3217608937 -1.1667762682
H -4.7934823032 -0.0005853702 -0.1937126612
H -2.1323340606 -0.8503051895 -1.4678609403

40
-1149.20594712
C -3.1262788888 1.3858748759 -1.0057038674
C -3.5289323611 0.0887455880 -0.2810308921
C -2.7486713593 -1.1584360475 -0.7169768016
C -3.1272942771 -2.4152303206 0.0761076480
C -1.9107622915 2.1298335019 -0.4044299891
N -0.7100642192 1.3100486605 -0.5602388686
H -4.2093468350 -2.5906519352 -0.0120970821
H -2.1278352306 2.3539324986 0.6488865307
C -1.6493161113 3.4571175673 -1.1510709890
C -2.3690040655 -3.6656416918 -0.3811038749
H -2.6511671078 -4.5447649487 0.2096635360
H -1.2848992208 -3.5292125813 -0.2803705780
H -2.9295029729 1.1852122198 -2.0672714094
H -3.9598891339 2.0996569896 -0.9682554427
S 0.2018593341 1.1854995990 0.8634428870
O -0.6085811079 0.5263505937 1.9008524319
O 0.7878417998 2.5131790448 1.1164599121
C 1.5042897307 0.0677966000 0.3692982460
C 2.5923097977 0.5591319785 -0.3553027954
C 1.4237809702 -1.2776796103 0.7267543627
C 3.6015396546 -0.3202905942 -0.7360184597
H 2.6473331833 1.6128420210 -0.6075311049
C 2.4460486715 -2.1424758570 0.3380660695
H 0.5788920752 -1.6329641279 1.3067421281
C 3.5462139413 -1.6811341196 -0.3966672875
H 4.4498121820 0.0555246919 -1.3028868080
H 2.3889792349 -3.1924092494 0.6140391579
C 4.6638794715 -2.6171290810 -0.7900005999
H 4.3304160694 -3.6594794708 -0.7976886975
H 5.0561031812 -2.3769169969 -1.7840044561
H 5.5022965612 -2.5454604492 -0.0846718119
H -2.5745789445 -3.8919827791 -1.4347274178
H -3.4194166070 0.2299101596 0.8032055378
H -2.9224440895 -1.3342403554 -1.7893685417
H -0.8128508001 3.9922830797 -0.6948035095
H -2.5458060798 4.0847019363 -1.0950876477
H -1.4165834274 3.2667558370 -2.2036288860
H -2.9328789478 -2.2371620256 1.1428956417
H -1.6706380717 -0.9773951532 -0.6153121360
H -4.5988303142 -0.0893173701 -0.4584370711

40
-1149.20499163
C -2.9531811718 0.7541767781 -0.4796289518
C -3.1225025580 -0.4909178507 0.4010546274
C -4.3634996011 -1.3337103287 0.0651953342
C -4.3354869591 -2.0047812372 -1.3157018308
C -1.7834882153 1.6627963879 -0.0500673113
N -0.5339686131 0.9352646982 -0.2793394023
H -3.4177144800 -2.6035981693 -1.4054970570
H -1.9140595407 1.9461163387 1.0035009277
C -1.7228006003 2.9501145428 -0.9026267078
C -5.5552058656 -2.8971728677 -1.5687357888
H -5.6211894828 -3.6988267449 -0.8227358362
H -5.5109852094 -3.3657142500 -2.5585890276
H -2.7986714506 0.4718441968 -1.5281534417
H -3.8694131970 1.3607736922 -0.4421740708
S 0.5512382666 1.0350604514 1.0171043980
O -0.0180723679 0.2598777891 2.1318353455
O 0.9207017494 2.4472630587 1.2103503455
C 1.9713415449 0.1592091562 0.3806344997
C 2.0533862568 -1.2246771090 0.5432721304
C 2.9827961038 0.8718682283 -0.2636316651
C 3.1631249152 -1.8985626530 0.0399827650
H 1.2648557281 -1.7574881504 1.0639228250
C 4.0855437699 0.1796468025 -0.7598113642
H 2.9070379892 1.9493015148 -0.3637284519
C 4.1941821376 -1.2106834864 -0.6169785616
H 3.2325786667 -2.9766222779 0.1626349083
H 4.8769438995 0.7292177692 -1.2633541159
C 5.4078916987 -1.9485188888 -1.1293875363
H 6.1640322384 -2.0553255375 -0.3403482230
H 5.1498432463 -2.9567479062 -1.4698372667
H 5.8785915328 -1.4168654747 -1.9624415148
H -6.4866725595 -2.3199591234 -1.5150821823
H -2.2221824481 -1.1147702865 0.3219658815
H -4.4720058261 -2.1136936650 0.8320909816
H -2.6678457205 3.4944458775 -0.7971101701
H -1.5730199423 2.7052796532 -1.9590335767
H -0.9048511847 3.5923626811 -0.5671423814
H -4.2787668565 -1.2416412089 -2.1032739566
H -5.2641569104 -0.7058382489 0.1445868644
H -3.1777257777 -0.1767856091 1.4520049811
40
-1149.20456331
C -3.0546428424 0.8402444884 -0.5242405616
C -3.2822414551 -0.3842532152 0.3713810988
C -4.5374243060 -1.1977044596 0.0120843260
C -4.5438327806 -1.8451517660 -1.3845386653
C -1.8615980473 1.7135563369 -0.0868621702
N -0.6348122776 0.9402274537 -0.2882427239
H -4.4930864755 -1.0672377729 -2.1584207197
H -1.9976380608 2.0143836087 0.9611414072
C -1.7429562237 2.9876935472 -0.9532135838
C -3.4213871434 -2.8643727377 -1.6131472972
H -2.4307132796 -2.3975655011 -1.5725802274
H -3.5186607344 -3.3428667918 -2.5944682737
H -2.8899981403 0.5362764936 -1.5654232365
H -3.9501058372 1.4780645790 -0.5150814856
S 0.4373460389 1.0240182460 1.0201862344
O -0.1661074175 0.2697983426 2.1314258531
O 0.8396126116 2.4275541821 1.2105399188
C 1.8434332628 0.1097030883 0.4072444275
C 1.8871486136 -1.2753270646 0.5744100132
C 2.8827119379 0.7942435066 -0.2232973115
C 2.9866688713 -1.9790571980 0.0896535638
H 1.0774184258 -1.7862765260 1.0843420827
C 3.9745277382 0.0724611122 -0.7009179110
H 2.8362725404 1.8729889795 -0.3270960327
C 4.0450041593 -1.3198953026 -0.5530521832
H 3.0263509544 -3.0581866194 0.2159065628
H 4.7874042148 0.6000296271 -1.1936971396
C 5.2473484343 -2.0896086961 -1.0449306863
H 4.9728786385 -3.0998800929 -1.3656629992
H 5.7307777092 -1.5835599188 -1.8866804051
H 5.9985068474 -2.1930286649 -0.2506804620
H -3.4462497246 -3.6556883107 -0.8532030958
H -2.3931686424 -1.0258579857 0.3395004939
H -4.6639604295 -1.9882655419 0.7653681862
H -2.6699108693 3.5657035291 -0.8684536953
H -1.5862654568 2.7250237988 -2.0043344396
H -0.9082538696 3.6052087614 -0.6126417761
H -5.5127437790 -2.3426624403 -1.5263153755
H -5.4202661541 -0.5482063421 0.1036849603
H -3.3672612269 -0.0507307818 1.4143963276
40
-1149.20456106
C -3.0072814864 1.4535071314 -1.1693287810
C -3.5864778412 0.0820973988 -0.7797814040
C -2.7583211005 -1.1252198617 -1.2403158546
C -3.3769747399 -2.4873715824 -0.8833630397
C -1.8762018813 1.9862296992 -0.2617310495
N -0.6675973771 1.1863462902 -0.4560248223
H -2.7703970402 -3.2750021383 -1.3510204885
H -2.2216178443 1.9730907932 0.7814839188
C -1.5104879948 3.4424061697 -0.6374621431
C -3.4774267308 -2.7629116439 0.6225552385
H -3.8186367934 -3.7872907247 0.8123090489
H -4.1846251062 -2.0865103497 1.1152525795
H -2.6479008696 1.4366116486 -2.2069552033
H -3.8073015292 2.2045367087 -1.1245168427
S 0.1177098028 0.7927409780 0.9959571668
O -0.7133412718 -0.1964318907 1.7027641600
O 0.5092460894 2.0429943556 1.6680780662
C 1.5963884932 -0.0184846683 0.4072610248
C 1.5968006998 -1.4017285731 0.2254367787
C 2.7357782591 0.7442594059 0.1464765855
C 2.7555666449 -2.0217757050 -0.2368739703
H 0.7073692668 -1.9793366735 0.4529459937
C 3.8842442758 0.1061913103 -0.3153830179
H 2.7207387741 1.8160617512 0.3128415231
C 3.9138127764 -1.2819073535 -0.5139624703
H 2.7616315786 -3.0992850040 -0.3812468937
H 4.7744051372 0.6953126451 -0.5217143968
C 5.1746762897 -1.9668980248 -0.9840827348
H 5.8179362687 -2.2306445310 -0.1341345125
H 4.9509632804 -2.8930128915 -1.5231299592
H 5.7598042786 -1.3195339431 -1.6454347142
H -2.5061433910 -2.6310728187 1.1141262624
H -3.7262924865 0.0515204819 0.3087060042
H -2.6286329445 -1.0657187429 -2.3305068210
H -2.4029519562 4.0709808390 -0.5435065008
H -1.1500245549 3.4931622932 -1.6698492767
H -0.7350076049 3.8252473710 0.0299746723
H -4.3754197896 -2.5649426128 -1.3385191846
H -1.7503727643 -1.0669433576 -0.8115103256
H -4.5919039625 0.0004638669 -1.2177576475
40
-1149.20446221
C -3.0484521380 1.3528073020 -0.9291135102
C -3.4396351936 -0.0413346419 -0.4044964518
C -2.6228449533 -1.1943918880 -1.0063617173
C -2.8893289867 -2.5630827873 -0.3597214733
C -1.8720669949 2.0309616877 -0.1881815095
N -0.6435474799 1.2711567272 -0.4161973461
H -2.6474667225 -2.5033207837 0.7106625128
H -2.1275040875 2.1011559599 0.8779606987
C -1.6231832666 3.4559419405 -0.7310608436
C -4.3213519595 -3.0839862827 -0.5339784260
H -4.4304819258 -4.0864107147 -0.1041746905
H -4.5927397740 -3.1463368477 -1.5956610490
H -2.8131769076 1.3059811784 -2.0007426664
H -3.9001214536 2.0377156702 -0.8226897641
S 0.2116218342 0.9463497852 1.0110957774
O -0.5979501924 0.0421456897 1.8450955289
O 0.6768957919 2.2338474957 1.5542413855
C 1.6245938517 0.0415021550 0.3991722154
C 1.5874449267 -1.3527912074 0.3731694302
C 2.7507461123 0.7425159464 -0.0353841606
C 2.6942361649 -2.0487849902 -0.1088913067
H 0.7096926324 -1.8782349804 0.7337838450

C 3.8464362735 0.0290150284 -0.5140976582
H 2.7672134713 1.8261896417 0.0109583335
C 3.8371490736 -1.3731047485 -0.5585395916
H 2.6710825408 -3.1354315578 -0.1324145442
H 4.7259002511 0.5692779640 -0.8557204851
C 5.0439514230 -2.1363836429 -1.0495142683
H 5.7591182328 -2.3062353277 -0.2337800827
H 4.7638852724 -3.1170189880 -1.4471750252
H 5.5728647263 -1.5874249954 -1.8355603158
H -5.0551207681 -2.4358219718 -0.0417741251
H -3.3430255213 -0.0572659678 0.6901780252
H -2.8277627652 -1.2519236332 -2.0863348222
H -0.8183632751 3.9407145548 -0.1728227469
H -2.5382060466 4.0486385405 -0.6209424093
H -1.3496924811 3.4216276420 -1.7904494312
H -2.1916449782 -3.2943565168 -0.7907016402
H -1.5540262127 -0.9636016037 -0.9166022874
H -4.5036887963 -0.2015442014 -0.6203688294
40
-1149.20392428
C -3.2745596110 0.8783438937 -0.6118012391
C -3.6533850903 -0.2005645345 0.4176111073
C -2.7004860168 -1.4013660563 0.5550319025
C -2.4712768105 -2.2312485129 -0.7194835353
C -2.0572559133 1.7601296483 -0.2575071305
N -0.8299395310 0.9834755629 -0.4226758897
H -1.7455321557 -3.0215516982 -0.4839740548
H -2.1693694055 2.1384964995 0.7683976397
C -1.9638739070 2.9688765736 -1.2232512022
C -3.7378893661 -2.8766790639 -1.2943535504
H -4.2367585877 -3.5058342235 -0.5460653431
H -3.5004532635 -3.5111222908 -2.1561347794
H -3.1036451612 0.4347547091 -1.6005825123
H -4.1280443339 1.5608478721 -0.7211418100
S 0.3521756729 1.3125912861 0.7521603266
O -0.1729570583 0.9481248069 2.0777883377
O 0.8511874422 2.6761397902 0.5037138056
C 1.6342163977 0.1495441462 0.3108585288
C 2.5266232562 0.4701611964 -0.7143011338
C 1.7355164668 -1.0554820267 1.0051058402
C 3.5225052297 -0.4417496444 -1.0514328199
H 2.4441761158 1.4211364048 -1.2297913628
C 2.7410928395 -1.9549190719 0.6540233611
H 1.0434266331 -1.2756569624 1.8107221433
C 3.6466684435 -1.6652895023 -0.3751919220
H 4.2188415053 -0.1990271180 -1.8503996329
H 2.8250220601 -2.8960957300 1.1915464199
C 4.7511588541 -2.6314575666 -0.7310831653
H 5.6924242812 -2.3492644735 -0.2412166515
H 4.5100141072 -3.6509514457 -0.4144059573
H 4.9393643500 -2.6446687299 -1.8099548330
H -4.4642723536 -2.1277646153 -1.6311206462
H -3.7569843572 0.2749098729 1.4029343602
H -1.7316348813 -1.0525216294 0.9313896348
H -1.1104921643 3.6000900994 -0.9669516164
H -2.8836867454 3.5586062331 -1.1438220466
H -1.8543722882 2.6244791727 -2.2566121506
H -1.9917396873 -1.6082609453 -1.4850830299
H -3.1053736328 -2.0597404624 1.3376641582
H -4.6563273599 -0.5655364310 0.1582412673
40
-1149.20313003
C -3.0979522413 1.2275333904 0.5403937510
C -3.7042933756 0.1370128834 -0.3551502300
C -2.8594071708 -1.1334825586 -0.5216579098
C -3.5699457268 -2.2236574301 -1.3349494503
C -1.8937105076 2.0545129133 -0.0193788915
N -0.7353312084 1.1903845083 -0.2369978962
H -3.8266632206 -1.8264188495 -2.3276183137
H -1.6783817864 2.8248040180 0.7339454376
C -2.2095836798 2.7470859525 -1.3529258839
C -2.7319384559 -3.4959219979 -1.4974150657
H -1.7844811099 -3.2831101139 -2.0076401389
H -3.2635464929 -4.2544553239 -2.0834541406
H -3.8704787134 1.9762616476 0.7629119039
H -2.7887939937 0.7971996750 1.4989339670
S 0.3817578156 1.2217143083 1.0384331825
O -0.2427845791 0.6151051118 2.2263463004
O 0.9412723389 2.5815689315 1.1098494899
C 1.6502612022 0.1167037412 0.4390033995
C 1.5837957057 -1.2400079971 0.7575366319
C 2.6945603388 0.6271136217 -0.3333978415
C 2.5760438262 -2.0944691515 0.2819132773
H 0.7729251646 -1.6130068923 1.3739231417
C 3.6769676274 -0.2430932963 -0.7995664850
H 2.7372014043 1.6882707590 -0.5544418402
C 3.6349454948 -1.6127838212 -0.5008062163
H 2.5294372746 -3.1528871249 0.5259164483
H 4.4926207134 0.1482151076 -1.4025826953
C 4.7230171122 -2.5414260045 -0.9843382850
H 4.3519596478 -3.5638597412 -1.1083546561
H 5.1368944497 -2.2097338116 -1.9421837143
H 5.5532217902 -2.5800941698 -0.2666892694
H -2.4900797420 -3.9374688827 -0.5226302298
H -4.6693849123 -0.1432289585 0.0920057738
H -1.9046479618 -0.8816683856 -1.0005004277
H -3.1213635108 3.3474583544 -1.2550375339
H -2.3530959034 2.0176055947 -2.1552151707
H -1.3885370284 3.4109961444 -1.6393807811
H -4.5256384830 -2.4741295736 -0.8521222040
H -2.6062456430 -1.5285904230 0.4734963219
H -3.9489177274 0.5504658412 -1.3431646096
40
-1149.20305325
C -3.0377229201 1.4842238852 0.3109292098
C -3.7522174243 0.2704852000 -0.3006975031
C -2.9947854551 -1.0614213164 -0.2135398080
C -3.8151194239 -2.2522059730 -0.7271706331
C -1.7855177294 2.0522047475 -0.4396466474
N -0.6946898841 1.0818762603 -0.4489105247
H -4.1125265120 -2.0663377721 -1.7693915518
H -1.4900759307 2.9581142344 0.1076602987
C -2.0832293305 2.4278997058 -1.8977863565
C -3.0608666018 -3.5831332600 -0.6430503367
H -2.1385602659 -3.5532525259 -1.2363048316
H -3.6703264458 -4.4143202463 -1.0160499739
H -3.7415596616 2.3263616257 0.3565247506
H -2.7398229657 1.2617801080 1.3406945533
S 0.3647356736 1.2140873608 0.8725017656
O -0.3119355256 0.6296912707 2.0439022210
O 0.8952737631 2.5856370699 0.9316998591
C 1.6870438566 0.1203044195 0.3772380437
C 2.8161337057 0.6503935184 -0.2468899741
C 1.5764628824 -1.2482972567 0.6299514771
C 3.8426046566 -0.2107432958 -0.6304041032
H 2.8878611547 1.7190504815 -0.4184265466
C 2.6118113660 -2.0924143096 0.2380175931
H 0.6975644396 -1.6388099615 1.1316991802
C 3.7585351498 -1.5899255768 -0.3955260619
H 4.7252038798 0.1962715452 -1.1174852039
H 2.5311951340 -3.1594422435 0.4305411327
C 4.8886766029 -2.5120524397 -0.7860517666
H 4.5159267762 -3.4943158814 -1.0948983197
H 5.4792695385 -2.0968692912 -1.6088026500
H 5.5718772163 -2.6745605453 0.0580804615
H -2.7806167487 -3.8140261143 0.3922084840
H -4.7087427866 0.1636115768 0.2318827078
H -2.0580299865 -0.9896090395 -0.7814646862
H -2.9423196019 3.1071324401 -1.9411676173
H -2.3057752472 1.5414997619 -2.4987980050
H -1.2221420738 2.9336300829 -2.3449860444
H -4.7502881700 -2.3249691400 -0.1533339409
H -2.7025110414 -1.2410015385 0.8313047660
H -4.0207536063 0.4755537296 -1.3461643936
40
-1149.20165404
C -3.0125442089 1.1350853410 0.4412744481
C -3.6086811535 -0.0672709787 -0.3062253204
C -2.7174307812 -1.3168239606 -0.3612199022
C -3.3610363871 -2.5195877439 -1.0725794384

C -1.8547962917 1.9322168020 -0.2464656143
N -0.6677647819 1.0923224415 -0.3926175635
H -2.5994194875 -3.3023190703 -1.1893255827
H -1.6527529677 2.7938369943 0.4047230452
C -2.2291059882 2.4494548851 -1.6428045834
C -4.5758972581 -3.1166649163 -0.3513929254
H -5.4048320952 -2.4032397043 -0.2815092850
H -4.3160293859 -3.4232405751 0.6697293193
H -3.8032069787 1.8800608427 0.6049747291
H -2.6606425649 0.8251932968 1.4310850294
S 0.4752409650 1.3160755821 0.8400324012
O -0.0981228644 0.8410271927 2.1106849881
O 0.9860077928 2.6923545744 0.7279630202
C 1.7683191968 0.1877199415 0.3455881950
C 2.7692862364 0.6295167729 -0.5208533210
C 1.7697283949 -1.1143632631 0.8464606442
C 3.7751827323 -0.2568347589 -0.8977451175
H 2.7651795347 1.6542286088 -0.8767981347
C 2.7847626937 -1.9860788300 0.4578978207
H 0.9973414404 -1.4287210411 1.5401369396
C 3.7967039438 -1.5760139571 -0.4216211623
H 4.5614256887 0.0832441447 -1.5672020689
H 2.7952591610 -2.9999565283 0.8501650127
C 4.8758232355 -2.5367660119 -0.8603803388
H 4.5896303624 -3.0507592267 -1.7876274987
H 5.8200082432 -2.0177447834 -1.0550661025
H 5.0591830032 -3.3068849690 -0.1043183672
H -4.9507100018 -4.0016359088 -0.8785330413
H -4.5490173562 -0.3173393415 0.2034077032
H -1.7780802295 -1.0634814864 -0.8670295167
H -3.1624235850 3.0218725679 -1.5913903476
H -2.3605175301 1.6251283926 -2.3496521787
H -1.4427033913 3.1056110003 -2.0275001164
H -3.6492455319 -2.2251506658 -2.0922667239
H -2.4430876876 -1.6059174773 0.6644917489
H -3.8953530103 0.2245276650 -1.3261887571

12

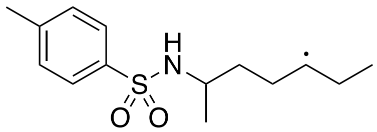

Name	E(B3LYP)	H(B3LYP)	E(RO-B2PLYP-D3)	H(RO-B2PLYP-D3)
tos_2Nheptan_15HAT_prod.conf002	-1150.01979369	-1149.658226	-1149.57287271210000	-1149.21130502210000
tos_2Nheptan_15HAT_prod.conf041	-1150.01805907	-1149.656117	-1149.57128384190000	-1149.20934177190000
tos_2Nheptan_15HAT_prod.conf026	-1150.01871224	-1149.656962	-1149.57091917480000	-1149.20916893480000
tos_2Nheptan_15HAT_prod.conf050	-1150.01736707	-1149.655396	-1149.57100695120000	-1149.20903588120000
tos_2Nheptan_15HAT_prod.conf060	-1150.01651056	-1149.654842	-1149.57012194470000	-1149.20845338470000
tos_2Nheptan_15HAT_prod.conf019	-1150.01877212	-1149.656981	-1149.56971630360000	-1149.20792518360000
tos_2Nheptan_15HAT_prod.conf055	-1150.01657367	-1149.655156	-1149.56932971230000	-1149.20791204230000
tos_2Nheptan_15HAT_prod.conf032	-1150.01869940	-1149.656952	-1149.56943767340000	-1149.20769027340000
CH_4	-40.518383	-40.469372	-40.483361	-40.434350
CH_3 radical	-39.838292	-39.804414	-39.807032	-39.773154
**11-H**	-1150.685000	-1150.308726	-1150.238282	-1149.862008
RSE (**12**/**11-H**)				-27.55

40
-1149.21130502
C 2.4054761238 2.1300823675 -0.6898371997
C 1.0036392618 2.5575500591 -1.1551772306
C 0.1161161160 3.0264797678 -0.0458306406
C -1.3710395380 3.0885073527 -0.1880049852
C 2.4278140847 0.8620123590 0.1818821594
N 1.9842352655 -0.2840624386 -0.6472263372
H -1.7291633181 2.1848594721 -0.7033647965
H 1.6776921624 0.9744765806 0.9725054649
C 3.8016783212 0.6233743664 0.8198053411
C -2.1099946078 3.2496429526 1.1474308836
H -1.9171715706 2.3946755950 1.8047830779
H -3.1927741758 3.3239031574 0.9960289121
H 3.0551680954 1.9717778121 -1.5624837106
H 2.8627150516 2.9461565205 -0.1136932114
S 1.2953233567 -1.6443489186 0.0797332414
O 1.6552430563 -1.7255515703 1.5020355784
O 1.5963594353 -2.7443389876 -0.8445945370
C -0.4631740570 -1.2979825461 0.0091164627
C -1.1127837781 -1.2845998059 -1.2284323643
C -1.1706692853 -1.0899498391 1.1910533581
C -2.4827834865 -1.0477987058 -1.2726847377
H -0.5515590511 -1.4641602345 -2.1396736448
C -2.5454366492 -0.8548765396 1.1282255146
H -0.6482098239 -1.1208663048 2.1408926626
C -3.2217361996 -0.8311575613 -0.0972929044
H -2.9915814555 -1.0370002798 -2.2337044688
H -3.1003593670 -0.6931016927 2.0489994081
C -4.7142492776 -0.6095602452 -0.1589576029
H -5.2474958735 -1.5603751771 -0.2896128002
H -5.0893281620 -0.1461198387 0.7587060995
H -4.9896233970 0.0329808968 -1.0024497827
H -1.7838397880 4.1561620132 1.6716937871
H 2.6920454414 -0.6187735199 -1.3001392470
H 0.5280173547 1.7239437314 -1.6897912908
H 1.1247424596 3.3592854388 -1.9094941140
H 0.5733002965 3.5958091651 0.7654438356
H 4.5738445334 0.5077375055 0.0473314716
H 3.7878574122 -0.2778995883 1.4375788285
H 4.0877814961 1.4733238684 1.4501262864
H -1.6542138701 3.9282737653 -0.8530379447
40
-1149.21056371
C 2.4040256421 2.0015962878 1.2879874908
C 2.8053034242 2.6484908356 -0.0525428665
C 1.7953400201 3.6224548533 -0.5746422622
C 1.4289364661 3.7064077517 -2.0217959884
C 1.2020668776 1.0445800160 1.1805229042
N 1.5868331935 -0.1212723388 0.3588661181
H 1.2591458010 2.6916102713 -2.4090245681
H 0.4138302863 1.5602447115 0.6220004349
C 0.6740307509 0.6234399410 2.5579250266
C 0.1959266184 4.5771877846 -2.2939242866
H -0.6866123278 4.1731508849 -1.7846959440
H -0.0272402630 4.6251501071 -3.3654521658
H 3.2584210119 1.4523361469 1.7093078347
H 2.1529570192 2.7874826417 2.0137643389
S 0.6191834576 -0.7669468619 -0.8408270241
O 1.4071428255 -1.8669618967 -1.4023618300
O 0.1465986346 0.3613948899 -1.6480894655
C -0.8371621577 -1.4974335894 -0.0770184783
C -2.0087527556 -0.7479706281 0.0435654210
C -0.7763492575 -2.8075700205 0.4048520833
C -3.1196845065 -1.3156272508 0.6664233693
H -2.0498674812 0.2568249734 -0.3632343301
C -1.8958260218 -3.3588486474 1.0230497205
H 0.1305270845 -3.3895921457 0.2760316177
C -3.0828899799 -2.6246618292 1.1656395547
H -4.0335163841 -0.7336106207 0.7585971217
H -1.8505475856 -4.3796618083 1.3951116736
C -4.3008525501 -3.2443415065 1.8083536955
H -4.8842994019 -3.8159722246 1.0745170777
H -4.0230129575 -3.9361195846 2.6105862140
H -4.9640170296 -2.4821417526 2.2297024019
H 0.3475224663 5.6037215635 -1.9372845602
H 2.1249558127 -0.8441024364 0.8294701758
H 2.9810648382 1.8571090361 -0.7911950392
H 3.7797937790 3.1487064629 0.0971664138
H 1.4781801333 4.4281819352 0.0899336301
H -0.1864590530 -0.0467300328 2.4683104999
H 0.3654022038 1.5008025291 3.1372285344
H 1.4531316799 0.1059630129 3.1333685400
H 2.2810835260 4.1029403134 -2.6080527081
40
-1149.20975133
C 2.3436083219 2.1720717242 -0.4646328803
C 0.9118459764 2.6255065881 -0.7988091619
C 0.0876780137 2.9529499336 0.4070834515
C -1.4082304845 2.9172402599 0.3954224200

C 2.4282918007 0.8171985299 0.2600176514
N 1.9496258165 -0.2388538583 -0.6636454880
H -1.7837939113 2.7455407770 1.4139585161
H 1.7234918074 0.8310520261 1.0986767712
C 3.8383420903 0.5320948080 0.7908139122
C -2.0553406459 4.2137823441 -0.1490459440
H -1.7495574342 4.4035224338 -1.1842255074
H -1.7608627144 5.0825670082 0.4508539316
H 2.9421310802 2.1204283604 -1.3854398998
H 2.8258939283 2.9276988051 0.1704867147
S 1.3129323546 -1.6796536139 -0.0526461216
O 1.7361889442 -1.8959076915 1.3376453973
O 1.5896844975 -2.6739118236 -1.0966668248
C -0.4525541158 -1.3650409218 -0.0146984556
C -1.1069277898 -1.2628635901 1.2107653322
C -1.1592737009 -1.2659578483 -1.2167263077
C -2.4862580637 -1.0481562288 1.2290852317
H -0.5406859302 -1.3588269941 2.1306747912
C -2.5330405087 -1.0516960479 -1.1797734169
H -0.6385934300 -1.3643233976 -2.1637075504
C -3.2192669585 -0.9411726424 0.0415770452
H -2.9995572855 -0.9676320494 2.1840505998
H -3.0863653017 -0.9752015553 -2.1128295459
C -4.7157973868 -0.7400078393 0.0647108829
H -5.2421834813 -1.6780831185 -0.1549056944
H -5.0587577954 -0.3918792045 1.0438363858
H -5.0335134311 -0.0089160359 -0.6870091774
H -3.1497535995 4.1436551205 -0.1277546087
H 2.6236318890 -0.4911752413 -1.3856526897
H 0.4134292064 1.8476223552 -1.3927663380
H 0.9853301837 3.5065204544 -1.4650927680
H 0.5770851852 3.4916344170 1.2202509309
H 3.8677545156 -0.4307306493 1.3068774907
H 4.1520761387 1.3131272036 1.4930485074
H 4.5665067828 0.5122283118 -0.0312072077
H -1.7540925343 2.0693932934 -0.2117907568
40
-1149.20934177
C 2.5948347155 1.6628285423 1.2178916602
C 2.5911632005 2.7579342080 0.1311828711
C 1.5046804115 3.7737015146 0.3092793640
C 0.6649042944 4.2724416147 -0.8243956852
C 1.3969700143 0.6984785735 1.1325982607
N 1.5093623315 -0.0961845348 -0.1077791342
H -0.2548611976 4.7273832880 -0.4322870252
H 0.4888796889 1.3007557325 1.0238810723
C 1.2843906404 -0.1934518752 2.3758099787
C 1.3827322514 5.3122703044 -1.7186391608
H 2.2881291370 4.8877698516 -2.1674500153
H 1.6781186903 6.1956771874 -1.1403947988
H 3.5245025626 1.0785178717 1.1576626587
H 2.5883736609 2.1328425854 2.2109715580
S 0.2230804104 -0.4067742774 -1.1286312408
O 0.8000734971 -1.1864478518 -2.2265530079
O -0.4602237368 0.8725304446 -1.3392469787
C -0.9451496904 -1.4787336550 -0.2777965853
C -0.7534285561 -2.8625108220 -0.3041130431
C -2.0227166562 -0.9220239051 0.4134512187
C -1.6429231695 -3.6867439983 0.3805719642
H 0.0701172044 -3.2836957028 -0.8716922124
C -2.9027858961 -1.7623760848 1.0943078535
H -2.1752715014 0.1519124890 0.3987335905
C -2.7289237465 -3.1530465156 1.0906440769
H -1.4966023689 -4.7640573333 0.3576007814
H -3.7439505984 -1.3300289058 1.6308204591
C -3.7059242409 -4.0588203918 1.8016725616
H -4.4851502400 -4.4130387213 1.1139045754
H -3.2077832564 -4.9444587300 2.2101235701
H -4.2084129870 -3.5402431185 2.6243864478
H 0.7272252923 5.6426298832 -2.5335159842
H 2.1574187977 -0.8790902559 -0.0809970556
H 2.5042889968 2.2814391514 -0.8529198099
H 3.5822094122 3.2460308058 0.1508179821
H 1.4709707077 4.3089043312 1.2594958580
H 2.1893449669 -0.8024395106 2.5026504799
H 0.4253863698 -0.8680273344 2.3076710749
H 1.1692471445 0.4167552894 3.2785841770
H 0.3548931295 3.4237754467 -1.4487947353
40
-1149.20916893
C 2.1199889629 2.1325460451 -0.7083371206
C 0.6937442631 2.4064742436 -1.2537716523
C -0.3248811157 2.7334100373 -0.2066368826
C -0.3754269552 4.0658766677 0.4732545888
C 2.2415916584 0.8805541318 0.1764268402
N 1.9173151392 -0.3124415461 -0.6414078236
H -0.0785059902 4.8529868212 -0.2372666251
H 1.4748878002 0.9315414999 0.9572889186
C 3.6204736020 0.7735418967 0.8392053199
C -1.7512748489 4.3977788637 1.0697778968
H -1.7417437295 5.3689452505 1.5771460825
H -2.0527365281 3.6404423293 1.8034867272
H 2.8178534299 2.0395820722 -1.5537695294
H 2.4608884381 2.9963593263 -0.1227924856
S 1.3083270568 -1.7056222794 0.0943338965
O 1.6443754696 -1.7400292934 1.5244304405
O 1.7004989661 -2.7967172629 -0.8056243403
C -0.4664654697 -1.4723703355 -0.0163117366
C -1.2029032790 -1.2338162651 1.1416254629
C -1.0951542502 -1.5606811219 -1.2622952773
C -2.5863631219 -1.0666515017 1.0449398561
H -0.6970809195 -1.1912856295 2.1000776740
C -2.4728873690 -1.3929773243 -1.3394305981
H -0.5101808027 -1.7664120073 -2.1528107171
C -3.2408666125 -1.1411333345 -0.1895231001
H -3.1643102583 -0.8814774715 1.9468824699
H -2.9652499173 -1.4621676177 -2.3066073039
C -4.7373302358 -0.9675434315 -0.2932269529
H -5.2167713566 -1.8800970685 -0.6686680755
H -5.1821519405 -0.7319361695 0.6781443330
H -4.9968003977 -0.1594227886 -0.9877527155
H -2.5203381647 4.4289243018 0.2897600764
H 2.6680384568 -0.6031651691 -1.2667488256
H 0.3739875710 1.5295316193 -1.8282266852
H 0.7737519212 3.2450300139 -1.9628805003
H -0.9489250299 1.9337500386 0.1888747276
H 3.6745980333 -0.1159454453 1.4719877150
H 3.8210981137 1.6548700000 1.4592926536
H 4.4125352276 0.7136075120 0.0808054406
H 0.3764050875 4.1194402628 1.2844498522
40
-1149.20903588
C 1.0496250755 1.3638445926 1.7794905215
C 0.8184779275 2.7742341710 1.1961842039
C 0.1462606117 2.8159318249 -0.1428375362
C 0.2543705360 4.0089342115 -1.0404717370
C 2.1079668359 0.4924633352 1.0657082636
N 1.7456814884 0.1593234068 -0.3264874681
H 1.2944816930 4.3676111017 -1.0559279276
H 2.1678492895 -0.4639555794 1.5948764603
C 3.5121367294 1.1122372453 1.0710476125
C -0.2313171382 3.7530633757 -2.4738626567
H -1.2788115424 3.4285995174 -2.4820114772
H 0.3634203222 2.9713206606 -2.9599716582
H 1.3624449147 1.4689934748 2.8267840385
H 0.0963269824 0.8206176084 1.7948837808
S 1.0958994857 -1.3156247617 -0.7625334878
O 1.8630646563 -2.3364951301 -0.0434721843
O 1.0061624631 -1.2733212768 -2.2238590700
C -0.5937149569 -1.4085042022 -0.1448416253
C -0.8513407810 -2.0063972435 1.0900115479
C -1.6342057928 -0.8635705771 -0.9018138955
C -2.1593034147 -2.0405947882 1.5724778866
H -0.0379027508 -2.4545471921 1.6507589171
C -2.9350302121 -0.9064179874 -0.4047717605
H -1.4248580867 -0.4335554053 -1.8760113349
C -3.2194678222 -1.4924645427 0.8376368973
H -2.3605586374 -2.5091661279 2.5329373477
H -3.7455927282 -0.4863312854 -0.9956935137
C -4.6377731500 -1.5647153967 1.3514595246

H -4.6673782403 -1.5940805049 2.4454832824
H -5.2300853562 -0.7065185202 1.0170978592
H -5.1422500069 -2.4694479883 0.9871501262
H -0.1587625669 4.6600791535 -3.0835999225
H 1.3474676607 0.9287489894 -0.8648524384
H 1.7659238850 3.3295144365 1.1558089798
H 0.1984735074 3.3288334054 1.9273195775
H -0.7077256059 2.1517393450 -0.2910704441
H 3.8164513591 1.3734528205 2.0916934322
H 3.5545947028 2.0207819819 0.4602717836
H 4.2332034324 0.4002801299 0.6604694443
H -0.3279441883 4.8489573677 -0.6137266450
40
-1149.20845338
C 1.1553904029 1.4021455165 -1.3973647188
C 0.7979131898 2.8917701953 -1.2296881144
C -0.0589948167 3.1775630881 -0.0370339458
C -0.1653863123 4.5439152277 0.5609531845
C 1.9921557025 0.8068197866 -0.2476190618
N 2.1199529180 -0.6580811882 -0.3853321814
H -0.7772638335 5.1981200494 -0.0907667791
H 1.4727391934 0.9790924061 0.7004752692
C 3.3969347026 1.4120578752 -0.1394274235
C -0.7711936030 4.5529023573 1.9714094280
H -0.8478954392 5.5727947337 2.3639251497
H -0.1591267585 3.9666650423 2.6658527779
H 0.2261346963 0.8254677133 -1.4865248735
H 1.7024118701 1.2655185888 -2.3420819488
S 1.2446721756 -1.7390609532 0.5390821904
O 1.3497496780 -1.2823163428 1.9265826489
O 1.7007940830 -3.0600786569 0.0998014637
C -0.4922413818 -1.6031524530 0.0850137495
C -1.3473110388 -0.8028627800 0.8440959846
C -0.9700766068 -2.2974427216 -1.0299650922
C -2.6859183755 -0.6864916045 0.4682515011
H -0.9701963527 -0.3033182516 1.7300440384
C -2.3087558425 -2.1698931496 -1.3906894132
H -0.3049208548 -2.9496619387 -1.5869309109
C -3.1862630163 -1.3587713487 -0.6544528466
H -3.3551969174 -0.0723885110 1.0659487988
H -2.6831665079 -2.7185175992 -2.2517930786
C -4.6304018482 -1.2111458709 -1.0707588563
H -4.7320342718 -0.4930579262 -1.8952724777
H -5.0459156628 -2.1629564498 -1.4190977420
H -5.2513173178 -0.8514642355 -0.2444255933
H -1.7788499580 4.1199299983 1.9685705370
H 2.2925925661 -1.0156922856 -1.3206813728
H 0.2826492035 3.2148316814 -2.1550884809
H 1.7106847538 3.5033068873 -1.1907198661
H -0.8257551390 2.4457553985 0.2209016850
H 3.9403479551 0.9470532714 0.6874113726
H 3.3505239117 2.4915069287 0.0388242082
H 3.9674735669 1.2443056214 -1.0616944332
H 0.8282456255 5.0172882223 0.5793860488
40
-1149.20792518
C 2.0588466826 2.1289692379 0.2281269578
C 0.5807390983 2.5015631352 0.0325657958
C 0.3766421576 3.9184323014 -0.4013584657
C -0.9162264589 4.6409060538 -0.1857011503
C 2.2963403012 0.6570363655 0.6060661476
N 1.8504433290 -0.2026159097 -0.5160969695
H -0.7444709241 5.7259638180 -0.2023080533
H 1.6492855981 0.4067065340 1.4549995601
C 3.7537879440 0.3865635021 0.9993638510
C -2.0023211473 4.3016645120 -1.2353092002
H -2.2413044249 3.2321801426 -1.2228295582
H -1.6645686707 4.5573191168 -2.2461243129
H 2.6207310064 2.3547378184 -0.6906914741
H 2.4946923358 2.7599519512 1.0135154033
S 1.3161090124 -1.7769907381 -0.2163173775
O 1.8492877926 -2.2864130611 1.0548410835
O 1.5517716341 -2.4890750150 -1.4777986145
C -0.4493462970 -1.5582815441 0.0105029230
C -1.2417124194 -1.1830384813 -1.0790225536
C -1.0168211653 -1.7963232702 1.2598200844
C -2.6124130294 -1.0351746416 -0.9007659182
H -0.7873337320 -1.0169678769 -2.0503765891
C -2.3956154060 -1.6430347515 1.4204252060
H -0.3850193966 -2.1030456307 2.0862654929
C -3.2120149004 -1.2619310596 0.3501508828
H -3.2318768408 -0.7437559184 -1.7457622240
H -2.8414542027 -1.8272260825 2.3946611087
C -4.7050088106 -1.1125585417 0.5205815177
H -5.0582853686 -0.1538746962 0.1231903088
H -5.2446752797 -1.9021569485 -0.0176792938
H -4.9964023348 -1.1701821396 1.5736102739
H -2.9265089646 4.8577110047 -1.0359758307
H 2.5110287131 -0.2483454362 -1.2911462824
H 0.0303047289 2.3193147260 0.9690123399
H 0.1457632638 1.7971287651 -0.6991946275
H 1.0824119475 4.3349616802 -1.1204972778
H 4.0481203148 1.0128109534 1.8493133181
H 4.4297262008 0.6186588442 0.1656022115
H 3.8878715971 -0.6620139927 1.2769386714
H -1.3127631607 4.4019050094 0.8122269303

13

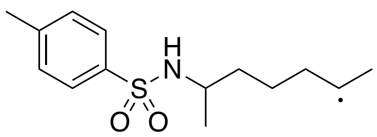

Name	E(B3LYP)	H(B3LYP)	E(RO-B2PLYP-D3)	H(RO-B2PLYP-D3)
tos_2Nheptan_16HAT_prod.conf005	-1150.018376	-1149.656936	-1149.571380	-1149.209939
tos_2Nheptan_16HAT_prod.conf029	-1150.017832	-1149.656176	-1149.571553	-1149.209896
tos_2Nheptan_16HAT_prod.conf006	-1150.018465	-1149.656936	-1149.570971	-1149.209442
tos_2Nheptan_16HAT_prod.conf080	-1150.015560	-1149.653689	-1149.571285	-1149.209414
tos_2Nheptan_16HAT_prod.conf025	-1150.018120	-1149.656352	-1149.570993	-1149.209225
tos_2Nheptan_16HAT_prod.conf049	-1150.016851	-1149.655216	-1149.570459	-1149.208824
tos_2Nheptan_16HAT_prod.conf070	-1150.015773	-1149.654210	-1149.570340	-1149.208777
tos_2Nheptan_16HAT_prod.conf057	-1150.016283	-1149.654819	-1149.570174	-1149.208710
tos_2Nheptan_16HAT_prod.conf066	-1150.015972	-1149.654301	-1149.570201	-1149.208530
tos_2Nheptan_16HAT_prod.conf043	-1150.017396	-1149.655676	-1149.570124	-1149.208404
CH_4	-40.518383	-40.469372	-40.483361	-40.434350
CH_3 radical	-39.838292	-39.804414	-39.807032	-39.773154
**11-H**	-1150.685000	-1150.308726	-1150.238282	-1149.862008
RSE (**13**/**11-H**)				-23.96

40
-1149.20993900
C 1.7920526041 2.6007241215 -0.4613976548
C 0.3359254290 2.6830177476 -0.9418662277
C -0.7047151254 2.8034627343 0.1850501670
C -2.1093223829 2.9206657397 -0.3149709294
C 2.1817383344 1.3073573837 0.2790054049
N 2.0068493417 0.1606522676 -0.6430440280
H -0.4644897939 3.6720687302 0.8199857709
H -2.3662103025 2.3961848364 -1.2341589740
H 1.4814264907 1.1409856429 1.1042262301
C 3.6029663969 1.3819206815 0.8513608818
C -3.2171591469 3.3942914591 0.5664104129
H -3.5282175422 2.6249002418 1.2976780248
H -4.1109989500 3.6621646434 -0.0088204875
H 2.4645324987 2.7158243061 -1.3241822748
H 2.0097745526 3.4474138301 0.2046954678
S 1.6892871403 -1.3803871785 -0.0293757478
O 2.1389021495 -1.5000060818 1.3646001882
O 2.1790283887 -2.2957750651 -1.0667048461
C -0.1032320730 -1.4339398992 -0.0026247105
C -0.7724629146 -1.4635543106 1.2187939835
C -0.8081578329 -1.4793945255 -1.2092995337
C -2.1676927344 -1.5230535832 1.2281443734
H -0.2039081050 -1.4529508778 2.1423512444
C -2.1968364584 -1.5394661267 -1.1808053145
H -0.2718093985 -1.4786715304 -2.1526449139
C -2.8996182733 -1.5574154762 0.0361105865
H -2.6927056475 -1.5495383305 2.1797281651
H -2.7482704952 -1.5797186873 -2.1172441905
C -4.4088680020 -1.6050580708 0.0473949279
H -4.7890025246 -2.3769578706 -0.6315184978
H -4.7961578281 -1.8147125684 1.0490169639
H -4.8360917124 -0.6486560038 -0.2805612646
H -2.9180744646 4.2719212030 1.1564130483
H 2.7489810614 0.0663606294 -1.3354654745
H 0.1084322240 1.8005149767 -1.5524587573
H 0.2352356121 3.5544078577 -1.6019249696
H -0.6220540563 1.9267673473 0.8561298393
H 4.3362701529 1.5365938987 0.0486381214
H 3.8529732907 0.4590565650 1.3806767813
H 3.6949815807 2.2208879374 1.5507584752

40
-1149.20989600
C 1.7640915243 2.5358234744 1.0957102951
C 2.2395456876 3.1719008801 -0.2191073428
C 1.1197048585 3.7601118460 -1.0989645866
C 0.3138536248 4.8339012427 -0.4394214604
C 0.8112398822 1.3341118671 0.9499100354
N 1.4601914787 0.2877317991 0.1360187897
H 0.4609382928 2.9564270824 -1.4564968048
H 0.8293377707 5.5132741451 0.2401437631
H -0.0633956256 1.6514464520 0.3751395311
C 0.3572027004 0.8092128168 2.3193301404
C -1.0176850333 5.2532025078 -0.9701059257
H -1.6177744990 4.3881593278 -1.2826818789
H -0.9262576171 5.9018951017 -1.8612397429
H 2.6405641581 2.2167520474 1.6797347352
H 1.2475734604 3.2897681470 1.7042116394
S 0.6639112905 -0.6104720167 -1.0246523296
O 1.7116747811 -1.4039498933 -1.6711995130
O -0.1873938697 0.3302008073 -1.7598510079
C -0.4400594127 -1.7739759779 -0.2078266357
C -1.7778994852 -1.4334908794 0.0000072988
C 0.0565784486 -3.0065968124 0.2231928534
C -2.6155858384 -2.3323861884 0.6590653189
H -2.1557083197 -0.4853542043 -0.3675602357
C -0.7944616437 -3.8925401166 0.8793315369
H 1.0901834255 -3.2721969863 0.0260480032
C -2.1406549712 -3.5718400143 1.1092695888
H -3.6584532325 -2.0692023181 0.8188121238
H -0.4093367758 -4.8534174764 1.2123801790
C -3.0625349242 -4.5540834142 1.7918093022
H -2.5314963135 -5.1462966084 2.5444769555
H -3.8971819896 -4.0453705667 2.2846887525
H -3.4913991769 -5.2590014483 1.0673197684
H -1.5943450303 5.8202565431 -0.2293140586
H 2.2014322588 -0.2442411774 0.5840324198
H 2.7932175856 2.4267698565 -0.8012360293
H 2.9534890167 3.9694774825 0.0302708769
H 1.5946769565 4.1568169157 -2.0179243186
H -0.3439276324 -0.0245192238 2.2171278311
H -0.1367194133 1.6033634890 2.8905115688
H 1.2165752848 0.4636410292 2.9087835951

40
-1149.20944200
C 1.4620677710 2.5618378832 -0.8065179986
C 0.0743585143 2.4378581504 -1.4533514414
C -1.1083703043 2.5839949157 -0.4761054665
C -1.1863845132 3.9156040426 0.2006865504
C 1.8550637103 1.4212650362 0.1497131506
N 1.8857693254 0.1494595510 -0.6112986233
H -1.0828400689 1.7834972912 0.2785457944
H -0.8842928078 4.7932456560 -0.3706213150
H 1.0723710955 1.3026011403 0.9063628143
C 3.1838986775 1.7059989421 0.8613159120
C -1.9972945991 4.1256176271 1.4365374504
H -3.0807754962 4.1820210678 1.2215348731
H -1.7307155363 5.0585114863 1.9468775118
H 2.2255171664 2.6267498286 -1.5957965947
H 1.5211789600 3.5032886470 -0.2448435466
S 1.6342491380 -1.3206652059 0.1798385879
O 1.9232927200 -1.2088682903 1.6163757170
O 2.3213553005 -2.3105311283 -0.6581944681
C -0.1371269567 -1.5576689401 0.0254738948
C -0.6889709269 -1.8241902709 -1.2314439314
C -0.9394521890 -1.5073806741 1.1630122554
C -2.0598250035 -2.0274925602 -1.3403392570
H -0.0488023628 -1.8773219594 -2.1060425667
C -2.3149146482 -1.7132782905 1.0345513981
H -0.4868243869 -1.3191661145 2.1305003103
C -2.8952720961 -1.9747308780 -0.2113197026
H -2.4923451595 -2.2353054623 -2.3162647981
H -2.9437749739 -1.6744312598 1.9204862889
C -4.3803258118 -2.2124575472 -0.3474763898
H -4.8167622460 -1.5751712513 -1.1255705466
H -4.5897900596 -3.2522713849 -0.6288668882
H -4.9067477420 -2.0095417706 0.5899556643
H -1.8698482066 3.2993257656 2.1493192875
H 2.7081849784 0.0396974553 -1.2034936991
H 0.0008494970 1.4725488722 -1.9670019327
H -0.0158488304 3.2127201332 -2.2272723330
H -2.0393459743 2.3937219356 -1.0455708817
H 3.1211472309 2.6359071114 1.4380924263
H 3.9968301866 1.8212976791 0.1319834454
H 3.4383629103 0.8903605591 1.5426663786
40
-1149.20941400
C 1.3757764287 2.6834094621 1.1072211954
C -0.1000220323 3.1208531180 1.0416572954
C -0.9024634672 2.7234734338 -0.2127161276
C -0.2877570046 3.1425464202 -1.5124116794
C 1.6714607507 1.1700200029 1.0968173812
N 1.6143450625 0.6854749166 -0.3036087791
H -1.9105885755 3.1724027993 -0.1191885826
H 0.3539347135 4.0234433728 -1.5118164183
H 0.8999683843 0.6616760081 1.6973473553
C 3.0495168915 0.8767091626 1.7024600854
C -0.8582257287 2.6994152574 -2.8204838297
H -1.1182976688 1.6329438590 -2.8120414746
H -0.1616613883 2.8703358733 -3.6483689777
H 1.9514272045 3.1499005841 0.2959313714
H 1.7942121012 3.0864847253 2.0376774830
S 1.3448330840 -0.9169106147 -0.6984906232
O 2.3222678766 -1.7249591640 0.0355136568
O 1.2554452376 -0.9323221643 -2.1614661465
C -0.2731243638 -1.3838390612 -0.0599484153
C -0.3900530377 -1.8966961442 1.2338086023
C -1.4043251279 -1.2252664260 -0.8651621531
C -1.6516287840 -2.2322357877 1.7245546232
H 0.4998103579 -2.0539803236 1.8339473633
C -2.6563775882 -1.5692118227 -0.3605873961
H -1.2935737295 -0.8626727292 -1.8819526429
C -2.8018668788 -2.0746410678 0.9400253566
H -1.7418993448 -2.6327898121 2.7314321324
H -3.5353616063 -1.4522499744 -0.9901614758
C -4.1601881213 -2.4749072876 1.4649341376
H -4.1952335979 -2.4366090567 2.5583823222
H -4.9490631407 -1.8228121530 1.0752593389
H -4.4103608093 -3.5012929888 1.1655558221
H -1.7878167052 3.2450925069 -3.0661363934
H 1.1443682084 1.3031124899 -0.9647368108
H -0.1197722233 4.2157618626 1.1321235903
H -0.6249419966 2.7353699855 1.9260193313
H -1.0906259608 1.6395791333 -0.2202248225
H 3.0659302954 1.1703062013 2.7589179593
H 3.8228367967 1.4425755728 1.1708285330
H 3.2929672028 -0.1847398239 1.6263433618
40
-1149.20922500
C 1.6846256677 2.6268365634 -0.2369924693
C 0.2196700723 2.7088510588 -0.6920903498
C -0.8121018844 2.6733986119 0.4668017887
C -2.2297256301 2.8055305505 0.0064265554
C 2.1236355223 1.2837345002 0.3760815993
N 1.9478367382 0.2176737069 -0.6378603216
H -0.5708600795 3.4851836161 1.1707203642
H -2.7166007703 1.9165046533 -0.3910949556
H 1.4507489991 1.0291118163 1.2016963416
C 3.5584523492 1.3455281563 0.9154278850
C -2.8623629409 4.1337545368 -0.2508081788
H -2.5737898091 4.5594918581 -1.2298414123
H -2.5667107154 4.8739507552 0.5053851518
H 2.3409600421 2.8423665513 -1.0933293938
H 1.8861607978 3.4143680357 0.5027134323
S 1.6975531364 -1.3806859744 -0.1534151155
O 2.2269726879 -1.6191120233 1.1966715068
O 2.1476905564 -2.1834368925 -1.2964889142
C -0.0894031350 -1.4794876260 -0.0337153970
C -0.8625180490 -1.4098507541 -1.1961698825
C -0.6854547223 -1.6623647196 1.2125846004
C -2.2462807129 -1.5098234101 -1.0967861515
H -0.3828269493 -1.2903623852 -2.1621644929
C -2.0757050565 -1.7599902578 1.2935300370
H -0.0637908547 -1.7385163008 2.0980338568
C -2.8759177083 -1.6809162384 0.1477834018
H -2.8505534806 -1.4626198365 -1.9996149246
H -2.5432434091 -1.9057679270 2.2640897287
C -4.3808579872 -1.7626919285 0.2422519700
H -4.8332802984 -0.7635975194 0.1912658271
H -4.8022499559 -2.3506503316 -0.5804101987
H -4.6991186948 -2.2190772989 1.1845832707
H -3.9572430953 4.0701277097 -0.2522734657
H 2.6657160378 0.2084919509 -1.3615909191
H 0.0088104940 1.8887264846 -1.3880067396
H 0.0829312339 3.6421019557 -1.2537500720
H -0.6960444242 1.7306273016 1.0184441480
H 3.8456024298 0.3872215973 1.3551058773
H 3.6499949485 2.1230072916 1.6826760544
H 4.2644672867 1.5885361682 0.1101603303
40
-1149.20882400
C 1.7106492488 2.6465192724 1.1330368653
C 2.3002187918 3.2303440962 -0.1626315353
C 1.3809827177 4.2148878337 -0.9138435783
C 0.1688118804 3.6172366162 -1.5564009146
C 0.7504639893 1.4526010539 0.9559632575
N 1.5133352105 0.3095824227 0.4144785947
H 1.9961694834 4.7165087102 -1.6877500394
H 0.2646475708 2.6316720738 -2.0074352134
H 0.0108029651 1.7125762786 0.1932030527
C 0.0398079990 1.1018752592 2.2705924646
C -0.9891792113 4.4727144607 -1.9549257195
H -1.8727092525 3.8733883161 -2.2030678436
H -0.7651652006 5.0880581603 -2.8465312026
H 2.5289818742 2.3209714085 1.7923739955
H 1.1771411257 3.4346686427 1.6826532019
S 0.9343603571 -0.7349492334 -0.7532650894
O 2.0436811651 -1.6565650120 -1.0105541103
O 0.3399004902 0.0990672984 -1.8018820973
C -0.4057219950 -1.7107574308 -0.0529718758
C -0.1087123789 -2.8764614756 0.6572981918
C -1.7277437314 -1.2907316206 -0.2114501962
C -1.1468180533 -3.6135009724 1.2221574136

H 0.9206932719 -3.2084923000 0.7456476729
C -2.7542148109 -2.0402540087 0.3615080356
H -1.9439208638 -0.3994892443 -0.7908793613
C -2.4831348622 -3.2092663667 1.0859338321
H -0.9167604843 -4.5227936508 1.7723870864
H -3.7843371450 -1.7151988035 0.2366590039
C -3.6034811538 -4.0337915075 1.6737263275
H -3.2890794813 -4.5418230611 2.5913776635
H -4.4764188345 -3.4159193932 1.9077379897
H -3.9319932563 -4.8091912747 0.9690304022
H -1.2674148373 5.1799152804 -1.1604660495
H 2.1266384627 -0.1701294842 1.0683986930
H 2.5897800511 2.4089156667 -0.8295879764
H 3.2264783734 3.7614432247 0.0916717121
H 1.0789552806 5.0253529917 -0.2302567713
H 0.7682142974 0.8461257906 3.0516590770
H -0.6389057145 0.2529014807 2.1457714508
H -0.5444249843 1.9558940189 2.6313190797
40
-1149.20877700
C 0.9475253962 1.9889475946 0.6691046825
C 0.6356062996 2.7899199301 -0.6031406447
C -0.6735536838 3.5944958443 -0.5010654107
C -1.9041886648 2.7537052422 -0.3741862192
C 2.2309882040 1.1303185812 0.6269457962
N 2.2154815779 0.0908322038 -0.4192941452
H -0.7495060538 4.2460037131 -1.3937265616
H -1.9078182079 1.7751446094 -0.8523043957
H 2.3042310641 0.5804701363 1.5704092868
C 3.5144345724 1.9571376995 0.4667219265
C -3.2065960911 3.3358286533 0.0647566864
H -3.9236374805 2.5587037183 0.3542255101
H -3.6897205356 3.9318408206 -0.7317772369
H 1.0400870573 2.6860720507 1.5143726827
H 0.0943883502 1.3402943557 0.9000534510
S 1.7170985295 -1.4811954453 -0.1708576230
O 2.2513882851 -1.8847689875 1.1322002334
O 2.0465246674 -2.1782954442 -1.4164506104
C -0.0772533773 -1.5070003350 -0.0237070015
C -0.6701714128 -1.5017682699 1.2400262979
C -0.8629890169 -1.5334167372 -1.1784169267
C -2.0606129288 -1.5041140374 1.3413852190
H -0.0452226208 -1.5204899789 2.1265387990
C -2.2512827418 -1.5350987531 -1.0589861434
H -0.3900934609 -1.5836213959 -2.1540197783
C -2.8724691542 -1.5126683204 0.1988460648
H -2.5225513650 -1.5099983660 2.3257242431
H -2.8634022629 -1.5687592604 -1.9571581665
C -4.3777588180 -1.4872456211 0.3181878743
H -4.7150377246 -1.9713636999 1.2404040414
H -4.7540868013 -0.4555727704 0.3357177629
H -4.8560660823 -1.9930233124 -0.5268739227
H -3.0794746619 4.0151903915 0.9191508135
H 2.0761504309 0.3913383327 -1.3786253667
H 0.5559649566 2.1098710275 -1.4643511265
H 1.4584189247 3.4801405652 -0.8277454068
H -0.6106538113 4.2915006682 0.3502724976
H 4.3919057822 1.3108465735 0.5558590649
H 3.5684278321 2.7333812956 1.2387358156
H 3.5586386081 2.4526580749 -0.5100565600
40
-1149.20871000
C 0.9067171009 1.9618226780 -0.3698872373
C 0.5581626281 3.1920898531 0.4801844591
C -0.7188289941 3.9102344603 0.0068895928
C -1.9659773503 3.0933782064 0.1284827351
C 2.1117808323 1.1495006622 0.1420698513
N 2.2687668664 -0.1153232447 -0.6053433071
H -0.5913892897 4.2386844749 -1.0375150408
H -2.0198960376 2.3577201942 0.9298960190
H 1.9358551816 0.8574750972 1.1831804434
C 3.4390727316 1.9158577907 0.0785411004
C -3.2296992383 3.4864548203 -0.5616282031
H -3.0426534758 3.8096241131 -1.5951441403
H -3.7345552825 4.3338345434 -0.0616614983
H 0.0316291911 1.3026628551 -0.4121483282
H 1.1065163449 2.2808443046 -1.4051648286
S 1.7586918965 -1.5924906758 -0.0137730248
O 2.2149801738 -1.6608906930 1.3765039134
O 2.1645063561 -2.5631982466 -1.0329041798
C -0.0409887507 -1.6039810262 0.0345076591
C -0.7594456097 -1.9365228762 -1.1171353323
C -0.7056002383 -1.2745169360 1.2167563428
C -2.1512042592 -1.9192013111 -1.0813401497
H -0.2307498640 -2.2313739839 -2.0179324611
C -2.1002770141 -1.2620365624 1.2347503401
H -0.1336421512 -1.0592434426 2.1130267125
C -2.8441905110 -1.5749076027 0.0892551510
H -2.7105217135 -2.1880017527 -1.9743607924
H -2.6187154877 -1.0175199572 2.1588038172
C -4.3536964750 -1.5340469130 0.1071681460
H -4.7835876290 -2.3356539104 -0.5029481640
H -4.7459210794 -1.6329595670 1.1241846680
H -4.7268296187 -0.5838073592 -0.2976429975
H -3.9524934721 2.6627214598 -0.5897704098
H 2.1713401654 -0.0602603543 -1.6154724431
H 1.3896647792 3.9071317322 0.4724627104
H 0.4264136069 2.8831534246 1.5277700040
H -0.8219631324 4.8486610425 0.5860999284
H 4.2525863675 1.2874819833 0.4508450022
H 3.3989321268 2.8283291432 0.6826022152
H 3.6739578934 2.2041058828 -0.9539675723
40
-1149.20853000
C 2.1730751563 2.2233981963 -1.3443127796
C 2.7896751672 3.0111221521 -0.1753809740
C 1.8157950363 3.5221545390 0.9063227744
C 0.7433966523 4.4270937381 0.3879866250
C 1.5493012220 0.8530320806 -1.0067319786
N 0.2434150307 1.0514829158 -0.3499522565
H 2.4252024412 4.0546762565 1.6627780033
H 1.0339185543 5.2009175479 -0.3238268417
H 2.1786435012 0.3535329243 -0.2622688914
C 1.4333242062 -0.0315672870 -2.2583096378
C -0.5808674040 4.5465327652 1.0677009096
H -1.3132079426 5.0754711621 0.4459762405
H -0.5116883600 5.1074933710 2.0185381368
H 1.4149900593 2.8396247978 -1.8485121152
H 2.9627285040 2.0457048971 -2.0867280996
S -0.3401143640 0.1186426990 0.8992270821
O -1.5802738733 0.7847473160 1.3119408646
O 0.7855418977 -0.0956235611 1.8135426610
C -0.8096715807 -1.4995859807 0.2664828687
C 0.0893746748 -2.5640324245 0.3493299904
C -2.0689223944 -1.6733278798 -0.3136103664
C -0.2758023794 -3.8063225102 -0.1684554917
H 1.0516518353 -2.4198026556 0.8289799548
C -2.4168743268 -2.9207704621 -0.8251553353
H -2.7704922095 -0.8458604399 -0.3426548985
C -1.5290474437 -4.0055427587 -0.7629807051
H 0.4226721130 -4.6369760883 -0.1018818376
H -3.3978313354 -3.0577321083 -1.2740710733
C -1.9307455869 -5.3618023583 -1.2918405936
H -2.5556544557 -5.2742364818 -2.1870257471
H -1.0559807545 -5.9691865808 -1.5448129867
H -2.5112030868 -5.9187945113 -0.5445280381
H -0.9909513039 3.5601802290 1.3218370019
H -0.4996387741 1.4540620524 -0.9131257577
H 3.3180641462 3.8744647533 -0.6048818207
H 3.5611986494 2.3929896522 0.3045746084
H 1.3678493358 2.6763095431 1.4382255164
H 0.9770591888 -0.9979456092 -2.0242635676
H 2.4218737762 -0.2167431275 -2.6935055890
H 0.8197651215 0.4592041117 -3.0250383628
40
-1149.20840400
C 1.3273314090 2.6055351898 -0.5695861522
C -0.1031935216 2.5079112938 -1.1179125187
C -1.2227808927 2.5098047317 -0.0387265064
C -1.2788549269 3.7399526098 0.8120648967

C 1.8182545388 1.3902377175 0.2385212184
N 1.8113634737 0.1984507133 -0.6423936096
H -1.1160078340 1.6193067163 0.5937912324
H -0.7791794270 3.7372141608 1.7785378673
H 1.1016443602 1.1781887669 1.0391625271
C 3.1945624215 1.6422591378 0.8676218168
C -1.7851450194 5.0425097512 0.2838177898
H -2.6239340105 4.8957726158 -0.4106870935
H -1.0151632560 5.6039165664 -0.2763443962
H 2.0240174511 2.7643119131 -1.4060132222
H 1.4169532397 3.4923926431 0.0717351745
S 1.6598644380 -1.3478166911 0.0199590815
O 2.0640596302 -1.3623847813 1.4327806509
O 2.2987357730 -2.2314533088 -0.9624755678
C -0.1117724317 -1.6249118647 -0.0147653400
C -0.8222648818 -1.6854433173 1.1818505798
C -0.7554328979 -1.8082739424 -1.2425789112
C -2.1988871422 -1.9183232863 1.1440170637
H -0.2987406614 -1.5616257358 2.1236097496
C -2.1258682269 -2.0404721365 -1.2609311207
H -0.1852603201 -1.7765166599 -2.1653116821
C -2.8703400284 -2.0978217783 -0.0701479195
H -2.7562798838 -1.9655569492 2.0761314328
H -2.6296890201 -2.1844095097 -2.2138981227
C -4.3565404377 -2.3623683402 -0.1100300945
H -4.5683745151 -3.3733767529 -0.4802787578
H -4.8071900454 -2.2702739895 0.8826506226
H -4.8683471114 -1.6613053739 -0.7799537029
H -2.1244224823 5.7041518343 1.0899740011
H 2.5827180746 0.1700097426 -1.3082179452
H -0.1983539736 1.5967076996 -1.7205108389
H -0.2716971682 3.3543832316 -1.7966180308
H -2.1792933472 2.3906596260 -0.5715550615
H 3.5190871618 0.7729761597 1.4448246421
H 3.1611417677 2.5114177660 1.5346969848
H 3.9432856590 1.8481292880 0.0910741890

14

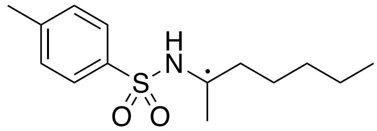

Name	E(B3LYP)	H(B3LYP)	E(RO-B2PLYP-D3)	H(RO-B2PLYP-D3)
Tosyl_NH_2_heptyl_C2_radical_0008	-1150.028518	-1149.666142	-1149.579216	-1149.216841
Tosyl_NH_2_heptyl_C2_radical_0082	-1150.028913	-1149.666688	-1149.57791	-1149.215685
Tosyl_NH_2_heptyl_C2_radical_0076	-1150.028648	-1149.666226	-1149.577924	-1149.215502
Tosyl_NH_2_heptyl_C2_radical_0058	-1150.029511	-1149.667267	-1149.577388	-1149.215144
Tosyl_NH_2_heptyl_C2_radical_0035	-1150.028816	-1149.666668	-1149.576602	-1149.214454
Tosyl_NH_2_heptyl_C2_radical_0095	-1150.030134	-1149.667889	-1149.576169	-1149.213924
Tosyl_NH_2_heptyl_C2_radical_0042	-1150.02903	-1149.666794	-1149.576085	-1149.213849
Tosyl_NH_2_heptyl_C2_radical_0060	-1150.02865	-1149.666592	-1149.575112	-1149.213054
CH_4	-40.518383	-40.469372	-40.483361	-40.434350
CH_3 radical	-39.838292	-39.804414	-39.807032	-39.773154
**11-H**	-1150.685000	-1150.308726	-1150.238282	-1149.862008
RSE (**14**/**11-H**)				-48.02

40

C 2.5727791959 -0.8938053769 -1.2808587056
C 3.3730209639 -0.5201172636 -0.0068311891
C 4.8794478327 -0.3963731519 -0.2669432577
C 5.6863850770 -0.0406301518 0.9886848708
C 1.0867264583 -0.9702718081 -1.0812909681
N 0.3133085896 0.1850380333 -1.2872614614
H 3.1931851073 -1.2819991482 0.7636647314
H 2.9831624164 0.4205283929 0.4021651895
H 5.0547491593 0.3688461749 -1.0383399071
H 5.2582310366 -1.3410254708 -0.6860756397
H 5.3057778298 0.9013756509 1.4073578283
C 0.4403547729 -2.1350459637 -0.4047207724
C 7.1900441527 0.0872395643 0.7236325923
H 7.3972089085 0.8705673921 -0.0159862400
H 7.6061673715 -0.8509980756 0.3359375510
H 2.8044416365 -0.1584814895 -2.0675484466
H 2.9360263494 -1.8628873536 -1.6518766023
S -0.4017262545 1.0815502970 0.0351055880
O -0.4720672121 2.4592589019 -0.4720544892
O 0.2678892540 0.7597737330 1.3029939264
C -2.0647026994 0.4192990787 0.1211371157
C -2.9401506318 0.6160591600 -0.9508995058
C -2.4798012376 -0.2394029249 1.2756823098
C -4.2395238309 0.1302146523 -0.8601852029
H -2.6061585719 1.1426258529 -1.8388226328
C -3.7893460079 -0.7174272666 1.3499898334
H -1.7854047765 -0.3701709125 2.0984743386
C -4.6859316676 -0.5427693398 0.2898910277
H -4.9229784608 0.2772368300 -1.6931205259
H -4.1174151054 -1.2324573351 2.2493415722
C -6.1059350275 -1.0490846558 0.3762478716
H -6.2583860705 -1.6664959318 1.2665151821
H -6.8196279036 -0.2167082999 0.4218027492
H -6.3691576270 -1.6502044956 -0.5019660632
H 7.7373627493 0.3409511483 1.6386805612
H 0.7743774488 0.8976854758 -1.8526264005
H 5.5135678445 -0.8062159340 1.7582432458
H -0.5939855204 -2.2649274156 -0.7446570942
H 0.4107562337 -2.0284526126 0.6922656881
H 0.9936688204 -3.0563158452 -0.6211487046
40

C 2.5930699846 -0.8270116772 -2.0077323332
C 3.5897694021 -0.0163118659 -1.1380327596
C 3.8788173042 -0.6327349399 0.2348962080
C 4.9278132091 0.1504377380 1.0351284688
C 1.1916904723 -0.9212122200 -1.4763275595
N 0.3606888112 0.2070244234 -1.6042315271
H 3.2031937067 1.0030485469 -1.0020982357
H 4.5282905575 0.0822701979 -1.7028696175
H 4.2223479667 -1.6707933858 0.1043478034
H 2.9486648436 -0.6771622329 0.8148867455
H 5.8606143052 0.2094849154 0.4556005318
C 0.6717199376 -2.0880755186 -0.7022487695
C 5.2177252677 -0.4635453471 2.4085477127
H 4.3101003851 -0.5006848331 3.0231405409
H 5.9678603316 0.1178348318 2.9569866286
H 2.5731886639 -0.3668618422 -3.0103843339
H 2.9861815619 -1.8421622041 -2.1484967582
S -0.1806191985 1.1252268733 -0.2236487291
O -0.3704323900 2.4819734996 -0.7548986411
O 0.6777312100 0.8666788537 0.9408197102
C -1.7893700372 0.4183743388 0.1270415810
C -2.8168173394 0.5460414841 -0.8125265294
C -2.0108257446 -0.2033252981 1.3531566317
C -4.0720691682 0.0277934186 -0.5158148519
H -2.6329198641 1.0452616133 -1.7581483374
C -3.2789617373 -0.7148688668 1.6348658655
H -1.2011333555 -0.2798274028 2.0706419249
C -4.3244115180 -0.6101443394 0.7107988530
H -4.8734368322 0.1215751673 -1.2448771789
H -3.4561399061 -1.2008734398 2.5909321409
C -5.6976760679 -1.1574096892 1.0187279311
H -6.0327137089 -1.8532275576 0.2402246567
H -5.7126629509 -1.6889237553 1.9748443449
H -6.4413410469 -0.3524727741 1.0718060394
H 5.5957963039 -1.4893154398 2.3134779260
H 0.7040856952 0.9083401474 -2.2602647181
H 4.5806145904 1.1848761137 1.1638759034
H 1.2508196429 -2.9884574344 -0.9335019742
H -0.3816481032 -2.2823931919 -0.9425664383
H 0.7296964792 -1.9408969834 0.3887304939
40

C 2.5772291663 0.4790711330 -0.7930959473
C 2.2501346917 -0.3253265674 0.4905150080
C 3.3417383427 -1.3407220613 0.8515414172
C 3.0333837098 -2.1424495793 2.1228472109

C 1.5438597920 1.4942120661 -1.1873660756
N 0.5219699350 1.0903113503 -2.0527362438
H 1.2917753065 -0.8449341222 0.3573903957
H 2.1066225530 0.3740187744 1.3253811588
H 4.3008117445 -0.8162403444 0.9772025863
H 3.4851744110 -2.0361338782 0.0106904676
H 2.8951427497 -1.4476821716 2.9634261957
C 1.5238293048 2.8766868390 -0.6190379038
C 4.1242960233 -3.1591772368 2.4746732306
H 3.8754257880 -3.7140721395 3.3864570748
H 5.0893589904 -2.6643035534 2.6391613013
H 3.5387180460 0.9909869194 -0.6482787414
H 2.7349965503 -0.2357011498 -1.6173147032
S -1.1807237070 1.2376513948 -1.7776386805
O -1.7711724679 0.5974399566 -2.9564425942
O -1.4292976592 2.6377547456 -1.4338430432
C -1.6030101239 0.2484090358 -0.3414922971
C -1.5603519196 0.8206692676 0.9315968840
C -1.9180873944 -1.1033063519 -0.5080107806
C -1.8342255248 0.0261910715 2.0435931774
H -1.3401580766 1.8772986018 1.0420717079
C -2.1898221960 -1.8807374667 0.6148527228
H -1.9688272351 -1.5271437744 -1.5057590038
C -2.1519645633 -1.3321047004 1.9061570849
H -1.8075105313 0.4711632928 3.0353706399
H -2.4437721360 -2.9303804225 0.4871931489
C -2.4808553426 -2.1776363103 3.1132263747
H -2.0120120035 -1.7824449089 4.0200013692
H -3.5642258441 -2.2046331111 3.2903841230
H -2.1488085464 -3.2127557361 2.9806945050
H 4.2612985538 -3.8892676491 1.6674548360
H 0.6826346229 0.2067324461 -2.5315728653
H 2.0730545380 -2.6625370099 1.9978960908
H 1.0403013919 3.5767460945 -1.3060521933
H 2.5496456889 3.2143519960 -0.4243928291
H 0.9814093233 2.9511345820 0.3379520100
40

C 2.5202494397 -1.2215992547 -1.9096914316
C 3.6971269458 -1.3057878586 -0.9017040213
C 3.9278010393 -0.0278792845 -0.0873924720
C 5.1270360091 -0.1291569231 0.8647951468
C 1.1470641864 -1.1152275328 -1.3118385355
N 0.4675999667 0.1138562418 -1.3542616227
H 4.6088264260 -1.5506573418 -1.4663557646
H 3.5233433550 -2.1507544291 -0.2207716155
H 3.0274347413 0.2097022809 0.4926079788
H 4.0870749902 0.8163839334 -0.7764928300
H 4.9695838976 -0.9713531887 1.5537585691
C 0.4915806379 -2.2650585759 -0.6186379494
C 5.3613359262 1.1517160664 1.6722424122
H 5.5592488748 2.0053063226 1.0120686131
H 6.2180957657 1.0480202506 2.3481921882
H 2.6994675783 -0.3717791238 -2.5856960568
H 2.5553955388 -2.1234399433 -2.5397798868
S -0.0987213847 0.9428640826 0.0709534390
O -0.0807514024 2.3599087966 -0.3176250107
O 0.5963704080 0.4605818541 1.2727880649
C -1.8055216903 0.4091993702 0.1932322047
C -2.2171168477 -0.3193049027 1.3063335846
C -2.7140128333 0.7756283949 -0.8042845931
C -3.5572469043 -0.6960259102 1.4127816175
H -1.4964453948 -0.5816255333 2.0731282419
C -4.0437768005 0.3892424799 -0.6826577821
H -2.3818937530 1.3558263596 -1.6588701186
C -4.4876470098 -0.3514731685 0.4260973832
H -3.8824383815 -1.2651669779 2.2799644977
H -4.7531659363 0.6688852821 -1.4579501987
C -5.9391053009 -0.7499013144 0.5472823656
H -6.2877749669 -1.2648625469 -0.3556840280
H -6.1026494424 -1.4155061997 1.4000212532
H -6.5802218421 0.1302198832 0.6831533602
H 4.4828940626 1.4013175077 2.2790747145
H 0.9277048089 0.8316110454 -1.9121109207
H 6.0322127824 -0.3720933824 0.2892402127
H -0.5838055257 -2.3005817873 -0.8368846976
H 0.5953153293 -2.2210619149 0.4772553324
H 0.9384529241 -3.2104452096 -0.9468380322
40

C 2.3930475548 -0.1512672282 -1.0998329464
C 2.7114709006 -0.8756842909 0.2233153646
C 4.1412076829 -0.6152895086 0.7133149530
C 4.4705252506 -1.3145531358 2.0383623806
C 1.0406317232 -0.4609346583 -1.6812699996
N 0.1949923967 0.6038439238 -2.0441166441
H 2.5601587309 -1.9585392537 0.1044596436
H 1.9974521823 -0.5444138475 0.9887351102
H 4.2923890895 0.4679822002 0.8272022783
H 4.8557279822 -0.9429303872 -0.0569415281
H 3.7581537009 -0.9846297864 2.8078354502
C 0.7544698382 -1.7824758401 -2.3264797877
C 5.9004887026 -1.0505316309 2.5210704003
H 6.6375280721 -1.4009319909 1.7878806665
H 6.1045289634 -1.5620965615 3.4687232446
H 2.4786983331 0.9279444591 -0.9497328298
H 3.1659836927 -0.4317208932 -1.8394578702
S -0.5762059060 1.6286965258 -0.8929903005
O -1.3138519744 2.5944370905 -1.7100531444
O 0.4555008936 2.0291664585 0.0639938560
C -1.7751152343 0.6200828421 -0.0199035596
C -3.0936070197 0.5725626155 -0.4808589868
C -1.3724630005 -0.1462630222 1.0763668993
C -4.0101242174 -0.2526316402 0.1660115842
H -3.3931395827 1.1895074605 -1.3217156945
C -2.3031896711 -0.9699993865 1.7071095292
H -0.3532792352 -0.0794874328 1.4404071752
C -3.6322002872 -1.0368323526 1.2662601372
H -5.0386504680 -0.2845434632 -0.1855240119
H -1.9930764485 -1.5655831524 2.5623300972
C -4.6421039800 -1.9043621355 1.9788572738
H -5.1773925416 -1.3325970534 2.7484931331
H -5.3936089493 -2.2971470372 1.2859536233
H -4.1619815334 -2.7521478056 2.4779919858
H 6.0739842362 0.0212807598 2.6768220824
H -0.4782507964 0.3973649420 -2.7800597611
H 4.3163236925 -2.3974509434 1.9253709728
H -0.2850166561 -2.0997311128 -2.1680331970
H 1.4066381830 -2.5633678414 -1.9227593347
H 0.9228818311 -1.7608752431 -3.4188343456
40

C 2.2465217517 0.7438377049 -0.2359688655
C 2.2762291866 -0.8008468237 -0.2197051361
C 2.6557936760 -1.3751654715 1.1503591020
C 2.6966993450 -2.9085223012 1.1781022767
C 1.8086582447 1.3310352711 -1.5442067720
N 0.4902199583 1.0845869691 -1.9702056632
H 2.9866928719 -1.1537220329 -0.9801257184
H 1.2908999974 -1.1796425583 -0.5184320816
H 1.9368680175 -1.0189670601 1.9032014430
H 3.6369484962 -0.9811466798 1.4559630352
H 1.7156839408 -3.3004878847 0.8742448302
C 2.6192433931 2.3474741149 -2.2867209480
C 3.0726635895 -3.4785550809 2.5497402225
H 3.0924954030 -4.5744199408 2.5370592900
H 2.3548900786 -3.1670674946 3.3188065086
H 1.5925782072 1.0902887360 0.5812784678
H 3.2453916182 1.1293598470 0.0028550516
S -0.8816928223 1.7674191751 -1.1180293517
O -1.8820393227 1.9779709630 -2.1739237823
O -0.4447473505 2.8577097825 -0.2365256386
C -1.4378924160 0.4192176790 -0.0755134128
C -1.9595535587 -0.7362269411 -0.6640520369
C -1.3832020910 0.5577185034 1.3094182936
C -2.4133794619 -1.7656119432 0.1536569451
H -2.0099698054 -0.8235686673 -1.7443929586
C -1.8473210825 -0.4842593094 2.1141882097
H -0.9870912174 1.4697601485 1.7427466862
C -2.3664201862 -1.6571761078 1.5536904281

H -2.8157168567 -2.6684157148 -0.2996644491
H -1.8071604402 -0.3797518212 3.1955067219
C -2.8864472032 -2.7746768721 2.4260265139
H -3.9833538180 -2.8110892777 2.4066723634
H -2.5260211328 -3.7508999235 2.0824507663
H -2.5785778829 -2.6467537434 3.4681667878
H 4.0644016181 -3.1314514540 2.8651049502
H 0.3176681249 1.3333230442 -2.9443474356
H 3.4130999218 -3.2656764957 0.4246461808
H 3.6920473743 2.1900753437 -2.1316537656
H 2.3853591100 3.3751222776 -1.9572265134
H 2.4283301333 2.3131090193 -3.3687137815
40

C 2.9234475809 0.1922741162 -1.1899586393
C 2.5915349774 -1.1580850923 -0.5151406839
C 2.4646431384 -1.0699080356 1.0099428721
C 2.1071299285 -2.4071840017 1.6703303947
C 1.8240845042 1.2203099671 -1.1534074597
N 0.7420000317 0.9560049220 -2.0208741716
H 3.3770848932 -1.8806554525 -0.7764670898
H 1.6581674049 -1.5612560910 -0.9352384092
H 1.7024579098 -0.3219984394 1.2669438957
H 3.4131714155 -0.6999336498 1.4281632506
H 1.1519273224 -2.7657214755 1.2618802471
C 2.1099835338 2.6436748370 -0.7796742745
C 2.0053418862 -2.3150387255 3.1962584013
H 1.7479278154 -3.2834307329 3.6405900580
H 1.2349360006 -1.5936624500 3.4954977222
H 3.8140447241 0.6197422168 -0.7131829877
H 3.2145020800 -0.0048916719 -2.2388439359
S -0.9060537740 1.2755268475 -1.6834806585
O -1.6069849590 0.7218209442 -2.8443724139
O -1.0045791611 2.6857816926 -1.3067111437
C -1.3683758658 0.3053685754 -0.2462126129
C -1.2038030658 0.8396485867 1.0339589538
C -1.8666418339 -0.9871959228 -0.4241051351
C -1.5409918858 0.0637893056 2.1416286561
H -0.8426143174 1.8551598547 1.1548681154
C -2.2003571255 -1.7469642396 0.6952753704
H -2.0105285225 -1.3777167541 -1.4262482395
C -2.0444815350 -1.2369857925 1.9926388980
H -1.4214479275 0.4799997123 3.1390952394
H -2.5981660652 -2.7496548237 0.5591924756
C -2.4452520952 -2.0524867116 3.1987727680
H -3.4696778243 -1.8115533801 3.5121193695
H -2.4128627435 -3.1257174741 2.9862231438
H -1.7895986038 -1.8543379542 4.0531280502
H 2.9542225256 -1.9876962832 3.6389203346
H 0.7930003760 0.0768133750 -2.5308087736
H 2.8594887135 -3.1615043296 1.3984077006
H 2.6653368690 3.1626326991 -1.5818633841
H 2.7310082752 2.6829029609 0.1220416409
H 1.1963730705 3.2162927430 -0.6067242559
40

C 1.6970963187 -0.8778268465 -1.1297847184
C 2.6881301130 -0.4363705338 -0.0273330303
C 2.9824875857 -1.5527686298 0.9819891774
C 3.9795108119 -1.1416356359 2.0731733415
C 1.3729617778 0.1674175356 -2.1541437923
N 0.0643670105 0.6693876370 -2.2372387285
H 2.2827365381 0.4420627598 0.4884991685
H 3.6287932318 -0.1123423342 -0.4946726653
H 3.3701883159 -2.4354305876 0.4507274948
H 2.0412772189 -1.8735722644 1.4537300446
H 4.9208879025 -0.8250652885 1.6020156653
C 2.4310834658 0.7839640402 -3.0157746800
C 4.2646831219 -2.2577580809 3.0833878169
H 3.3474684371 -2.5717484191 3.5970980290
H 4.9795880076 -1.9330847946 3.8481039512
H 2.1338781929 -1.7527725913 -1.6428322364
H 0.7677996330 -1.2315827812 -0.6677891275
S -0.6305012771 1.6673205377 -0.9683359799
O -1.5579416084 2.5522631390 -1.6873481706
O 0.4221272975 2.2001555717 -0.0924239994
C -1.5910286782 0.4976353067 -0.0088134671
C -2.6989133911 -0.1243748097 -0.5922606466
C -1.2489484223 0.2563368129 1.3194931580
C -3.4564833243 -1.0073647967 0.1684338732
H -2.9620641680 0.0852963782 -1.6237487349
C -2.0234387529 -0.6316418418 2.0686699602
H -0.3948372003 0.7631092681 1.7551382663
C -3.1320967741 -1.2763861337 1.5091566642
H -4.3179267462 -1.4950662305 -0.2816862170
H -1.7610702262 -0.8213857681 3.1063696711
C -3.9707152610 -2.2351824887 2.3200902246
H -4.0044251848 -3.2268332579 1.8526873423
H -3.5764658590 -2.3547334996 3.3335616185
H -5.0065366897 -1.8835755126 2.4022923099
H 4.6846875487 -3.1425626361 2.5890548858
H -0.1150388533 1.2069605205 -3.0858506642
H 3.5935917323 -0.2583276052 2.6012799143
H 2.8328891905 1.7139545420 -2.5790949878
H 2.0467216269 1.0446295750 -4.0126546494
H 3.2743702208 0.0981381591 -3.1548698436
40

C 1.7782506607 -1.4852854433 0.5682829353
C 3.3008850657 -1.6459716730 0.3071808131
C 4.0449292639 -0.3251144256 0.0793933596
C 5.5546724956 -0.5112083476 -0.1219125697
C 0.9974149577 -0.9607113773 -0.6030051940
N 0.7583007963 0.4232751075 -0.7177927010
H 3.4569727970 -2.3122222271 -0.5538046000
H 3.7370105572 -2.1631341419 1.1741670109
H 3.8690712570 0.3411051309 0.9360712365
H 3.6230581346 0.1939384249 -0.7919075244
H 5.9773872327 -1.0241495781 0.7539848205
C 0.8414074447 -1.7629266900 -1.8587946115
C 6.2993143424 0.8086344654 -0.3481883390
H 7.3740585188 0.6441598356 -0.4876666149
H 6.1730171568 1.4853879795 0.5056323320
H 1.3774834472 -2.4727961246 0.8396752168
H 1.6336359797 -0.8307309064 1.4325063696
S -0.2719649313 1.3011628882 0.3526278046
O -0.3412697518 2.6375130030 -0.2433685241
O 0.2282422441 1.0734246052 1.7086593286
C -1.8960851308 0.5503762064 0.2383423541
C -2.1861305875 -0.5892941449 0.9922437333
C -2.8365422494 1.0835594994 -0.6465032536
C -3.4320687940 -1.1973390323 0.8491209147
H -1.4563767486 -0.9763864185 1.6951050534
C -4.0784855277 0.4654847450 -0.7702598957
H -2.5977067844 1.9785105557 -1.2116619889
C -4.3961030280 -0.6824338182 -0.0288632181
H -3.6625651634 -2.0830401910 1.4362174882
H -4.8162621490 0.8838255296 -1.4507821118
C -5.7566573917 -1.3265025219 -0.1490242666
H -5.7193947170 -2.3924972420 0.0972861961
H -6.4748895375 -0.8577346473 0.5366696628
H -6.1611514879 -1.2239997148 -1.1614547006
H 5.9229650525 1.3283807778 -1.2379285823
H 0.5590877701 0.7501264735 -1.6615818028
H 5.7279281390 -1.1805799658 -0.9772305567
H -0.1430583339 -1.6044759324 -2.3207115409
H 0.9446013913 -2.8328267247 -1.6495519737
H 1.5977558886 -1.5141291356 -2.6247869066
40

C 2.7203933844 0.5148654063 -0.6789644058
C 2.3473570613 -0.3765265466 0.5329777144
C 3.4447409665 -1.3940293822 0.8750605493
C 3.1484472829 -2.2631766033 2.1088267862
C 1.6919950851 1.5431173752 -1.0515586560
N 0.6933195354 1.1730383328 -1.9584203083
H 1.4031220765 -0.8913453921 0.3172995686
H 2.1571724694 0.2641851558 1.4051121218
H 4.3874743884 -0.8533968662 1.0417290372
H 3.6191548346 -2.0470424205 0.0063810796

H 4.0474412894 -2.8465558516 2.3491166262
C 1.6496976879 2.8983913217 -0.4223014934
C 1.9647142686 -3.2234957356 1.9403241695
H 1.0232493859 -2.6883574823 1.7722366317
H 1.8363799570 -3.8475975007 2.8323984149
H 3.6677393692 1.0261594124 -0.4594779171
H 2.9232439544 -0.1428863302 -1.5402971399
S -1.0158894614 1.3077642797 -1.7230490749
O -1.5736908062 0.7101154724 -2.9394805796
O -1.2769370276 2.6939377058 -1.3350369440
C -1.4725624710 0.2662384028 -0.3349602287
C -1.4658216795 0.7923492201 0.9583408855
C -1.7865451245 -1.0775952969 -0.5590016514
C -1.7717219035 -0.0411047940 2.0331794683
H -1.2551192482 1.8454859731 1.1122056905
C -2.0911179352 -1.8941937862 0.5269039187
H -1.8179465355 -1.4629247699 -1.5730144930
C -2.0822745751 -1.3940402715 1.8389398538
H -1.7796439226 0.3697196093 3.0398242715
H -2.3520194151 -2.9355116017 0.3535097379
C -2.3892269177 -2.2974608343 3.0094338400
H -3.1852367835 -3.0098027558 2.7672684365
H -1.5060634009 -2.8835835959 3.2960567756
H -2.7013870887 -1.7238626554 3.8876760672
H 2.1209970151 -3.8950543087 1.0865164963
H 0.8687064804 0.3107597788 -2.4698111779
H 2.9701492956 -1.6102305673 2.9752045016
H 1.1820187711 3.6271690914 -1.0901952813
H 2.6680207629 3.2304361883 -0.1840834483
H 1.0805600088 2.9276304102 0.5216269913

TS 11 → 12

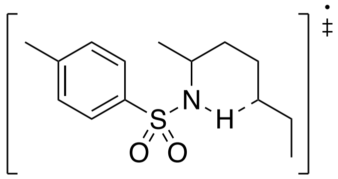

Name	E(B3LYP)	H(B3LYP)	E(RO-B2PLYP-D3)	H(RO-B2PLYP-D3)	NImag
tos_2Nheptan_15HAT_ts.conf000	-1150.00082912	-1149.645019	-1149.55014840500000	-1149.19433828500000	-1554.2589
tos_2Nheptan_15HAT_ts.conf008	-1150.00151574	-1149.644789	-1149.55051252440000	-1149.19378578440000	-1587.2063
tos_2Nheptan_15HAT_ts.conf026	-1149.99953084	-1149.642705	-1149.54948183320000	-1149.19265599320000	-1706.7263
tos_2Nheptan_15HAT_ts.conf021	-1150.00041791	-1149.643485	-1149.54939471160000	-1149.19246180160000	-1497.7941
tos_2Nheptan_15HAT_ts.conf035	-1149.99751181	-1149.640514	-1149.54888532310000	-1149.19188751310000	-1542.2226
tos_2Nheptan_15HAT_ts.conf033	-1149.99808489	-1149.641222	-1149.54823623480000	-1149.19137334480000	-1529.3218
tos_2Nheptan_15HAT_ts.conf029	-1149.99860079	-1149.641687	-1149.54773192190000	-1149.19081813190000	-1639.1198
tos_2Nheptan_15HAT_ts.conf040	-1149.99705246	-1149.640055	-1149.54620955200000	-1149.18921209200000	-1596.8328

40
-1149.19433828
C -3.6072784486 -0.1565812663 -1.0891861906
C -3.5169617452 1.2598049469 -0.5004458781
C -2.1283268295 1.8373381167 -0.6976892119
C -1.6470501434 2.8960111565 0.2693481326
C -2.6504560252 -1.1375550229 -0.3838705005
N -1.2680514766 -0.5862528370 -0.4681486403
H -3.3663941601 -0.1285812414 -2.1610569214
H -4.6266779607 -0.5496669781 -1.0001080866
H -3.7566666101 1.2298429110 0.5710757071
H -4.2697069689 1.9082286549 -0.9753472125
H -1.8989357643 2.0534321896 -1.7491757952
H -1.4335870583 0.7054338308 -0.5560180212
H -1.6207007961 2.4717699347 1.2808738502
H -2.4009468902 3.7022799935 0.2971136253
H -2.9735809729 -1.2293394694 0.6629614742
C -0.2824167731 3.4937454821 -0.0878793106
H 0.0055797356 4.2694165467 0.6302089022
H 0.5001990453 2.7272471282 -0.0826876309
S -0.3377925926 -0.8629395315 0.9203929242
O -0.4079340780 -2.3046804790 1.2088959437
O -0.6614549730 0.0902737526 1.9982068332
C 1.2990203727 -0.4618564405 0.3107146989
C 1.8068076965 -1.1190524586 -0.8141909484
C 2.0793688440 0.4479554077 1.0207769340
C 3.1034438154 -0.8417825263 -1.2324353944
H 1.1883173749 -1.8273696914 -1.3548773280
C 3.3807727699 0.7111291975 0.5882494187
H 1.6677160661 0.9378892052 1.8965795540
C 3.9114603100 0.0757369343 -0.5397220160
H 3.4998069021 -1.3468609438 -2.1102663200
H 3.9917411192 1.4211995142 1.1398961902
C 5.3170659737 0.3638903340 -1.0107577781
H 5.9149890183 -0.5540717664 -1.0616459193
H 5.3167575640 0.8029822964 -2.0162144414
H 5.8292028043 1.0616074225 -0.3414970576
H -0.2972182750 3.9506178348 -1.0851121755
C -2.6874078406 -2.5239111460 -1.0402619458
H -3.7097513447 -2.9203463831 -1.0201249630
H -2.3583749894 -2.4588781175 -2.0832802853
H -2.0320405440 -3.2149053121 -0.5062788150
40
-1149.19378578
C -3.1448571471 -0.3040145411 -1.6032641276
C -3.9076288419 0.5857687675 -0.6079769877
C -3.0493170998 1.7511633981 -0.1515168842
C -3.3346042389 2.3743033140 1.1977492005
C -1.8777890936 -0.9123799416 -0.9703427850
N -1.0272199165 0.1979908972 -0.4858598549
H -2.8609154715 0.2838755898 -2.4869619871
H -3.7823667027 -1.1242757988 -1.9532904076
H -4.2126579731 -0.0136767545 0.2607994874
H -4.8330197803 0.9558837580 -1.0758210725
H -2.8699018871 2.4859950788 -0.9469490728
H -1.8779397247 1.1545627749 -0.1569249431
H -3.2271669451 1.6128182044 1.9817550571
H -4.3984422531 2.6706627549 1.2141298274
H -2.1807680500 -1.5540595110 -0.1319429336
C -2.4581556714 3.5874137671 1.5241774463
H -1.4054813026 3.2959391240 1.5766129443
H -2.5668532736 4.3694703892 0.7621767976
S -0.1160299763 -0.0918231041 0.9180822561
O -0.5755525020 -1.2930761604 1.6364693846
O -0.0389060434 1.1992722964 1.6165557448
C 1.5089006123 -0.4542330731 0.2470133296
C 2.0578426659 -1.7225010268 0.4252671768
C 2.2198527011 0.5506489708 -0.4161973287
C 3.3343058788 -1.9863895512 -0.0746123056
H 1.4922816259 -2.4818354536 0.9547181500
C 3.4890062853 0.2688797632 -0.9089102869
H 1.7819210552 1.5357311025 -0.5379943786
C 4.0673180775 -1.0014984484 -0.7466983101
H 3.7671088168 -2.9739471107 0.0647686288
H 4.0449378053 1.0470509655 -1.4267554968
C 5.4545012860 -1.2833969006 -1.2727541345
H 6.2143563479 -0.7472550942 -0.6897564330
H 5.6925241958 -2.3503300800 -1.2252616370
H 5.5605312418 -0.9584354376 -2.3143260913
H -2.7395342067 4.0217697968 2.4898165296
C -1.0996145414 -1.7600489873 -1.9886032353
H -0.7550730473 -1.1379799461 -2.8213415388
H -0.2258146598 -2.2294881613 -1.5277909805
H -1.7439660609 -2.5541736184 -2.3837352668
40
-1149.19265599
C -3.0906530379 -0.2236177540 -1.6291615850
C -3.8503347258 0.7372441178 -0.6965318810
C -2.9651316428 1.9006078984 -0.2854924817
C -3.2008053149 2.6448581870 1.0160688145
C -1.8387375618 -0.8152028052 -0.9490921144
N -0.9644919905 0.3122237950 -0.5564663145
H -2.7857598936 0.3039457490 -2.5433375196
H -3.7406147200 -1.0514312372 -1.9359249138
H -4.1915969265 0.1843426043 0.1865460589
H -4.7538220512 1.1072292834 -1.2060685931
H -2.7554257867 2.5790555412 -1.1217153004
H -1.8114216735 1.2820881361 -0.2602313513
H -4.0761103747 3.3030800074 0.8745762571
H -2.3484118295 3.3141765162 1.1841180250

H -2.1547498902 -1.3818727813 -0.0624066790
C -3.4176368017 1.7816671212 2.2647335745
H -4.3253758881 1.1720587795 2.1931308368
H -2.5683138157 1.1141542248 2.4404941071
S -0.0399404619 0.1301724663 0.8554722009
O -0.5277273904 -0.9763453975 1.6984630485
O 0.0838068647 1.4822761250 1.4183413654
C 1.5633492815 -0.3511865260 0.2080655731
C 2.0675738203 -1.6183372570 0.4947973782
C 2.3023075822 0.5630893335 -0.5487227833
C 3.3274819639 -1.9732854378 0.0100118557
H 1.4809874743 -2.3053638171 1.0953921025
C 3.5546472395 0.1910339870 -1.0248355280
H 1.8991755247 1.5491800949 -0.7539482884
C 4.0881685511 -1.0801912497 -0.7537688181
H 3.7256062717 -2.9599677360 0.2336595224
H 4.1324744763 0.8983458937 -1.6152266150
C 5.4595052450 -1.4589138138 -1.2601967186
H 5.5852812716 -1.1881322132 -2.3147642742
H 6.2452518128 -0.9376753662 -0.6981040288
H 5.6409318759 -2.5335526233 -1.1620594098
H -3.5237856969 2.4210980002 3.1478344640
C -1.0842868168 -1.7575556264 -1.8993770549
H -0.7291978787 -1.2107248687 -2.7791857997
H -0.2193027442 -2.2095461548 -1.4056342566
H -1.7480837285 -2.5650894730 -2.2297297418
40
-1149.19256729
C -3.0360324930 -0.1204111466 -1.6143717542
C -3.7646349033 0.7880115601 -0.6072529667
C -2.8462995092 1.8896919713 -0.1066171002
C -3.0467514541 2.4465291399 1.2866216454
C -1.8004685613 -0.7973406866 -0.9871746595
N -0.8954723167 0.2679162730 -0.5018887401
H -2.7195186482 0.4683262086 -2.4862953935
H -3.7099843752 -0.9031519108 -1.9816725596
H -4.1141735130 0.1839446411 0.2413340419
H -4.6606453593 1.2146556548 -1.0827751459
H -2.6499115112 2.6602122439 -0.8631047611
H -1.7066744864 1.2486856417 -0.1602737754
H -2.1649022140 3.0300808907 1.5739492097
H -3.1149605663 1.6195218789 2.0047900233
H -2.1327976628 -1.4272621864 -0.1508429581
C -4.3070261880 3.3271707267 1.3960360283
H -4.4106294251 3.7246716344 2.4120115114
H -4.2576980998 4.1780434090 0.7060102326
S -0.0074964260 -0.0531754544 0.9112993788
O -0.5263369982 -1.2284877014 1.6328179817
O 0.1194406582 1.2395693312 1.5985413138
C 1.6035190509 -0.4932204960 0.2538123857
C 2.0821283142 -1.7915328288 0.4191760872
C 2.3735900706 0.4806577663 -0.3891732863
C 3.3470535439 -2.1170370762 -0.0733451909
H 1.4720850836 -2.5262221534 0.9338389016
C 3.6304280233 0.1376190176 -0.8749013574
H 1.9905121467 1.4896040817 -0.5000071827
C 4.1381695024 -1.1639624981 -0.7253718260
H 3.7249865527 -3.1282218845 0.0560244115
H 4.2322058914 0.8916238905 -1.3770128410
C 5.5139241287 -1.5128800900 -1.2411983893
H 6.2955377187 -1.0499525302 -0.6249961229
H 5.6833852941 -2.5938812868 -1.2328444021
H 5.6591164684 -1.1545191873 -2.2668421136
H -5.2158091731 2.7597567347 1.1640593011
C -1.0677257672 -1.6772257414 -2.0116590545
H -0.6985911008 -1.0695262124 -2.8444871271
H -0.2141603557 -2.1872065828 -1.5558256760
H -1.7495801135 -2.4399076558 -2.4057560025
40
-1149.19246180
C -3.4882948788 -0.0259290807 -1.0850415336
C -3.2258712781 1.4098458360 -0.5983981212
C -1.7739811149 1.7951071419 -0.8179688828
C -1.1350428975 2.8139650150 0.1009958209
C -2.6477080501 -1.0625646882 -0.3134447786
N -1.2114363559 -0.6950031557 -0.4594997905
H -3.2552670489 -0.1031346075 -2.1561839237
H -4.5467875420 -0.2857219318 -0.9676849005
H -3.4707356156 1.4853609928 0.4698040048
H -3.8964027037 2.1034636190 -1.1283056338
H -1.5215489390 1.9317285109 -1.8771545273
H -1.2289202742 0.5931924427 -0.6196185066
H -0.0529730478 2.8413111397 -0.0816357563
H -1.2699802571 2.4962525686 1.1418198640
H -2.9551999020 -1.0260340787 0.7418569084
C -1.7109494299 4.2316268113 -0.0890772602
H -1.5718020026 4.5829735499 -1.1184507897
H -2.7836188395 4.2601514949 0.1332363477
S -0.2913025798 -0.9821534440 0.9355734300
O -0.3560723760 -2.4276956308 1.2045207615
O -0.6283676022 -0.0418932754 2.0206565045
C 1.3429699176 -0.5641498558 0.3323527711
C 2.0556177505 0.4606961539 0.9508011160
C 1.9057148853 -1.3031474887 -0.7124556086
C 3.3455127220 0.7557113271 0.5056392814
H 1.6015412219 1.0117486319 1.7672301587
C 3.1902979437 -0.9927213983 -1.1450737566
H 1.3397331974 -2.1022460635 -1.1793171687
C 3.9314429309 0.0381883184 -0.5434443518
H 3.9045685229 1.5555663835 0.9848872503
H 3.6298012345 -1.5605698431 -1.9618148034
C 5.3338312382 0.3459870573 -1.0117112071
H 5.3839741398 0.4168423620 -2.1043497817
H 5.6986609496 1.2891673130 -0.5938136569
H 6.0330631434 -0.4437429502 -0.7080679872
H -1.2121835732 4.9425894953 0.5794357370
C -2.8702951149 -2.4814595856 -0.8536859213
H -2.5638098336 -2.5402605697 -1.9037702084
H -2.2871763616 -3.2035254156 -0.2783226770
H -3.9320610634 -2.7465701472 -0.7828742779
40
-1149.19188751
C -2.6688416052 0.3533832190 1.7061849404
C -2.1451065350 1.7363509180 1.2802450563
C -2.1318221575 1.8654200014 -0.2329896559
C -1.1747672183 2.8404612756 -0.8855159939
C -1.7945687349 -0.8005030170 1.1704204958
N -1.7173972788 -0.6597772492 -0.2965880815
H -3.6946219952 0.2181406987 1.3378290048
H -2.7067160737 0.2764668153 2.7989630700
H -1.1295319956 1.8841028819 1.6742954428
H -2.7743006646 2.5179851268 1.7324651035
H -3.1395351026 1.8951914399 -0.6659362073
H -1.8199686649 0.6319559700 -0.5384667113
H -1.1178435266 2.6267826509 -1.9603160453
H -0.1658235872 2.6930036348 -0.4787574992
H -0.7979554851 -0.7106555771 1.6318473725
C -1.5935848929 4.3102857941 -0.6840551626
H -0.8902142127 4.9846424993 -1.1856415591
H -2.5910515898 4.4967138541 -1.0987736677
S -0.4002787580 -1.2850448182 -1.1362865731
O -0.4623611225 -0.6730777260 -2.4664319605
O -0.4421631185 -2.7443637817 -0.9605660257
C 1.1265245590 -0.7220814895 -0.3626441358
C 1.6930433045 -1.4553212590 0.6825909560
C 1.7469323566 0.4383674509 -0.8323959835
C 2.8736606589 -1.0049438184 1.2726290158
H 1.2318140976 -2.3835537910 1.0028705421
C 2.9280844586 0.8719782470 -0.2343843027
H 1.3209396240 0.9696597057 -1.6765330068
C 3.5053341533 0.1655664896 0.8314430262
H 3.3182885475 -1.5807580371 2.0807505712
H 3.4186803245 1.7666665893 -0.6106018598
C 4.7709497376 0.6601690781 1.4903062896
H 4.5460952123 1.3991847652 2.2709389344
H 5.3223050125 -0.1577551946 1.9648319658
H 5.4357944247 1.1449413685 0.7676712392
H -1.6159561503 4.5785196343 0.3783383965
C -2.3979351463 -2.1649932295 1.5402998973
H -1.7807901606 -2.9805951370 1.1577705646

H -2.4750360634 -2.2536239730 2.6306377611
H -3.3981949538 -2.2639242857 1.1063866870
40
-1149.19137334
C -2.9959320315 -0.9136061087 0.0604928407
C -3.7852628710 0.2432258452 -0.5847298789
C -3.0000073870 1.5499389236 -0.5533521292
C -3.0444211025 2.3862420642 0.7131421443
C -1.6127189900 -1.1243807696 -0.6090311786
N -0.8448269408 0.1417767105 -0.6574083958
H -3.5701106330 -1.8464103641 0.0000284564
H -2.8296714969 -0.7037690324 1.1225361341
H -4.7353178279 0.3630073223 -0.0421709697
H -4.0475477003 -0.0145207011 -1.6171967323
H -3.0914631824 2.1520131104 -1.4644228052
H -1.7815716084 1.0710489120 -0.6793268640
H -2.1802510274 3.0595705022 0.7320100732
H -2.9425710020 1.7382768309 1.5928528674
H -1.0618535050 -1.8394521907 0.0157559270
C -4.3391610196 3.2127297405 0.8254587274
H -5.2295859115 2.5730861744 0.8393916561
H -4.3384988864 3.8034584701 1.7484417281
S 0.1191323890 0.4957854700 0.6950144150
O -0.3542236749 -0.1717081710 1.9222982319
O 0.3031497121 1.9526164850 0.6756642731
C 1.6624644673 -0.2890920667 0.2229093643
C 2.4006848064 0.2333238034 -0.8440747560
C 2.1193794554 -1.3946103662 0.9378852881
C 3.6041531994 -0.3690111109 -1.1928535826
H 2.0334053212 1.0993466198 -1.3845885026
C 3.3302364737 -1.9857515445 0.5733590221
H 1.5365411616 -1.7725869518 1.7709948378
C 4.0890485254 -1.4860164942 -0.4914301734
H 4.1816289879 0.0334220112 -2.0218849647
H 3.6913903298 -2.8471118987 1.1298741792
C 5.4089709260 -2.1139731555 -0.8707326653
H 5.5444976554 -3.0863945961 -0.3876018762
H 5.4859663629 -2.2593214356 -1.9543556425
H 6.2499291992 -1.4751492203 -0.5712303301
H -4.4399419550 3.9070036120 -0.0173127891
C -1.7019205884 -1.6825583958 -2.0336575767
H -2.2036695916 -0.9821287537 -2.7096856235
H -0.6991885548 -1.8650425969 -2.4312845072
H -2.2589413483 -2.6269745996 -2.0428788833
40
-1149.19081813
C -3.4828570906 0.0087031170 -1.1680337496
C -3.1687974232 1.4845813322 -0.8698441276
C -1.6961387767 1.7741734052 -1.0902376069
C -1.0291993479 2.9696427008 -0.4342409029
C -2.7032554559 -0.9540033731 -0.2496832134
N -1.2528723621 -0.6772956775 -0.4321091724
H -3.2356796568 -0.2217805473 -2.2135034829
H -4.5536641709 -0.1878172200 -1.0399297872
H -3.4523814399 1.7127826291 0.1644508670
H -3.7851581141 2.1306167561 -1.5156004968
H -1.4038384211 1.6621391526 -2.1417906297
H -1.2088977054 0.6068082980 -0.6603775458
H -1.3032048030 3.8676315485 -1.0154462224
H 0.0577281353 2.8689698932 -0.5566841927
H -3.0088746228 -0.7577331621 0.7890494238
C -1.3604536171 3.2085720005 1.0443985053
H -0.7655612922 4.0423923484 1.4338880886
H -2.4150692277 3.4693579759 1.1870736887
S -0.3019709910 -0.9736849722 0.9394139602
O -0.3277969184 -2.4254053599 1.1812661185
O -0.6300635481 -0.0659512375 2.0551328592
C 1.3086344858 -0.5111892599 0.3050603638
C 1.8679350499 -1.2336766537 -0.7531888766
C 2.0066148864 0.5323726160 0.9089153258
C 3.1330446741 -0.8868924179 -1.2148190151
H 1.3145625617 -2.0492542217 -1.2064553641
C 3.2771183839 0.8639276109 0.4344536315
H 1.5581828261 1.0684655538 1.7383690258
C 3.8584481373 0.1641936853 -0.6291808142
H 3.5698292917 -1.4421793913 -2.0415974739
H 3.8248669084 1.6780524916 0.9025759866
C 5.2404141004 0.5118058267 -1.1292190361
H 5.5909001015 1.4611439457 -0.7130414624
H 5.9670543380 -0.2616549533 -0.8490754566
H 5.2616788236 0.5918042501 -2.2221923251
H -1.1458641319 2.3220532579 1.6492999065
C -3.0051590155 -2.4186746418 -0.5984936198
H -4.0811150470 -2.6079971102 -0.5040435600
H -2.7008628580 -2.6326773717 -1.6287890019
H -2.4663238248 -3.0933752931 0.0693910551
40
-1149.18979508
C -1.5981942519 1.2429991590 1.5992807666
C -2.5863978339 1.9907517623 0.6833375466
C -2.4163089582 1.5984329590 -0.7820601795
C -1.3932749930 2.3551672266 -1.6065746928
C -1.7428608328 -0.2972294584 1.4961599444
N -1.7120491455 -0.7162067388 0.0788499418
H -1.7434074470 1.5448696902 2.6435862812
H -0.5708117516 1.5164625217 1.3278528019
H -2.4272551617 3.0732133128 0.8013158404
H -3.6130980919 1.7884209942 1.0083281816
H -3.3618098255 1.4514209039 -1.3156790509
H -2.0262067380 0.3567295214 -0.6264229663
H -0.4109801243 2.3147913402 -1.1160754922
H -1.6791633797 3.4209752355 -1.6058162069
H -0.8863427253 -0.7387025692 2.0213519899
C -1.2841307735 1.8588892470 -3.0534045835
H -2.2443013502 1.9617167551 -3.5733339828
H -0.5411498796 2.4385506043 -3.6128878369
S -0.3840678856 -1.5534994907 -0.5084435827
O -0.4942756628 -1.5013719796 -1.9696701095
O -0.3691915680 -2.8309177720 0.2186955109
C 1.1411971222 -0.7055085857 -0.0577177742
C 1.6814375511 0.2531284687 -0.9192903326
C 1.7815434107 -1.0188832784 1.1437534918
C 2.8534893989 0.9139774730 -0.5582454277
H 1.2004960065 0.4551879198 -1.8700916444
C 2.9518057157 -0.3449723741 1.4914479416
H 1.3808938392 -1.8011981581 1.7795344635
C 3.5050678073 0.6306735895 0.6514199269
H 3.2761810265 1.6554829548 -1.2321867502
H 3.4497207327 -0.5904615441 2.4264459034
C 4.7924254283 1.3303046129 1.0171881215
H 5.6567553604 0.8209692155 0.5708971408
H 4.8039912271 2.3640812453 0.6559033890
H 4.9470486359 1.3458511635 2.1007760800
H -0.9992444191 0.8022354208 -3.0909499316
C -3.0251642288 -0.8375763701 2.1403214414
H -3.0768938552 -0.5510289690 3.1978197664
H -3.9188577634 -0.4583252308 1.6339701164
H -3.0400994216 -1.9286603474 2.0702454069

TS 11 → 13

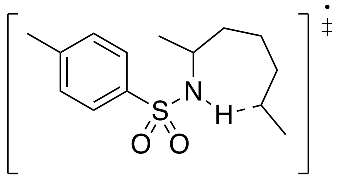

Name	E(B3LYP)	H(B3LYP)	E(RO-B2PLYP-D3)	H(RO-B2PLYP-D3)	NImag
tos_2Nheptan_16HAT_ts.conf003	-1149.999728	-1149.643057	-1149.549332	-1149.192661	-1554.2589
tos_2Nheptan_16HAT_ts.conf005	-1149.997328	-1149.640557	-1149.549388	-1149.192617	-1587.2063
tos_2Nheptan_16HAT_ts.conf004	-1149.997614	-1149.640860	-1149.547562	-1149.190808	-1706.7263
tos_2Nheptan_16HAT_ts.conf007	-1149.996505	-1149.639829	-1149.546652	-1149.189976	-1497.7941
tos_2Nheptan_16HAT_ts.conf013	-1149.996411	-1149.639701	-1149.546626	-1149.189916	-1542.2226
tos_2Nheptan_16HAT_ts.conf032	-1149.992941	-1149.636253	-1149.546050	-1149.189362	-1529.3218
tos_2Nheptan_16HAT_ts.conf041	-1149.992262	-1149.635258	-1149.545931	-1149.188927	-1639.1198
tos_2Nheptan_16HAT_ts.conf027	-1149.994687	-1149.637733	-1149.545618	-1149.188664	-1596.8328
tos_2Nheptan_16HAT_ts.conf025	-1149.994874	-1149.637876	-1149.545638	-1149.188640	-1723.8393
tos_2Nheptan_16HAT_ts.conf020	-1149.995249	-1149.638519	-1149.544872	-1149.188141	-1639.5118

40
-1149.19266100
C 2.7744235355 1.9170877673 -0.8242221278
C 4.1094377677 1.2033569263 -0.5382723167
C 4.1801681782 -0.2631939647 -0.9940316724
C 3.2767841777 -1.1948798660 -0.2122234950
C 1.5815000905 1.5938812075 0.0980983115
N 1.0821705461 0.2261533305 -0.1874817999
H 4.3363383524 1.2679866545 0.5359394291
H 4.9045829943 1.7605292683 -1.0500510113
H 5.2191056958 -0.6208625405 -0.9038806607
H 3.9307288240 -0.3233334443 -2.0631798912
H 2.1321034350 -0.5805203356 -0.2219469391
H 1.9066568950 1.6745316610 1.1448278999
C 3.0258785763 -2.5698413780 -0.7873422780
H 2.6774637989 -2.5097197944 -1.8254090227
H 2.2799920212 -3.1097451440 -0.1993603901
H 2.9463691041 2.9970802005 -0.7314649021
H 2.4731052660 1.7410349347 -1.8664339323
H 3.5028120692 -1.2094074655 0.8602714695
S 0.0891080389 -0.4734566140 1.0113896525
O 0.1425283249 0.2743418069 2.2792255826
O 0.3908141903 -1.9121746850 0.9974965310
C -1.5527000231 -0.2512440236 0.3176800115
C -2.5253587014 0.4060048623 1.0677766936
C -1.8558294688 -0.7936220572 -0.9350039214
C -3.8163583156 0.5265478475 0.5503740329
H -2.2687725847 0.8119349511 2.0405094147
C -3.1459909421 -0.6608552267 -1.4358276452
H -1.0885200671 -1.3054708658 -1.5060141199
C -4.1470901752 -0.0011104433 -0.7028830229
H -4.5779825821 1.0381700677 1.1335883312
H -3.3844454316 -1.0768765281 -2.4119939348
C -5.5466298409 0.1199525176 -1.2571190338
H -6.1694811280 0.7700371727 -0.6353429771
H -5.5393813411 0.5300354954 -2.2739265072
H -6.0371986220 -0.8603631666 -1.3095546503
H 3.9573083473 -3.1576205317 -0.7880036749
C 0.4484220328 2.6087505961 -0.1321889289
H 0.8248044975 3.6236047294 0.0405254722
H 0.0759249381 2.5435393181 -1.1601514875
H -0.3864217910 2.4411484054 0.5520234402
40
-1149.19261700
C 2.5464476989 1.5058986186 -1.3301639048
C 2.4228412802 2.4479440088 -0.1165043272
C 2.8975368727 1.8496895336 1.2189367761
C 2.0068935682 0.7401211226 1.7405469761
C 1.4527624336 0.4356978266 -1.5340004149
N 1.5404368024 -0.5840888078 -0.4638189216
H 1.3838400217 2.7963173902 -0.0206923695
H 3.0236737078 3.3426288634 -0.3233126472
H 2.9532849023 2.6482431234 1.9765306460
H 3.9224697913 1.4712609571 1.0994010287
H 1.7947504518 0.0035001943 0.6924583698
H 0.4747111469 0.9432972367 -1.5191691078
C 2.5754996834 -0.1854026856 2.7922482610
H 2.7403944657 0.3596878645 3.7350028008
H 3.5409817015 -0.5962917928 2.4760964267
H 2.5336485282 2.1203595141 -2.2391798055
H 3.5243998232 1.0051313014 -1.3112454923
H 0.9931473439 1.0909407488 1.9689848334
S 0.2165380179 -1.5977901207 -0.1745411481
O 0.4789714944 -2.2126962534 1.1308451262
O 0.0275786127 -2.4250969393 -1.3740923937
C -1.2692245672 -0.5940519715 0.0014705950
C -1.6599622950 -0.1607498429 1.2711599025
C -2.0246306704 -0.2602337116 -1.1249966298
C -2.8043625806 0.6223785802 1.4040153647
H -1.0856228332 -0.4599352764 2.1413430879
C -3.1653820645 0.5272808527 -0.9745318121
H -1.7350816814 -0.6354271546 -2.1008369461
C -3.5732127701 0.9812274672 0.2870781395
H -3.1117532324 0.9533509609 2.3932525163
H -3.7535885135 0.7855070008 -1.8518569751
C -4.8300395613 1.8031687366 0.4462644175
H -5.0584008953 2.3677615094 -0.4634213536
H -5.6951306207 1.1608497532 0.6577786201
H -4.7429837846 2.5128688077 1.2756656397
H 1.8999848753 -1.0213490299 2.9931911167
C 1.6450269207 -0.2379147059 -2.9053978199
H 0.8929124383 -1.0067042986 -3.0871832401
H 1.5814221088 0.5195106659 -3.6957928122
H 2.6302601162 -0.7133836859 -2.9538469984
40
-1149.19080800
C 3.3931489332 1.5571446623 -0.1707370081
C 4.0380980708 0.2405017030 -0.6444198738
C 3.2654079711 -0.4843514428 -1.7582614012
C 1.9277670571 -1.0380480026 -1.3154741883

C 2.2548887096 1.4702555095 0.8652674173
N 1.0327656854 0.8705788784 0.2505531151
H 4.1780702182 -0.4337488951 0.2125167107
H 5.0435757816 0.4696707576 -1.0194603723
H 3.8793712181 -1.3131822470 -2.1477365847
H 3.1184978119 0.2062273154 -2.6012723919
H 1.3998079620 -0.0573209415 -0.5900331792
H 2.6090818807 0.8582192977 1.7079251086
C 0.9324096471 -1.3773917569 -2.4020489297
H 0.7296709712 -0.5119480480 -3.0441428349
H -0.0177878047 -1.7318194833 -1.9921395110
H 4.1730274529 2.1698428670 0.2985297017
H 3.0370360586 2.1297255816 -1.0393006678
H 2.0140359520 -1.8043207551 -0.5382861279
S 0.0470733139 0.0446809518 1.3682649586
O -0.3162797291 0.9977765472 2.4287460216
O 0.6235194883 -1.2557689232 1.7547503015
C -1.3980664115 -0.2760981974 0.3574345390
C -2.0577215165 0.7797005881 -0.2782074446
C -1.8938733627 -1.5765838619 0.2865607029
C -3.2144079626 0.5161213736 -1.0044624044
H -1.6597054597 1.7866511093 -0.2131979671
C -3.0587138369 -1.8215528285 -0.4426930151
H -1.3674739968 -2.3786098152 0.7930200516
C -3.7356046500 -0.7850155009 -1.0963808322
H -3.7252390204 1.3333510895 -1.5083076149
H -3.4466151277 -2.8354538950 -0.5022539905
C -5.0068710927 -1.0485213218 -1.8677706677
H -4.9982349067 -0.5409258169 -2.8390244787
H -5.1544524989 -2.1181767765 -2.0451295030
H -5.8835369153 -0.6799487365 -1.3194998341
H 1.3326291556 -2.1746552990 -3.0484166325
C 1.9111242853 2.8798321756 1.3759505227
H 1.5776682927 3.5102823622 0.5438712755
H 1.1207697005 2.8428254918 2.1261820674
H 2.8023542118 3.3370132346 1.8225420366
40
-1149.18997600
C 2.5384112608 1.8411114177 0.2056418628
C 3.8948572309 1.1815907005 -0.1108779381
C 4.0595212275 -0.2610142648 0.4138219417
C 3.2074112517 -1.3024145800 -0.2931990604
C 1.2974521708 1.3855942730 -0.6047182014
N 0.9235705400 -0.0391384684 -0.4427508683
H 4.6732987977 1.7963305189 0.3586493437
H 4.0998492428 1.2188987490 -1.1890880596
H 3.8267679079 -0.2717172739 1.4861082893
H 5.1186115994 -0.5511687458 0.3227644184
H 2.0427592851 -0.7667130972 -0.4107347143
H 0.4534577869 1.9746487248 -0.2195908458
C 3.6147983377 -1.7104053393 -1.6946391585
H 3.7877188032 -0.8476116363 -2.3477000777
H 2.8549820071 -2.3447599539 -2.1630877616
H 2.3144246412 1.7170213484 1.2706648595
H 2.6345848795 2.9212805334 0.0304055380
H 2.9502948427 -2.1565397062 0.3393548488
S 0.0746245635 -0.4552672367 0.9703105068
O 0.2936396727 0.4930818416 2.0773101714
O 0.3204221778 -1.8887624644 1.1724812363
C -1.6157719445 -0.2313304503 0.4076937838
C -2.1487107238 -1.1140554045 -0.5373152021
C -2.3850516781 0.8039081640 0.9354792168
C -3.4641558138 -0.9456577689 -0.9541268299
H -1.5380828290 -1.9197905105 -0.9309237736
C -3.7040025031 0.9575680471 0.5047638989
H -1.9550974352 1.4658886091 1.6794810513
C -4.2629361235 0.0907379651 -0.4414193016
H -3.8830909242 -1.6300023569 -1.6882474487
H -4.3082761501 1.7624493513 0.9159455835
C -5.6959336457 0.2462439085 -0.8916218552
H -6.1200070341 1.1986647469 -0.5594152825
H -5.7790787886 0.2001025394 -1.9836199363
H -6.3257835939 -0.5565026212 -0.4869523627
H 4.5521468863 -2.2865473838 -1.6619836262
C 1.4174094076 1.6638208722 -2.1080644998
H 2.2051822023 1.0593571926 -2.5694617198
H 0.4769129581 1.4254751685 -2.6132491932
H 1.6527954863 2.7199070961 -2.2847049823
40
-1149.18991600
C 2.7639365404 1.7993062594 -0.3488949961
C 4.0719568332 1.0915675087 0.0506587395
C 4.0520829953 -0.4480911214 0.0235729351
C 3.1799321056 -1.0756740676 1.0930798926
C 1.5636495182 1.6024569547 0.6058871993
N 0.9789642785 0.2364876042 0.6329262847
H 4.3744439715 1.4269473598 1.0546624171
H 4.8590452201 1.4368476618 -0.6318598907
H 5.0827941891 -0.8163024694 0.1569177558
H 3.7292838685 -0.8035239369 -0.9648459596
H 2.0049673218 -0.5693135844 0.8962601484
H 1.9286147206 1.7268038065 1.6356190020
C 3.0262686998 -2.5796885141 1.0712654446
H 3.9998647598 -3.0592338925 1.2592403874
H 2.6535003865 -2.9252761358 0.1035410284
H 2.9698471926 2.8779428285 -0.3820247354
H 2.4661080929 1.5084386178 -1.3615693637
H 3.3921320726 -0.6765977137 2.0942248269
S 0.2000431566 -0.3898593426 -0.7393935634
O 0.5318091954 -1.8220157107 -0.7674464247
O 0.3955312474 0.4236637164 -1.9533093079
C -1.5254796817 -0.2279711016 -0.2653908189
C -2.3922379548 0.4963021869 -1.0804615061
C -1.9938002904 -0.8796642587 0.8801429333
C -3.7433455052 0.5736669665 -0.7373817894
H -2.0088258238 0.9860985894 -1.9689472547
C -3.3417224446 -0.7899166698 1.2075690521
H -1.3074506813 -1.4424229989 1.5038307024
C -4.2387476019 -0.0639069949 0.4054493986
H -4.4221404246 1.1379370914 -1.3721012950
H -3.7083663532 -1.2921103596 2.0998867167
C -5.7024900996 0.0101780911 0.7693931994
H -5.8397557392 0.3267654888 1.8101100087
H -6.2401132993 0.7152834499 0.1283147678
H -6.1858195558 -0.9696348399 0.6653543218
H 2.3329502381 -2.9245773596 1.8454695921
C 0.4711950060 2.6608201508 0.3821882420
H 0.8814283686 3.6538687393 0.5972977687
H 0.1180895648 2.6530925191 -0.6523294106
H -0.3799470964 2.4886204536 1.0480296690
40
-1149.18936200
C 1.3028879523 0.9872661725 -1.7014740302
C 2.1624795771 2.0691851918 -1.0167099689
C 1.8233392547 2.3288070370 0.4668032056
C 2.1821455739 1.1938844484 1.4130495021
C 1.6317463809 -0.5011941984 -1.4172270710
N 1.5214922711 -0.8993845973 0.0031485135
H 2.0049406154 3.0083326916 -1.5620057067
H 3.2297314507 1.8380544341 -1.1277456100
H 0.7492276027 2.5422758830 0.5444725497
H 2.3451504757 3.2423529992 0.7936883085
H 1.8435892256 0.1191149521 0.8038303189
H 0.8974856614 -1.0972440327 -1.9742534266
C 3.6468890958 0.9948173327 1.7477896534
H 4.2744352993 0.9533415442 0.8504363420
H 3.8008930219 0.0696100194 2.3120360367
H 0.2473121309 1.1702854828 -1.4649157475
H 1.3971636001 1.1123812451 -2.7883154433
H 1.5426134250 1.1448171267 2.3002204415
S 0.1102500417 -1.6241993363 0.5604897707
O 0.2568208907 -1.6890656008 2.0168771180
O -0.0640015524 -2.8335791189 -0.2573002547
C -1.3119980162 -0.5716100975 0.2244242603
C -1.6672898432 0.4202162249 1.1424774977
C -2.0458648056 -0.7477752401 -0.9502276885
C -2.7515390139 1.2497677257 0.8660951390
H -1.1124242639 0.5199054486 2.0695439232
C -3.1281097635 0.0919473173 -1.2116878959
H -1.7818887972 -1.5467833483 -1.6348442342

C -3.4971033038 1.1024431173 -0.3134456225
H -3.0311830529 2.0186857673 1.5825186626
H -3.7011672765 -0.0465510578 -2.1254022168
C -4.6910916876 1.9856414845 -0.5872295364
H -4.8897620354 2.0708744104 -1.6603797703
H -5.5967131081 1.5781991047 -0.1186702376
H -4.5447814470 2.9940929694 -0.1860715067
H 4.0129176372 1.8289482104 2.3654454715
C 3.0264781834 -0.9117832547 -1.9089858823
H 3.8177138278 -0.4061468661 -1.3456450687
H 3.1624856850 -1.9889865334 -1.7811080415
H 3.1490640406 -0.6643281777 -2.9702924468
40
-1149.18892700
C 2.7006167158 0.8717956783 -1.4268152407
C 2.4010369001 2.2428433317 -0.7820572438
C 1.6154588482 2.2209681614 0.5480446771
C 2.0351056573 1.1312255769 1.5173031438
C 1.5634350960 -0.1741998155 -1.4430741089
N 1.5368137153 -0.8352911914 -0.1166130125
H 1.8381872851 2.8612512275 -1.4932486673
H 3.3604411230 2.7556098429 -0.6365477153
H 0.5462365630 2.0934286326 0.3367996530
H 1.7085987726 3.2074579369 1.0300883517
H 1.8440959213 0.0506448569 0.8118701282
H 0.6090156575 0.3319802886 -1.6593101476
C 3.4648397810 1.0994248251 2.0123670438
H 3.6585753491 0.1958921991 2.5993861883
H 3.6607060088 1.9657524489 2.6634538141
H 3.0047568270 1.0506762661 -2.4651574962
H 3.5618819831 0.3980658511 -0.9391197104
H 1.3108456890 0.9741999054 2.3229039896
S 0.1458383958 -1.6424115874 0.3981302576
O 0.3267551205 -1.8446540238 1.8383965350
O -0.0518878107 -2.7794334623 -0.5123163771
C -1.2823414484 -0.5647125914 0.1868368992
C -1.6881925890 0.2551348067 1.2431640107
C -1.9801491695 -0.5591645565 -1.0232340343
C -2.7883196129 1.0932353111 1.0744341558
H -1.1609526526 0.2114016279 2.1902361162
C -3.0772967152 0.2875889262 -1.1761519397
H -1.6807340746 -1.2293325112 -1.8220459190
C -3.4984568019 1.1257219659 -0.1348670955
H -3.1077922844 1.7260119603 1.8991415557
H -3.6208155737 0.2901097701 -2.1179338157
C -4.7098004574 2.0130609797 -0.2954822032
H -4.8776233196 2.2772938890 -1.3444996099
H -5.6165401406 1.5082723360 0.0632606382
H -4.6069049826 2.9403297526 0.2779026482
H 4.1924051594 1.1374232564 1.1948332272
C 1.8292953306 -1.2281102755 -2.5324067036
H 1.8243892717 -0.7452742917 -3.5170391385
H 2.8089434435 -1.6912006668 -2.3734344355
H 1.0764484389 -2.0179982280 -2.5174861365
40
-1149.18866400
C 2.8379035139 1.5398068839 -1.0780228704
C 4.1369338466 1.0737954275 -0.3842622655
C 4.0229517870 -0.2115935572 0.4640048771
C 3.2200443272 -1.3255107761 -0.1807421771
C 1.5429561302 1.4497699478 -0.2417155002
N 1.0173126095 0.0643478981 -0.3507983694
H 4.5044032495 1.8756498355 0.2689160784
H 4.9042275951 0.9427700301 -1.1580655277
H 3.5569109159 0.0280432378 1.4281188614
H 5.0367219971 -0.5715670966 0.7022102896
H 2.0602716700 -0.7539571058 -0.3620114194
H 1.7576278507 1.7007202520 0.8056739646
C 3.6720159473 -1.8725448004 -1.5172470763
H 4.6276979404 -2.4077822068 -1.4026842057
H 3.8295125071 -1.0859529125 -2.2630443437
H 2.9740726185 2.5844483368 -1.3830270775
H 2.6705989844 0.9758745196 -2.0046724673
H 2.9283167016 -2.1077064546 0.5249734230
S 0.0727115236 -0.4977576717 0.9532073721
O 0.2424244735 0.3400511508 2.1534843326
O 0.3111337628 -1.9460003659 1.0256160699
C -1.5996018701 -0.2464570390 0.3498382803
C -2.0357613957 -0.9387319320 -0.7839152097
C -2.4595071808 0.5980651800 1.0490059238
C -3.3453510824 -0.7679985267 -1.2187121565
H -1.3560797861 -1.5984450100 -1.3128250702
C -3.7717843411 0.7546243296 0.5994529655
H -2.1007142357 1.1164673710 1.9318167751
C -4.2345632790 0.0786727473 -0.5354948338
H -3.6878045716 -1.3016710412 -2.1022744427
H -4.4461819661 1.4115072664 1.1432474443
C -5.6592081760 0.2362081388 -1.0109376266
H -6.1642025716 1.0609317214 -0.4989718696
H -5.7017926264 0.4293736931 -2.0890986653
H -6.2406326102 -0.6759111678 -0.8243600451
H 2.9449437338 -2.5852991454 -1.9202755977
C 0.4837242529 2.4305363416 -0.7710448732
H 0.8632666798 3.4565740743 -0.7000121129
H 0.2504974746 2.2136785685 -1.8191124432
H -0.4434024530 2.3728561458 -0.1946352302
40
-1149.18864000
C 3.2930649863 1.4063609152 -0.4133646107
C 3.9923930948 0.0687539266 -0.7382044840
C 3.0864978177 -1.1817090397 -0.7571371425
C 1.7484862535 -0.9817907469 -1.4407335989
C 2.2679259765 1.4002199835 0.7398646049
N 0.9662406895 0.8944121871 0.2068471398
H 4.7912691043 -0.1066785270 -0.0062626075
H 4.4948665345 0.1813781186 -1.7077050556
H 2.8865933976 -1.5082240154 0.2698027521
H 3.6327789189 -2.0084980163 -1.2397063585
H 1.2324835778 0.0053855270 -0.7050563394
H 2.6395568587 0.7563342414 1.5504305037
C 1.7140470744 -0.5482011928 -2.8893836102
H 2.3323561226 0.3360186977 -3.0770024115
H 0.6927386251 -0.3245377906 -3.2160285591
H 4.0691580044 2.1413165537 -0.1675527086
H 2.7798443049 1.7993830925 -1.3009300084
H 1.0379387422 -1.7832723179 -1.2161912008
S 0.0245381943 0.0674875430 1.3547554163
O -0.3024656994 1.0238732066 2.4241257938
O 0.6182225528 -1.2316007327 1.7233150138
C -1.4464566067 -0.2576863117 0.3837510948
C -2.1759886744 0.8080494907 -0.1515719939
C -1.8795216739 -1.5727065532 0.2282035410
C -3.3412308069 0.5406221449 -0.8621731068
H -1.8276262205 1.8266817022 -0.0180690882
C -3.0539426500 -1.8216871742 -0.4839935255
H -1.3011680964 -2.3816873664 0.6615949053
C -3.8008351651 -0.7753679866 -1.0375397906
H -3.9082519266 1.3658719571 -1.2865795834
H -3.3942596752 -2.8466707652 -0.6087368392
C -5.0818759159 -1.0448525362 -1.7905299257
H -5.1914999545 -2.1071390099 -2.0290249561
H -5.9567705890 -0.7462076849 -1.1987117571
H -5.1207489571 -0.4801227525 -2.7290452246
H 2.0962484658 -1.3537802874 -3.5361879959
C 2.0562540310 2.8216015946 1.2840181227
H 1.3004352570 2.8288408776 2.0711635444
H 2.9991007741 3.2035776927 1.6937076996
H 1.7305951200 3.4893067161 0.4783820978
40
-1149.18814100
C 3.1613490820 -0.1567731647 1.7016415826
C 3.9049144410 -0.9293654319 0.5922006007
C 3.0446665163 -1.9162592929 -0.2243839657
C 2.0157375749 -1.2730383216 -1.1441765775
C 2.1773951093 0.9561054867 1.2763860980
N 0.9942356709 0.3062415663 0.6600835350
H 4.7042830946 -1.5061812410 1.0749786395
H 4.4136723399 -0.2270733859 -0.0803506034
H 2.5309021148 -2.5929005111 0.4707323916
H 3.7127828172 -2.5476028558 -0.8325534421

H 1.4238404668 -0.4340022426 -0.3407208215
H 1.7909737173 1.4081701701 2.1996680955
C 2.5088133077 -0.5382511383 -2.3723157601
H 3.3685441907 0.1041319716 -2.1555781868
H 1.7217161221 0.0888731584 -2.8024214536
H 2.6167836974 -0.8702427092 2.3337090526
H 3.9127769938 0.3205004548 2.3443701095
H 1.1699773098 -1.9342063549 -1.3645111542
S -0.2127254447 1.3290636611 0.0642964146
O 0.0652148076 1.7858268984 -1.3093128173
O -0.4812476400 2.3219326915 1.1181015807
C -1.5911752916 0.1841431391 -0.0226371266
C -2.1266831088 -0.3451074014 1.1553595949
C -2.1422651843 -0.1281512059 -1.2628337055
C -3.2164208714 -1.2048655482 1.0775583281
H -1.6922714017 -0.0842135275 2.1146812378
C -3.2373898873 -0.9924858763 -1.3221983263
H -1.7195141534 0.3070306852 -2.1619106422
C -3.7898290303 -1.5423364160 -0.1602341973
H -3.6341088741 -1.6210377694 1.9914191494
H -3.6698329950 -1.2387785111 -2.2888334268
C -4.9844105592 -2.4641786862 -0.2237200189
H -5.8777740768 -1.9823941664 0.1937923110
H -5.2142810493 -2.7527581980 -1.2538426976
H -4.8136314560 -3.3798112760 0.3545063368
H 2.8258541894 -1.2592999985 -3.1414089521
C 2.8435271126 2.0743795210 0.4581164254
H 3.1580621408 1.7311956691 -0.5312302967
H 2.1592111202 2.9146361264 0.3174822435
H 3.7280587269 2.4438964133 0.9906623942

TS 13 → 14

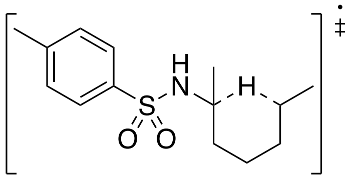

Name	E(B3LYP)	H(B3LYP)	E(RO-B2PLYP-D3)	H(RO-B2PLYP-D3)	NImag
Tosyl_NH_C2_radical_2_heptyl_1_5_HAT_TS_0040	-1149.990114	-1149.633181	-1149.544933	-1149.188000	-1745.4719
Tosyl_NH_C2_radical_2_heptyl_1_5_HAT_TS_0005	-1149.991390	-1149.635594	-1149.543563	-1149.187767	-1695.3806
Tosyl_NH_C2_radical_2_heptyl_1_5_HAT_TS_0027	-1149.992455	-1149.635524	-1149.544508	-1149.187577	-1749.5542
Tosyl_NH_C2_radical_2_heptyl_1_5_HAT_TS_0025	-1149.991841	-1149.635011	-1149.544195	-1149.187365	-1734.7440
Tosyl_NH_C2_radical_2_heptyl_1_5_HAT_TS_0030	-1149.992893	-1149.636074	-1149.544051	-1149.187232	-1724.9568
Tosyl_NH_C2_radical_2_heptyl_1_5_HAT_TS_0013	-1149.991390	-1149.634651	-1149.543562	-1149.186823	-1695.3812
Tosyl_NH_C2_radical_2_heptyl_1_5_HAT_TS_0016	-1149.991965	-1149.635257	-1149.542224	-1149.185516	-1691.6100
Tosyl_NH_C2_radical_2_heptyl_1_5_HAT_TS_0021	-1149.990725	-1149.634025	-1149.541876	-1149.185176	-1705.1486

40

C 3.9909747918 -1.9673029196 -0.1834225689
C 4.6637396255 -0.6237530025 0.1644488216
C 3.7686950008 0.2486248819 1.0305742881
C 3.9405399099 1.7478323745 0.9567229737
C 2.6654075206 -1.7581249976 -0.9426306355
H 3.8076799012 -2.5365825030 0.7384113497
H 4.6633471038 -2.5808915321 -0.7953547190
H 4.8963613285 -0.0922921178 -0.7707232231
H 5.6320142915 -0.8136619768 0.6547701336
H 3.6716284727 -0.1285296550 2.0551081438
H 2.5275937822 -0.1137665052 0.5065199436
H 4.9062175509 2.0689525711 1.3809472511
H 3.1459742555 2.2660022245 1.5026788402
H 2.2201961125 -2.7275430767 -1.2134228160
H 2.8937928067 -1.2479987481 -1.8905985607
C 1.6716998512 -0.9162776511 -0.1375329722
N 0.7944800663 -0.1706180485 -1.0297040734
H 1.2626986548 0.1676103042 -1.8689151442
S -0.1264204620 1.1450511839 -0.4619073644
O -0.2357185467 2.0265546779 -1.6316313614
O 0.3908863643 1.6443414637 0.8188841691
C -1.7338679558 0.4188760987 -0.1386727459
C -2.3169732764 0.5903507961 1.1144595059
C -2.4160486184 -0.2423467030 -1.1642312633
C -3.5954025568 0.0787348838 1.3448203991
H -1.7734251786 1.1164908007 1.8916710038
C -3.6874260797 -0.7471677070 -0.9162929375
H -1.9537990885 -0.3605467080 -2.1386995924
C -4.2974519294 -0.5970038737 0.3407290649
H -4.0530052598 0.2106715502 2.3220292559
H -4.2198523836 -1.2646421442 -1.7108785131
C -5.6742305800 -1.1628424540 0.5949860072
H -6.3829389101 -0.8505288665 -0.1808785458
H -5.6578085898 -2.2602468519 0.5925689817
H -6.0685102215 -0.8387686070 1.5627493919
C 0.9147140072 -1.6813303244 0.9331338954
H 0.2094758292 -2.3942802628 0.4829119226
H 1.6148539502 -2.2431362165 1.5597033745
H 0.3529237735 -1.0102193743 1.5893128138
H 3.9143937987 2.1016280543 -0.0818930620
40

C 1.5572676931 -2.0548890215 0.0485461068
C 2.9159952525 -2.2957816718 -0.6460997446
C 3.5337203304 -1.0111185987 -1.1837837794
C 3.0435078678 -0.5162962861 -2.5275511558
C 1.6934904437 -1.0425549818 1.2013857365
H 1.1681045443 -3.0043299611 0.4361582721
H 0.8220897218 -1.6867694231 -0.6773537233
H 2.7841307578 -3.0282058499 -1.4590512626
H 3.6004636052 -2.7579989624 0.0780733743
H 4.6195490747 -0.9518265285 -1.0680825388
H 3.0360214957 -0.1171071906 -0.2306760773
H 3.3128259021 -1.2186171042 -3.3327095095
H 3.4819683403 0.4549298412 -2.7860347340
H 2.4091850454 -1.4333525139 1.9370004518
H 0.7408126483 -0.9333423876 1.7357728227
C 2.2106971254 0.3120044439 0.7238172617
N 1.2313442383 1.0736963467 -0.0434566006
H 1.6377282425 1.8799933138 -0.5161834764
S -0.2222965212 1.6562346309 0.6496554884
O -0.4664979549 2.9284912778 -0.0434678981
O -0.1746488962 1.5825503741 2.1164940457
C -1.4572094048 0.4741861354 0.1020057560
C -1.7092203310 0.3210455769 -1.2648734425
C -2.1958030853 -0.2310478201 1.0484875496
C -2.6996948423 -0.5632974606 -1.6766017087
H -1.1384715203 0.8899057298 -1.9916766141
C -3.1889236291 -1.1130076780 0.6169684712
H -1.9943159171 -0.0843389730 2.1040972452
C -3.4548615862 -1.2953918928 -0.7442417543
H -2.8964750534 -0.6864446178 -2.7390633857
H -3.7671947933 -1.6645490515 1.3539749820
C -4.5266531217 -2.2512848828 -1.2110820327
H -4.1032521335 -3.0502725075 -1.8322950558
H -5.0406917150 -2.7205118741 -0.3669823631
H -5.2800604102 -1.7366405494 -1.8197003236
C 3.0256809393 1.1189779200 1.7216730252
H 3.8701289832 0.5283930981 2.0957583213
H 3.4306453205 2.0275042607 1.2586559273
H 2.4104436545 1.4249890621 2.5773053844
H 1.9529871878 -0.4057178162 -2.5440147589
40

C 2.6427114438 -1.8972156200 -1.2305226389
C 3.3337402060 -0.5887103282 -1.6651586080
C 3.8647225345 0.1881271933 -0.4721252588
C 4.0893814905 1.6732043878 -0.6381548556
C 1.4324581953 -1.6268719626 -0.3135240390
H 3.3699901975 -2.5432308394 -0.7182976096
H 2.3046761392 -2.4565362967 -2.1114895743
H 2.6096874722 0.0352986605 -2.2067505308
H 4.1427353089 -0.8192011158 -2.3774960360
H 4.6938521170 -0.3275013513 0.0283937735
H 2.8136502585 -0.0623227138 0.4139781026
H 4.8532903410 1.8809949457 -1.4059079647
H 4.4365441632 2.1349241598 0.2937720999
H 0.9591761887 -2.5747034370 -0.0123077882

H 0.6852602180 -1.0765369785 -0.8940754045
C 1.8244654573 -0.8180207592 0.9233523050
N 0.7357552558 -0.0449802366 1.5092064302
H 0.9664718014 0.3436822395 2.4219816703
S -0.0740326967 1.1922286427 0.6731236463
O 0.6748642578 1.6096939382 -0.5195238972
O -0.4279845888 2.1548749051 1.7238043646
C -1.5771630461 0.3979146024 0.1006322488
C -1.9088101710 0.4638997764 -1.2506029298
C -2.4365087007 -0.2081407979 1.0218755528
C -3.1106991268 -0.0992187046 -1.6839857899
H -1.2329292034 0.9494447356 -1.9460871827
C -3.6282116926 -0.7649638296 0.5723196417
H -2.1710748215 -0.2446892560 2.0733015553
C -3.9847942068 -0.7217231827 -0.7863082116
H -3.3716246280 -0.0494647686 -2.7381077809
H -4.2972442605 -1.2399219898 1.2860516786
C -5.2780223016 -1.3427262937 -1.2576286214
H -6.1285311722 -0.9932332604 -0.6607337775
H -5.4796389470 -1.1024088467 -2.3057783688
H -5.2485614517 -2.4360128027 -1.1661231321
C 2.4844004746 -1.6309157639 2.0258629147
H 3.3396363257 -2.1867648876 1.6302182688
H 2.8582484200 -0.9874403979 2.8328575406
H 1.7819712474 -2.3541628086 2.4642986443
H 3.1662919331 2.1772500563 -0.9422752084
40

C 2.7185050005 -2.1640970753 0.6228793083
C 3.1012102513 -2.1866825140 -0.8702430065
C 3.6570080462 -0.8492344180 -1.3295349476
C 3.4795473211 -0.4654123159 -2.7799399671
C 1.6437689003 -1.0954875077 0.9139263595
H 3.6147978073 -1.9727375562 1.2295720073
H 2.3425637731 -3.1473996860 0.9301888244
H 2.2068916689 -2.4320218199 -1.4616273763
H 3.8248425617 -2.9966364928 -1.0558102515
H 4.6597134062 -0.6446332533 -0.9379020810
H 2.8776224441 -0.0134175610 -0.5218779810
H 2.4214762978 -0.4871456706 -3.0697984759
H 4.0116531347 -1.1587607229 -3.4515998869
H 1.3658079804 -1.1187958386 1.9761957552
H 0.7427430651 -1.3476432031 0.3418182414
C 2.1086582289 0.3042276484 0.5132140665
N 1.0635931711 1.1403103514 -0.0587004538
H 1.4189648398 2.0262415667 -0.4169736740
S -0.3424735495 1.5579482473 0.8303544486
O -0.6533532317 2.9285986784 0.4011708135
O -0.1783508078 1.2163354540 2.2502366020
C -1.6071897461 0.4703659890 0.1673399300
C -2.0339142366 0.6364992685 -1.1533637317
C -2.1835502793 -0.4944598386 0.9897902654
C -3.0380661251 -0.1872033308 -1.6495338325
H -1.5872010504 1.4029333519 -1.7782006180
C -3.1919772701 -1.3115779816 0.4752466943
H -1.8461677832 -0.5958455441 2.0155044912
C -3.6338810559 -1.1733345649 -0.8452220863
H -3.3705534794 -0.0626439930 -2.6774499653
H -3.6436255887 -2.0659057057 1.1147226872
C -4.7372205718 -2.0456269633 -1.3950560916
H -4.4641849836 -2.4692996962 -2.3684698390
H -4.9688666351 -2.8737121358 -0.7184154853
H -5.6595934364 -1.4692674909 -1.5424091460
C 3.0229522477 1.0062269293 1.5073227000
H 3.8641484895 0.3626307971 1.7887868920
H 3.4399075656 1.9276455819 1.0813568859
H 2.4714285764 1.2715122010 2.4179092913
H 3.8651541601 0.5401771001 -2.9844525172
40

C 3.8871212570 -1.2651190125 -1.0475744730
C 4.6658257896 -0.6769654644 0.1494301694
C 3.8256216273 0.3056521048 0.9579671669
C 3.7568464341 1.7308800534 0.4528247187
C 2.6084081334 -1.9863067480 -0.5771487836
H 4.5215965881 -1.9677804663 -1.6012926705
H 3.6356450268 -0.4627597272 -1.7572315903
H 5.5851696818 -0.1932564410 -0.2177508448
H 4.9891366658 -1.5086421012 0.7904207034
H 3.9801888204 0.2409474404 2.0385767783
H 2.5617512847 -0.2650808341 0.7761104848
H 3.5058635791 1.7773890520 -0.6148316286
H 4.7263206029 2.2411820594 0.5729073320
H 2.8988446894 -2.8025923806 0.0968288612
H 2.0873340314 -2.4539572769 -1.4263882266
C 1.6557618174 -1.0607350097 0.1855059640
N 0.8703546544 -0.2835812504 -0.7660872598
H 1.3934816316 0.0079408018 -1.5896396626
S -0.0485812770 1.0668887445 -0.2963587074
O -0.0363514794 1.9301496497 -1.4846240318
O 0.3747667566 1.5737180833 1.0154122287
C -1.6991912540 0.3943334782 -0.0940532401
C -2.3769365493 0.6073249122 1.1039166107
C -2.3165882262 -0.2651910218 -1.1608978785
C -3.6853586742 0.1388538339 1.2372978150
H -1.8823564756 1.1317794239 1.9142378289
C -3.6193220394 -0.7263871461 -1.0098031017
H -1.7807674633 -0.4162015389 -2.0922253650
C -4.3245196143 -0.5342905272 0.1904274349
H -4.2164511354 0.3031820853 2.1714817910
H -4.1014923854 -1.2423164962 -1.8368193972
C -5.7343207050 -1.0540891380 0.3408259843
H -5.7508346049 -2.1511865920 0.3680350338
H -6.1988349593 -0.6922524641 1.2629807474
H -6.3652967292 -0.7440713395 -0.5002705569
C 0.8123657513 -1.7331690698 1.2507290405
H 0.1174233246 -2.4626855550 0.8094732131
H 1.4632220973 -2.2639076481 1.9537112533
H 0.2275017284 -1.0079969785 1.8233456561
H 3.0005101410 2.3096707890 0.9908522655
40

C 4.1217867936 -1.2664050058 -0.6779645448
C 4.7247102218 -0.5796443662 0.5653562271
C 3.7519297850 -0.5622284357 1.7324269425
C 3.8892110718 0.5377754675 2.7594734982
C 2.8376554708 -0.5560013455 -1.1491355740
H 3.9085185548 -2.3197866125 -0.4480958528
H 4.8549200139 -1.2724593804 -1.4933723267
H 4.9970137562 0.4535255416 0.3022091800
H 5.6649068074 -1.0808292730 0.8460874207
H 3.5949492914 -1.5532982784 2.1726009677
H 2.5577326941 -0.4099686512 1.0168563703
H 3.8743819224 1.5301669090 2.2905294950
H 4.8409496616 0.4610959145 3.3100118777
H 2.4343023770 -1.0369594966 -2.0505707308
H 3.0950384476 0.4721700104 -1.4398702981
C 1.7677313334 -0.5070809844 -0.0506338298
N 0.9355998712 0.6849331193 -0.0063628399
H 1.4768748058 1.5488660336 0.0008287925
S -0.3010828529 0.9550212697 -1.1612121927
O -0.1160915275 0.1053575415 -2.3466221155
O -0.3668221590 2.4181259684 -1.2820661488
C -1.7796762607 0.3805871147 -0.3230523593
C -2.5996778655 -0.5504774663 -0.9555955922
C -2.1334626673 0.9256749675 0.9146801140
C -3.7835436259 -0.9485048569 -0.3317456473
H -2.3094951576 -0.9529648320 -1.9200332941
C -3.3145494581 0.5155594349 1.5227702629
H -1.4886635110 1.6554767421 1.3929967127
C -4.1580744715 -0.4277014167 0.9117183428
H -4.4258176700 -1.6749211283 -0.8233311159
H -3.5913093260 0.9351569751 2.4871548093
C -5.4309294106 -0.8704124367 1.5931676680
H -6.0495022125 -1.4816181005 0.9291552401
H -5.2138789174 -1.4669706416 2.4885361303
H -6.0293581990 -0.0109708357 1.9171383820
C 0.9829621247 -1.7879313166 0.1603092675
H 1.6664502783 -2.6288150955 0.3195484076
H 0.3292157619 -1.7076665071 1.0346981508

H 0.3643800863 -2.0289679315 -0.7137852840
H 3.0829430542 0.5033587971 3.5009902022
40

C 1.5565156818 -2.0548196392 0.0491578110
C 2.9150353466 -2.2963146606 -0.6457005836
C 3.5331845821 -1.0119729269 -1.1836772753
C 3.0431569860 -0.5173081579 -2.5275698987
C 1.6932632059 -1.0422373037 1.2017100261
H 1.1671547860 -3.0040509167 0.4370922689
H 0.8213055829 -1.6866509634 -0.6766872354
H 2.7827372867 -3.0287917579 -1.4585350323
H 3.5994478349 -2.7587145506 0.0784086520
H 4.6190307262 -0.9530048538 -1.0679637057
H 3.0358044556 -0.1175366175 -0.2308053560
H 1.9526718832 -0.4063807511 -2.5440793412
H 3.3122613461 -1.2199036423 -3.3325597218
H 2.4090302849 -1.4330246326 1.9372612872
H 0.7407392031 -0.9326368402 1.7362940131
C 2.2107012948 0.3120584077 0.7236578480
N 1.2314136232 1.0737072152 -0.0437310221
H 1.6378679751 1.8798288365 -0.5166926910
S -0.2221157083 1.6565583166 0.6493424929
O -0.4661083397 2.9288182318 -0.0438450215
O -0.1744215414 1.5829190256 2.1161840174
C -1.4571519093 0.4745528672 0.1018116895
C -1.7099656726 0.3221063130 -1.2648996323
C -2.1959739195 -0.2303840400 1.0484799404
C -2.7012690052 -0.5615408105 -1.6764654910
H -1.1401240089 0.8918035028 -1.9917586404
C -3.1897823121 -1.1115164669 0.6171913854
H -1.9947553770 -0.0831279405 2.1040662588
C -3.4557774984 -1.2940933748 -0.7440980518
H -2.8995373645 -0.6832408481 -2.7387988861
H -3.7689309808 -1.6619635300 1.3543461340
C -4.5221506344 -2.2562572318 -1.2104368057
H -5.1019705260 -2.6500149278 -0.3703358655
H -5.2190663691 -1.7724572080 -1.9048488010
H -4.0809984376 -3.1099764665 -1.7404900213
C 3.0259617917 1.1191675849 1.7211720538
H 2.4108998608 1.4254918850 2.5768198500
H 3.8703786502 0.5285548173 2.0952817437
H 3.4309900887 2.0275131172 1.2578572896
H 3.4819384996 0.4537152552 -2.7862746236
40

C 3.7646844150 -1.3429130813 -1.3363264055
C 4.0809723699 0.1578259660 -1.1748552798
C 3.8901337993 0.6230774310 0.2590573744
C 3.6355790441 2.0935652265 0.4969456308
C 2.3033735644 -1.6606784510 -0.9613957494
H 4.4544160561 -1.9318410502 -0.7140573275
H 3.9453673455 -1.6557216996 -2.3720752441
H 3.4147924186 0.7345787855 -1.8302160977
H 5.1096187732 0.3567071593 -1.5173774517
H 4.6273893625 0.1891637229 0.9461435489
H 2.7653168948 -0.1365161100 0.5738281652
H 3.4168305828 2.3020257297 1.5500696772
H 2.7867492302 2.4468227116 -0.0976151358
H 2.1077004893 -2.7395278133 -1.0724592282
H 1.6409812606 -1.1403017591 -1.6590687498
C 1.9656260124 -1.2153542509 0.4626282362
N 0.5427365874 -0.9156961541 0.6572970720
H 0.2511485568 -1.0912415506 1.6192924768
S -0.0263519385 0.6467642276 0.2583725637
O 0.5570331271 0.9887917833 -1.0439735490
O 0.1217108933 1.5671609047 1.3960285091
C -1.7766400997 0.3143299788 0.0599374963
C -2.6837758281 0.8869568804 0.9485157338
C -2.2141060827 -0.4800579598 -1.0043190091
C -4.0482022108 0.6496675506 0.7720227516
H -2.3210077093 1.5099859375 1.7589317793
C -3.5763871809 -0.7060915980 -1.1636696682
H -1.4953442775 -0.9126242794 -1.6924162745
C -4.5150223581 -0.1456526511 -0.2804355305
H -4.7595399694 1.0936596779 1.4638407836
H -3.9214729083 -1.3256922238 -1.9880295644
C -5.9919735602 -0.3886228332 -0.4799057353
H -6.5769837753 -0.0247392527 0.3700353544
H -6.3578371064 0.1231450699 -1.3791810297
H -6.2071836146 -1.4558839540 -0.6077004396
C 2.4337808262 -2.1620579745 1.5544074957
H 3.4917516457 -2.4070530017 1.4199900868
H 2.3222485577 -1.7168547428 2.5511538279
H 1.8698508850 -3.1070961206 1.5338287819
H 4.5130290176 2.6998153684 0.2167776735
40

C 4.0735153691 -0.1006741541 -0.7320053349
C 4.6794499810 -0.6777307066 0.5681893080
C 3.6850717198 -0.6842144315 1.7230143927
C 3.5352082264 0.5963068105 2.5169242971
C 2.8190648170 -0.8848033772 -1.1616937862
H 4.8210175492 -0.1280448417 -1.5335331918
H 3.8251378765 0.9617868165 -0.5908041981
H 5.5798684003 -0.1023576158 0.8369534673
H 5.0161347886 -1.7031164309 0.3655389194
H 3.7309704706 -1.5765755297 2.3531615679
H 2.4998556927 -0.8602514899 0.9959753057
H 3.3979680964 1.4712506726 1.8682637315
H 4.4325202475 0.7949842480 3.1242277543
H 3.1058894585 -1.9305220656 -1.3392085350
H 2.4196612992 -0.4995318235 -2.1082445651
C 1.7257640197 -0.8567552370 -0.0869986443
N 1.0095202409 0.4052091824 0.0564769604
H 1.6229149763 1.2116243979 0.1623692990
S -0.1886560560 0.8990916473 -1.0582575239
O -0.0772548573 0.1651366890 -2.3275216467
O -0.1234840596 2.3667680568 -1.0265194334
C -1.7170463938 0.3745190620 -0.2787590460
C -2.6085850183 -0.4179810122 -0.9973568624
C -2.0320854991 0.8261335360 1.0062637535
C -3.8262202782 -0.7722074232 -0.4132723306
H -2.3467888701 -0.7488488269 -1.9964776485
C -3.2478539804 0.4613442263 1.5733545463
H -1.3312445645 1.4492865941 1.5518782762
C -4.1639803493 -0.3436169451 0.8750688777
H -4.5241603619 -1.3908474084 -0.9718310223
H -3.4948693428 0.8082126657 2.5740351487
C -5.4749471393 -0.7390510379 1.5118642860
H -6.0072704956 0.1354185182 1.9040180006
H -6.1326400511 -1.2417402014 0.7964483165
H -5.3152583084 -1.4230688868 2.3551771118
C 0.8229587330 -2.0708298604 -0.0153857107
H 1.4252421352 -2.9743357796 0.1312460016
H 0.1160122760 -1.9875570740 0.8162235596
H 0.2490948698 -2.2051886641 -0.9421739267
H 2.6805801075 0.5528102771 3.2015249507
40

C 3.9988158746 -1.2023029730 0.4570786220
C 3.1001694055 -1.6774187564 1.6147108128
C 1.6770053094 -1.9484840603 1.1556888403
C 0.5826964748 -1.8673783704 2.1941684860
C 3.4615934455 0.0920147805 -0.1803789456
H 4.0728452400 -1.9935839963 -0.3012403300
H 5.0193821298 -1.0273068740 0.8185362673
H 3.0888657158 -0.9017845917 2.3956816796
H 3.5419237113 -2.5720513508 2.0827740779
H 1.6022231616 -2.8346025412 0.5148237949
H 1.5509314058 -0.9239512901 0.1863055009
H -0.4078084522 -2.0148400792 1.7516301134
H 0.5809704098 -0.8934368484 2.7002452502
H 4.1328946092 0.4376643111 -0.9803003580
H 3.4695158883 0.8781606633 0.5898005706
C 2.0365131569 -0.0699183167 -0.7266851375
N 1.3745267066 1.2208218375 -0.7192773000
H 1.5918852611 1.8073259788 0.0816087972
S -0.1895930985 1.5823301466 -1.2392435307
O -0.3259845063 1.1161202202 -2.6192365510

O -0.3461206418 2.9924600126 -0.8705358987
C -1.3646710454 0.6445130417 -0.2521786646
C -1.7682415332 1.1437458790 0.9899485826
C -1.8882841945 -0.5519881629 -0.7422871941
C -2.6949499196 0.4290500933 1.7430140292
H -1.3795710426 2.0931852902 1.3437229125
C -2.8167690759 -1.2553759503 0.0257156925
H -1.5929422990 -0.9092358382 -1.7228451982
C -3.2352169362 -0.7794047448 1.2751938815
H -3.0142391951 0.8202070821 2.7061638741
H -3.2311186788 -2.1840468419 -0.3590402076
C -4.2661399458 -1.5258468552 2.0879070500
H -5.2509460204 -1.0470889323 2.0092063094
H -4.3749519966 -2.5597488034 1.7462629244
H -4.0009060962 -1.5447053312 3.1507612745
C 1.9694573768 -0.7938736465 -2.0614971992
H 0.9477059791 -1.0494528561 -2.3490468415
H 2.3880730468 -0.1699260492 -2.8616433840
H 2.5480577465 -1.7212010512 -2.0041950625
H 0.7110562309 -2.6331284774 2.9773666853

12-Cl

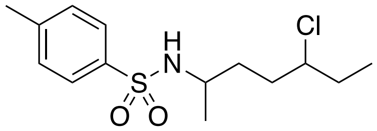

Name	E(B3LYP)	H(B3LYP)	E(RO-B2PLYP-D3)	H(RO-B2PLYP-D3)
Tosyl_NH_C5_Cl_2_heptane_0012	-1610.284715	-1609.916299	-1609.736586	-1609.36817
Tosyl_NH_C5_Cl_2_heptane_0010	-1610.284158	-1609.915875	-1609.735925	-1609.367642
Tosyl_NH_C5_Cl_2_heptane_0061	-1610.284191	-1609.915625	-1609.736049	-1609.367483
Tosyl_NH_C5_Cl_2_heptane_0023	-1610.283276	-1609.914728	-1609.735165	-1609.366617
Tosyl_NH_C5_Cl_2_heptane_0096	-1610.283071	-1609.914752	-1609.733655	-1609.365336
Tosyl_NH_C5_Cl_2_heptane_0003	-1610.283894	-1609.915575	-1609.733417	-1609.365098
Tosyl_NH_C5_Cl_2_heptane_0050	-1610.283223	-1609.914942	-1609.732649	-1609.364368
Tosyl_NH_C5_Cl_2_heptane_0068	-1610.284044	-1609.915912	-1609.732491	-1609.364359
Tosyl_NH_C5_Cl_2_heptane_0029	-1610.283106	-1609.914853	-1609.731676	-1609.363423

41

C -2.0405535573 -0.9271930577 1.8742331215
C -3.0601214206 0.0771111946 1.3205037658
C -3.3465159899 0.0140607942 -0.1808952984
C -4.3985716157 1.0323804621 -0.6205778541
C -0.6320500326 -0.8565630827 1.2549347651
N -0.0856684929 0.5071613386 1.4187184486
H -2.7003732314 1.0946318715 1.5206189731
H -4.0042991346 -0.0429451547 1.8668111543
Cl -3.9036526054 -1.6930655530 -0.6414784149
H -2.4302838300 0.1600795876 -0.7561157490
H -5.3465833420 0.8109812629 -0.1136457607
C -4.6094184651 1.1266197266 -2.1334916096
H -3.6721580972 1.3742662048 -2.6454396700
H -5.3381959790 1.9093931520 -2.3710445562
H -1.9541833231 -0.7681238411 2.9588265870
H -2.4164325069 -1.9477014908 1.7392736703
S 0.6835216472 1.3366009331 0.1868121265
O 0.9972247855 2.6485406005 0.7567839579
O -0.1600027235 1.1718352573 -1.0022708021
C 2.2469405931 0.5249893770 -0.1724994328
C 2.3134134812 -0.4414279373 -1.1780416102
C 3.3804400160 0.8582048283 0.5735225525
C 3.5249416576 -1.0865358676 -1.4227935212
H 1.4325652968 -0.6670001876 -1.7696082671
C 4.5822775556 0.2043614200 0.3140849605
H 3.3216820655 1.6336964515 1.3304117884
C 4.6752316953 -0.7772152392 -0.6840795588
H 3.5786650179 -1.8375679774 -2.2071367771
H 5.4660348999 0.4653213587 0.8913706199
C 5.9914700356 -1.4561453347 -0.9785458388
H 6.5874289807 -0.8659178706 -1.6871344979
H 6.5926182314 -1.5765225615 -0.0712053416
H 5.8422639116 -2.4453381236 -1.4228878367
H -4.9808172924 0.1823951018 -2.5430707762
C 0.2975096173 -1.9222905329 1.8497874545
H -4.0588892356 2.0071105980 -0.2420291196
H 0.4190883864 -1.7743198034 2.9309116301
H 1.2879223287 -1.8910191684 1.3855466501
H -0.1203950745 -2.9235279415 1.6986433733
H 0.3244926270 0.7206630273 2.3242355512
H -0.7180599565 -1.0189970157 0.1765523470
41

C -2.1469551700 -0.4253796625 1.2732134523
C -1.8896188568 -0.6567653930 -0.2188057905
C -3.1483382982 -0.7793027272 -1.0781831666
C -2.8499425063 -1.1690864616 -2.5269586251
C -0.8639667756 -0.3284783637 2.1171963129
N -0.0889011167 0.8523313614 1.6704313777
H -1.3306765851 -1.5965230513 -0.3438456141
H -1.2526716472 0.1405596327 -0.6166284313
Cl -4.0788849861 0.8165656959 -1.0463049994
H -3.8471463343 -1.4955909110 -0.6334767283
H -2.2249037767 -0.3900751338 -2.9811541744
C -4.0832774567 -1.4353511587 -3.3932162419
H -4.7000522987 -0.5376286179 -3.4937944780
H -4.7074411650 -2.2270938152 -2.9610916722
H -2.7367901992 0.4894675285 1.4118945624
H -2.7547961127 -1.2479176493 1.6750139111
S 1.5884979678 0.8900104729 1.8692342421
O 2.0165500870 -0.0667890286 2.8989259218
O 1.9150934044 2.3167683018 1.9706307964
C 2.1913768214 0.2675168023 0.2988708081
C 2.8351952439 -0.9666213912 0.2492844425
C 2.0371203876 1.0444061766 -0.8535938943
C 3.3186140938 -1.4323036628 -0.9752760700
H 2.9599858336 -1.5436090569 1.1591366724
C 2.5249024153 0.5649582021 -2.0639743363
H 1.5484712157 2.0117090091 -0.7966694796
C 3.1731326831 -0.6793791288 -2.1456257873
H 3.8214852247 -2.3952591198 -1.0172913632
H 2.4069543858 1.1665375961 -2.9621952084
C 3.7159521617 -1.1741099895 -3.4651130051
H 4.5998350004 -0.5983295318 -3.7682760855
H 4.0101828038 -2.2264962446 -3.4089523285
H 2.9751622527 -1.0707571396 -4.2663480549
H -3.7847549155 -1.7565976852 -4.3970041374
C -1.1673126999 -0.2924983174 3.6201841051
H -2.2330974322 -2.0785335166 -2.4837962144
H -1.7882147544 0.5779945370 3.8692187948
H -0.2402544770 -0.2382485848 4.1961999437
H -1.7158845244 -1.1906379410 3.9265934245
H -0.4582032423 1.7424359504 2.0024887352
H -0.2348182436 -1.2009288191 1.9049991942
41

C -2.5539130400 0.4858246134 1.4018189826
C -2.4396458643 0.8397022855 -0.0869524692
C -2.1075260655 -0.3094396341 -1.0405403834
C -1.9713220677 0.1501337172 -2.4930521307
C -1.2656788457 -0.0256912868 2.0716659178
N -0.2192676692 1.0182809474 1.9508111648
H -1.6478410753 1.5893015799 -0.2134918091
H -3.3776688049 1.3036369808 -0.4164265188
Cl -3.4093187176 -1.6170933055 -0.9226718705
H -1.1936834055 -0.8232952418 -0.7307644051
H -2.9283480277 0.5735878211 -2.8230579209
C -1.5009191353 -0.9297900844 -3.4700635876
H -2.2142748866 -1.7574436539 -3.5232856586
H -0.5293124124 -1.3373317804 -3.1660324456

H -2.8929942886 1.3802252036 1.9438979401
H -3.3336238980 -0.2709954291 1.5427853464
S 1.4163384128 0.5899232897 1.9966613898
O 1.6086621249 -0.7054504470 2.6621363256
O 2.0969470698 1.7997560833 2.4720059400
C 1.8111417223 0.3454348252 0.2636034945
C 2.0905876824 -0.9367231729 -0.2039229079
C 1.8753443717 1.4513289551 -0.5904372218
C 2.4242720201 -1.1125375697 -1.5492777591
H 2.0612826952 -1.7765481865 0.4818294941
C 2.2093639138 1.2577851180 -1.9261340882
H 1.6809663800 2.4471298547 -0.2053257806
C 2.4869876255 -0.0258150478 -2.4288274157
H 2.6495275687 -2.1111015862 -1.9150166172
H 2.2681313805 2.1163987149 -2.5910237107
C 2.8395245976 -0.2165518701 -3.8847698037
H 3.6278596931 0.4776151904 -4.1979585414
H 3.1866776562 -1.2348768098 -4.0827570292
H 1.9711463915 -0.0300533561 -4.5294160291
H -1.3906999610 -0.5134531984 -4.4776606829
C -1.5157430024 -0.4431122039 3.5263088255
H -1.2450732179 0.9754352641 -2.4884279788
H -2.2722479986 -1.2344561425 3.5745281150
H -1.8845847325 0.4073133305 4.1150465211
H -0.5952423632 -0.8130493570 3.9841717184
H -0.3425793440 1.8003742291 2.5934861364
H -0.8934134504 -0.8961054815 1.5205562840
41

C -2.0773549843 -0.8826669326 1.8337651958
C -3.1055951942 0.0107012451 1.1263258633
C -3.3409066524 -0.2620997287 -0.3591813449
C -4.3813807759 0.6546075817 -1.0082451131
C -0.6432635041 -0.8109541201 1.2758123920
N -0.1549299268 0.5835413758 1.3368921006
H -2.7744519833 1.0528122474 1.2071770456
H -4.0646584443 -0.0649061769 1.6545834397
Cl -3.8873803849 -2.0182631674 -0.5931907258
H -2.4052785046 -0.1980281129 -0.9171114673
H -4.5550009391 0.3005309247 -2.0303378375
C -3.9393204408 2.1257083958 -1.0499734433
H -3.9069233118 2.5755636032 -0.0520941505
H -4.6428740819 2.7118555927 -1.6513180082
H -2.0506406816 -0.6041646935 2.8970369337
H -2.4045225260 -1.9278568704 1.7939105104
S 0.6060984538 1.3408475121 0.0548697687
O 0.8842375820 2.6971486854 0.5316917137
O -0.2214031421 1.0730860849 -1.1274978503
C 2.1894118063 0.5396254047 -0.2329462302
C 2.2856899042 -0.4965641804 -1.1638615809
C 3.3084601194 0.9509901195 0.4958054915
C 3.5127144871 -1.1316593223 -1.3506410209
H 1.4157400202 -0.7838112502 -1.7446940731
C 4.5261297513 0.3059705264 0.2947595744
H 3.2262234684 1.7782816079 1.1933392687
C 4.6491554460 -0.7440835487 -0.6276259136
H 3.5895763969 -1.9370746530 -2.0769381365
H 5.3986714831 0.6276751275 0.8582675091
C 5.9820934922 -1.4141254413 -0.8608744998
H 5.8579445391 -2.4375366875 -1.2288706761
H 6.5705742059 -0.8668158785 -1.6090607739
H 6.5785472899 -1.4511012496 0.0567205501
H -2.9433079336 2.2286656416 -1.4948075683
C 0.2967401550 -1.7751763552 2.0104715880
H -5.3351975888 0.5551839089 -0.4743266531
H 0.3572842801 -1.5234912152 3.0774748381
H 1.3072655938 -1.7421407830 1.5917169864
H -0.0725508068 -2.8036096301 1.9346945502
H 0.2317115740 0.8843043706 2.2278111908
H -0.6678310315 -1.0773094039 0.2149931368
41

C -2.1195051883 -0.5068945417 2.0528306310
C -2.7382718018 0.6277817793 1.2231241649
C -3.3155454449 0.2414667958 -0.1408418846
C -3.8421126219 1.4485707835 -0.9168639669
C -0.8586868773 -1.1808423897 1.4764014215
N 0.2838586336 -0.2429386196 1.3426835147
H -1.9849739571 1.4053180146 1.0381540107
H -3.5319959897 1.1009350580 1.8147306504
Cl -4.6747684505 -0.9959316260 0.0934238285
H -2.5748613624 -0.2871519556 -0.7432290468
H -4.6467692542 1.9231084851 -0.3404325783
C -4.3126859140 1.1430548397 -2.3407060465
H -3.5066450032 0.6924055552 -2.9317041925
H -4.6241483882 2.0635168886 -2.8467220887
H -1.8700634886 -0.1114140335 3.0492002585
H -2.8734479253 -1.2853536588 2.2167001653
S 0.6169606066 0.5650696729 -0.0959173390
O 0.4376974577 2.0089919594 0.1183717353
O -0.1081692549 -0.1416833931 -1.1608020109
C 2.3700880423 0.2849700418 -0.3415269022
C 3.2800178860 1.2883242291 -0.0137058407
C 2.8035131844 -0.9352580550 -0.8669439020
C 4.6428220240 1.0568113110 -0.2042487162
H 2.9221449897 2.2370635923 0.3714056964
C 4.1657801011 -1.1472479737 -1.0508527313
H 2.0812770465 -1.6984184366 -1.1377352950
C 5.1070645646 -0.1579045167 -0.7227374789
H 5.3553936250 1.8375055337 0.0499423533
H 4.5067344334 -2.0948957114 -1.4609462291
C 6.5806271027 -0.3951713765 -0.9519798642
H 6.8794892456 -1.3981781686 -0.6275336651
H 6.8315526001 -0.3126149921 -2.0175091389
H 7.1931902347 0.3332238279 -0.4119760023
H -5.1610660407 0.4520821034 -2.3410841906
C -0.4152732954 -2.3632588393 2.3472421593
H -3.0132538219 2.1692762424 -0.9571093686
H -0.1745515100 -2.0350301572 3.3661716680
H 0.4742268640 -2.8401834265 1.9254642607
H -1.2149430991 -3.1090238093 2.4152212242
H 0.3984589495 0.3948999513 2.1274649522
H -1.0611194258 -1.5470932895 0.4669408261
41

C -2.1665837423 -0.5179662940 1.3791841223
C -1.9093791553 -0.8366378787 -0.0966844996
C -3.1681841897 -1.1176725578 -0.9165155150
C -2.9040925447 -1.6680142363 -2.3225649111
C -0.8828918785 -0.2547059817 2.1859042330
N -0.2076651243 0.9414514210 1.6311114558
H -1.2804186787 -1.7377071798 -0.1665953898
H -1.3398704800 -0.0222470568 -0.5536395372
Cl -4.1995926383 0.4126596377 -1.0346261093
H -3.8167378034 -1.8159686138 -0.3792073416
H -2.4376106875 -2.6549842784 -2.1859598533
C -2.0217448611 -0.8034807231 -3.2281774855
H -2.4572597601 0.1910519638 -3.3674109082
H -1.9257439158 -1.2697020537 -4.2148274924
H -2.8272512505 0.3547297945 1.4603436196
H -2.6995426243 -1.3542222450 1.8526748006
S 1.4689222389 1.1004736644 1.7544703822
O 1.9987843906 0.2718611009 2.8461478103
O 1.7050262770 2.5478265227 1.7138895224
C 2.0473152875 0.3711104583 0.2209427878
C 2.7928912096 -0.8048136589 0.2569617545
C 1.7699937121 1.0091014415 -0.9921838870
C 3.2549624147 -1.3539943190 -0.9409956751
H 3.0094996095 -1.2731871276 1.2109139489
C 2.2377522471 0.4478711876 -2.1754773289
H 1.2014715036 1.9334077124 -1.0037979297
C 2.9876382175 -0.7411590778 -2.1701132157
H 3.8365399166 -2.2721714967 -0.9159327458
H 2.0254144597 0.9421508799 -3.1205467716
C 3.5091293412 -1.3228978744 -3.4623863038
H 3.8690903655 -2.3470523439 -3.3251862190
H 2.7340961533 -1.3355317243 -4.2371082035
H 4.3447662705 -0.7277535507 -3.8527434369
H -1.0125438247 -0.6795647039 -2.8207610343
C -1.1615595011 -0.1203082984 3.6877533613

H -3.8699159316 -1.8473328316 -2.8085605724
H -1.8412161951 0.7196471951 3.8822755889
H -0.2307428624 0.0479021713 4.2352938382
H -1.6356864745 -1.0285106189 4.0773118237
H -0.6246270822 1.8269498377 1.9154119118
H -0.1917978885 -1.0913985250 2.0296081131
41

C -2.1579405020 -1.2542904137 0.7729626448
C -2.6636330411 -0.1196787627 -0.1257766335
C -4.1593438591 0.1716651043 -0.0054331623
C -4.6429700133 1.2299207522 -0.9977047692
C -0.6876549889 -1.6522478942 0.5413642607
N 0.2424614250 -0.5240710934 0.7710764855
H -2.4714045767 -0.3773259733 -1.1763187736
H -2.1067565403 0.8044682664 0.0683364735
Cl -4.5537512500 0.7132047982 1.7193067586
H -4.7391696384 -0.7496840834 -0.1240162404
H -4.1192647219 2.1718686025 -0.7937659582
C -6.1568092973 1.4537493238 -1.0178444749
H -6.4250627110 2.1870494484 -1.7860472399
H -6.5172547701 1.8252163291 -0.0540981560
H -2.2928288838 -0.9802362372 1.8278963579
H -2.7742562764 -2.1500701491 0.6121879924
S 0.8481890120 0.4089280071 -0.4925470720
O 0.6134461774 1.8131169417 -0.1315536713
O 0.3572737068 -0.1629056667 -1.7529407613
C 2.6204141940 0.1361137488 -0.4404737330
C 3.1781265318 -0.9095509903 -1.1777626786
C 3.4223815618 0.9704930053 0.3389163370
C 4.5532715240 -1.1240127417 -1.1179263238
H 2.5435979316 -1.5329442130 -1.7987394129
C 4.7959662280 0.7407153925 0.3864684947
H 2.9760017880 1.7941885400 0.8859036022
C 5.3831643163 -0.3062643651 -0.3375161363
H 4.9907450153 -1.9370868662 -1.6923242382
H 5.4233878282 1.3899356695 0.9923531569
C 6.8768142244 -0.5268618968 -0.3062596763
H 7.3138715263 -0.1803730147 0.6358041361
H 7.1286459634 -1.5852793522 -0.4300963383
H 7.3725396299 0.0226102620 -1.1175287592
H -6.6925638866 0.5232751253 -1.2429483492
C -0.2758825431 -2.8290586386 1.4346359457
H -4.3102928979 0.8983017647 -1.9918098122
H -0.3662928940 -2.5679263596 2.4960107768
H 0.7632944925 -3.1119807744 1.2425788489
H -0.9164466110 -3.6985234639 1.2472679022
H 0.0047872822 0.0807166617 1.5534385618
H -0.5572919223 -1.9356876339 -0.5071805331
41

C -2.2344387238 -0.1106698440 1.2826857138
C -1.9423947621 -0.7122504793 -0.0957182071
C -3.1803637386 -1.1179104885 -0.8943794375
C -2.8796854446 -1.8414758148 -2.2110121819
C -0.9705395974 0.2540106611 2.0808218616
N -0.2275722414 1.3027021072 1.3438896845
H -1.3335480282 -1.6177556227 0.0375165533
H -1.3407409260 -0.0152842095 -0.6892948328
Cl -4.1870015105 0.3822356273 -1.2813371127
H -3.8491441099 -1.7285721247 -0.2789876346
H -3.8173989245 -1.9436624521 -2.7677923452
C -2.2561721753 -3.2309747853 -2.0150172980
H -1.2614036905 -3.1813149471 -1.5601306343
H -2.1497724267 -3.7344038685 -2.9819461263
H -2.8609223159 0.7835650758 1.1741001398
H -2.8174048018 -0.8246504715 1.8809839240
S 1.4447404117 1.4486180951 1.5336458455
O 1.8892139165 0.8066279840 2.7783430729
O 1.7244334137 2.8630860048 1.2622639532
C 2.0884653767 0.4613131577 0.1814740842
C 1.9269335843 0.9082210973 -1.1335691242
C 2.7692281590 -0.7223568251 0.4567371734
C 2.4454690916 0.1480633218 -2.1758885387
H 1.4084958271 1.8407655114 -1.3316958656
C 3.2831261758 -1.4729808070 -0.6026832906
H 2.8983308129 -1.0401222726 1.4856596862
C 3.1318541574 -1.0531386024 -1.9289623730
H 2.3218996530 0.4926529982 -3.1998847410
H 3.8148298882 -2.3973768146 -0.3911129846
C 3.7104625760 -1.8531188671 -3.0716508522
H 4.6031642596 -1.3638287926 -3.4822208178
H 4.0029263238 -2.8576153505 -2.7510063244
H 2.9926722075 -1.9538926243 -3.8936707719
H -2.8863043412 -3.8657422994 -1.3799614017
C -1.3003077237 0.6748053573 3.5182911848
H -2.2194709469 -1.2115796803 -2.8201026707
H -0.3853015924 0.9124306321 4.0665223306
H -1.8244195755 -0.1295371987 4.0472475437
H -1.9532982916 1.5573307484 3.5234968870
H -0.6298237390 2.2354028014 1.4283492076
H -0.3087789962 -0.6195407697 2.1122909956
41

C -2.1593427877 -0.6529173238 1.9351253089
C -3.0156737707 0.3086335439 1.0974805033
C -3.4115581442 -0.1691203167 -0.3006400333
C -4.2754769728 0.8258222871 -1.0813906557
C -0.7483023281 -0.9611254619 1.3989807898
N 0.0875114966 0.2570221407 1.3558855420
H -2.4771619876 1.2564344788 0.9682742553
H -3.9298603419 0.5433527066 1.6575868749
Cl -4.3462877629 -1.7634641800 -0.1701936372
H -2.5269638150 -0.4240502174 -0.8858319290
H -4.5949988829 0.3399548202 -2.0098425075
C -3.5396588827 2.1312927938 -1.4206661581
H -2.5844535154 1.9326180172 -1.9182580143
H -3.3308038443 2.7330614950 -0.5299099984
H -2.0565219738 -0.2350201421 2.9481431968
H -2.6924271263 -1.6025965302 2.0526387129
S 0.5863292354 0.9988811570 -0.0569400371
O 0.6320995286 2.4319168657 0.2549599737
O -0.2084587787 0.4727177147 -1.1768531139
C 2.2701969136 0.4391400617 -0.3177286460
C 3.3184638426 1.0959698497 0.3307051727
C 2.5161067039 -0.6472283288 -1.1572179597
C 4.6218638268 0.6463233704 0.1398010122
H 3.1129679472 1.9541972058 0.9618890700
C 3.8283408014 -1.0846758796 -1.3346942614
H 1.6929213577 -1.1293285023 -1.6734018730
C 4.8989497782 -0.4489917062 -0.6926569335
H 5.4401540712 1.1571716723 0.6416447624
H 4.0228888150 -1.9307094039 -1.9892430831
C 6.3206068335 -0.9072965984 -0.9145791483
H 6.9170208976 -0.8204303590 -0.0000295339
H 6.3589675655 -1.9488376929 -1.2487323429
H 6.8147456505 -0.2978938470 -1.6827337692
H -4.1533846910 2.7421361231 -2.0918245919
C -0.0524489602 -2.0388754939 2.2406996704
H -5.1873472711 1.0378289951 -0.5083381087
H 0.0509122749 -1.7155629801 3.2836451016
H 0.9485077949 -2.2453654214 1.8499365398
H -0.6310769183 -2.9694184278 2.2299332895
H -0.0653337356 0.9504509163 2.0815624545
H -0.8247690387 -1.3160798894 0.3671561574
41

C -2.0769868354 -0.8571301740 1.6876352587
C -3.0539801019 0.1251923231 1.0267560658
C -3.2552814253 -0.0277334391 -0.4811732117
C -4.1753232328 1.0246605246 -1.1083944995
C -0.6284003074 -0.8118197287 1.1662142001
N -0.0881354734 0.5557592029 1.3178928505
H -2.6925019743 1.1497039395 1.1868021958
H -4.0218388603 0.0431209283 1.5335112163
Cl -3.8981527976 -1.7258491314 -0.8646056059
H -2.2957031906 0.0086883443 -0.9979535639
H -3.6579167584 1.9877261896 -0.9913505422
C -5.5927356181 1.1218063378 -0.5366117417
H -5.5925408853 1.4220138961 0.5168829681

H -6.1178149841 0.1645070131 -0.6174799213
H -2.0639656672 -0.6561115507 2.7685514560
H -2.4477197251 -1.8816874712 1.5671617871
S 0.7418793126 1.3472302676 0.1008521941
O 1.0366643030 2.6731434572 0.6480093641
O -0.0467869605 1.1529982415 -1.1208939835
C 2.3147642475 0.5169284358 -0.1611180749
C 2.4225024489 -0.4764727273 -1.1363700978
C 3.4133241562 0.8625005014 0.6301662191
C 3.6393749944 -1.1357606721 -1.3047605872
H 1.5697981182 -0.7117679154 -1.7642404682
C 4.6212234979 0.1941749166 0.4468628675
H 3.3241051562 1.6582284092 1.3626909324
C 4.7548776648 -0.8142723541 -0.5193552122
H 3.7252091545 -1.9078262704 -2.0654791623
H 5.4779383371 0.4647549598 1.0593867629
C 6.0784950779 -1.5092614050 -0.7317615298
H 6.6394168724 -1.5988529846 0.2043954973
H 5.9426528634 -2.5134357980 -1.1458193199
H 6.7077096470 -0.9481100149 -1.4352152489
H -6.1707008102 1.8701191289 -1.0897942301
C 0.2543180859 -1.8548656026 1.8635505691
H -4.2247520541 0.8328614630 -2.1862348065
H 0.3011173848 -1.6664362222 2.9442006793
H 1.2745697260 -1.8408657369 1.4679847414
H -0.1548610012 -2.8610204211 1.7216621629
H 0.2775966848 0.7951168192 2.2358166696
H -0.6379226244 -1.0151097794 0.0913629797

13-Cl

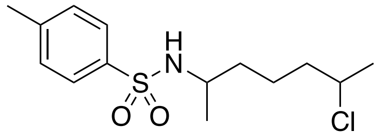

Name	E(B3LYP)	H(B3LYP)	E(RO-B2PLYP-D3)	H(RO-B2PLYP-D3)
Tosyl_NH_C6_Cl_2_heptane_0033	-1610.284161	-1609.915894	-1609.73487	-1609.366603
Tosyl_NH_C6_Cl_2_heptane_0022	-1610.284134	-1609.91577	-1609.734482	-1609.366118
Tosyl_NH_C6_Cl_2_heptane_0004	-1610.28328	-1609.914822	-1609.734058	-1609.3656
Tosyl_NH_C6_Cl_2_heptane_0011	-1610.28456	-1609.916419	-1609.732724	-1609.364583
Tosyl_NH_C6_Cl_2_heptane_0012	-1610.283502	-1609.915194	-1609.732454	-1609.364146
Tosyl_NH_C6_Cl_2_heptane_0014	-1610.283669	-1609.915395	-1609.732073	-1609.363799
Tosyl_NH_C6_Cl_2_heptane_0018	-1610.283662	-1609.915386	-1609.732055	-1609.363779
Tosyl_NH_C6_Cl_2_heptane_0055	-1610.283558	-1609.915329	-1609.731809	-1609.36358
Tosyl_NH_C6_Cl_2_heptane_0087	-1610.282739	-1609.91437	-1609.730585	-1609.362216

41

C -0.9493184055 2.5720787214 0.5856175481
C -1.6975776465 1.2350121945 0.5415447079
C -3.2158092809 1.4154629063 0.4329096384
C -4.0266259881 0.1198558132 0.4437995476
C 0.5818900327 2.4387903558 0.6647308719
N 1.0684705740 1.7779336789 -0.5684848195
H -1.4647454465 0.6592939507 1.4495829670
H -1.3337549650 0.6427646711 -0.3033448410
H -3.5744221891 2.0137716564 1.2847327127
Cl -3.6276289209 -0.8940774356 -1.0457684119
C -5.5321505726 0.3484223143 0.4845710842
H -6.0776167172 -0.5991315884 0.4772331298
H -5.8605201965 0.9434229407 -0.3737893338
H -1.2061635331 3.1715233565 -0.3009408427
H -1.2819380848 3.1578899561 1.4540222679
S 2.4997411825 0.8792753442 -0.5355371412
O 3.3765517063 1.3111574365 0.5625223953
O 2.9696569541 0.8952153042 -1.9256109709
C 1.9174393778 -0.7646326809 -0.1212781259
C 2.3792989614 -1.3809310876 1.0392770656
C 1.0429302224 -1.4274158810 -0.9884296018
C 1.9485517968 -2.6751657695 1.3383400164
H 3.0652945845 -0.8510599877 1.6910621199
C 0.6223539545 -2.7140505546 -0.6731577260
H 0.6960598925 -0.9383245747 -1.8926726654
C 1.0681596774 -3.3593097220 0.4931481569
H 2.3057108084 -3.1587803481 2.2441499602
H -0.0650699352 -3.2280049001 -1.3403952971
C 0.6109502166 -4.7628856641 0.8112079145
H 1.0230744270 -5.4842188758 0.0941389900
H 0.9280292553 -5.0713682713 1.8119772816
H -0.4808497549 -4.8473154878 0.7596976819
H -5.7941777053 0.8910675913 1.4015462059
C 1.2679888046 3.7882531968 0.9110685174
H -3.7147498093 -0.5126502259 1.2799865150
H 1.0461231893 4.4903318625 0.0963566084
H 2.3512501784 3.6601281632 0.9768626135
H 0.9128630130 4.2397652140 1.8446442890
H 1.1163906368 2.3948150419 -1.3786582884
H 0.8295927130 1.7603576123 1.4896904966
H -3.4678300180 1.9832716693 -0.4726474226
41

C -0.9223685142 2.8429190815 -0.3833298179
C -1.7130668172 1.5796334605 -0.7549406600
C -2.1540683105 0.7418398646 0.4518551433
C -2.9289194278 -0.5283901003 0.1026768060
C 0.4645964220 2.6087082255 0.2447807282
N 1.3004243828 1.8449880071 -0.7115226039
H -1.1040802369 0.9599421171 -1.4247938599
H -2.6018868133 1.8796502694 -1.3211707797
H -1.2723565593 0.4078487941 1.0167035341
Cl -4.5284086123 -0.1003387921 -0.7125563003
C -3.2195243196 -1.4084210070 1.3114553667
H -3.7930738487 -2.2951608406 1.0276676018
H -3.7866209732 -0.8583943351 2.0695576096
H -0.7913941833 3.4595375534 -1.2842948570
H -1.5086251792 3.4542993504 0.3167713860
S 2.5454150633 0.8548196297 -0.1459900680
O 2.9685332792 1.2542171883 1.2031607315
O 3.5012217442 0.7937072820 -1.2574065989
C 1.7413471957 -0.7417703332 0.0165885654
C 1.3065099027 -1.4146911551 -1.1295001933
C 1.5953821675 -1.3123116395 1.2792325702
C 0.7103937017 -2.6643589193 -0.9976863397
H 1.4406558684 -0.9654306923 -2.1081751415
C 0.9949592076 -2.5685275852 1.3933037392
H 1.9565886479 -0.7802777266 2.1526678349
C 0.5467170269 -3.2633227230 0.2636005188
H 0.3733962320 -3.1910587549 -1.8873790176
H 0.8839799501 -3.0177770903 2.3771256715
C -0.0708270915 -4.6360407620 0.3854546499
H 0.6361169428 -5.4133411639 0.0677779754
H -0.3606125987 -4.8562673005 1.4173480401
H -0.9606500597 -4.7332165764 -0.2465124501
H -2.2711300913 -1.7357677027 1.7552781383
C 1.1271401291 3.9236188558 0.6741006566
H -2.3885834742 -1.0972356703 -0.6590478544
H 2.1000414770 3.7313250911 1.1334022238
H 0.4990240153 4.4550955708 1.3982314925
H 1.2708036956 4.5856498909 -0.1900679942
H 1.6777282086 2.4050393992 -1.4750564130
H 0.3575644569 1.9704520484 1.1281447157
H -2.7576250076 1.3500065868 1.1393847698
41

C -0.6564426623 2.3816211434 1.2845202401
C -1.7147022219 2.5845088367 0.1905653530
C -2.8080289357 1.5088741753 0.1667250123
C -3.9115885559 1.7487368108 -0.8620132507
C 0.1579117572 1.0747301246 1.2015937648
N 0.7911031145 0.9720055887 -0.1275239852
H -1.2191793241 2.6131162649 -0.7877330374
H -2.1773353220 3.5666062748 0.3417025049
H -2.3618166074 0.5391140353 -0.0900322644
Cl -4.8692600458 3.2718742850 -0.4338297514
C -4.8837666487 0.5832927819 -0.9863909645
H -4.3359725040 -0.3099599058 -1.3106830936

H -5.6661885195 0.7956335657 -1.7201402911
H 0.0442051323 3.2294021331 1.2639183482
H -1.1355082107 2.4096038014 2.2733840457
S 0.8442078281 -0.4392488921 -1.0192345552
O 1.4184872270 -0.0495918150 -2.3085822476
O -0.4844900540 -1.0494738676 -0.8956271772
C 2.0073439619 -1.5735539075 -0.2474160880
C 1.5496990941 -2.5221334414 0.6691553156
C 3.3655060495 -1.4835912723 -0.5624543535
C 2.4669396453 -3.3739085363 1.2831095594
H 0.4884654798 -2.6005860929 0.8798399702
C 4.2673131738 -2.3432348246 0.0599166764
H 3.7034001664 -0.7634592491 -1.3006471576
C 3.8359381988 -3.2993171284 0.9914829620
H 2.1113509855 -4.1150312719 1.9948306044
H 5.3241317563 -2.2761914505 -0.1871191143
C 4.8185082312 -4.2483233125 1.6350224052
H 4.4573714546 -4.6027983740 2.6056934324
H 4.9810856325 -5.1321886866 1.0041379318
H 5.7939848492 -3.7742545994 1.7860285996
H -5.3605158482 0.3671755744 -0.0245313256
C 1.1809616096 0.9762928449 2.3415192298
H -3.4732422658 1.9832088596 -1.8359582958
H 1.7320361818 0.0317605464 2.3039822398
H 0.6809417551 1.0423711540 3.3144068615
H 1.9057017900 1.7989906356 2.2866866094
H 1.6065905729 1.5560983889 -0.2916687575
H -0.5320015580 0.2280527636 1.2620611828
H -3.2666908424 1.4034892045 1.1599047549
41

C -0.3397326595 2.9577766334 0.3244717380
C -1.4757499259 2.4934454895 -0.6006482091
C -2.6683774985 1.8698825866 0.1359064023
C -3.8111580910 1.4236217406 -0.7742083614
C 0.4384529702 1.8590645932 1.0766195009
N 1.1182177329 0.9226832045 0.1493243154
H -1.0883807327 1.7753940819 -1.3358256074
H -1.8246739169 3.3605718177 -1.1729177880
H -2.3410492774 0.9695292031 0.6711976967
Cl -4.5741544830 2.8863303592 -1.6106695808
C -4.9046096407 0.6605569835 -0.0389102317
H -5.3308274862 1.2663569415 0.7676523584
H -4.4763322747 -0.2494042082 0.3989738438
H 0.3837815481 3.5431063264 -0.2637721048
H -0.7432579069 3.6529558124 1.0738955682
S 0.4792977879 -0.5855912387 -0.2365338699
O 0.2531357773 -0.6301352581 -1.6878610110
O -0.6042792765 -0.8577763038 0.7183354622
C 1.8155488114 -1.7262821080 0.1221403453
C 2.6385054788 -2.1689396496 -0.9123103679
C 2.0156012878 -2.1605387044 1.4347664110
C 3.6819400897 -3.0476135194 -0.6207245197
H 2.4530774538 -1.8393545604 -1.9289651851
C 3.0611259092 -3.0376359226 1.7063390391
H 1.3521645676 -1.8253146357 2.2251789977
C 3.9107135712 -3.4948101077 0.6865027540
H 4.3250488359 -3.3951353847 -1.4254654657
H 3.2186740648 -3.3788404011 2.7267540578
C 5.0232977948 -4.4690573068 0.9920921330
H 4.6361652695 -5.4924243087 1.0831723299
H 5.7806236155 -4.4739857983 0.2020853883
H 5.5195091222 -4.2264244436 1.9381315557
H -5.7115669933 0.3712805264 -0.7177262658
C 1.4837009879 2.4621123588 2.0238962176
H -3.4199683954 0.8251063337 -1.6012417237
H 1.0038329669 3.1173483177 2.7598779904
H 2.2186994083 3.0596821528 1.4706813098
H 2.0218771845 1.6728869132 2.5571803920
H 1.4967880378 1.3554570509 -0.6898800128
H -0.2547299545 1.2484309045 1.6605718441
H -3.0620130156 2.5700063216 0.8856092074
41

C -0.3383104767 2.9575843067 0.3215723111
C -1.4751196602 2.4925304773 -0.6022191013
C -2.6677455162 1.8708796022 0.1359358565
C -3.8110928086 1.4235106943 -0.7729295203
C 0.4393850131 1.8595963585 1.0752525722
N 1.1182316520 0.9210442940 0.1494361742
H -1.0885982714 1.7731095392 -1.3364955807
H -1.8237945809 3.3589941085 -1.1756461881
H -2.3406736291 0.9713289842 0.6727393678
Cl -4.5735937096 2.8850489473 -1.6118583352
C -4.9046886988 0.6624122066 -0.0358109516
H -5.3303355754 1.2699680358 0.7697333336
H -4.4767436412 -0.2469410493 0.4036589348
H 0.3853527309 3.5413735124 -0.2680175254
H -0.7409552911 3.6543468083 1.0700053838
S 0.4771650173 -0.5869013609 -0.2344441099
O 0.2478308545 -0.6324898763 -1.6852562574
O -0.6047014828 -0.8574148024 0.7228425435
C 1.8141571462 -1.7273172591 0.1224586690
C 2.6298766599 -2.1771480908 -0.9144123973
C 2.0170445614 -2.1603271462 1.4352298570
C 3.6706173276 -3.0600164903 -0.6250096966
H 2.4373383734 -1.8546090095 -1.9319979044
C 3.0595272109 -3.0414930589 1.7045115891
H 1.3539954316 -1.8258842562 2.2263015861
C 3.9062675906 -3.5008158593 0.6829954789
H 4.3043242148 -3.4178152956 -1.4326499042
H 3.2150650138 -3.3867661890 2.7238721555
C 5.0477714036 -4.4387426676 0.9953793903
H 5.8900463946 -3.8976874437 1.4462795769
H 4.7449551509 -5.2144748221 1.7073223074
H 5.4197564978 -4.9327634135 0.0925971166
H -5.7120150660 0.3722595232 -0.7138162607
C 1.4854486284 2.4635089848 2.0210803700
H -3.4204931056 0.8232269467 -1.5989578195
H 1.0063276627 3.1202733944 2.7561843309
H 2.2206102892 3.0597040089 1.4665876642
H 2.0232967346 1.6747848355 2.5554321098
H 1.4972751925 1.3522363662 -0.6903840771
H -0.2539409849 1.2504470355 1.6605787816
H -3.0607460333 2.5725455165 0.8845270920
41

C -1.1986990778 1.9973376299 -0.0459440579
C -1.7435597756 0.5749234786 -0.2231731893
C -3.1956787445 0.5067060261 -0.7177042491
C -4.2590638649 1.1472683991 0.1760920295
C 0.3021368842 2.0528167300 0.2931844236
N 1.0705648229 1.5115972840 -0.8527351216
H -1.6593696916 0.0321442261 0.7263461619
H -1.1117448190 0.0432052744 -0.9435238492
H -3.2693760738 1.0115726393 -1.6934333960
Cl -4.2722184965 0.3348229627 1.8323476048
C -5.6608642676 1.0709157567 -0.4153806844
H -5.9531373452 0.0312384834 -0.5951848085
H -5.6821323509 1.6058297384 -1.3732611321
H -1.3749530523 2.5856752504 -0.9601271594
H -1.7367031388 2.5130453739 0.7599997395
S 2.5836347646 0.8008680142 -0.5953004990
O 3.1891668924 1.2807126829 0.6545699350
O 3.2767861414 0.9481439393 -1.8804899144
C 2.1732411325 -0.9263433115 -0.3463948090
C 2.3293334057 -1.4957447745 0.9151869928
C 1.7320947805 -1.6910176643 -1.4308250907
C 2.0233375509 -2.8463948420 1.0934287584
H 2.6962648919 -0.8896838551 1.7363439338
C 1.4330745842 -3.0344439413 -1.2348017371
H 1.6389585440 -1.2386141072 -2.4127925794
C 1.5688493485 -3.6331898690 0.0294560806
H 2.1457489788 -3.2942653155 2.0763373899
H 1.0942723657 -3.6338444014 -2.0765691122
C 1.2234387286 -5.0898349983 0.2270049503
H 1.5507891890 -5.4503853359 1.2067336539
H 0.1399607901 -5.2514762303 0.1585007920
H 1.6915112125 -5.7183515346 -0.5393573300
H -6.3981598090 1.5276037892 0.2504471242

C 0.7561655140 3.4649579801 0.6826269282
H -4.0028066608 2.1858432526 0.3988165633
H 0.1940336760 3.8268555539 1.5511238888
H 0.5841535036 4.1689754325 -0.1425527873
H 1.8204247476 3.4706363169 0.9307312230
H 1.1748366282 2.1676560514 -1.6261996516
H 0.4918333660 1.3754871507 1.1339498705
H -3.4736486507 -0.5410418579 -0.8889825249
41

C -0.9486676279 2.6196947654 0.2907434176
C -1.6873529118 1.2819394755 0.1678535103
C -3.1924414312 1.4646451997 -0.0605144627
C -4.0230753392 0.1793937774 -0.1379724120
C 0.5732853375 2.4770134781 0.4679030543
N 1.1396814001 1.8477446911 -0.7488861482
H -1.5287282865 0.6945877116 1.0811617138
H -1.2444681220 0.7103231327 -0.6546714123
H -3.6153546841 2.1019713154 0.7260245659
Cl -4.0434158163 -0.6640112552 1.5048728707
C -3.5789638209 -0.8178057275 -1.2011289049
H -2.5691793521 -1.1905155346 -1.0082738408
H -3.5846101694 -0.3269771569 -2.1830046450
H -1.1447913951 3.2421066668 -0.5957778620
H -1.3395523498 3.1819863752 1.1499332558
S 2.5285221983 0.8906978626 -0.6339863950
O 3.3250928794 1.2502700041 0.5473179736
O 3.1089834754 0.9293289090 -1.9812479307
C 1.8514664123 -0.7415009845 -0.3310137429
C 1.1829190509 -1.4096881173 -1.3607888482
C 2.0187222672 -1.3358505208 0.9181608788
C 0.6651789712 -2.6784566295 -1.1209857827
H 1.0853537976 -0.9449589425 -2.3365928371
C 1.4918293577 -2.6089537713 1.1409100938
H 2.5638787561 -0.8096682225 1.6942206375
C 0.8038214930 -3.2956076415 0.1337007143
H 0.1519456676 -3.2055045909 -1.9218303895
H 1.6222736495 -3.0758923293 2.1139011450
C 0.2083145552 -4.6592471640 0.3895370085
H 0.2980412684 -5.3070908818 -0.4890940418
H 0.6965878940 -5.1583053424 1.2322817702
H -0.8605092950 -4.5814114424 0.6283562830
H -4.2596954375 -1.6726948305 -1.2411009204
C 1.2517887799 3.8120021184 0.7967402937
H -5.0710839805 0.4461071122 -0.2956422605
H 0.8366671408 4.2426498045 1.7151008791
H 1.0935828725 4.5377787771 -0.0121164996
H 2.3269171550 3.6707804676 0.9343950472
H 1.2811208017 2.4952112438 -1.5235498775
H 0.7608745427 1.7748905395 1.2885258846
H -3.3564226133 1.9965674734 -1.0106243161
41

C -0.9980692599 3.0326164958 -0.1832428456
C -1.8901853115 1.8730651305 -0.6515164408
C -2.2369060245 0.8665978747 0.4607185461
C -3.3704732500 -0.0826968467 0.0659962661
C 0.4147239116 2.6547919622 0.3009052361
N 1.1252811584 1.9630671436 -0.7998265110
H -1.3975750397 1.3574754913 -1.4828103540
H -2.8193549174 2.3041853272 -1.0509304627
H -1.3568783521 0.2696180569 0.7268420743
Cl -3.7714058675 -1.1552719839 1.5138867672
C -3.0774014410 -0.9720624169 -1.1347026340
H -2.1634689354 -1.5536412880 -0.9769991586
H -2.9407170089 -0.3530781834 -2.0291907548
H -0.8972686092 3.7572148712 -1.0046351256
H -1.4957138442 3.5715721036 0.6346093355
S 2.4345649620 0.9436689146 -0.4599963242
O 3.0378105759 1.2750549480 0.8379752460
O 3.2282514680 0.9494934573 -1.6940674328
C 1.6606707345 -0.6626728163 -0.2747697077
C 1.2818859704 -1.3779252272 -1.4118829503
C 1.4670828139 -1.1908501167 1.0028686456
C 0.6869326272 -2.6284400760 -1.2586586472
H 1.4681460907 -0.9672445309 -2.3988333190
C 0.8643645580 -2.4405381836 1.1369766481
H 1.8025841668 -0.6345809603 1.8714601918
C 0.4596404944 -3.1754717331 0.0137181784
H 0.4002870000 -3.1939299912 -2.1419046598
H 0.7115222872 -2.8539481211 2.1307060958
C -0.2181137671 -4.5149561663 0.1752601539
H -1.2716526762 -4.3870155371 0.4556531542
H -0.1901443080 -5.0942742295 -0.7527778786
H 0.2558943656 -5.1102489534 0.9631778953
H -3.9052738368 -1.6614943220 -1.3220416959
C 1.1730671799 3.8845337836 0.8184761039
H -4.2913161842 0.4839370015 -0.0995820424
H 0.6277166760 4.3539072947 1.6454654395
H 1.2812057289 4.6339828334 0.0232441651
H 2.1676025844 3.6035683674 1.1717969534
H 1.4168389424 2.5819989504 -1.5560254602
H 0.3313098406 1.9246604415 1.1132995100
H -2.5409960507 1.4087224694 1.3650457748
41

C -0.5234240691 2.6091250273 0.1554886825
C -1.8107796663 1.7765005630 0.0863827627
C -3.0285284101 2.6350324551 -0.2951385406
C -4.2962190186 1.8087285714 -0.5221122961
C 0.7283335143 1.8470298819 0.6316638749
N 1.0857598805 0.7183196899 -0.2580519934
H -1.9742113715 1.2881962826 1.0540566380
H -1.6906732217 0.9684180003 -0.6467327029
H -3.2249079131 3.3820377486 0.4853782779
Cl -5.6200223757 2.9295712555 -1.1534699790
C -4.8176744144 1.0811249506 0.7096342431
H -4.0807711854 0.3421714309 1.0446148963
H -5.7498447184 0.5546334513 0.4876972176
H -0.3150329598 3.0530370835 -0.8308975022
H -0.6749333911 3.4568444966 0.8383303203
S 0.5541038314 -0.8539688276 0.0360198046
O -0.1833165562 -1.3013638466 -1.1539970799
O -0.0496189346 -0.8728425441 1.3740441575
C 2.0657988350 -1.8161771216 0.1108465459
C 2.7556855919 -1.9191440683 1.3205248187
C 2.5307003729 -2.4593909422 -1.0359526936
C 3.9313589466 -2.6632348855 1.3693680760
H 2.3624572431 -1.4410796796 2.2115266336
C 3.7102305153 -3.1997049721 -0.9679406484
H 1.9636077544 -2.3969442745 -1.9585815645
C 4.4314477193 -3.3088273403 0.2283294811
H 4.4659744644 -2.7527153928 2.3120000580
H 4.0707841343 -3.7086927400 -1.8584522090
C 5.7224397250 -4.0899784997 0.2879982655
H 5.8422281317 -4.5932516329 1.2532496453
H 5.7691727685 -4.8485816763 -0.4996584286
H 6.5890562177 -3.4283073115 0.1573449357
H -5.0032439859 1.7840978171 1.5288283613
C 1.9423850300 2.7761718755 0.7559303581
H -4.1392894346 1.1014035067 -1.3410729528
H 2.8144685892 2.2234847499 1.1173032598
H 1.7314151889 3.5942934205 1.4539352703
H 2.2001195166 3.2208986116 -0.2132850555
H 1.0098956996 0.9193177354 -1.2524521257
H 0.5190185223 1.3990023059 1.6073357686
H -2.8085520236 3.1919300028 -1.2142335396
41

C -0.5609893200 2.5066988400 -0.0193038119
C -2.0926405264 2.6212949743 0.0688053210
C -2.8384606925 1.4319929858 -0.5514610333
C -4.3557775782 1.5957392699 -0.6281132713
C 0.0719957402 1.3310118190 0.7530695730
N 1.5310092888 1.2430610734 0.5034954632
H -2.3927984657 3.5376046032 -0.4583039244
H -2.4117421970 2.7537956084 1.1084373548
H -2.4819328366 1.2831111079 -1.5815781174
Cl -5.0689479858 1.6905874356 1.0724373666
C -5.0490160878 0.4695577866 -1.3835718362

H -4.8383774222 -0.4998555766 -0.9203594451
H -4.6824528817 0.4451628789 -2.4173729231
H -0.2643549717 2.4161312360 -1.0701292004
H -0.1164409444 3.4396996723 0.3590661342
S 2.0988240227 0.4487891292 -0.8734979780
O 3.4283078888 1.0243692333 -1.1072795079
O 1.0879811550 0.4309666139 -1.9417710851
C 2.2721370698 -1.2404544879 -0.2971075299
C 3.1481399134 -1.5256914041 0.7552115060
C 1.5728927989 -2.2579331376 -0.9414890487
C 3.3083100454 -2.8440140556 1.1647566444
H 3.6912560307 -0.7235053755 1.2439446608
C 1.7462290362 -3.5769162016 -0.5165973058
H 0.9074371738 -2.0140005458 -1.7623271194
C 2.6112907233 -3.8909247677 0.5369044399
H 3.9867598841 -3.0692147842 1.9843024013
H 1.2001331580 -4.3730745594 -1.0162688793
C 2.8044752635 -5.3177801058 0.9918765186
H 2.5917692518 -5.4266993539 2.0621988417
H 2.1500478524 -6.0047107837 0.4471102663
H 3.8394458011 -5.6463499779 0.8351779666
H -6.1325269286 0.6145794574 -1.4047778552
C -0.1477711196 1.4061895225 2.2677790944
H -4.6092366799 2.5635512556 -1.0704482318
H 0.3362460331 0.5608391760 2.7652774392
H -1.2145709342 1.3888036634 2.5128635667
H 0.2754985382 2.3316680558 2.6784506216
H 2.0208818165 2.1345892745 0.5684821719
H -0.3477796988 0.3883434186 0.3860947989
H -2.6178579924 0.5030550523 -0.0106561906

14-Cl

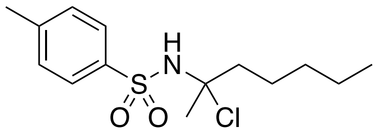

Name	E(B3LYP)	H(B3LYP)	E(RO-B2PLYP-D3)	H(RO-B2PLYP-D3)
Tosyl_NH_C2_Cl_2_heptane_0012	-1610.280807	-1609.912939	-1609.731698	-1609.36383
Tosyl_NH_C2_Cl_2_heptane_0048	-1610.280898	-1609.913117	-1609.7309	-1609.363119
Tosyl_NH_C2_Cl_2_heptane_0031	-1610.279484	-1609.911516	-1609.73093	-1609.362962
Tosyl_NH_C2_Cl_2_heptane_0042	-1610.279392	-1609.911479	-1609.730791	-1609.362878
Tosyl_NH_C2_Cl_2_heptane_0050	-1610.279373	-1609.911452	-1609.730795	-1609.362874
Tosyl_NH_C2_Cl_2_heptane_0051	-1610.279206	-1609.911276	-1609.73074	-1609.36281
Tosyl_NH_C2_Cl_2_heptane_0058	-1610.278486	-1609.910406	-1609.730456	-1609.362376
Tosyl_NH_C2_Cl_2_heptane_0094	-1610.27957	-1609.911714	-1609.730066	-1609.36221
Tosyl_NH_C2_Cl_2_heptane_0028	-1610.278354	-1609.910539	-1609.729794	-1609.361979

41

C -2.4330016127 -1.1293299774 -0.1294368490
C -3.5629700280 -0.1480560359 0.2220460697
C -4.9081803529 -0.5811445571 -0.3776785709
C -6.0595653479 0.3713913296 -0.0300770860
C -1.0393313240 -0.7075902480 0.3727744696
N -0.6438185718 0.4738330001 -0.3221828878
H -3.3050996777 0.8564200302 -0.1357521637
H -3.6645399869 -0.0720162208 1.3124620840
H -5.1571236975 -1.5941611384 -0.0281437909
H -4.8143742490 -0.6522473848 -1.4715680126
H -6.1549617923 0.4397733167 1.0627970111
Cl 0.1054765440 -2.1787841121 -0.1179990629
C -7.3999655251 -0.0592202423 -0.6341690060
H -7.6943995296 -1.0542905221 -0.2784273115
H -7.3462430643 -0.1022717578 -1.7289728759
H -2.6483854029 -2.1186400264 0.2876327130
H -2.3814956554 -1.2607135958 -1.2184823636
S 0.5982964481 1.5864217450 0.0341254374
O 0.4140706651 2.0258377191 1.4160707486
O 0.5133280222 2.5089548686 -1.0996027980
C 2.1711394227 0.7358486078 -0.0631588768
C 2.7231696824 0.4601817129 -1.3167090415
C 2.8389185474 0.3827189957 1.1090707411
C 3.9474508988 -0.1960866025 -1.3868518567
H 2.2108591862 0.7734973000 -2.2203922504
C 4.0661166090 -0.2718977578 1.0184707774
H 2.4119652843 0.6383359183 2.0725976656
C 4.6340549153 -0.5800691976 -0.2243262946
H 4.3824872565 -0.4089244212 -2.3601823783
H 4.5927850876 -0.5430459692 1.9299017305
C 5.9465628986 -1.3202307537 -0.3159169314
H 6.5345348622 -1.2102089771 0.6005705930
H 5.7792535091 -2.3940666172 -0.4721784235
H 6.5516974213 -0.9612557483 -1.1552290488
H -8.2006129286 0.6398870660 -0.3677094587
C -0.9444850070 -0.6031877481 1.8900931263
H -5.8085792579 1.3835343961 -0.3770260916
H -1.3526450009 -1.5147740021 2.3330619192
H -1.5130337688 0.2624693078 2.2393163983
H 0.0863935221 -0.4880558674 2.2244433379
H -0.7847370834 0.4444892735 -1.3282534768
41

C -1.9454830610 -1.0442073659 0.0560958975
C -3.1982533874 -0.1585432167 0.1681038563
C -4.0240929737 -0.1824842019 -1.1251518972
C -5.3032378297 0.6600162748 -1.0405217227
C -1.0042562654 -0.9762368666 1.2663853494
N -0.4328327130 0.3119213790 1.5078861770
H -2.9003413127 0.8718902586 0.3864918916
H -3.8268611543 -0.4983449176 1.0025207909
H -4.2893527585 -1.2207277317 -1.3763492034
H -3.4012341709 0.1860355584 -1.9523808280
H -5.9261090935 0.2929364122 -0.2121019314
Cl 0.3718774836 -2.2769010852 0.9191928666
C -6.1192343329 0.6465934113 -2.3371448254
H -7.0252936147 1.2564523610 -2.2458519021
H -6.4286939965 -0.3723673896 -2.6012015860
H -2.2389186606 -2.0934958177 -0.0553266105
H -1.3904787389 -0.7732284660 -0.8447770581
S 0.2955957055 1.4109321065 0.4237522108
O 0.6232014097 2.5347558720 1.3030409943
O -0.5944830638 1.5518142283 -0.7283374368
C 1.8381861978 0.7135268202 -0.1605983864
C 1.8969674434 0.1236808632 -1.4231244345
C 2.9734719015 0.7969506787 0.6488580391
C 3.1077581871 -0.4016991775 -1.8682796575
H 1.0123071512 0.0938724456 -2.0491615513
C 4.1731014119 0.2627019355 0.1883189167
H 2.9220532511 1.2939057923 1.6120237500
C 4.2588906438 -0.3507889268 -1.0705277268
H 3.1593866881 -0.8565870720 -2.8542038726
H 5.0606561545 0.3304434574 0.8124633293
C 5.5577437206 -0.9553884073 -1.5463905201
H 5.6185082650 -0.9669762470 -2.6392528676
H 6.4220162668 -0.4040109869 -1.1617487574
H 5.6542482127 -1.9936852706 -1.2023801289
H -5.5345426998 1.0423614949 -3.1765606046
C -1.6524739050 -1.4496543744 2.5685746056
H -5.0361596510 1.6950831123 -0.7861269925
H -0.9168662357 -1.4958881778 3.3775355216
H -2.0728717770 -2.4487431330 2.4340318900
H -2.4507457068 -0.7581647099 2.8557299014
H 0.0025368987 0.4184983733 2.4200512747
41

C -2.5678309972 -0.6556034485 -0.1815387107
C -3.5814315881 0.4207915309 0.2446578606
C -4.9606519455 0.2489996577 -0.4151463148
C -5.7216249777 -1.0209099884 -0.0080315228
C -1.1354467979 -0.4144772416 0.3314023473
N -0.6161548872 0.7567176111 -0.2961616958
H -3.1826865616 1.4099652018 -0.0089160104
H -3.7027171073 0.4052143257 1.3358215001
H -4.8464416199 0.2694083937 -1.5093818383
H -5.5743118493 1.1240594526 -0.1596765346
H -5.1519852601 -1.9123072278 -0.3027649112
Cl -0.1551539039 -1.9672752186 -0.2556522449

C -7.1229029740 -1.0960779320 -0.6235258390
H -7.7368562936 -0.2404473088 -0.3166034823
H -7.6458491703 -2.0090012058 -0.3171107369
H -2.8771903791 -1.6427995787 0.1731262054
H -2.5352366113 -0.7184287574 -1.2777917642
S 0.7344282216 1.7109088988 0.1195713969
O 0.5898156631 2.0928232087 1.5231539611
O 0.7537712027 2.6967306084 -0.9627260697
C 2.2090618519 0.7050014718 -0.0229911372
C 2.8323492209 0.2265250764 1.1291255112
C 2.7334820450 0.4354175458 -1.2896337447
C 3.9844451552 -0.5479261784 1.0040026732
H 2.4313386589 0.4779471095 2.1048235578
C 3.8824616471 -0.3413152493 -1.3943968083
H 2.2598955478 0.8448626915 -2.1758271243
C 4.5211534020 -0.8521185874 -0.2535601606
H 4.4768047849 -0.9173356254 1.8999550514
H 4.2960928122 -0.5502170361 -2.3778519242
C 5.7492295988 -1.7202835366 -0.3839223953
H 5.4708492316 -2.7622398141 -0.5897494720
H 6.3900597347 -1.3875315497 -1.2073768977
H 6.3438488626 -1.7154489182 0.5348702300
H -7.0750165080 -1.0917821311 -1.7194529987
C -1.0266700340 -0.4110218752 1.8513187158
H -5.8005022108 -1.0569732044 1.0882053683
H -1.5276004750 -1.2993860421 2.2432976005
H -1.5004671641 0.4871890200 2.2555250799
H 0.0113799179 -0.4249462670 2.1831451204
H -0.7569827149 0.8004883476 -1.3017199214
41

C -1.9490665566 -1.2014111022 -0.0934198708
C -3.2401675220 -0.3667426281 -0.0383976635
C -4.0127298683 -0.4485692407 -1.3633118032
C -5.3687905873 0.2761613350 -1.3507838629
C -1.0567320893 -1.0814177175 1.1495538446
N -0.5522664361 0.2331116488 1.3991679498
H -2.9877688801 0.6742853552 0.1809709052
H -3.8864309257 -0.7241407641 0.7754006424
H -4.1791835114 -1.5055322301 -1.6176156187
H -3.3864274932 -0.0323845141 -2.1651585867
H -5.8865843811 0.0598171296 -2.2950314981
Cl 0.3866275595 -2.3227184467 0.8658960231
C -5.2741199614 1.7960993522 -1.1716045118
H -4.6459823363 2.2461377762 -1.9500737750
H -4.8411870630 2.0684671620 -0.2027932901
H -2.1948161110 -2.2631609340 -0.2027742643
H -1.3727855724 -0.9169577313 -0.9765905234
S 0.1754756242 1.3499743963 0.3330155868
O 0.4026036492 2.5028324495 1.2063960865
O -0.6607668863 1.4270944047 -0.8645485099
C 1.7785274869 0.7213678307 -0.1596122460
C 1.9308798548 0.1141419156 -1.4060957854
C 2.8654778852 0.8758996016 0.7037439996
C 3.1873236116 -0.3564177452 -1.7798887022
H 1.0817762320 0.0288261148 -2.0747487387
C 4.1122541626 0.3953964027 0.3142059091
H 2.7405984756 1.3860818418 1.6531875185
C 4.2924769328 -0.2338956955 -0.9265622943
H 3.3117096272 -0.8245122183 -2.7530780587
H 4.9623890094 0.5186024113 0.9805032243
C 5.6419947211 -0.7802294047 -1.3257278438
H 5.7701792559 -0.7782686544 -2.4129017576
H 6.4575033481 -0.1981969759 -0.8842485976
H 5.7596573907 -1.8174061871 -0.9848946292
H -6.2651615308 2.2605952109 -1.2313844154
C -1.7314667905 -1.5717852733 2.4319211005
H -5.9989914792 -0.1487814937 -0.5558788544
H -2.1018993827 -2.5897648627 2.2923765605
H -2.5698996759 -0.9142844780 2.6821466936
H -1.0252219113 -1.5780255954 3.2679169531
H -0.1624851133 0.3684420762 2.3279881771
41

C -2.0821755431 -0.6183062852 0.2725243400
C -3.2215719894 0.4103311010 0.3980160591
C -4.0844176523 0.5023548799 -0.8714541461
C -4.8601827122 -0.7739796932 -1.2265998010
C -1.0695128745 -0.5808458536 1.4252048824
N -0.3466287104 0.6465835548 1.5480203542
H -2.7930163407 1.3964134460 0.6002223729
H -3.8640939742 0.1550175561 1.2520491724
H -3.4433834328 0.7915870041 -1.7161726849
H -4.8014259191 1.3249373659 -0.7398280058
H -4.1606547372 -1.5990361927 -1.4171687917
Cl 0.1337263985 -2.0477729724 1.0994065657
C -5.7678197300 -0.5956223273 -2.4483815735
H -6.3106719310 -1.5183370274 -2.6832563220
H -5.1862701996 -0.3158412646 -3.3354149466
H -2.4844192581 -1.6352160081 0.2479156744
H -1.5559441538 -0.4611918547 -0.6719649501
S 0.4329180443 1.5853866613 0.3544841026
O 0.9361349016 2.7161900271 1.1364987717
O -0.5040111639 1.7560520623 -0.7556462846
C 1.8499565231 0.6800475487 -0.2617110592
C 3.0326690933 0.6772169964 0.4813337314
C 1.7679670070 0.0157608086 -1.4853718465
C 4.1352116079 -0.0188825731 -0.0050319061
H 3.0942622784 1.2314002654 1.4121473413
C 2.8834557747 -0.6718188632 -1.9576351346
H 0.8506830607 0.0536851045 -2.0620262893
C 4.0774943291 -0.7096167807 -1.2248280105
H 5.0595911914 -0.0192022535 0.5671597835
H 2.8257658930 -1.1854118681 -2.9139585576
C 5.2694437862 -1.4878847962 -1.7273682989
H 5.2703216318 -1.5597524870 -2.8196551875
H 6.2115639234 -1.0260445389 -1.4143739551
H 5.2615515944 -2.5123900851 -1.3323333132
H -6.5096843823 0.1944076908 -2.2783993492
C -1.6898087720 -0.8945613412 2.7878201595
H -5.4667199953 -1.0813549321 -0.3619447173
H -2.3908306756 -0.1009658092 3.0648154485
H -0.9188114514 -0.9711636759 3.5607351716
H -2.2232371190 -1.8467005939 2.7442535570
H 0.1500636900 0.7604820079 2.4273506240
41

C -2.0923951532 -0.6356601789 0.1613944902
C -3.2324182465 0.3816459133 0.3551522297
C -4.0612970246 0.5989553210 -0.9216645977
C -4.8296740825 -0.6339793869 -1.4180619938
C -1.1083658798 -0.7108108730 1.3368449221
N -0.3867945292 0.4970049455 1.5921431837
H -2.8071426893 1.3418324010 0.6619264070
H -3.8977444984 0.0488748358 1.1638606293
H -3.3975428734 0.9651566664 -1.7175542887
H -4.7800299977 1.4082135917 -0.7303631088
H -4.1267994493 -1.4400227640 -1.6683110841
Cl 0.1000333706 -2.1449640476 0.9043271370
C -5.7038885415 -0.3352501470 -2.6407182408
H -6.2419014896 -1.2287871753 -2.9774552807
H -5.0982367774 0.0258317422 -3.4810247089
H -2.4959335164 -1.6440433237 0.0316362116
H -1.5427104180 -0.3928604205 -0.7510341979
S 0.4245788171 1.5398738448 0.5116840122
O 0.9077362841 2.5918545496 1.4080096659
O -0.4826594296 1.8154177028 -0.6020315615
C 1.8575342961 0.6928412810 -0.1490501573
C 1.8057980237 0.1399598958 -1.4284287524
C 3.0197285136 0.6164532544 0.6220650477
C 2.9317336908 -0.5079988544 -1.9311294720
H 0.9014356393 0.2265252203 -2.0200381712
C 4.1329883822 -0.0385115852 0.1042445392
H 3.0553263070 1.0774695726 1.6036587386
C 4.1079858533 -0.6111541903 -1.1763701798
H 2.8953360146 -0.9410819345 -2.9275163986
H 5.0392229935 -0.1019987779 0.7014631234
C 5.3318949363 -1.2923957777 -1.7396191555
H 5.0631666441 -2.0434858990 -2.4890768242
H 5.9958891314 -0.5661639754 -2.2270157221

H 5.9124525654 -1.7866404435 -0.9537491725
H -6.4486331877 0.4380512012 -2.4153956038
C -1.7629374927 -1.1483266114 2.6483604599
H -5.4597524559 -1.0201649901 -0.6032863148
H -2.2966305186 -2.0900939042 2.5025391108
H -2.4693995143 -0.3814393954 2.9811562514
H -1.0115672889 -1.3000741039 3.4294655046
H 0.0875218805 0.5260983162 2.4905197831
41

C -2.1080323292 -0.9028058414 0.5022667042
C -3.3098796129 0.0442719691 0.6674054769
C -4.3941926846 -0.1687764142 -0.4041718050
C -3.9705110009 0.1805468993 -1.8383863568
C -1.0213166067 -0.7470957738 1.5756949272
N -0.3940194787 0.5371161981 1.6139178971
H -2.9626010858 1.0817542009 0.6318628203
H -3.7670607248 -0.1085122668 1.6534597428
H -5.2621250882 0.4499048696 -0.1351171391
H -4.7458915501 -1.2113477896 -0.3706501202
H -3.5534609022 1.1959449636 -1.8542095865
Cl 0.2690769600 -2.1262440808 1.1966155294
C -5.1289349530 0.0775459435 -2.8362070130
H -5.9437957251 0.7624179517 -2.5702376846
H -5.5459370125 -0.9372658365 -2.8594015440
H -2.4469484093 -1.9436895330 0.5502989257
H -1.6558328865 -0.7611519242 -0.4804363424
S 0.2398767701 1.4905704883 0.3477574358
O 0.6757022234 2.6930342192 1.0601253359
O -0.7594640575 1.5314386136 -0.7194594169
C 1.7027119409 0.6900310283 -0.3055344482
C 2.9162348187 0.8314419771 0.3713913969
C 1.6222353194 -0.0363790461 -1.4935437917
C 4.0538294748 0.2176188552 -0.1443985185
H 2.9719337027 1.4329835713 1.2727059570
C 2.7725339849 -0.6396586077 -1.9962881993
H 0.6775043352 -0.1111099795 -2.0201056344
C 4.0002820798 -0.5323841610 -1.3287869370
H 5.0017715054 0.3297667032 0.3758949505
H 2.7155866535 -1.2012557250 -2.9252759716
C 5.2335829721 -1.2203455839 -1.8623412690
H 5.3394728267 -2.2235892360 -1.4287208393
H 5.1885524510 -1.3384780460 -2.9496752303
H 6.1428845768 -0.6622100122 -1.6164319166
H -4.8039210876 0.3266109969 -3.8528174677
C -1.5075594406 -1.0681610883 2.9904243542
H -3.1582838845 -0.4829953110 -2.1628365176
H -1.9691564094 -2.0578363076 3.0101607108
H -2.2416969077 -0.3214974644 3.3085134314
H -0.6737185111 -1.0674388075 3.6991607887
H 0.1428071100 0.7208068909 2.4571706138
41

C -1.9694438595 -0.1663746706 0.7606189316
C -1.9750716489 -0.0351972049 -0.7727764314
C -2.2861776108 -1.3728745936 -1.4583830798
C -2.3025129762 -1.2794120981 -2.9893731897
C -1.5540701052 1.1038430205 1.5172762040
N -0.1834902045 1.4903812134 1.2996348192
H -1.0058650187 0.3372178431 -1.1245132232
H -2.7244954969 0.7054019609 -1.0819886080
H -3.2580412451 -1.7486666198 -1.1055464564
H -1.5405054811 -2.1199587601 -1.1502785952
H -3.0388694791 -0.5243895661 -3.2996719728
Cl -1.8704087155 0.7509896119 3.3580093772
C -2.6224011986 -2.6138588912 -3.6707476764
H -1.8843254661 -3.3811864209 -3.4062006098
H -2.6242598852 -2.5157516563 -4.7623448438
H -2.9743305919 -0.4290497737 1.1070744240
H -1.3144150038 -0.9855403726 1.0713404034
S 1.2006035853 0.4937994680 1.5103393520
O 0.8742328318 -0.7267176767 2.2468568777
O 2.2092982913 1.4456653072 1.9848656277
C 1.6325551407 0.0124377728 -0.1621519417
C 1.6189064548 -1.3372125325 -0.5075773154
C 2.0371307600 0.9862604197 -1.0799528635
C 1.9988025707 -1.7111857848 -1.7978729077
H 1.3202677806 -2.0774098963 0.2267197704
C 2.4081992791 0.5954482531 -2.3617775475
H 2.0617553049 2.0322612062 -0.7922256398
C 2.3966711950 -0.7573151311 -2.7421405077
H 1.9892685666 -2.7633378838 -2.0708303089
H 2.7186924945 1.3502970119 -3.0801452168
C 2.8320384303 -1.1671224951 -4.1286265199
H 3.9250342859 -1.1296771744 -4.2235246041
H 2.5157024971 -2.1878643090 -4.3635344601
H 2.4193104871 -0.4981860364 -4.8918973418
H -3.6084358971 -2.9874117871 -3.3685029400
C -2.4291879976 2.3180269345 1.1974729249
H -1.3256949461 -0.9143365476 -3.3367465565
H -3.4806995461 2.0752709414 1.3685474400
H -2.2920007288 2.6130633693 0.1525877015
H -2.1678936921 3.1649093559 1.8392501279
H 0.0508811448 2.3802326107 1.7361233673
41

C -2.1976661927 -0.9972922278 0.4390254262
C -3.4052381596 -0.0559714513 0.5980389179
C -4.5784462773 -0.4304209235 -0.3282385319
C -4.3236312101 -0.3054273674 -1.8407677192
C -1.0928290240 -0.8069005895 1.4894702988
N -0.4923000414 0.4913041086 1.5091076503
H -3.0889098637 0.9750835269 0.4149498049
H -3.7729804038 -0.0935778107 1.6312328921
H -5.4348415942 0.2070605048 -0.0660839210
H -4.8887364164 -1.4615770451 -0.1038657632
H -3.4903051983 -0.9573008390 -2.1364882704
Cl 0.2157767046 -2.1639777800 1.0999032152
C -4.0483439588 1.1250808483 -2.3192126374
H -3.1175090779 1.5218006610 -1.9028057160
H -4.8649631301 1.7993964702 -2.0302441930
H -2.5264014769 -2.0392414923 0.5254215156
H -1.7652924654 -0.8819474352 -0.5561768004
S 0.1475442873 1.4249564961 0.2329872571
O 0.4805548400 2.6820058331 0.9046760620
O -0.8001697600 1.3568783040 -0.8793276871
C 1.6847709935 0.6870638329 -0.3153712669
C 2.8574769199 0.9512761017 0.3956509610
C 1.7011872880 -0.1111766873 -1.4592878203
C 4.0539657610 0.3889987676 -0.0403819617
H 2.8361990305 1.6075802914 1.2594391081
C 2.9087748743 -0.6604304739 -1.8825604981
H 0.7853467458 -0.2841578594 -2.0128530000
C 4.0988915176 -0.4293675926 -1.1786578758
H 4.9704055013 0.5969872290 0.5061666998
H 2.9271993357 -1.2775275292 -2.7772701531
C 5.3958661326 -1.0593138225 -1.6258824253
H 6.2574800417 -0.4410110098 -1.3540158982
H 5.5363374791 -2.0408549957 -1.1540731196
H 5.4141603407 -1.2137120541 -2.7095235269
H -3.9626273015 1.1600360761 -3.4116291516
C -1.5463266733 -1.1253662389 2.9161881504
H -5.2068133698 -0.6970043284 -2.3639450821
H -0.6983487215 -1.1065237940 3.6077008104
H -1.9911012019 -2.1221718711 2.9515874833
H -2.2862713422 -0.3892899032 3.2451145857
H 0.0238196186 0.7049960351 2.3582849804
41

C -2.3613345574 -1.8435213254 0.2010775242
C -3.6937382912 -1.1240634251 0.4994390125
C -4.0611382306 0.0544221499 -0.4136593673
C -5.4715577209 0.5950769644 -0.1415318210
C -1.0664793668 -1.1060325987 0.5996929714
N -0.8728438975 0.0145834339 -0.2569951369
H -3.7173568776 -0.8004947878 1.5482503896
H -4.4790565083 -1.8879431459 0.4105758982
H -3.9969925621 -0.2688705724 -1.4643953207
H -3.3335344105 0.8654198458 -0.2969183468
H -6.2034727906 -0.2164679785 -0.2635923219

Cl 0.3210836651 -2.4204055426 0.2869038210
C -5.8501273457 1.7678490429 -1.0515748473
H -6.8593099319 2.1342750432 -0.8317416406
H -5.8253255754 1.4737537108 -2.1081511643
H -2.3464562414 -2.7983271649 0.7352343363
H -2.3048085003 -2.0921262046 -0.8669943724
S 0.1915732534 1.3399110521 -0.1026116899
O -0.0113637972 1.9139848482 1.2264796515
O -0.0814841433 2.0900045890 -1.3299835487
C 1.8750170370 0.7366830254 -0.1871655630
C 2.6288955001 0.6273263267 0.9810712540
C 2.4215819565 0.4086458961 -1.4302509605
C 3.9409349842 0.1650972643 0.8990315078
H 2.1987711552 0.9210256235 1.9322036245
C 3.7321209582 -0.0536264439 -1.4915084278
H 1.8359767226 0.5339126540 -2.3350210300
C 4.5092639855 -0.1911861651 -0.3309389485
H 4.5340995953 0.0845959234 1.8063848225
H 4.1631283292 -0.3059305148 -2.4571348024
C 5.9192071455 -0.7250206599 -0.4077664111
H 6.4196518821 -0.4055549217 -1.3279236112
H 6.5219416326 -0.3910656524 0.4426064125
H 5.9217529358 -1.8229944947 -0.4005563643
H -5.1550138327 2.6065987835 -0.9260094608
C -0.9845317679 -0.7810657981 2.0860543129
H -5.5403673688 0.9110220408 0.9092295785
H -1.2278272038 -1.6788845272 2.6590717558
H -1.6921108534 0.0151001449 2.3321068979
H 0.0116021334 -0.4455393572 2.3731077501
H -1.0389284199 -0.1654586925 -1.2430791948

$12_{pyrrolidine}$

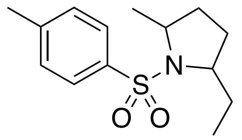

Name	E(B3LYP)	H(B3LYP)	E(RO-B2PLYP-D3)	H(RO-B2PLYP-D3)
Tosyl_2_ethyl_6_methyl_pyrrolidine_0000	-1149.475445	-1149.121877	-1149.036616	-1148.683048
Tosyl_2_ethyl_6_methyl_pyrrolidine_0016	-1149.476007	-1149.122407	-1149.036253	-1148.682653
Tosyl_2_ethyl_6_methyl_pyrrolidine_0010	-1149.475776	-1149.12216	-1149.036117	-1148.682501
Tosyl_2_ethyl_6_methyl_pyrrolidine_0054	-1149.474748	-1149.121028	-1149.035854	-1148.682134
Tosyl_2_ethyl_6_methyl_pyrrolidine_0088	-1149.474373	-1149.120586	-1149.03543	-1148.681643
Tosyl_2_ethyl_6_methyl_pyrrolidine_0025	-1149.474365	-1149.120944	-1149.0328	-1148.679379
Tosyl_2_ethyl_6_methyl_pyrrolidine_0035	-1149.474538	-1149.121061	-1149.032841	-1148.679364
Tosyl_2_ethyl_6_methyl_pyrrolidine_0006	-1149.473299	-1149.119857	-1149.032772	-1148.67933
Tosyl_2_ethyl_6_methyl_pyrrolidine_0058	-1149.473979	-1149.120508	-1149.032212	-1148.678741
Tosyl_2_ethyl_6_methyl_pyrrolidine_0042	-1149.473717	-1149.120201	-1149.032016	-1148.678521

39

C -2.1792941894 -0.3176308730 2.2480223278
C -1.7547082406 1.0925357020 1.8246653835
C -3.0928344290 1.4305712812 -0.3759664452
C -1.7625462143 1.0335125531 0.2841057114
C -1.4928150193 -1.2484442067 1.2322209328
N -1.4455054906 -0.4017404408 0.0022876213
H -0.7398727916 1.3041567280 2.1823038839
H -2.4133274123 1.8736554800 2.2160983320
H -1.8953118763 -0.5660448860 3.2754309644
H -3.2674541330 -0.4315498593 2.1699239256
S -0.3150231728 -0.7301625875 -1.1903992299
O -0.6281748227 0.1635901834 -2.3093735738
O -0.2740967165 -2.1866558158 -1.3493490314
C 1.2938877748 -0.2320384180 -0.5539787203
C 2.0530685178 -1.1368547885 0.1944134776
C 1.7673074048 1.0605390156 -0.7948271109
C 3.2792411799 -0.7314965295 0.7176374333
H 1.6970920208 -2.1516666150 0.3362222862
C 2.9964433839 1.4484982827 -0.2640194362
H 1.1904375066 1.7383499398 -1.4148539887
C 3.7697820392 0.5641486788 0.5006968291
H 3.8705869558 -1.4372586013 1.2962923473
H 3.3660503155 2.4529808494 -0.4561498175
C 5.1162429662 0.9815164821 1.0426126733
H 5.3373401978 0.4853872183 1.9934796036
H 5.9205757284 0.7170427902 0.3434371592
H 5.1667226486 2.0633725308 1.2025441710
H -3.0461969285 1.1080328094 -1.4210794975
H -0.4673584078 -1.4541631870 1.5757879177
C -2.2331926979 -2.5679943400 1.0213424842
C -3.3777225391 2.9349229509 -0.3111611358
H -0.9741832120 1.6728921076 -0.1281299245
H -2.5825746494 3.5085775579 -0.8034529828
H -3.4603264055 3.2986044118 0.7201642960
H -4.3186729755 3.1735024472 -0.8186064845
H -1.7184603823 -3.1992012789 0.2952144056
H -3.2479917658 -2.3767303116 0.6555817074
H -2.3032696865 -3.1081406485 1.9737607535
H -3.9125176785 0.8678845451 0.0912055327
39

C -1.4996832781 -0.2990481359 2.3756751013
C -2.3197747557 0.8556813307 1.7895541708
C -2.9186004749 1.5009080960 -0.6346963114
C -1.8484252574 0.9646040033 0.3249939469
C -1.4074755696 -1.3116369697 1.2190578029
N -1.4440393073 -0.4385957120 0.0053819049
H -2.1817977435 1.7956950373 2.3314298018
H -3.3903764604 0.6167588151 1.8123240116
H -0.4946690760 0.0503420111 2.6413937968
H -1.9510170717 -0.7345219870 3.2729262838
S -0.3141447072 -0.7297168634 -1.1970086317
O -0.6262587710 0.1783239076 -2.3046544350
O -0.2716126282 -2.1842295708 -1.3740596152
C 1.2927130711 -0.2307115732 -0.5558326581
C 2.0996392709 -1.1635975157 0.1016064229
C 1.7163513220 1.0933372230 -0.7032583406
C 3.3237622794 -0.7571699290 0.6294631698
H 1.7811192649 -2.1983583404 0.1689746014
C 2.9433359664 1.4820326430 -0.1687090406
H 1.1033125441 1.7972773558 -1.2557448180
C 3.7644434863 0.5681570999 0.5067034260
H 3.9527593372 -1.4850079720 1.1366177018
H 3.2735521771 2.5113224247 -0.2872794555
C 5.1082938671 0.9902295362 1.0514218109
H 5.1157978506 2.0505869436 1.3240345122
H 5.3829670780 0.4082460106 1.9373099001
H 5.8997082486 0.8399822981 0.3051977702
H -2.5386765963 1.4308238805 -1.6570073792
H -0.4554447929 -1.8497881775 1.2543238274
C -2.5419929525 -2.3378180206 1.1815258267
C -3.3216001846 2.9473369570 -0.3248675001
H -0.9646317562 1.6203850052 0.2816384973
H -4.0552488229 3.3077739885 -1.0538998581
H -2.4559029444 3.6204945180 -0.3698025352
H -3.7696001620 3.0479104658 0.6708425224
H -2.4582976707 -2.9495915143 0.2793334031
H -3.5220185164 -1.8476366169 1.1744836460
H -2.4908853722 -2.9955063132 2.0580153938
H -3.7943750793 0.8410798285 -0.5756040973
39

C -1.8608184957 -0.2636213044 2.3908435489
C -1.4326392334 1.1451309356 1.9598754013
C -3.2041559141 1.5791637174 0.1510146967
C -1.7512260029 1.1686646507 0.4527643267
C -1.4630947967 -1.1701181135 1.2068888674
N -1.4775679833 -0.2396395537 0.0324203875
H -0.3546227432 1.2767649345 2.1120286489
H -1.9494960212 1.9384219352 2.5094788920
H -1.3879879026 -0.5927531791 3.3214098417
H -2.9457519817 -0.3062627095 2.5434634233
S -0.3634789424 -0.5186446561 -1.1944541142
O -0.6324801618 0.4733303442 -2.2388034149
O -0.4009734246 -1.9598128646 -1.4620655262
C 1.2753364817 -0.1569522340 -0.5397351426

C 2.0256823172 -1.1750509779 0.0549522451
C 1.7825170058 1.1435857786 -0.6177820320
C 3.2775374281 -0.8779543601 0.5917309279
H 1.6413138705 -2.1892609620 0.0676498478
C 3.0352737473 1.4223932089 -0.0755646665
H 1.2132675067 1.9158861415 -1.1239542103
C 3.8011568314 0.4210019247 0.5390434452
H 3.8616695732 -1.6718732817 1.0510478291
H 3.4305685856 2.4333647595 -0.1408774748
C 5.1728927875 0.7267123251 1.0918294881
H 5.9427051275 0.6152810797 0.3167755723
H 5.2351984004 1.7541349245 1.4653888821
H 5.4356081516 0.0493648591 1.9108474351
H -3.8857949907 0.9206278797 0.7059009989
H -0.4365644074 -1.5275173037 1.3668633055
C -2.3842879407 -2.3716039199 1.0058492497
C -3.5756498262 1.5706348397 -1.3329784835
H -1.0839772466 1.8438465201 -0.0909498643
H -2.9390456444 2.2516306443 -1.9079187209
H -4.6180588181 1.8812224521 -1.4680781989
H -3.4544087445 0.5732213541 -1.7638126148
H -2.3768496011 -3.0042119295 1.9023038261
H -2.0596181806 -2.9690698601 0.1512909067
H -3.4125364855 -2.0383724041 0.8266020858
H -3.3467909507 2.5886992492 0.5627559535
39

C -2.7629684929 -1.1546674398 1.6431840950
C -3.5491574574 -0.6197631528 0.4405160304
C -2.7523467255 0.5325941580 -1.7362390387
C -2.5977901430 0.4000430354 -0.2179412162
C -1.3232579325 -1.2573627267 1.1095192200
N -1.2279011573 -0.1094876970 0.1507798717
H -4.5001411766 -0.1592201518 0.7237087195
H -3.7748313437 -1.4302266859 -0.2641624864
H -2.7928518845 -0.4309579480 2.4658509902
H -3.1426285911 -2.1112521333 2.0172630408
S -0.1020983648 1.0899358019 0.5521468362
O -0.0515669915 1.3288972838 2.0050219058
O -0.3683948885 2.2147790790 -0.3532727045
C 1.4492816306 0.3135636558 0.0925667732
C 2.4531940976 0.1816275473 1.0493014378
C 1.6611756903 -0.0889327909 -1.2298599217
C 3.6799468164 -0.3711538578 0.6766997987
H 2.2683536006 0.5074225318 2.0671880444
C 2.8886096281 -0.6387151802 -1.5829339384
H 0.8707638276 0.0217479754 -1.9648628760
C 3.9180857863 -0.7871010427 -0.6379202813
H 4.4637243950 -0.4785557208 1.4223847029
H 3.0549421229 -0.9573161026 -2.6093604367
C 5.2513155827 -1.3685422079 -1.0439923975
H 5.8769429555 -1.5845906079 -0.1727360552
H 5.1260421006 -2.2985059381 -1.6106378026
H 5.8056608570 -0.6725496370 -1.6866050102
H -1.9645690431 1.1982826270 -2.1027475271
H -0.6025444269 -1.1081566121 1.9181196153
C -1.0165987677 -2.5797919574 0.4001957890
C -4.1222157519 1.0808392524 -2.1521573145
H -2.7548000309 1.3860531435 0.2369753957
H -4.1799207488 1.1977151981 -3.2398119183
H -4.3064967425 2.0650479418 -1.7042304940
H -4.9398572715 0.4166836756 -1.8477775335
H -1.0535548967 -3.4108316787 1.1149311434
H -0.0183006094 -2.5541899081 -0.0471532885
H -1.7378563842 -2.7844499768 -0.3987333310
H -2.5830680073 -0.4525831733 -2.1923334459
39

C -1.5086602240 -0.3372845426 2.4314372277
C -2.2342408550 0.9258295503 1.9553596508
C -2.6260779184 1.9759767281 -0.3814058463
C -1.6909852527 1.1643904034 0.5340331579
C -1.4438425973 -1.2241843758 1.1727034992
N -1.4196293775 -0.2258332206 0.0570159836
H -2.0556351221 1.7945333721 2.5967053031
H -3.3158624160 0.7566274062 1.9233758350
H -0.4929820897 -0.0856091200 2.7598888268
H -2.0145314312 -0.8387219808 3.2626215119
S -0.3381435711 -0.4859666665 -1.1955193823
O -0.6140493215 0.5322170994 -2.2132004031
O -0.3984279655 -1.9192320605 -1.4982995094
C 1.3112698908 -0.1520857514 -0.5563045212
C 1.8196075436 1.1497288741 -0.5949786148
C 2.0665001629 -1.1916991154 -0.0066702309
C 3.0808388381 1.4069905363 -0.0609986701
H 1.2437456500 1.9400851407 -1.0644131071
C 3.3261884787 -0.9159382831 0.5221794399
H 1.6797051641 -2.2050160184 -0.0229756206
C 3.8525854930 0.3833082889 0.5068560264
H 3.4769330664 2.4191775873 -0.0949861924
H 3.9150899106 -1.7264582466 0.9450055580
C 5.2329655419 0.6660457159 1.0503438696
H 5.3060934672 1.6824660942 1.4509890650
H 5.5018537660 -0.0349510064 1.8472074295
H 5.9923969458 0.5713385564 0.2629081726
H -2.8318308424 2.9231165479 0.1383463795
H -0.5175047591 -1.8070982558 1.1632019292
C -2.6232052802 -2.1858417716 1.0151326668
C -3.9409987482 1.2898587649 -0.7618756803
H -0.7383613898 1.7107821582 0.6190369218
H -4.5317319299 1.9446553374 -1.4119092482
H -4.5607155010 1.0529379823 0.1110946291
H -3.7490989821 0.3599734842 -1.3047097025
H -3.5789294410 -1.6511408035 1.0488785073
H -2.6154218648 -2.9320987561 1.8191721708
H -2.5535120929 -2.7049568007 0.0556198131
H -2.0823188960 2.2287404525 -1.2953650666
39

C -3.7323366388 -0.3806957369 -0.0349582754
C -3.2000995341 0.4815695539 1.1146567534
C -1.5984180362 2.0349927412 -0.2013476672
C -1.7522083061 0.7884582825 0.6873350988
C -2.5466165983 -1.2865738715 -0.4130696077
N -1.3469933986 -0.4449842654 -0.0629929929
H -3.1886261409 -0.0955755839 2.0464414095
H -3.7934516943 1.3850299556 1.2862793777
H -4.6127757578 -0.9705493022 0.2388802889
H -4.0109815239 0.2464018830 -0.8911105771
S -0.0347006419 -1.3013709470 0.5793252332
O -0.0022180984 -2.5854227377 -0.1318668789
O -0.0454631513 -1.2893286638 2.0522952352
C 1.3628305044 -0.3046737618 0.0557757661
C 1.6161385178 -0.1271163517 -1.3080928837
C 2.2177998077 0.2295601760 1.0169708213
C 2.7309052670 0.6051454918 -1.7009732122
H 0.9444521682 -0.5538900768 -2.0456682345
C 3.3331929498 0.9611774811 0.6043674339
H 2.0068610523 0.0697729534 2.0688203671
C 3.6080031328 1.1596513897 -0.7531507435
H 2.9286729239 0.7485039200 -2.7607178826
H 4.0003960050 1.3817542162 1.3526040881
C 4.8241823651 1.9360698033 -1.1987532767
H 4.5630681813 2.6926171649 -1.9478430358
H 5.3059911516 2.4442790580 -0.3580724811
H 5.5690220031 1.2731190902 -1.6573501115
H -0.6012652619 2.0054140764 -0.6553700905
H -2.5623180892 -2.1814521188 0.2206055730
C -2.5307133680 -1.7160005284 -1.8771815423
C -1.7734824939 3.3506711690 0.5655831170
H -1.1090522809 0.9003672580 1.5659084868
H -1.6267326542 4.2104580781 -0.0972672218
H -1.0447644300 3.4321567547 1.3816016146
H -2.7735366198 3.4423545318 1.0050078973
H -3.4464942558 -2.2705151688 -2.1167319096
H -1.6744000117 -2.3635570458 -2.0798253886
H -2.4767562873 -0.8394707153 -2.5331841577
H -2.3149346138 1.9854604975 -1.0322496336
39

C -2.1821204889 -0.1083674710 2.2360212564
C -1.7313658329 1.3017485084 1.8389148296
C -2.8012370491 1.8655281040 -0.4781813481
C -1.6152010021 1.2665467689 0.2986152968
C -1.4931326923 -1.0403902987 1.2250182020
N -1.4244129743 -0.1900848116 -0.0031711806
H -0.7490022797 1.5174271146 2.2752163003
H -2.4199652741 2.0816339283 2.1792523786
H -1.9142642577 -0.3716054537 3.2642896580
H -3.2686838434 -0.2099461989 2.1406947706
S -0.3126014891 -0.5769390405 -1.2031403860
O -0.5576586500 0.3557975747 -2.3071760239
O -0.3716807382 -2.0288078942 -1.3931984817
C 1.3245453768 -0.2031059004 -0.5536132067
C 2.0160402825 -1.1705286125 0.1817230862
C 1.8896072778 1.0557889693 -0.7756935315
C 3.2674532196 -0.8614138934 0.7111586199
H 1.5887585033 -2.1594485303 0.3086632798
C 3.1424965059 1.3471924664 -0.2389112090
H 1.3635361818 1.7814914102 -1.3865883398
C 3.8495601634 0.3987705075 0.5132391560
H 3.8060705199 -1.6158938655 1.2796552353
H 3.5831461490 2.3253847193 -0.4163219069
C 5.2216264005 0.7116915163 1.0611825349
H 6.0044501248 0.4315291123 0.3439399302
H 5.3372130516 1.7807526916 1.2672261830
H 5.4186569036 0.1623313295 1.9876256417
H -2.8218526515 2.9429247528 -0.2584263502
H -0.4727726795 -1.2555910037 1.5793463033
C -2.2446652917 -2.3534430456 1.0125344738
C -4.1753114045 1.2546159093 -0.1913926693
H -0.7212015747 1.8211194447 -0.0082549421
H -4.9372372650 1.7194451938 -0.8268639539
H -4.4867502691 1.4017733777 0.8498750688
H -4.1778877213 0.1800349829 -0.4018536803
H -2.3308692066 -2.8835923375 1.9691604174
H -1.7314137247 -2.9975546347 0.2973390706
H -3.2540388021 -2.1549659223 0.6358896645
H -2.5732043918 1.7658459967 -1.5443574835
39

C -3.0840437396 -1.4271263897 -0.8848638658
C -3.7121579092 -0.2560990332 -0.1227889540
C -2.4117630086 1.8403530249 -0.9187367540
C -2.5388220192 0.7171868439 0.1213218164
C -1.7153821911 -1.5990131859 -0.2160546169
N -1.3309303578 -0.1824952735 0.0704957368
H -4.1117427537 -0.6060734239 0.8360837174
H -4.5337949134 0.2177504770 -0.6680897702
H -3.6758102066 -2.3466629180 -0.8332762697
H -2.9494699966 -1.1750746136 -1.9445520250
S -0.1509764448 0.0709033579 1.2558958392
O -0.1458940141 -1.0368165942 2.2248322892
O -0.3204012172 1.4544553773 1.7200481423
C 1.3984351128 -0.0244597661 0.3498002116
C 2.4139804669 -0.8426478049 0.8410874806
C 1.6061126859 0.7731834798 -0.7786052786
C 3.6464946078 -0.8667771244 0.1861175302
H 2.2323026631 -1.4532435717 1.7189008087
C 2.8397054869 0.7357309004 -1.4198738689
H 0.8071719155 1.4029046158 -1.1556480360
C 3.8805115817 -0.0812061604 -0.9481380760
H 4.4380025791 -1.5082234682 0.5656355866
H 3.0004145012 1.3502988341 -2.3026485293
C 5.2204482061 -0.0941180673 -1.6445901439
H 5.1062535283 -0.1906687022 -2.7304104917
H 5.7696486640 0.8382421295 -1.4602563341
H 5.8462740020 -0.9208100461 -1.2949858284
H -1.4365523874 2.3189914458 -0.7732934143
H -1.8401619179 -2.1367255271 0.7336950380
C -0.6959340564 -2.3384745802 -1.0795263143
C -3.5098187902 2.9034702701 -0.8025857953
H -2.6149921136 1.1772947959 1.1098694640
H -4.5095379238 2.4861510561 -0.9712618617
H -3.3591933575 3.7001130411 -1.5393402885
H -3.5050763514 3.3654773270 0.1919552311
H -0.5022754311 -1.7882966587 -2.0067624602
H -1.0860288483 -3.3302326420 -1.3374147282
H 0.2549228501 -2.4814703035 -0.5588855783
H -2.4058795051 1.4048499132 -1.9275847599
39

C -3.7129452968 -0.9641773525 -0.0931756947
C -3.4643181343 0.5307223782 -0.3182328678
C -1.3464873465 2.0007806470 -0.2087367917
C -2.0912709522 0.7724961581 0.3265776428
C -2.3330477232 -1.6163913649 -0.3086159384
N -1.3742236474 -0.5101102388 0.0387004728
H -4.2386896889 1.1646166089 0.1233796394
H -3.4172108301 0.7598925271 -1.3908820079
H -4.0479666330 -1.1353007670 0.9366399370
H -4.4716642354 -1.3804354666 -0.7636179645
S -0.0697025213 -0.9109566214 1.0366785768
O 0.0838300379 -2.3699131187 0.9577060587
O -0.2046629772 -0.2504021921 2.3445622243
C 1.3503272140 -0.1657291591 0.2241802609
C 1.6197114513 -0.4429047669 -1.1187859120
C 2.2188541374 0.6219573585 0.9772351147
C 2.7635911654 0.0865688320 -1.7062335130
H 0.9354913015 -1.0543966070 -1.6975246332
C 3.3633686225 1.1443280838 0.3720098405
H 1.9921240819 0.8239797467 2.0184406327
C 3.6551744218 0.8861903297 -0.9719290463
H 2.9707703178 -0.1226132048 -2.7532136642
H 4.0394102715 1.7626164056 0.9573741234
C 4.9016441507 1.4385311389 -1.6214877536
H 5.6252838102 0.6400210601 -1.8297911914
H 4.6731994262 1.9226146615 -2.5781551375
H 5.3959515557 2.1741609537 -0.9798190712
H -0.3529026118 2.0507078084 0.2497900024
H -2.1854302822 -2.4543553377 0.3750308180
C -2.0958619396 -2.1082876481 -1.7374961435
C -2.0888241735 3.3132432598 0.0700213545
H -2.2130477493 0.8774672313 1.4136586748
H -3.0585760424 3.3511035055 -0.4391617863
H -1.5014211150 4.1710120582 -0.2749013976
H -2.2695889893 3.4462021518 1.1437717382
H -1.0824266004 -2.5093952578 -1.8384398243
H -2.2239890824 -1.2996496155 -2.4662929151
H -2.8005037934 -2.9116991391 -1.9845207413
H -1.1872777500 1.8727688688 -1.2875431039
39

C -3.7955739533 -0.4424084307 0.3316440032
C -3.0348681046 -0.5074311469 1.6620205891
C -1.8191542884 1.7413520436 1.4574524293
C -1.7121396071 0.2104236994 1.3383080005
C -2.7078961645 -0.6467062136 -0.7452331698
N -1.4248779462 -0.2179600029 -0.0691769451
H -2.8218883888 -1.5487280612 1.9286053453
H -3.5769665855 -0.0447070027 2.4934385717
H -4.5809030930 -1.2003251632 0.2491258054
H -4.2740118387 0.5364758691 0.2066314934
S -0.1531368299 -1.3368764756 -0.2542338274
O -0.3040359584 -1.8952397092 -1.6043232617
O -0.0614940833 -2.2382903200 0.9075309197
C 1.3143053275 -0.3063592948 -0.2367634864
C 2.1750571913 -0.3530066263 0.8573087762
C 1.6060197042 0.4923981787 -1.3463641521
C 3.3322980947 0.4283211786 0.8458964349
H 1.9435752120 -1.0013232419 1.6955385559
C 2.7631143895 1.2633466937 -1.3410702188
H 0.9367573529 0.5001455218 -2.2003501538
C 3.6435433985 1.2464030956 -0.2459257420
H 4.0058869797 0.3945199687 1.6985497354
H 2.9929824953 1.8863225991 -2.2023269806
C 4.9028485794 2.0795847708 -0.2642939043
H 4.6857523172 3.1250554756 -0.5129376638
H 5.4103177065 2.0611505800 0.7048376737
H 5.6100141649 1.7100930363 -1.0177490944

H -2.6335381593 2.1027309009 0.8163988038
H -2.6399996641 -1.7105158308 -0.9910733425
C -2.9389857493 0.1331062952 -2.0362408194
C -0.5348015956 2.5031823467 1.1264563595
H -0.9049955133 -0.1402829230 1.9879662973
H -0.6780721006 3.5810295625 1.2641286444
H -0.2303406185 2.3305903234 0.0903027250
H 0.2940189305 2.1912668137 1.7731365302
H -2.9604145686 1.2116053906 -1.8423888439
H -3.8966754022 -0.1554891561 -2.4869658296
H -2.1452495043 -0.0778274579 -2.7583835634
H -2.1210022392 1.9626856359 2.4909151039

13$_{piperidine}$

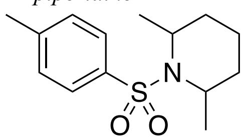

Name	E(B3LYP)	H(B3LYP)	E(RO-B2PLYP-D3)	H(RO-B2PLYP-D3)
Tosyl_2_methyl_6_methyl_piperidine_0001	-1149.471436	-1149.117408	-1149.031422	-1148.677394
Tosyl_2_methyl_6_methyl_piperidine_0000	-1149.471033	-1149.116995	-1149.03085	-1148.676812
Tosyl_2_methyl_6_methyl_piperidine_0006	-1149.467507	-1149.113371	-1149.029688	-1148.675552
Tosyl_2_methyl_6_methyl_piperidine_0012	-1149.468512	-1149.114549	-1149.028453	-1148.67449
Tosyl_2_methyl_6_methyl_piperidine_0002	-1149.467623	-1149.113688	-1149.028038	-1148.674103
Tosyl_2_methyl_6_methyl_piperidine_0024	-1149.464946	-1149.11091	-1149.028015	-1148.673979
Tosyl_2_methyl_6_methyl_piperidine_0043	-1149.46615	-1149.111871	-1149.027542	-1148.673263
Tosyl_2_methyl_6_methyl_piperidine_0013	-1149.465984	-1149.112034	-1149.025844	-1148.671894
Tosyl_2_methyl_6_methyl_piperidine_0018	-1149.463795	-1149.109678	-1149.023835	-1148.669718

39

C 2.4203356563 2.1135209114 0.7220299040
C 3.6226098655 1.8663945115 -0.1906721170
C 2.5104825108 -0.2860513608 -0.9684965146
C 3.1895917544 1.0253797872 -1.3934758373
C 1.6883491487 0.8258950944 1.1498867637
N 1.3421488562 0.0539123163 -0.0866888960
H 4.4172424827 1.3528719034 0.3664129631
H 4.0456485525 2.8218793969 -0.5242954686
H 2.7291248925 2.6432708182 1.6318376931
H 1.6962880542 2.7586997930 0.2049872210
S 0.2355401056 -1.2378254702 0.0707708921
O 0.3278999593 -1.8705925579 1.3968789852
O 0.3934281122 -2.0648411544 -1.1337355417
C -1.3701686062 -0.4383323050 -0.0417993550
C -1.6399946213 0.4573152646 -1.0795569985
C -2.3683118213 -0.8137591243 0.8548364651
C -2.9194793201 0.9900296658 -1.2011444430
H -0.8523666930 0.7439495477 -1.7683043925
C -3.6471656327 -0.2728963546 0.7156968458
H -2.1385053753 -1.5114506965 1.6530675609
C -3.9441941707 0.6324882678 -0.3096847730
H -3.1290606175 1.6958631230 -2.0015349680
H -4.4255195777 -0.5599930835 1.4183686523
C -5.3343107175 1.2002639247 -0.4706593996
H -5.9367263878 1.0477740595 0.4300898697
H -5.3062200236 2.2749811863 -0.6828646556
H -5.8614416241 0.7203671831 -1.3055739945
H 2.0785883040 -0.7474809534 -1.8572929539
H 2.4828113427 1.6028617750 -2.0037014516
H 2.3626489794 0.2201061035 1.7728922607
C 0.4771017872 1.2116357572 2.0067478272
C 3.4894285414 -1.2983960586 -0.3485764676
H 4.0464203366 0.7829321853 -2.0341244402
H 4.2997114634 -1.5049721226 -1.0577153750
H 2.9828630717 -2.2435483659 -0.1355125187
H 3.9449744038 -0.9388107083 0.5792578477
H 0.8317505461 1.8343426973 2.8356793813
H -0.0243878622 0.3436429724 2.4361333812
H -0.2475331581 1.7960844771 1.4309165682
39

C 2.8766919676 1.7158534226 -0.1824810153
C 4.0458965123 0.7715749338 0.1358130463
C 2.3474903976 -1.0448331325 0.7658946767
C 3.6300912006 -0.6989635204 -0.0180168770
C 1.6295776954 1.3497820442 0.6416690637
N 1.2444976988 -0.0845036520 0.4412480553
H 4.3949788859 0.9576258148 1.1615933512
H 4.8974684524 0.9875237867 -0.5208082761
H 3.1580434083 2.7537959600 0.0396529703
H 2.6224392258 1.6667667916 -1.2474199760
S 0.3090255941 -0.4971625044 -0.9179697435
O 0.4881433033 -1.9405870789 -1.1293964215
O 0.5348025738 0.4289840400 -2.0405513848
C -1.3785959008 -0.2401511435 -0.3536084504
C -2.2689184276 0.4352740804 -1.1858369522
C -1.8123957353 -0.8049483772 0.8484006736
C -3.6041582492 0.5577719222 -0.7988658023
H -1.9143671547 0.8595037923 -2.1189409319
C -3.1462554940 -0.6715080020 1.2202252914
H -1.1107952749 -1.3325844384 1.4860119992
C -4.0633623001 0.0127090552 0.4057652044
H -4.2993918891 1.0855729562 -1.4470420461
H -3.4845354331 -1.1071450333 2.1575164743
C -5.5028607677 0.1698138847 0.8345065010
H -5.6037735765 0.9470663110 1.6034485885
H -6.1426785153 0.4543784723 -0.0064545039
H -5.8965629994 -0.7591152814 1.2618400408
H 1.9965363557 -2.0219857859 0.4339273429
H 3.4481002778 -0.9159072776 -1.0784735929
H 1.9067236292 1.3872706846 1.7029014755
C 0.4810781742 2.3459964812 0.4649388386
C 2.5524052992 -1.1258078012 2.2852810574
H 4.4418273227 -1.3635652059 0.3054012403
H 1.6043629063 -1.3421112157 2.7882624929
H 2.9655227618 -0.2094214901 2.7185869147
H 3.2527157129 -1.9378016958 2.5115351679
H 0.8260444660 3.3307288082 0.7993465574
H -0.3857845747 2.0676918325 1.0713863341
H 0.1699831158 2.4350840583 -0.5782988435
39

C 3.6276221059 -0.3383937759 0.3267533805
C 3.7361224900 1.1294748387 0.7655103957
C 1.2814274750 1.4524463586 0.0717561789
C 2.3608195055 1.6954753480 1.1496396192
C 2.5895790336 -0.4887776611 -0.7979138443
N 1.2438224485 0.0186564676 -0.3699836578
H 4.1744824648 1.7161033051 -0.0548292435
H 4.4240836876 1.2269776520 1.6140533855
H 4.6002548288 -0.7006757561 -0.0308759089
H 3.3398687132 -0.9738594433 1.1748619864
S 0.2322572198 -0.9389953048 0.6085080572
O 0.3983662655 -0.6375479442 2.0414516036
O 0.3459636446 -2.3291922965 0.1569934025
C -1.3862820080 -0.3164051831 0.1266777176
C -1.7997471060 -0.4128740928 -1.2054097897
C -2.2452006983 0.1758170674 1.1068746828
C -3.0809824874 0.0034127805 -1.5502780610
H -1.1219215285 -0.8026945927 -1.9575990793

C -3.5285392738 0.5885103309 0.7431578748
H -1.9058205570 0.2328820722 2.1355940966
C -3.9665968859 0.5087332033 -0.5834193232
H -3.4035213566 -0.0655954008 -2.5866128211
H -4.1987798297 0.9772756781 1.5057932985
C -5.3615961040 0.9369344860 -0.9722633703
H -5.8515273750 1.4891916126 -0.1646409706
H -5.3503386078 1.5762892546 -1.8624935136
H -5.9893052330 0.0679183220 -1.2080627700
H 0.3057672507 1.6579291263 0.5228181875
H 2.0237245934 1.2176884803 2.0750359277
H 2.8626144081 0.2214925643 -1.5887460899
C 2.6032496842 -1.8709875637 -1.4557507245
C 1.3982252759 2.3785357676 -1.1471816026
H 2.4314216491 2.7732738993 1.3461513887
H 2.3726767227 2.3153994692 -1.6414272477
H 1.2615194543 3.4179693830 -0.8282597095
H 0.6247152405 2.1385099424 -1.8831978656
H 3.5602648526 -1.9826583553 -1.9793316752
H 1.7975494544 -1.9690294125 -2.1886320167
H 2.5028649635 -2.6817695246 -0.7342719838
39

C 2.7722491926 1.8693979234 -0.3172877326
C 3.7233333352 0.7128013256 -0.6884182598
C 2.4306563355 -0.9823228192 0.7004491733
C 3.8024650873 -0.3048721373 0.4554343331
C 1.6562244562 1.3844483061 0.6374293920
N 1.3026761362 -0.0440364125 0.3917399976
H 4.7200283383 1.0978635566 -0.9321099800
H 3.3528796939 0.2116543658 -1.5908527284
H 3.3239580238 2.6718586583 0.1912852646
H 2.3309113096 2.2994446276 -1.2212893248
S 0.3320708425 -0.4271598089 -0.9443121671
O 0.5490138188 -1.8518933913 -1.2337379999
O 0.4879758364 0.5650060505 -2.0213105773
C -1.3428016990 -0.2525601676 -0.3139313189
C -1.7173729380 -0.8759653520 0.8789413426
C -2.2809212284 0.4302165956 -1.0854732535
C -3.0402769138 -0.7974465230 1.3017086560
H -0.9778681174 -1.4011502860 1.4742205017
C -3.6045388006 0.4976705548 -0.6478029261
H -1.9709048425 0.9071383656 -2.0089204510
C -4.0060414339 -0.1128671006 0.5457492037
H -3.3307614654 -1.2743864278 2.2349535985
H -4.3356695817 1.0356078592 -1.2462018044
C -5.4426336745 -0.0554523584 1.0078761595
H -6.0137434542 0.6931311175 0.4503374847
H -5.9407843103 -1.0235758769 0.8677501416
H -5.5110037251 0.1901010350 2.0738064248
H 2.3320692363 -1.8170275966 0.0053479944
H 4.5407708110 -1.0857370399 0.2380253556
H 2.0730653141 1.3514961843 1.6518335414
C 0.4618582260 2.3369744146 0.6924846088
C 2.2933939502 -1.5190029259 2.1274310866
H 4.1453680581 0.1955260832 1.3726761870
H 3.1361226254 -2.1795117225 2.3652585129
H 1.3683110125 -2.0927837374 2.2438875034
H 2.2885099489 -0.7051010932 2.8627030457
H -0.2885789552 1.9975567082 1.4127947588
H -0.0122726489 2.4528841387 -0.2852141607
H 0.8183221606 3.3239996357 1.0088336326
39

C 1.5069491170 1.9650370403 0.8511218330
C 2.2246571347 2.3160431849 -0.4589070133
C 2.2909169022 -0.1865298113 -1.0234268487
C 1.9735476946 1.2388770929 -1.5225937975
C 1.8896602153 0.5543714666 1.3379210663
N 1.6126791621 -0.4625010972 0.2782267542
H 3.3023432927 2.4118362569 -0.2658265473
H 1.8908818055 3.2931324394 -0.8291302756
H 1.7665175524 2.6918410632 1.6317874751
H 0.4182186220 2.0160674882 0.7152279822
S 0.2731601777 -1.4834404781 0.2569429331
O 0.1132685961 -2.0522560748 1.5965681806
O 0.4585816065 -2.3530927507 -0.9106326858
C -1.2170566215 -0.5179447291 -0.0691355803
C -1.5585681106 -0.2024985812 -1.3874296250
C -2.0341411718 -0.1151050384 0.9876273751
C -2.7055584521 0.5470373313 -1.6366973101
H -0.9492586602 -0.5692957568 -2.2066899697
C -3.1801392055 0.6339530005 0.7208331129
H -1.7904466069 -0.4152640277 2.0008494040
C -3.5295621905 0.9859851809 -0.5893414334
H -2.9741655674 0.7834281447 -2.6636387858
H -3.8201821151 0.9384790845 1.5454355176
C -4.7563731738 1.8207329997 -0.8696242081
H -4.4969985640 2.8836057059 -0.9647475037
H -5.2399105545 1.5209574205 -1.8054708759
H -5.4922752909 1.7352121115 -0.0638507457
H 1.8809194675 -0.9050558504 -1.7328740563
H 0.9176875089 1.2754143302 -1.8204946566
H 2.9831635385 0.5287942016 1.4373410369
C 1.3333465691 0.2557299104 2.7313431709
C 3.7951143134 -0.4836053989 -0.9201285687
H 2.5640441438 1.4427816349 -2.4250395498
H 3.9503286562 -1.5037213689 -0.5570368432
H 4.3270605973 0.2027415382 -0.2534055699
H 4.2528346543 -0.3968594059 -1.9123352396
H 0.2463991927 0.3572041947 2.7791400039
H 1.7661370678 0.9882379066 3.4223473224
H 1.5956539164 -0.7477213132 3.0679269749
39

C 2.9631959119 1.5937799735 1.2036498504
C 3.1414035776 2.1916195789 -0.1955630881
C 1.4959912715 0.4589910254 -1.0617683819
C 1.8910572457 1.9458211351 -1.0454106487
C 2.5357532037 0.1109761429 1.1948132942
N 1.2916851217 0.0269284336 0.3597954541
H 4.0196158305 1.7440598806 -0.6794317126
H 3.3454224009 3.2669551429 -0.1228518901
H 3.8979617871 1.6760308009 1.7723013546
H 2.1989834536 2.1587075507 1.7549007039
S 0.1749215095 -1.2482530785 0.5207033439
O -0.0183876820 -1.5229798340 1.9472932039
O 0.4786684282 -2.3679216903 -0.3876002020
C -1.3271926673 -0.4632603654 -0.0875727428
C -2.0024094795 -1.0256975880 -1.1686265337
C -1.8531247310 0.6402773888 0.5906193915
C -3.2119754133 -0.4646186004 -1.5824951401
H -1.5803460244 -1.8888715010 -1.6722264451
C -3.0577679371 1.1883547994 0.1631131050
H -1.3205832994 1.0616067715 1.4368793496
C -3.7579119211 0.6449267764 -0.9270620819
H -3.7389551052 -0.8996303100 -2.4281058609
H -3.4668405889 2.0502821107 0.6851352803
C -5.0776578495 1.2364795445 -1.3617549155
H -5.8771510911 0.9765553487 -0.6558514346
H -5.0309029481 2.3305236908 -1.4060176496
H -5.3765591277 0.8693138182 -2.3482897730
H 0.5154930755 0.3885438980 -1.5419922529
H 1.0510076015 2.5212379775 -0.6361170748
H 3.3248781682 -0.4780882564 0.7042807695
C 2.4197314795 -0.3885656819 2.6381611314
C 2.4690110891 -0.4160618204 -1.8724207345
H 2.0469188011 2.2854117653 -2.0770251684
H 2.4721905587 -0.0766966077 -2.9150598652
H 2.1549234159 -1.4615756550 -1.8491108212
H 3.4987776439 -0.3566525473 -1.5074701860
H 2.2463666403 -1.4620874960 2.7055008422
H 1.6125416756 0.1158621504 3.1755653376
H 3.3677018559 -0.1594644172 3.1392627368
39

C 3.4334082401 0.5209054763 1.1240763159
C 3.6191279257 0.8839600628 -0.3694213727
C 1.6852134194 -0.6235585471 -1.0943731161
C 3.1878493131 -0.2782703264 -1.2755871269

C 2.3318950967 -0.5430142876 1.2967001296
N 1.2278996348 -0.3434268774 0.3073345249
H 4.6619573300 1.1538482045 -0.5710728765
H 3.0104383076 1.7607616043 -0.6124108604
H 4.3646286270 0.1195896723 1.5462780863
H 3.1806930360 1.4150931438 1.7060647885
S 0.1811629898 0.9860817299 0.4501578843
O 0.5064323159 2.0483448564 -0.5179357618
O 0.0844121006 1.3205405541 1.8752954806
C -1.3792823914 0.2717183957 -0.0864328606
C -2.0667109738 0.8571149603 -1.1469324091
C -1.9232488384 -0.8146843365 0.6063282108
C -3.3072629967 0.3383153304 -1.5244175905
H -1.6310877414 1.7051946123 -1.6638580231
C -3.1594279369 -1.3180257705 0.2178697886
H -1.3800463317 -1.2591879500 1.4337981260
C -3.8720444692 -0.7509778914 -0.8528142050
H -3.8434167428 0.7920777106 -2.3542040701
H -3.5828513458 -2.1646787705 0.7533566287
C -5.2169687704 -1.3072808382 -1.2556431520
H -5.9522791996 -1.1874458459 -0.4501809511
H -5.6098597772 -0.8030404756 -2.1435559426
H -5.1549746710 -2.3797156394 -1.4765258159
H 1.1142431082 0.0387675569 -1.7496814490
H 3.3639254723 -0.0326863903 -2.3294415703
H 2.7477811352 -1.5023384846 0.9649154017
C 1.8943929548 -0.7623842522 2.7453523288
C 1.3690189665 -2.0738369431 -1.4688988158
H 3.8001498693 -1.1660627373 -1.0623023958
H 1.7023486228 -2.2817873425 -2.4929023614
H 0.2941468804 -2.2683552975 -1.4064593773
H 1.8829362790 -2.7765785736 -0.8021333350
H 1.0987248999 -1.5131620351 2.7996976589
H 1.5382542140 0.1532887573 3.2157932807
H 2.7541119623 -1.1400441007 3.3120562188
39

C 2.2312102606 2.1704661043 -0.1876197967
C 3.4288821691 1.8518952008 0.7360318313
C 2.0475657966 -0.2594637940 1.2562789822
C 3.4467158907 0.3722010294 1.1836673099
C 1.7677972668 0.9619182467 -1.0173590319
N 1.4189529680 -0.1495551598 -0.0888678209
H 3.3887993286 2.5140614470 1.6084059768
H 4.3723701452 2.0797870574 0.2275432615
H 1.3815604749 2.5212677401 0.4087730284
H 2.4847632257 2.9862922260 -0.8745023561
S 0.2621062230 -1.2577957003 -0.5583209601
O 0.4110840557 -2.4240886919 0.3194337155
O 0.3366461538 -1.3756241448 -2.0193947205
C -1.3577362519 -0.5462302796 -0.2117217586
C -1.9498419433 0.3167485907 -1.1374795169
C -2.0238888578 -0.8811898062 0.9688369504
C -3.1968460872 0.8715683915 -0.8552182585
H -1.4564692753 0.5195193167 -2.0819234638
C -3.2728901171 -0.3223743312 1.2327636313
H -1.5776386051 -1.5953510704 1.6526184882
C -3.8744092318 0.5701715200 0.3344218424
H -3.6587220608 1.5381806560 -1.5796650605
H -3.7948667233 -0.5939078093 2.1472851934
C -5.2138998621 1.1953843958 0.6434292772
H -5.8324220783 0.5343560270 1.2593277813
H -5.0916083663 2.1359320087 1.1971285597
H -5.7693254627 1.4267751743 -0.2712928391
H 2.1674091247 -1.3281961785 1.4473782983
H 4.0491004878 -0.2221389940 0.4884156089
H 0.8457851416 1.2533271243 -1.5329013401
C 2.7895298516 0.5516307677 -2.0907773133
C 1.1971708879 0.3286483054 2.3947178088
H 3.9224065057 0.2755564254 2.1666078130
H 1.0412323768 1.4064080717 2.2862790169
H 1.6979814928 0.1533593651 3.3546703526
H 0.2153512796 -0.1491860252 2.4409261594
H 2.4197035745 -0.3066026317 -2.6541238674
H 3.7582897825 0.2896625281 -1.6541103904
H 2.9472243427 1.3853272995 -2.7866588440
39

C 1.3901409620 2.1039774281 0.5101968337
C 1.3823376301 2.0344216821 -1.0335644245
C 2.2231924769 -0.3660420890 -1.0609719432
C 2.4741877562 1.0837799410 -1.5378301335
C 1.9896300586 0.8204260691 1.1267887465
N 1.6350936462 -0.3767428472 0.3164943225
H 1.5120548283 3.0335778841 -1.4648288357
H 0.4086793998 1.6695885859 -1.3829158350
H 1.9911233123 2.9557489036 0.8555597854
H 0.3735385923 2.2638187450 0.8884994174
S 0.2967859544 -1.3560437853 0.5729397369
O 0.1725372409 -1.5824753694 2.0142773522
O 0.4507871375 -2.4752347817 -0.3635696692
C -1.1943140962 -0.4769546280 0.0631253249
C -1.9067581046 0.2851669941 0.9918040986
C -1.6305953287 -0.5663788806 -1.2612423040
C -3.0446287409 0.9770879821 0.5817553692
H -1.5853656690 0.3129832073 2.0275474537
C -2.7701901116 0.1333388978 -1.6554406900
H -1.0978166306 -1.1986890800 -1.9633049126
C -3.4932776785 0.9168374847 -0.7454377487
H -3.5997377919 1.5666294176 1.3076571300
H -3.1093535096 0.0605635522 -2.6860951850
C -4.7463728695 1.6434843601 -1.1725847662
H -5.6355295036 1.0174174113 -1.0201359977
H -4.8938134688 2.5622924303 -0.5954711399
H -4.7140346468 1.9091939902 -2.2342180265
H 1.4824538272 -0.8129611403 -1.7282061109
H 2.5160979617 1.0817010431 -2.6334120227
H 3.0788444535 0.8742788279 1.0021008444
H 1.7498558091 0.7195824270 2.6330413366
C 3.4856175612 -1.2304639365 -1.1060263832
H 3.4595961326 1.4293675133 -1.1959520986
H 3.9290440713 -1.2078124918 -2.1093593910
H 3.2430562532 -2.2654891823 -0.8525723545
H 4.2370431410 -0.8642870868 -0.3960773373
H 0.6896923433 0.7336331047 2.8934501486
H 2.2270048231 1.5857007915 3.1069474683
H 2.1835815231 -0.1931281407 3.0464968562
39

C 2.8874448585 1.6692924365 -1.1153536362
C 2.7372775202 2.3349825313 0.2637770695
C 1.8450604270 0.2181668073 1.3595711657
C 2.9534216071 1.3024420135 1.3707675798
C 2.3900185374 0.1969697288 -1.1360022200
N 1.3772272310 0.0213508193 -0.0515337015
H 1.7342950182 2.7674363734 0.3631567925
H 3.4473377034 3.1640546752 0.3644726960
H 2.3453615879 2.2535830500 -1.8679622896
H 3.9421024950 1.6767180014 -1.4168969669
S 0.2686233933 -1.2435168961 -0.2983319565
O 0.3863075107 -2.2384343443 0.7784695524
O 0.3966775152 -1.6611511678 -1.7014544186
C -1.3431403074 -0.4627792962 -0.1269547239
C -2.3182811877 -1.0968547342 0.6400564303
C -1.6375515027 0.7071087715 -0.8317331661
C -3.5979844212 -0.5434621609 0.7085963610
H -2.0704153542 -2.0044583732 1.1800215353
C -2.9172245532 1.2462455952 -0.7508873630
H -0.8702456500 1.1945302295 -1.4242184914
C -3.9189084330 0.6301921969 0.0172746407
H -4.3580949464 -1.0345347824 1.3111402034
H -3.1452224394 2.1615767219 -1.2922278399
C -5.3084744455 1.2185172334 0.0787176472
H -5.8370832584 1.0827366892 -0.8736291758
H -5.9099143236 0.7455576004 0.8609334467
H -5.2785644104 2.2957828136 0.2790213967
H 2.2574077582 -0.7253656934 1.7379398110
H 3.9417707650 0.8434284422 1.2508742828
H 1.8482648722 0.0289902570 -2.0663892268
C 3.5268815923 -0.8390378252 -1.0948937762

C 0.6910151097 0.6355734031 2.2801353561
H 2.9543502487 1.7793446825 2.3573499409
H 0.2418852957 1.5791171746 1.9512658368
H 1.0792020997 0.7720321915 3.2957938890
H -0.0925744402 -0.1223725247 2.3292334851
H 4.2003340846 -0.6784186319 -1.9452396137
H 3.1223361475 -1.8522170877 -1.1748889548
H 4.1264944367 -0.7757663992 -0.1811620806

14imine

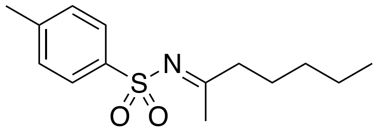

Name	E(B3LYP)	H(B3LYP)	E(RO-B2PLYP-D3)	H(RO-B2PLYP-D3)
Tosyl_imine_2_heptane_0063	-1149.470259	-1149.118714	-1149.020741	-1148.669196
Tosyl_imine_2_heptane_0021	-1149.468909	-1149.11731	-1149.020294	-1148.668695
Tosyl_imine_2_heptane_0035	-1149.470527	-1149.118605	-1149.020459	-1148.668537
Tosyl_imine_2_heptane_0016	-1149.468842	-1149.116967	-1149.020222	-1148.668347
Tosyl_imine_2_heptane_0014	-1149.468834	-1149.116954	-1149.020204	-1148.668324
Tosyl_imine_2_heptane_0000	-1149.469059	-1149.116934	-1149.020414	-1148.668289
Tosyl_imine_2_heptane_0065	-1149.469771	-1149.118041	-1149.019935	-1148.668205
Tosyl_imine_2_heptane_0023	-1149.469267	-1149.117321	-1149.019932	-1148.667986
Tosyl_imine_2_heptane_0050	-1149.4692	-1149.117217	-1149.01966	-1148.667677

39

C -3.4370736975 -0.3419123594 -0.6949222120
C -3.0596368605 1.0950200054 -1.0700704931
C -2.7861172275 2.0071949292 0.1336721853
C -2.4346731552 3.4441129128 -0.2721304603
C -2.3238343691 -1.2431910669 -0.2012509251
N -1.1193869430 -0.7947677330 -0.2660974668
H -3.8835140204 1.5183146825 -1.6607245216
H -2.1796244377 1.0747312344 -1.7233433294
H -3.6724785396 2.0225535142 0.7865026826
C -2.1729234970 4.3613581143 0.9267335263
H -1.3398517585 3.9885979360 1.5351940079
H -3.0539747296 4.4255695932 1.5773589418
H -4.2296114557 -0.3480035227 0.0691989189
H -3.8803527579 -0.8595932058 -1.5606578189
S 0.1765313258 -1.7945843972 0.2512298188
O 0.3652123957 -2.8768441346 -0.7278938139
O 0.0241968602 -2.1068870823 1.6814471153
C 1.5352941478 -0.6436267594 0.0775993751
C 1.9425549266 0.1063313090 1.1802070815
C 2.1813906161 -0.5254771813 -1.1534282300
C 3.0061591096 0.9957445066 1.0376803560
H 1.4378454532 -0.0157958986 2.1326174890
C 3.2417965177 0.3688347211 -1.2770355627
H 1.8611813282 -1.1323816595 -1.9936785662
C 3.6709832396 1.1412757536 -0.1876252316
H 3.3279081845 1.5835760317 1.8939259680
H 3.7483521761 0.4662093543 -2.2342643624
C 4.8435167687 2.0831896722 -0.3248325908
H 4.7853415729 2.9041165844 0.3970673745
H 5.7918915391 1.5587629733 -0.1476270225
H 4.8951516068 2.5155360020 -1.3296292399
H -1.9219180081 5.3787855393 0.6055161301
H -3.2500610544 3.8609353611 -0.8810326912
H -1.9659365784 1.5883895460 0.7293911413
H -1.5476693945 3.4275313143 -0.9207938743
C -2.7497473888 -2.6015960899 0.3103888742
H -2.1502080479 -3.3918746762 -0.1510640689
H -3.8085103895 -2.7842001141 0.1095384033
H -2.5757235668 -2.6569101461 1.3906492443
39

C -2.7300121567 -0.4598089847 -0.2452995901
C -3.2368240957 0.7680613092 0.5485504738
C -4.3105838198 1.5466188020 -0.2219981532
C -4.8256746768 2.7742130776 0.5405306466
C -1.7075445394 -1.2605284847 0.5350952708
N -0.4237626084 -1.1666701046 0.4865373963
H -2.3844381747 1.4223573848 0.7598559617
H -3.6453957538 0.4441028083 1.5160346166
H -3.8982038340 1.8667028326 -1.1893656351
C -5.8925727346 3.5568465659 -0.2318852024
H -6.2397462210 4.4267131891 0.3371863025
H -6.7664458607 2.9298480126 -0.4483588877
H -3.5803208055 -1.1172731376 -0.4692379192
H -2.3102556435 -0.1320674093 -1.1996674067
S 0.3134028736 -0.0571401575 -0.5983958260
O 0.0080068326 1.3192710473 -0.1730819249
O 0.0584634850 -0.4757154342 -1.9863549432
C 2.0350092269 -0.3800572289 -0.2320559414
C 2.6709703525 0.3491160956 0.7724937134
C 2.7234840527 -1.3429547296 -0.9697210754
C 4.0136292090 0.0960959269 1.0450415232
H 2.1218218305 1.1064286233 1.3217367650
C 4.0657672140 -1.5821246467 -0.6831854505
H 2.2141950397 -1.8855885079 -1.7589563817
C 4.7310198822 -0.8693774732 0.3245117875
H 4.5143306516 0.6607191916 1.8278509289
H 4.6072748203 -2.3324040295 -1.2543021669
C 6.1949718777 -1.1128222309 0.6046847555
H 6.4444648611 -0.8992883484 1.6491518652
H 6.4772025562 -2.1490879077 0.3916078868
H 6.8274294112 -0.4680583546 -0.0199658338
H -5.5008584685 3.9200472303 -1.1898054526
H -3.9795225123 3.4362172064 0.7705817265
H -5.1554817305 0.8803399177 -0.4528030407
H -5.2354584374 2.4543778358 1.5093901900
C -2.2563410079 -2.2777090684 1.5052490695
H -2.7682308069 -3.0755550765 0.9514083936
H -3.0029779582 -1.8180862580 2.1641810502
H -1.4554056825 -2.7133666150 2.1047658581
39

C -2.8404277890 -0.0855771399 1.6362570858
C -3.8542733081 0.8739856459 0.9714661396
C -3.2043484975 2.0023431061 0.1603653165
C -4.2279768564 2.9728745827 -0.4428301291
C -2.0135532771 -0.8667085465 0.6402653571
N -0.7576610987 -0.5810236889 0.5954983857
H -4.4739354353 1.3062940774 1.7683751522
H -4.5410771801 0.3053123033 0.3293522248
H -2.5099629366 2.5582112939 0.8069609872
C -3.5808159302 4.1028788869 -1.2499291135
H -2.9077411212 4.7015048851 -0.6239354570
H -4.3357936245 4.7783096572 -1.6679946626
H -2.1614243512 0.4771214920 2.2839269516
H -3.3889269984 -0.8049425100 2.2609945812
S 0.2460153172 -1.4066153931 -0.5295678480
O -0.1275060768 -1.0032783110 -1.8954729469
O 0.3217306114 -2.8329537879 -0.1724313182
C 1.8223674262 -0.6552267173 -0.1403774232

C 2.2158493854 0.5007035256 -0.8141776852
C 2.6451334000 -1.2462842627 0.8184290383
C 3.4481104484 1.0748945257 -0.5090397457
H 1.5685995016 0.9333253213 -1.5695848762
C 3.8737362449 -0.6580127397 1.1102894398
H 2.3273527092 -2.1557291517 1.3170812097
C 4.2945626180 0.5075033581 0.4539180772
H 3.7598474718 1.9767407681 -1.0303506582
H 4.5188774620 -1.1136634539 1.8576303689
C 5.6428488334 1.1181674990 0.7541336517
H 5.6458229456 2.1966032978 0.5654767350
H 5.9345503471 0.9534756571 1.7966428775
H 6.4247202605 0.6743950047 0.1235507200
H -2.9899625764 3.7053417897 -2.0840349418
H -4.8405828705 3.4019785638 0.3630907971
H -2.5892261527 1.5768351399 -0.6444279610
H -4.9217814807 2.4141075844 -1.0871404218
C -2.7291083967 -1.8954379773 -0.2070100217
H -2.6266481251 -1.6467005582 -1.2685609602
H -3.7892892377 -1.9577259805 0.0483372028
H -2.2640100747 -2.8766144599 -0.0667845980
39

C -2.8174284515 -0.4757827708 -0.0569000109
C -3.3229410483 0.7834389059 -0.8037046848
C -4.4639119269 1.5061775826 -0.0693321852
C -4.0749152935 2.1088291689 1.2882349801
C -1.7571380064 -1.2113561868 -0.8504146361
N -0.4776014230 -1.0912926089 -0.7613401781
H -3.6711971284 0.4959771950 -1.8048391650
H -2.4788109682 1.4677596066 -0.9401317436
H -4.8282914345 2.3124746571 -0.7218050361
C -5.2068458327 2.9282592377 1.9169763281
H -4.9069419005 3.3443116253 2.8853072621
H -6.1001212572 2.3125641902 2.0816486990
H -2.4282713916 -0.2006780714 0.9252902297
H -3.6623629604 -1.1588964346 0.1026629430
S 0.2036937625 -0.0302488022 0.4057022379
O -0.0678886775 -0.5443599547 1.7579635095
O -0.1324398184 1.3622505005 0.0631265446
C 1.9412471355 -0.2803128575 0.0580715512
C 2.5792790846 0.5389175833 -0.8729461158
C 2.6390205869 -1.2789168025 0.7373826277
C 3.9340732503 0.3413261606 -1.1313814394
H 2.0220116153 1.3215927544 -1.3765317460
C 3.9931481347 -1.4617400582 0.4659262608
H 2.1274171462 -1.8927749764 1.4709926618
C 4.6610641810 -0.6578099892 -0.4688744314
H 4.4365388903 0.9763319429 -1.8570437262
H 4.5419518211 -2.2394449891 0.9915873013
C 6.1368222838 -0.8425964642 -0.7313757494
H 6.7380449211 -0.2357375573 -0.0413291566
H 6.4408028788 -1.8856952633 -0.5954474579
H 6.4043906504 -0.5392154348 -1.7487674586
H -5.4978420281 3.7652733191 1.2700908893
H -3.1857432664 2.7406717596 1.1596139830
H -5.3124385405 0.8178282746 0.0637306461
H -3.7805284946 1.3119411534 1.9835596754
C -2.2555381144 -2.1880081932 -1.8870239125
H -2.7600288561 -3.0264781882 -1.3892151194
H -1.4292110077 -2.5715752990 -2.4876780285
H -2.9975198228 -1.7125113375 -2.5399092637
39

C -3.5110534765 -0.2562898099 0.4723421601
C -3.1578350660 1.2309681588 0.5834648496
C -2.8533113094 1.8961462912 -0.7672781086
C -2.5376893141 3.3980803795 -0.6724388486
C -2.3704179471 -1.2142953386 0.1945799905
N -1.1748027238 -0.7394713185 0.2222530443
H -2.3043416073 1.3393656159 1.2605085571
H -4.0058579320 1.7482789085 1.0531787222
H -2.0104239041 1.3804294663 -1.2436696138
C -1.2330274190 3.7251475205 0.0646633764
H -1.0283532249 4.8018611199 0.0411019804
H -0.3808843450 3.2104809657 -0.3958845743
H -3.9813887219 -0.6098743920 1.4038851698
H -4.2740600856 -0.4177824089 -0.3047118783
S 0.1542560675 -1.7856294169 -0.0670026493
O 0.0799468289 -2.3010186488 -1.4434898468
O 0.3006741021 -2.7140078415 1.0655696482
C 1.4902707995 -0.5978767222 0.0116927125
C 1.8720180238 0.0823507614 -1.1451794031
C 2.1463948831 -0.3836814852 1.2231568202
C 2.9164465455 1.0014814698 -1.0755521915
H 1.3686671104 -0.1216461896 -2.0843246712
C 3.1905200517 0.5380775274 1.2731031153
H 1.8505439435 -0.9434336482 2.1039821111
C 3.5870363850 1.2482865781 0.1315051471
H 3.2227211559 1.5296835895 -1.9752855744
H 3.7103454412 0.7039627600 2.2135453710
C 4.6991376398 2.2676546685 0.2016586083
H 4.3001238016 3.2715640627 0.3989427421
H 5.4067074932 2.0349986677 1.0039374452
H 5.2560777499 2.3192445320 -0.7397267135
H -1.2697205535 3.4211839079 1.1166807197
H -3.3755615692 3.9166360466 -0.1836840584
H -3.7203349648 1.7584776722 -1.4301637480
H -2.4824245177 3.8062221556 -1.6906910113
C -2.7592738788 -2.6501536342 -0.0788448800
H -2.1734170319 -3.3320099086 0.5445986466
H -2.5340796908 -2.8968905218 -1.1224522735
H -3.8244716145 -2.8128591488 0.1041525442
39

C -2.8087344805 -0.6785299302 0.1462774714
C -3.3788098133 0.6949155894 -0.2826737280
C -4.4912101102 1.1671712030 0.6639985059
C -5.1380227637 2.5022173734 0.2586962435
C -1.7549724028 -1.1843125972 -0.8178136845
N -0.4768211957 -1.0452231526 -0.7386591140
H -3.7767533706 0.6306172295 -1.3054981678
H -2.5575653705 1.4174163822 -0.2981004314
H -5.2720875781 0.3944074250 0.7116480336
C -4.1968928582 3.7108033190 0.3283081336
H -3.7760355588 3.8245545965 1.3348232469
H -4.7297364854 4.6365978941 0.0826773533
H -2.3983832709 -0.6060426760 1.1566838811
H -3.6251934667 -1.4120280791 0.1756310388
S 0.2147965608 -0.2408501609 0.6126515910
O -0.0067005049 -1.0367442136 1.8308679036
O -0.1617122221 1.1825460464 0.5805458587
C 1.9467875955 -0.3644957740 0.1798324816
C 2.6862409843 -1.4620866160 0.6200162161
C 2.5391618991 0.6477969140 -0.5749641795
C 4.0359726335 -1.5463490172 0.2849792289
H 2.2099402896 -2.2288779243 1.2216019410
C 3.8900056247 0.5464073630 -0.9003539875
H 1.9501310920 1.5019977996 -0.8913841597
C 4.6582998603 -0.5475964081 -0.4773177593
H 4.6171783634 -2.4003277692 0.6244167788
H 4.3568951773 1.3320476269 -1.4895071450
C 6.1297290318 -0.6315766328 -0.8071184223
H 6.3570881035 -0.1348578573 -1.7560345281
H 6.7351073133 -0.1444094729 -0.0311683039
H 6.4664478078 -1.6710562114 -0.8770576735
H -3.3578354519 3.6187467353 -0.3699113069
H -5.9995868963 2.6825586202 0.9155438799
H -4.0829262764 1.2556409102 1.6809354936
H -5.5464924235 2.4121333226 -0.7584315668
C -2.2622277368 -1.9226163948 -2.0321587652
H -3.0352947618 -1.3367791887 -2.5441178593
H -2.7309900384 -2.8651695480 -1.7204190023
H -1.4462545321 -2.1386297631 -2.7237131711
39

C -2.6646694345 -0.0575892618 -0.0923187502
C -3.0313665682 1.4372539214 -0.2675558805
C -4.0592835690 1.9324857566 0.7626783802
C -5.4576055979 1.3107515864 0.6425840107

C -1.6422251286 -0.5104766646 -1.1143686897
N -0.3623181278 -0.5705719504 -0.9816720743
H -3.4211646791 1.6080413251 -1.2809603613
H -2.1136261841 2.0253793318 -0.1696044769
H -3.6658843538 1.7600959103 1.7745962587
C -6.4584560095 1.9060336225 1.6388914558
H -7.4485810896 1.4481305146 1.5339318391
H -6.5722804985 2.9865438823 1.4879362079
H -2.2917006754 -0.2248397134 0.9214708851
H -3.5620323357 -0.6750010586 -0.2187508913
S 0.3636224179 -0.1439512706 0.5156322902
O -0.0168471365 -1.1220325522 1.5477129465
O 0.1771298920 1.2966882648 0.7575984825
C 2.0832261898 -0.4176474734 0.1026658974
C 2.8365798302 0.6299454573 -0.4267539615
C 2.6524472253 -1.6697883417 0.3338157642
C 4.1764479830 0.4092710555 -0.7383442833
H 2.3788677882 1.6011944352 -0.5814346330
C 3.9938392481 -1.8718066231 0.0161574970
H 2.0530181237 -2.4653001721 0.7634409026
C 4.7754908979 -0.8399820935 -0.5226766627
H 4.7683692460 1.2219765405 -1.1524204722
H 4.4426569484 -2.8462591080 0.1931287925
C 6.2371979829 -1.0596022557 -0.8326771727
H 6.5730222625 -0.4207707656 -1.6560458183
H 6.8635155874 -0.8248707573 0.0382638293
H 6.4372932730 -2.1009412915 -1.1054816411
H -6.1270030903 1.7504127336 2.6727729146
H -5.4025723045 0.2247792596 0.7977476959
H -4.1485034375 3.0226440861 0.6547054158
H -5.8306656270 1.4554018287 -0.3820021618
C -2.1832959266 -0.9232770490 -2.4613113676
H -1.3701661931 -1.1169968712 -3.1627384910
H -2.8431846431 -0.1464539997 -2.8662189382
H -2.7902664917 -1.8315039083 -2.3516990489
39

C -3.0531053339 -0.0913730791 -1.4849150866
C -4.1250481461 0.6728978540 -0.6700517998
C -3.5745969943 1.6495151598 0.3824549201
C -2.7188171845 2.7913394018 -0.1834698111
C -2.1501006443 -0.9440527509 -0.6235144165
N -0.9283999125 -0.5470839494 -0.5162646876
H -4.7898200386 -0.0488239063 -0.1773283665
H -4.7508323077 1.2217502132 -1.3865167187
H -4.4292629209 2.0785641454 0.9245103927
C -2.3054019136 3.8048993524 0.8888924760
H -3.1821799172 4.2680442123 1.3584298557
H -1.6914464391 4.6073349677 0.4643205995
H -3.5641595521 -0.7480831055 -2.2030675706
H -2.4328169873 0.6069161932 -2.0523386443
S 0.1563528357 -1.4408574722 0.4736287311
O 0.3135146336 -2.8048819262 -0.0575855804
O -0.2085747426 -1.2321144457 1.8844590786
C 1.6712500000 -0.5451204574 0.1514463819
C 2.5221655851 -0.9813184695 -0.8638273213
C 1.9903840857 0.5661222004 0.9318178919
C 3.7029548088 -0.2816402992 -1.1036553059
H 2.2630182317 -1.8590195998 -1.4462062549
C 3.1751042892 1.2533425028 0.6772278330
H 1.3238440096 0.8772941855 1.7291348147
C 4.0481771569 0.8425705637 -0.3403200960
H 4.3697242392 -0.6165039489 -1.8946054213
H 3.4286241802 2.1210518633 1.2814419085
C 5.3461915259 1.5741999709 -0.5867757312
H 5.2707589048 2.6301927744 -0.3076964228
H 6.1619039067 1.1379366735 0.0049818396
H 5.6436035895 1.5199200985 -1.6391692928
H -1.7214124453 3.3229580180 1.6824673026
H -3.2793153792 3.3060753826 -0.9778320718
H -2.9877901750 1.0983708080 1.1305252695
H -1.8178635169 2.3786532468 -0.6539925845
C -2.7563608055 -2.1664286625 0.0288348996
H -3.8279935297 -2.2356176593 -0.1710229089
H -2.5900927247 -2.1450712754 1.1106092218
H -2.2599558195 -3.0663255346 -0.3496541436
39

C -3.0811847254 -0.0479821015 1.4237369942
C -4.1365188852 0.6933259221 0.5670965006
C -3.5650236169 1.6395258020 -0.5018436147
C -2.7188300649 2.7963279644 0.0478474572
C -2.1622707374 -0.9248953594 0.6045100510
N -0.9383256087 -0.5315870078 0.5101498723
H -4.7754326524 1.2624982299 1.2555722491
H -4.7923964369 -0.0417600015 0.0822763194
H -2.9647288266 1.0671360915 -1.2227691039
C -2.2842390461 3.7791753172 -1.0445472145
H -1.6860389598 3.2748290467 -1.8132222321
H -1.6774373817 4.5928336945 -0.6311866874
H -2.4714501566 0.6658493412 1.9832082928
H -3.6065832329 -0.6840603594 2.1500733404
S 0.1647705462 -1.4532896286 -0.4326496873
O -0.1706698026 -1.2814957529 -1.8557207968
O 0.3080002568 -2.8029763449 0.1376905431
C 1.6741121474 -0.5510224959 -0.1023153378
C 2.0108279562 0.5399374236 -0.9041289308
C 2.5078538781 -0.9675775741 0.9348238149
C 3.1940801316 1.2281940485 -0.6470917105
H 1.3624257194 0.8296432145 -1.7241714743
C 3.6880066423 -0.2669373120 1.1768131827
H 2.2401838803 -1.8356058778 1.5277386153
C 4.0465105753 0.8417221790 0.3975874507
H 3.4648040985 2.0753120315 -1.2727245495
H 4.3451744464 -0.5912096100 1.9800319016
C 5.3124357570 1.6132492821 0.6857110411
H 5.7447704801 2.0309968742 -0.2295113001
H 5.1147234540 2.4533401306 1.3648007206
H 6.0684288330 0.9806137679 1.1618498752
H -3.1515392824 4.2295206925 -1.5432318754
H -1.8273327758 2.3965040284 0.5466479627
H -4.4087340771 2.0536344024 -1.0719567357
H -3.2936215342 3.3334567969 0.8167679128
C -2.7567847320 -2.1649660052 -0.0248430153
H -3.8325174029 -2.2270333714 0.1542804317
H -2.2701597841 -3.0540808012 0.3900809422
H -2.5677803607 -2.1753172394 -1.1030276188
39

C -2.8968910037 -0.5944172322 0.1495725815
C -3.4824790757 0.4177053809 -0.8662136680
C -4.6893423596 1.1960329385 -0.3133954218
C -4.4095464228 2.1048126110 0.8963468487
C -1.8096952320 -1.4479829601 -0.4701841789
N -0.5359377364 -1.2570629513 -0.4374228751
H -3.8013315024 -0.1186590264 -1.7699633579
H -2.6875594782 1.1053782579 -1.1704652345
H -5.0948376487 1.8119511455 -1.1287828136
C -3.4097444857 3.2344672677 0.6210191821
H -2.4096758779 2.8511908207 0.3938221074
H -3.3197332520 3.8930911055 1.4924609478
H -2.5122261783 -0.0705290748 1.0269204965
H -3.7003815434 -1.2607751541 0.4909712779
S 0.1088698484 0.0736103137 0.4351782195
O -0.1286435048 -0.1156800369 1.8752399080
O -0.2903382878 1.3321909309 -0.2198893265
C 1.8526809190 -0.1787028841 0.1235016354
C 2.6027038744 -0.9455938947 1.0148164802
C 2.4426374508 0.4097989091 -0.9950666676
C 3.9615465313 -1.1313290649 0.7692632140
H 2.1272806221 -1.3790467917 1.8881999987
C 3.8025491378 0.2127732991 -1.2242832087
H 1.8448883251 1.0178221737 -1.6656869948
C 4.5820318120 -0.5574979346 -0.3494062964
H 4.5511946719 -1.7290299095 1.4601460963
H 4.2677481558 0.6681515856 -2.0952094639
C 6.0622787811 -0.7372446029 -0.5890057000
H 6.3012361573 -0.7153886142 -1.6573299132
H 6.6384331994 0.0655061442 -0.1099196236
H 6.4228251669 -1.6859118570 -0.1782047837

H -3.7366303228 3.8501457599 -0.2271213722
H -4.0560541450 1.5025693137 1.7443252631
H -5.4822030470 0.4810929048 -0.0487340418
H -5.3659121446 2.5383007602 1.2192259235

C -2.2713838702 -2.6624586742 -1.2377068072
H -2.7256667905 -3.3829624368 -0.5449688218
H -1.4343558843 -3.1377643635 -1.7516707801
H -3.0450704719 -2.3890391966 -1.9651937828

EPR calculations of PBN adducts

All calculations were performed on B3LYP/6-31G(d) optimized geometries with properties calculated with mixed basis set: EPR-III for C, H, O atoms, def2-QZVP for S-atom, and 6-31G(d) for N-atom (see above). Additional options: ultrafine grid, tight SCF convergence, PCM solvation using toluene, and empirical dispersion D3-BJ were used in these calculations. Only the lowest 10 structures are shown here. For more details, please consult experimental repository for this article on Zenodo.[32]

After the first decomposition, we have had an extensive discussion on whether proposed structures for **10-PBN** and **14-PBN** are correct, due to extremely high α_H of 7.3 and 6.4, respectively. We have examined the NIEHS database,[33] and indeed we couldn't match any previously published radical with detected high hyperfine values. Therefore, due to PBN adduct very flexible structure, we have done MD and conformer generation, and for each unique conformer, we have done calculation of EPR parameters. There were 1300 calculations for **10-PBN** and 500 calculations for **14-PBN** performed just to be certain that complete potential energy surface has been sampled (deposited in Zenodo repository). For **10-PBN**, energy difference between lowest and highest conformer amounted to ~ 55 kJ/mol, and α_H values spanned from 1.12 G to 11.99 G. When hyperfine coupling values were Boltzmann averaged with calculated enthalpies at B3LYP/6-31G(d) level of theory, we arrived at the value of 5.46 G, with lowest lying conformer having α_H value of 5.48 G. The first α_H value larger then experimentally observed 7.5 G corresponded to conformer +7.8 kJ/mol higher then global minima (11.1 G), while first α_H value lower then 3.0 G (arbitrary limit to typical C-centered radicals trapped with PBN) corresponded to conformer +11.9 kJ/mol (2.40 G). Structures with α_H value larger then 3.0 G have a well define structural motif with weak interaction between Tos-N(alkyl)-**H** and radical center **O**-N-PBN and distance 235 pm. Structures where no such interaction occurs have much smaller α_H value, which is comparable to the typical *C*-centered radicals trapped with PBN (see Figure S33).

The same pattern can be observed with calculations on 14-PBN; lowest α_H value being 1.36 G, largest 6.52 G, while Boltzmann averaged 4.32 G. Energy gap between conformers is ~ 45 kJ/mol. Lowest lying conformer has α_H 4.16 G, while first α_H value below 2.5 G is +11.3 kJ/mol less stable than global minima (1.39 G). Conformer with larger then experimental 6.4 G was found to be +21.9 kJ/mol (6.41 G). Again, a Tos-N(alkyl)-H---O-N-PBN motif was present in the most stable conformers (up to 10 kJ/mol), as seen in Figure S34.

Observed interaction in global minimum of **10-PBN** and **14-PBN** was analyzed with Mayer's bond order analysis using MultiWFN program,[34] for which it gave 0.0325 bond order, respectively. Negative bond order was observed for conformers of **10-PBN** with α_H of 2.40 G, and **14-PBN** with α_H of 1.39 G, due to large distance between H-atom of sulfonamide moiety and O-atom of the PBN moiety.

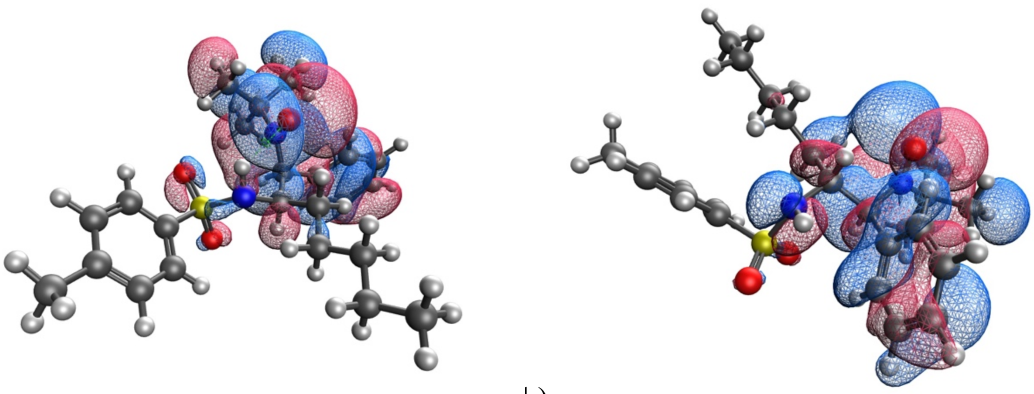

a) b)

Figure S33. SOMO orbital in system **10-PBN**. a) global minima with α_H 5.48 G; b) local minima with α_H 2.40 G. Visualized with IQmol program package,[35] with isosurface equal to 0.0200 Å^{-3}. Interaction is denoted with a green line

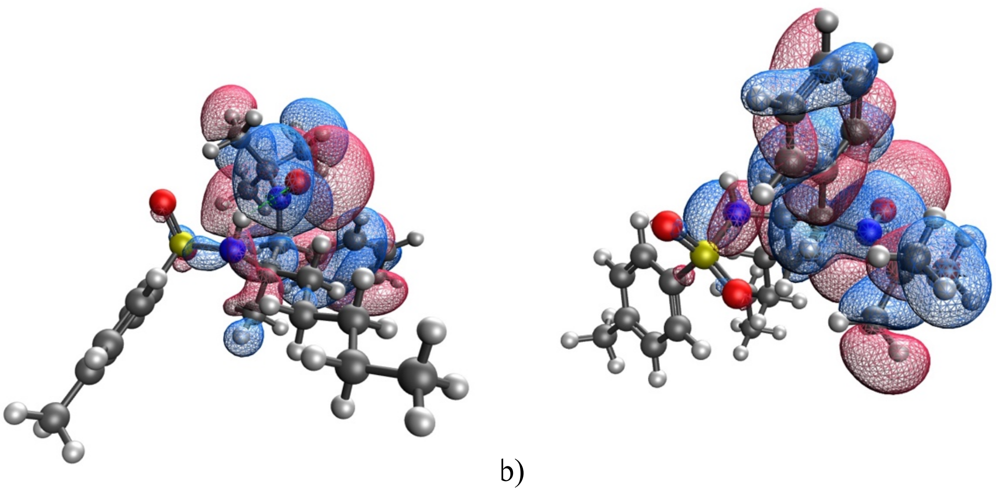

a) b)

Figure S34. SOMO orbital in system **14-PBN**. a) global minima with α_H 4.16 G; b) local minima with α_H 1.39 G. Visualized with IQmol program package,[35] with isosurface equal to 0.0200 Å^{-3}. Interaction is denoted with a green line

7'-PBN

A model for 7-PBN, with full alkyl chain substituted for ethyl.

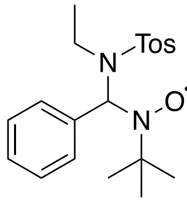

Name	E(B3LYP)	H(B3LYP)	g-factor	α_N	$\alpha_{N'}$	α_H
tos_Net_Nrad_PBN.conf.022.log	-1511.62414567	-1511.152883	2.00611	13.85046	1.50899	2.99032
tos_Net_Nrad_PBN.conf.000.log	-1511.62416598	-1511.151964	2.00611	13.84190	1.50789	3.00497
tos_Net_Nrad_PBN.conf.002.log	-1511.62416599	-1511.151964	2.00611	13.84144	1.50802	3.00528
tos_Net_Nrad_PBN.conf.039.log	-1511.62416587	-1511.151964	2.00611	13.84115	1.50779	3.00583
tos_Net_Nrad_PBN.conf.138.log	-1511.62415126	-1511.151955	2.00611	13.84739	1.50464	3.00496
tos_Net_Nrad_PBN.conf.007.log	-1511.62415195	-1511.151953	2.00611	13.84385	1.50706	3.00483
tos_Net_Nrad_PBN.conf.026.log	-1511.62415096	-1511.151953	2.00611	13.84759	1.50369	3.01246
tos_Net_Nrad_PBN.conf.015.log	-1511.62415205	-1511.151952	2.00611	13.84318	1.50817	3.00338
tos_Net_Nrad_PBN.conf.042.log	-1511.62415189	-1511.151950	2.00611	13.84392	1.50901	2.99888
tos_Net_Nrad_PBN.conf.031.log	-1511.62248338	-1511.151237	2.00613	13.63661	1.70213	3.07110
tos_Net_Nrad_PBN.conf.023.log	-1511.62273186	-1511.150632	2.00621	14.54630	1.34408	2.20326
tos_Net_Nrad_PBN.conf.032.log	-1511.62268833	-1511.150600	2.00621	14.55021	1.34430	2.20444
Boltzman averaged for 298.15 K			2.00612	13.8150	1.5244	2.8859

55
-1511.152883
S 0.990563 -1.155135 1.146005
N -0.242820 -0.909372 0.030766
C 2.503548 -0.621768 0.328533
C 2.517373 0.451993 -0.561734
H 1.594547 0.938606 -0.860930
C 3.736475 0.865947 -1.100893
H 3.748237 1.695382 -1.803639
C 4.938586 0.233895 -0.761146
C 6.254263 0.688412 -1.346936
C 4.892262 -0.846860 0.133017
H 5.812626 -1.359249 0.403584
C 3.686931 -1.281916 0.675033
H 3.653668 -2.128706 1.351909
O 0.705478 -0.254504 2.274742
O 1.146222 -2.593562 1.392482
C -0.203751 -1.672457 -1.236562
C -0.819861 -3.070649 -1.162058
H 6.721023 -0.107234 -1.940798
H 6.966834 0.960605 -0.558857
H 6.124407 1.558615 -1.997270
H -0.306891 -3.678741 -0.412908
H -0.723323 -3.562766 -2.137692
H -1.882105 -3.024379 -0.907107
H -0.700077 -1.052451 -1.984474
C -1.274912 0.076617 0.339715
C -2.668113 -0.378878 -0.090882
C -3.398924 -1.191790 0.786933
H -2.959141 -1.485138 1.737431
C -4.676437 -1.634401 0.446686
H -5.231462 -2.262927 1.137841
C -5.241027 -1.266215 -0.776909
H -6.237484 -1.608378 -1.042992
C -4.521239 -0.452650 -1.652602
H -4.955205 -0.159086 -2.604717
C -3.241690 -0.007549 -1.312718
H -2.686693 0.630505 -1.992524
N -0.933356 1.416468 -0.206958
C -1.196236 2.723899 0.491994
C -2.276745 3.476473 -0.307075
C -1.657821 2.499321 1.938293
C 0.126472 3.511560 0.479028
O -0.525530 1.472507 -1.424768
H -1.275245 0.183517 1.419727
H -3.228732 2.935494 -0.286470
H -1.963791 3.591980 -1.348071
H -2.436247 4.470608 0.124479
H -0.903428 1.970702 2.529389
H -2.604414 1.950055 1.987715
H -1.823340 3.476555 2.403879
H 0.894779 2.985989 1.055850
H -0.024292 4.501243 0.923528
H 0.482124 3.638391 -0.546533
H 0.843557 -1.740137 -1.551019
55
-1511.151964
S 0.991282 -1.148462 1.150944
N -0.242191 -0.908193 0.034452
C 2.504258 -0.621811 0.329185
C 3.684163 -1.290420 0.666097
H 3.649087 -2.141540 1.337394
C 4.890140 -0.861157 0.117567
H 5.808048 -1.382959 0.377434
C 4.938686 0.223023 -0.770127
C 6.254113 0.698058 -1.339593
C 3.737070 0.862136 -1.104298
H 3.749339 1.690358 -1.808718
C 2.519736 0.454795 -0.559546
H 1.597455 0.944399 -0.855530
O 0.707722 -0.239634 2.273523
O 1.145201 -2.585360 1.407328
C -0.201120 -1.674403 -1.230798
C -0.814001 -3.073833 -1.152852
H 6.728795 1.430478 -0.673094
H 6.118911 1.182628 -2.311941
H 6.959423 -0.129985 -1.466005
H -0.715646 -3.568477 -2.127022

H -1.876517 -3.029357 -0.898706
H -0.300160 -3.678662 -0.401636
H -0.698631 -1.057520 -1.980516
C -1.276176 0.076705 0.340541
C -2.667958 -0.380860 -0.092399
C -3.239622 -0.010742 -1.315501
H -2.683972 0.627514 -1.994587
C -4.518083 -0.457298 -1.657550
H -4.950569 -0.164655 -2.610620
C -5.238691 -1.271127 -0.782777
H -6.234313 -1.614412 -1.050534
C -4.676017 -1.638125 0.442055
H -5.231708 -2.266844 1.132497
C -3.399582 -1.194056 0.784465
H -2.961306 -1.486447 1.735952
N -0.935357 1.416407 -0.206892
C -1.199559 2.724198 0.490788
C 0.124338 3.509940 0.484216
C -2.275219 3.478117 -0.313518
C -1.668538 2.500457 1.934841
O -0.523985 1.471595 -1.423530
H -1.278884 0.184896 1.420420
H 0.484870 3.636656 -0.539674
H 0.889088 2.982884 1.064394
H -0.026941 4.499702 0.928383
H -3.228061 2.938483 -0.297102
H -1.957314 3.592817 -1.353085
H -2.435271 4.472596 0.117033
H -1.836661 3.477971 2.398909
H -0.917036 1.972497 2.530170
H -2.615254 1.951001 1.979673
H 0.846426 -1.740515 -1.544864
55
-1511.151964
S 0.991263 -1.148428 1.150951
N -0.242258 -0.908231 0.034512
C 2.504215 -0.621831 0.329097
C 2.519699 0.454606 -0.559818
H 1.597420 0.944119 -0.855968
C 3.737059 0.861902 -1.104579
H 3.749307 1.689998 -1.809150
C 4.938678 0.222919 -0.770219
C 6.254120 0.697912 -1.339688
C 4.890127 -0.861104 0.117686
H 5.808056 -1.382793 0.377709
C 3.684147 -1.290323 0.666219
H 3.649056 -2.141337 1.337647
O 0.707756 -0.239535 2.273494
O 1.145202 -2.585308 1.407424
C -0.201259 -1.674574 -1.230646
C -0.814372 -3.073900 -1.152615
H 6.728305 1.431153 -0.673738
H 6.119064 1.181509 -2.312544
H 6.959772 -0.129990 -1.465085
H -0.300693 -3.678713 -0.401275
H -0.715976 -3.568665 -2.126720
H -1.876910 -3.029247 -0.898597
H -0.698595 -1.057680 -1.980475
C -1.276208 0.076710 0.340563
C -2.668001 -0.380793 -0.092429
C -3.239607 -0.010657 -1.315550
H -2.683891 0.627547 -1.994634
C -4.518086 -0.457142 -1.657626
H -4.950531 -0.164480 -2.610710
C -5.238766 -1.270922 -0.782865
H -6.234403 -1.614141 -1.050649
C -4.676143 -1.637948 0.441983
H -5.231882 -2.266638 1.132415
C -3.399694 -1.193941 0.784425
H -2.961460 -1.486357 1.735924
N -0.935313 1.416381 -0.206876
C -1.199391 2.724184 0.490815
C -1.668353 2.500471 1.934888
C 0.124594 3.509793 0.484250
C -2.274995 3.478225 -0.313439
O -0.523870 1.471523 -1.423490
H -1.278960 0.184913 1.420439
H -2.615082 1.951042 1.979764
H -1.836436 3.477996 2.398943
H -0.916855 1.972502 2.530212
H 0.485104 3.636565 -0.539643
H 0.889304 2.982577 1.064336
H -0.026542 4.499538 0.928508
H -2.434966 4.472693 0.117170
H -3.227885 2.938672 -0.297037
H -1.957110 3.592957 -1.353006
H 0.846298 -1.740916 -1.544638
55
-1511.151964
S 0.991375 -1.147625 1.151556
N -0.242074 -0.908012 0.034866
C 2.504344 -0.621556 0.329428
C 3.684011 -1.290650 0.665837
H 3.648815 -2.141870 1.336996
C 4.890057 -0.861728 0.116953
H 5.807846 -1.383903 0.376430
C 4.938754 0.222524 -0.770482
C 6.253923 0.697659 -1.340458
C 3.737201 0.862219 -1.104198
H 3.749552 1.690547 -1.808514
C 2.519972 0.455283 -0.559159
H 1.597764 0.945235 -0.854785
O 0.707800 -0.238008 2.273499
O 1.145239 -2.584352 1.408952
C -0.200686 -1.674561 -1.230160
C -0.813117 -3.074168 -1.151871
H 6.967026 -0.126516 -1.446105
H 6.717680 1.447280 -0.685483
H 6.121109 1.162133 -2.322963
H -0.714573 -3.569040 -2.125906
H -1.875657 -3.029960 -0.897773
H -0.299121 -3.678642 -0.400475
H -0.698377 -1.058053 -1.980068
C -1.276333 0.076685 0.340604
C -2.667911 -0.381209 -0.092650
C -3.239374 -0.011265 -1.315895
H -2.683686 0.627046 -1.994899
C -4.517678 -0.458079 -1.658197
H -4.950011 -0.165565 -2.611376
C -5.238323 -1.271999 -0.783543
H -6.233822 -1.615482 -1.051500
C -4.675844 -1.638837 0.441428
H -5.231566 -2.267630 1.131778
C -3.399569 -1.194510 0.784092
H -2.961447 -1.486765 1.735692
N -0.935664 1.416408 -0.206912
C -1.200105 2.724223 0.490607
C -1.669922 2.500558 1.934405
C 0.123894 3.509807 0.484762
C -2.275244 3.478229 -0.314297
O -0.523893 1.471507 -1.423417
H -1.279363 0.185035 1.420466
H -0.918743 1.972693 2.530221
H -2.616632 1.951046 1.978706
H -1.838381 3.478095 2.398300
H 0.484982 3.636504 -0.538938
H 0.888254 2.982622 1.065336
H -0.027483 4.499584 0.928864
H -3.228146 2.938683 -0.298396
H -1.956775 3.592896 -1.353694
H -2.435441 4.472725 0.116160
H 0.846894 -1.740401 -1.544171
55
-1511.151955
S -0.992324 1.145150 1.153935
N 0.240097 0.907137 0.035675
C -2.505346 0.617362 0.333027
C -3.686503 1.280126 0.676503
H -3.653089 2.126156 1.354301
C -4.893310 0.845756 0.133505

H -5.813330 1.358116 0.404414
C -4.940569 -0.234331 -0.758953
C -6.248605 -0.676047 -1.370888
C -3.738562 -0.872658 -1.093788
H -3.751755 -1.706528 -1.791574
C -2.520613 -0.460303 -0.554664
H -1.598430 -0.951232 -0.848671
O -0.705843 0.236373 2.275793
O -1.148191 2.581686 1.411164
C 0.196064 1.673294 -1.229508
C 0.805782 3.074141 -1.152044
H -7.091173 -0.481910 -0.699156
H -6.239879 -1.745121 -1.607019
H -6.447392 -0.137251 -2.306932
H 1.868580 3.032008 -0.898654
H 0.291181 3.677848 -0.400448
H 0.705643 3.568548 -2.126155
H 0.694455 1.057657 -1.979658
C 1.276254 -0.075888 0.340351
C 2.666624 0.384368 -0.094253
C 3.397873 1.198627 0.781936
H 2.960303 1.489860 1.734105
C 4.673037 1.645201 0.438029
H 5.228436 2.274712 1.127986
C 5.234809 1.279685 -0.787653
H 6.229445 1.624902 -1.056585
C 4.514574 0.464825 -1.661785
H 4.946369 0.173330 -2.615522
C 3.237398 0.015770 -1.318237
H 2.682048 -0.623284 -1.996823
N 0.937155 -1.416191 -0.206850
C 1.206192 -2.723590 0.489752
C -0.115121 -3.513672 0.483540
C 2.283738 -3.473566 -0.315738
C 1.675335 -2.499188 1.933644
O 0.524312 -1.471894 -1.422969
H 1.280505 -0.184308 1.420203
H -0.475991 -3.640785 -0.540179
H -0.881192 -2.989644 1.064720
H 0.039780 -4.503266 0.926823
H 1.965476 -3.588658 -1.355162
H 2.447226 -4.467803 0.114085
H 3.234885 -2.930952 -0.299648
H 1.846204 -3.476434 2.397271
H 0.922789 -1.973336 2.529521
H 2.620669 -1.947334 1.978214
H -0.851881 1.736901 -1.542743
55
-1511.151953
S 0.991883 -1.146533 1.152855
N -0.240435 -0.907300 0.034830
C 2.504956 -0.617926 0.332589
C 3.686646 -1.279386 0.677647
H 3.653455 -2.124726 1.356325
C 4.893245 -0.844717 0.135037
H 5.813525 -1.356043 0.407152
C 4.940166 0.234546 -0.758838
C 6.248958 0.675853 -1.369444
C 3.738045 0.871318 -1.095135
H 3.750985 1.704305 -1.793927
C 2.519868 0.458529 -0.556217
H 1.597519 0.948484 -0.851372
O 0.705459 -0.238970 2.275723
O 1.147693 -2.583364 1.408512
C -0.197282 -1.673444 -1.230380
C -0.808586 -3.073608 -1.153102
H 6.221942 1.732190 -1.655530
H 6.475891 0.097773 -2.275141
H 7.083656 0.531644 -0.675410
H -0.708804 -3.568032 -2.127242
H -1.871379 -3.030409 -0.899895
H -0.294700 -3.677912 -0.401497
H -0.694835 -1.057119 -1.980528
C -1.276116 0.076082 0.340126
C -2.666900 -0.383489 -0.093896
C -3.398211 -1.197366 0.782598
H -2.960408 -1.488799 1.734598
C -4.673726 -1.643307 0.439197
H -5.229183 -2.272529 1.129370
C -5.235793 -1.277534 -0.786280
H -6.230703 -1.622277 -1.054806
C -4.515508 -0.463034 -1.660695
H -4.947531 -0.171323 -2.614262
C -3.237973 -0.014600 -1.317650
H -2.682591 0.624191 -1.996455
N -0.936719 1.416274 -0.207002
C -1.204139 2.723674 0.490134
C -2.281335 3.474897 -0.314617
C -1.672867 2.499312 1.934175
C 0.117948 3.512500 0.483600
O -0.524465 1.472025 -1.423325
H -1.279812 0.184225 1.420006
H -3.232948 2.933095 -0.298324
H -1.963435 3.590096 -1.354136
H -2.443794 4.469111 0.115649
H -0.920460 1.972814 2.529661
H -2.618583 1.948141 1.979006
H -1.842799 3.476592 2.398076
H 0.883781 2.987474 1.064191
H -0.035767 4.502074 0.927340
H 0.478437 3.639652 -0.540248
H 0.850631 -1.738300 -1.543462
55
-1511.151953
S -0.992330 -1.145348 -1.153818
N 0.239734 -0.906651 -0.035405
C -2.505451 -0.617834 -0.333060
C -3.686440 -1.280861 -0.676261
H -3.652871 -2.127189 -1.353686
C -4.893343 -0.846387 -0.133381
H -5.813301 -1.358986 -0.404010
C -4.940720 0.234025 0.758545
C -6.248633 0.676211 1.370412
C -3.738723 0.872654 1.093199
H -3.752038 1.706770 1.790700
C -2.520805 0.460240 0.554262
H -1.598592 0.951175 0.848191
O -0.706017 -0.236615 -2.275752
O -1.147693 -2.581970 -1.410864
C 0.196000 -1.672966 1.229644
C 0.807632 -3.073010 1.152480
H -6.245179 1.748456 1.592057
H -6.439861 0.149191 2.314697
H -7.093010 0.468082 0.705249
H 1.870546 -3.029597 0.899836
H 0.294230 -3.677218 0.400457
H 0.707400 -3.567672 2.126446
H 0.692889 -1.056622 1.980222
C 1.276001 0.076101 -0.340378
C 2.666348 -0.384285 0.093968
C 3.397874 -1.197775 -0.782706
H 2.960606 -1.488201 -1.735259
C 4.672910 -1.644662 -0.438765
H 5.228549 -2.273577 -1.129069
C 5.234275 -1.280235 0.787437
H 6.228806 -1.625740 1.056386
C 4.513791 -0.466086 1.662020
H 4.945282 -0.175384 2.616133
C 3.236741 -0.016709 1.318428
H 2.681231 0.621880 1.997329
N 0.937158 1.416453 0.207026
C 1.206431 2.723901 -0.489512
C 1.675249 2.499450 -1.933494
C -0.114712 3.514236 -0.483013
C 2.284283 3.473458 0.315903
O 0.523930 1.472103 1.422985
H 1.279942 0.184577 -1.420224
H 0.922422 1.973868 -2.529260
H 2.620395 1.947295 -1.978268
H 1.846331 3.476669 -2.397100

H -0.475363 3.641384 0.540776
H -0.881004 2.990368 -1.064052
H 0.040273 4.503817 -0.926300
H 2.448018 4.467696 -0.113823
H 3.235259 2.930552 0.299636
H 1.966187 3.588529 1.355380
H -0.852123 -1.738129 1.541976
55
-1511.151952
S 0.991653 -1.147016 1.152460
N -0.240745 -0.907504 0.034694
C 2.504740 -0.618191 0.332331
C 2.519614 0.458016 -0.556642
H 1.597255 0.947765 -0.852095
C 3.737896 0.870926 -1.095465
H 3.750823 1.703717 -1.794468
C 4.939993 0.234508 -0.758826
C 6.249151 0.675798 -1.368670
C 4.893102 -0.844695 0.135277
H 5.813423 -1.355859 0.407613
C 3.686560 -1.279435 0.677716
H 3.653337 -2.124694 1.356492
O 0.705335 -0.239799 2.275646
O 1.147445 -2.583947 1.407651
C -0.197794 -1.673437 -1.230691
C -0.809579 -3.073395 -1.153610
H 6.215076 1.726292 -1.674610
H 6.487694 0.082637 -2.261519
H 7.080160 0.551920 -0.666222
H -0.296015 -3.677874 -0.401925
H -0.709756 -3.567825 -2.127742
H -1.872405 -3.029864 -0.900613
H -0.695083 -1.056792 -1.980749
C -1.276141 0.076192 0.340084
C -2.667084 -0.383096 -0.093726
C -3.398339 -1.196971 0.782808
H -2.960396 -1.488588 1.734685
C -4.673977 -1.642695 0.439578
H -5.229397 -2.271931 1.129768
C -5.236213 -1.276688 -0.785748
H -6.231216 -1.621267 -1.054141
C -4.515982 -0.462170 -1.660193
H -4.948150 -0.170275 -2.613637
C -3.238321 -0.013958 -1.317325
H -2.682978 0.624854 -1.996140
N -0.936507 1.416259 -0.207093
C -1.203195 2.723737 0.490286
C -2.280166 3.475529 -0.314252
C -1.671822 2.499422 1.934361
C 0.119258 3.511904 0.483597
O -0.524569 1.471967 -1.423520
H -1.279648 0.184321 1.419967
H -1.962359 3.590663 -1.353806
H -2.442092 4.469782 0.116118
H -3.232019 2.934156 -0.297862
H -2.617741 1.948600 1.979262
H -1.841356 3.476726 2.398354
H -0.919553 1.972584 2.529715
H 0.479648 3.638969 -0.540298
H 0.884954 2.986486 1.064015
H -0.033900 4.501520 0.927436
H 0.850115 -1.738624 -1.543703
55
-1511.151950
S 0.991399 -1.148776 1.151162
N -0.241055 -0.907771 0.033796
C 2.504492 -0.618815 0.331772
C 3.686712 -1.279292 0.677964
H 3.653624 -2.124499 1.356816
C 4.893109 -0.844022 0.136091
H 5.813601 -1.354651 0.408962
C 4.939789 0.235149 -0.758365
C 6.249645 0.677008 -1.366304
C 3.737605 0.870540 -1.095823
H 3.750350 1.703011 -1.795172
C 2.519140 0.456991 -0.557428
H 1.596658 0.946079 -0.853603
O 0.705154 -0.243191 2.275665
O 1.147244 -2.586050 1.404291
C -0.198882 -1.673043 -1.232032
C -0.811166 -3.072815 -1.155561
H 7.072733 0.592726 -0.648602
H 6.202285 1.715126 -1.710018
H 6.511073 0.056111 -2.233468
H -0.297349 -3.678120 -0.404728
H -0.712266 -3.566492 -2.130169
H -1.873797 -3.028997 -0.901780
H -0.696349 -1.055815 -1.981488
C -1.275874 0.076301 0.339952
C -2.667230 -0.382326 -0.093267
C -3.398230 -1.196287 0.783417
H -2.959806 -1.488382 1.734928
C -4.674212 -1.641484 0.440801
H -5.229408 -2.270793 1.131104
C -5.237070 -1.274859 -0.784060
H -6.232347 -1.619013 -1.051982
C -4.517095 -0.460273 -1.658647
H -4.949732 -0.167906 -2.611734
C -3.239080 -0.012592 -1.316393
H -2.683934 0.626253 -1.995337
N -0.935952 1.416371 -0.207069
C -1.201837 2.723807 0.490703
C 0.120588 3.511990 0.482612
C -2.279676 3.475743 -0.312538
C -1.668868 2.499318 1.935274
O -0.524943 1.472172 -1.423818
H -1.278795 0.184114 1.419865
H 0.479888 3.639046 -0.541664
H 0.886944 2.986653 1.062227
H -0.032159 4.501608 0.926589
H -1.962979 3.591134 -1.352405
H -2.441155 4.469903 0.118224
H -3.231512 2.934375 -0.295276
H -1.837572 3.476561 2.399697
H -0.916091 1.972105 2.529660
H -2.614893 1.948761 1.981144
H 0.848856 -1.738355 -1.545595
55
-1511.151237
S -0.996100 -0.909273 -1.354540
N 0.217413 -0.916743 -0.181380
C -2.505963 -0.535769 -0.457072
C -2.530861 0.465317 0.518923
H -1.616102 0.966373 0.820188
C -3.742316 0.779161 1.128760
H -3.764727 1.551272 1.894331
C -4.930577 0.117694 0.779641
C -6.231294 0.467766 1.462405
C -4.872912 -0.880161 -0.200503
H -5.780657 -1.407978 -0.482091
C -3.669214 -1.213585 -0.821657
H -3.625171 -1.993417 -1.574006
O -0.681289 0.213857 -2.252440
O -1.161407 -2.256424 -1.915930
C 0.246668 -2.054665 0.764788
C -0.133403 -1.707930 2.204078
H -6.215453 0.173038 2.519435
H -7.080534 -0.034958 0.989909
H -6.418293 1.547613 1.433095
H 0.525238 -0.941114 2.616096
H -0.056700 -2.610973 2.822226
H -1.159801 -1.335457 2.266050
H -0.429756 -2.813135 0.361970
C 1.340151 0.007637 -0.381343
C 2.672997 -0.575274 0.080291
C 3.364825 -1.422834 -0.796533
H 2.934977 -1.655503 -1.768297
C 4.592126 -1.973919 -0.430066
H 5.117716 -2.627707 -1.120784
C 5.145885 -1.678642 0.817740

H 6.104343 -2.103466 1.103531
C 4.464435 -0.832100 1.693099
H 4.890425 -0.595937 2.664571
C 3.233583 -0.281869 1.329171
H 2.706645 0.378472 2.009496
N 1.067338 1.333576 0.231479
C 1.409543 2.657796 -0.399155
C 2.000366 2.480227 -1.804721
C 0.103134 3.469011 -0.478007
C 2.426258 3.360244 0.520051
O 0.593376 1.360491 1.424175
H 1.414474 0.191628 -1.448831
H 1.284850 2.019423 -2.492170
H 2.923568 1.890412 -1.791834
H 2.251491 3.470403 -2.199598
H -0.618388 2.969798 -1.132580
H 0.306679 4.467678 -0.879811
H -0.336218 3.575660 0.517282
H 3.368738 2.803222 0.559762
H 2.026230 3.441385 1.533753
H 2.637321 4.366666 0.142636
H 1.247422 -2.494887 0.731829
55
-1511.150632
S -0.887886 -1.440506 -1.099579
N 0.352814 -1.046711 -0.030774
C -2.423381 -0.780682 -0.438539
C -2.857639 0.486665 -0.831143
H -2.282013 1.053436 -1.554118
C -4.050300 0.988879 -0.316668
H -4.394517 1.970954 -0.631922
C -4.820194 0.246681 0.590654
C -6.093240 0.815369 1.169976
C -4.372994 -1.031259 0.951943
H -4.968581 -1.634109 1.633050
C -3.185645 -1.552757 0.441184
H -2.868456 -2.558874 0.692677
O -0.598811 -0.718940 -2.347218
O -1.024332 -2.899189 -1.073537
C 0.390532 -1.742927 1.277462
C 1.145397 -3.072269 1.253703
H -5.883850 1.405951 2.071765
H -6.793735 0.023610 1.454257
H -6.597318 1.477478 0.458218
H 1.133323 -3.516716 2.256744
H 2.188521 -2.925159 0.958819
H 0.680232 -3.771690 0.555437
H 0.832836 -1.042775 1.985031
C 1.193594 0.110807 -0.317572
C 2.659940 -0.173117 0.010772
C 3.254389 0.190693 1.224568
H 2.666837 0.710723 1.973861
C 4.596672 -0.110111 1.468046
H 5.046550 0.176566 2.414896
C 5.357482 -0.772238 0.504065
H 6.401983 -1.002779 0.695736
C 4.770503 -1.134393 -0.710830
H 5.355802 -1.647109 -1.469390
C 3.430891 -0.835799 -0.954812
H 2.972605 -1.127298 -1.897055
N 0.685224 1.351760 0.331572
C 0.839906 2.732118 -0.256809
C -0.465281 3.489278 0.043879
C 2.021754 3.434817 0.439051
C 1.075806 2.663085 -1.772003
O 0.395771 1.286361 1.581826
H 1.115699 0.270701 -1.388849
H -0.377723 4.528176 -0.291597
H -0.670068 3.478087 1.116464
H -1.313668 3.028835 -0.472235
H 2.100862 4.469236 0.086592
H 2.967591 2.926701 0.227504
H 1.866142 3.446608 1.521467
H 2.038442 2.203261 -2.020027
H 1.091102 3.683096 -2.170109
H 0.281043 2.114070 -2.288079
H -0.639905 -1.891117 1.618409
55
-1511.150600
S 0.894023 -1.427906 1.103289
N -0.347321 -1.041333 0.032603
C 2.429157 -0.772104 0.437581
C 2.855230 0.504389 0.809569
H 2.271901 1.082297 1.517359
C 4.044603 1.005659 0.287335
H 4.378638 1.998044 0.580677
C 4.822193 0.251425 -0.603667
C 6.123430 0.795595 -1.142968
C 4.379092 -1.031600 -0.950677
H 4.973840 -1.637917 -1.629444
C 3.194625 -1.552254 -0.431843
H 2.878575 -2.560783 -0.674978
O 0.605262 -0.698614 2.346522
O 1.031740 -2.886647 1.086965
C -0.381389 -1.741247 -1.273654
C -1.130591 -3.073778 -1.247265
H 6.067639 1.876855 -1.307529
H 6.394423 0.320327 -2.091001
H 6.946966 0.616038 -0.439151
H -1.115313 -3.520804 -2.249115
H -2.174712 -2.930481 -0.954084
H -0.663315 -3.769353 -0.546559
H -0.825869 -1.044745 -1.983482
C -1.195512 0.111118 0.317661
C -2.660043 -0.182386 -0.010317
C -3.427669 -0.846298 0.957055
H -2.968297 -1.131924 1.900570
C -4.765371 -1.153606 0.713331
H -5.348076 -1.667185 1.473302
C -5.353751 -0.799042 -0.503110
H -6.396760 -1.036377 -0.694611
C -4.596280 -0.135675 -1.468889
H -5.047294 0.145170 -2.416946
C -3.255954 0.173852 -1.225666
H -2.671053 0.694905 -1.976332
N -0.695094 1.354351 -0.333497
C -0.860208 2.734738 0.252116
C -2.046491 3.427563 -0.446096
C -1.096741 2.666893 1.767259
C 0.439643 3.500846 -0.048999
O -0.404771 1.288596 -1.583525
H -1.118554 0.273306 1.388646
H -1.889912 3.438652 -1.528379
H -2.133556 4.461959 -0.095450
H -2.988753 2.912848 -0.234593
H -2.056285 2.200729 2.015421
H -1.119543 3.687523 2.163430
H -0.298451 2.124499 2.284902
H 0.343359 4.540342 0.282192
H 0.646698 3.486930 -1.121114
H 1.290531 3.049292 0.470875
H 0.650034 -1.885990 -1.613215

8-PBN

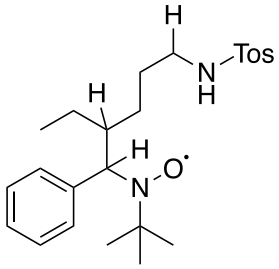

**Name**	**E(B3LYP)**	**H(B3LYP)**	**g-factor**	α_N	α_H
Tosyl_N_radical_hexane_C5_radical_PBN_adduct_ 0131	-1668.884100	-1668.291654	2.00601990	15.296630	2.719150
Tosyl_N_radical_hexane_C5_radical_PBN_adduct_ 0143	-1668.884347	-1668.291550	2.00601093	15.441580	2.353580
Tosyl_N_radical_hexane_C5_radical_PBN_adduct_ 0009	-1668.883995	-1668.291450	2.00587403	14.716370	1.712350
Tosyl_N_radical_hexane_C5_radical_PBN_adduct_ 0028	-1668.884002	-1668.291372	2.00587553	14.770610	1.572880
Tosyl_N_radical_hexane_C5_radical_PBN_adduct_ 0173	-1668.884079	-1668.291337	2.00600763	15.505020	2.478350
Tosyl_N_radical_hexane_C5_radical_PBN_adduct_ 0138	-1668.883513	-1668.291168	2.00604103	15.544240	2.859410
Tosyl_N_radical_hexane_C5_radical_PBN_adduct_ 0050	-1668.883458	-1668.291111	2.00587917	14.808020	2.165860
Tosyl_N_radical_hexane_C5_radical_PBN_adduct_ 0099	-1668.883246	-1668.290936	2.00598773	15.121750	3.574900
Boltzman averaged for 298.15 K			2.005944	15.0887	2.3647

65

C 2.8676213112 0.2345754447 0.4423378919
C 3.6700846801 0.2493090052 -1.9797414549
H 2.6360789363 0.1434107195 -2.2925877959
C 4.6872358884 0.3135002560 -2.9313909573
H 4.4363002467 0.2772828157 -3.9883138410
C 6.0225208030 0.4200245137 -2.5334350559
H 6.8126833325 0.4703561305 -3.2777334901
C 6.3345739729 0.4552610020 -1.1746920931
H 7.3697511640 0.5321989197 -0.8526265496
C 5.3154061464 0.3875707740 -0.2220537311
H 5.5689132367 0.4122570087 0.8355810002
C 3.9729366940 0.2906446916 -0.6099782232
N 2.0381171208 -0.9919224644 0.2578444964
C 2.3865945959 -2.3620309669 0.7795017328
C 3.4611185358 -2.2856790310 1.8717532901
C 2.8889431556 -3.2075526860 -0.4071121081
C 1.0934856141 -2.9629228536 1.3594378656
H 3.6481736811 -3.2986784127 2.2428596368
H 3.1359201532 -1.6804321896 2.7251258712
H 4.4108450380 -1.8933586245 1.4949554882
H 2.1359311995 -3.2245964745 -1.1988174984
H 3.0771569038 -4.2354967990 -0.0784217817
H 3.8184670354 -2.7996356131 -0.8173058709
H 0.2891801195 -2.9545351861 0.6218189806
H 0.7545638347 -2.3968196895 2.2339729179
H 1.2763676635 -3.9966650496 1.6723225344
O 1.1256454082 -0.9514703831 -0.6486453999
C -1.2374381047 1.7506874957 0.0844600335
N -1.5116367357 0.3158189979 -0.0724046819
S -2.6484486905 -0.4494053491 0.8794710424
O -2.3259371359 -1.8797436928 0.7913917184
O -2.7879737526 0.2060523188 2.1896059035
C -4.1807597572 -0.1458946877 -0.0074710020
C -4.3412366493 -0.6554828080 -1.2999972257
C -5.2099795215 0.5552181394 0.6154068632
C -5.5424572205 -0.4476331170 -1.9674742230
H -3.5321935806 -1.2040825693 -1.7713604080
C -6.4104350242 0.7559465113 -0.0701012870
H -5.0665353845 0.9320417686 1.6222314743
C -6.5964693068 0.2607144200 -1.3648377012
H -7.2145627468 1.3050379488 0.4136632395
C -0.4241271895 2.1698856840 1.3255234754
H -0.2038414644 3.2423775058 1.2261995967
H -1.0588727440 2.0568287717 2.2095878069
C 0.8684651080 1.3692599782 1.5590311874
H 0.5904398069 0.3198007160 1.6991795330
H 1.3034462630 1.6851219695 2.5155362032
C 1.9335083832 1.4791083221 0.4423253973
H 1.4222510158 1.4555362460 -0.5254598136
C 2.7141402749 2.8141781990 0.4921367473
H 1.9819602908 3.6269897873 0.3979356515
C 3.5727913536 3.0720102765 1.7381037688
H 4.3724225001 2.3310880021 1.8480416355
H 4.0523122455 4.0548559396 1.6677168754
H 2.9818997131 3.0647076965 2.6604498999
H -5.6692663265 -0.8414668210 -2.9733667921
C -7.8976635661 0.4689549424 -2.1028444346
H -8.5834624355 1.1048464079 -1.5348852524
H -7.7316748632 0.9397833180 -3.0793257209
H -8.4048604706 -0.4860826057 -2.2892466144
H -2.1978593501 2.2781530856 0.0833231202
H 3.3322332483 0.1468226168 1.4264142571
H -0.6926470886 -0.2814417097 -0.2220723754
H -0.7246560593 2.0514243318 -0.8359678784
H 3.3509871984 2.8831148335 -0.3973449253
65

C 2.5476355736 0.2529470660 0.3342799547
C 2.2541436254 0.3653280320 -2.1984983049
H 1.1820341920 0.2825168906 -2.0508032982
C 2.7781004894 0.4606491060 -3.4871537540
H 2.1052453021 0.4714798114 -4.3406591117
C 4.1591019102 0.5393303394 -3.6855016439
H 4.5637081589 0.6142328462 -4.6912096309
C 5.0146960028 0.5159839301 -2.5845718767
H 6.0907717633 0.5715491787 -2.7262334924
C 4.4894150976 0.4176107558 -1.2941414025
H 5.1648753767 0.3974350958 -0.4415589458
C 3.1064038640 0.3474077734 -1.0835297153
N 1.7212513668 -0.9822568914 0.4814227249
C 2.2557959859 -2.3577458451 0.7845876488
C 3.6629280032 -2.2854128308 1.3911575294
C 2.2790011049 -3.1602615216 -0.5310337183
C 1.2852509024 -3.0026774256 1.7913322568
H 3.9839308682 -3.3016117893 1.6421314320
H 3.6804820070 -1.6986501742 2.3162256175

H 4.3969776016 -1.8733647055 0.6917342262
H 1.2816246748 -3.1758996121 -0.9775427713
H 2.5906199938 -4.1915575005 -0.3327626061
H 2.9770614832 -2.7197603409 -1.2500571600
H 1.2938080311 -2.4625072804 2.7446333066
H 1.5882496565 -4.0374363856 1.9831380240
H 0.2626096229 -3.0022229272 1.4084624544
O 0.5190605579 -0.9351264842 0.0225122312
C -1.3311443698 1.6087128968 1.8217922165
N -1.4812768598 0.1504695822 1.9243488937
S -2.9947144449 -0.5386016136 1.7248942623
O -2.7737602054 -1.9833678684 1.8341099923
O -3.9210174351 0.1769107583 2.6075961683
C -3.5081583534 -0.2001171973 0.0321771204
C -2.8709815210 -0.8581690879 -1.0248130036
C -4.5243459203 0.7224608385 -0.2118965268
C -3.2626846633 -0.5812123187 -2.3312248777
H -2.0808074580 -1.5751859714 -0.8252678520
C -4.9067409104 0.9846457148 -1.5282244709
H -5.0113279798 1.2140687834 0.6234805211
C -4.2870113884 0.3392471866 -2.6050052038
H -5.7023178140 1.7009046227 -1.7195505168
C -0.0816364441 2.0799406063 2.5843961920
H 0.0282486656 3.1596891786 2.4140549712
H -0.2662548570 1.9467465329 3.6569464605
C 1.2222672863 1.3397152700 2.2316704178
H 1.0795024904 0.2811356384 2.4732265188
H 2.0120197178 1.6892638102 2.9083804279
C 1.6954603417 1.4826331245 0.7647622005
H 0.8142822981 1.4661348800 0.1154388559
C 2.4083823274 2.8301909965 0.5004679093
H 1.7055006609 3.6303883828 0.7676367401
C 3.7321871555 3.0737035771 1.2382329363
H 3.6195669615 3.0229876297 2.3268027839
H 4.5034765038 2.3519473863 0.9476716152
H 4.1181013724 4.0708850187 0.9987013637
H -2.7687046973 -1.0916208361 -3.1549288951
C -4.7239047279 0.6033540702 -4.0265769691
H -3.8682860994 0.6340411441 -4.7102917447
H -5.3959485272 -0.1871907553 -4.3858627469
H -5.2611219936 1.5532521509 -4.1114999621
H -2.2166267244 2.0638089907 2.2733607691
H 3.3837384574 0.1472764310 1.0281700631
H -0.8014272998 -0.3658371308 1.3575824859
H -1.2935637486 1.9314791471 0.7703996761
H 2.5800543564 2.9326328618 -0.5772316826
65

C 2.7718661696 0.0606371117 0.3185770310
C 3.4356314908 0.4689381874 -2.1131455014
H 2.3832016686 0.5835114382 -2.3513967763
C 4.3988115114 0.5878257012 -3.1151034464
H 4.0891346412 0.8141999769 -4.1321344226
C 5.7530141986 0.4157441927 -2.8190392279
H 6.5009544017 0.5104989921 -3.6017070271
C 6.1377891760 0.1157910742 -1.5122784339
H 7.1876265736 -0.0269871076 -1.2698495791
C 5.1725358834 -0.0080787870 -0.5112132722
H 5.4827917816 -0.2505347325 0.5029395264
C 3.8126887450 0.1717279163 -0.7948963473
N 1.7090000101 -0.9143254316 -0.0532665564
C 1.7388063989 -2.3589699353 0.3738705303
C 1.5301440648 -2.4392240539 1.8997439429
C 3.0886654709 -2.9871374597 -0.0235421674
C 0.5928544000 -3.0894478721 -0.3366659908
H 0.5502528711 -2.0403498020 2.1779891494
H 2.3044041669 -1.8969638702 2.4534134290
H 1.5753133608 -3.4873509828 2.2163215710
H 3.2583344946 -2.9061318711 -1.1015895797
H 3.0841485513 -4.0489300198 0.2466630043
H 3.9334269830 -2.5190168312 0.4905257592
H 0.6180874249 -4.1439467326 -0.0413284210
H 0.6939759835 -3.0270141191 -1.4230997070
H -0.3744595876 -2.6709443582 -0.0509486783
O 0.8030733711 -0.5153613271 -0.8751334125
C -0.9656099030 2.3490907244 0.6777585522
N -1.4866732277 1.0443921341 0.2373420056
S -2.5683488722 0.1998309538 1.1928974970
O -1.9756543282 -1.1083645276 1.5236334349
O -3.0395484812 1.1109785668 2.2445718589
C -3.9567287656 -0.1243388221 0.1010154470
C -3.9596408615 -1.2659098070 -0.7014468992
C -5.0218669289 0.7763987418 0.0660198148
C -5.0371069060 -1.4942219315 -1.5548148043
H -3.1346196643 -1.9682685863 -0.6491787570
C -6.0922333485 0.5308288241 -0.7915951472
H -5.0125926611 1.6475506248 0.7123066015
C -6.1184798861 -0.6039528641 -1.6141980845
H -6.9239842386 1.2308351044 -0.8192000713
C -0.0143720255 2.3318980893 1.8908846617
H 0.4126370781 3.3396951975 1.9927917687
H -0.6132590264 2.1547319320 2.7908014869
C 1.0944330741 1.2673538732 1.8335808678
H 0.6087119687 0.2903967252 1.7622771140
H 1.6223270632 1.2617346782 2.7952701551
C 2.1132601174 1.4268044097 0.6817214072
H 1.5693889358 1.7236831825 -0.2216660964
C 3.1484616653 2.5439346508 0.9544203905
H 2.5926965739 3.4808448816 1.0921737658
C 4.0799878185 2.3480591289 2.1585732287
H 3.5293496823 2.2587974843 3.1012721815
H 4.7076107630 1.4561790981 2.0534480974
H 4.7541475407 3.2064258096 2.2564254541
H -5.0403089039 -2.3824143813 -2.1822436289
C -7.2977243851 -0.8785789090 -2.5170806572
H -6.9962706714 -1.4294073409 -3.4141815710
H -8.0556658887 -1.4842789022 -2.0025934841
H -7.7843105852 0.0494327513 -2.8347661350
H -1.8185785347 2.9968303596 0.8963627022
H 3.2613467004 -0.3481621737 1.2076896677
H -0.7824887061 0.4157041383 -0.1630551978
H -0.4680742033 2.7661902352 -0.2047616003
H 3.7571523156 2.6889202525 0.0547346863
65

C 2.6863919270 0.0117159138 0.3172841720
C 3.3522519009 0.6011931118 -2.0769852357
H 2.3075449107 0.8050974364 -2.2876089070
C 4.3119522496 0.7506103510 -3.0782222538
H 4.0084828500 1.0904111759 -4.0651111438
C 5.6546052845 0.4647645036 -2.8200570012
H 6.3999378200 0.5838459090 -3.6018855482
C 6.0308451511 0.0200952123 -1.5526340977
H 7.0712630826 -0.2118200581 -1.3403703969
C 5.0687328958 -0.1341291936 -0.5527733980
H 5.3717581236 -0.4892457411 0.4299057960
C 3.7209522071 0.1584338427 -0.7978312438
N 1.5588858884 -0.8570162825 -0.1212518712
C 1.5031526804 -2.3341104578 0.1711496039
C 0.2787428407 -2.9148107484 -0.5466531776
C 1.3539245986 -2.5449141369 1.6913830731
C 2.7867660313 -3.0108128627 -0.3480307518
H 0.3403183903 -2.7633561117 -1.6269798472
H -0.6434778541 -2.4554017642 -0.1846129842
H 0.2361311249 -3.9904808718 -0.3440947129
H 2.1931779296 -2.1220768621 2.2541685236
H 1.3269387584 -3.6193197000 1.9053304629
H 0.4237513754 -2.0993022382 2.0556953679
H 2.7192826793 -4.0909632280 -0.1767874207
H 3.6833573586 -2.6495007786 0.1643580963
H 2.9150335595 -2.8386903326 -1.4211365684
O 0.6792110359 -0.3334794299 -0.9007262044
C -0.8832409363 2.5023774769 0.9211271495
N -1.4993367390 1.2907234133 0.3467201425
S -2.5784271526 0.4118432668 1.2795099842
O -1.9536231111 -0.8213137434 1.7969544866
O -3.1985991249 1.3625827958 2.2122105915
C -3.7945507519 -0.1042827367 0.0641756245
C -4.1550261010 -1.4479089694 -0.0073185729
C -4.4233757543 0.8489356635 -0.7420421740

C -5.1486963385 -1.8400932732 -0.9059880165
H -3.6599758704 -2.1698776198 0.6327350169
C -5.4106730961 0.4410951077 -1.6319726004
H -4.1332547380 1.8923151823 -0.6775112324
C -5.7886073027 -0.9084072897 -1.7306409880
H -5.8985738947 1.1806021361 -2.2627496487
C 0.0599234573 2.2983558465 2.1224383533
H 0.5479368074 3.2618297139 2.3270497783
H -0.5516329054 2.0701521877 3.0031420857
C 1.0999393251 1.1771634575 1.9567402609
H 0.5519636269 0.2436795595 1.8059200480
H 1.6322215574 1.0532454922 2.9080459962
C 2.1192170346 1.3775679240 0.8118799137
H 1.5893192175 1.7895106446 -0.0540072290
C 3.2250327384 2.3981852833 1.1710313748
H 2.7303536150 3.3508278331 1.4024842362
C 4.1527849372 2.0347006315 2.3386914237
H 4.8783830290 2.8384599072 2.5070427898
H 3.6052986738 1.8900644665 3.2763330556
H 4.7245943193 1.1208968725 2.1423063299
H -5.4298739975 -2.8887179983 -0.9643091797
C -6.8488381081 -1.3366848451 -2.7171624275
H -7.1751826184 -2.3649436310 -2.5338116638
H -7.7299219676 -0.6868689120 -2.6639085574
H -6.4736794960 -1.2865718216 -3.7476123943
H -1.6913379387 3.1799727983 1.2076888857
H 3.1582679039 -0.5053533345 1.1579407485
H -0.8300784046 0.6659801455 -0.1178260315
H -0.3546547035 2.9690011687 0.0826691716
H 3.8339009633 2.5883115645 0.2798604386
65

C 2.6403493693 0.1846943703 0.2782820390
C 2.4258039076 0.2931366917 -2.2636669027
H 1.3473396227 0.2562528175 -2.1470869348
C 2.9926040908 0.3610982097 -3.5360037757
H 2.3472733591 0.3942814383 -4.4099525194
C 4.3809740070 0.3845926146 -3.6917309210
H 4.8189774710 0.4376176940 -4.6847684987
C 5.2010140217 0.3362183227 -2.5645095430
H 6.2822488142 0.3518696196 -2.6732162845
C 4.6331684868 0.2653171000 -1.2906677412
H 5.2794310426 0.2325558412 -0.4159729666
C 3.2424458059 0.2464247443 -1.1236544275
N 1.7223339856 -0.9820433424 0.3917984303
C 2.1479771665 -2.3984482115 0.6849308562
C 1.1240000314 -2.9828914792 1.6752445232
C 3.5507697610 -2.4381993786 1.3050603175
C 2.1256874683 -3.1859585283 -0.6396580253
H 1.3638381705 -4.0332585553 1.8717092973
H 0.1077994926 -2.9277971210 1.2795100634
H 1.1478524249 -2.4441567578 2.6289286672
H 3.6010716314 -1.8717318467 2.2415057689
H 4.3192050279 -2.0669529529 0.6198989685
H 3.7953107256 -3.4792983448 1.5393760845
H 2.8644058557 -2.7933835438 -1.3456012196
H 1.1357280768 -3.1205291545 -1.0978961356
H 2.3542995426 -4.2401211689 -0.4489561794
O 0.5387510861 -0.8352493741 -0.0947704531
C -1.1865135512 1.5530157857 1.7664448689
N -1.3506855969 0.0989026366 1.9001801565
S -2.8807289934 -0.5712446524 1.7889280552
O -2.6651375819 -2.0202580079 1.8560454498
O -3.7411869009 0.1313078917 2.7454801724
C -3.5069441628 -0.1948711577 0.1424089220
C -2.9143033009 -0.7931603023 -0.9744098629
C -4.5716514117 0.6923661969 -0.0059807922
C -3.3996787356 -0.4921834373 -2.2433318303
H -2.0839284721 -1.4814367277 -0.8508540566
C -5.0484099537 0.9789832701 -1.2860292893
H -5.0205843545 1.1392237590 0.8745845191
C -4.4750318688 0.3932053434 -2.4207891878
H -5.8815924009 1.6680211803 -1.4025037233
C 0.0545217245 2.0399793637 2.5294403523
H 0.1287254608 3.1227627670 2.3760150727
H -0.1278609460 1.8832118966 3.5995878758
C 1.3867314053 1.3452284671 2.1783059458
H 1.2709655682 0.2831935955 2.4222763890
H 2.1583176038 1.7212730297 2.8650904433
C 1.9075418673 1.4897280099 0.7222596254
H 1.0593883747 1.6232045984 0.0413173037
C 2.8623547868 2.6922254771 0.5488418104
H 3.2130341972 2.7188973750 -0.4883320671
C 2.2670030548 4.0629923247 0.8914148359
H 1.3465083883 4.2523714688 0.3258217854
H 2.0336196525 4.1610720550 1.9564177243
H 2.9783455042 4.8577663692 0.6395103263
H -2.9394096396 -0.9551111720 -3.1133122281
C -5.0112093958 0.6837722417 -3.8026509399
H -5.6126068957 1.5981726304 -3.8156818297
H -4.2010086659 0.7992258362 -4.5313229526
H -5.6502911603 -0.1352863254 -4.1580573043
H -2.0705502519 2.0284567261 2.2003809267
H 3.4512246384 0.0091107754 0.9894720533
H -0.7109817343 -0.4250219256 1.2954268873
H -1.1378105525 1.8468925446 0.7076176633
H 3.7572642815 2.5275993091 1.1684440791
65

C 2.9100893883 0.1778776887 0.5083503198
C 3.8006572533 0.2868127324 -1.8805508737
H 2.7751373997 0.2434858106 -2.2336997885
C 4.8546055602 0.3580315769 -2.7909351109
H 4.6422773985 0.3886057376 -3.8564629366
C 6.1771149332 0.3872123876 -2.3408775183
H 6.9960006830 0.4426699603 -3.0530752199
C 6.4394795532 0.3408220512 -0.9717199192
H 7.4641431236 0.3605791843 -0.6098905997
C 5.3837254202 0.2664995991 -0.0605765908
H 5.5961218666 0.2352065725 1.0060736297
C 4.0542458759 0.2423087613 -0.5012960930
N 2.0303028045 -0.9865287439 0.2171219271
C 2.3199916669 -2.4078122599 0.6307192382
C 2.8821937162 -3.1570818878 -0.5933745014
C 0.9843609563 -3.0312220957 1.0717314128
C 3.3218986059 -2.4533849145 1.7920112321
H 3.0188093860 -4.2164044887 -0.3496136855
H 3.8483999022 -2.7457339468 -0.9018515683
H 2.1851836621 -3.0780758824 -1.4315974771
H 0.2378930264 -2.9739733674 0.2781312670
H 0.5790835180 -2.5207514641 1.9517056871
H 1.1405181848 -4.0841586960 1.3299505849
H 4.3040057325 -2.0603414033 1.5114286988
H 3.4595178237 -3.4983814499 2.0883370709
H 2.9550427446 -1.9100346984 2.6698679222
O 1.1554290744 -0.8340865159 -0.7144639866
C -1.1582826962 1.6237630340 0.0914791543
N -1.4703239127 0.1956325964 -0.0347139587
S -2.6401038496 -0.5320626547 0.8990715916
O -2.3654407100 -1.9723239108 0.8036900533
O -2.7721455446 0.1150768042 2.2141865711
C -4.1531509114 -0.1685971423 0.0009511419
C -4.3105249243 -0.6428015683 -1.3053884814
C -5.1730571278 0.5417864191 0.6287398234
C -5.4985645113 -0.3906079044 -1.9811891664
H -3.5086828835 -1.1979842305 -1.7813315214
C -6.3603072422 0.7873324342 -0.0650729003
H -5.0322976218 0.8917052586 1.6455913984
C -6.5427106098 0.3278602889 -1.3734460206
H -7.1568338531 1.3439093337 0.4227075975
C -0.3963203538 2.0688038547 1.3535503964
H -0.2303675911 3.1487199767 1.2580596216
H -1.0529598439 1.9237718636 2.2170459506
C 0.9295687760 1.3371646282 1.6415836666
H 0.6941754808 0.2760783130 1.7783785552
H 1.3034518893 1.6813074970 2.6163474345
C 2.0652522646 1.4869348815 0.5940012596
H 1.6282735648 1.6458760852 -0.3983960487
C 3.0088707580 2.6716709074 0.9028275971
H 3.8017260759 2.6984790123 0.1476085741

C 2.3470008781 4.0537509160 0.9534023263
H 1.7957825480 4.2681828459 0.0296128115
H 1.6496647784 4.1508288626 1.7914573533
H 3.1086254256 4.8331492426 1.0711282153
H -5.6225346727 -0.7568439882 -2.9978176339
C -7.8292527981 0.5859982316 -2.1213281590
H -8.3501009790 -0.3522814164 -2.3500830036
H -8.5120681544 1.2129710842 -1.5400173160
H -7.6408543910 1.0894437946 -3.0773299627
H -2.1006923576 2.1802627403 0.0288869024
H 3.3376160148 0.0008834686 1.4984981122
H -0.6684516511 -0.4197234907 -0.1951754782
H -0.5938395091 1.8756662678 -0.8129910485
H 3.5108483767 2.4829821633 1.8642363240
65

C 2.8755226938 0.0577281072 0.3669359055
C 3.6987377624 0.2798790100 -2.0405341983
H 2.6637059784 0.3584457778 -2.3570781052
C 4.7276958138 0.3346390101 -2.9811941330
H 4.4864539117 0.4721153353 -4.0320987438
C 6.0600243937 0.2125368577 -2.5804614889
H 6.8590843558 0.2562153373 -3.3156809934
C 6.3576736730 0.0302255958 -1.2295751295
H 7.3902593262 -0.0695967600 -0.9053342209
C 5.3274678524 -0.0292853306 -0.2896915755
H 5.5683331388 -0.1721041157 0.7617849233
C 3.9881682500 0.0974730314 -0.6804996403
N 1.8338157530 -0.9286933078 -0.0190490917
C 1.8465677880 -2.3618343184 0.4524078022
C 1.5000067067 -2.4000308150 1.9545097309
C 3.2366247121 -2.9783793902 0.2024349179
C 0.7855957778 -3.1363558644 -0.3395119722
H 2.2066700398 -1.8181491562 2.5565406437
H 1.5417756470 -3.4358434347 2.3096377432
H 0.4878280515 -2.0232677033 2.1291024806
H 4.0240791111 -2.4821427238 0.7773201763
H 3.5057240564 -2.9304580618 -0.8570241793
H 3.2191228187 -4.0306455374 0.5067617913
H -0.2169901382 -2.7500414830 -0.1453446929
H 0.8172548301 -4.1848169391 -0.0240445139
H 0.9789214904 -3.0886618124 -1.4143133655
O 0.9761798813 -0.5572325360 -0.9037558253
C -0.9876883617 2.0248570184 0.3504234699
N -1.4291011140 0.6421546810 0.1361365792
S -2.5498436547 -0.1019973426 1.1085378120
O -2.0893052722 -1.4902920070 1.2818602186
O -2.8392061462 0.7611048888 2.2616657443
C -4.0509461018 -0.1653067664 0.1230351091
C -5.0762767941 0.7446619803 0.3721142176
C -4.1758275410 -1.1241035794 -0.8862178571
C -6.2333088319 0.6978693663 -0.4074702977
H -4.9700711322 1.4670752314 1.1741080657
C -5.3354932698 -1.1563269700 -1.6534714260
H -3.3784374994 -1.8399143021 -1.0571891941
C -6.3821199934 -0.2471611826 -1.4288154983
H -5.4344532618 -1.9024979048 -2.4385936577
C -0.0953747660 2.2780009113 1.5802475034
H 0.2091999233 3.3309520462 1.5435996515
H -0.7149752064 2.1590687591 2.4749494302
C 1.1297613151 1.3547310706 1.7283227324
H 0.7554181106 0.3275957756 1.7756444595
H 1.5845964007 1.5451248494 2.7106677144
C 2.2335605156 1.4569092894 0.6426169270
H 1.7856062097 1.7806034796 -0.3042618281
C 3.3438224279 2.4630176108 1.0217204161
H 4.0994489235 2.4739125581 0.2294468369
C 2.8794303269 3.9044830676 1.2616781608
H 2.3288288330 4.2969669057 0.3979523316
H 2.2336198676 3.9933585166 2.1409610997
H 3.7445597665 4.5573476415 1.4251350743
H -7.0350658444 1.4061286568 -0.2129670568
C -7.6425478545 -0.3088405042 -2.2588031879
H -7.4138488140 -0.3653986529 -3.3294012838
H -8.2377159465 -1.1968968287 -2.0096895227
H -8.2743859160 0.5693262943 -2.0941334766
H -1.8739764079 2.6642378184 0.4153779203
H 3.3047551265 -0.3048676187 1.3075249192
H -0.7114694208 -0.0030349032 -0.2055190657
H -0.4682640135 2.3027490776 -0.5731511153
H 3.8560908367 2.1002235843 1.9261854700
65

C 2.8558291296 0.1711631938 0.5745317395
C 3.8340551052 0.2299001513 -1.7832461055
H 2.8374507284 0.0438085386 -2.1715414616
C 4.9125186136 0.3774218166 -2.6549099093
H 4.7482435174 0.3149305070 -3.7274971530
C 6.1985885855 0.6038098812 -2.1567701991
H 7.0365835954 0.7194941030 -2.8388292955
C 6.4005912685 0.6729188354 -0.7784529178
H 7.3973648820 0.8414622455 -0.3793910981
C 5.3211351354 0.5185421586 0.0943654482
H 5.4875807511 0.5687263926 1.1683734515
C 4.0263093605 0.3052883043 -0.3953326221
N 2.0403388967 -1.0306480074 0.2437497067
C 2.3624611875 -2.4423662937 0.6615004510
C 2.8281820836 -3.2114552744 -0.5901186843
C 1.0637312386 -3.0552425847 1.2161869787
C 3.4554376515 -2.4693616848 1.7379192131
H 3.7614504604 -2.7959158075 -0.9839388823
H 2.0644181912 -3.1552127679 -1.3693873458
H 2.9966124050 -4.2640406728 -0.3378934062
H 0.7513042190 -2.5478345112 2.1356136755
H 1.2258812996 -4.1131239548 1.4500688788
H 0.2490794060 -2.9753889449 0.4939390668
H 3.1473758020 -1.9456578285 2.6495278464
H 4.3995357150 -2.0448002728 1.3824072253
H 3.6445112374 -3.5127657448 2.0107251520
O 1.1446536071 -0.9017091596 -0.6717008368
C -1.2406643657 1.7088413149 0.2773702896
N -1.5090808935 0.2956308983 -0.0214456700
S -2.6558351639 -0.5594453208 0.8373059493
O -2.3377973658 -1.9742488116 0.6024079509
O -2.8031002046 -0.0452140395 2.2082735555
C -4.1796010465 -0.1571993627 -0.0245320059
C -5.2169405726 0.4654850712 0.6647801089
C -4.3264717937 -0.5159932171 -1.3683046772
C -6.4118409676 0.7401577864 -0.0045261706
H -5.0838152304 0.7256031647 1.7092446259
C -5.5223656209 -0.2362285776 -2.0189082855
H -3.5109077312 -1.0043077914 -1.8918201390
C -6.5843982456 0.3955951737 -1.3490777467
H -5.6385118951 -0.5124336311 -3.0644746886
C -0.4461901384 2.0099102422 1.5642210895
H -0.2240024838 3.0869652666 1.5657276675
H -1.0927443798 1.8174246812 2.4256935329
C 0.8434470458 1.1916331620 1.7388390450
H 0.5662014229 0.1335882918 1.7811769482
H 1.2762402686 1.4261117893 2.7218988069
C 1.9261727203 1.4173288755 0.6553378016
H 1.4412478308 1.5033942362 -0.3222446089
C 2.7118315472 2.7263675293 0.9337247422
H 3.5962905802 2.5050830394 1.5477524568
C 3.1297311814 3.5281929232 -0.3054019360
H 3.6338704698 4.4566891071 -0.0122100724
H 3.8096811294 2.9680801469 -0.9523939506
H 2.2518783508 3.8034428460 -0.9033006349
H -7.2222252771 1.2284779066 0.5311576867
C -7.8798973748 0.6828947150 -2.0703634156
H -7.7064680550 1.2553686968 -2.9895243557
H -8.3856912501 -0.2463193837 -2.3617079445
H -8.5700407274 1.2547685717 -1.4427316674
H -2.2022885273 2.2329693769 0.3124807971
H 3.2633229637 0.0044393655 1.5751613625
H -0.6863519338 -0.2828488423 -0.2174680390
H -0.7147420826 2.0968566438 -0.6019850126
H 2.0806063413 3.3724823241 1.5568637889
65

C 2.7313664968 -0.0662345397 0.3131116396
C 3.4875230908 0.5511601816 -2.0495110580
H 2.4626630423 0.8250229222 -2.2773232162
C 4.4799228635 0.6702192356 -3.0230277029
H 4.2222059145 1.0542892602 -4.0066910341
C 5.7964340326 0.2989899051 -2.7411402962
H 6.5669291441 0.3941430467 -3.5015631715
C 6.1146776095 -0.1980228651 -1.4768991752
H 7.1348753870 -0.4930112636 -1.2455907113
C 5.1202856513 -0.3219174009 -0.5052592525
H 5.3770930481 -0.7103878531 0.4782325543
C 3.7974285366 0.0520922765 -0.7760734638
N 1.5533684552 -0.8234788476 -0.1822724809
C 1.4075659957 -2.3145818127 -0.0050452569
C 1.2744245838 -2.6378057899 1.4964383606
C 2.6340702603 -3.0279943573 -0.6077970324
C 0.1351959514 -2.7592638617 -0.7365665578
H 0.3835173450 -2.1644979076 1.9191540838
H 2.1517591874 -2.3211976785 2.0707012888
H 1.1784462984 -3.7220681200 1.6225056368
H 2.4974014708 -4.1118848149 -0.5231343673
H 3.5616300900 -2.7682692021 -0.0894207146
H 2.7536903224 -2.7765662250 -1.6661445600
H -0.7518377823 -2.2858724843 -0.3097910159
H 0.0341293258 -3.8442173816 -0.6250575315
H 0.1811503908 -2.5187303996 -1.8010818269
O 0.7421638809 -0.1953061724 -0.9593697049
C -0.7972790985 2.4080098201 0.8820701332
N -1.4598238210 1.2060050961 0.3427092154
S -2.5366687204 0.3659579973 1.3074385023
O -1.9293404475 -0.8773894705 1.8228559017
O -3.1136735742 1.3390179980 2.2447220764
C -3.7928644429 -0.1354502149 0.1265141709
C -4.2076558083 -1.4652315466 0.1019743186
C -4.3986767711 0.8177688562 -0.6970752948
C -5.2329484862 -1.8436778525 -0.7664772389
H -3.7291751011 -2.1874668592 0.7542895575
C -5.4181003046 0.4236173636 -1.5566295708
H -4.0653727693 1.8498132704 -0.6701170016
C -5.8511576682 -0.9118135251 -1.6074446470
H -5.8879017879 1.1630441272 -2.2010984594
C 0.1198721672 2.2205406011 2.1036419370
H 0.5675667881 3.1985764373 2.3168290120
H -0.5125459681 1.9779529968 2.9657031568
C 1.2020187860 1.1288003044 1.9904366861
H 0.6819043579 0.1788662274 1.8378869996
H 1.6979792374 1.0377432695 2.9671963881
C 2.2873801585 1.3147860817 0.8977803960
H 1.8673120311 1.8814306052 0.0581532874
C 3.5313870267 2.0710656235 1.4163923640
H 4.2659230247 2.1426820801 0.6074729663
C 3.2744173642 3.4828018683 1.9569282841
H 2.7592747868 4.1075701415 1.2169811147
H 2.6711855024 3.4759717080 2.8701819804
H 4.2245300976 3.9734073603 2.1977813080
H -5.5565287530 -2.8814362686 -0.7879039691
C -6.9460508292 -1.3263391476 -2.5614682083
H -7.7972287532 -0.6372822403 -2.5163232133
H -6.5873476442 -1.3273923208 -3.5989915109
H -7.3131056700 -2.3326496702 -2.3376854512
H -1.5771113485 3.1312436808 1.1351318623
H 3.1495367975 -0.6583791612 1.1338287700
H -0.8159616292 0.5689322251 -0.1399326089
H -0.2402074128 2.8181484897 0.0327343852
H 4.0063571029 1.4696464818 2.2068045728
65

C 2.5840976094 0.2057848849 0.4320717235
C 2.5202366602 0.1922819538 -2.1218044956
H 1.4550492571 -0.0099790006 -2.0684447976
C 3.1514496293 0.3161242488 -3.3590262744
H 2.5688588538 0.2175704245 -4.2712639654
C 4.5247317012 0.5658560928 -3.4306441026
H 5.0130504785 0.6631966932 -4.3964578351
C 5.2654395116 0.6825647251 -2.2546782213
H 6.3350217052 0.8701677251 -2.2980956946
C 4.6340603036 0.5516089952 -1.0156902526
H 5.2196010015 0.6393712899 -0.1029027837
C 3.2560427998 0.3148455339 -0.9332913724
N 1.7340189583 -1.0173496016 0.4969670290
C 2.2260221816 -2.4086087913 0.8004913711
C 3.6341434653 -2.3785027043 1.4085270828
C 2.2297507839 -3.2096654179 -0.5161613019
C 1.2335507994 -3.0278040267 1.8022558694
H 3.6626727183 -1.8196023500 2.3503642658
H 4.3743359887 -1.9584618015 0.7202431416
H 3.9368638184 -3.4070289243 1.6302421842
H 1.2341899783 -3.1952668248 -0.9664673416
H 2.5093304617 -4.2499130203 -0.3173415126
H 2.9437799204 -2.7896558236 -1.2317536519
H 0.2126031737 -2.9990869883 1.4152297218
H 1.2530119261 -2.4899989852 2.7567061829
H 1.5064635957 -4.0710113242 1.9938222382
O 0.5469257240 -0.9319396731 0.0039180286
C -1.3088152720 1.5551358874 1.8764386804
N -1.4641351883 0.0948917817 1.9276041311
S -2.9798488853 -0.5854116158 1.7197846907
O -2.7577244112 -2.0333048768 1.7756648222
O -3.8964487906 0.0980237034 2.6372909975
C -3.5119793925 -0.1878447688 0.0460050304
C -4.5382355860 0.7340841971 -0.1536879755
C -2.8800902792 -0.8010679977 -1.0408019349
C -4.9361746591 1.0411211702 -1.4557364617
H -5.0204842957 1.1906137475 0.7040413743
C -3.2872354860 -0.4796806484 -2.3320398741
H -2.0818680138 -1.5180276919 -0.8755977690
C -4.3219495183 0.4414314930 -2.5615617892
H -2.7971594403 -0.9547333279 -3.1788994170
C -0.0642778838 1.9964501718 2.6655096594
H 0.0591563548 3.0787184955 2.5199876316
H -0.2625194304 1.8410605784 3.7326051532
C 1.2336726356 1.2505384470 2.3086887508
H 1.0751809797 0.1876631066 2.5190896758
H 2.0259484276 1.5762001817 2.9977659937
C 1.7351077389 1.4417176255 0.8553088429
H 0.8744312550 1.4714319611 0.1795931002
C 2.5130376855 2.7774222335 0.7228534986
H 3.5797133706 2.6072525054 0.9271327794
C 2.3512651973 3.5158070539 -0.6117884101
H 1.2942592321 3.7352234626 -0.8073844118
H 2.8886255920 4.4712268612 -0.5903116158
H 2.7323021050 2.9366447301 -1.4566093726
H -5.7396091249 1.7569201665 -1.6127230990
C -4.7736249092 0.7549331958 -3.9683960959
H -5.3448981780 1.6877047436 -4.0079666193
H -3.9220547949 0.8485525342 -4.6516113969
H -5.4175472088 -0.0421249326 -4.3629102352
H -2.1966918838 1.9983584387 2.3350522936
H 3.3675851963 0.0783547441 1.1835071714
H -0.7888048633 -0.4035548691 1.3398649686
H -1.2598774000 1.9122343895 0.8368696135
H 2.1677474000 3.4462664498 1.5215710172

9-PBN

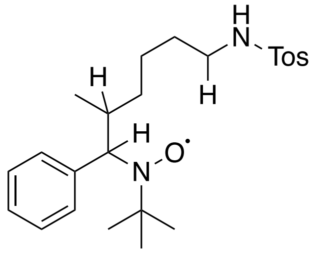

Name	E(B3LYP)	H(B3LYP)	g-factor	α_N	α_H
Tosyl_N_radical_hexane_C6_radical_PBN_adduct_0034	-1668.884597	-1668.291769	2.00578310	14.852520	2.105480
Tosyl_N_radical_hexane_C6_radical_PBN_adduct_0023	-1668.883001	-1668.290683	2.00580567	14.664560	1.997850
Tosyl_N_radical_hexane_C6_radical_PBN_adduct_0104	-1668.882381	-1668.290135	2.00621927	15.021740	1.769650
Tosyl_N_radical_hexane_C6_radical_PBN_adduct_0106	-1668.882740	-1668.289990	2.00588413	14.985110	4.005260
Tosyl_N_radical_hexane_C6_radical_PBN_adduct_0272	-1668.882147	-1668.289814	2.00620050	14.739910	2.405460
Tosyl_N_radical_hexane_C6_radical_PBN_adduct_0329	-1668.881876	-1668.289757	2.00619183	14.722270	2.582710
Tosyl_N_radical_hexane_C6_radical_PBN_adduct_0099	-1668.882032	-1668.289688	2.00621560	14.861820	2.449870
Tosyl_N_radical_hexane_C6_radical_PBN_adduct_0170	-1668.881658	-1668.289516	2.00618207	15.025510	1.995360
Boltzman averaged for 298.15 K			2.005966	14.8036	2.2686

65

C 2.7010387647 -0.4142004032 -0.3917177074
C 3.1616471568 1.6528320322 1.0576189352
H 2.0986997978 1.8693414295 1.0560656805
C 4.0460954845 2.4893071253 1.7395893270
H 3.6604420928 3.3523536504 2.2762055344
C 5.4172039309 2.2257873304 1.7339156896
H 6.1030694503 2.8797806802 2.2656893108
C 5.9008801684 1.1217181087 1.0316279023
H 6.9670370062 0.9117651751 1.0087520926
C 5.0165673316 0.2886638269 0.3443291928
H 5.4058998916 -0.5601366248 -0.2132242730
C 3.6365125632 0.5357743180 0.3552614440
N 1.6068189160 0.3221064407 -1.0666419318
C 1.6229149568 0.5819554151 -2.5499717685
C 0.4922897831 1.5638858077 -2.8810496767
C 1.3838234017 -0.7549886267 -3.2820842486
C 2.9778589978 1.1988027155 -2.9469038431
H 0.6024056212 2.4965365323 -2.3195505850
H -0.4850834615 1.1308774760 -2.6636077116
H 0.5388221058 1.7968699424 -3.9502737538
H 2.1706524151 -1.4869378057 -3.0655544592
H 1.3818313614 -0.5850628127 -4.3647390918
H 0.4150552222 -1.1764881670 -2.9984114997
H 3.8209754993 0.5252385929 -2.7649726619
H 3.1609968446 2.1299888738 -2.4015285385
H 2.9658212882 1.4246842204 -4.0185399945
O 0.5842973707 0.6812944924 -0.3697954916
C -1.5136955038 -2.1596117717 1.4533176736
N -1.3002908336 -1.5737580078 0.1201900438
S -2.6193082767 -1.2484043045 -0.8544222087
O -2.0569372072 -0.7784482504 -2.1266621667
O -3.5075043225 -2.4133747821 -0.7962468482
C -3.5025940059 0.1338287302 -0.1133603224
C -2.8909943778 1.3905616155 -0.0490783092
C -4.7855720873 -0.0633809148 0.3940295538
C -3.5819545190 2.4496932895 0.5315149262
H -1.8882038453 1.5348079050 -0.4407086856
C -5.4642343878 1.0125061684 0.9685018334
H -5.2406403289 -1.0458755264 0.3297591101
C -4.8782933421 2.2813880189 1.0448141968
H -6.4663923362 0.8610082020 1.3623977602
C -0.3343137865 -3.0429160031 1.9006689222
H -0.7312898810 -3.7792973463 2.6124345710
H 0.0077414225 -3.6157529387 1.0304287352
C 0.8409447736 -2.3307480294 2.5973270721
H 1.5621406801 -3.0988177920 2.9063198571
H 0.4681388547 -1.8879026068 3.5320024800
C 1.5662599864 -1.2138959265 1.8221824637
H 2.3763998716 -0.8346947574 2.4588131447
H 0.8838621309 -0.3727363281 1.6769716150
C 2.1596969256 -1.6186229837 0.4559720285
H 1.3641487789 -2.0598890076 -0.1571974462
C 3.2644803413 -2.6771077634 0.6144245071
H 4.0941331218 -2.3015946547 1.2248259974
H 2.8753132961 -3.5762429427 1.1022589307
H 3.6700458917 -2.9853673029 -0.3570366615
H -3.1085727619 3.4273993498 0.5859849936
C -5.6250024827 3.4497947472 1.6435498360
H -4.9702240233 4.0650640253 2.2707670528
H -6.0280533340 4.1039553248 0.8592243392
H -6.4673890729 3.1157196913 2.2572651239
H -2.4059112885 -2.7844938973 1.3677453491
H 3.2794266080 -0.8631491759 -1.2035068625
H -0.6211185102 -0.8088569836 0.0540094868
H -1.7216677842 -1.3845260796 2.2078338625
65

C 2.8073140406 -0.4965044329 -0.5158496371
C 4.0403355688 1.2442500110 0.9145499511
H 3.0963304851 1.7522685584 1.0803638301
C 5.2139019621 1.7427578689 1.4815659456
H 5.1723561919 2.6375461852 2.0973844130
C 6.4349990264 1.1026309090 1.2611454317
H 7.3468392539 1.4939737112 1.7042947105
C 6.4765260534 -0.0379013668 0.4588990096
H 7.4219707544 -0.5393123048 0.2688319292
C 5.3035005149 -0.5324531337 -0.1136605756
H 5.3497109260 -1.4125345704 -0.7510642294
C 4.0696476629 0.0939742537 0.1130236732
N 1.8733065087 0.5547851120 -0.9752453514
C 1.8170588521 0.9941482292 -2.4149505024
C 0.8465165448 2.1769577987 -2.5145885946
C 1.2954157135 -0.1769500724 -3.2730997806
C 3.2242230562 1.4261931393 -2.8717323005
H 0.8151880715 2.5120065530 -3.5570474129
H 1.1732310519 3.0122945335 -1.8894963173
H -0.1596854778 1.8846953904 -2.2092153460
H 1.2248568604 0.1431705570 -4.3188537533

H 0.3007935374 -0.4831324407 -2.9373981324
H 1.9660557439 -1.0432206985 -3.2434173645
H 3.9486229130 0.6068923153 -2.8268069880
H 3.6021641358 2.2482153730 -2.2557201620
H 3.1759692441 1.7683121173 -3.9114603383
O 1.1282557964 1.1397167932 -0.1033467294
C -1.3597219210 -0.9127664955 2.1221567304
N -1.3570698216 -0.1797076557 0.8485008761
S -2.3342856962 -0.6829889044 -0.3986997006
O -1.7808205477 -0.0588372303 -1.6110167934
O -2.5598509820 -2.1355299170 -0.3525699942
C -3.9125880752 0.0903635222 -0.0282266269
C -5.0550594501 -0.6990883557 0.0761684187
C -3.9886332197 1.4809451539 0.1004145928
C -6.2865463047 -0.0868767202 0.3200006407
H -4.9727137861 -1.7751456942 -0.0313416048
C -5.2223020576 2.0738714301 0.3420144852
H -3.0900589210 2.0841761188 0.0194627564
C -6.3911737409 1.3014156771 0.4548820699
H -5.2836084428 3.1547972444 0.4458693882
C -0.5198929341 -2.2075477894 2.1939782933
H -1.0158160694 -2.8829582628 2.9045281595
H -0.5726800132 -2.7100508484 1.2228341507
C 0.9349977287 -2.0365432596 2.6686308007
H 1.3816205378 -3.0349191476 2.7647481937
H 0.9170759734 -1.6263846227 3.6890416719
C 1.8530878466 -1.1298321732 1.8281561762
H 2.8300819350 -1.0824517243 2.3267848509
H 1.4609445343 -0.1097058478 1.8373979684
C 2.0689945345 -1.5661190630 0.3636357300
H 1.0902829192 -1.6969228323 -0.1150431214
C 2.8096115221 -2.9112406573 0.2782377106
H 2.9396258488 -3.2336354723 -0.7620534107
H 3.8012329072 -2.8514188253 0.7423052500
H 2.2503204079 -3.6986088553 0.7929244877
H -7.1791171379 -0.7017636239 0.4054175322
C -7.7240886525 1.9646068977 0.7088275325
H -7.6870607254 2.6082367583 1.5959371427
H -8.0179434562 2.5997153505 -0.1364159293
H -8.5168140171 1.2260536241 0.8615551091
H -2.4062232444 -1.1452133282 2.3447154114
H 3.1164062907 -1.0174773616 -1.4261784219
H -0.4811050242 0.2298926683 0.5070955323
H -1.0356012345 -0.2018215068 2.8926069008
65

C 2.9091859347 0.3131645292 0.0400054865
C 4.0086880262 -1.6401656960 -1.1790337823
H 4.9573651970 -1.3326667758 -0.7505552444
C 3.9458536793 -2.7440688377 -2.0287056919
H 4.8495322473 -3.3091550189 -2.2430515437
C 2.7311037908 -3.1225494763 -2.6066810785
H 2.6853297247 -3.9831243223 -3.2691971060
C 1.5799584405 -2.3843745352 -2.3320889161
H 0.6306580877 -2.6648446203 -2.7819386281
C 1.6407803774 -1.2762073287 -1.4827464083
H 0.7353330778 -0.7094838346 -1.2766362263
C 2.8542793023 -0.8945182314 -0.8937090537
N 3.8870907693 1.3211034643 -0.4532375436
C 3.5848208734 2.3513509010 -1.5078678177
C 4.0880661228 1.8204380048 -2.8647400760
C 4.3593468566 3.6220863381 -1.1149101051
C 2.0833904215 2.6604762061 -1.5747108175
H 3.5269821529 0.9331643919 -3.1742615348
H 5.1458464279 1.5538950018 -2.7896379340
H 3.9721759170 2.5891584824 -3.6373698066
H 4.2352781465 4.3883661164 -1.8877543279
H 5.4220640039 3.3989896533 -1.0020328033
H 3.9874554359 4.0257070887 -0.1664198961
H 1.4923639543 1.8031721462 -1.9105841911
H 1.9252708927 3.4691714467 -2.2963445862
H 1.6936325993 2.9994924945 -0.6085772970
O 5.1316933902 1.0788318785 -0.2405238538
C -1.5416807268 -1.5695980522 2.8273909827
N -2.2096285236 -1.4112300021 1.5203197735
S -2.3515303883 0.1036085249 0.7927082716
O -2.1290496958 1.1073220533 1.8389358429
O -1.5646849918 0.1070101541 -0.4525529983
C -4.0770065254 0.1712485345 0.3114470843
C -5.0410070082 0.5041858126 1.2666244722
C -4.4382099283 -0.1000390648 -1.0069941804
C -6.3792319526 0.5515091029 0.8900285103
H -4.7417304111 0.7332043962 2.2840723447
C -5.7855705069 -0.0486025578 -1.3658331999
H -3.6731856148 -0.3341159810 -1.7393549237
C -6.7741765627 0.2759747467 -0.4290813482
H -6.0706178338 -0.2591815980 -2.3936044465
C -0.0394833798 -1.8786753270 2.7551985588
H 0.2959168917 -2.1242344664 3.7732434296
H 0.1085820874 -2.7945379810 2.1622809605
C 0.8237246750 -0.7483082421 2.1819899410
H 0.6965751608 0.1479403062 2.8019450253
H 0.4486595253 -0.4871000637 1.1867994192
C 2.3079009275 -1.1321690001 2.0851330341
H 2.3997951985 -2.0436073645 1.4807335051
H 2.6762995215 -1.3935550194 3.0876682017
C 3.2520332850 -0.0500991035 1.5129939895
H 4.2601810852 -0.4788456525 1.5005774122
C 3.2956894581 1.2048462003 2.4002322480
H 3.4764005577 0.9254443841 3.4452528770
H 4.1013148081 1.8735892629 2.0874772836
H 2.3537006885 1.7657262532 2.3656180126
H -7.1319909462 0.8114056549 1.6304872455
C -8.2285263826 0.3569086936 -0.8273449578
H -8.8761506388 -0.1030292384 -0.0726526957
H -8.5496749307 1.4011589968 -0.9350838647
H -8.4114691384 -0.1439329041 -1.7829343021
H -2.0635795590 -2.3813044374 3.3488760421
H 1.9319332411 0.7984458889 0.0273983600
H -1.9284131924 -2.0960532269 0.8220215990
H -1.7230926699 -0.6502563448 3.3898277133
65

C 2.7345617021 -0.4798598328 -0.4015758624
C 2.9293162135 1.6996249796 0.9371993837
H 1.8555729487 1.8287056398 0.8487002186
C 3.6930659922 2.6436041403 1.6243135538
H 3.2037327982 3.5027705430 2.0756996988
C 5.0769927852 2.4909435250 1.7324268110
H 5.6689949582 3.2285277118 2.2674625207
C 5.6949277368 1.3893469033 1.1403759504
H 6.7724109643 1.2643797450 1.2082449012
C 4.9311343642 0.4479279499 0.4478654768
H 5.4237282725 -0.4003647877 -0.0218628485
C 3.5394802170 0.5842055033 0.3446716339
N 1.6577446626 0.1259232430 -1.2202837198
C 1.8300839345 0.6491759141 -2.6252237513
C 1.5701534227 2.1675420887 -2.5972732293
C 0.7860078961 -0.0652906635 -3.5027785799
C 3.2426419292 0.3756434310 -3.1601981323
H 2.3156043013 2.6822581250 -1.9816617167
H 0.5777920795 2.3764078677 -2.1921777500
H 1.6240852009 2.5695650117 -3.6145263052
H 1.0030338548 -1.1372979056 -3.5690870187
H 0.8088096267 0.3505689001 -4.5163055021
H -0.2201477866 0.0474165277 -3.0943224443
H 3.3191370045 0.8112633521 -4.1617836513
H 3.4479619439 -0.6957959695 -3.2587043116
H 4.0191853357 0.8335052469 -2.5392133712
O 0.5365596708 0.3989868689 -0.6476404190
C -1.5536999264 -2.2726414771 1.4255529116
N -1.3917756818 -1.7779526014 0.0491136682
S -2.7638376187 -1.3947720161 -0.8313095058
O -2.2874584271 -1.0029125540 -2.1608545931
O -3.7178341804 -2.4935119118 -0.6497817287
C -3.4891991049 0.0665024947 -0.0687078547
C -4.6904518613 -0.0430754452 0.6297860417
C -2.8331369596 1.2972558813 -0.1760497526
C -5.2408769012 1.0940140645 1.2232548895
H -5.1853804139 -1.0061774948 0.6936180861

C -3.3950179760 2.4191536201 0.4258091503
H -1.8957404857 1.3706662093 -0.7191641454
C -4.6070550022 2.3386574549 1.1303608589
H -2.8866847784 3.3774479704 0.3456421926
C -0.3353213047 -3.0842824160 1.9053605447
H -0.7011021774 -3.8233490950 2.6304647131
H 0.0487785470 -3.6603491179 1.0549297910
C 0.7877277938 -2.2912745372 2.6003270881
H 1.5281490352 -3.0109529009 2.9732541941
H 0.3652823103 -1.8167717603 3.4975378036
C 1.4939114366 -1.1874959219 1.7902205820
H 2.2457166774 -0.7229594353 2.4415615387
H 0.7753916058 -0.3991769666 1.5513233007
C 2.1849949717 -1.6476774180 0.4888904675
H 1.4475839245 -2.1600715020 -0.1418126531
C 3.3224233439 -2.6426386956 0.7784350195
H 4.0995341483 -2.1892301235 1.4053432677
H 2.9465515805 -3.5251162888 1.3049047916
H 3.7950166033 -2.9940973999 -0.1468288792
H -6.1799528310 1.0107336382 1.7652353789
C -5.2190377299 3.5726150909 1.7503116316
H -5.9653253923 3.3131701411 2.5077637854
H -4.4582041021 4.2026828614 2.2245059768
H -5.7205199958 4.1880879502 0.9918081445
H -2.4291901655 -2.9256979088 1.4050670881
H 3.4095247622 -0.9500980982 -1.1186951141
H -0.6711799726 -1.0652224994 -0.1027176386
H -1.7676339600 -1.4547394457 2.1328624444
65

C 2.9824003190 -0.7767157811 -1.4795355909
C 3.0785464406 1.5249343449 -0.3817259322
H 3.6936075465 1.8606017627 -1.2108503955
C 2.7361025949 2.4046699881 0.6453444517
H 3.0801162633 3.4350839517 0.6056255764
C 1.9528700662 1.9699516694 1.7177406572
H 1.6812106304 2.6590349412 2.5127219501
C 1.5245041703 0.6418829394 1.7681493642
H 0.9071910034 0.2943828051 2.5909287184
C 1.8768997531 -0.2406936875 0.7414089858
H 1.5456885230 -1.2763536357 0.7908724670
C 2.6458452898 0.1906383727 -0.3483802178
N 4.4391422099 -0.7621383050 -1.7736698278
C 5.4761173851 -1.5292473911 -0.9978889522
C 6.4869453417 -2.0657998740 -2.0273391495
C 4.8486264527 -2.6974698144 -0.2263588651
C 6.1730615814 -0.5573530361 -0.0256529261
H 6.0099155633 -2.7847399804 -2.7030203514
H 7.3121344235 -2.5708642990 -1.5137966317
H 6.8894466977 -1.2449852098 -2.6245208786
H 4.3301015571 -3.3958352961 -0.8927804412
H 4.1514746667 -2.3583726960 0.5462947188
H 5.6474870265 -3.2537886737 0.2752546114
H 5.4746350042 -0.1872645032 0.7314198192
H 6.5720072941 0.2975491430 -0.5779218294
H 6.9997703114 -1.0637511117 0.4851776032
O 4.8773010457 0.1972802913 -2.5092058600
C -2.1461588191 -0.1519169927 -1.0946248400
N -1.8294418971 0.3918864453 0.2399235171
S -2.4754357837 -0.4271346710 1.5693462128
O -2.6158564957 -1.8653044807 1.2974274506
O -1.6872966731 0.0356465129 2.7198937069
C -4.1264958804 0.2620976292 1.6693374660
C -5.2288386809 -0.5720158954 1.4988519057
C -4.2891583803 1.6203113710 1.9598113813
C -6.5120395011 -0.0334307098 1.6133037841
H -5.0763850682 -1.6242544327 1.2843728228
C -5.5740393601 2.1390811372 2.0693506707
H -3.4196223810 2.2548873773 2.0955724433
C -6.7053494053 1.3224949533 1.8992515500
H -5.7057839556 3.1951894803 2.2927832872
C -1.5531850707 0.7294200255 -2.2003384099
H -1.9384080099 0.3495219927 -3.1569221053
H -1.9476321920 1.7463055205 -2.0824510573
C -0.0156367616 0.7862950257 -2.2663937440
H 0.3870426465 1.2264551388 -1.3474207492
H 0.2592820384 1.4854004411 -3.0679555070
C 0.6493878057 -0.5704410216 -2.5540326025
H 0.1773271858 -0.9856949418 -3.4552259490
H 0.4210108063 -1.2851848113 -1.7510575262
C 2.1829409095 -0.5323100626 -2.7932763536
H 2.4636947146 0.4594666523 -3.1644149777
C 2.5795059571 -1.5672763568 -3.8596693221
H 2.0386688029 -1.3740424432 -4.7930860883
H 3.6493300753 -1.5289754229 -4.0771334685
H 2.3254252305 -2.5862044254 -3.5369801987
H -7.3750981468 -0.6807467908 1.4795413314
C -8.0931505424 1.9015660207 2.0374364001
H -8.8604072548 1.1801905533 1.7407939207
H -8.2963497245 2.1963041790 3.0748311355
H -8.2145257753 2.7987261552 1.4191314898
H -3.2376640529 -0.1549651503 -1.1889970194
H 2.7399354603 -1.7880176464 -1.1427058344
H -0.8298442810 0.5069593134 0.4154934632
H -1.8126220908 -1.1926836528 -1.1942668956
65

C 3.4610751965 -1.1661122279 -0.7148819139
C 2.2462796842 0.6452685172 -2.0395948425
H 2.6099339379 0.1808739831 -2.9510278706
C 1.4101903388 1.7604054726 -2.0983843466
H 1.1221705443 2.1648057784 -3.0655850902
C 0.9348812843 2.3514101352 -0.9233226974
H 0.2678839567 3.2076745689 -0.9666205672
C 1.3249289990 1.8365171996 0.3149274620
H 0.9507607881 2.2854552527 1.2295272518
C 2.1748856509 0.7292917857 0.3717140172
H 2.4675724476 0.3285507553 1.3399933117
C 2.6263004664 0.1079350372 -0.8001897736
N 4.6595934504 -1.0820865719 -1.5888057074
C 5.9400230001 -0.3839068137 -1.2170641149
C 7.0890048361 -1.2655663805 -1.7384999362
C 6.0636565479 -0.2107623971 0.3023345347
C 5.9650118186 0.9895774935 -1.9160159011
H 7.1029609019 -2.2323624566 -1.2226193642
H 8.0502842369 -0.7693574836 -1.5666055345
H 6.9691722736 -1.4468772369 -2.8086454971
H 6.0350238783 -1.1723421706 0.8269641229
H 5.2844481598 0.4407875535 0.7102985603
H 7.0296829776 0.2554424726 0.5232658988
H 5.1699248745 1.6395445924 -1.5373815901
H 5.8252111094 0.8607357596 -2.9924937751
H 6.9276194727 1.4835950456 -1.7422005158
O 4.4920056346 -1.3279949041 -2.8399765297
C -2.3241932387 -1.3882736709 -0.1390966490
N -2.2273452017 0.0816227558 -0.0473933364
S -2.5094178254 0.8291776898 1.4358126467
O -2.1426997205 -0.0344559663 2.5682799709
O -1.9144444746 2.1655144334 1.2982871030
C -4.2974236265 0.9732722707 1.4503076343
C -5.0213898728 0.3883836793 2.4864594270
C -4.9402274510 1.7156541744 0.4545890959
C -6.4089966085 0.5421540337 2.5178522919
H -4.4998696664 -0.1757990668 3.2519474928
C -6.3219299471 1.8585615380 0.5018184066
H -4.3625048595 2.1674047404 -0.3450352627
C -7.0788606361 1.2760197725 1.5330670621
H -6.8257460702 2.4321956319 -0.2726592151
C -1.0683830125 -2.1640761865 0.2857764324
H -0.7937218404 -1.8481742260 1.2987722440
H -1.3303899876 -3.2300697806 0.3480899776
C 0.1177109036 -1.9834402645 -0.6720336515
H 0.2861231060 -0.9181877120 -0.8567543000
H -0.1287925541 -2.4271202779 -1.6480724395
C 1.4111844683 -2.6207645164 -0.1402303108
H 1.2242745990 -3.6940015404 0.0019885301
H 1.6216137411 -2.2264732859 0.8647523904
C 2.6668731811 -2.4678603976 -1.0388020148
H 2.3526230849 -2.4029160549 -2.0864013467
C 3.5785019410 -3.6975652807 -0.8914209661

H 3.0292449452 -4.6089411923 -1.1537269412
H 4.4509944343 -3.6309942818 -1.5455508823
H 3.9271455994 -3.8104297564 0.1443027508
H -6.9771717391 0.0840910558 3.3235639866
C -8.5779767938 1.4531470993 1.5742040455
H -8.8470154535 2.4996720630 1.7656385556
H -9.0392097759 1.1725627671 0.6199113041
H -9.0325681382 0.8437687622 2.3610053874
H -2.5820536486 -1.6184258100 -1.1807510604
H 3.8258847294 -1.2637631913 0.3110394180
H -1.3838875835 0.4888380943 -0.4501114076
H -3.1827927710 -1.6904690012 0.4688050522
65

C 2.9676538078 0.2810365723 0.3290006392
C 4.1289806425 -1.1065792517 -1.4691519226
H 5.0690264108 -0.9263885870 -0.9566761328
C 4.0979615520 -1.8708156607 -2.6352638814
H 5.0194094010 -2.3001938483 -3.0206225423
C 2.8916076461 -2.0850774720 -3.3074310134
H 2.8702667168 -2.6833116318 -4.2147048032
C 1.7160277878 -1.5255682360 -2.8070859125
H 0.7701664269 -1.6857694288 -3.3176270828
C 1.7455428487 -0.7545258287 -1.6421670793
H 0.8172097045 -0.3342933362 -1.2620799182
C 2.9506744257 -0.5398805148 -0.9594645748
N 3.9817567944 1.3665837868 0.2616274635
C 3.7684059485 2.6860785842 -0.4298700792
C 2.2754992082 3.0144543639 -0.5624085372
C 4.4282233854 2.6196500014 -1.8212857117
C 4.4622961566 3.7527741602 0.4361115628
H 1.7775312053 3.0465817519 0.4133323118
H 1.7486514817 2.3039011702 -1.2066047841
H 2.1750438523 4.0063682743 -1.0159781952
H 4.3752721378 3.5987270792 -2.3110096836
H 3.9280673457 1.8856505909 -2.4604951472
H 5.4784365316 2.3334216184 -1.7200117785
H 3.9801433007 3.8312338166 1.4171950707
H 4.4050133472 4.7299291571 -0.0556652992
H 5.5116530398 3.4919275866 0.5876804162
O 5.2050305475 1.0429058903 0.4908819263
C -1.6888068005 -2.1343532314 2.3761525760
N -2.3265449492 -0.9425278645 1.7725517149
S -2.6554603950 -0.9694685549 0.1119100711
O -1.6319571542 -0.2006268516 -0.6188265766
O -2.9318102494 -2.3686110999 -0.2293621497
C -4.1795603482 -0.0317680377 0.0153120027
C -4.1701316431 1.2422019438 -0.5477029251
C -5.3669850491 -0.6044272286 0.4812504522
C -5.3673362820 1.9554311147 -0.6324493997
H -3.2407144732 1.6596622805 -0.9194414337
C -6.5492697987 0.1205395480 0.3880801291
H -5.3603927564 -1.6039175205 0.9034960103
C -6.5699125861 1.4107515805 -0.1686444744
H -7.4750580856 -0.3207714441 0.7495607594
C -0.2565970432 -2.4544411002 1.9241416222
H 0.0886901018 -3.3119067826 2.5198266363
H -0.2766061970 -2.7915285879 0.8804507460
C 0.7356339767 -1.2925390933 2.0659009311
H 0.6904185645 -0.8884329074 3.0877655033
H 0.4236482246 -0.4832620153 1.3933906179
C 2.1773985251 -1.7177112282 1.7370232440
H 2.1711439586 -2.3057965395 0.8117325409
H 2.5281513478 -2.3975468682 2.5261288022
C 3.2128864515 -0.5778840709 1.6035930644
H 4.1937693598 -1.0481573191 1.4739254397
C 3.2783700741 0.2906082278 2.8701563029
H 4.1381917234 0.9633042331 2.8365548727
H 2.3734217938 0.8983568274 2.9973504438
H 3.3781224787 -0.3425925233 3.7602311312
H -5.3641093735 2.9500670310 -1.0710961316
C -7.8669166187 2.1766233759 -0.2750269059
H -8.5416753917 1.7106153891 -1.0043140431
H -8.3967947311 2.1987347977 0.6844120491
H -7.6980018798 3.2100658507 -0.5919867043
H -2.3463144686 -2.9828677031 2.1734750460
H 1.9978417493 0.7719328902 0.4310887851
H -1.8330726801 -0.0784062323 1.9898258046
H -1.7166775079 -1.9494759977 3.4569808326
65

C 2.3167432871 -0.1421564140 -1.2402783446
C 1.4405188892 1.5105815939 0.4988883221
H 0.5469137109 1.6340615114 -0.1035805675
C 1.5810447540 2.2119674106 1.6958392304
H 0.7847654227 2.8743041694 2.0253381109
C 2.7336330846 2.0651450582 2.4722062404
H 2.8386537384 2.6131278890 3.4047050099
C 3.7476998803 1.2095447890 2.0422964329
H 4.6489054829 1.0865236577 2.6373267040
C 3.6063998905 0.5073213320 0.8432496574
H 4.3993426591 -0.1631749001 0.5177337021
C 2.4544956089 0.6470675764 0.0583436439
N 2.0923726512 0.7716176342 -2.3971433046
C 3.1827574752 1.5164838541 -3.1195407282
C 4.5410272536 0.8237024842 -2.9486558533
C 3.2395012728 2.9539485396 -2.5661205246
C 2.7928221321 1.5344370069 -4.6083657050
H 4.8884034810 0.8403742628 -1.9110448890
H 5.2836806443 1.3578887757 -3.5506339201
H 4.5161723612 -0.2138688493 -3.3000353091
H 3.9617375194 3.5492063364 -3.1361353109
H 3.5403540168 2.9604793781 -1.5139195226
H 2.2555482297 3.4232370256 -2.6486813270
H 3.5164499879 2.1286512725 -5.1765654353
H 1.7993170904 1.9688532606 -4.7351748097
H 2.7818401511 0.5189701088 -5.0202648956
O 0.8987299361 1.2196528656 -2.5634408808
C -0.9881338245 -2.6750614384 2.3847651165
N -2.3558272539 -2.6622318383 1.8348711793
S -3.4051516318 -1.4175722636 2.2996398446
O -4.7445808992 -1.9619766232 2.0470200185
O -3.0488046320 -0.8847612720 3.6215240959
C -3.0292935485 -0.1471778866 1.0933290539
C -2.6634508603 1.1235757450 1.5309367406
C -3.1547871156 -0.4278351115 -0.2713779533
C -2.4044709966 2.1189916077 0.5859931050
H -2.5840991516 1.3214974127 2.5943908784
C -2.8826783074 0.5723459014 -1.1977584979
H -3.4554150317 -1.4181711924 -0.5971620249
C -2.4964249150 1.8601437475 -0.7869501029
H -2.9669671249 0.3550938164 -2.2595165360
C -0.1094760238 -3.6372615745 1.5727368759
H 0.8371280263 -3.7762719757 2.1127638637
H -0.5949932695 -4.6232215046 1.5552418120
C 0.1652424596 -3.1614068288 0.1348480783
H -0.7730355098 -2.7779014605 -0.2860825395
H 0.4505642863 -4.0223585033 -0.4813242841
C 1.2579311023 -2.0785978631 0.0549656198
H 2.2457360683 -2.5560417543 0.1488334565
H 1.1662617555 -1.4092043031 0.9176347275
C 1.2020711715 -1.2269132604 -1.2285572118
H 0.2430036303 -0.6968295306 -1.2391646754
C 1.2903017722 -2.0844159519 -2.5005170097
H 0.4644408095 -2.8007819386 -2.5512772165
H 1.2426010819 -1.4622105032 -3.3988126115
H 2.2278237890 -2.6569732035 -2.5276169624
H -2.1236195331 3.1135871933 0.9241395876
C -2.1599958638 2.9183066919 -1.8089964817
H -2.1314066874 3.9157474510 -1.3590362967
H -1.1779174301 2.7176857021 -2.2553221851
H -2.8931270929 2.9332918389 -2.6234362147
H -0.9861500472 -2.9473134667 3.4482983493
H 3.2664858534 -0.6509014411 -1.4243604035
H -2.8598830975 -3.5382969508 1.9669702894
H -0.5985252224 -1.6541714512 2.3176182141
65

C 2.4299315041 0.2039840493 -1.2662091371
C 2.5520979729 1.8203146732 0.7057868176

H 1.6753023167 2.3227270067 0.3098070286
C 3.1569144081 2.2770405791 1.8763422885
H 2.7343998794 3.1297206538 2.4016590398
C 4.3006405485 1.6471418359 2.3741856932
H 4.7691124497 2.0056931297 3.2868332416
C 4.8387261779 0.5571572622 1.6901873355
H 5.7291593156 0.0602946348 2.0665295063
C 4.2341987324 0.1023024081 0.5161336223
H 4.6572813683 -0.7513496858 -0.0097003880
C 3.0848750833 0.7238787747 0.0110481930
N 2.2934131370 1.2963211172 -2.2700030702
C 3.3961684315 1.7563710543 -3.1856080882
C 4.0371168024 3.0198856212 -2.5786934068
C 2.7370727329 2.0904744377 -4.5358270643
C 4.4533107613 0.6618405562 -3.3813703996
H 4.5337512387 2.7939671411 -1.6299691512
H 3.2684634582 3.7756546005 -2.3968634213
H 4.7813121087 3.4333147565 -3.2687025964
H 3.4836637632 2.5001233323 -5.2245763268
H 1.9412983286 2.8250458098 -4.3974637351
H 2.3046555380 1.1921891024 -4.9908516043
H 4.9872286481 0.4297046802 -2.4546382088
H 5.1940720331 1.0173760026 -4.1053491510
H 4.0167135976 -0.2598323739 -3.7820793727
O 1.3145010340 2.1162653313 -2.1150240176
C -1.6360835128 -3.4494812040 2.1269966937
N -2.3319162498 -2.3058681171 2.7463782215
S -3.8785660519 -1.8411969137 2.2549178907
O -4.5361974278 -2.9418936669 1.5393820039
O -4.4726384131 -1.2360644940 3.4528571282
C -3.5605390739 -0.5488760914 1.0539143494
C -3.0527576608 0.6793649968 1.4865386752
C -3.7809370683 -0.7939149869 -0.2999963133
C -2.7322260544 1.6508953369 0.5440517236
H -2.9105721224 0.8662270035 2.5461007526
C -3.4565505337 0.1939425946 -1.2313886928
H -4.1987499305 -1.7450323906 -0.6118751100
C -2.9099705883 1.4198380174 -0.8307478785
H -3.6250209601 0.0044417401 -2.2886335778
C -0.2330610507 -3.0731555216 1.6290873354
H 0.3494332831 -2.6661815247 2.4674406050
H 0.2755288484 -3.9962955134 1.3151709000
C -0.2643062227 -2.0654714754 0.4751445591
H -0.8469319819 -1.1923927042 0.7909657656
H -0.8103620927 -2.5140210302 -0.3652745123
C 1.1200449936 -1.5852109427 0.0165379043
H 1.7022111606 -2.4320472432 -0.3797007367
H 1.6662147638 -1.2205061699 0.8945480610
C 1.0442633418 -0.4647912729 -1.0437516727
H 0.3746068137 0.3154915596 -0.6645665771
C 0.4760320262 -0.9804889776 -2.3757544471
H -0.5265047956 -1.3966002502 -2.2387762592
H 0.3999275297 -0.1765310071 -3.1119254366
H 1.1116112044 -1.7732925647 -2.7938281049
H -2.3318485891 2.6055263897 0.8766497811
C -2.4874758781 2.4555551036 -1.8444273860
H -2.7078206875 3.4704071504 -1.4954984418
H -1.4058230599 2.3993014684 -2.0256129148
H -2.9940638197 2.3075001551 -2.8034623781
H -2.2529265391 -3.8146204651 1.2995850888
H 3.0921753006 -0.5456176524 -1.7071856314
H -2.3072635090 -2.2716955976 3.7611403598
H -1.5773857121 -4.2688888114 2.8540747811
65

C 2.3636083529 0.2028939562 -1.0846663354
C 2.5189221451 1.5218142578 1.0948855214
H 1.5229493226 1.9054953560 0.8983427493
C 3.2112181217 1.9147053824 2.2398772298
H 2.7399463082 2.5937822853 2.9457367753
C 4.5035791802 1.4419271100 2.4825717812
H 5.0395837076 1.7495391635 3.3764424397
C 5.1018110812 0.5744053562 1.5688458900
H 6.1069985712 0.2008777667 1.7461263554
C 4.4089409540 0.1839160458 0.4206417677
H 4.8804445371 -0.4971805509 -0.2850685877
C 3.1113055543 0.6486941867 0.1700060496
N 1.9415983734 1.3817325870 -1.8899601376
C 2.8326911305 2.1162937621 -2.8560333965
C 3.4028337032 3.3588533705 -2.1441771258
C 1.9465811144 2.5419036030 -4.0401783735
C 3.9706111842 1.2186895671 -3.3599023821
H 4.0618613851 3.0737055898 -1.3183267243
H 2.5859937631 3.9645932831 -1.7429186192
H 3.9779929112 3.9672127944 -2.8512710029
H 1.1082434476 3.1478343679 -3.6909319496
H 1.5476280193 1.6653559343 -4.5628722325
H 2.5348571228 3.1299260563 -4.7526938707
H 3.5907849121 0.3083999496 -3.8371483107
H 4.6642304740 0.9391882677 -2.5609101814
H 4.5441026024 1.7707693091 -4.1119248731
O 0.9032959370 2.0211244593 -1.4814343700
C -1.2857057770 -2.9458932304 2.5065947024
N -2.4829222736 -3.0337100781 1.6412408926
S -3.7526221994 -1.9292379212 1.8759625709
O -4.9474991578 -2.6573473801 1.4316162743
O -3.6703433230 -1.3406985011 3.2196382244
C -3.4100091665 -0.6211156218 0.7026187476
C -3.6678909737 -0.8297825999 -0.6527285048
C -2.8979742041 0.5954226089 1.1571945507
C -3.3861984122 0.1893196750 -1.5601330660
H -4.0940704313 -1.7701743365 -0.9866177170
C -2.6179827579 1.6006459775 0.2341942811
H -2.7341616213 0.7457377844 2.2188923056
C -2.8474966457 1.4132084343 -1.1368464047
H -2.2140249318 2.5478527716 0.5825330487
C -0.1506775624 -2.1361685919 1.8692744505
H -0.5157814445 -1.1269889360 1.6441038858
H 0.6389605253 -2.0144983274 2.6243653694
C 0.4392914662 -2.7881023922 0.6118060087
H -0.3655518421 -2.9748750410 -0.1086552919
H 0.8347930507 -3.7766553313 0.8875183450
C 1.5777856115 -1.9794912679 -0.0384154514
H 2.1187383470 -2.6295905737 -0.7423873924
H 2.2984266943 -1.7144001966 0.7446846573
C 1.1348362000 -0.7074672421 -0.7974691548
H 0.4481477712 -0.1286859300 -0.1706765631
C 0.4071457098 -1.0690663794 -2.1016928419
H 0.0283994628 -0.1772736764 -2.6056851685
H 1.0783079366 -1.6002320595 -2.7907802995
H -0.4479990508 -1.7222691278 -1.9027493492
H -3.5882488192 0.0325693362 -2.6167677884
C -2.4974321384 2.4977471434 -2.1255360572
H -2.8592310849 3.4760941773 -1.7886725998
H -1.4083036176 2.5701211870 -2.2352109532
H -2.9293671090 2.2972837258 -3.1111403007
H -0.9538899710 -3.9769366202 2.6766378323
H 3.0552834933 -0.3693156007 -1.7083676243
H -2.9179067481 -3.9529566417 1.6235301618
H -1.5554856715 -2.5253615638 3.4822106018

10-PBN

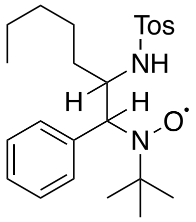

Name	E(B3LYP)	H(B3LYP)	g-factor	α_N	α_H
Tosyl_N_radical_hexane_C2_radical_PBN_adduct_0081	-1668.888193	-1668.296061	2.00609767	14.230040	5.863620
Tosyl_N_radical_hexane_C2_radical_PBN_adduct_0000	-1668.887582	-1668.295425	2.00610500	13.969900	5.031820
Tosyl_N_radical_hexane_C2_radical_PBN_adduct_0085	-1668.887251	-1668.295124	2.00611410	13.948440	5.016140
Tosyl_N_radical_hexane_C2_radical_PBN_adduct_0088	-1668.887212	-1668.295110	2.00611413	13.958350	5.015990
Tosyl_N_radical_hexane_C2_radical_PBN_adduct_0044	-1668.887193	-1668.295078	2.00611147	13.952530	5.055890
Tosyl_N_radical_hexane_C2_radical_PBN_adduct_0060	-1668.887144	-1668.294841	2.00607927	14.215620	6.397750
Tosyl_N_radical_hexane_C2_radical_PBN_adduct_0428	-1668.886843	-1668.294624	2.00609650	14.236780	5.896480
Tosyl_N_radical_hexane_C2_radical_PBN_adduct_0081	-1668.888193	-1668.296061	2.00609767	14.230040	5.863620
Boltzman averaged for 298.15 K			2.006105	14.0753	5.4608

```
65

C -1.8188498494 0.2724876304 0.2918278156
C -2.8253286825 -0.7672970134 2.3568297357
H -2.1588962344 -0.1374591499 2.9427346088
C -3.6961064689 -1.6450103271 3.0049457163
H -3.7013337647 -1.6963189396 4.0905925255
C -4.5644811929 -2.4441097030 2.2603250487
H -5.2481413151 -3.1234700027 2.7622944843
C -4.5538373586 -2.3609947094 0.8659809653
H -5.2295016382 -2.9776605543 0.2789930252
C -3.6787183492 -1.4890083598 0.2178124294
H -3.6694683882 -1.4276133252 -0.8660295003
C -2.8026899094 -0.6824247682 0.9582356090
N -2.3589049321 0.8875859606 -0.9347981149
C -3.1990618252 2.1372768256 -0.9775018275
C -4.6237347042 1.7296334865 -1.3993962168
C -2.5723298503 3.0632640169 -2.0368287046
C -3.2251888716 2.8394800531 0.3866046543
H -5.2429570150 2.6229659074 -1.5360706875
H -5.0940300621 1.0970492697 -0.6392271024
H -4.5919848291 1.1778924025 -2.3426813331
H -2.5343150973 2.5593164382 -3.0055449148
H -1.5544790987 3.3531711808 -1.7547834769
H -3.1756885146 3.9720381771 -2.1367775068
H -3.6618598948 2.2069476541 1.1666750354
H -3.8502163358 3.7346598139 0.3013291038
H -2.2272580459 3.1597380627 0.7026919671
O -2.2115213768 0.2526961106 -2.0464866680
C -0.4052360927 -0.3638737487 0.0234540226
N 0.4424869496 0.5385794623 -0.7765238036
S 1.1375706915 1.9073416780 -0.0936938829
O 1.2035437305 2.8910600916 -1.1821998244
O 0.4806420926 2.3342230607 1.1840438267
C 2.8057834434 1.3847351213 0.3045580004
C 3.1903615460 1.2612013672 1.6372728473
C 3.7103686056 1.1312376690 -0.7313225571
C 4.4968422614 0.8670479391 1.9335722120
H 2.4773788166 1.4794308523 2.4247022619
C 5.0066431177 0.7400093595 -0.4173430993
H 3.4016458257 1.2471842587 -1.7651888873
C 5.4215025394 0.6004384643 0.9182142284
H 5.7128441861 0.5421004151 -1.2202674115
C -0.3816005807 -1.7628964595 -0.6162398809
H -1.1069575028 -2.3930545972 -0.0896791451
H -0.7292614766 -1.6870248247 -1.6513344447
C 0.9997462014 -2.4279329729 -0.5594538816
H 1.3222456113 -2.5124375822 0.4896974712
H 1.7375812143 -1.7845759483 -1.0551445645
C 1.0146081989 -3.8194414797 -1.2053386887
H 0.6905588902 -3.7363778041 -2.2534114101
C 2.3873357350 -4.5025190369 -1.1540980911
H 2.7109296600 -4.5866680783 -0.1066977207
C 2.3968605373 -5.8898788963 -1.8041244467
H 1.6892461492 -6.5652035996 -1.3073554634
H 2.1114182445 -5.8338115216 -2.8618803079
H 3.3898343750 -6.3511520946 -1.7513703047
H 4.8001990934 0.7695915601 2.9729428573
C 6.8377274174 0.1862519699 1.2392374248
H 6.9707087503 0.0086930079 2.3106082002
H 7.1147855513 -0.7315836548 0.7073454378
H 7.5541456792 0.9609904278 0.9380589375
H 0.0605380955 -0.4413236533 1.0128182305
H -1.6257414433 1.0911389481 0.9837867117
H 0.0214418619 0.7810892331 -1.6731334308
H 3.1291825895 -3.8602961130 -1.6498752400
H 0.2719766101 -4.4624035271 -0.7094170490
65

C -1.8601369325 0.1635659203 0.3235969189
C -3.8268513690 -1.3479396133 -0.3148827831
H -3.5733993717 -1.2040822635 -1.3607683245
C -4.9043660007 -2.1588039959 0.0425201630
H -5.4913215751 -2.6441304626 -0.7328876519
C -5.2293215277 -2.3483552784 1.3878196540
H -6.0699058617 -2.9795344751 1.6633025517
C -4.4710305844 -1.7187772529 2.3755583182
H -4.7184150587 -1.8553861849 3.4249782618
C -3.3966199924 -0.9023095265 2.0179185991
H -2.8134161892 -0.4076026634 2.7919619552
C -3.0606578598 -0.7105014151 0.6713006391
N -2.0576372879 0.9517402752 -0.9091941540
C -2.8213834984 2.2486963258 -0.9881594026
C -4.1586755354 1.9702565010 -1.7016058953
C -1.9669389377 3.2219230458 -1.8209357473
C -3.0666643495 2.8350288174 0.4083378264
H -4.7981016996 1.3124752875 -1.1043000870
H -3.9752779890 1.4960537975 -2.6698236099
H -4.6939619841 2.9113929098 -1.8692921713
H -1.7582009418 2.7970026664 -2.8055749210
H -1.0199140678 3.4294770603 -1.3139103337
H -2.5076492907 4.1655336667 -1.9524712099
```

H -3.6203102183 3.7732993185 0.2961306353
H -2.1260236383 3.0628880092 0.9191216644
H -3.6712210038 2.1715865990 1.0355780977
O -1.6994653888 0.4241246801 -2.0288100921
C -0.5091619916 -0.6351234383 0.2834444517
N 0.6152976879 0.2167393136 -0.1551739495
S 1.1863877539 1.4100257045 0.8942270341
O 0.6218603377 2.7284725845 0.5528515435
O 1.0245194243 0.8737585808 2.2519615850
C 2.9285747670 1.4595807275 0.4829058405
C 3.4447168065 2.5629863335 -0.1922233952
C 3.7547475431 0.3995397314 0.8692049892
C 4.8074161038 2.5965835940 -0.4942909119
H 2.7880460319 3.3809699417 -0.4677509427
C 5.1090042893 0.4512430926 0.5604963805
H 3.3392121428 -0.4462598237 1.4069685371
C 5.6575236552 1.5480262982 -0.1261019996
H 5.7554912016 -0.3708311199 0.8585049575
C -0.4860551354 -1.9373135714 -0.5364394476
H -1.3816169132 -2.5152673500 -0.2823499773
H -0.5623084279 -1.6973835472 -1.6012303076
C 0.7581888021 -2.7935659872 -0.2661373659
H 0.8094528013 -3.0360656310 0.8063156824
H 1.6593525848 -2.2098132730 -0.4932215599
C 0.7715590273 -4.0943698269 -1.0794698401
H 0.7146341873 -3.8530077235 -2.1513617927
C 2.0101085986 -4.9625481591 -0.8229589450
H 2.0667125101 -5.2050628511 0.2479149323
C 2.0205556043 -6.2582310208 -1.6405878750
H 2.0000574105 -6.0476705837 -2.7170573313
H 2.9165736221 -6.8553110204 -1.4351943441
H 1.1463680014 -6.8795559224 -1.4097400997
H 5.2141582376 3.4554241044 -1.0221559951
C 7.1335592905 1.5911070977 -0.4418470444
H 7.7313822205 1.6750586205 0.4745472877
H 7.4581502315 0.6779536697 -0.9545186376
H 7.3825235369 2.4436719972 -1.0806990203
H -0.3050927617 -0.8917582214 1.3274903410
H -1.7379880927 0.8881231016 1.1314318745
H 0.4401543557 0.6098932987 -1.0812311004
H 2.9136751833 -4.3788300113 -1.0501185607
H -0.1328563957 -4.6786118979 -0.8525461397
65

C -1.8867838948 0.2065341410 0.1973539724
C -3.8360688225 -1.4336911990 -0.0801330696
H -3.8812896275 -1.1744263292 -1.1333064242
C -4.7191264583 -2.3737669431 0.4515658568
H -5.4554055434 -2.8440398089 -0.1950455859
C -4.6605877128 -2.7113159631 1.8057622238
H -5.3505915229 -3.4431216181 2.2171624995
C -3.7146951316 -2.0983767106 2.6283629958
H -3.6653623849 -2.3479866939 3.6850305580
C -2.8356388874 -1.1525175743 2.0979722762
H -2.1080438864 -0.6688083125 2.7466230683
C -2.8820944156 -0.8133279754 0.7391006577
N -2.4598256054 1.0578308139 -0.8621116042
C -3.2317759124 2.3299405909 -0.6260299704
C -4.6973638826 2.0683235877 -1.0229761557
C -2.6147540721 3.4055892025 -1.5396575172
C -3.1453392458 2.7719235610 0.8407701600
H -4.7471074030 1.6972472106 -2.0501281842
H -5.2738904158 2.9975593697 -0.9573221238
H -5.1589606655 1.3285843924 -0.3604802175
H -1.5688628365 3.5955009156 -1.2756972903
H -3.1728815338 4.3424459524 -1.4363323463
H -2.6567761944 3.0856361890 -2.5835006756
H -3.5735996105 2.0277269904 1.5205443640
H -3.7238130576 3.6944853002 0.9571493169
H -2.1155199630 2.9843722811 1.1451218157
O -2.4066903491 0.6328457265 -2.0775398034
C -0.5232168073 -0.4274788062 -0.2674470917
N 0.3171969250 0.5634968429 -0.9619940362
S 1.1304769269 1.7589120906 -0.1085246268
O 1.2122583559 2.9014200962 -1.0272602767
O 0.5578438309 1.9124738379 1.2404222957
C 2.7752178636 1.0769419985 0.1080464423
C 3.2184161477 0.7369358194 1.3839879248
C 3.6037060340 0.9162205146 -1.0070064669
C 4.5055037537 0.2175411988 1.5406452942
H 2.5652708795 0.8859688829 2.2367923611
C 4.8818964042 0.3982754119 -0.8321057465
H 3.2507985344 1.1989767459 -1.9934955514
C 5.3541893852 0.0399546754 0.4424231624
H 5.5289244732 0.2724733880 -1.6970093525
C -0.6167822276 -1.6985191673 -1.1324057405
H -1.2751812813 -2.4089401654 -0.6195811506
H -1.1084849575 -1.4405563432 -2.0747420135
C 0.7346439827 -2.3745951895 -1.4173146584
H 1.3984221112 -1.6631681975 -1.9243477078
H 0.5545280830 -3.1912023428 -2.1306377300
C 1.4504217047 -2.9477226972 -0.1859199288
H 0.7718185135 -3.6300850611 0.3484840955
C 2.7454354939 -3.6924944166 -0.5367188169
H 3.4124051151 -3.0123456641 -1.0849785005
C 3.4778149897 -4.2422643897 0.6914906643
H 3.7624623686 -3.4325357866 1.3748340352
H 2.8447875217 -4.9413950446 1.2519909165
H 4.3922387445 -4.7764264276 0.4080437257
H 4.8546082444 -0.0476627762 2.5354398550
C 6.7521030799 -0.5045139828 0.6146403032
H 6.9161891922 -0.8744259700 1.6311356489
H 6.9478236042 -1.3284648345 -0.0813124849
H 7.5042056171 0.2697656769 0.4165104449
H -0.0055239198 -0.6832140398 0.6625698653
H -1.6168636934 0.8767823642 1.0123365512
H -0.1427821208 0.9736540766 -1.7745702244
H 2.5144304415 -4.5164625521 -1.2269127347
H 1.6939159992 -2.1416197100 0.5202792597
65

C -1.6920731963 0.2099131304 0.2697653343
C -2.6355394551 -0.9131236746 2.3210870678
H -1.9971981651 -0.2619753206 2.9149075025
C -3.4589553959 -1.8434128757 2.9577473306
H -3.4554818464 -1.9138405018 4.0423241532
C -4.2916050674 -2.6708410788 2.2033645266
H -4.9384662397 -3.3914191020 2.6965386927
C -4.2929741151 -2.5629434702 0.8106699430
H -4.9413530222 -3.2012894025 0.2161199394
C -3.4650506320 -1.6380618143 0.1738574699
H -3.4652437708 -1.5571926779 -0.9087499027
C -2.6249272902 -0.8028479458 0.9241499766
N -2.2665465284 0.8151641377 -0.9459557316
C -3.1653099295 2.0239503941 -0.9688307888
C -2.5867456656 2.9938042909 -2.0162378593
C -3.2209149323 2.7044482031 0.4053227879
C -4.5701850569 1.5548809062 -1.3933373374
H -2.5275043511 2.5059809432 -2.9921188958
H -1.5831860285 3.3284637750 -1.7325571119
H -3.2333607907 3.8738349465 -2.1018585630
H -2.2385141119 3.0676581650 0.7235209238
H -3.6243768576 2.0404584239 1.1771163072
H -3.8885069276 3.5696616894 0.3344978755
H -5.0077236333 0.8901155569 -0.6411070213
H -4.5149993693 1.0185761010 -2.3444167746
H -5.2315963493 2.4195468514 -1.5159919405
O -2.0923554087 0.2036599064 -2.0668643006
C -0.2495739218 -0.3514436405 -0.0127042593
N 0.5480874630 0.6024449150 -0.8042811775
S 1.1781603272 1.9952178535 -0.1075869406
O 1.1985932039 2.9907851500 -1.1870608604
O 0.5064502914 2.2809204870 1.1723833557
C 2.8681287221 1.5454486379 0.2881283284
C 3.7776824611 1.3156887373 -0.7491725518
C 3.2646034648 1.4592678866 1.6201461208
C 5.0911524783 0.9856458854 -0.4369548470
H 3.4590334088 1.4024420362 -1.7829040439
C 4.5886269911 1.1267151327 1.9147548133
H 2.5468349119 1.6585597520 2.4082309294

C 5.5183883450 0.8838789329 0.8982895348
H 4.9014884348 1.0593740058 2.9536776194
C -0.1595011800 -1.7397100748 -0.6707609723
H -0.8629059661 -2.4025960092 -0.1578634783
H -0.5011693850 -1.6641836238 -1.7082908896
C 1.2548577655 -2.3343952452 -0.6132602600
H 1.5512499515 -2.4698692238 0.4384188797
H 1.9617422304 -1.6129131722 -1.0400011184
C 1.3995570121 -3.6737357489 -1.3545695233
H 2.4633590483 -3.9516728000 -1.3703383445
C 0.5985265420 -4.8378221174 -0.7539048266
H -0.4742292930 -4.6054219668 -0.7757619947
C 0.8397849741 -6.1648317734 -1.4812818299
H 1.8985170675 -6.4504661754 -1.4447792176
H 0.2592209771 -6.9795273184 -1.0333392505
H 0.5539470197 -6.0946569658 -2.5381627108
H 5.8013092570 0.8070600345 -1.2409301975
C 6.9506138996 0.5265945038 1.2166842255
H 7.2233090518 -0.4442121914 0.7850933349
H 7.6455612016 1.2680744185 0.8037095728
H 7.1203356384 0.4735324667 2.2961840643
H 0.2240270718 -0.4184021909 0.9737996435
H -1.5377000394 1.0266089811 0.9736690557
H 0.1108305347 0.8351304040 -1.6957477296
H 0.8649949483 -4.9481794937 0.3074441191
H 1.1069216617 -3.5382848189 -2.4065927091
65

C -1.7410333115 0.2106438703 0.2832860367
C -3.5446975079 -1.6097499983 0.2573819332
H -3.4840010695 -1.6297737192 -0.8263484180
C -4.4258441198 -2.4578129655 0.9287589657
H -5.0542076111 -3.1380761040 0.3597489616
C -4.5028435952 -2.4359588282 2.3233046856
H -5.1909932365 -3.0969938970 2.8432804290
C -3.6951307325 -1.5557081829 3.0443331859
H -3.7524370739 -1.5251811807 4.1292606953
C -2.8186040978 -0.7019946968 2.3723722527
H -2.1999158114 -0.0088671492 2.9388537439
C -2.7293363147 -0.7221844980 0.9739980516
N -2.2357535104 0.7112674565 -1.0125609682
C -3.1068902917 1.9268546813 -1.1942338026
C -3.2216993249 2.7311979323 0.1074574603
C -4.4964598693 1.4431068905 -1.6513448062
C -2.4529950062 2.7875362373 -2.2915792664
H -2.2504177252 3.1066270759 0.4451172686
H -3.6794780212 2.1472814190 0.9129299340
H -3.8664054420 3.5967398366 -0.0782264194
H -4.9870222255 0.8562510512 -0.8676566154
H -4.4016565777 0.8218464241 -2.5459163114
H -5.1324914293 2.3032914797 -1.8873646454
H -1.4592985975 3.1306285278 -1.9842183282
H -3.0755681597 3.6662333712 -2.4920707628
H -2.3517170070 2.2116647202 -3.2145938077
O -2.0150218791 -0.0027705724 -2.0624319596
C -0.2975725232 -0.3973991823 0.1347756790
N 0.5623634873 0.4674368791 -0.6932252413
S 1.1847456373 1.9061195567 -0.0897260768
O 1.2794385178 2.8024184569 -1.2492719885
O 0.4555533016 2.3128310919 1.1242617392
C 2.8439281383 1.4679341705 0.4297443684
C 3.8042477114 1.1531175626 -0.5369070110
C 3.1662389393 1.4720244581 1.7844421650
C 5.0934122204 0.8299855034 -0.1301993941
H 3.5433463943 1.1697449148 -1.5901391638
C 4.4666472982 1.1446789143 2.1743658541
H 2.4103223809 1.7361578614 2.5157630329
C 5.4461967656 0.8189956235 1.2303039513
H 4.7215558941 1.1472617376 3.2311605904
C -0.2014809447 -1.8407023318 -0.3895032450
H -0.9262124038 -2.4531364349 0.1591248560
H -0.5117860353 -1.8537218671 -1.4377176470
C 1.1989237588 -2.4457313018 -0.2159856239
H 1.4819228664 -2.3857022743 0.8453393448
H 1.9321033331 -1.8377891374 -0.7610325129
C 1.3064639651 -3.9109289528 -0.6709619472
H 0.5619718694 -4.5172287632 -0.1330365254
C 1.1404349483 -4.1302855604 -2.1816393007
H 1.8661868646 -3.5016188414 -2.7175947800
C 1.3286347446 -5.5937177973 -2.5952287256
H 0.5967055566 -6.2429915763 -2.0987746491
H 1.2080537502 -5.7234589557 -3.6768781577
H 2.3279709592 -5.9572825458 -2.3249389042
H 5.8429048015 0.5851473492 -0.8791414297
C 6.8546319896 0.4737728183 1.6515629445
H 7.5721317714 1.2160254630 1.2799414116
H 6.9493652292 0.4346845062 2.7408254968
H 7.1628513764 -0.4993160249 1.2508054110
H 0.1200122325 -0.3814614398 1.1483202123
H -1.6075196998 1.0861143831 0.9173122204
H 0.1813031418 0.6246255723 -1.6259579162
H 0.1476986949 -3.7898240368 -2.5032199199
H 2.2889445051 -4.3000586605 -0.3668944700
65

C -1.8339878328 0.2420391368 0.2524782132
C -3.7146988376 -1.4873327040 0.0557298168
H -3.7088302867 -1.3450162856 -1.0205038268
C -4.5974469827 -2.3955333609 0.6407615882
H -5.2823393827 -2.9588545707 0.0123132832
C -4.6039156025 -2.5827151115 2.0250123943
H -5.2934804731 -3.2900505707 2.4778574535
C -3.7236834489 -1.8513362454 2.8233733850
H -3.7255227405 -1.9836933669 3.9021541158
C -2.8451979789 -0.9373974755 2.2388228220
H -2.1693616315 -0.3607890289 2.8672165069
C -2.8266304571 -0.7484091153 0.8504111488
N -2.3714134214 0.9524729768 -0.9226751373
C -3.1955010534 2.2124451692 -0.8692335529
C -3.2106682862 2.8103503895 0.5438064018
C -4.6259562836 1.8555226482 -1.3168144688
C -2.5588680809 3.2074046088 -1.8577509014
H -2.2084412014 3.0943406879 0.8802285827
H -3.6533746633 2.1260201129 1.2752497828
H -3.8252310693 3.7166354859 0.5277761037
H -4.6027508358 1.3751199504 -2.2985859350
H -5.2338233037 2.7643602516 -1.3851808728
H -5.1032673057 1.1742648683 -0.6045719127
H -1.5368989073 3.4626738208 -1.5576870628
H -3.1508396991 4.1285901162 -1.8875822802
H -2.5290108570 2.7771241118 -2.8616400566
O -2.2348429935 0.4004195271 -2.0790685715
C -0.4290604999 -0.3892258103 -0.0687439999
N 0.4255491444 0.5582044286 -0.8068888479
S 1.1407085279 1.8660225175 -0.0322115382
O 1.2197649890 2.9217209318 -1.0500636513
O 0.4901898806 2.1134982168 1.2665040117
C 2.8013615560 1.2923289048 0.3255573517
C 3.6990230491 1.0915736670 -0.7278588656
C 3.1878246554 1.0783238735 1.6461481364
C 4.9898559419 0.6611947577 -0.4444790580
H 3.3891676407 1.2781665925 -1.7509991400
C 4.4889066533 0.6459230412 1.9117220341
H 2.4808275724 1.2574391826 2.4487058546
C 5.4061908086 0.4298670327 0.8779074787
H 4.7938606232 0.4783604596 2.9416368807
C -0.4245665984 -1.7394279388 -0.8062774160
H -1.1500626530 -2.3991878912 -0.3173635122
H -0.7822661045 -1.5878991424 -1.8296193466
C 0.9515449173 -2.4182081681 -0.8113860436
H 1.2848336254 -2.5632125507 0.2261404166
H 1.6874786562 -1.7524673120 -1.2802972266
C 0.9371248069 -3.7649387043 -1.5487157853
H 0.5507199502 -3.6048544934 -2.5653181407
C 2.3072350303 -4.4565057559 -1.6457476956
H 3.0176756037 -3.7803257658 -2.1427607168
C 2.8891076073 -4.9073344102 -0.3004221442
H 2.2000768556 -5.5850373588 0.2195438157
H 3.8365847216 -5.4407581317 -0.4399245478
H 3.0847249673 -4.0592495366 0.3652804807

H 5.6907453992 0.5045930026 -1.2610745787
C 6.8153896791 -0.0302169331 1.1656630511
H 6.9626001850 -0.2295425701 2.2312938899
H 7.0548625391 -0.9475777616 0.6145904444
H 7.5488844488 0.7273045507 0.8624960041
H 0.0402604104 -0.5423330519 0.9100831402
H -1.6285836417 1.0048933932 1.0022886868
H 0.0038017212 0.8684604875 -1.6820416636
H 2.2117504190 -5.3319685391 -2.3022765658
H 0.2240531727 -4.4438740345 -1.0570457844
65

C -1.8682751031 0.0623803443 0.2340165725
C -3.9478654264 -1.1070372334 -0.6957052957
H -3.7184585941 -0.6953415710 -1.6737958526
C -5.0638308155 -1.9259038750 -0.5220084029
H -5.7048327010 -2.1470833786 -1.3714259406
C -5.3581531612 -2.4606071076 0.7345151363
H -6.2287541656 -3.0972167109 0.8672593411
C -4.5302826397 -2.1686170197 1.8190968449
H -4.7530061733 -2.5751425407 2.8020731205
C -3.4170829451 -1.3439958312 1.6467038411
H -2.7796877652 -1.1131995548 2.4978599135
C -3.1114525041 -0.8077480420 0.3889993780
N -2.0580210455 1.1681511684 -0.7265442596
C -2.7457641109 2.4719655138 -0.4120357217
C -1.8425313074 3.5967388324 -0.9506460858
C -2.9480271354 2.6445906277 1.0990843233
C -4.1014457281 2.4812487152 -1.1446291388
H -0.8822897272 3.5995042114 -0.4261866608
H -2.3288444829 4.5659061501 -0.7948209704
H -1.6644718277 3.4615589222 -2.0201402602
H -3.5808073028 1.8584561301 1.5239275040
H -3.4531623002 3.6008402187 1.2722068940
H -1.9925086270 2.6710898456 1.6316828499
H -3.9523784920 2.2940267025 -2.2116597645
H -4.5823198965 3.4583622023 -1.0248939595
H -4.7738142552 1.7156434364 -0.7444568516
O -1.7479479118 0.9629435083 -1.9603028966
C -0.5745819603 -0.7677046855 -0.0892553976
N 0.5837869701 0.1069979680 -0.3651923869
S 1.2918841417 0.9209642054 0.9332453301
O 0.6764364829 2.2434506023 1.1552401823
O 1.3386474474 -0.0462147160 2.0380667416
C 2.9443990967 1.2046205606 0.3051772890
C 3.3636118079 2.5065726212 0.0425104347
C 3.8072930482 0.1189806849 0.1236940657
C 4.6629109224 2.7199850590 -0.4219135665
H 2.6823639874 3.3342775870 0.2065762247
C 5.0971577200 0.3511176687 -0.3387774445
H 3.4697777922 -0.8876718870 0.3468767793
C 5.5456954747 1.6529699979 -0.6210591935
H 5.7718522067 -0.4896893836 -0.4814929690
C -0.6840560216 -1.8136270540 -1.2145887414
H -1.5587460381 -2.4402476245 -1.0060877990
H -0.8835137056 -1.2995344453 -2.1588436819
C 0.5546268421 -2.7130887152 -1.3614914425
H 1.4288581927 -2.0930673648 -1.5969545747
H 0.3921628803 -3.3612603228 -2.2343478729
C 0.8675157532 -3.5944846104 -0.1435475423
H -0.0314193000 -4.1654080164 0.1354360145
C 2.0262556122 -4.5703506967 -0.3894543140
H 2.9226214006 -4.0019506391 -0.6777628629
C 2.3472263371 -5.4471237058 0.8252761275
H 1.4805933541 -6.0542068264 1.1149295542
H 3.1782530078 -6.1313823081 0.6182446740
H 2.6256810631 -4.8361297618 1.6927154095
H 4.9939431725 3.7345198182 -0.6286546237
C 6.9501350455 1.8837848387 -1.1253769939
H 7.1146883673 1.3787101097 -2.0852084683
H 7.1553295626 2.9488697451 -1.2679670746
H 7.6939811618 1.4886175611 -0.4229654569
H -0.3419416860 -1.2912857587 0.8415237357
H -1.6746068470 0.5336467992 1.1998662459
H 0.3806298383 0.7525621144 -1.1306519165
H 1.7850147198 -5.2101215801 -1.2504237967
H 1.1131954097 -2.9667590595 0.7244214672
65

C -1.9092453759 0.1923242421 0.3093268489
C -3.9144328741 -1.3350262421 -0.1464103856
H -3.6851286812 -1.2885021435 -1.2066082777
C -4.9951420657 -2.0917890209 0.3070719014
H -5.6089428609 -2.6332437708 -0.4080507031
C -5.2890442630 -2.1559750192 1.6712038982
H -6.1321289242 -2.7453977875 2.0214332821
C -4.4965261998 -1.4550408743 2.5809424320
H -4.7196209163 -1.4939225220 3.6438959608
C -3.4189625704 -0.6924548882 2.1268729975
H -2.8088040267 -0.1416140651 2.8398231271
C -3.1139707546 -0.6265753566 0.7609421734
N -2.1235298900 0.8704861059 -0.9843843727
C -2.8581477190 2.1744524677 -1.1614449384
C -2.0098280283 3.0412262672 -2.1099939322
C -3.0399686428 2.8961357420 0.1802831128
C -4.2260028146 1.8667315170 -1.8010692243
H -2.5307546089 3.9836707519 -2.3111843715
H -1.8474425372 2.5210136804 -3.0568685270
H -1.0400202435 3.2678342813 -1.6569222052
H -2.0768375996 3.1448915008 0.6367797713
H -3.6386484593 2.3120932832 0.8870987980
H -3.5733734399 3.8350281113 -0.0024138384
H -4.7444809455 2.8020588783 -2.0392726352
H -4.8594264796 1.2849970202 -1.1237814942
H -4.0879900445 1.2992806976 -2.7256905135
O -1.8130906191 0.2325104145 -2.0599319631
C -0.5710188992 -0.6281950478 0.3107390159
N 0.5521772945 0.1645218658 -0.2285512742
S 1.1799947428 1.4316814662 0.6927411042
O 0.7002020576 2.7285023690 0.1831253957
O 0.9666628572 1.0659899806 2.0993148249
C 2.9307403475 1.3365480490 0.3270442647
C 3.6953608391 0.3154714065 0.8996359226
C 3.5131313645 2.2878472765 -0.5070564566
C 5.0550122571 0.2497724450 0.6174776615
H 3.2291926209 -0.4075105485 1.5608870453
C 4.8798673275 2.2049815473 -0.7795290831
H 2.9046508913 3.0818880599 -0.9260873968
C 5.6693500074 1.1907551703 -0.2262819771
H 5.3381528020 2.9451422428 -1.4305720437
C -0.5841077855 -1.9990811490 -0.3883315588
H -1.4799206966 -2.5395019957 -0.0623885976
H -0.6836755565 -1.8547816179 -1.4683835970
C 0.6577747377 -2.8429840556 -0.0709479151
H 0.7259014831 -2.9984967413 1.0170286061
H 1.5545560742 -2.2831802262 -0.3601662846
C 0.6351779008 -4.2083134297 -0.7726237028
H 0.5873664447 -4.0553349260 -1.8612388890
C 1.8322033978 -5.1158356518 -0.4428476847
H 1.6510633362 -6.1074835195 -0.8792051845
C 3.1833227821 -4.5929915767 -0.9458506463
H 3.1657437929 -4.4385460772 -2.0321705462
H 3.4513476452 -3.6374332496 -0.4813871799
H 3.9878893143 -5.3039286502 -0.7244791720
H 5.6539238277 -0.5420764240 1.0611667380
C 7.1504436586 1.1112042831 -0.5096335951
H 7.4481969379 1.8179715688 -1.2899260725
H 7.7373517126 1.3403514073 0.3889479295
H 7.4405979411 0.1053720336 -0.8350668958
H -0.3459394725 -0.7922998945 1.3689064800
H -1.7560208555 0.9832076446 1.0463742692
H 0.3640074770 0.4755273410 -1.1824195197
H 1.8809776333 -5.2678592814 0.6451433081
H -0.2913447960 -4.7335014817 -0.4995819096
65

C -1.7373199125 0.1077940475 0.2313435302
C -3.6025972426 -1.5196192206 -0.4264152971
H -3.3073524599 -1.4083323215 -1.4652819604
C -4.6579900409 -2.3644090495 -0.0817309023

H -5.1857684351 -2.9091910736 -0.8601871295
C -5.0365620709 -2.5116878636 1.2548126550
H -5.8597302718 -3.1695147998 1.5203447208
C -4.3544666060 -1.8056014913 2.2463556005
H -4.6442421894 -1.9089283645 3.2887024313
C -3.3023770102 -0.9554245486 1.9010750203
H -2.7788447797 -0.4014415107 2.6776102056
C -2.9127465220 -0.8055449387 0.5636347165
N -1.9095466774 0.8283445438 -1.0457649813
C -2.7225838540 2.0851505156 -1.2221812418
C -3.0602489926 2.7230903799 0.1317692257
C -4.0105798175 1.7153576590 -1.9831527387
C -1.8702685413 3.0561341941 -2.0597444456
H -3.6479269965 3.6291039447 -0.0506258153
H -2.1562699491 3.0166236882 0.6742738076
H -3.6652363662 2.0620854541 0.7611886799
H -4.6504916062 1.0584727552 -1.3853609684
H -3.7603560680 1.2054094081 -2.9176444229
H -4.5758162493 2.6232603548 -2.2210150372
H -2.4423970222 3.9686434161 -2.2598974576
H -1.5975277492 2.5969218850 -3.0128403466
H -0.9578252267 3.3270403544 -1.5202218798
O -1.4758277077 0.2661655651 -2.1209711665
C -0.3512527709 -0.6271644475 0.2926098716
N 0.7532230090 0.2574802087 -0.1312382839
S 1.2191412666 1.5199081243 0.8888289222
O 0.6175087864 2.7951996257 0.4585761169
O 1.0105083259 1.0375234872 2.2603296776
C 2.9766376794 1.6291499907 0.5631055167
C 3.8254998150 0.6243713269 1.0376249339
C 3.4802160186 2.7242242233 -0.1347872753
C 5.1905762839 0.7224774032 0.7945637651
H 3.4185623523 -0.2150299065 1.5917719207
C 4.8540406647 2.8049928954 -0.3698405593
H 2.8049041141 3.4996236283 -0.4796626451
C 5.7273252639 1.8119313348 0.0874471888
H 5.2510642517 3.6573087085 -0.9154012337
C -0.2302598180 -1.9629816796 -0.4624907376
H -1.1175487375 -2.5595900297 -0.2295710918
H -0.2544379841 -1.7743799138 -1.5403244754
C 1.0357409867 -2.7451790270 -0.0837885378
H 1.0171300699 -2.9707039282 0.9936079209
H 1.9101024530 -2.1042665607 -0.2493091417
C 1.2195773625 -4.0537256744 -0.8703961396
H 2.2107073418 -4.4675901187 -0.6345460010
C 0.1614907865 -5.1311724691 -0.5926971640
H -0.8337089884 -4.7602048075 -0.8706433707
C 0.4351408546 -6.4414705871 -1.3387160837
H 0.4494413919 -6.2836112681 -2.4243390727
H 1.4069652440 -6.8641623345 -1.0546019877
H -0.3316438199 -7.1942515219 -1.1223536930
H 5.8546862536 -0.0568082477 1.1609051808
C 7.2148247472 1.9082850284 -0.1539429219
H 7.4576936291 2.7232593216 -0.8423294006
H 7.7557592566 2.0916305510 0.7831998501
H 7.6112327938 0.9779120294 -0.5770226734
H -0.1877683925 -0.8268585779 1.3561121915
H -1.6877459335 0.8734706230 1.0083463062
H 0.6061077868 0.6020838271 -1.0811345642
H 0.1226752673 -5.3267153577 0.4888426524
H 1.2342779259 -3.8298825825 -1.9476543948
65

C -1.8099898357 0.1354943330 0.2882850814
C -3.7168372209 -1.4881517683 -0.2481326295
H -3.3845102794 -1.5072904786 -1.2815591140
C -4.8156800785 -2.2491782414 0.1513827691
H -5.3397141333 -2.8600532914 -0.5790589779
C -5.2422015310 -2.2289203438 1.4814255829
H -6.0991268259 -2.8218332493 1.7896196105
C -4.5642562963 -1.4392766713 2.4108593678
H -4.8909188572 -1.4122231200 3.4470482241
C -3.4685540297 -0.6727384196 2.0099226463
H -2.9483522011 -0.0527966067 2.7372831859
C -3.0308757738 -0.6909341815 0.6790919981
N -1.9149852754 0.7149259899 -1.0658713635
C -2.6759523449 1.9702808128 -1.4077721722
C -3.0335445856 2.7646348929 -0.1447554944
C -3.9513445619 1.5605697134 -2.1695539717
C -1.7627625811 2.8145857205 -2.3155504864
H -2.1372524486 3.0860238012 0.3946023727
H -3.6824557677 2.1974610346 0.5307487724
H -3.5813458853 3.6637984819 -0.4462791514
H -4.4751463398 2.4545011835 -2.5257444373
H -4.6336784837 0.9953218358 -1.5267389360
H -3.6899111753 0.9422815958 -3.0328630847
H -1.4757154751 2.2439769921 -3.2019251285
H -0.8593305841 3.1145782090 -1.7763293526
H -2.2952532463 3.7169435621 -2.6353524767
O -1.4681337421 0.0236392994 -2.0572146270
C -0.4574937731 -0.6389022569 0.4782595235
N 0.6951782394 0.1489353741 -0.0052492969
S 1.1785309354 1.5018216579 0.8821215425
O 0.6095492286 2.7403785102 0.3194349213
O 0.9465913854 1.1689713626 2.2936844108
C 2.9397418907 1.5362512076 0.5602811309
C 3.7565838393 0.5401472540 1.1047274288
C 3.4793966522 2.5725854531 -0.1974679138
C 5.1257582476 0.5864494067 0.8706443874
H 3.3220309412 -0.2521765619 1.7052367670
C 4.8571439438 2.6019731341 -0.4223375418
H 2.8285102400 3.3434740196 -0.5952774796
C 5.6983369083 1.6148156627 0.1023483940
H 5.2825259702 3.4093830443 -1.0129207682
C -0.3699591456 -2.0546270059 -0.1190281577
H -1.2738800410 -2.6018471023 0.1724782755
H -0.3854125099 -1.9837711460 -1.2094514573
C 0.8616664844 -2.8307530636 0.3699507987
H 0.8604735293 -2.8412581990 1.4698065925
H 1.7728930614 -2.2978213126 0.0695274746
C 0.9257667542 -4.2817885951 -0.1357943401
H 0.0064206230 -4.8118685584 0.1557643640
C 1.1349605586 -4.4281266492 -1.6499715193
H 2.0377847482 -3.8728803707 -1.9430320096
C 1.2666283132 -5.8885900417 -2.0947202055
H 1.4176111816 -5.9661388844 -3.1775919170
H 2.1168862524 -6.3780659474 -1.6034176059
H 0.3660296336 -6.4622591935 -1.8426082389
H 5.7652299116 -0.1852059052 1.2927789015
C 7.1875127173 1.6437240591 -0.1460362630
H 7.4847138171 2.5397160900 -0.6989973752
H 7.7480992802 1.6273950345 0.7962873393
H 7.5092880945 0.7699531718 -0.7264056117
H -0.3323709434 -0.7229986096 1.5622223195
H -1.7531873288 0.9813323740 0.9766732599
H 0.5893997410 0.3882042067 -0.9924592058
H 0.3018765925 -3.9604925038 -2.1905107865
H 1.7477245837 -4.7968411092 0.3822136489

11'-PBN

A model for 11-PBN, with full alkyl chain substituted for propyl.

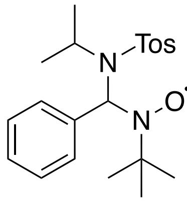

Name	E(B3LYP)	H(B3LYP)	g-factor	α_N	$\alpha_{N'}$	α_H
tos_Nipro_Nrad_PBN.conf.032	-1550.93394785	-1550.433013	2.00617	14.01711	1.37118	3.49630
tos_Nipro_Nrad_PBN.conf.112	-1550.93392226	-1550.432992	2.00617	14.01892	1.37269	3.48635
tos_Nipro_Nrad_PBN.conf.002	-1550.93376450	-1550.432118	2.00617	13.95405	1.38649	2.64644
tos_Nipro_Nrad_PBN.conf.013	-1550.93354099	-1550.432104	2.00617	12.98385	2.24697	0.82626
tos_Nipro_Nrad_PBN.conf.031	-1550.93395939	-1550.432080	2.00617	14.01616	1.37229	3.49476
tos_Nipro_Nrad_PBN.conf.006	-1550.93143315	-1550.430586	2.00611	13.79899	1.58581	3.34458
tos_Nipro_Nrad_PBN.conf.041	-1550.93166837	-1550.430303	2.00619	12.85659	2.26341	0.76687
tos_Nipro_Nrad_PBN.conf.014	-1550.93147242	-1550.429678	2.00611	13.80205	1.58347	3.34396
Boltzman averaged for 298.15 K			2.00617	13.7470	1.5912	2.6491

```
56
-1550.433013
S 0.918324 1.320613 -0.999788
N -0.284433 0.888138 0.096814
C 2.471577 0.631005 -0.402494
C 2.522412 -0.527328 0.370384
H 1.611878 -0.995980 0.725164
C 3.766710 -1.051579 0.725429
H 3.806611 -1.948781 1.338164
C 4.958987 -0.442141 0.317694
C 6.302385 -1.010912 0.708394
C 4.876750 0.725899 -0.455908
H 5.789770 1.222220 -0.776694
C 3.646433 1.267225 -0.815783
H 3.591577 2.179333 -1.400423
O 0.595004 0.639926 -2.265442
O 1.081144 2.778496 -0.984612
C -0.514699 1.679855 1.351504
C 0.744449 1.822864 2.212893
H 1.166041 0.845663 2.459551
C -1.183783 3.037065 1.090154
H 0.471827 2.324400 3.148289
H 1.510015 2.432106 1.723082
H 6.858630 -0.313425 1.347082
H 6.924476 -1.203922 -0.173897
H 6.195302 -1.951823 1.256464
H -0.507122 3.715481 0.566534
H -1.463371 3.491227 2.048564
H -2.091950 2.921619 0.492667
H -1.206179 1.049909 1.912047
C -1.216954 -0.165968 -0.312584
C -2.685536 0.206642 -0.111586
C -3.327335 0.922980 -1.131881
H -2.768088 1.201794 -2.022140
C -4.667852 1.286569 -1.008423
H -5.152118 1.840922 -1.807778
C -5.385270 0.934414 0.137217
H -6.430564 1.215007 0.234243
C -4.754586 0.215804 1.153611
H -5.307262 -0.065001 2.046207
C -3.412284 -0.149968 1.030808
H -2.926944 -0.716385 1.819225
N -0.875342 -1.464527 0.328614
C -1.072652 -2.809626 -0.322990
C -1.307232 -2.663929 -1.832842
C 0.208351 -3.623855 -0.066159
C -2.277266 -3.498645 0.346156
O -0.611967 -1.455691 1.586764
H -1.059882 -0.303382 -1.377474
H -1.398210 -3.664690 -2.267933
H -0.473515 -2.158258 -2.331210
H -2.234236 -2.125373 -2.056353
H 1.068802 -3.167794 -0.567370
H 0.085347 -4.641295 -0.452326
H 0.414483 -3.677141 1.005568
H -2.390826 -4.515195 -0.046139
H -3.204440 -2.950021 0.152975
H -2.123887 -3.556379 1.427261
56
-1550.432992
S -0.920588 1.311525 1.009315
N 0.281268 0.885563 -0.091217
C -2.474955 0.629456 0.406235
C -3.646449 1.277990 0.799960
H -3.591100 2.197316 1.373047
C -4.879691 0.739146 0.435127
H -5.791861 1.245684 0.740552
C -4.963357 -0.435960 -0.321764
C -6.299370 -1.015950 -0.721394
C -3.769274 -1.060040 -0.712538
H -3.809665 -1.964480 -1.315264
C -2.527369 -0.540604 -0.353668
H -1.616391 -1.017888 -0.695490
O -0.597532 0.619086 2.268616
O -1.080843 2.769758 1.006674
C 0.504369 1.679554 -1.345579
C -0.757873 1.817109 -2.203306
H -1.176320 0.837971 -2.447733
C 1.167735 3.039711 -1.085024
H -0.490060 2.318994 -3.139910
H -1.524480 2.423758 -1.711852
H -6.416612 -2.039747 -0.345613
H -6.403226 -1.060590 -1.812532
H -7.128732 -0.418788 -0.330662
H 1.442080 3.496160 -2.043865
H 2.078322 2.928007 -0.490508
H 0.489607 3.714410 -0.558503
H 1.197260 1.053530 -1.908753
C 1.219316 -0.165227 0.314020
C 2.685643 0.212803 0.106738
C 3.329752 0.930360 1.124712
H 2.773835 1.206342 2.017934
```

C 4.668411 1.298726 0.995387
H 5.154463 1.853969 1.793037
C 5.381719 0.950192 -0.153916
H 6.425572 1.234511 -0.255503
C 4.748801 0.230388 -1.168065
H 5.298272 -0.047648 -2.063502
C 3.408354 -0.140135 -1.039375
H 2.921329 -0.707484 -1.826082
N 0.879615 -1.464859 -0.326100
C 1.085862 -2.809383 0.323999
C -0.192476 -3.629378 0.072218
C 2.290685 -3.492715 -0.350552
C 1.325987 -2.663393 1.832961
O 0.611041 -1.456648 -1.583166
H 1.067188 -0.303677 1.379468
H -0.403391 -3.682442 -0.998594
H -1.052639 -3.177971 0.578145
H -0.062908 -4.646653 0.456668
H 2.132950 -3.550732 -1.431019
H 2.410527 -4.508898 0.040839
H 3.216201 -2.940013 -0.161130
H 2.251688 -2.121146 2.052931
H 1.422866 -3.663962 2.267227
H 0.492209 -2.161316 2.334820
56
-1550.432118
S -0.596457 -1.844523 -0.685039
N 0.529879 -1.068854 0.292809
C -2.151685 -0.958428 -0.470128
C -2.796070 -0.956564 0.770346
H -2.347123 -1.454156 1.622489
C -4.019828 -0.308374 0.900753
H -4.518402 -0.303822 1.867122
C -4.624249 0.335823 -0.191696
C -5.964596 1.013789 -0.036674
C -3.961973 0.312460 -1.424501
H -4.414555 0.798967 -2.285052
C -2.732863 -0.333485 -1.573185
H -2.229377 -0.365831 -2.533158
O -0.211823 -1.635372 -2.090846
O -0.767396 -3.197561 -0.150739
C 1.190446 -1.744024 1.458924
C 0.215861 -2.065349 2.595991
H 0.781793 -2.422941 3.464168
C 2.049220 -2.948644 1.050992
H -0.491745 -2.848811 2.311321
H -0.323599 -1.161515 2.892585
H -5.990941 1.647924 0.856787
H -6.769529 0.275071 0.068918
H -6.201002 1.638540 -0.903330
H 1.432397 -3.779820 0.703137
H 2.630574 -3.286721 1.917316
H 2.751517 -2.675282 0.257632
H 1.854153 -0.962884 1.830739
C 0.995014 0.240042 -0.174108
C 2.514186 0.303399 -0.355899
C 3.055950 -0.272413 -1.514790
H 2.393033 -0.755028 -2.228824
C 4.431142 -0.242383 -1.741247
H 4.837981 -0.690110 -2.644042
C 5.282043 0.362522 -0.812464
H 6.353970 0.387299 -0.989444
C 4.748454 0.934261 0.342413
H 5.403620 1.404074 1.071238
C 3.370175 0.908006 0.571367
H 2.958944 1.351290 1.472097
N 0.471282 1.330336 0.688111
C 0.046653 2.690060 0.195242
C -0.075800 2.720870 -1.333837
C -1.317187 2.988473 0.843931
C 1.092251 3.720488 0.663048
O 0.609839 1.196356 1.957768
H 0.555248 0.386839 -1.157665
H -0.830784 2.017438 -1.700174
H 0.878188 2.514603 -1.830797
H -0.390656 3.725193 -1.636110
H -1.637074 4.005126 0.590213
H -1.241238 2.903316 1.930514
H -2.079915 2.286185 0.492337
H 1.216731 3.662406 1.747422
H 0.762289 4.731827 0.401490
H 2.062862 3.542259 0.188924
56
-1550.432104
S 0.700453 -1.752106 0.617135
N -0.350997 -0.491624 0.986933
C 2.186538 -0.963277 -0.033605
C 3.092359 -0.360363 0.844714
H 2.905197 -0.370828 1.912741
C 4.238112 0.241844 0.334885
H 4.942165 0.709809 1.018934
C 4.506000 0.249364 -1.043993
C 5.766811 0.885837 -1.578090
C 3.589736 -0.371551 -1.900583
H 3.782100 -0.386756 -2.970518
C 2.436217 -0.982650 -1.405689
H 1.744463 -1.486279 -2.071903
O 1.064798 -2.403037 1.876927
O 0.136088 -2.522944 -0.501717
C -0.787052 -0.193856 2.392607
C -1.543923 -1.346746 3.064314
H -1.984349 -0.984237 4.001223
C 0.357672 0.341703 3.260691
H -0.880424 -2.182561 3.294902
H -2.355333 -1.691246 2.419767
H 5.707758 1.048437 -2.658588
H 6.640214 0.248873 -1.387048
H 5.961883 1.851607 -1.098617
H -0.049333 0.699605 4.213472
H 0.862678 1.181086 2.771361
H 1.088628 -0.440559 3.483884
H -1.500697 0.618356 2.243259
C -1.019581 0.151614 -0.155531
C -1.018421 1.680240 -0.026383
C 0.207263 2.347867 -0.170155
H 1.114441 1.774620 -0.342007
C 0.273555 3.737834 -0.095039
H 1.230947 4.238603 -0.213001
C -0.886925 4.483785 0.125636
H -0.837384 5.567839 0.183416
C -2.108265 3.827155 0.268579
H -3.016939 4.397573 0.441899
C -2.177605 2.433326 0.191441
H -3.129465 1.929702 0.312293
N -2.372333 -0.389488 -0.406501
C -2.879567 -0.959896 -1.705893
C -4.128780 -0.149480 -2.098997
C -1.825546 -0.866468 -2.816443
C -3.249206 -2.431206 -1.441897
O -3.206537 -0.346098 0.568841
H -0.429186 -0.093833 -1.035483
H -4.583126 -0.579011 -2.998297
H -3.868780 0.894260 -2.309377
H -4.862682 -0.169123 -1.290160
H -2.267909 -1.243684 -3.744486
H -0.948826 -1.481322 -2.594718
H -1.513648 0.168193 -3.001773
H -3.690804 -2.874104 -2.341793
H -3.976558 -2.492903 -0.628109
H -2.360127 -3.004460 -1.164268
56
-1550.432080
S 0.919028 1.317688 -1.002592
N -0.284071 0.887594 0.094691
C 2.472684 0.631914 -0.401862
C 2.524263 -0.530111 0.367054
H 1.613641 -1.001302 0.718230
C 3.767829 -1.049992 0.727207
H 3.807968 -1.947128 1.340231
C 4.960645 -0.434627 0.326290

C 6.301636 -1.022926 0.695275
C 4.877649 0.737983 -0.438390
H 5.790183 1.242755 -0.746588
C 3.646247 1.275654 -0.803800
H 3.590822 2.192842 -1.380338
O 0.597381 0.631517 -2.265694
O 1.079908 2.775827 -0.992937
C -0.513109 1.680734 1.348672
C 0.746439 1.822946 2.209592
H 1.167248 0.845440 2.456385
C -1.180676 3.038493 1.086344
H 0.474590 2.325034 3.144920
H 1.512449 2.431364 1.719432
H 6.695132 -1.646438 -0.118534
H 6.231162 -1.654966 1.586219
H 7.042931 -0.240600 0.890359
H -0.503481 3.715654 0.561792
H -1.459308 3.493875 2.044456
H -2.089234 2.923570 0.489347
H -1.205204 1.052157 1.909992
C -1.217646 -0.166143 -0.313299
C -2.685785 0.207572 -0.111048
C -3.411499 -0.147764 1.032406
H -2.925623 -0.713870 1.820716
C -4.753463 0.218862 1.156422
H -5.305305 -0.060973 2.049839
C -5.384850 0.937081 0.140200
H -6.429875 1.218354 0.238150
C -4.668481 1.287974 -1.006489
H -5.153310 1.842016 -1.805718
C -3.328315 0.923522 -1.131154
H -2.769939 1.201340 -2.022271
N -0.876259 -1.464530 0.328299
C -1.075291 -2.809874 -0.322314
C 0.205296 -3.625001 -0.066231
C -2.279780 -3.497543 0.348417
C -1.311151 -2.664931 -1.832046
O -0.611658 -1.455179 1.586185
H -1.061647 -0.304302 -1.378244
H 0.081040 -4.642609 -0.451555
H 0.412528 -3.677702 1.005314
H 1.065587 -3.170020 -0.568704
H -2.394530 -4.514258 -0.043117
H -3.206718 -2.948330 0.155793
H -2.125404 -3.554723 1.429411
H -2.237964 -2.125819 -2.055018
H -1.403297 -3.665892 -2.266434
H -0.477501 -2.160174 -2.331437
56
-1550.430586
S -1.043594 -0.778672 -1.376775
N 0.157801 -0.825035 -0.186281
C -2.559184 -0.440982 -0.469092
C -3.723078 -1.104586 -0.866680
H -3.674688 -1.852313 -1.650758
C -4.926463 -0.800298 -0.236312
H -5.832978 -1.317134 -0.542763
C -4.987412 0.155542 0.788858
C -6.300594 0.472386 1.464205
C -3.802753 0.799043 1.169886
H -3.827818 1.535275 1.969411
C -2.586922 0.513750 0.548500
H -1.674749 1.006021 0.871677
O -0.730791 0.371647 -2.240806
O -1.205220 -2.105648 -1.986627
C 0.045639 -1.978517 0.764322
C 0.886698 -3.197783 0.358268
H 0.586046 -4.058630 0.967765
C 0.244831 -1.588235 2.232185
H 1.954708 -3.031975 0.517168
H 0.715335 -3.447303 -0.692214
H -6.734515 -0.421183 1.929454
H -7.036731 0.848379 0.743115
H -6.178115 1.230229 2.243726
H -0.054777 -2.437343 2.857984
H -0.357415 -0.718704 2.498319
H 1.289420 -1.363479 2.457015
H -1.003162 -2.279801 0.669862
C 1.269746 0.116314 -0.364665
C 2.644563 -0.404036 0.053157
C 3.231461 -0.085134 1.283627
H 2.683618 0.525102 1.993645
C 4.513904 -0.543637 1.592599
H 4.956063 -0.291205 2.552673
C 5.227575 -1.315489 0.674779
H 6.226508 -1.668130 0.916705
C 4.655391 -1.622080 -0.561929
H 5.207350 -2.211715 -1.288877
C 3.375488 -1.163579 -0.871177
H 2.933708 -1.404177 -1.835486
N 0.972207 1.435338 0.259708
C 1.295705 2.769876 -0.362262
C 2.290993 3.487350 0.568783
C 1.901940 2.612497 -1.763732
C -0.025118 3.556896 -0.447164
O 0.499344 1.450483 1.452601
H 1.325808 0.318634 -1.429847
H 1.883000 3.552352 1.580419
H 2.481487 4.500913 0.199614
H 3.245574 2.951677 0.610230
H 1.199477 2.147804 -2.461652
H 2.833617 2.036376 -1.746990
H 2.142181 3.609710 -2.147658
H -0.474037 3.649798 0.545295
H -0.731786 3.048205 -1.110301
H 0.163212 4.561602 -0.841396
56
-1550.430303
S 0.898499 -1.280180 -0.363973
N -0.069958 -0.076597 0.346037
C 2.558796 -0.601073 -0.329520
C 3.528360 -1.206796 0.466343
H 3.264269 -2.063529 1.076610
C 4.829407 -0.700510 0.454796
H 5.587809 -1.168777 1.077007
C 5.174746 0.393121 -0.347372
C 6.584993 0.932236 -0.368722
C 4.179329 0.972446 -1.152694
H 4.431350 1.816753 -1.790003
C 2.878744 0.481830 -1.153400
H 2.119554 0.925782 -1.788617
O 0.920233 -2.521468 0.421482
O 0.476562 -1.338645 -1.774657
C 0.112190 0.357590 1.776575
C 0.344150 -0.775469 2.784226
H 1.331946 -1.232852 2.681847
C 1.162114 1.470526 1.903391
H -0.414476 -1.552020 2.677331
H 0.272809 -0.354434 3.793985
H 6.599804 2.016184 -0.205350
H 7.063799 0.746469 -1.338506
H 7.204609 0.465605 0.402766
H 0.967996 2.273948 1.186576
H 2.180133 1.103175 1.747043
H 1.113436 1.894529 2.913488
H -0.858656 0.789097 2.019034
C -1.398139 0.091361 -0.280044
C -1.858729 1.549425 -0.209282
C -1.183815 2.484148 -1.008464
H -0.350672 2.154947 -1.624697
C -1.571902 3.822198 -1.023827
H -1.040748 4.531542 -1.653100
C -2.646228 4.247224 -0.237832
H -2.952612 5.289793 -0.248990
C -3.324292 3.323644 0.556327
H -4.161903 3.644105 1.170233
C -2.936707 1.980761 0.571457
H -3.464747 1.268991 1.195367
N -2.412011 -0.849029 0.243400
C -3.197971 -1.846635 -0.570063

C -4.685362 -1.615380 -0.245787
C -2.957191 -1.665414 -2.074533
C -2.761040 -3.252824 -0.118016
O -2.615007 -0.852104 1.512442
H -1.258922 -0.155509 -1.327771
H -5.009504 -0.625026 -0.585425
H -4.855532 -1.687197 0.830745
H -5.297321 -2.369745 -0.751773
H -1.921796 -1.874729 -2.357494
H -3.227230 -0.657648 -2.411490
H -3.598497 -2.371660 -2.612430
H -1.701694 -3.423880 -0.332884
H -3.351832 -4.014051 -0.639857
H -2.916588 -3.364327 0.958371
56
-1550.429678
S -1.043698 -0.778327 -1.377378
N 0.157585 -0.824869 -0.186764
C -2.559680 -0.443653 -0.469451
C -2.589584 0.516406 0.545266
H -1.678119 1.011630 0.865838
C -3.803452 0.797514 1.168586
H -3.829267 1.534446 1.967761
C -4.987646 0.146654 0.791776
C -6.300702 0.487921 1.454884
C -4.923865 -0.814981 -0.225288
H -5.827492 -1.341283 -0.523076
C -3.719419 -1.115760 -0.859075
H -3.669037 -1.869710 -1.637035
O -0.732526 0.374251 -2.239042
O -1.203271 -2.104428 -1.989704
C 0.045269 -1.978339 0.763793
C 0.886926 -3.197407 0.358372
H 0.586121 -4.058321 0.967706
C 0.243327 -1.587837 2.231751
H 1.954801 -3.031369 0.517928
H 0.716306 -3.447036 -0.692214
H -6.160357 0.760287 2.506216
H -7.003168 -0.350536 1.410315
H -6.780082 1.342569 0.959213
H -0.057021 -2.436713 2.857497
H -0.358810 -0.718003 2.497117
H 1.287834 -1.363366 2.457357
H -1.003363 -2.280044 0.668679
C 1.269564 0.116381 -0.364999
C 2.644139 -0.403806 0.053424
C 3.230106 -0.085299 1.284454
H 2.681713 0.524714 1.994231
C 4.512371 -0.543787 1.594185
H 4.953817 -0.291712 2.554679
C 5.226783 -1.315207 0.676575
H 6.225548 -1.667904 0.919116
C 4.655571 -1.621321 -0.560696
H 5.208184 -2.210536 -1.287487
C 3.375838 -1.162857 -0.870701
H 2.934792 -1.403077 -1.835436
N 0.971700 1.435654 0.258934
C 1.296281 2.769931 -0.363044
C 1.902150 2.612202 -1.764619
C -0.023870 3.558083 -0.447756
C 2.292333 3.486531 0.567885
O 0.498215 1.451220 1.451542
H 1.325905 0.318611 -1.430206
H 1.199205 2.148065 -2.462441
H 2.833420 2.035430 -1.748119
H 2.143042 3.609274 -2.148505
H 0.165179 4.562582 -0.842198
H -0.472493 3.651529 0.544783
H -0.731102 3.049879 -1.110677
H 2.483674 4.499939 0.198739
H 3.246444 2.949998 0.609186
H 1.884515 3.551837 1.579573

12-PBN

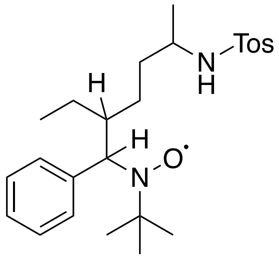

Name	E(B3LYP)	H(B3LYP)	g-factor	α_N	α_H
Tosyl_N_radical_2_heptyl_C5_radical_PBN_adduct_0015	-1708.197895	-1707.575926	2.00587237	14.709420	2.233100
Tosyl_N_radical_2_heptyl_C5_radical_PBN_adduct_0070	-1708.197890	-1707.575755	2.00604370	15.684090	2.502760
Tosyl_N_radical_2_heptyl_C5_radical_PBN_adduct_0020	-1708.197659	-1707.575743	2.00588187	14.810850	2.444950
Tosyl_N_radical_2_heptyl_C5_radical_PBN_adduct_0002	-1708.197108	-1707.575211	2.00586663	14.693160	3.041900
Tosyl_N_radical_2_heptyl_C5_radical_PBN_adduct_0248	-1708.197009	-1707.575207	2.00621983	14.931000	2.826430
Tosyl_N_radical_2_heptyl_C5_radical_PBN_adduct_0036	-1708.196964	-1707.575081	2.00609713	15.832100	1.262250
Tosyl_N_radical_2_heptyl_C5_radical_PBN_adduct_0032	-1708.196738	-1707.574687	2.00601883	15.504430	3.421380
Tosyl_N_radical_2_heptyl_C5_radical_PBN_adduct_0142	-1708.196712	-1707.574631	2.00584103	14.611170	5.311740
Boltzman averaged for 298.15 K			2.005965	15.0852	2.4188

68

C 2.9070416868 0.1341386417 0.3797487861
C 3.8715253808 -0.4598236085 -1.9066677587
H 2.8558005334 -0.5349650354 -2.2814468662
C 4.9521873458 -0.6969307281 -2.7561129029
H 4.7705814176 -0.9392754641 -3.8000508068
C 6.2607529302 -0.6274465257 -2.2723377388
H 7.1006998732 -0.8114953383 -2.9368490656
C 6.4811613928 -0.3262511559 -0.9283163858
H 7.4941667728 -0.2762732025 -0.5376100777
C 5.3985259556 -0.0938529213 -0.0779258248
H 5.5815796786 0.1307796849 0.9706087290
C 4.0828169881 -0.1508327034 -0.5545683033
N 1.9259713780 -0.9860101435 0.3374928729
C 1.9429894142 -2.1288924736 1.3204661134
C 3.3652106264 -2.7148639772 1.4089324471
C 0.9675027680 -3.2026798173 0.8223216106
C 1.4838151356 -1.6114532705 2.6987639741
H 3.3545204351 -3.5749963702 2.0873422814
H 4.0906667090 -1.9957104315 1.8008224135
H 3.7148515971 -3.0533158154 0.4288946712
H 0.9954989248 -4.0454510035 1.5215599212
H 1.2491115018 -3.5636105661 -0.1705681924
H -0.0546162647 -2.8219634151 0.7794463146
H 0.4513209498 -1.2530581256 2.6531382506
H 2.1254694444 -0.8054820048 3.0720327751
H 1.5274515737 -2.4296255691 3.4264821534
O 1.1039358551 -1.0121055445 -0.6520722523
C -0.9759187996 1.8857088607 -0.6553048412
N -1.4036990823 0.5031280403 -0.3299630472
S -2.4641767943 0.1216443389 0.8992773307
O -2.0052809421 -1.1767472196 1.4225903154
O -2.6622388003 1.2604709360 1.8087897648
C -4.0320447057 -0.1500001806 0.0645958665
C -4.1101268789 -1.0650467306 -0.9895938456
C -5.1707022867 0.5064621934 0.5241685544
C -5.3410284571 -1.3084618064 -1.5881179536
H -3.2161448219 -1.5718346111 -1.3385346620
C -6.3993637444 0.2499178309 -0.0882064253
H -5.0887574767 1.2081964033 1.3470137086
C -6.5053934618 -0.6555377968 -1.1490812000
H -7.2890347072 0.7632660748 0.2682674184
C -0.1445272083 2.5811338219 0.4489516317
H 0.2376998841 3.5217123585 0.0273335713
H -0.8186287094 2.8534012687 1.2671177162
C 1.0121646280 1.7479148657 1.0281294941
H 0.5994611298 0.8033778207 1.3930110852
H 1.3932166981 2.2600775771 1.9199795382
C 2.1795618781 1.4730154293 0.0497001433
H 1.7609945609 1.3239472087 -0.9512780837
C 3.1482857363 2.6753055378 -0.0588889324
H 2.5563378196 3.5440542485 -0.3764743388
C 3.9313016742 3.0551544017 1.2055578411
H 4.5547087286 3.9353273039 1.0116634527
H 3.2731558099 3.3026080884 2.0455051083
H 4.6009924039 2.2521872679 1.5322631464
H -5.4031199662 -2.0176006163 -2.4104539699
C -7.8322815216 -0.9253199854 -1.8182041566
H -8.0554221977 -1.9986434015 -1.8426344305
H -8.6530882524 -0.4227455203 -1.2976646606
H -7.8317201701 -0.5737786846 -2.8578290479
C -2.1585900536 2.7516585464 -1.1002700994
H 3.2817868218 0.1719548750 1.4073272912
H -0.6425533048 -0.1828655292 -0.3592365284
H -0.3341218050 1.7298827996 -1.5307612630
H 3.8585331486 2.4857830829 -0.8717238353
H -2.7130576997 2.2660737948 -1.9093540039
H -1.7945494163 3.7195703458 -1.4622609067
H -2.8450922511 2.9410490008 -0.2695903273
68

C 2.7794984437 0.1189586541 0.7788480408
C 2.5917683716 -0.8583723063 -1.5712311462
H 1.5151894353 -0.9188055735 -1.4461445614
C 3.1712223141 -1.2841151489 -2.7663268125
H 2.5356516508 -1.6706729551 -3.5584131588
C 4.5542404294 -1.2106591723 -2.9507817740
H 4.9997650333 -1.5430743672 -3.8846745116
C 5.3592922141 -0.7026540680 -1.9312824449
H 6.4357913489 -0.6343213361 -2.0652631438
C 4.7804456843 -0.2765505623 -0.7338960439
H 5.4134033279 0.1291163978 0.0528896590
C 3.3951692172 -0.3506179628 -0.5383490895
N 2.1204468196 -1.0235007854 1.4739923365
C 2.8561315569 -2.0564674434 2.2895827799
C 4.1587841862 -1.4862699411 2.8665710867
C 3.1492904327 -3.2727228648 1.3890203266
C 1.9183222205 -2.4631237001 3.4403544581
H 4.8917641571 -1.2526129977 2.0890988143

H 4.6076726884 -2.2397111362 3.5223050585
H 3.9791360471 -0.5889037895 3.4692692181
H 3.8419247724 -3.0118348959 0.5836135854
H 2.2206265589 -3.6391917227 0.9436144803
H 3.5962144102 -4.0777749043 1.9832053629
H 0.9701842224 -2.8363712736 3.0489968521
H 1.7128013854 -1.6102419114 4.0973877640
H 2.3887115387 -3.2505723605 4.0386277290
O 0.9562274844 -1.3834769076 1.0599430620
C -1.4834227960 1.1807771263 1.7475800604
N -1.4049258361 0.2678170079 0.5885616743
S -1.8388987278 0.7588963414 -0.9408316612
O -1.7890842456 2.2252080220 -1.0678079742
O -1.0679223254 -0.0967654509 -1.8555090010
C -3.5717097440 0.2996020078 -1.0589553624
C -4.5322383624 1.2835948705 -1.2770082481
C -3.9344188420 -1.0481506619 -0.9695491553
C -5.8735308076 0.9120085193 -1.3970283276
H -4.2266990616 2.3213607124 -1.3534569262
C -5.2731741451 -1.4004963967 -1.0925342110
H -3.1748028997 -1.8057072949 -0.8054096905
C -6.2648231561 -0.4276833367 -1.3075896040
H -5.5578979034 -2.4479133372 -1.0226632988
C -0.3435083392 2.2253508400 1.8338482879
H -0.4372353540 2.8872172958 0.9703819889
H -0.5573542337 2.8397178452 2.7199979940
C 1.1070982683 1.6996553335 1.9482513277
H 1.1257882702 0.8471379792 2.6357952831
H 1.7128376942 2.4776489390 2.4336507532
C 1.8003219534 1.3184399870 0.6097359782
H 1.0448338054 1.0190490221 -0.1210229800
C 2.5872868781 2.5041307232 0.0022978007
H 3.0823308885 2.1628189267 -0.9132696769
C 1.7548990917 3.7470941221 -0.3361578183
H 2.3752266833 4.4822975595 -0.8618927209
H 0.9072187692 3.4966045814 -0.9835754915
H 1.3591466160 4.2377272033 0.5596478094
H -6.6253363812 1.6791356897 -1.5656672194
C -7.7140897710 -0.8307443846 -1.4430588811
H -7.8648999792 -1.4842252512 -2.3113805215
H -8.0578956958 -1.3849640473 -0.5611374106
H -8.3630735831 0.0416729762 -1.5652460972
C -1.6161477197 0.3134863508 3.0061422453
H 3.5870825337 0.4444010542 1.4394686627
H -0.5726012827 -0.3307597374 0.5634788543
H -2.4151296163 1.7467529953 1.6289956398
H 3.3924041326 2.7870330510 0.6986939270
H -1.6720789436 0.9417846781 3.9024411383
H -0.7623467408 -0.3634822188 3.1131447948
H -2.5210483711 -0.2991408064 2.9501766729
68

C 2.7018722426 0.1323520947 0.7241496063
C 2.4260930165 -0.7618961695 -1.6489152958
H 1.3488922803 -0.7316223415 -1.5183384860
C 2.9630640418 -1.2040818709 -2.8577118064
H 2.2945107168 -1.5138713178 -3.6564661490
C 4.3468000421 -1.2446198186 -3.0480103601
H 4.7591069317 -1.5888624274 -3.9928199501
C 5.1956193142 -0.8361525624 -2.0195977167
H 6.2736439418 -0.8588795206 -2.1567263419
C 4.6591279364 -0.3941157978 -0.8081178414
H 5.3285389435 -0.0726966162 -0.0127219939
C 3.2733469205 -0.3516732194 -0.6073696537
N 1.9688880032 -0.9758520218 1.4079090068
C 2.6350310891 -2.0737011664 2.1965696412
C 3.9878304235 -1.6153741029 2.7573875116
C 2.8163932749 -3.2959511725 1.2749425358
C 1.6877148823 -2.4214787418 3.3590552546
H 4.7234206345 -1.4275038710 1.9701381806
H 4.3877705258 -2.4111795591 3.3945266349
H 3.8887781092 -0.7160506086 3.3755513456
H 3.2124899401 -4.1414564671 1.8486781025
H 3.5103763933 -3.0764437674 0.4583058809
H 1.8535662122 -3.5840814025 0.8447129338
H 2.1011834951 -3.2557792071 3.9355186820
H 0.7046303256 -2.7066043627 2.9800606966
H 1.5664382080 -1.5659492498 4.0334536468
O 0.7720849502 -1.2361367693 1.0129558961
C -1.4687728517 1.6384015861 1.7914782276
N -1.5063922914 0.6400667000 0.7033029234
S -1.9748042883 1.0479835381 -0.8419785496
O -2.2236659713 2.4956288826 -0.8994735115
O -1.0281341473 0.3939928389 -1.7595098753
C -3.5628040621 0.2380228923 -1.0683861001
C -4.7378396853 0.9780592052 -0.9507110199
C -3.6041693245 -1.1278368313 -1.3596753575
C -5.9661606535 0.3368445351 -1.1163819825
H -4.6847533621 2.0418238545 -0.7455750586
C -4.8376866920 -1.7511740471 -1.5210539579
H -2.6808635323 -1.6875509881 -1.4681645380
C -6.0374071851 -1.0316884316 -1.4026327276
H -4.8718614283 -2.8143571930 -1.7477781571
C -0.2038649912 2.5319001129 1.7954243453
H -0.2198459863 3.1406261726 0.8840395542
H -0.3206352204 3.2339252810 2.6324044859
C 1.1564329802 1.8097283940 1.9289066677
H 1.0458156796 0.9438453883 2.5896738679
H 1.8585484134 2.4780464912 2.4427691156
C 1.7780386862 1.3714971638 0.5771649274
H 0.9705112968 1.0745293119 -0.0952323611
C 2.5021260512 2.5444905813 -0.1305476764
H 1.7810286797 3.3674289420 -0.2159978249
C 3.7817377893 3.0743368698 0.5312552132
H 3.6198113476 3.3843325621 1.5701882417
H 4.5863344765 2.3303123993 0.5259531990
H 4.1516383675 3.9502844727 -0.0134574277
H -6.8839205374 0.9131094789 -1.0264653694
C -7.3678072586 -1.7164965705 -1.6089498250
H -7.3881701234 -2.7037575853 -1.1340094788
H -8.1915706976 -1.1248678329 -1.1974537375
H -7.5728012104 -1.8679752254 -2.6769652924
C -1.6928032068 0.8953562483 3.1152990853
H 3.5317709201 0.3909826410 1.3845909560
H -0.7361710373 -0.0365034630 0.6966155115
H -2.3215116662 2.3034064551 1.6236278038
H 2.7333465073 2.2458280850 -1.1590069789
H -0.9227694329 0.1348586342 3.2845061806
H -2.6623420575 0.3884319532 3.1047776522
H -1.6714165991 1.5957344084 3.9581627338
68

C 2.9459718398 0.0539720179 0.4089097026
C 3.9306886081 -0.5043824858 -1.8796708678
H 2.9194703036 -0.5465087042 -2.2714377204
C 5.0200346351 -0.7439509746 -2.7178423422
H 4.8501473773 -0.9578491974 -3.7699358624
C 6.3217308468 -0.7120528039 -2.2127547907
H 7.1681133002 -0.8986178568 -2.8683337558
C 6.5275679019 -0.4428438179 -0.8592531812
H 7.5354898596 -0.4187395145 -0.4532380929
C 5.4368956948 -0.2082223636 -0.0203934058
H 5.6061941293 0.0000784704 1.0340886604
C 4.1276567165 -0.2324892035 -0.5181376928
N 1.9212208051 -1.0140681048 0.2881542875
C 1.8960816766 -2.2292519359 1.1825984498
C 1.4544513430 -1.8011760550 2.5966750467
C 3.2960538499 -2.8719952858 1.2260353546
C 0.8851923945 -3.2286871689 0.6063730702
H 1.4691087472 -2.6732134919 3.2601016263
H 0.4349426191 -1.4050996791 2.5820723423
H 2.1245442826 -1.0493861740 3.0284091215
H 3.6344608057 -3.1518781985 0.2239088460
H 3.2516412160 -3.7777978455 1.8406226122
H 4.0472932152 -2.2107150275 1.6677540394
H 1.1496554240 -3.5157379592 -0.4147956145
H -0.1266113849 -2.8189143443 0.6058609037
H 0.8921937902 -4.1259420801 1.2346439728
O 1.1188127050 -0.9446179557 -0.7158700276
C -0.8987938083 1.8137120552 -0.5633518673

N -1.3604209945 0.4441973369 -0.2359956122
S -2.4368948325 0.0761671509 0.9776518311
O -2.0252620815 -1.2497157578 1.4721395846
O -2.5993677809 1.1988276461 1.9139364858
C -4.0123466842 -0.1205752236 0.1354001382
C -4.1134432200 -0.9777649503 -0.9642474001
C -5.1364514299 0.5320536161 0.6352886472
C -5.3520316632 -1.1673717865 -1.5668900297
H -3.2305885365 -1.4806983689 -1.3455460387
C -6.3728154144 0.3301338468 0.0184250679
H -5.0370168008 1.1894675919 1.4921229920
C -6.5014701986 -0.5168547943 -1.0875182509
H -7.2508364314 0.8405933226 0.4065894566
C -0.0788649282 2.5077327133 0.5480417510
H 0.2703838187 3.4621363967 0.1364915946
H -0.7584577395 2.7476397657 1.3719770439
C 1.1062695334 1.7074993267 1.1234752028
H 0.7163044191 0.7520085384 1.4869385215
H 1.4680216472 2.2320937839 2.0187495476
C 2.3094934698 1.4623884366 0.1734467441
H 1.9596587820 1.4710241441 -0.8656501449
C 3.4028768308 2.5451045017 0.3227758900
H 4.2336207346 2.3083476255 -0.3500026103
C 2.9542883570 3.9841670947 0.0419334476
H 2.2193650782 4.3415977985 0.7702264716
H 3.8139529487 4.6627486708 0.0858867082
H 2.5115669892 4.0774507783 -0.9573332028
H -5.4316674033 -1.8316009849 -2.4244572311
C -7.8360567707 -0.7225963550 -1.7640521891
H -8.6519915124 -0.2821663656 -1.1829939252
H -7.8511154375 -0.2619235288 -2.7602452074
H -8.0546393381 -1.7882723085 -1.8998686873
C -2.0592293111 2.6972297127 -1.0335689458
H 3.3051710747 0.0158248356 1.4433187601
H -0.6206051832 -0.2622098203 -0.2890032985
H -0.2431816326 1.6369675492 -1.4243021892
H 3.8127535927 2.4941797579 1.3433080153
H -2.6122587066 2.2126962692 -1.8441932303
H -1.6746111798 3.6552116167 -1.4005013252
H -2.7536674684 2.9071210319 -0.2139838143
68

C 2.8916036697 0.0864643220 0.4594579510
C 3.9779539388 -0.5506955188 -1.7616611658
H 2.9851276921 -0.7199947631 -2.1666044964
C 5.1056362782 -0.7388179479 -2.5608869276
H 4.9842407266 -1.0483613242 -3.5957521004
C 6.3850351927 -0.5321832503 -2.0388293797
H 7.2615465041 -0.6781808357 -2.6643780675
C 6.5297706073 -0.1445107867 -0.7064969601
H 7.5201270434 0.0111279040 -0.2866399780
C 5.4008269476 0.0365890225 0.0948454885
H 5.5231630563 0.3303162537 1.1353360067
C 4.1137695833 -0.1544543610 -0.4231328177
N 1.9190663781 -1.0316969200 0.3226013933
C 1.9256607668 -2.2389615839 1.2258493446
C 1.4243517849 -1.8232954778 2.6239914814
C 3.3525303989 -2.8136264591 1.3140530150
C 0.9770921860 -3.2863170245 0.6292042752
H 2.0482565824 -1.0407461085 3.0700388767
H 1.4570533328 -2.6904200863 3.2932614647
H 0.3898897939 -1.4711958913 2.5758180645
H 3.3358790174 -3.7109658889 1.9422614628
H 4.0603977334 -2.1102713154 1.7628349314
H 3.7291656174 -3.0922328435 0.3252875764
H -0.0482003487 -2.9144923838 0.5861552014
H 0.9984366103 -4.1757774827 1.2682166392
H 1.2880101078 -3.5737535295 -0.3789309523
O 1.1020930767 -0.9854991062 -0.6710880356
C -0.9770163197 1.9411833125 -0.4396389925
N -1.4045193660 0.5350721103 -0.2368872149
S -2.4631582553 0.0493783285 0.9571547802
O -2.0159143204 -1.2979174269 1.3506268301
O -2.6431593626 1.0969706243 1.9739004919
C -4.0383121692 -0.1228625651 0.1101237613

C -4.1292241623 -0.9153341649 -1.0380977899
C -5.1705507869 0.4809665079 0.6507780716
C -5.3666087210 -1.0886146068 -1.6476513855
H -3.2398568230 -1.3809436062 -1.4503736170
C -6.4057922994 0.2960132333 0.0260723969
H -5.0785473486 1.0880158154 1.5447976418
C -6.5247806752 -0.4864812687 -1.1272649885
H -7.2903860140 0.7687453141 0.4457302422
C -0.1348785781 2.5343083180 0.7152304302
H 0.2516298762 3.5051285193 0.3725623777
H -0.8008761122 2.7390347703 1.5593069089
C 1.0196557786 1.6466439862 1.2076977292
H 0.6016936797 0.6825304441 1.5096664321
H 1.4355432377 2.0887025308 2.1239863857
C 2.1703314631 1.4427055335 0.1916765533
H 1.7470498143 1.3604940133 -0.8146153642
C 3.1367623793 2.6568562242 0.2189080565
H 3.9523865354 2.4677143177 0.9309336351
C 3.7190122746 3.0771600966 -1.1366533229
H 2.9162563043 3.3224852891 -1.8433035215
H 4.3462894571 3.9697826342 -1.0266265102
H 4.3304713163 2.2919711911 -1.5882728779
H -5.4386391884 -1.7017751509 -2.5430918362
C -7.8591095638 -0.6794532541 -1.8080318838
H -8.6712962260 -0.2223721454 -1.2347402118
H -7.8631763960 -0.2301787809 -2.8092275485
H -8.0922981816 -1.7436657431 -1.9330558611
C -2.1617240745 2.8448157372 -0.7952216735
H 3.2210771168 0.0816924695 1.5046378512
H -0.6446314899 -0.1472130356 -0.3259635710
H -0.3424204835 1.8651417269 -1.3307020410
H 2.5871253136 3.5136235712 0.6290605661
H -2.7231049616 2.4351261910 -1.6406788763
H -1.7984592149 3.8410019399 -1.0709414689
H -2.8417207942 2.9593204563 0.0542831569
68

C 2.7552456303 0.0395583478 -0.2263228799
C 5.0966047714 -0.5270723291 -1.0668417112
H 4.6869698006 -0.5831398238 -2.0707298136
C 6.4522018120 -0.7674596335 -0.8445902206
H 7.1024354042 -0.9931978677 -1.6859440863
C 6.9755131472 -0.7222903259 0.4503425553
H 8.0324225628 -0.9099556716 0.6201125444
C 6.1313438749 -0.4389980438 1.5240141831
H 6.5260671773 -0.4045174522 2.5361762938
C 4.7731334433 -0.2017345770 1.3009955208
H 4.1211777505 0.0205024731 2.1433171028
C 4.2398644369 -0.2387680885 0.0062668073
N 2.1257381368 -1.0702552208 -0.9898227550
C 1.6948403901 -2.3838725201 -0.3879410508
C 0.3495884002 -2.7452789537 -1.0434549330
C 1.5183415662 -2.2799374901 1.1324719079
C 2.7598644024 -3.4424171044 -0.7354645137
H 0.4456944446 -2.7371607513 -2.1316718671
H -0.4258195103 -2.0311496346 -0.7466029935
H 0.0366256852 -3.7448870913 -0.7222161282
H 1.1823564783 -3.2531430791 1.5065246857
H 0.7535986987 -1.5473589866 1.4088446253
H 2.4577859264 -2.0431936019 1.6419862958
H 3.7158105438 -3.2137509581 -0.2538143370
H 2.9147317544 -3.4759941440 -1.8170694777
H 2.4299832204 -4.4304108984 -0.3951455884
O 2.2727128262 -1.0563804557 -2.2677338646
C -1.4176667827 1.8749012565 -0.4309226430
N -2.2789357332 1.9027676851 0.7775798825
S -2.6688375116 0.4676026027 1.5691465151
O -1.6617753162 -0.5791874897 1.3246840024
O -2.9960917050 0.8775651100 2.9396560009
C -4.1619846274 -0.0475742018 0.7212130426
C -4.2108489208 -1.3031530931 0.1206638466
C -5.2810968838 0.7914943794 0.7245859513
C -5.3941131404 -1.7163757012 -0.4951291420
H -3.3349477266 -1.9421271625 0.1392896007
C -6.4502630124 0.3634505045 0.1073041674

H -5.2305733493 1.7645160760 1.2020240044
C -6.5265989690 -0.8954815345 -0.5134318200
H -7.3218744308 1.0137655940 0.1067268095
C 0.0793834607 1.8292030310 -0.0666401316
H 0.3341339678 2.7604038434 0.4617402733
H 0.2132059963 1.0116418554 0.6486758038
C 1.0052161387 1.6292583858 -1.2771503624
H 0.9437673403 2.5047253935 -1.9342361549
H 0.6498411892 0.7815705585 -1.8718117449
C 2.5003394801 1.3956681163 -0.9507768121
H 3.0172781959 1.3307676066 -1.9153737517
C 3.1329076242 2.5586111619 -0.1551070013
H 4.1897681312 2.3310801837 0.0196504028
C 3.0392987560 3.9218868404 -0.8517026816
H 3.4664141291 3.8794501069 -1.8613204390
H 2.0060255465 4.2746015337 -0.9437330339
H 3.5947763319 4.6806223258 -0.2888659469
H -5.4342980585 -2.6948673135 -0.9668801435
C -7.8042581615 -1.3399114641 -1.1843631423
H -8.0535134787 -0.6899058215 -2.0324192985
H -7.7244436228 -2.3640143902 -1.5607693551
H -8.6520430143 -1.3008575311 -0.4899210850
C -1.8024024923 3.0720504402 -1.3068575705
H 2.2614111683 0.0691005844 0.7474019397
H -1.9857610330 2.5756235596 1.4847358193
H -1.6759870740 0.9534674109 -0.9656591643
H 2.6727535466 2.6260036125 0.8412170561
H -1.2544894892 3.0570492460 -2.2539967022
H -1.5712986168 4.0174300685 -0.7993096252
H -2.8741821392 3.0566767659 -1.5250266349
68

C 2.7962653944 0.1546249146 0.8075300968
C 2.5361930053 -0.8146175685 -1.5432092070
H 1.4606232385 -0.8496323552 -1.4034573367
C 3.0854115912 -1.2547076645 -2.7477063289
H 2.4262461619 -1.6234365390 -3.5289129950
C 4.4664141212 -1.2181958809 -2.9550602029
H 4.8875909107 -1.5618158421 -3.8962137099
C 5.3014444715 -0.7324318997 -1.9487743116
H 6.3771117804 -0.6927956395 -2.1000326829
C 4.7532560955 -0.2914376090 -0.7425619473
H 5.4098419330 0.0967015430 0.0338146572
C 3.3694465248 -0.3287908566 -0.5238437231
N 2.1472419052 -0.9770109711 1.5287565457
C 2.8921397365 -1.9834457121 2.3703414504
C 1.9442627917 -2.3924732984 3.5119890738
C 4.1733627282 -1.3785793808 2.9596913814
C 3.2244960169 -3.2055598303 1.4917703912
H 1.7148531294 -1.5354310358 4.1553056042
H 2.4183915348 -3.1654676826 4.1259425475
H 1.0082341033 -2.7854213215 3.1108440634
H 4.9190809944 -1.1508088900 2.1923935074
H 4.6209693186 -2.1087892123 3.6420468919
H 3.9670258278 -0.4702643657 3.5369776213
H 3.9215446208 -2.9405627219 0.6914132655
H 2.3104794444 -3.5994452526 1.0396620446
H 3.6818912030 -3.9914711153 2.1034015096
O 1.0026901285 -1.3740898587 1.0938286413
C -1.3246016456 1.3899582736 1.6043707253
N -1.3376815879 0.3307397477 0.5668022768
S -1.7764669046 0.6558423596 -1.0075937171
O -1.6854730569 2.1003258277 -1.2748018616
O -1.0322805888 -0.3128741628 -1.8269597585
C -3.5170603219 0.2237890945 -1.1078004870
C -4.4680912866 1.2238351098 -1.2945802119
C -3.8965109508 -1.1191828202 -1.0225321943
C -5.8169725506 0.8725840626 -1.3846247334
H -4.1495538538 2.2574687593 -1.3754007408
C -5.2433376944 -1.4509402152 -1.1150826152
H -3.1431027719 -1.8894408019 -0.8927838772
C -6.2256299900 -0.4623485936 -1.2958359167
H -5.5411251692 -2.4949928027 -1.0497083552
C -0.1025781603 1.1891465847 2.5266327057
H -0.3389564070 1.6722621313 3.4838173368
H 0.0014990964 0.1217105005 2.7448283181
C 1.2284039423 1.8027206859 2.0404437456
H 1.9833034174 1.6398359868 2.8243553202
H 1.0779756159 2.8883914162 2.0079075430
C 1.8332373731 1.3702147546 0.6764049429
H 1.0348031141 1.0934939840 -0.0154109617
C 2.5992761828 2.5524235126 0.0326361714
H 3.1938860831 2.1785295414 -0.8074460903
C 1.6933595963 3.6772178791 -0.4849310823
H 1.1475881284 4.1812175614 0.3208879803
H 2.2888962843 4.4404896129 -0.9988441388
H 0.9497399783 3.2920623007 -1.1911785839
H -6.5611898304 1.6515199774 -1.5313249034
C -7.6835404628 -0.8430061911 -1.3998092811
H -7.8662379802 -1.4797778912 -2.2744190913
H -8.0120604711 -1.4069155161 -0.5182742124
H -8.3229311875 0.0402345722 -1.4908406418
C -2.6373493793 1.3595141322 2.3981514038
H 3.6276013744 0.4756178352 1.4397981528
H -0.5464755977 -0.3198351702 0.5873592399
H -1.2376059105 2.3627586366 1.1046868248
H 3.3181108014 2.9568551819 0.7622051571
H -2.7367251901 0.4135682571 2.9429349926
H -3.5007934645 1.4565670482 1.7323232380
H -2.6702922557 2.1832619784 3.1213711357
68

C 2.7487876626 0.1285264635 0.8722368086
C 2.7357391435 -0.7275669921 -1.5286317857
H 1.6809438580 -0.9614205995 -1.4197130092
C 3.3805057281 -0.9786958334 -2.7390604139
H 2.8195802945 -1.4110688462 -3.5631900124
C 4.7346280471 -0.6704698309 -2.8985551385
H 5.2316575876 -0.8657485587 -3.8450792720
C 5.4434922331 -0.1082011586 -1.8372799665
H 6.4960198855 0.1381803146 -1.9508118820
C 4.7995537910 0.1394896630 -0.6225145866
H 5.3570584085 0.5827534893 0.2002298963
C 3.4427548135 -0.1631159457 -0.4551881747
N 2.1613355699 -1.1233027723 1.4342531812
C 2.9542718300 -2.1891541435 2.1445590622
C 4.2532088244 -1.6197525294 2.7302775056
C 3.2639917761 -3.3179147915 1.1415474015
C 2.0628887424 -2.7201205715 3.2818215963
H 4.9432592073 -1.2770017966 1.9539112392
H 4.7585325750 -2.4131655550 3.2909667770
H 4.0603157097 -0.7958905167 3.4265371069
H 3.9243819739 -2.9674486042 0.3429950058
H 2.3365550197 -3.6810220035 0.6910187634
H 3.7549149926 -4.1511305832 1.6569186803
H 1.1153565985 -3.0888011468 2.8842419628
H 1.8510486500 -1.9312420628 4.0127426843
H 2.5724173844 -3.5393578620 3.8000991837
O 1.0059213144 -1.4936866793 1.0035476588
C -1.6237257458 1.0189101659 1.8168343686
N -1.4807660041 0.1233732674 0.6489351371
S -1.8441699693 0.6543958788 -0.8892520878
O -1.6706315925 2.1117696058 -1.0188696560
O -1.1286284683 -0.2590382695 -1.7912403044
C -3.6079382033 0.3407976611 -1.0209935039
C -4.0882104218 -0.9656703881 -0.8847107602
C -4.4737743643 1.3929979660 -1.3074808350
C -5.4492700721 -1.2069778267 -1.0299525883
H -3.4013071635 -1.7763946201 -0.6643247272
C -5.8388354171 1.1328186078 -1.4491325597
H -4.0773946207 2.3965608566 -1.4167793905
C -6.3469028190 -0.1633253221 -1.3144226000
H -6.5170857219 1.9533453267 -1.6700389865
C -0.4708531829 2.0361949355 2.0111689106
H -0.5016363903 2.7501919428 1.1813736347
H -0.7099638654 2.6059660899 2.9194256992
C 0.9492896681 1.4430406745 2.1425464850
H 0.9025283633 0.5023204091 2.6993341936
H 1.5630111624 2.1192992944 2.7539102310
C 1.6672253831 1.2391307070 0.7826798823

H 0.9366986300 0.9113511669 0.0414415980
C 2.2686789076 2.5850939899 0.2864834655
H 3.3169467768 2.6637078964 0.6105476892
C 2.1616805488 2.8459896171 -1.2215022712
H 2.5538444251 3.8409425785 -1.4649288504
H 2.7199231897 2.1136080031 -1.8103359838
H 1.1144765655 2.8085567952 -1.5418048153
H -5.8257995774 -2.2217242442 -0.9226614330
C -7.8217437335 -0.4464231928 -1.4749695711
H -8.3916971651 0.4739101997 -1.6341404969
H -8.0077740402 -1.1071743561 -2.3309383781
H -8.2305963400 -0.9468709947 -0.5887767237
C -1.8581482907 0.1307027314 3.0462750827
H 3.5037324443 0.4573278952 1.5913780198
H -0.6361753595 -0.4591440212 0.6412258077
H -2.5370658808 1.6010150927 1.6448449991
H 1.7424885107 3.3971389819 0.8038017867
H -1.9776676766 0.7424716597 3.9476702170
H -1.0193326679 -0.5551591171 3.2075864657
H -2.7598274624 -0.4737139242 2.9095739794
68

C 2.8399905036 0.2551847651 0.1642227112
C 3.2952141716 -0.8321061613 -2.0911232414
H 2.2209952413 -0.9087649310 -2.2307233528
C 4.1672383454 -1.2981613904 -3.0749518055
H 3.7656259570 -1.7272935234 -3.9892550121
C 5.5493285932 -1.2177715946 -2.8887733999
H 6.2268047080 -1.5817545645 -3.6564887347
C 6.0545074791 -0.6713573403 -1.7085595996
H 7.1279810907 -0.6079045776 -1.5509015818
C 5.1807708479 -0.2090687018 -0.7225858337
H 5.5822667185 0.2123156302 0.1967033172
C 3.7939259242 -0.2797573890 -0.9026613890
N 1.7953037938 -0.7570717497 0.4717613042
C 1.9389034068 -1.7769036481 1.5733276453
C 0.9253359287 -2.9023385411 1.3219851592
C 1.6431499194 -1.0918467530 2.9236127307
C 3.3640796352 -2.3596618986 1.5690129266
H 1.0794884826 -3.6816384489 2.0760118066
H 1.0710321163 -3.3457247684 0.3322703172
H -0.1051617043 -2.5523458472 1.4017175253
H 0.6251032813 -0.6917935052 2.9434273216
H 2.3483427184 -0.2778865339 3.1289787644
H 1.7366961658 -1.8232423444 3.7341082109
H 4.1300951596 -1.6069068774 1.7777022474
H 3.5989548377 -2.8317404113 0.6106404158
H 3.4330489866 -3.1211676253 2.3531036738
O 0.7178830412 -0.7356627137 -0.2332924929
C -1.4997247300 2.0241539504 0.9962293197
N -1.4548352998 0.5888191206 1.3815338087
S -2.8652774136 -0.2641278284 1.6508626926
O -2.4324880183 -1.6261569504 1.9842460768
O -3.7008744240 0.5250830850 2.5608420823
C -3.7346871182 -0.3481262671 0.0763190603
C -3.2498080850 -1.1880551271 -0.9318312205
C -4.8716783042 0.4313364286 -0.1314414719
C -3.9141678615 -1.2371074676 -2.1529949908
H -2.3680784263 -1.7971260872 -0.7574481863
C -5.5268450653 0.3676823896 -1.3626224775
H -5.2399622339 1.0640064159 0.6689938909
C -5.0626435990 -0.4635149668 -2.3885604392
H -6.4156500756 0.9726905057 -1.5248646939
C -0.2878620653 2.2993633919 0.0867842736
H -0.3776395788 1.6482660532 -0.7900381171
H -0.3623199761 3.3305377012 -0.2811716027
C 1.0891726916 2.0675973952 0.7518143447
H 0.9810262191 1.3172965707 1.5394844833
H 1.4058432256 2.9826802678 1.2663522530
C 2.1983826695 1.6188825037 -0.2427016128
H 1.7417501606 1.4587519163 -1.2261940303
C 3.2914993280 2.6958122517 -0.4086442394
H 4.1000700321 2.3078576962 -1.0376848261
C 2.7746660766 3.9988769068 -1.0296796675
H 2.3536809064 3.8188173273 -2.0263546334
H 1.9944911778 4.4671249054 -0.4188079011
H 3.5869779884 4.7262589278 -1.1384576449
H -3.5392048946 -1.8902164877 -2.9376513093
C -5.7874521686 -0.5495313487 -3.7108218833
H -6.4853598958 0.2833739380 -3.8407238891
H -5.0864506935 -0.5400627175 -4.5531298559
H -6.3662023404 -1.4795978414 -3.7837106520
C -1.5521636694 2.9155484822 2.2404792153
H 3.4030708723 0.4067439584 1.0925158694
H -0.8121756154 0.0223385735 0.8171874196
H -2.4019453449 2.2120903442 0.3947115653
H 3.7388374421 2.9103206185 0.5736375499
H -2.4334917825 2.6792562290 2.8409789579
H -1.5989929499 3.9713583983 1.9462665319
H -0.6653320422 2.7666287219 2.8650915931
68

C 2.6971590563 0.0627268725 0.8460322079
C 2.5418946532 -0.6372032687 -1.5998545679
H 1.4657038305 -0.7193499067 -1.4854515076
C 3.1345986394 -0.9072173073 -2.8323928560
H 2.5118783518 -1.2056923207 -3.6714989128
C 4.5176445907 -0.7867480687 -2.9958275980
H 4.9743311923 -0.9954989544 -3.9596356489
C 5.3078020030 -0.3935678759 -1.9158881511
H 6.3838337039 -0.2934036726 -2.0321391844
C 4.7150745150 -0.1272967740 -0.6791896517
H 5.3363097355 0.1856191174 0.1576489007
C 3.3301121899 -0.2422128096 -0.5078503026
N 1.9713438234 -1.1413525588 1.3527614898
C 2.6404158018 -2.3286281493 1.9954110480
C 2.8212296869 -3.4257193694 0.9276993149
C 1.6959676281 -2.8214763302 3.1067005323
C 3.9950696137 -1.9428376527 2.6040977676
H 1.8583182568 -3.6574316282 0.4648944069
H 3.2168387752 -4.3364064180 1.3913777518
H 3.5154298476 -3.1060922989 0.1450563005
H 0.7124791401 -3.0576902266 2.6960030464
H 1.5750081418 -2.0580176085 3.8838163116
H 2.1108679330 -3.7214609977 3.5727920698
H 4.4057836276 -2.8171025690 3.1200449185
H 3.8963880536 -1.1400856336 3.3433661521
H 4.7210735385 -1.6406800613 1.8438676589
O 0.7797492243 -1.3537839906 0.9141119644
C -1.4877102736 1.3692059800 2.0930222421
N -1.5189972018 0.5545322624 0.8601848691
S -1.9665067884 1.2098243949 -0.6058999990
O -2.3001980758 2.6268270812 -0.4026431780
O -0.9607090895 0.7936404777 -1.5972213811
C -3.4944473063 0.3671064164 -1.0350770552
C -3.4429386624 -0.8982135387 -1.6240663991
C -4.7174442180 0.9826965878 -0.7724643061
C -4.6318801039 -1.5505234222 -1.9382610915
H -2.4837025517 -1.3562786797 -1.8416427877
C -5.8988950412 0.3150613291 -1.0950602285
H -4.7372890320 1.9749864805 -0.3347852383
C -5.8771271915 -0.9571332213 -1.6803464108
H -6.8537015828 0.7945174798 -0.8926515587
C -0.2224989984 2.2489687813 2.2475596800
H -0.2346242887 3.0013393864 1.4500217820
H -0.3401192072 2.8019177834 3.1895487077
C 1.1337741079 1.5099322577 2.2597637268
H 1.0210722406 0.5532905920 2.7793887129
H 1.8489414401 2.0893486709 2.8604981852
C 1.7536799157 1.2962418597 0.8524341859
H 0.9569932483 1.0987908909 0.1326807849
C 2.4823717101 2.5927099188 0.4040719590
H 3.5083449403 2.5948806049 0.8026401687
C 2.4999457249 2.8842465831 -1.1027460020
H 2.9065150398 3.8858541642 -1.2880233486
H 3.1077870754 2.1695864124 -1.6621636704
H 1.4849684421 2.8514311013 -1.5146636212
H -4.5937474123 -2.5360749090 -2.3966037374
C -7.1588777130 -1.6628545256 -2.0552285009
H -7.0923843626 -2.7404012410 -1.8691308009

H -8.0116141073 -1.2724881383 -1.4907283048
H -7.3841548660 -1.5321932751 -3.1219977403
C -1.7206021868 0.4257742431 3.2806936549
H 3.4985039306 0.2561357804 1.5642378346
H -0.7481808422 -0.1129906591 0.7551895119
H -2.3391300614 2.0528622548 2.0257479195
H 1.9782223242 3.4336723220 0.8970384745
H -2.6897028449 -0.0725979837 3.1829728613
H -1.7061609297 0.9842366971 4.2237204982
H -0.9512490175 -0.3521838647 3.3336026175

13-PBN

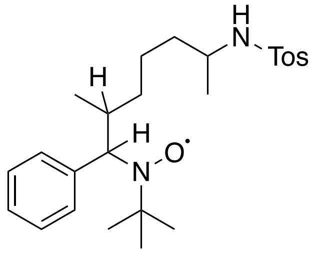

**Name**	**E(B3LYP)**	**H(B3LYP)**	**g-factor**	α_N	α_H
Tosyl_N_radical_2_heptyl_C6_radical_PBN_adduct_0077	-1708.198555	-1707.577265	2.00621523	14.884230	2.126830
Tosyl_N_radical_2_heptyl_C6_radical_PBN_adduct_0037	-1708.198373	-1707.576914	2.00618467	15.028900	2.022990
Tosyl_N_radical_2_heptyl_C6_radical_PBN_adduct_0122	-1708.198397	-1707.576821	2.00622080	15.043230	1.719150
Tosyl_N_radical_2_heptyl_C6_radical_PBN_adduct_0076	-1708.197888	-1707.576556	2.00619560	14.927490	2.160700
Tosyl_N_radical_2_heptyl_C6_radical_PBN_adduct_0232	-1708.197619	-1707.576182	2.00616713	14.898960	2.085380
Tosyl_N_radical_2_heptyl_C6_radical_PBN_adduct_0038	-1708.197412	-1707.575921	2.00621323	15.034110	1.925770
Tosyl_N_radical_2_heptyl_C6_radical_PBN_adduct_0140	-1708.197528	-1707.575916	2.00621453	15.070790	1.967930
Tosyl_N_radical_2_heptyl_C6_radical_PBN_adduct_0276	-1708.197344	-1707.575912	2.00622060	14.850900	2.243510
Boltzman averaged at 298.15K			2.0062	14.9351	2.1202

68

C -3.1166988550 -0.0558469190 -0.5273273086
C -3.8900815086 1.0059293291 1.6604747707
H -3.5632212406 0.1080048663 2.1753510038
C -4.4655187017 2.0572463728 2.3733965935
H -4.5677036084 1.9812128406 3.4529010740
C -4.9118030000 3.2025526296 1.7086193926
H -5.3594950074 4.0199739097 2.2675575005
C -4.7821496286 3.2880215116 0.3224747175
H -5.1278524508 4.1727205361 -0.2059649757
C -4.2084744787 2.2333577332 -0.3912775749
H -4.1070289961 2.3090548952 -1.4721272026
C -3.7533537681 1.0830630333 0.2660546341
N -3.8148987683 -1.3409347837 -0.2564377622
C -5.0933348174 -1.7747216637 -0.9215915153
C -6.2659697180 -1.4675839803 0.0303994173
C -4.9773499006 -3.2925916887 -1.1489410786
C -5.2949096684 -1.0627266399 -2.2656255562
H -6.3806825906 -0.3895924032 0.1808929601
H -6.0875603735 -1.9365671831 1.0016592836
H -7.2013767524 -1.8602764203 -0.3840959797
H -5.9131106880 -3.6811620377 -1.5649812547
H -4.7696900130 -3.8012019153 -0.2054130254
H -4.1670904075 -3.5200562435 -1.8509047463
H -4.4577142620 -1.2395376771 -2.9501926863
H -5.4361855864 0.0160975564 -2.1469547036
H -6.1981531823 -1.4606379759 -2.7401630952
O -3.5134314645 -1.9477238044 0.8361915623
C 2.8179167069 2.4514681482 -0.0456760321
N 3.5240155047 1.5700616002 -1.0060846450
S 4.9429998523 0.7798374292 -0.5424232722
O 5.6477950471 0.5248257559 -1.8043391435
O 5.6037646270 1.4801851238 0.5678839051
C 4.3292309797 -0.7727783512 0.1117185682
C 4.4483342332 -1.0413530103 1.4731535172
C 3.7627391348 -1.7094270901 -0.7584956612
C 3.9777875050 -2.2587950542 1.9695599523
H 4.9118863550 -0.3095019334 2.1257013208
C 3.2997708917 -2.9165355829 -0.2467150424
H 3.6979524879 -1.4953545118 -1.8203637025
C 3.3941331085 -3.2092977866 1.1248833319
H 2.8629698138 -3.6494938383 -0.9208895620
C 1.3146716882 2.4414586164 -0.3741993248
H 0.8224060624 3.1880656846 0.2641816490
H 1.1766053629 2.7889373481 -1.4095193215
C 0.6414165913 1.0766490516 -0.1883933602
H 0.7381003265 0.7688615635 0.8634340910
H 1.1878341763 0.3340176317 -0.7786091194
C -0.8450357476 1.0913906480 -0.5740800730
H -0.9381210801 1.3240554669 -1.6469066571
H -1.3356911564 1.9142976114 -0.0404628281
C -1.5909497822 -0.2255012951 -0.2720369929
H -1.4685640800 -0.4569918105 0.7926111834
C -1.0300124570 -1.4016677233 -1.0868038331
H -1.5465596431 -2.3320022556 -0.8399096170
H -1.1368013429 -1.2203445442 -2.1653773362
H 0.0345960417 -1.5493009802 -0.8804645910
H 4.0708134842 -2.4719262740 3.0314982727
C 2.8661449084 -4.5161332998 1.6664308319
H 3.2013762843 -4.6893914684 2.6934523311
H 1.7685713472 -4.5265748200 1.6692444645
H 3.1946870011 -5.3638305961 1.0543751619
C 3.4036219889 3.8684785072 -0.0262137695
H -3.2484722505 0.1556055171 -1.5918777558
H 3.6975036481 1.9995584374 -1.9140798910
H 2.9596032981 1.9981447131 0.9423622891
H 2.8864158958 4.4918424003 0.7121501051
H 3.2880408497 4.3495810736 -1.0066454764
H 4.4667614457 3.8402764280 0.2263802676

68

C -2.5230136051 -0.1459186708 -0.8771749382
C -3.1973038309 1.8267286279 0.5666213924
H -3.7484766535 2.2170076626 -0.2867035743
C -3.1834293953 2.5451252981 1.7642780745
H -3.7262197252 3.4839635716 1.8373226349
C -2.4756119809 2.0559905326 2.8619987883
H -2.4622596351 2.6110014813 3.7961339263
C -1.7841239889 0.8466469755 2.7524593636
H -1.2314250508 0.4592222713 3.6045547020
C -1.7966626887 0.1308319743 1.5557515518
H -1.2675776912 -0.8129889240 1.4768499540
C -2.5047013537 0.6150220071 0.4456912128
N -3.0154308655 -1.5383151240 -0.6823328335
C -4.4674562352 -1.9257220631 -0.5950884438
C -5.3660194174 -0.8849195643 -1.2756858760
C -4.8438945619 -2.0697145338 0.8926459227
C -4.6061117979 -3.2804770817 -1.3123373510
H -5.3423803890 0.0822580401 -0.7641180549

H -6.4000283633 -1.2445749199 -1.2476875564
H -5.0961057501 -0.7389362998 -2.3275868749
H -4.7770246663 -1.1086083335 1.4118062457
H -4.1665783304 -2.7756599309 1.3805406685
H -5.8697099862 -2.4433360206 0.9874262977
H -4.3725913132 -3.1823605599 -2.3785516128
H -5.6327789937 -3.6499834863 -1.2173218528
H -3.9238280684 -4.0122634152 -0.8753893869
O -2.1766817989 -2.3954193135 -0.2184747412
C 2.3170406799 2.6934393030 -0.9203865196
N 3.4359994767 1.7225767682 -0.9731429238
S 4.0596911047 1.0578306127 0.4498309590
O 5.4367365907 0.6865809349 0.1016878519
O 3.7807855714 1.9159079048 1.6103129103
C 3.0747171678 -0.4261218341 0.6497235648
C 3.1044885858 -1.4149396273 -0.3379293837
C 2.3249633161 -0.5991909894 1.8116644354
C 2.3506981160 -2.5712184608 -0.1640984864
H 3.7091576214 -1.2768846769 -1.2284223627
C 1.5816804307 -1.7693545268 1.9723603278
H 2.3303854407 0.1741079580 2.5719571946
C 1.5692637896 -2.7652397181 0.9871277175
H 0.9998370901 -1.9099748249 2.8801966750
C 1.5482868417 2.6116818906 -2.2538621175
H 0.8450134903 3.4546997064 -2.3002723258
H 2.2675596571 2.7730809033 -3.0694604180
C 0.7927821884 1.2902400527 -2.4757039390
H 1.4363480169 0.4600804592 -2.1576991724
H 0.6224881197 1.1545308138 -3.5503661697
C -0.5519727223 1.2312839435 -1.7293642657
H -1.2654694280 1.9059330611 -2.2286029393
H -0.4209265884 1.6296691700 -0.7165977171
C -1.1582103072 -0.1829582909 -1.6226909772
H -0.4799905079 -0.8004781689 -1.0231520743
C -1.3259055451 -0.8501839319 -2.9967589227
H -1.9782728485 -0.2540173376 -3.6499531658
H -0.3614382865 -0.9577026666 -3.5023122922
H -1.7592038936 -1.8491981506 -2.8981939549
H 2.3665818984 -3.3394854017 -0.9331428183
C 0.7110721348 -3.9970153325 1.1388150900
H -0.2822096794 -3.8220556798 0.7068150980
H 1.1516412596 -4.8579926382 0.6251357003
H 0.5734878852 -4.2617135180 2.1925023459
C 2.7971444738 4.1172690964 -0.6138567396
H -3.2397980454 0.3484105797 -1.5380330714
H 4.2290514248 2.0384305179 -1.5304078588
H 1.6632348668 2.3597557146 -0.1067914054
H 3.3448704092 4.1424392661 0.3315506574
H 1.9467996399 4.8051982733 -0.5426853602
H 3.4564371904 4.4844159377 -1.4118566472
68

C -2.9180062452 0.4882419305 0.5511298569
C -1.7883375062 0.8977762869 -1.6871820083
H -1.0013532909 0.2094398945 -1.3863921656
C -1.7208292853 1.5143820612 -2.9395563202
H -0.8860871672 1.2964266098 -3.6013213687
C -2.7217305208 2.3987396447 -3.3415472907
H -2.6713748894 2.8770825197 -4.3163565647
C -3.7940730795 2.6604705871 -2.4845268035
H -4.5827415149 3.3422641375 -2.7928333481
C -3.8627121014 2.0479290336 -1.2336784726
H -4.7090082408 2.2325669094 -0.5796373058
C -2.8570444777 1.1610342215 -0.8188819422
N -4.1755895668 -0.2939011995 0.7022479282
C -4.3711737578 -1.6935183442 0.1844349244
C -3.0339918812 -2.4352923953 0.0570813213
C -5.0764516532 -1.6118440800 -1.1838024468
C -5.2705941411 -2.4166731110 1.2028170544
H -2.4932439993 -2.4667770754 1.0093786216
H -2.3805432680 -1.9972580883 -0.7033102672
H -3.2355769745 -3.4694948330 -0.2421292069
H -6.0021745327 -1.0374032565 -1.0905704276
H -5.3206627293 -2.6180571796 -1.5432591398
H -4.4385868250 -1.1251866754 -1.9281901249
H -5.5082846610 -3.4230638340 0.8415196265
H -6.1996043778 -1.8623895618 1.3496165366
H -4.7652387509 -2.5083623231 2.1708342674
O -5.2470694777 0.3693646160 0.9570721064
C 2.3894714943 1.9249843822 1.4956917163
N 2.5517580214 1.0646179203 0.2974012806
S 2.2598603358 -0.5922335257 0.3385222935
O 2.1242138729 -0.9861618633 1.7451699870
O 1.2097416078 -0.8896426492 -0.6503968830
C 3.7767779101 -1.3164947887 -0.2864925473
C 4.8317182749 -1.5624789548 0.5949435750
C 3.8885506824 -1.6256046618 -1.6416549323
C 6.0120141606 -2.1123654273 0.1027532120
H 4.7200647155 -1.3383103729 1.6505821588
C 5.0788333288 -2.1752224650 -2.1165375010
H 3.0502418871 -1.4508602528 -2.3074791346
C 6.1562777416 -2.4268181102 -1.2572262769
H 5.1686504678 -2.4179473147 -3.1725198088
C 1.0069211368 2.6029240820 1.5440735721
H 1.0013825778 3.2968189282 2.3963321804
H 0.8930037860 3.2313667505 0.6461415776
C -0.1830912714 1.6423547141 1.6567252166
H -0.0815085293 1.0525281036 2.5761228141
H -0.1416200727 0.9266239435 0.8290476715
C -1.5338317218 2.3735118276 1.6307925892
H -1.5978710971 2.9672806006 0.7100100938
H -1.5679400808 3.0943439296 2.4604160695
C -2.7901347203 1.4797854289 1.7426586383
H -3.6606060150 2.1429740790 1.6895394091
C -2.8550398433 0.7413022159 3.0896425236
H -2.0832081642 -0.0337825870 3.1713894679
H -2.7059056162 1.4455875110 3.9169966499
H -3.8296554947 0.2660308473 3.2252272587
H 6.8346931440 -2.3065454943 0.7867933393
C 7.4310017508 -3.0516227166 -1.7721489585
H 8.3111538255 -2.6430705239 -1.2641650074
H 7.4335979243 -4.1364821761 -1.6027451502
H 7.5520288829 -2.8883198449 -2.8476300580
C 3.5297608614 2.9501709346 1.5176390312
H -2.0982978336 -0.2290272750 0.6153212150
H 2.1601998488 1.4553484837 -0.5566605794
H 2.4904859429 1.2572058777 2.3561736350
H 3.4746761148 3.5660538712 2.4224535538
H 3.4735991699 3.6212620194 0.6516093680
H 4.5014792097 2.4480594147 1.4945637162
68

C -2.8084870805 -0.1052585422 -0.6925920105
C -3.0412865974 0.4999889875 1.7758958701
H -2.5227708454 -0.4218813125 2.0195585261
C -3.4794406890 1.3506179326 2.7903546980
H -3.2815909039 1.0950641040 3.8281848854
C -4.1708238951 2.5246553063 2.4800354994
H -4.5101944518 3.1854773742 3.2731888221
C -4.4253994508 2.8409646002 1.1456538151
H -4.9641828492 3.7502021999 0.8919334231
C -3.9893816378 1.9865699724 0.1303676969
H -4.1884049225 2.2418662699 -0.9086195158
C -3.2908119291 0.8098414210 0.4304097191
N -3.3083906363 -1.4939466008 -0.4919329135
C -4.6823459107 -1.9713276430 -0.8789482608
C -5.2983501866 -1.0804455589 -1.9655836313
C -5.5699039225 -1.9726560117 0.3811832352
C -4.5160065336 -3.4050288886 -1.4137552117
H -5.4811962151 -0.0609486267 -1.6120459373
H -6.2651350540 -1.5039020370 -2.2576377333
H -4.6716548980 -1.0397234268 -2.8635113542
H -5.7217541763 -0.9565821192 0.7585266725
H -5.0979624716 -2.5680600754 1.1671855457
H -6.5497879745 -2.4060850026 0.1513022499
H -5.4969330840 -3.8335045636 -1.6457419084
H -4.0222026484 -4.0320316049 -0.6686987769
H -3.9118609563 -3.4116989910 -2.3280114812
O -2.6769910062 -2.2277294519 0.3552280409
C 2.9460678107 2.7385009872 -0.5004619940

N 3.7519840217 1.6602634496 -1.1210930373
S 4.7979325307 0.7238796830 -0.1833985190
O 5.7948103123 0.2215746698 -1.1361502851
O 5.1977512635 1.4489829668 1.0303703958
C 3.7429956579 -0.6301078650 0.3342967539
C 3.4046830065 -1.6250367966 -0.5864455385
C 3.2258583918 -0.6482436814 1.6286839097
C 2.5098945863 -2.6220901182 -0.2097227145
H 3.8399143005 -1.6142858416 -1.5805695341
C 2.3279637223 -1.6544432908 1.9879630946
H 3.5270822980 0.1146633525 2.3385981445
C 1.9383939596 -2.6417298188 1.0735054928
H 1.9183775443 -1.6675177301 2.9950230649
C 1.4956980750 2.6745506025 -1.0160911654
H 0.9363495911 3.5012931571 -0.5552823806
H 1.4870292531 2.8611974968 -2.1002389147
C 0.8118651048 1.3388834343 -0.7067126107
H 0.9009002604 1.1326219299 0.3694584038
H 1.3674552115 0.5422864261 -1.2116695399
C -0.6744887031 1.2741940337 -1.0858394840
H -0.8093875425 1.5636054183 -2.1394408584
H -1.2208708883 2.0149986465 -0.4903644961
C -1.2643526732 -0.1378121566 -0.8619779945
H -0.8557229479 -0.5332949368 0.0748190894
C -0.8639040658 -1.0898412139 -2.0008306114
H -1.3205064540 -0.7780984069 -2.9502059311
H 0.2214995915 -1.0924149599 -2.1418295245
H -1.1740537549 -2.1160459440 -1.7899364593
H 2.2423850613 -3.3966092774 -0.9243557068
C 0.9047979418 -3.6789162892 1.4413922748
H -0.0980103968 -3.3514886332 1.1351090076
H 1.1023824630 -4.6354847981 0.9459211579
H 0.8783999043 -3.8533579196 2.5218788359
C 3.5980600461 4.1080685209 -0.7161045084
H -3.2391470828 0.2511793078 -1.6319801720
H 4.2470196730 1.9262971956 -1.9699730751
H 2.9350696713 2.5319681579 0.5753677693
H 4.6025168409 4.1261970119 -0.2823304507
H 3.0043069102 4.9004651317 -0.2468018103
H 3.6751096982 4.3416337578 -1.7864181158
68

C -2.5597215641 -0.1362157459 -0.9164661879
C -3.2102356198 2.0394083467 0.2100769855
H -3.6893904543 2.3348750393 -0.7212141946
C -3.2205128208 2.9150034677 1.2981341394
H -3.7111210713 3.8807893709 1.2088449071
C -2.6042071847 2.5488906242 2.4945451948
H -2.6081211407 3.2279241060 3.3427428972
C -1.9840785634 1.3010201996 2.5963078721
H -1.5056808918 1.0060093618 3.5263389210
C -1.9741254563 0.4276680981 1.5086679374
H -1.5148996075 -0.5514907735 1.5985552638
C -2.5827177328 0.7898989020 0.2973081687
N -3.0436588230 -1.4974762109 -0.5580632847
C -4.4934219541 -1.8932085782 -0.4705070129
C -5.3838954384 -0.9398890740 -1.2779882066
C -4.9095384592 -1.8868344895 1.0135486184
C -4.5977304316 -3.3170485122 -1.0458596544
H -5.3757068793 0.0773599180 -0.8741203180
H -6.4160834516 -1.3027253295 -1.2308979598
H -5.0943483864 -0.9068696976 -2.3342595944
H -4.8645702601 -0.8764007637 1.4316654110
H -4.2405610108 -2.5311288620 1.5900163360
H -5.9346449808 -2.2594255233 1.1190118353
H -4.3361118398 -3.3272800919 -2.1100349434
H -5.6223186280 -3.6893302585 -0.9396527916
H -3.9193326119 -3.9894071230 -0.5168250590
O -2.2112166873 -2.2835273335 0.0289689592
C 2.2099813768 2.6998009289 -1.0189914069
N 3.2856037526 1.7123820493 -0.7700558441
S 3.6367943579 1.1848871957 0.7976832503
O 5.0997073127 1.2245235785 0.9203189901
O 2.7578424452 1.8928851709 1.7363899959
C 3.1389696247 -0.5379746329 0.7919864726

C 4.0882402108 -1.5261235464 0.5326344719
C 1.8011583925 -0.8704569850 1.0165246645
C 3.6837160138 -2.8598327512 0.4878224223
H 5.1258728032 -1.2502131559 0.3780537326
C 1.4112447079 -2.2065809115 0.9537345852
H 1.0776521092 -0.0940232509 1.2429471715
C 2.3448523956 -3.2205580169 0.6898414395
H 0.3643748769 -2.4636058624 1.0948401913
C 1.6015570295 2.4252342038 -2.4085279148
H 0.9293058950 3.2572166556 -2.6610744311
H 2.4119436844 2.4539539214 -3.1507313424
C 0.8430260633 1.0914421448 -2.5164369017
H 1.4480863518 0.3069173668 -2.0437804205
H 0.7551785174 0.8176256544 -3.5746422008
C -0.5559985730 1.1353921959 -1.8744838007
H -1.2262892687 1.7312411089 -2.5139749831
H -0.4974955813 1.6720802608 -0.9209018719
C -1.1747126655 -0.2533553232 -1.6173021724
H -0.5152017173 -0.7951927842 -0.9310779676
C -1.3030857166 -1.0787378460 -2.9069399964
H -1.9396894741 -0.5674188381 -3.6424212374
H -0.3245508196 -1.2378267351 -3.3702073192
H -1.7347957657 -2.0626730666 -2.7039045884
H 4.4220719533 -3.6329284250 0.2886486684
C 1.9144926012 -4.6675501495 0.6517586620
H 1.8946856146 -5.0987776558 1.6615117772
H 0.9070268795 -4.7717769641 0.2357021328
H 2.5993189489 -5.2746566680 0.0508621032
C 2.7131699378 4.1411125891 -0.8746025219
H -3.2626183970 0.2616496314 -1.6529128649
H 4.1658409430 1.9312645689 -1.2324287403
H 1.4544132991 2.5215577353 -0.2486192824
H 3.1163115905 4.3080341623 0.1285043911
H 1.8983321433 4.8550309952 -1.0411772070
H 3.5012917876 4.3577144025 -1.6080887290
68

C -3.1787057813 0.1368118387 0.5495557133
C -1.6412742585 1.0443959022 -1.2563184531
H -0.9955987113 0.1817586535 -1.1133576667
C -1.2827740879 2.0035426299 -2.2064747142
H -0.3699123859 1.8658442261 -2.7798506501
C -2.0944019747 3.1190606458 -2.4126168354
H -1.8214091539 3.8664212669 -3.1533680985
C -3.2663813363 3.2667426799 -1.6650376750
H -3.9085787138 4.1291946275 -1.8256301489
C -3.6223005287 2.3110414779 -0.7136322211
H -4.5444455287 2.4160392444 -0.1504930948
C -2.8092964229 1.1878060498 -0.4953827982
N -4.5269607725 -0.4346346963 0.2775988336
C -4.7996552116 -1.5304130320 -0.7175380652
C -5.2871621980 -0.8854001323 -2.0298840889
C -5.9106778488 -2.4066525959 -0.1116814684
C -3.5487020575 -2.3817992172 -0.9710441470
H -4.4984467997 -0.2817365165 -2.4894386301
H -6.1465137547 -0.2399591963 -1.8290578520
H -5.5878185133 -1.6616016210 -2.7428607222
H -5.5628298012 -2.8965697781 0.8048551866
H -6.2056884212 -3.1825850092 -0.8263610261
H -6.7830354462 -1.7972228344 0.1323994329
H -2.7495035625 -1.8157176452 -1.4589229381
H -3.8175524083 -3.2076806059 -1.6382769726
H -3.1587327481 -2.8195803651 -0.0453989500
O -5.5422428358 0.2708678737 0.6304731561
C 2.0193737976 0.6807299038 2.0012286534
N 1.9485332383 0.6504068579 0.5288930740
S 2.2775180696 -0.6728744480 -0.4210867579
O 1.7674687509 -1.8405200222 0.3029297522
O 1.7982085702 -0.3044745622 -1.7585135641
C 4.0612340362 -0.8659028616 -0.5406116326
C 4.7300309542 -1.6920094314 0.3647716727
C 4.7657843916 -0.1695941953 -1.5255605971
C 6.1172790919 -1.8058624715 0.2863024691
H 4.1660320589 -2.2503068522 1.1044753606
C 6.1512946894 -0.2965175658 -1.5897968019

H 4.2300995121 0.4453439860 -2.2413891884
C 6.8493997030 -1.1128251002 -0.6875860944
H 6.7002704379 0.2423233435 -2.3583761764
C 0.7362385766 1.3469629565 2.5358750186
H 0.8495311702 1.5158706466 3.6153458375
H 0.6534882435 2.3447697521 2.0789578005
C -0.5357303131 0.5359596856 2.2503531192
H -0.5803420811 -0.3172454732 2.9399127885
H -0.4486346847 0.1139444372 1.2443194208
C -1.8150039840 1.3795325765 2.3515570789
H -1.7103428755 2.2502194396 1.6920666600
H -1.9062799076 1.7801470848 3.3716392289
C -3.1391455263 0.6579760244 2.0150694359
H -3.9388228120 1.4019864459 2.0986166067
C -3.4539549985 -0.4706381172 3.0102839510
H -3.3860390491 -0.1004080263 4.0403299443
H -4.4664357693 -0.8533396191 2.8580280507
H -2.7541040561 -1.3100233912 2.9132772245
H 6.6388924730 -2.4512668255 0.9889050170
C 8.3480899471 -1.2686135211 -0.7876478357
H 8.6138037661 -2.0623860086 -1.4982875654
H 8.8246933238 -0.3471297011 -1.1381346895
H 8.7893273353 -1.5349226575 0.1781316893
C 3.2929559519 1.3764914121 2.4979914566
H -2.4707083549 -0.6893967187 0.4635539247
H 2.0589535648 1.5305479099 0.0349155154
H 2.0258050166 -0.3676931994 2.3172368350
H 4.1905656166 0.8874274955 2.1064494519
H 3.3408853505 1.3573971532 3.5925861972
H 3.3115954802 2.4281546292 2.1840336980
68

C -3.1544362736 -0.0369833365 0.4486489121
C -1.5892574342 1.2192671634 -1.1029321792
H -0.9467325613 0.3422094193 -1.1198947667
C -1.2113005538 2.3527724524 -1.8265482598
H -0.2772634486 2.3398194455 -2.3812341784
C -2.0268072618 3.4844773201 -1.8215365554
H -1.7394165184 4.3687391004 -2.3848562230
C -3.2191537187 3.4744212159 -1.0916150128
H -3.8634138539 4.3502688093 -1.0906505548
C -3.5913187182 2.3453365200 -0.3622372280
H -4.5269118777 2.3323136801 0.1883497031
C -2.7748935267 1.2037674530 -0.3562804546
N -4.4907154805 -0.5526336236 0.0378594822
C -4.7304875846 -1.4208534943 -1.1678814341
C -5.1964518315 -0.5224759600 -2.3305723903
C -5.8450159745 -2.4097607410 -0.7816594131
C -3.4654068408 -2.1943032607 -1.5619908031
H -6.0667962937 0.0625127399 -2.0212304628
H -5.4735065697 -1.1371421404 -3.1947172513
H -4.4046729872 0.1677118486 -2.6375921820
H -6.7288737999 -1.8688646687 -0.4378704698
H -5.5120457849 -3.0758324301 0.0224639305
H -6.1161816891 -3.0235903257 -1.6474562736
H -3.7108800223 -2.8664320011 -2.3912449091
H -3.0913068619 -2.8110208858 -0.7371493503
H -2.6618572991 -1.5348372974 -1.9031238700
O -5.5213649013 0.0580745455 0.5047962993
C 2.0076129878 0.3349242306 2.1299525999
N 2.0331373466 0.8568528391 0.7478725388
S 2.3125564594 -0.0691791579 -0.6064311267
O 1.4688862868 -1.2623798909 -0.4825708521
O 2.1878416916 0.8648179066 -1.7308883903
C 4.0184274448 -0.6360928136 -0.5702882593
C 5.0292297235 0.1933322553 -1.0638224282
C 4.3228645651 -1.8867155102 -0.0301820146
C 6.3524052312 -0.2353144618 -1.0015124277
H 4.7757264531 1.1504056582 -1.5082534145
C 5.6539957013 -2.2987559491 0.0253778308
H 3.5242135709 -2.5317290205 0.3202808358
C 6.6874017485 -1.4843036988 -0.4563530061
H 5.8921242998 -3.2739341981 0.4431126297
C 0.6918021135 0.7753023147 2.8014298030
H 0.7619477381 0.5876903251 3.8816735598
H 0.5991043886 1.8649044621 2.6841208456
C -0.5450736674 0.0764749936 2.2177747541
H -0.5990150708 -0.9443845900 2.6193449694
H -0.4016108241 -0.0245746443 1.1382048889
C -1.8488441608 0.8375064875 2.4973658051
H -1.7634139698 1.8400608974 2.0596258768
H -1.9580334073 0.9867958425 3.5816277564
C -3.1515231643 0.1790842941 1.9891062783
H -3.9673120577 0.8779030346 2.2037687271
C -3.4640566258 -1.1322668025 2.7284327519
H -2.7431589693 -1.9228606931 2.4850878144
H -3.4303261784 -0.9758764416 3.8133302792
H -4.4633134106 -1.4944334145 2.4731972298
H 7.1392786452 0.4072961336 -1.3892542597
C 8.1231857352 -1.9509421648 -0.4239814826
H 8.4125919036 -2.3975673264 -1.3843722003
H 8.8107547040 -1.1199923791 -0.2335811923
H 8.2816442839 -2.7088449529 0.3496435748
C 3.2494112470 0.7789669112 2.9120980619
H -2.4346048525 -0.8239385759 0.2157797458
H 2.3444861496 1.8158046901 0.6253296109
H 2.0039423028 -0.7573362604 2.0437327036
H 3.2774758054 1.8720001863 3.0114590960
H 4.1689005595 0.4560485266 2.4133126770
H 3.2400957681 0.3571756494 3.9230871039
68

C -2.3578556384 -0.1112235360 0.7019947451
C -1.9756268746 1.3621263542 -1.3318148450
H -1.0075053886 0.8817446539 -1.4584984459
C -2.3685189182 2.3636719303 -2.2228342061
H -1.7082219200 2.6513457962 -3.0369630448
C -3.6061200321 2.9881153809 -2.0686682942
H -3.9163297473 3.7661216889 -2.7611190638
C -4.4464316985 2.6035041516 -1.0203846088
H -5.4149164169 3.0813902388 -0.8971283023
C -4.0523885927 1.6059698252 -0.1291680290
H -4.7151012382 1.2937683717 0.6719152994
C -2.8078528032 0.9738120173 -0.2737041307
N -3.3622096639 -1.2075423855 0.7777429068
C -3.4746873209 -2.3323208323 -0.2153301286
C -4.6191742927 -2.0044532257 -1.1942232723
C -3.8118038129 -3.5988526776 0.5918133685
C -2.1612019599 -2.5379654930 -0.9810933212
H -4.7899568235 -2.8484456403 -1.8721224829
H -4.3831591759 -1.1206879376 -1.7952063576
H -5.5388816048 -1.8093361816 -0.6366546994
H -2.9889566273 -3.8606052758 1.2667253297
H -3.9837558054 -4.4414686990 -0.0867025162
H -4.7101746376 -3.4361130845 1.1906240047
H -2.2750625811 -3.4054149394 -1.6404162323
H -1.3215510381 -2.7405110675 -0.3066785765
H -1.9098711310 -1.6784931133 -1.6103602450
O -4.3962248299 -1.0048307692 1.5140304045
C 2.6950893209 2.3104888450 1.2516547432
N 2.6668373699 1.9241865513 -0.1800816393
S 3.9133407830 0.9890167597 -0.8330601225
O 3.9454747554 1.3575663710 -2.2534429843
O 5.1173340529 1.0619244751 0.0054338053
C 3.2863925419 -0.6859978312 -0.6930412104
C 3.7788201797 -1.5263055362 0.3037334476
C 2.3262460078 -1.1375310084 -1.6025422422
C 3.2891095851 -2.8299382520 0.3973814125
H 4.5433128843 -1.1647726455 0.9828995925
C 1.8523860758 -2.4414983906 -1.4969767149
H 1.9731240435 -0.4790187696 -2.3895465046
C 2.3240407921 -3.3081825436 -0.4968776165
H 1.1111635602 -2.7988207641 -2.2078959715
C 1.2514499222 2.5436563175 1.7291868758
H 1.2950360866 2.9342522002 2.7553520952
H 0.8020162751 3.3428523829 1.1205048884
C 0.3573990988 1.2988896016 1.6894249623
H 0.7976857044 0.5233084567 2.3308318628
H 0.3657421824 0.8969138458 0.6698868786
C -1.0795940747 1.6034475880 2.1403464167

H -1.4833109718 2.4121110534 1.5183654745
H -1.0498699560 1.9979987188 3.1661853966
C -2.0727300990 0.4191302933 2.1357420439
H -3.0259387468 0.8014339903 2.5168661961
C -1.6241031190 -0.7157040224 3.0708944778
H -1.3628909167 -0.3141633732 4.0573262614
H -2.4246131376 -1.4467359928 3.2071257644
H -0.7424031104 -1.2418912418 2.6843366608
H 3.6713073721 -3.4873725075 1.1743391233
C 1.8239597360 -4.7308808320 -0.4147099274
H 2.3108772442 -5.3626048531 -1.1691127874
H 2.0292455939 -5.1725404916 0.5652774789
H 0.7447390631 -4.7879635693 -0.5947401576
C 3.5932441577 3.5273064059 1.5069239328
H -1.4394414393 -0.5581074370 0.3158628230
H 2.5098756724 2.7059199907 -0.8152175232
H 3.1057500742 1.4481962829 1.7896511311
H 3.6176395324 3.7722858942 2.5749791007
H 3.2131123277 4.4080921528 0.9725951236
H 4.6145531781 3.3287934645 1.1726236323
68

C -3.5936803680 -0.3869443360 -0.9982160196
C -2.8976575645 0.6020940849 1.2489213990
H -3.6605012656 -0.0095844835 1.7206355610
C -2.0681893588 1.4174589957 2.0188269435
H -2.1878965428 1.4372789379 3.0992261084
C -1.0817042999 2.1995887892 1.4097164022
H -0.4248633518 2.8233776472 2.0095782143
C -0.9463229760 2.1818218570 0.0197856075
H -0.1742540409 2.7767103549 -0.4574021164
C -1.7882844876 1.3754763165 -0.7504472803
H -1.6716152143 1.3606497271 -1.8320889010
C -2.7566546157 0.5626880758 -0.1468583489
N -5.0371610441 -0.2742423231 -0.6651553048
C -5.9560711645 0.7910335722 -1.1999156794
C -7.2999254632 0.1027220940 -1.4992074575
C -5.3968206433 1.4224606123 -2.4814676245
C -6.1405104177 1.8655414973 -0.1103537441
H -8.0382969905 0.8453161723 -1.8204363964
H -7.6711189555 -0.4041122624 -0.6060455772
H -7.1876803617 -0.6396330742 -2.2975224559
H -6.1223108732 2.1519979760 -2.8567159041
H -5.2415808201 0.6775932866 -3.2699863150
H -4.4572755618 1.9556825045 -2.3053448225
H -6.8804289822 2.6066203566 -0.4331984202
H -5.1991262577 2.3845914426 0.0954858727
H -6.4899809472 1.3998042143 0.8148291728
O -5.4504717343 -0.9080030267 0.3748202054
C 1.8785720076 -1.8106365383 0.4801019373
N 1.9660036429 -0.3621948461 0.7919365043
S 2.8654815988 0.6505586404 -0.2058810678
O 2.8604602324 0.2019392703 -1.6067662629
O 2.4170300879 2.0075604790 0.1359406919
C 4.5310878340 0.4004388174 0.4126995220
C 4.8246407604 0.6896330990 1.7492143278
C 5.5277329770 -0.0245237546 -0.4625131735
C 6.1291828748 0.5382054865 2.2045416666
H 4.0381482941 1.0224363328 2.4184865434
C 6.8328024746 -0.1711389006 0.0122150945
H 5.2775072322 -0.2357218655 -1.4964585692
C 7.1543269077 0.1068515005 1.3450764719
H 7.6120070872 -0.5062516740 -0.6677610002
C 0.7972416201 -2.1518511668 -0.5683480087
H 0.9942795775 -1.5398785972 -1.4558779259
H 0.9393152217 -3.1985856808 -0.8736696808
C -0.6544798088 -1.9495677494 -0.1071491128
H -0.7630498228 -0.9723876346 0.3738596113
H -0.9164431405 -2.6956755988 0.6552616534
C -1.6520815890 -2.0602449537 -1.2720371995
H -1.5316002195 -3.0544975189 -1.7237236086
H -1.3696792646 -1.3469474144 -2.0600692057
C -3.1495615701 -1.8782880897 -0.9086787965
H -3.3101892380 -2.2023932624 0.1255530168
C -4.0274392595 -2.7505241685 -1.8217290013
H -3.9076668675 -2.4658517669 -2.8760605744
H -3.7372996426 -3.8035043109 -1.7313903481
H -5.0846859241 -2.6693765494 -1.5587126608
H 6.3604416628 0.7586688080 3.2440926055
C 8.5681145766 -0.0395426247 1.8552077661
H 9.2201576796 -0.4963986310 1.1047625136
H 8.9958633447 0.9359682700 2.1191119676
H 8.6042880403 -0.6606854850 2.7580011865
C 1.7207535571 -2.5742608784 1.8001880847
H -3.4876495099 -0.0865910853 -2.0439843534
H 1.0765892577 0.0950363758 0.9925091675
H 2.8507318019 -2.0765777966 0.0492749716
H 1.6620441203 -3.6528463405 1.6135873618
H 0.8090106067 -2.2751848932 2.3289115563
H 2.5720509834 -2.3779841086 2.4586983811
68

C -2.8517817658 0.2685824315 -0.9328258161
C -1.8538193496 1.0831205523 1.2586938935
H -1.9916555168 2.1029474971 0.9058056669
C -1.2797059041 0.8626620327 2.5126797034
H -0.9702509438 1.7081999467 3.1200624892
C -1.1012356723 -0.4400329415 2.9775466015
H -0.6608179806 -0.6154678977 3.9552560037
C -1.4908090223 -1.5174926678 2.1762748555
H -1.3657403935 -2.5359879198 2.5363162814
C -2.0469012808 -1.2966805469 0.9160183664
H -2.3626666054 -2.1327193992 0.3000071160
C -2.2392130134 0.0109268723 0.4434585803
N -4.1130147675 -0.4971891791 -1.0990708742
C -5.4579202126 -0.0519246088 -0.5907202422
C -6.4853555919 -0.4393329313 -1.6693823962
C -5.4956997804 1.4637674435 -0.3559761333
C -5.7549844801 -0.8041868394 0.7213163766
H -6.3033022617 0.1148358273 -2.5970613502
H -7.4982880902 -0.2082827863 -1.3223960218
H -6.4193374994 -1.5073336463 -1.8869633859
H -5.2675634372 2.0243716816 -1.2694503211
H -4.8083279664 1.7782206544 0.4357184108
H -6.5069478857 1.7436562518 -0.0422230755
H -5.0494867212 -0.5193242959 1.5081480983
H -5.6753867621 -1.8820061624 0.5571683590
H -6.7693280312 -0.5741247671 1.0664615961
O -4.0042147651 -1.7450136975 -1.3901401800
C 2.2615584530 2.1183540753 -1.8543630946
N 2.9250444106 1.0211018777 -1.1029770102
S 3.3025738055 1.3106181867 0.5292050857
O 4.6269109065 1.9373566596 0.6712735232
O 2.1166983691 1.9556434961 1.1057778766
C 3.4380650783 -0.3671094119 1.1416393036
C 2.2844100534 -1.1429744609 1.2891978176
C 4.6915278906 -0.8677251214 1.4876707266
C 2.4030853663 -2.4396590389 1.7763980262
H 1.3118096486 -0.7356567291 1.0356220822
C 4.7897227925 -2.1723575175 1.9746033267
H 5.5693836768 -0.2393535710 1.3823728883
C 3.6540532301 -2.9763364981 2.1247824903
H 5.7655885338 -2.5678890699 2.2450140930
C 1.6104367654 1.5181320328 -3.1128463067
H 1.1655598462 2.3493565764 -3.6778897600
H 2.4034731747 1.1078877445 -3.7563392475
C 0.5431963200 0.4388872906 -2.8738775967
H 0.9985086100 -0.4176840984 -2.3620622507
H 0.2146906353 0.0760272061 -3.8561130128
C -0.6674030485 0.9246303566 -2.0594330027
H -0.9862181309 1.9130090149 -2.4277942531
H -0.3519843310 1.0707718705 -1.0207869744
C -1.8809405596 -0.0321546259 -2.1132355568
H -1.5260487052 -1.0621939459 -1.9877894793
C -2.6039655619 0.0733601847 -3.4658686232
H -3.0746231664 1.0592784134 -3.5817469083
H -1.8998373708 -0.0518684033 -4.2952624016
H -3.3756972470 -0.6924853419 -3.5633473006
H 1.5073336209 -3.0449635735 1.8924804935
C 3.7592493894 -4.3838618245 2.6618673338

H 3.2756840827 -4.4698807948 3.6432085053
H 3.2652301535 -5.1020190028 1.9967774193
H 4.8025049838 -4.6928231752 2.7771402086
C 3.2182164862 3.2667719505 -2.2040136542
H -3.1170066374 1.3273658765 -0.9954715783
H 3.7721155023 0.6985401473 -1.5738599793
H 1.4812600678 2.4944761775 -1.1875206421
H 4.0230957143 2.9174344533 -2.8642347901
H 3.6781328982 3.6886453037 -1.3058537292
H 2.6826848891 4.0677663134 -2.7267643420

14-PBN

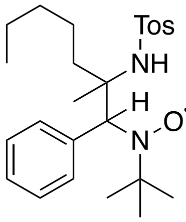

Name	E(B3LYP)	H(B3LYP)	g-factor	α_N	α_H
Tosyl_N_radical_2_heptyl_C2_radical_PBN_adduct_ 0002	-1708.195891	-1707.574403	2.00609403	14.529060	4.453180
Tosyl_N_radical_2_heptyl_C2_radical_PBN_adduct_0045	-1708.194636	-1707.573096	2.00609443	14.528040	4.393290
Tosyl_N_radical_2_heptyl_C2_radical_PBN_adduct_0085	-1708.194617	-1707.573044	2.00609463	14.531110	4.401040
Tosyl_N_radical_2_heptyl_C2_radical_PBN_adduct_0018	-1708.194494	-1707.572968	2.00609447	14.532690	4.423620
Tosyl_N_radical_2_heptyl_C2_radical_PBN_adduct_0012	-1708.194347	-1707.572820	2.00609393	14.536730	4.410310
Tosyl_N_radical_2_heptyl_C2_radical_PBN_adduct_0058	-1708.193368	-1707.571750	2.00609413	14.528010	4.409230
Tosyl_N_radical_2_heptyl_C2_radical_PBN_adduct_0210	-1708.192999	-1707.571628	2.00604217	14.903040	2.286660
Tosyl_N_radical_2_heptyl_C2_radical_PBN_adduct_0015	-1708.193222	-1707.571557	2.00609417	14.531210	4.424490
Boltzman averaged for 298.15 K			2.006088	14.5480	4.3230

68

C 1.6928586549 -0.4492678124 0.3234125649
C 3.7633838761 1.0601574238 0.3992462188
H 3.6496645170 1.2351000601 -0.6658863414
C 4.8134087633 1.6547903465 1.0986358423
H 5.5102500826 2.3012763900 0.5715697385
C 4.9740933471 1.4208549967 2.4664776014
H 5.7940269016 1.8844699281 3.0083505856
C 4.0806097129 0.5793910189 3.1292664640
H 4.2019196992 0.3796246899 4.1907070100
C 3.0326918938 -0.0197586099 2.4274953880
H 2.3489542947 -0.6852860920 2.9493497165
C 2.8524157034 0.2199955546 1.0579221519
N 2.1080984640 -0.9514036287 -1.0060163328
C 2.8108169810 -2.2661788921 -1.2447269989
C 4.2848933593 -1.9611322951 -1.5752499986
C 2.1223479245 -2.9256482165 -2.4543244275
C 2.7147134453 -3.1851294192 -0.0199698298
H 4.8021993750 -1.5229825432 -0.7157058628
H 4.3447744618 -1.2619938374 -2.4137357800
H 4.8016629972 -2.8862472235 -1.8535775473
H 2.6262852973 -3.8685676913 -2.6930197763
H 2.1747840932 -2.2693858244 -3.3259777023
H 1.0694219268 -3.1374955735 -2.2437609083
H 3.2148586864 -2.7580633343 0.8557565693
H 3.2206498946 -4.1270044370 -0.2575985161
H 1.6758357519 -3.4162833898 0.2332020592
O 2.0638471056 -0.1431343517 -2.0082802248
C 0.3637530180 0.4250122774 0.2326645798
N -0.5722224030 -0.1852308347 -0.7514734254
S -1.3721720651 -1.6506136276 -0.5296374116
O -1.5464665808 -2.1822851092 -1.8878324725
O -0.7363359169 -2.4807716966 0.5103270441
C -2.9800845716 -1.1692306791 0.1044334989
C -3.4494887244 -1.7423265453 1.2835566162
C -3.7771801421 -0.2869996563 -0.6316184775
C -4.7283684672 -1.4135228862 1.7375848369
H -2.8193946914 -2.4335614388 1.8323587126
C -5.0472696282 0.0299983643 -0.1639932548
H -3.4036838748 0.1439329388 -1.5546902973
C -5.5437122982 -0.5256723653 1.0275844646
H -5.6680954681 0.7182979676 -0.7326939656
C 0.6190502029 1.8727179832 -0.2681099265
H 1.3736134627 2.3214831412 0.3876118631
H 1.0703221452 1.8128003834 -1.2623389242
C -0.6091613712 2.7941933736 -0.3200199363
H -0.9905991864 2.9836694711 0.6921119928
H -1.4198335691 2.2992191678 -0.8694267086
C -0.2889761893 4.1407479390 -0.9846908424
H 0.0784382362 3.9640855206 -2.0063571865
C -1.4886584526 5.0954127110 -1.0366840371
H -1.8537937903 5.2744531020 -0.0151662402
C -1.1662016955 6.4356943354 -1.7053822579
H -2.0419876301 7.0944962655 -1.7267196724
H -0.3659761369 6.9626926487 -1.1710930890
H -0.8322142631 6.2918922069 -2.7403499826
H -5.0964063183 -1.8584094086 2.6586863327
C -6.9232782216 -0.1632074216 1.5233557295
H -6.9827943791 0.8999477810 1.7883933192
H -7.6829332773 -0.3451164680 0.7538439250
H -7.1962135288 -0.7435714387 2.4097428796
C -0.2762935082 0.4651807954 1.6316646635
H 1.4036948438 -1.3325119254 0.8908938127
H -0.1523860577 -0.2130821058 -1.6821412664
H -2.3153375135 4.6080200184 -1.5731677032
H 0.5376852049 4.6284641040 -0.4463731904
H -0.4051044629 -0.5354379675 2.0510266212
H -1.2558441776 0.9467786628 1.5901508850
H 0.3594893203 1.0430768819 2.3087200967
68

C 1.5932280884 -0.3472032142 0.2665340343
C 3.5776021735 1.2747766070 0.2148872663
H 3.4259364194 1.3939040521 -0.8532359046
C 4.6110144269 1.9574612041 0.8562335804
H 5.2562973657 2.6162310623 0.2809325709
C 4.8212156538 1.7954237184 2.2277885430
H 5.6280118737 2.3276687751 2.7244148153
C 3.9945964971 0.9372458020 2.9529071770
H 4.1554184797 0.7930664182 4.0180913662
C 2.9634546933 0.2500395646 2.3093056210
H 2.3324940520 -0.4275242749 2.8798731964
C 2.7331247618 0.4168675538 0.9364566458
N 2.0000665781 -0.8858869837 -1.0510774629
C 2.7663231509 -2.1710504982 -1.2524934408
C 2.7487104951 -3.0395581293 0.0120920553
C 4.2137259054 -1.8038118548 -1.6338061198
C 2.0855560089 -2.9176908936 -2.4148083353
H 3.2473049364 -2.5497377198 0.8552661653
H 3.2972203018 -3.9637341598 -0.1990567243
H 1.7299779493 -3.3124133833 0.3028884805
H 4.7726256753 -2.7118030718 -1.8858908756
H 4.7269779975 -1.3019067459 -0.8073368696

H 4.2163220738 -1.1398756299 -2.5025086263
H 2.0818907502 -2.2979705716 -3.3143197396
H 1.0508375836 -3.1753289745 -2.1680738113
H 2.6336319954 -3.8421989250 -2.6267064563
O 1.8868593753 -0.1259849630 -2.0852394053
C 0.2153703392 0.4480614055 0.1760002163
N -0.7097942117 -0.2572702456 -0.7529611344
S -1.4251307847 -1.7501101170 -0.4440633810
O -1.5982154077 -2.3551960397 -1.7713558927
O -0.7245768843 -2.4936493895 0.6194534637
C -3.0440228193 -1.3245699887 0.2018050322
C -3.9070813909 -0.5356974030 -0.5651762758
C -3.4532712761 -1.8501618651 1.4246636563
C -5.1827688610 -0.2628748715 -0.0849639785
H -3.5804875171 -0.1432621999 -1.5225979313
C -4.7386298958 -1.5668752735 1.8908396763
H -2.7727146091 -2.4709356343 1.9969208789
C -5.6194382279 -0.7712013188 1.1503019022
H -5.0600509099 -1.9752632153 2.8456883566
C 0.3747237964 1.8839571341 -0.3941853004
H 1.1318543403 2.3920660910 0.2110199999
H 0.7875779519 1.8039963525 -1.4040685064
C -0.9085483516 2.7305221682 -0.4322142711
H -1.2212762791 2.9970034148 0.5865027819
H -1.7216716905 2.1322091569 -0.8602344061
C -0.7558605738 4.0223158905 -1.2538061052
H -1.7399456725 4.5073464401 -1.3287735444
C 0.2474907938 5.0356971801 -0.6846307912
H 1.2504529712 4.5903218196 -0.6509006661
C 0.3023119854 6.3373736073 -1.4913332607
H 0.5975446927 6.1467516932 -2.5305844976
H -0.6766131949 6.8323791617 -1.5125355860
H 1.0237962240 7.0438523096 -1.0649634908
H -5.8552311943 0.3522460898 -0.6782594226
C -7.0050062343 -0.4548726481 1.6606771710
H -7.2393553301 -1.0252038639 2.5644789900
H -7.1038920896 0.6107612446 1.9037094958
H -7.7685866300 -0.6847748734 0.9083378224
C -0.3885209331 0.5179505270 1.5898405783
H 1.3688315701 -1.2179330292 0.8807915160
H -0.3130792189 -0.3055156157 -1.6928314763
H -0.0211749314 5.2618343468 0.3577408776
H -0.4650042705 3.7651542920 -2.2832560099
H 0.2326013128 1.1596096591 2.2215688392
H -0.4512604815 -0.4676914278 2.0569463129
H -1.3934175355 0.9448869026 1.5551262682
68

C 1.7411441191 -0.4348814963 0.3153838209
C 3.8432018345 1.0306630702 0.3799236117
H 3.7243793053 1.2121189353 -0.6835584882
C 4.9110039591 1.6007031324 1.0728532510
H 5.6167135581 2.2347630182 0.5425001607
C 5.0780181031 1.3580718140 2.4384193281
H 5.9117222107 1.8025703349 2.9752771069
C 4.1728160713 0.5325569886 3.1053421400
H 4.2986345540 0.3261384474 4.1649821557
C 3.1070476011 -0.0421054177 2.4099826055
H 2.4139541202 -0.6953357994 2.9350268412
C 2.9205040376 0.2068333341 1.0428971288
N 2.1348863911 -0.9408100129 -1.0190763148
C 2.8057607414 -2.2701830132 -1.2683594493
C 2.6932736322 -3.1935208967 -0.0482836325
C 4.2851733290 -1.9977740379 -1.6035118268
C 2.0969163627 -2.9063855339 -2.4786156294
H 1.6503488635 -3.4004019514 0.2093806639
H 3.2084056597 -2.7839836537 0.8271414068
H 3.1744832837 -4.1462647584 -0.2937751045
H 4.8162008232 -1.5769081479 -0.7437049798
H 4.3578415626 -1.2952395122 -2.4381532057
H 4.7790251815 -2.9330177525 -1.8895064522
H 2.5770944905 -3.8598608108 -2.7242061957
H 2.1618001384 -2.2469260604 -3.3469950284
H 1.0399843042 -3.0939108279 -2.2649972436
O 2.1026288967 -0.1268378425 -2.0171879190
C 0.4302821780 0.4676145767 0.2380137655
N -0.5254509593 -0.1196603013 -0.7410776598
S -1.3602567448 -1.5648998871 -0.5177817040
O -1.5494736865 -2.0928330250 -1.8754340442
O -0.7432739291 -2.4085815337 0.5224400868
C -2.9557427248 -1.0434098932 0.1161333617
C -3.7409259136 -0.1625291369 -0.6343892185
C -3.4276376168 -1.5805983396 1.3109172500
C -5.0011017580 0.1899857720 -0.1653445072
H -3.3668794522 0.2382763020 -1.5707731934
C -4.6964109835 -1.2161890649 1.7663650953
H -2.8078348354 -2.2727484734 1.8701843303
C -5.4992541590 -0.3283233370 1.0423388080
H -5.0665403064 -1.6333610404 2.6995227450
C 0.7091805605 1.9114882785 -0.2609685552
H 1.4851936099 2.3417166296 0.3819597259
H 1.1408835392 1.8458722182 -1.2635752312
C -0.5017116419 2.8573716061 -0.2864025547
H -0.8431824592 3.0709581933 0.7356108433
H -1.3364771308 2.3647734773 -0.7977709442
C -0.1686286011 4.1876764841 -0.9793069362
H 0.1210042618 3.9905150655 -2.0221812615
C -1.3074738411 5.2206160020 -0.9568440788
H -0.9294875515 6.1675668580 -1.3654179121
C -2.5573459138 4.8053366482 -1.7423795623
H -2.3106048186 4.5888089472 -2.7895094627
H -3.0256299967 3.9089544478 -1.3203944995
H -3.3093471713 5.6028920335 -1.7365433973
H -5.6130517727 0.8763532448 -0.7458885561
C -6.8671013030 0.0735282222 1.5401963038
H -6.8978432832 1.1399346859 1.7972062132
H -7.6342166778 -0.0944166103 0.7750286312
H -7.1513929789 -0.4924186484 2.4323269608
C -0.1970129523 0.5169303115 1.6424657573
H 1.4375355694 -1.3136403111 0.8824359001
H -0.1130419888 -0.1541361356 -1.6747680995
H -1.5849058763 5.4298881257 0.0864741922
H 0.7160768325 4.6286992616 -0.4977695049
H -0.3429265224 -0.4818840478 2.0603994962
H -1.1665198969 1.0191597914 1.6105864296
H 0.4564788468 1.0797820319 2.3153405907
68

C 1.7074403315 -0.4393353846 0.3210547385
C 3.8005937092 1.0392440365 0.3819653218
H 3.7135169758 1.1715583854 -0.6917762900
C 4.8446009200 1.6458316482 1.0800792114
H 5.5636093972 2.2588523633 0.5428981250
C 4.9710170213 1.4664310941 2.4597146749
H 5.7862964288 1.9392811787 3.0006288702
C 4.0492147584 0.6673264000 3.1359851612
H 4.1436209141 0.5098957504 4.2072369491
C 3.0073433364 0.0559940036 2.4356737618
H 2.3013658428 -0.5766921891 2.9686489653
C 2.8613906059 0.2415531985 1.0536608717
N 2.1441183510 -1.0043306742 -0.9759477686
C 2.8293166681 -2.3392887764 -1.1422650673
C 2.1606922074 -3.0364062908 -2.3418066209
C 2.6852942031 -3.2051821925 0.1160050461
C 4.3166688944 -2.0728235388 -1.4456979378
H 1.0990706285 -3.2217770560 -2.1508065796
H 2.6538334365 -3.9966257668 -2.5283387075
H 2.2473157639 -2.4175794765 -3.2377560360
H 3.1789202260 -4.1650300890 -0.0699958130
H 1.6362188433 -3.4068743786 0.3517903162
H 3.1716608383 -2.7515453114 0.9861364703
H 4.8239708937 -3.0171345202 -1.6721765513
H 4.8192465511 -1.6082181636 -0.5913242345
H 4.4106787190 -1.4100228282 -2.3101759287
O 2.1381028044 -0.2381668292 -2.0116847463
C 0.3959265943 0.4521278919 0.1621354289
N -0.5267550476 -0.1829232744 -0.8188223852
S -1.3580325820 -1.6238699046 -0.5568293290
O -1.5089926287 -2.2085890848 -1.8958639641
O -0.7628281577 -2.4206014447 0.5321001655

C -2.9719646974 -1.0891783148 0.0164258001
C -3.7368434161 -0.2293348598 -0.7778897068
C -3.4784141024 -1.6005271804 1.2086144668
C -5.0121072694 0.1285135834 -0.3558622242
H -3.3351949113 0.1521951718 -1.7108374399
C -4.7617771882 -1.2312973721 1.6163651247
H -2.8734175226 -2.2765547701 1.8027136746
C -5.5452256792 -0.3637767679 0.8476185524
H -5.1588714581 -1.6284076707 2.5471968726
C 0.6884905652 1.8733117897 -0.3912216094
H 1.4333474419 2.3370182209 0.2652969711
H 1.1645802455 1.7646610030 -1.3696766977
C -0.5221966257 2.8115402287 -0.5136500747
H -0.9302848576 3.0340899424 0.4795381863
H -1.3221468880 2.3114515018 -1.0746528927
C -0.1516681816 4.1256122126 -1.2186063365
H 0.2831532247 3.8887064105 -2.1998514524
C -1.3271135730 5.0963483249 -1.4211940388
H -2.1207991861 4.5882511430 -1.9877691884
C -1.9126501254 5.6685318608 -0.1244169142
H -1.1413148132 6.1832082707 0.4625355427
H -2.7065060252 6.3938926764 -0.3371594072
H -2.3446068806 4.8866628310 0.5103179801
H -5.6082437279 0.7989566299 -0.9704438424
C -6.9279186474 0.0457884830 1.2956614063
H -7.6663870918 -0.1086923198 0.5002786663
H -7.2510150028 -0.5245657937 2.1716651712
H -6.9591460225 1.1102918615 1.5609036718
C -0.2754891646 0.5598046042 1.5428319720
H 1.3901427876 -1.2926271854 0.9185903852
H -0.0851275540 -0.2563828626 -1.7368428083
H -0.9884365923 5.9278658845 -2.0539304786
H 0.6419634215 4.6344715490 -0.6506212266
H 0.3531014198 1.1567820270 2.2099864562
H -0.4281669899 -0.4205585720 2.0003108164
H -1.2469605701 1.0522233087 1.4591895981
68

C 1.6160535152 -0.3854127111 0.3553203104
C 3.6467552914 1.1644141375 0.5708198330
H 3.5171082365 1.4456729075 -0.4694116342
C 4.6882752936 1.7118318872 1.3197244150
H 5.3620069022 2.4283830204 0.8571391285
C 4.8700719472 1.3416816942 2.6544240074
H 5.6832885117 1.7689274035 3.2349312913
C 4.0065352178 0.4116566635 3.2332081973
H 4.1447169114 0.1061072448 4.2670451521
C 2.9672983936 -0.1400220269 2.4813894040
H 2.3073423463 -0.8748407970 2.9366328156
C 2.7655738542 0.2356717430 1.1457446034
N 2.0295843055 -0.7349639892 -1.0227038487
C 2.7634711640 -1.9983868454 -1.4032129983
C 2.0796517543 -2.5460421454 -2.6698910899
C 2.7038893518 -3.0425701857 -0.2808218106
C 4.2256762977 -1.6210217818 -1.7111285726
H 2.1045019988 -1.8004620401 -3.4678183794
H 1.0352992630 -2.8085801495 -2.4741512784
H 2.6063206824 -3.4440296310 -3.0108617714
H 3.2308948306 -3.9408047307 -0.6199533289
H 1.6739564124 -3.3260820601 -0.0449702681
H 3.2025195031 -2.6964928535 0.6307970152
H 4.7634058152 -2.4978852366 -2.0883599679
H 4.7401911981 -1.2610390328 -0.8144081988
H 4.2588764361 -0.8369889822 -2.4724587546
O 1.9548599506 0.1721152230 -1.9345802532
C 0.2626861685 0.4562667889 0.3676954871
N -0.6676675216 -0.0747742392 -0.6663864868
S -1.4257594944 -1.5766320915 -0.5922637588
O -1.6027119413 -1.9674254065 -1.9971435709
O -0.7555749201 -2.4936349733 0.3484307372
C -3.0378355914 -1.2089834688 0.1042742569
C -3.4777283767 -1.9158650205 1.2205511531
C -3.8668233568 -0.2766495839 -0.5272709752
C -4.7592795991 -1.6721322519 1.7184120326
H -2.8232022789 -2.6437173753 1.6872774511
C -5.1392220589 -0.0458586405 -0.0172064390
H -3.5161760408 0.2587779287 -1.4034111946
C -5.6062416721 -0.7370599064 1.1137586827
H -5.7850803818 0.6806219311 -0.5047368159
C 0.4752005668 1.9543238157 0.0159116859
H 1.2199984031 2.3552295756 0.7129337310
H 0.9249016184 2.0009292295 -0.9781084384
C -0.7794872421 2.8410197373 0.0697996860
H -1.1569295983 2.8900797001 1.0987375893
H -1.5788381751 2.3894949268 -0.5314901708
C -0.5251919492 4.2798760337 -0.4164520595
H 0.3184285981 4.7118839706 0.1430383125
C -0.2590143232 4.4191626563 -1.9223631456
H -1.0964218394 3.9718294885 -2.4771314552
C -0.0763831613 5.8759141613 -2.3615552346
H -0.9669138032 6.4745759379 -2.1324611916
H 0.7741326238 6.3410587847 -1.8477257801
H 0.1062729115 5.9493211355 -3.4397344484
H -5.1044801347 -2.2218183332 2.5904771332
C -6.9875427923 -0.4648492731 1.6598872136
H -7.2482438231 -1.1655657583 2.4586882757
H -7.0581868632 0.5504306712 2.0704962827
H -7.7491946338 -0.5476918085 0.8756713677
C -0.3615744470 0.3321419447 1.7688765998
H 1.3572511230 -1.3305473072 0.8301660087
H -0.2596071985 0.0073214366 -1.5990161736
H 0.6327130454 3.8445273439 -2.2037138556
H -1.4009953595 4.8933609017 -0.1602307326
H 0.2655458787 0.8551101932 2.4967656789
H -0.4564536480 -0.7100800346 2.0827604108
H -1.3551086342 0.7856068611 1.7871987898
68

C 1.5877487729 -0.3339698303 0.2674801294
C 3.5931704957 1.2626375533 0.2424170749
H 3.4850424930 1.3488017340 -0.8341350003
C 4.6091766560 1.9540399262 0.9019481818
H 5.2846289529 2.5865423938 0.3318233909
C 4.7635504444 1.8341620622 2.2852201650
H 5.5568801878 2.3731346944 2.7960772422
C 3.8985796729 1.0092049273 3.0041259239
H 4.0158726456 0.8977025197 4.0788717389
C 2.8848431423 0.3131877113 2.3425657950
H 2.2237300877 -0.3385701993 2.9090448359
C 2.7103540660 0.4381729102 0.9570945612
N 2.0386623267 -0.9189368744 -1.0156399760
C 2.7963534119 -2.2182224970 -1.1467132979
C 2.1508095371 -2.9945114381 -2.3097060824
C 2.7205446896 -3.0451547030 0.1432853030
C 4.2613742634 -1.8791162776 -1.4842117280
H 1.1046817666 -3.2333129231 -2.0940764415
H 2.6955544938 -3.9310398294 -2.4714951514
H 2.1879202660 -2.4038673971 -3.2278318333
H 1.6882917938 -3.2975723150 0.4032891996
H 3.1926750747 -2.5336585982 0.9888203139
H 3.2656327542 -3.9814859454 -0.0171248374
H 4.3045868282 -1.2436476819 -2.3728929714
H 4.8184605405 -2.8007269961 -1.6857974482
H 4.7490243334 -1.3563293492 -0.6551789831
O 1.9744047325 -0.1913822615 -2.0769465494
C 0.2247403820 0.4741999441 0.0977839711
N -0.6723254695 -0.2487653703 -0.8450327010
S -1.4176156527 -1.7230887923 -0.5185884677
O -1.5521320476 -2.3644184264 -1.8332420109
O -0.7642520764 -2.4440500028 0.5896783474
C -3.0521277735 -1.2595145042 0.0580492131
C -3.5100578020 -1.7452233365 1.2801009445
C -3.8777353117 -0.4820037153 -0.7601367528
C -4.8068152986 -1.4330156117 1.6933232498
H -2.8576636341 -2.3575219330 1.8928615465
C -5.1653582740 -0.1799592217 -0.3322364707
H -3.5130049746 -0.1204254580 -1.7159397266
C -5.6510353828 -0.6480177921 0.9007092517
H -5.8086248005 0.4268192869 -0.9651820175
C 0.4257042109 1.8895582876 -0.5093332054

H 1.1616048881 2.4086689747 0.1130739951
H 0.8828744966 1.7731811499 -1.4962713234
C -0.8440062611 2.7477843074 -0.6315214032
H -1.2048034703 3.0416060469 0.3619967340
H -1.6430971414 2.1469739847 -1.0817776707
C -0.6370493279 4.0078649634 -1.4919965047
H -1.6050717234 4.5185352181 -1.5997688544
C 0.3956788370 5.0210143536 -0.9663020436
H 0.4933500687 5.8266214533 -1.7065730604
C 0.0422709710 5.6360337861 0.3930396432
H 0.0167185247 4.8831174567 1.1892593611
H 0.7769258761 6.3952819396 0.6849604295
H -0.9424614710 6.1197101565 0.3639685241
H -5.1661239243 -1.8099439242 2.6475935522
C -7.0500043747 -0.3024359160 1.3521374714
H -7.1544266968 0.7754257648 1.5295240796
H -7.7920641217 -0.5761028153 0.5927432939
H -7.3112375504 -0.8196795902 2.2801657964
C -0.4332307823 0.5945939559 1.4838867399
H 1.3287018111 -1.1820315320 0.8996064356
H -0.2392028893 -0.3312868846 -1.7663119826
H 1.3852153154 4.5483742538 -0.9072256128
H -0.3415131794 3.6978859587 -2.5044084308
H -1.4301920933 1.0327184347 1.3974022104
H 0.1716306731 1.2467601805 2.1206572889
H -0.5276214684 -0.3756623946 1.9772398559
68

C 1.9141894464 -0.0596599922 0.1302844283
C 1.6021332680 1.1918076584 -2.0455572499
H 1.8751248686 2.1007538400 -1.5142757964
C 1.2926257767 1.2621452114 -3.4055290581
H 1.3262913141 2.2205041458 -3.9169646932
C 0.9517795812 0.1041835419 -4.1033265782
H 0.7158831634 0.1526443715 -5.1630142765
C 0.9277421951 -1.1208573515 -3.4320481290
H 0.6768742486 -2.0313366606 -3.9701031469
C 1.2306037701 -1.1891523906 -2.0720501051
H 1.2290540606 -2.1473485187 -1.5653417495
C 1.5648545074 -0.0287049496 -1.3571758050
N 2.8590950902 -1.1605650636 0.4378351787
C 4.3568139500 -1.0734021435 0.2886212631
C 4.8307933520 0.3836437117 0.2149509381
C 4.7548220577 -1.8297252069 -0.9938028166
C 4.9641106277 -1.7610971796 1.5246217416
H 5.9236686730 0.3893078038 0.1481969428
H 4.5551256774 0.9512747221 1.1107550758
H 4.4463570771 0.9010943951 -0.6698161450
H 4.3809258939 -2.8559269037 -0.9543137944
H 5.8459648048 -1.8565273173 -1.0882238853
H 4.3425626603 -1.3424828779 -1.8830108565
H 4.7088725683 -1.2155195979 2.4398039501
H 6.0551369059 -1.7918603499 1.4344749271
H 4.5878870870 -2.7822160018 1.6144824789
O 2.3818696645 -2.3523218260 0.5556123467
C 0.6881027873 -0.0635379254 1.1283058005
N -0.1456531682 -1.2438188239 0.7735314844
S -1.6379730670 -1.6116310731 1.4548711129
O -1.7203482209 -3.0745054106 1.3635139125
O -1.8519023547 -0.9401650713 2.7477071244
C -2.8228879907 -0.8976697290 0.3081308557
C -3.9456521416 -0.2532452853 0.8252443986
C -2.6710182318 -1.0726543143 -1.0698924861
C -4.9196814797 0.2280871290 -0.0503795484
H -4.0458657529 -0.1286675504 1.8980073866
C -3.6504310800 -0.5830993798 -1.9284233489
H -1.7889112995 -1.5669265580 -1.4618493925
C -4.7891797247 0.0739212325 -1.4354218142
H -3.5294388617 -0.7119371069 -3.0015342561
C -0.1534646800 1.2222037575 0.9210898067
H -0.4748638948 1.2648984161 -0.1255365714
H -1.0614549537 1.1109578524 1.5222102472
C 0.4980084252 2.5565961239 1.3181790924
H 1.4450583638 2.7136891471 0.7823053183
H 0.7501077323 2.5477865104 2.3862160834
C -0.4254110669 3.7508438057 1.0358317588
H -1.3777554813 3.6040361399 1.5657335778
C 0.1789569135 5.1002534998 1.4444994314
H 1.1323891445 5.2463312106 0.9163103211
C -0.7478563062 6.2866933500 1.1604410746
H -0.2886905389 7.2347387354 1.4628479147
H -0.9856851864 6.3573682943 0.0918220417
H -1.6956808651 6.1874055776 1.7034796108
H -5.7953878343 0.7314173808 0.3521526196
C -5.8331557488 0.6159468301 -2.3827899492
H -5.4753070547 1.5209642739 -2.8910572292
H -6.0826483358 -0.1131152587 -3.1620873291
H -6.7559543402 0.8770027464 -1.8556246811
C 1.2113045418 -0.1614293153 2.5773999132
H 2.4509922919 0.8596416276 0.3603503008
H 0.4230640939 -2.0943579322 0.7809068567
H 0.4258883564 5.0769545727 2.5153891278
H -0.6752320866 3.7731364391 -0.0352377270
H 0.3809654730 -0.0437565063 3.2773212153
H 1.9588342650 0.6126420514 2.7856096615
H 1.6718749297 -1.1361989884 2.7543147841
68

C 1.6598410559 -0.3633297037 0.3477753729
C 3.7230779723 1.1400085095 0.5812934580
H 3.5875213579 1.4517222460 -0.4494794428
C 4.7834042257 1.6466294395 1.3325326009
H 5.4661003453 2.3620732346 0.8815359386
C 4.9723514307 1.2372909935 2.6547399876
H 5.8001667035 1.6328094883 3.2370939344
C 4.0968441303 0.3091837059 3.2184184605
H 4.2401431105 -0.0265929419 4.2421337162
C 3.0387172229 -0.2016957079 2.4641214060
H 2.3692841167 -0.9353393646 2.9072198243
C 2.8299948431 0.2135497344 1.1413529964
N 2.0516069986 -0.6845155107 -1.0433978001
C 2.7552905050 -1.9519744251 -1.4651687248
C 4.2217250803 -1.5964617825 -1.7787954898
C 2.0471325918 -2.4516853386 -2.7382536810
C 2.6857860499 -3.0241605926 -0.3700503574
H 4.2632869670 -0.7927752541 -2.5189323916
H 4.7371234928 -2.4735614720 -2.1854989624
H 4.7528851733 -1.2717727418 -0.8783136477
H 2.0803691189 -1.6864312067 -3.5170147744
H 0.9992612483 -2.6964980995 -2.5383084208
H 2.5505928976 -3.3517148856 -3.1076740740
H 1.6527737860 -3.2931009642 -0.1304117072
H 3.2008167513 -2.7124824730 0.5448451594
H 3.1907002591 -3.9235829099 -0.7383827142
O 1.9846716923 0.2475810639 -1.9303544458
C 0.3238341769 0.5040345048 0.3973612001
N -0.6272314396 0.0186245271 -0.6409982409
S -1.4186617735 -1.4674601894 -0.5956802623
O -1.6142794132 -1.8211984972 -2.0078953380
O -0.7621179959 -2.4203753187 0.3184445437
C -3.0171619318 -1.0798907363 0.1208280188
C -3.8333498510 -0.1226316682 -0.4898646347
C -3.4606790348 -1.7937414712 1.2311781039
C -5.0962579658 0.1257496067 0.0352315910
H -3.4806902852 0.4178512229 -1.3620943415
C -4.7325981954 -1.5319734360 1.7444097284
H -2.8165997343 -2.5411237069 1.6812790584
C -5.5664381590 -0.5720348460 1.1608495833
H -5.0806540388 -2.0871599946 2.6118410082
C 0.5585364974 2.0065478308 0.0812608135
H 1.3315897371 2.3743906803 0.7655194399
H 0.9810406211 2.0706318266 -0.9238502419
C -0.6777321645 2.9127832469 0.1952836135
H -1.0024626788 2.9704404852 1.2417429107
H -1.5133758076 2.4700530443 -0.3588032834
C -0.4220937614 4.3505266072 -0.2964620004
H 0.4175040871 4.7774422009 0.2711500025
C -0.1357447231 4.5125490757 -1.8003814328
H 0.7784944480 3.9687708900 -2.0717384650
C -1.2826059082 4.0635962859 -2.7135667960

H -1.0528942293 4.2721802812 -3.7646572591
H -1.4745812072 2.9879140219 -2.6290198193
H -2.2144673024 4.5880557896 -2.4656742277
H -5.7320860260 0.8714133211 -0.4362320425
C -6.9371062490 -0.2805417477 1.7235286136
H -7.7072460930 -0.3372240154 0.9453016481
H -7.2044531283 -0.9872610082 2.5148058169
H -6.9839219780 0.7300116623 2.1489360787
C -0.2873578359 0.3551813880 1.8018423010
H 1.3871828774 -1.3154490579 0.8005546182
H -0.2229847576 0.1119542741 -1.5742859143
H 0.0798324676 5.5722426741 -1.9934753996
H -1.3001800081 4.9619997532 -0.0427769075
H -1.2725614882 0.8251318641 1.8428485857
H 0.3566808322 0.8491410620 2.5352306469
H -0.3966251970 -0.6927765896 2.0908335854
68

C 1.7349883165 -0.3466295562 0.4679681344
C 3.7630753994 0.8585200651 -0.5288445238
H 3.2212605991 0.9492249190 -1.4651167122
C 5.0572299761 1.3666944541 -0.4185149296
H 5.5142151681 1.8603114673 -1.2721967698
C 5.7655187815 1.2411284732 0.7790286844
H 6.7742483850 1.6365178457 0.8620890893
C 5.1720039468 0.5963848463 1.8642511120
H 5.7164068740 0.4831368633 2.7980129751
C 3.8778613191 0.0843087971 1.7515456061
H 3.4271862166 -0.4260894012 2.5994091696
C 3.1524342725 0.2167934874 0.5594918627
N 1.5150616147 -1.0420334002 -0.8225274177
C 2.0341758635 -2.4233537219 -1.1426665783
C 0.8833521878 -3.1870449285 -1.8228567249
C 2.4715116254 -3.1669907633 0.1260604053
C 3.2171059564 -2.2744203070 -2.1196473304
H 0.5305906660 -2.6416952395 -2.7012477637
H 0.0518454159 -3.3185557197 -1.1249010067
H 1.2351122095 -4.1750111530 -2.1393349022
H 2.8201795760 -4.1641596861 -0.1635726842
H 1.6382460485 -3.2922636017 0.8234544441
H 3.3016553250 -2.6657661828 0.6338065310
H 2.9118146681 -1.6911255215 -2.9928110367
H 3.5441449197 -3.2639538923 -2.4576917217
H 4.0668357006 -1.7755772928 -1.6438273237
O 1.0295510937 -0.3748564461 -1.8125000222
C 0.5877800759 0.7093415560 0.7957075745
N -0.7456292509 0.1666626129 0.3990921795
S -1.5103098532 -1.0892966273 1.2423252035
O -0.7800945710 -2.3703548531 1.1422783401
O -1.8628176439 -0.5922371218 2.5766013435
C -2.9950712732 -1.2245626161 0.2450159084
C -3.2367418243 -2.3919037805 -0.4759043506
C -3.9203149789 -0.1761262117 0.2501166580
C -4.4153771667 -2.5007794123 -1.2152360623
H -2.5157298734 -3.2014038184 -0.4468947742
C -5.0877037471 -0.3013365499 -0.4950925449
H -3.7256537135 0.7170858163 0.8342485104
C -5.3527790444 -1.4615005336 -1.2416580901
H -5.8104547014 0.5111024980 -0.4947883634
C 0.7418389581 2.0403871313 0.0089851283
H 1.7404261084 2.4378762324 0.2219843109
H 0.7193228832 1.8105419556 -1.0600226615
C -0.3019810002 3.1242557753 0.3176222220
H -0.2136719438 3.4563327102 1.3602980459
H -1.3096963509 2.7047292965 0.2094745101
C -0.1525463057 4.3439811746 -0.6027875175
H -0.2520497800 4.0242337032 -1.6507810331
C -1.1720390335 5.4533940362 -0.3140736845
H -1.0709112620 5.7749416876 0.7323364802
C -1.0229952797 6.6670088253 -1.2373258033
H -0.0283277077 7.1194539704 -1.1396587243
H -1.1540850424 6.3835312800 -2.2890168003
H -1.7643948576 7.4405612361 -1.0061299869
H -4.6090154021 -3.4107906969 -1.7774816424
C -6.6158280511 -1.5723136666 -2.0617355078
H -6.8040909906 -2.6047909985 -2.3713019330
H -7.4887674132 -1.2209238697 -1.5003350412
H -6.5495882008 -0.9608914596 -2.9711055570
C 0.6383037718 0.9944791189 2.3077183843
H 1.6340318627 -1.1190986840 1.2312093477
H -0.7104380937 -0.0848455652 -0.5925773511
H -2.1879760003 5.0439186523 -0.4083107747
H 0.8641394415 4.7544133439 -0.5073531843
H 0.6377509736 0.0799810063 2.9061005228
H -0.2274862054 1.5812725432 2.6164574920
H 1.5471191054 1.5584444276 2.5379407727
68

C 1.7451289056 -0.4139743188 0.2101197992
C 3.0827054293 0.4274668111 2.1876660758
H 2.3820093142 -0.0977087199 2.8326825162
C 4.1394827809 1.1414620244 2.7560899550
H 4.2504752189 1.1704183060 3.8368784396
C 5.0546523282 1.8040318928 1.9381555878
H 5.8813327955 2.3562496616 2.3768980772
C 4.9068251953 1.7435169981 0.5503456233
H 5.6206540383 2.2482525896 -0.0954254683
C 3.8480775480 1.0344923609 -0.0167416133
H 3.7447721862 0.9796299128 -1.0957667991
C 2.9150318300 0.3731420736 0.7967180404
N 2.1588450946 -1.2007137716 -0.9739168621
C 2.8286002574 -2.5524766539 -0.9137469946
C 2.6958419679 -3.1838892962 0.4781838272
C 4.3135185207 -2.3607573599 -1.2790989790
C 2.1348070679 -3.4400451543 -1.9638689437
H 1.6486573393 -3.3298823522 0.7590973435
H 3.1990046754 -2.5909220776 1.2493618369
H 3.1783373868 -4.1668791793 0.4560674494
H 4.8333820059 -1.7598753165 -0.5260363126
H 4.4005067861 -1.8604640085 -2.2473767576
H 4.8085005693 -3.3360482155 -1.3435319348
H 2.6164669560 -4.4236415382 -1.9873523918
H 2.2124421581 -2.9875882690 -2.9550491079
H 1.0748230435 -3.5772499167 -1.7283158183
O 2.1467460742 -0.6259123330 -2.1268473853
C 0.4367919479 0.4474698746 -0.0831783230
N -0.5010735082 -0.3372849973 -0.9321580874
S -1.3456420330 -1.7025577941 -0.4247254641
O -1.5198953569 -2.5003517150 -1.6456307080
O -0.7471209502 -2.3153261428 0.7757367150
C -2.9451797865 -1.0553503001 0.0673571170
C -3.4492943988 -1.3638402518 1.3282900690
C -3.7025575544 -0.3166890257 -0.8472035613
C -4.7221925860 -0.9114361045 1.6813942873
H -2.8503062606 -1.9488678211 2.0174956467
C -4.9673578496 0.1270233657 -0.4779721510
H -3.3030948808 -0.0937389859 -1.8310905619
C -5.4976036749 -0.1604577887 0.7912959518
H -5.5575263536 0.7036257280 -1.1863558075
C 0.7285144769 1.7500935814 -0.8792312738
H 1.4816986540 2.3163821207 -0.3207074394
H 1.2028473464 1.4568483037 -1.8176875044
C -0.4922462273 2.6588136552 -1.1628770815
H -1.4175957095 2.0729707427 -1.0983366844
H -0.4358014030 3.0014392998 -2.2047307330
C -0.5949897942 3.8993298741 -0.2617613276
H 0.3238371494 4.4958384605 -0.3646973588
C -1.8074227726 4.7821464935 -0.5838204614
H -2.7262408393 4.1879627512 -0.4753103267
C -1.8993267943 6.0306911575 0.2995150625
H -1.9782129929 5.7613160360 1.3601345188
H -1.0100394634 6.6628665132 0.1856349592
H -2.7747144796 6.6392132173 0.0447828727
H -5.1173318014 -1.1501263068 2.6656330404
C -6.8683412247 0.3405722129 1.1790701341
H -7.1956263932 -0.0843420122 2.1326965041
H -6.8763306815 1.4333188602 1.2809250549
H -7.6160021414 0.0833906979 0.4197102839
C -0.2174535445 0.7895998542 1.2660501669
H 1.4316304296 -1.1471892407 0.9518129872

H -0.0726293410 -0.5678994667 -1.8301169325
H -1.7649208468 5.0834248838 -1.6403776506
H -0.6400769963 3.5979213692 0.7940031288

H -0.4104572867 -0.1040002512 1.8644940198
H -1.1664295452 1.3083869411 1.1105351546
H 0.4427304223 1.4488269143 1.8362520708

oxo-PBN

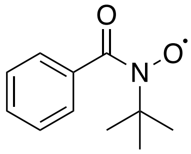

Name	E(B3LYP)	H(B3LYP)	g-factor	α_N
PBN_acyl_rad.crest.00	-632.745214155	-632.498068	2.00692	7.6362
Boltzman averaged for 298.15 K			2.00692	7.6362

28
-632.498068
O -0.764543 1.506643 -0.663136
N -1.079184 0.368043 -0.173709
C -2.554214 0.050848 0.038123
C -3.001245 -0.981964 -1.013702
C -2.765112 -0.470283 1.471113
C -3.328600 1.360812 -0.153667
C -0.059344 -0.604669 0.050602
C 1.374128 -0.179871 0.007389
C 1.847505 1.113882 0.282375
C 3.218579 1.365163 0.299523
C 4.129709 0.342700 0.028850
C 3.666246 -0.946664 -0.242887
C 2.299570 -1.207937 -0.241137
H -4.078819 -1.154737 -0.913724
H -2.480141 -1.931350 -0.884533
H -2.811513 -0.605362 -2.024878
H -2.249271 -1.415960 1.638804
H -3.836900 -0.623820 1.638171
H -2.412858 0.262870 2.205573
H -3.001337 2.128939 0.552854
H -4.390366 1.158360 0.021188
H -3.210127 1.758165 -1.163837
H 1.150877 1.916444 0.483063
H 3.574982 2.367239 0.521607
H 5.196825 0.548842 0.033330
H 4.369458 -1.748520 -0.450134
H 1.925477 -2.208863 -0.427843
O -0.379874 -1.766725 0.266490

(oxo-PBN)-PBN

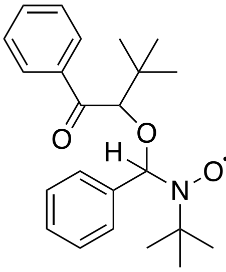

Name	E(B3LYP)	H(B3LYP)	g-factor	α_N	α_H
spin_trap_PBN_acyladduct.crest.020	-1190.86902323	-1190.364905	2.00643	13.95227	1.83726
spin_trap_PBN_acyladduct.crest.206	-1190.86181527	-1190.358920		12.30408	1.57746
spin_trap_PBN_acyladduct.crest.112	-1190.86237842	-1190.358530		13.23232	1.89911
spin_trap_PBN_acyladduct.crest.196	-1190.86132150	-1190.357623		11.83741	1.89966
spin_trap_PBN_acyladduct.crest.092	-1190.86066469	-1190.356750		14.47883	1.38562
spin_trap_PBN_acyladduct.crest.082	-1190.86001948	-1190.356360		12.80800	0.96580
Boltzman averaged for 298.15 K			2.00643	13.9522	1.8372

56
-1190.36490500
O 0.1828959591 -0.2283280689 1.0567144684
N 1.5025892430 -0.6173710403 0.7253753570
C 2.2895186322 0.2703989790 0.0242100265
O 3.3769859872 -0.0878829175 -0.4308497928
C 1.8196550989 1.6849467752 -0.2029180380
C 1.1465482712 2.4512834216 0.7605022281
C 0.8608317940 3.7940467297 0.5108888865
C 1.2280893730 4.3798816120 -0.7022882972
C 1.9042943005 3.6233836101 -1.6627664021
C 2.2117957162 2.2888925434 -1.4071779514
H 2.7724914799 1.6990364971 -2.1251418258
H 2.2030095147 4.0768774120 -2.6040709314
H 0.9958033111 5.4240119536 -0.8952039975
H 0.3489549228 4.3826443270 1.2673746576
H 0.8475346557 2.0004672299 1.6991129605
C 2.0493802178 -1.8176423933 1.4623758359
C 2.3663906977 -2.9361639951 0.4533684234
H 3.0679340866 -2.5801681834 -0.3043281378
H 1.4498988593 -3.2835184865 -0.0315820861
H 2.8175369625 -3.7881932967 0.9756476500
C 1.0054416504 -2.3069987260 2.4774117355
H 0.7743896908 -1.5372192778 3.2199062068
H 1.4299321460 -3.1700170277 3.0017616560
H 0.0795009720 -2.6139783421 1.9895440492
C 3.3222630288 -1.4009624002 2.2271119335
H 4.1113208066 -1.0692225187 1.5512528385
H 3.6913579922 -2.2594004752 2.7988179090
H 3.0986839925 -0.5940986211 2.9344303981
C -0.7224509267 -0.2528314166 -0.0647790027
H -0.3191319424 0.3744300180 -0.8579402057
N -0.8632020405 -1.6111166560 -0.6240581398
C -0.9661036626 -1.8601272659 -2.1118057619
C 0.0590853755 -1.0082011741 -2.8788764675
H 0.0861823524 -1.3456536025 -3.9202720714
H -0.1951729917 0.0564175446 -2.8964090664
H 1.0651677893 -1.1227685908 -2.4622475869
C -2.4045259632 -1.5344753202 -2.5597077565
H -3.1213841042 -2.1318939183 -1.9879158699
H -2.5282418473 -1.7653121880 -3.6238044045
H -2.6435683531 -0.4764942284 -2.4105189595
C -0.6663337428 -3.3486611505 -2.3467636158
H -1.3435389479 -3.9809964953 -1.7713132964
H -0.7877294026 -3.5736117938 -3.4114532846
H 0.3617366431 -3.5911922572 -2.0599333922
O -1.3266366643 -2.5175170746 0.1569101316
C -2.0168163357 0.3471519164 0.4458469668
C -2.7558550506 -0.2599539672 1.4705197035
C -3.9388669442 0.3270175277 1.9174805513
C -4.3945868336 1.5208458304 1.3523694551
C -3.6581146269 2.1325972135 0.3377190920
C -2.4716940916 1.5491252268 -0.1097037393
H -1.8908133831 2.0377538882 -0.8882775341
H -4.0016455644 3.0644971059 -0.1031583937
H -5.3183081183 1.9727755888 1.7040483437
H -4.5076483349 -0.1501604236 2.7110763927
H -2.4059343456 -1.1904564829 1.9001574809
56
-1190.35892000
O 0.5132874542 -0.8412508519 0.4526778738
N 1.5622401956 -0.4453781391 -0.3981650516
C 1.9933528246 0.8726613817 -0.2834414409
O 2.9251996930 1.2600809618 -0.9868181484
C 1.3528031632 1.8373628857 0.6792736249
C 0.8774636285 1.5102207928 1.9580126404
C 0.4314848477 2.5163278012 2.8166323357
C 0.4394160500 3.8506190038 2.4090019608
C 0.9174208919 4.1837697671 1.1388383647
C 1.3850041091 3.1861472138 0.2888983098
H 1.7887582217 3.4327341933 -0.6871143916
H 0.9346740866 5.2213586992 0.8158107262
H 0.0801247371 4.6279294960 3.0787069510
H 0.0698033975 2.2506387157 3.8062347048
H 0.8413428601 0.4790971452 2.2808804428
C 2.5242688381 -1.5350407967 -0.8300724915
C 2.8093569754 -1.3834819554 -2.3366408065
H 1.8773109912 -1.4376299535 -2.9102970106
H 3.4643231650 -2.1975154544 -2.6700207035
H 3.2897755509 -0.4284208665 -2.5466005805
C 1.8975549289 -2.9144536003 -0.5803682874
H 2.6363049369 -3.6747424658 -0.8533020018
H 1.0144440675 -3.0872531611 -1.2036345377
H 1.6278545878 -3.0604429869 0.4678223145
C 3.8263722684 -1.4371813127 -0.0092899313
H 4.5193137788 -2.2272808086 -0.3198016031
H 3.6175758556 -1.5683082149 1.0587754992
H 4.3160238349 -0.4736576024 -0.1583880520
C -0.7645081570 -1.0219509691 -0.2524329629
H -0.6600289007 -1.8833471315 -0.9084402069
N -1.6963245746 -1.3804418819 0.8128192953
C -2.2358160047 -2.7648801178 1.0653665396
C -3.6986114577 -2.5891339152 1.5099732531
H -3.7585387370 -1.9226189878 2.3720433401
H -4.3016759324 -2.1630593032 0.7007125476
H -4.1211443415 -3.5621084908 1.7817183309

C -1.4020833629 -3.3982592897 2.1942200193
H -1.4247472110 -2.7558263734 3.0786683944
H -0.3588242760 -3.5277145223 1.8875561233
H -1.8073311068 -4.3806024496 2.4615325857
C -2.1875812636 -3.6294500268 -0.2017508304
H -1.1666441180 -3.8969998138 -0.4921188043
H -2.7213739642 -4.5651339654 -0.0058802482
H -2.6815964680 -3.1368567454 -1.0466476394
O -1.8544956455 -0.5255485214 1.7570021878
C -1.2221535259 0.1550074660 -1.0909865888
C -1.7194895238 1.3454580364 -0.5410719755
C -2.1529210333 2.3727669922 -1.3778208637
C -2.0923867873 2.2325508424 -2.7663904306
C -1.5970759615 1.0526413335 -3.3209566981
C -1.1683795112 0.0213996508 -2.4848301822
H -0.7870962946 -0.8986629148 -2.9223966126
H -1.5441842394 0.9325066427 -4.3995783213
H -2.4295869219 3.0392658449 -3.4119848668
H -2.5363904004 3.2904712606 -0.9403370083
H -1.7698210782 1.4553544605 0.5343959466
56
-1190.35853000
O 0.5703646311 -0.8628443685 0.5249511021
N 1.7390416948 -0.3990151586 -0.1118172558
C 2.0955635051 0.9216522818 0.1613958549
O 3.1343398110 1.3722541516 -0.3165645923
C 1.2335381797 1.8114973244 1.0173118945
C 1.2583216362 3.1752958560 0.6854772845
C 0.5893696765 4.1099369168 1.4705734259
C -0.0849069316 3.6981891352 2.6226597539
C -0.0895726844 2.3482139629 2.9763341995
C 0.5557368805 1.4037893682 2.1768479777
H 0.5157969955 0.3575721579 2.4492110344
H -0.6029024748 2.0222766693 3.8770694846
H -0.5990871740 4.4269308374 3.2443033772
H 0.6037554736 5.1601040210 1.1910765495
H 1.8166151306 3.4859164494 -0.1911878934
C 2.8101832867 -1.4552464280 -0.3231731249
C 2.1698388947 -2.8520292316 -0.3089757179
H 1.4831322192 -2.9988261712 -1.1492748284
H 1.6362835378 -3.0524592880 0.6220910768
H 2.9706611955 -3.5908410214 -0.4155669043
C 3.8496698354 -1.3759139638 0.8122360666
H 4.6212944300 -2.1397049175 0.6640276194
H 3.3733573414 -1.5579771364 1.7824134900
H 4.3360643669 -0.3987809010 0.8340326120
C 3.4714795698 -1.2273737545 -1.6956239967
H 3.9678498158 -0.2585577673 -1.7355434227
H 2.7209674607 -1.2680302756 -2.4934099606
H 4.2082292030 -2.0182825450 -1.8807668971
C -0.5164575549 -1.0340968900 -0.4268247658
H -0.1465232291 -1.6423392097 -1.2504359678
N -1.4968964259 -1.8261038081 0.3235003288
C -2.4044025981 -2.8601537691 -0.2886576459
C -3.8067049273 -2.2366015020 -0.4258885399
H -4.5174058284 -2.9856108323 -0.7919365274
H -4.1492655893 -1.8720244489 0.5459308826
H -3.7969183913 -1.3966360343 -1.1282114551
C -2.4384175051 -4.0472865873 0.6893392968
H -2.7345747537 -3.7073546782 1.6840034681
H -1.4522209537 -4.5185872179 0.7638535804
H -3.1560804565 -4.7981363229 0.3418782495
C -1.8911839913 -3.3205120249 -1.6588760377
H -0.8884253707 -3.7573154092 -1.5955756244
H -2.5631939473 -4.0971463605 -2.0384267749
H -1.8828552321 -2.5087766991 -2.3939053091
O -1.8170518284 -1.3946736837 1.4896501924
C -1.0682046583 0.2629497589 -0.9893650896
C -1.9240573190 1.0958038453 -0.2540295166
C -2.4087976418 2.2729961638 -0.8197912606
C -2.0464550119 2.6351333604 -2.1204085038
C -1.1960974791 1.8116749191 -2.8575856682
C -0.7131282995 0.6294408598 -2.2935516520
H -0.0472832865 -0.0093569939 -2.8691829864
H -0.9083463485 2.0853887404 -3.8690247678
H -2.4265611752 3.5555039292 -2.5558541628
H -3.0687952150 2.9133906994 -0.2411346654
H -2.1994117523 0.8158249388 0.7562247691
56
-1190.35762300
O 0.6326131597 -0.8648960434 0.4376281507
N 1.6225881699 -0.3802765920 -0.4410234765
C 2.0022149678 0.9495463091 -0.2635673094
O 2.9110137329 1.4073121170 -0.9537455573
C 1.3245041918 1.8462132543 0.7388463815
C 0.8830281831 1.4544064580 2.0116494632
C 0.3994858052 2.4093655947 2.9077903012
C 0.3352782058 3.7546861222 2.5442399955
C 0.7780524140 4.1520124917 1.2797385418
C 1.2838745816 3.2070434379 0.3925504316
H 1.6619019389 3.5055314951 -0.5792782424
H 0.7385771129 5.1988585221 0.9902041954
H -0.0528065090 4.4911531280 3.2434604346
H 0.0651101353 2.0937130994 3.8923155231
H 0.8989843683 0.4131319238 2.3010444614
C 2.6381168524 -1.4068861462 -0.9082209261
C 2.9554987783 -1.1587717910 -2.3954931300
H 2.0386340857 -1.2013726811 -2.9942018610
H 3.6361588695 -1.9388371508 -2.7574176765
H 3.4179203178 -0.1835024851 -2.5404846758
C 2.0539938703 -2.8192181923 -0.7618955461
H 2.8256261636 -3.5366488419 -1.0589562332
H 1.1956528204 -2.9788634537 -1.4229887531
H 1.7578386765 -3.0390108659 0.2655011329
C 3.9124806442 -1.3095984092 -0.0458425774
H 3.6803465361 -1.5114975485 1.0062163517
H 4.3671633152 -0.3205261164 -0.1247980737
H 4.6454525174 -2.0522054352 -0.3807475159
C -0.6574456396 -1.0736179182 -0.2177492957
H -0.5471663207 -1.9004508490 -0.9220208921
N -1.5176415669 -1.5385125415 0.8687939048
C -2.3057271733 -2.8163337457 0.7740105374
C -1.3345283635 -4.0118677483 0.7435892945
H -0.6879265672 -3.9973962825 -0.1393144749
H -0.6993121622 -4.0145855567 1.6350750283
H -1.9015047116 -4.9492733548 0.7213101174
C -3.1796161058 -2.7864038801 -0.4953524652
H -3.8526769616 -1.9234371758 -0.4836612227
H -3.7867212391 -3.6971775721 -0.5442878357
H -2.5824049266 -2.7399439932 -1.4118620301
C -3.1990274297 -2.9031122659 2.0169773527
H -3.7866973043 -3.8256024138 1.9624567295
H -2.6052659661 -2.9170810542 2.9335318855
H -3.8835881832 -2.0532934970 2.0736877540
O -1.6275981802 -0.7890649505 1.9037094913
C -1.2050850656 0.1253560328 -0.9693922000
C -1.8181994808 1.2086961335 -0.3241192373
C -2.3279003526 2.2693725413 -1.0714100643
C -2.2295608100 2.2673851505 -2.4649319859
C -1.6202453071 1.1934675841 -3.1139556021
C -1.1154705603 0.1283363331 -2.3675103153
H -0.6445378376 -0.7087687092 -2.8779318429
H -1.5384122885 1.1817975280 -4.1973961336
H -2.6267883031 3.0986834143 -3.0414660072
H -2.8002705157 3.1039962548 -0.5606505781
H -1.8931469550 1.2113494215 0.7564623399
56
-1190.35675000
O 0.4314185951 -0.5706626490 0.5955144437
N 1.8051047861 -0.4922473792 0.2559080694
C 2.2636848337 0.7450458983 -0.1723129121
O 3.2248389522 0.8437134612 -0.9305010839
C 1.6264553938 1.9734018100 0.4083286760
C 1.2775427010 2.0562428503 1.7631053604
C 0.8329744233 3.2644260481 2.2985289569
C 0.7163322316 4.3918716974 1.4817516987
C 1.0671837901 4.3140904632 0.1318729971
C 1.5375938844 3.1143410199 -0.3982137804
H 1.8486028346 3.0486045330 -1.4361292298
H 0.9854126474 5.1913099413 -0.5040512998

H 0.3627635081 5.3312530209 1.8987538625
H 0.5804479496 3.3275590113 3.3538166939
H 1.3678484320 1.1773356793 2.3935967518
C 2.5728900368 -1.7729353240 0.1008947898
C 4.0125646659 -1.5277532686 0.5997344708
H 4.0051052816 -1.1587509231 1.6314739720
H 4.5432760838 -0.8114785751 -0.0269484100
H 4.5561632759 -2.4785343303 0.5819211538
C 2.5881259388 -2.2504722985 -1.3648939406
H 3.1977240602 -3.1581063485 -1.4486367796
H 3.0262749789 -1.4814779922 -2.0060678311
H 1.5829008089 -2.4905302314 -1.7244422403
C 1.9315287654 -2.8314492553 1.0095414051
H 2.5508224247 -3.7339009564 0.9819861965
H 0.9292542541 -3.0993682159 0.6752615314
H 1.8762497507 -2.4841879430 2.0467322431
C -0.4329275359 -0.6816717050 -0.5574355833
H 0.1794794492 -0.6116632595 -1.4549629237
N -1.0151367427 -2.0469246650 -0.6791931501
C -2.0537724832 -2.6734329116 0.2234926772
C -1.8007908578 -4.1916702875 0.1951162478
H -2.5698832519 -4.6969411804 0.7887399508
H -1.8373770269 -4.5693451685 -0.8282376897
H -0.8239545054 -4.4407289577 0.6218955438
C -3.4554924629 -2.3791120990 -0.3482748110
H -3.5030058495 -2.6887752685 -1.3963213662
H -3.7038529013 -1.3166953634 -0.2844330338
H -4.2080967805 -2.9420785057 0.2151831067
C -1.9306607036 -2.1530196815 1.6615759863
H -2.6550227604 -2.6895161664 2.2840989173
H -0.9332115723 -2.3252665654 2.0728288321
H -2.1599277687 -1.0880471562 1.7367972010
O -0.9149975569 -2.5509393161 -1.8616339195
C -1.4914539406 0.4125666976 -0.5868465083
C -1.8093569743 1.2121641498 0.5138444575
C -2.8004643192 2.1905700637 0.4082853972
C -3.4789488157 2.3803237968 -0.7955123312
C -3.1590787428 1.5883596758 -1.9013673256
C -2.1709644675 0.6113510512 -1.7978096339
H -1.9284259548 -0.0126077693 -2.6543422676
H -3.6761308184 1.7337255612 -2.8459910797
H -4.2470333535 3.1448408874 -0.8755355951
H -3.0335562788 2.8104458731 1.2697189312
H -1.2669271215 1.0878684129 1.4428246914
56
-1190.35636000
O -0.0489339669 0.9294979316 0.7431846572
N 0.8347100271 -0.0460780399 1.2680568255
C 1.9557368789 -0.3213451698 0.4762676112
O 2.7195065938 -1.2208431840 0.8091609635
C 2.2753245766 0.4999360805 -0.7432263237
C 2.8717069502 -0.1676349295 -1.8215860677
C 3.3310773044 0.5441697742 -2.9273116319
C 3.2303114103 1.9381476769 -2.9535720730
C 2.6624772630 2.6120658567 -1.8705875849
C 2.1764887067 1.8973785839 -0.7736312980
H 1.7273679815 2.4268702027 0.0586928259
H 2.5994798680 3.6973028552 -1.8763138353
H 3.6005273051 2.4962323272 -3.8097509637
H 3.7796820271 0.0139249360 -3.7631840410
H 2.9697072835 -1.2467568079 -1.7746028209
C 0.9896971418 -0.0029777779 2.7832552485
C 2.2824403225 0.7526761007 3.1536540158
H 3.1675123987 0.2530677087 2.7533253509
H 2.3832844510 0.7989963621 4.2436482617
H 2.2544867413 1.7811498617 2.7745243093
C 1.0380972424 -1.4460647487 3.3170893693
H 1.1545900507 -1.4261981196 4.4075569423
H 1.8740270336 -1.9926254550 2.8797085111
H 0.1097922774 -1.9739309152 3.0802062210
C -0.2053028472 0.7304566764 3.4077417530
H -0.2666603178 1.7706108532 3.0782410222
H -0.0693452837 0.7239954665 4.4946195397
H -1.1524433322 0.2359577225 3.1842137540
C -1.2172648072 0.4029741982 0.0546774163
H -1.8168071072 1.3091070757 -0.0285051990
N -0.8843789134 -0.0055072310 -1.3148658984
C -1.5346276854 0.6331676436 -2.5152946756
C -0.8425977872 0.0762333731 -3.7658873759
H 0.2275468685 0.2999546549 -3.7555047943
H -0.9614710510 -1.0063979406 -3.8366976582
H -1.2910689682 0.5397489249 -4.6511409996
C -1.3487568054 2.1608421756 -2.4618546166
H -1.7455196125 2.6020876997 -3.3826974187
H -0.2895592389 2.4215265748 -2.3818147440
H -1.8855620674 2.6256595257 -1.6278832596
C -3.0310001005 0.2605256029 -2.5263839310
H -3.5140203301 0.6786605884 -3.4169284905
H -3.1552129193 -0.8269170729 -2.5437437023
H -3.5568970085 0.6494159566 -1.6475941756
O -0.2094606716 -1.0856228363 -1.4664581214
C -2.0267702447 -0.6360969062 0.8053037168
C -1.6654136032 -1.9895540149 0.8851856514
C -2.4770680573 -2.8897360033 1.5773014537
C -3.6526465469 -2.4591498715 2.1959673806
C -4.0208365926 -1.1155705667 2.1182700440
C -3.2122361823 -0.2140742486 1.4257696657
H -3.5043003950 0.8327525265 1.3712380708
H -4.9353530625 -0.7685716186 2.5916270061
H -4.2793187779 -3.1671349704 2.7317844730
H -2.1870604033 -3.9357056480 1.6308535936
H -0.7621817427 -2.3290661201 0.3962656722

References

(1) https://www.kessil.com/downloads/science/PR160L_Intensity_Map_2.pdf

(2) Stoll, S.; Schweiger, A. EasySpin, a Comprehensive Software Package for Spectral Simulation and Analysis in EPR. *Journal of Magnetic Resonance* **2006**, *178* (1), 42–55. https://doi.org/10.1016/j.jmr.2005.08.013.

(3) https://github.com/DSakicLab/visualEPR

(4) Pracht, P.; Bohle, F.; Grimme, S. Automated Exploration of the Low-Energy Chemical Space with Fast Quantum Chemical Methods. *Phys. Chem. Chem. Phys.* **2020**, *22* (14), 7169–7192. https://doi.org/10.1039/C9CP06869D.

(5) Bannwarth, C.; Caldeweyher, E.; Ehlert, S.; Hansen, A.; Pracht, P.; Seibert, J.; Spicher, S.; Grimme, S. Extended Tight-Binding Quantum Chemistry Methods. *WIREs Comput Mol Sci* **2021**, *11* (2), e1493. https://doi.org/10.1002/wcms.1493.

(6) Bannwarth, C.; Ehlert, S.; Grimme, S. GFN2-xTB—An Accurate and Broadly Parametrized Self-Consistent Tight-Binding Quantum Chemical Method with Multipole Electrostatics and Density-Dependent Dispersion Contributions. *J. Chem. Theory Comput.* **2019**, *15* (3), 1652–1671. https://doi.org/10.1021/acs.jctc.8b01176.

(7) Becke, A. D. Density-Functional Thermochemistry. III. The Role of Exact Exchange. *The Journal of Chemical Physics* **1993**, *98* (7), 5648–5652. https://doi.org/10.1063/1.464913.

(8) Stephens, P. J.; Devlin, F. J.; Chabalowski, C. F.; Frisch, M. J. Ab Initio Calculation of Vibrational Absorption and Circular Dichroism Spectra Using Density Functional Force Fields. *J. Phys. Chem.* **1994**, *98* (45), 11623–11627. https://doi.org/10.1021/j100096a001.

(9) Ditchfield, R.; Hehre, W. J.; Pople, J. A. Self-Consistent Molecular-Orbital Methods. IX. An Extended Gaussian-Type Basis for Molecular-Orbital Studies of Organic Molecules. *The Journal of Chemical Physics* **1971**, *54* (2), 724–728. https://doi.org/10.1063/1.1674902.

(10) Grimme, S. Semiempirical Hybrid Density Functional with Perturbative Second-Order Correlation. *The Journal of Chemical Physics* **2006**, *124* (3), 034108. https://doi.org/10.1063/1.2148954.

(11) Neese, F.; Schwabe, T.; Grimme, S. Analytic Derivatives for Perturbatively Corrected "Double Hybrid" Density Functionals: Theory, Implementation, and Applications. *The Journal of Chemical Physics* **2007**, *126* (12), 124115. https://doi.org/10.1063/1.2712433.

(12) Curtiss, L. A.; Redfern, P. C.; Raghavachari, K.; Rassolov, V.; Pople, J. A. Gaussian-3 Theory Using Reduced Mo/Ller-Plesset Order. *The Journal of Chemical Physics* **1999**, *110* (10), 4703–4709. https://doi.org/10.1063/1.478385.

(13) Grimme, S.; Antony, J.; Ehrlich, S.; Krieg, H. A Consistent and Accurate *Ab Initio* Parametrization of Density Functional Dispersion Correction (DFT-D) for the 94 Elements H-Pu. *The Journal of Chemical Physics* **2010**, *132* (15), 154104. https://doi.org/10.1063/1.3382344.

(14) Vrček, I. V.; Šakić, D.; Vrček, V.; Zipse, H.; Biruš, M. Computational Study of Radicals Derived from Hydroxyurea and Its Methylated Analogues. *Org. Biomol. Chem.* **2012**, *10* (6), 1196–1206. https://doi.org/10.1039/C1OB06594G.

(15) Hermosilla, L.; Calle, P.; García De La Vega, J. M.; Sieiro, C. Density Functional Theory Study of ^{14}N Isotropic Hyperfine Coupling Constants of Organic Radicals. *J. Phys. Chem. A* **2006**, *110* (50), 13600–13608. https://doi.org/10.1021/jp064900z.

(16) Hermosilla, L.; Vega, J. M. G. D. L.; Sieiro, C.; Calle, P. DFT Calculations of Isotropic Hyperfine Coupling Constants of Nitrogen Aromatic Radicals: The Challenge of Nitroxide Radicals. *J. Chem. Theory Comput.* **2011**, *7* (1), 169–179. https://doi.org/10.1021/ct1006136.

(17) Gaussian 16, Revision C.01, M. J. Frisch, G. W. Trucks, H. B. Schlegel, G. E. Scuseria, M. A. Robb, J. R. Cheeseman, G. Scalmani, V. Barone, G. A. Petersson, H. Nakatsuji, X. Li, M. Caricato, A. V. Marenich, J. Bloino, B. G. Janesko, R. Gomperts, B. Mennucci, H. P. Hratchian, J. V. Ortiz, A. F. Izmaylov, J. L. Sonnenberg, D. Williams-Young, F. Ding, F. Lipparini, F. Egidi, J. Goings, B. Peng, A. Petrone, T. Henderson, D. Ranasinghe, V. G. Zakrzewski, J. Gao, N. Rega, G. Zheng, W. Liang, M. Hada, M. Ehara, K. Toyota, R. Fukuda, J. Hasegawa, M. Ishida, T. Nakajima, Y. Honda, O. Kitao, H. Nakai, T. Vreven, K. Throssell, J. A. Montgomery, Jr., J. E. Peralta, F. Ogliaro, M. J. Bearpark, J. J. Heyd, E. N. Brothers, K. N. Kudin, V. N. Staroverov, T. A. Keith, R. Kobayashi, J. Normand, K. Raghavachari, A. P. Rendell, J. C. Burant, S. S. Iyengar, J. Tomasi, M. Cossi, J. M. Millam, M. Klene, C. Adamo, R. Cammi, J. W. Ochterski, R. L. Martin, K. Morokuma, O. Farkas, J. B. Foresman, and D. J. Fox, Gaussian, Inc., Wallingford CT, 2016.

(18) Cluster Isabella, University of Zagreb University Computing Centre - SRCE; Cluster Supek, University of Zagreb University Computing Centre - SRCE, HR-ZOO, KK.01.1.1.08.0001, EU funded within OPCC for Republic of Croatia

(19) PharmInova project, University of Zagreb Faculty of Pharmacy and Biochemistry, KK.01.1.1.02.0021, EU funded by the European Regional Development Fund

(20) Tang, X.; Huang, L.; Qi, C.; Wu, X.; Wu, W.; Jiang, H. Copper-Catalyzed Sulfonamides Formation from Sodium Sulfinates and Amines. *Chem. Commun.* **2013**, *49* (54), 6102–6104. https://doi.org/10.1039/C3CC41249K.

(21) Marcotullio, M. C.; Campagna, V.; Sternativo, S.; Costantino, F.; Curini, M. A New, Simple Synthesis of N -Tosyl Pyrrolidines and Piperidines. *Synthesis* **2006**, *16*, 2760–2766. https://doi.org/10.1055/s-2006-942488.

(22) Esch, P. M.; Boska, I. M.; Hiemstra, H.; de Boer, R. F.; Speckamp, W. N. Tin Tetrachloride-Induced π-Cyclizations of Glycine Cation Equivalents to Substituted Pipecolic

Acid Derivatives. *Tetrahedron* **1991**, *47* (24), 4039–4062. https://doi.org/10.1016/S0040-4020(01)86443-7.

(23) Jones, A. D.; Knight, D. W.; Hibbs, D. E. A Stereochemically Flexible Approach to Pyrrolidines Based on 5-Endo-Trig Iodocyclisations of Homoallylic Sulfonamides. *J. Chem. Soc., Perkin Trans. 1* **2001**, No. 10, 1182–1203. https://doi.org/10.1039/B008537P.

(24) Lee, S.; Lei, H.; Rovis, T. A Rh(III)-Catalyzed Formal [4+1] Approach to Pyrrolidines from Unactivated Terminal Alkenes and Nitrene Sources. *J. Am. Chem. Soc.* **2019**, *141* (32), 12536–12540. https://doi.org/10.1021/jacs.9b07012.

(25) Chang, D.; Zhao, R.; Wei, C.; Yao, Y.; Liu, Y.; Shi, L. Sulfonamide-Directed Chemo- and Site-Selective Oxidative Halogenation/Amination Using Halogenating Reagents Generated in Situ from Cyclic Diacyl Peroxides. *J. Org. Chem.* **2018**, *83* (6), 3305–3315. https://doi.org/10.1021/acs.joc.8b00243.

(26) Novaes, L. F. T.; Ho, J. S. K.; Mao, K.; Liu, K.; Tanwar, M.; Neurock, M.; Villemure, E.; Terrett, J. A.; Lin, S. Exploring Electrochemical C(Sp3)-H Oxidation for the Late-Stage Methylation of Complex Molecules. *J Am Chem Soc* **2022**, *144* (3), 1187–1197. https://doi.org/10.1021/jacs.1c09412.

(27) Bosnidou, A. E.; Duhamel, T.; Muñiz, K. Detection of the Elusive Nitrogen-Centered Radicals from Catalytic Hofmann–Löffler Reactions. *Eur. J. Org. Chem.* **2020**, *2020* (40), 6361–6365. https://doi.org/10.1002/ejoc.201900497.

(28) Buettner, G. R. Spin Trapping: ESR Parameters of Spin Adducts 1474 1528V. *Free Radical Biology and Medicine* **1987**, *3* (4), 259–303. https://doi.org/10.1016/S0891-5849(87)80033-3.

(29) Liu, R.; Li, J.; Sun, J.; Liu, X.; Qu, S.; Li, P.; Zhang, B. Generation and Reactivity of Amidyl Radicals: Manganese-Mediated Atom-Transfer Reaction. *Angew Chem Int Ed* **2020**, *59* (11), 4428–4433. https://doi.org/10.1002/anie.201913042.

(30) Chang, D.; Zhao, R.; Wei, C.; Yao, Y.; Liu, Y.; Shi, L. Sulfonamide-Directed Chemo- and Site-Selective Oxidative Halogenation/Amination Using Halogenating Reagents Generated in Situ from Cyclic Diacyl Peroxides. *J. Org. Chem.* **2018**, *83* (6), 3305–3315. https://doi.org/10.1021/acs.joc.8b00243.

(31) Herron, A. N.; Hsu, C.-P.; Yu, J.-Q. δ-C–H Halogenation Reactions Enabled by a Nitrogen-Centered Radical Precursor. *Org. Lett.* **2022**, *24* (20), 3652–3656. https://doi.org/10.1021/acs.orglett.2c01261.

(32) Šakić, D.; Zubčić, G. Additional data for "Regioselective Rearrangement of Nitrogen- and Carbon-centered Radical Intermediates in Hofmann–Löffler–Freytag Reaction" [Data set]. *Zenodo* **2023**, https://doi.org/10.5281/zenodo.10161638

(33) National Institute of Environmental Healtsh Sciences Spin-trap Database, accessed 2024. https://tools.niehs.nih.gov/stdb/index.cfm

(34) Tian, Lu; Feiwu, Chen. Multiwfn: A Multifunctional Wavefunction Analyzer, *J. Comput. Chem.* **2012**, *33*, 580-592. https://doi.org/10.1002/jcc.22885

(35) Gibert, Andrew. IQmol 3.1.11 – molecular visualizer. **2023**. http://iqmol.org
